# Minimal Functional Sites Allow a Classification of Zinc Sites in Proteins

**DOI:** 10.1371/journal.pone.0026325

**Published:** 2011-10-17

**Authors:** Claudia Andreini, Ivano Bertini, Gabriele Cavallaro

**Affiliations:** 1 Magnetic Resonance Center (CERM), University of Florence, Sesto Fiorentino, Italy; 2 Department of Chemistry, University of Florence, Sesto Fiorentino, Italy; Weizmann Institute of Science, Israel

## Abstract

Zinc is indispensable to all forms of life as it is an essential component of many different proteins involved in a wide range of biological processes. Not differently from other metals, zinc in proteins can play different roles that depend on the features of the metal-binding site. In this work, we describe zinc sites in proteins with known structure by means of three-dimensional templates that can be automatically extracted from PDB files and consist of the protein structure around the metal, including the zinc ligands and the residues in close spatial proximity to the ligands. This definition is devised to intrinsically capture the features of the local protein environment that can affect metal function, and corresponds to what we call a minimal functional site (MFS). We used MFSs to classify all zinc sites whose structures are available in the PDB and combined this classification with functional annotation as available in the literature. We classified 77% of zinc sites into ten clusters, each grouping zinc sites with structures that are highly similar, and an additional 16% into seven pseudo-clusters, each grouping zinc sites with structures that are only broadly similar. Sites where zinc plays a structural role are predominant in eight clusters and in two pseudo-clusters, while sites where zinc plays a catalytic role are predominant in two clusters and in five pseudo-clusters. We also analyzed the amino acid composition of the coordination sphere of zinc as a function of its role in the protein, highlighting trends and exceptions. In a period when the number of known zinc proteins is expected to grow further with the increasing awareness of the cellular mechanisms of zinc homeostasis, this classification represents a valuable basis for structure-function studies of zinc proteins, with broad applications in biochemistry, molecular pharmacology and *de novo* protein design.

## Introduction

Zinc is an essential element for living organisms. While this statement applies to several other metals, the pervasive occurrence of zinc in biological processes is unique. This mostly results from the association of zinc with an impressive variety of proteins involved in a wide range of physiological activities [1], [2]. Estimates of zinc proteomes in various organisms indicated that the amount of genes encoding zinc proteins varies from 4% to 10% of the genome and that approximately 3,000 zinc proteins are encoded in the human genome [3], [4]. Zinc enzymes in which zinc plays a catalytic role are present across all living organisms and constitute the largest share of prokaryotic zinc proteins. The main reason for the selection of zinc as a catalytic cofactor lies in its distinctive chemical properties, which combine Lewis acid strength, lack of redox reactivity, and fast ligand exchange [5]. As a reflection of the widespread use and the remarkable versatility of zinc in biological catalysis, zinc enzymes are present in all six major classes of enzymes (oxidoreductases, transferases, hydrolases, lyases, isomerases, and ligases) [5], [6]. In eukaryotes but not in prokaryotes the majority of zinc proteins function in the regulation of gene expression, pointing out that the biological importance of zinc increased as increasingly complex cellular, and in particular multi-cellular, systems evolved. Many of these proteins contain one or more so-called zinc fingers, which are small protein domains stabilized by a zinc ion playing a structural role [7]. Originally discovered as DNA-binding motifs, zinc fingers are now known to mediate protein-RNA and protein-protein interactions [8]. Other zinc proteins whose importance emerged more recently include proteins for zinc sensing, transport, buffering, and storage. As the molecular mechanisms of cellular zinc homeostasis are just beginning to be elucidated, the number of these proteins and thus the size of zinc proteomes is likely to be larger than what is currently realized [9], [10].

Given the above considerations, the wealth of studies in which zinc proteins were analysed appears to be adequate to the “sphere of influence” of zinc on biological systems. Indeed, the size of this sphere is so large that even the largest surveys were necessarily conducted on subsets of zinc proteins, e.g., enzymes [11] or zinc finger proteins [12]. In many of these studies, attempts were made to classify zinc sites in proteins and relate their function to properties such as coordination number and geometry, and the type of zinc ligands [13]–[16]. A structural classification of zinc fingers dating back to 2003 was developed based on the spatial arrangement of secondary structure elements around the zinc sites [17].

In this work we propose a new, comprehensive classification of zinc sites in proteins with known structures. It is the opinion of the authors that this effort is timely, as an up-to-date classification of zinc sites appears to be needed at a time when the abovementioned sphere of influence of zinc is possibly going through a further expansion. This classification is based on the widely recognized concept that metal sites in proteins are not adequately described, and thus classified, only on the basis of the metal ligands (i.e., the metal coordination sphere) [18]–[20]. Indeed, models of metal sites in proteins that include only the metal ligands may not be sufficiently accurate to reproduce biochemical functions. The surroundings of the coordination sphere must also be taken into account in order to define what can be thought of as the minimal environment determining metal function, or the “minimal functional site” (MFS). The precise definition of MFSs, however, is not obvious. In our approach we define them by means of three-dimensional templates that encompass the structure of the protein matrix around the metal well beyond its coordination sphere, by including all residues within 5 Å from any metal-binding residue [21]. This definition is most convenient in that (i) it incorporates the characteristics of the protein environment that affect metal function, (ii) it can be implemented in automated routines for building the templates from PDB structures, and (iii) it allows the comparison of metal sites via structural alignment, thereby providing a basis for classification. Specifically, the use of a distance threshold of 5 Å for building the MFS templates appears to be an ideal compromise between the need of including all residues that interact with metal ligands (also accounting for the various accuracy degrees of PDB structures) and the need of describing metal sites only in terms of their local structure (i.e., without extending too far from the metal at the risk of detecting similarities that are not relevant to the sites).

Our results indicate that over 77% of zinc sites can be accounted for in terms of ten structural motifs conserved across protein superfamilies, and an additional 16% in terms of more general but also useful structural descriptors. We also analyze and discuss correlations between the function performed by zinc in a protein and the structural motif as well as the amino acid residues used to bind it, thereby providing a valuable reference for future studies aimed at unveiling the subtleties of the structure-function relationships in zinc proteins.

## Methods

All the available protein structures containing zinc were downloaded from the Protein Data Bank (PDB) [22] by searching for entries that contained any of the following non-standard PDB residues: BAZ, BOZ, DAZ, DOZ, DTZ, HE5, HES, ZEM, ZH3, ZN, ZN2, ZN3, ZNH, ZNO, and ZO3. At the time of the download (January 2011), these were all the non-standard PDB residues containing at least one zinc atom as described in the Chemical Component Dictionary (http://www.wwpdb.org/ccd.html). Zinc sites in each structure were identified by taking all the zinc atoms in the structure, and considering zinc atoms at a distance of less than 5.0 Å from one another as belonging to the same site. A structural template was built for each site by extracting the PDB coordinates of all the zinc atoms in the site, of the zinc ligands, and of the protein residues in spatial proximity of the zinc ligands. Specifically, zinc ligands were defined as those (protein or non-protein) residues having a non-hydrogen atom at a distance of less than 3.0 Å from any zinc atom in the site, and spatially proximal residues were defined as those having a non-hydrogen atom at a distance of less than 5.0 Å from any atom of a zinc-binding residue. Each of these templates defines a zinc minimal functional site (MFS).

Zinc sites were grouped based on the CATH [23] (http://www.cathdb.info, version 3.3) and SCOP [24] (http://scop.mrc-lmb.cam.ac.uk/scop, release 1.75) classifications of the protein domains containing the zinc-binding residues of each site. Specifically, each site was assigned to both a CATH and a SCOP superfamily, and sites assigned either to the same CATH or to the same SCOP superfamily were grouped together. The superfamily level is common to both the CATH (where it corresponds to a four-digit code) and the SCOP (where it corresponds to a three-digit code) hierarchical classification schemes, and groups together similar folds for which there is good evidence of common ancestry. The sites of proteins that have not yet been included in the CATH or in the SCOP database were also assigned to an existing CATH and/or SCOP superfamily or to an “unclassified” superfamily, using a procedure described in [21]. Zinc sites placed in the same superfamily were compared against one another in an all-versus-all fashion using the structural alignment program FAST, and clustered by single linkage clustering using a threshold similarity score of 1.5 [21], [25]. By this clustering, structurally distinct sites present in the same protein domain (e.g., the catalytic and the structural zinc site of alcohol dehydrogenase, PDB code 6adh [26]) were placed into different groups. The relevant literature was examined to annotate the functions of grouped zinc sites and to identify non-physiological zinc sites, such as sites in metalloproteins where zinc has been substituted for the native metal ion (e.g., cytochrome *c*, PDB code 1m60 [27]), or non-specific sites due to adventitious binding of zinc to the protein (e.g., acyl carrier protein, PDB code 1l0h [28]).

A set of representative zinc sites was selected by choosing the PDB structure in each group with the highest resolution (unless the highest resolution structure was not appropriate, e.g., due to engineered mutations of the zinc ligands). This set was used to analyse the coordination sphere of zinc sites as described in [21]. The coordination geometry of four-coordinated zinc ions in this set was calculated by FindGeo, an in-house developed tool that automatically determines the best-fit geometry among a number of possible ideal geometries. The representative zinc sites were compared against one another in an all-versus-all fashion using FAST, and clustered by single linkage clustering using progressively lower threshold similarity scores, corresponding to the 99^th^, 98^th^, 97^th^, 96^th^, and 95^th^ percentile of all non-zero similarity scores obtained from FAST (i.e., the score below which 99%, 98%, 97%, 96%, and 95% of all scores fall, respectively). The clusters built with the 99^th^ percentile threshold were used as the reference set of clusters, and their composition was compared to that of the clusters built with lower thresholds with the aim of extending their coverage. The composition of clusters was then manually refined. For each cluster, the amino acid sequences of the protein chains containing the sites in the cluster were aligned using the program T-Coffee [29].

To countercheck the correctness of the use of a 5.0 Å value as the distance threshold to build zinc MFS templates, we re-built the templates of representative zinc sites using other different values (i.e., 3.0, 4.0, 6.0, 7.0, 8.0, 9.0, and 10.0 Å), and repeated the above procedure including all-versus-all comparison and clustering. Each set of clusters built with the 99^th^ percentile threshold was then compared with the reference set of clusters. The comparison confirmed that the 5.0 Å value represents an optimal choice for the size of structural templates, although the 4.0 Å value yields comparable results (Table S5).

## Results and Discussion

### Occurrence, physiological relevance and functions of zinc sites in PDB structures

At the time of the present study, the PDB contained 6170 protein structures having at least one zinc atom (referred to as Zn-structures hereafter), for a total of 15763 zinc sites (Zn-sites hereafter). As described in the Methods section, Zn-sites found in protein domains that belong to the same superfamily according to either the CATH or the SCOP classification were grouped together, and, subsequently, structurally distinct sites present in the same domain were divided into different groups. We define the groups formed by this procedure as superfamilies of Zn-sites (Zn-superfamilies hereafter). As proteins classified in the same CATH or SCOP superfamily are not only structurally but also functionally related, Zn-sites included in the same Zn-superfamily were assumed to have the same general function (i.e., catalytic, structural, regulatory, or substrate), despite the specific functions of the proteins that contain them may vary, especially in the largest CATH and SCOP superfamilies. The general functions of Zn-sites were assigned by inspection of the available literature. Concurrently, non-physiological Zn-sites were identified and discarded, resulting in the removal of 4832 Zn-sites and 1288 Zn-structures from the original dataset (a list of the Zn-sites removed is given in Table S1). This result highlights the importance of considering the physiological relevance of zinc atoms (and of metal atoms in general) bound to proteins, as more than 20% of PDB structures containing zinc are not in fact zinc proteins.

The 10931 physiological Zn-sites (found in a total of 4882 Zn-structures) that formed our final dataset were grouped into 367 Zn-superfamilies. A summary of the relevant information on Zn-superfamilies is given in Table S2, and the lists of Zn-sites belonging to each Zn-superfamily are given in Table S3. The number of Zn-sites included in a Zn-superfamily is highly variable, ranging from only one to 758. However, as this number depends on the redundancy of the PDB, a better measure of the size of a Zn-superfamily is the number of non-redundant proteins (defined here as proteins with sequence identity lower than 50%) in which the Zn-sites of the Zn-superfamily were found. Using this criterion, the large majority (about 86%) of Zn-superfamilies map to five or less non-redundant proteins, and only a few (about 7%) map to ten or more non-redundant proteins (Table S2).

**Figure 1:**
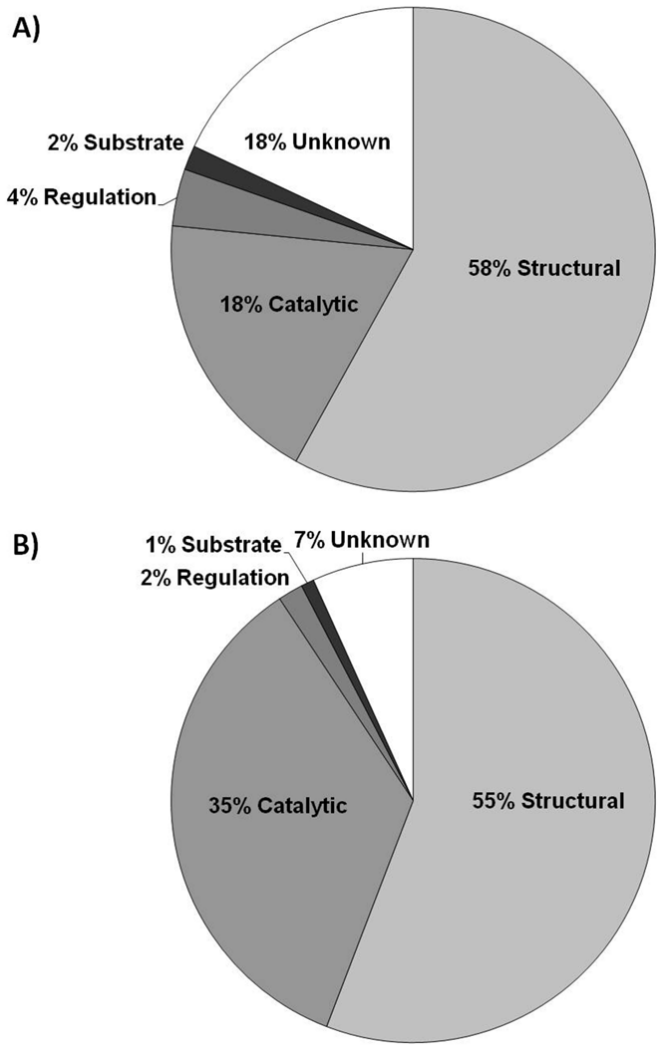
Pie charts showing the functions of zinc sites in (A) Zn-superfamilies, and (B) non-redundant zinc proteins (defined as proteins with sequence identity lower than 50%). The higher proportion of unknown zinc sites and the lower proportion of catalytic zinc sites in (A) with respect to (B) reflect the fact that Zn-superfamilies with unknown functions are generally small (consisting on average of 1.5 non-redundant proteins), whereas those with catalytic functions are generally larger (consisting on average of 7.5 non-redundant proteins).

On the basis of literature analysis, 301 Zn-superfamilies could be assigned one of the four abovementioned general functions (Table S2 and Figure 1). The most widespread function was structural (213 cases), followed by catalytic (68 cases), regulatory (14 cases), and substrate (6 cases). These results come as no surprise as zinc has been long known to stabilize the tertiary and/or the quaternary structure of proteins (structural function), and to occur in the active site of many various enzymes (catalytic function). On the other hand, the cellular pathways of zinc homeostasis and of zinc-mediated signalling have only recently begun to emerge, and a relatively low number of proteins is known in which zinc acts as a regulatory element (regulatory function) or zinc is bound to be transported and/or stored (substrate function). Furthermore, as the latter two functions usually involve a transient binding of zinc to the protein, such Zn-sites are often elusive to catch during protein structure determination, which is another reason for their scarcity in the PDB.

### Zinc coordination: trends and exceptions

**Figure 2:**
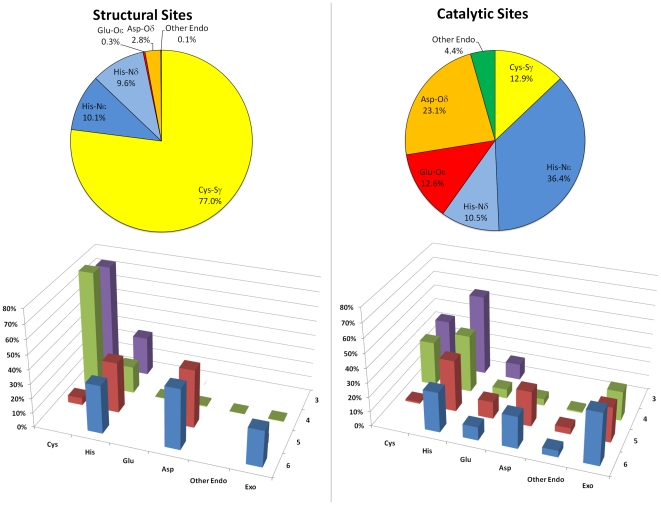
Zinc ligands found in the representative Zn-sites with structural (left) and catalytic (right) functions, overall (pie charts, top) and as a function of the coordination number (histograms, bottom). “Other endo” includes all endogenous (i.e., provided by the protein) ligands different from those explicitly indicated, and “Exo” includes all exogenous (i.e., non-protein) ligands. The histogram for structural sites does not take into account the single case of coordination number seven (PDB code 2faw [44]).

To have a survey of the modes of zinc coordination found in proteins, a representative Zn-site was selected for each Zn-superfamily (Table S2). These sites are most often mononuclear (about 93% of the cases), with the coordination number of individual zinc atoms varying from three to seven, and being four in most cases (about 76%). The coordination geometry of four-coordinated zinc atoms (as determined automatically from the structures using an in-house developed tool) is most commonly tetrahedral (87% of the cases). In the remaining 13% of the cases, the geometry can be generally viewed as distorted tetrahedral, although our tool indicated that it can also be described as trigonal bipyramidal (10%) or square pyramidal (3%) with a vacant coordination position. Some of these cases may therefore represent structures where a fifth zinc ligand has been overlooked. In no case a square planar geometry was observed. When the representative Zn-sites are examined on a per-function basis, the correlation between the coordination features of the site and the specific role that zinc plays in the protein becomes apparent, highlighting the capability of the protein matrix to modulate metal function (Figure 2).

In structural sites, zinc is by far most frequently coordinated by four ligands (94% of the cases), which are all provided by the protein except for the only cases of human interferon beta (PDB code 1au1 [30]), hexameric insulin (PDB code 1ev6 [31]), and Shank SAM domain (PDB code 2f44 [32]), where an exogenous ligand is also present. Almost all protein ligands in four-coordinate sites are Cys (80%) and His (19%), the only exceptions being Asp/Glu and Ser. In more detail, in more than 96% of these sites at least two of the four protein ligands are Cys, which are generally preferred to other residues by virtue of their capability to transfer negative charge to the Zn^2+^ ion, thus forming stronger bonds [16]. In the few cases (8 out of 213) where a structural zinc ion is coordinated by more than four ligands, however, Cys are practically absent, and coordination is accomplished by a mixture of His and Asp/Glu (which are often bidentate), sometimes accompanied by backbone N and O atoms and water molecules.

At variance with structural sites, catalytic zinc sites most often contain at least one exogenous ligand, and display a higher variability in their coordination. This observation can be traced back to the mechanism of action of zinc in enzymatic catalysis, where it is involved in substrate binding and activation, and can vary coordination number and geometry [5], [33]. These variations are mainly due to changes in the bonds that zinc forms with the exogenous ligands (e.g., enzyme substrate/product), whereas protein ligands generally remain unchanged. The number of protein ligands in catalytic zinc sites is most frequently three (49% of the cases), followed by four (43%) and five (9%), and the most common ligands are His (47%) and Asp/Glu (36%), whereas Cys are relatively rare (13%). In addition, a preference appears to exist for the Nε2 atom of His to act as the ligand atom rather than Nδ1 (the Nε2/Nδ1 ratio is about 3.5). This tendency has been previously noted, and attributed to the stricter steric requirements imposed by Nδ1 ligation with respect to Nε2 [13]. The use of Cys as a ligand, instead, appears to be linked to the coordination number of zinc, in that when zinc is bound by more than four ligands, Cys residues are either only one or absent (a situation that also occurs in structural sites, as noted above). Furthermore, in the 13 catalytic sites that contain two or more Cys ligands, the coordination number of zinc does not appear to be higher than four at any state of the enzymatic reaction. This observation has been drawn upon the analysis of all the sites included in the superfamilies of these Zn-sites, which represent all the available structural information on the various coordination states accessible to zinc in these enzymes. Out of 261 structures inspected, only two structures of blasticidin S deaminase (PDB codes 1wn6 and 2z3i, the latter being a single mutant of the former), which have been determined within the same study, show a five-coordinate zinc, which would occur in a putative reaction intermediate [34]. Indeed, this observation still holds when all the catalytic sites in our dataset (i.e., a total of 4524 sites in 2404 PDB structures) are taken into account. Also, the analysis of the enzymatic reactions collected in Metal-MACiE (a database containing information on catalytic metal ions) [35] shows that zinc ions coordinated by two or more Cys have at most four ligands at any reaction step. This leads to two considerations. First, the presence of at least two Cys in the coordination sphere of zinc, which has been previously taken as a criterion to discriminate between structural and catalytic zinc sites [16], could be a determinant of the accessible coordination states, and thus of the mechanism of action, of the metal. Namely, two Cys ligands would be sufficient to prevent zinc from extending its coordination number above four. This looks much like a requirement in structural sites, where zinc must be rigidly fixed, and Cys are in fact predominant. Still, Cys may well be used as the predominant ligands in catalytic sites as well, as long as the reaction mechanism involves a zinc coordination number not higher than four. In this respect, we suggest that the number of Cys ligands may be a discriminating factor in the contentious mechanism of the zinc-dependent medium-chain alcohol dehydrogenase (ADH) superfamily of enzymes. In the classical mechanism, zinc is believed to maintain a tetrahedral coordination during the entire catalytic process [36]. However, a five-coordinate zinc intermediate has been proposed to occur based on studies on human sorbitol dehydrogenase [37] and *Haloferax mediterranei* glucose dehydrogenase [38]. As the latter enzymes contains a single or no Cys ligand whereas the majority of these enzymes contain two, it is possible that ADHs with two Cys ligands follow the classical mechanism, while ADHs with one or no Cys ligand follow the other one. The second consideration is that predictive rules using the number of Cys ligands to predict zinc function could be improved by taking into account the coordination number as well. For instance, the prediction that every zinc bound by one or zero Cys residues is catalytic, as proposed in [16], should not be applied when the zinc coordination number is higher than four, as in this case Cys ligands appear to be one or zero in both structural and catalytic sites.

As previously mentioned, regulatory and substrate zinc sites for which a structure is available are still a few. Nevertheless, some trends in their coordination features can be recognized, although they should be regarded with some caution. Regulatory sites appear to resemble catalytic sites in their ligand preferences, as the most frequent protein ligands are His and Asp/Glu (35% and 29%, respectively), and exogenous ligands can be also found (in 5 out of 14 cases). Cys ligands are less uncommon than in catalytic sites (18%), yet they appear to be predominant only in sites specifically designed to sense zinc (exemplified by the transcriptional regulator ZntR, PDB code 1q08 [39]), or to act as redox switches involving thiol-disulfide redox reactions (exemplified by the bacterial heat shock protein Hsp33, PDB code 1vzy [40]). In substrate sites, a clear difference exists between those found in zinc trafficking proteins and those found in zinc storage proteins. The former also show a preference for His and Asp/Glu (35% and 40%, respectively) with respect to Cys (10%), and can contain exogenous ligands (present in one out of three cases) within coordination spheres that include from three to five ligands. In storage proteins (metallothioneins), instead, zinc is invariably four-coordinated by Cys (88%) and, much less frequently, His (12%). These sites are thus more similar to structural ones, although they typically contain clusters of zinc ions which are very unusual among structural sites (about 2% of the cases).

### Clustering of representative zinc sites

The representative Zn-sites, each selected from a different Zn-superfamily, were compared against one another with the aim of grouping those that have similar structures into clusters (Zn-clusters hereafter). The comparison was performed by structural alignment of the MFS templates describing the local environment of the representative Zn-sites (see Methods). In this way, zinc-binding motifs that are common to different Zn-superfamilies were identified, thereby allowing zinc sites to be classified into more general types on a purely structural basis. At the same time, these shared motifs can be regarded as potential examples of convergent evolution, in which proteins belonging to different superfamilies independently evolved the same kind of zinc-binding site.

**Figure 3:**
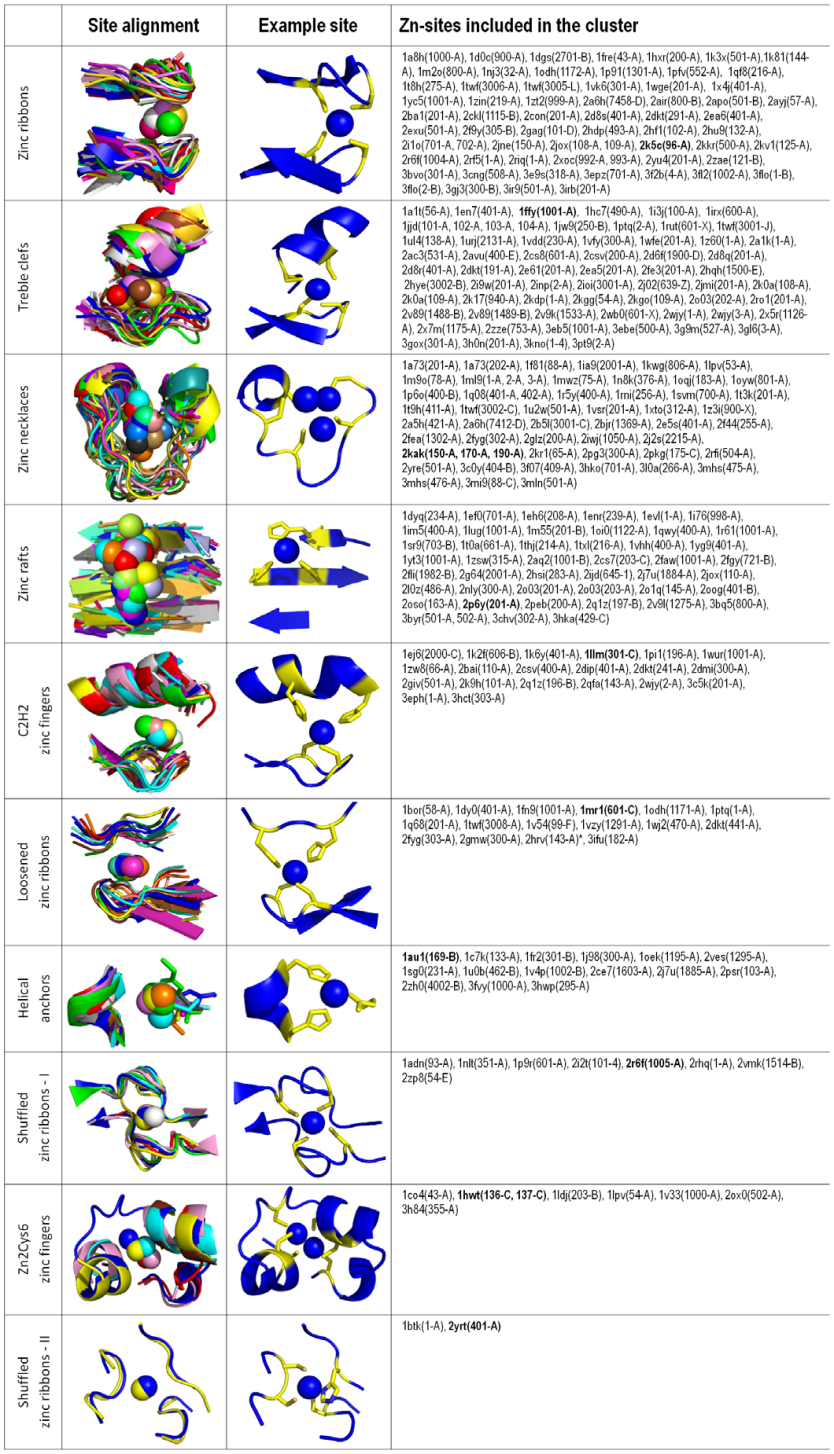
Structure and composition of the Zn-clusters identified. For each cluster, the superimposition of the structures of the representative Zn-sites included in the cluster, the picture of an example structure (shown as a cartoon representation with zinc atoms as blue spheres and zinc ligands as blue sticks), and the list of the representative Zn-sites included in the cluster are given (with the example structure in bold). Each Zn-site is identified by the PDB code and (in parentheses) the residue number(s) and the chain identifier(s) of the zinc atom(s) in the site.

A total of 10 Zn-clusters were identified (Table 1 and Figure 3), which together comprise 77% of Zn-superfamilies (i.e., 284 of 367), and cover 75% of non-redundant zinc proteins (i.e., 926 of 1233). In terms of size, there are four Zn-clusters that can be regarded as large (containing 61, 61, 49, and 45 Zn-superfamilies, respectively), three that can be regarded as medium (containing 20, 16, and 15 Zn-superfamilies, respectively), and three that can be regarded as small (containing 8, 7, and 2 Zn-superfamilies, respectively). Zn-clusters generally contain sites that have the same function (with a very few exceptions which will be discussed later), and most of them contain structural sites. Specifically, almost 90% of the Zn-superfamilies with a structural function were included in a cluster. This was also the case for the majority of the Zn-superfamilies with a substrate (67%) or unknown (71%) function, and for about a half of the Zn-superfamilies with a catalytic (51%) or regulatory (50%) function. These data indicate that structural zinc sites are built around a limited range of motifs, some of which are especially widespread, while the other zinc sites display a wider variety of local structures. Zn-clusters are discussed in more detail in the following (a schematic picture of the structures of the representative Zn-sites included in each Zn-cluster is given in Table S4).

**Table 1:** Summary of the Zn-clusters identified, showing the number of representative Zn-sites (i.e., of Zn-superfamilies) included in each cluster, their functions, and the average sequence identity of the protein chains that contain those Zn-sites.

Zn-cluster	# of sites	Structural function	Catalytic function	Regulatory function	Substrate function	Unknown function	Average sequence identity
**Zinc ribbons**	61	48	0	0	0	13	23±6%
**Treble clefs**	61	55	0	0	1	5	24±6%
**Zinc necklaces**	49	32	1	2	2	12	22±5%
**Zinc rafts**	45	7	23	3	1	11	21±5%
**C2H2 zinc fingers**	20	19	1	0	0	0	22±6%
**Loosened zinc ribbons**	16	15	0	1	0	0	21±5%
**Helical anchors**	15	1	9	1	0	4	21±5%
**Shuffled zinc ribbons – type I**	8	6	1	0	0	1	27±8%
**Zn2Cys6 zinc fingers**	7	7	0	0	0	0	23±6%
**Shuffled zinc ribbons – type II**	2	1	0	0	0	1	19%

### *1*. Zinc ribbons

The Zn-sites included in this cluster have a structure that consists of two β-hairpins providing two zinc ligands each, with the axes of the β-hairpins oriented nearly perpendicular to each other. This structure is classically referred to as a “zinc ribbon” [17], therefore we use this term to indicate this cluster. Each β-hairpin most often harbours two Cys ligands (86% of the cases), and the spacing between two zinc ligands on a β-hairpin is most commonly two residues (75% of the cases). Almost all the zinc ribbon structures in the cluster can be entirely superimposed, as the mutual orientation of the two β-hairpins is highly conserved across them. The only exception is represented by a Zn-site of human DNA (cytosine-5)-methyltranferase 1 (PDB code 3epz), whose β-hairpins, despite having perpendicular axes like the other zinc ribbons, are oriented in a different way, i.e. by superimposing the N-terminal β-hairpins, the C-terminal β-hairpins do not overlap but are rotated by approximately 180 degrees with respect to each other (and vice versa). This latter site is not shown in the structural alignment of Figure 3, and was classified among zinc ribbons upon visual inspection.

In terms of function, 48 of the 61 Zn-superfamilies included in this cluster have a structural role, and the remaining 13 have no known function. It is therefore reasonable to predict that these latter 13 Zn-superfamilies also have a structural function.

### *2*. Treble clefs

The term “treble clef” that we use to indicate this cluster denotes a structural motif formed by an N-terminal β-hairpin and a C-terminal α-helix, which provide two zinc ligands each [17]. The majority of the Zn-sites belonging to the cluster (i.e., 45 out of 61) indeed conform to this definition, whereas in 6 sites the β-hairpin and the α-helix elements are permuted, i.e., the α-helix is N-terminal and the β-hairpin is C-terminal (PDB codes 1hc7, 1jw9, 2ioi, 2j02, 2k0a, and 2v9k). The remaining Zn-sites in the cluster represent variants that do not strictly fall within the definition given above (e.g., the β-hairpin is replaced by a loop in 2ac3 and 3g9m), but can be closely superimposed to classical treble clefs. The β-hairpin and the α-helix are most often oriented with their axes approximately parallel to each other, however their relative orientation can vary depending on the specific arrangement of the zinc ligands within these elements. For example, in a Zn-site of yeast RNA polymerase II (PDB code 1twf [41]) the two zinc ligands on the α-helix are adjacent in the sequence (whereas in 72% of the cases they are separated by two residues), thereby enforcing a configuration where the axes of the β-hairpin and the α-helix are almost perpendicular. This and a few other Zn-sites (PDB codes 1irx, 1jw9, 2ioi, 2j02, and 2x7m) cannot thus be entirely superimposed on the other treble clefs, and were classified as such by visual inspection.

Regarding the function, all the Zn-superfamilies of the cluster have a structural role except for that of the cyanobacterial metallothionein SmtA (which has a substrate function) and for 5 Zn-superfamilies with unknown functions, which can thus be predicted to play a structural role as well.

### *3*. Zinc necklaces

**Figure 4:**
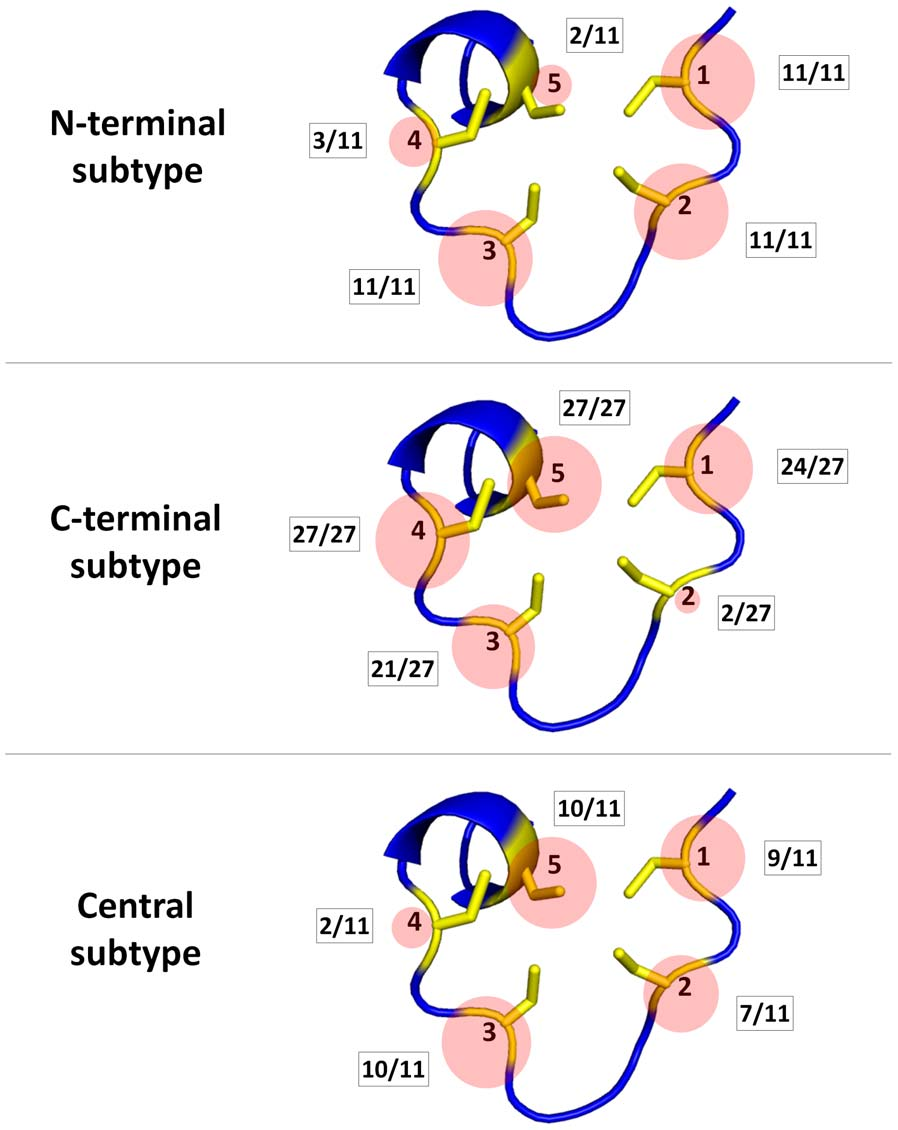
Schematic picture of the positions occupied by zinc ligands in the three subtypes of zinc necklaces. The occupancy of each position is given as the ratio between the number of sites in which a zinc ligand occurs at that position and the total number of sites belonging to the subtype, and shown as a circle sized proportionally to this ratio. Details on the specific ligands occurring in individual sites are given in Table S4.

We introduce the term “zinc necklaces” to indicate the Zn-sites that belong to this cluster, because they can be superimposed onto a structural motif resembling a necklace. The complete zinc necklace motif has five possible positions for zinc ligands, and the zinc ligands in each Zn-site occupy a certain subset of these positions (Figure 4). Depending on the specific positions occupied by the zinc ligands and their distances in sequence, three major subtypes of zinc necklaces can be recognized (Figure 4). The “N-terminal” subtype is characterized by the presence of two closely spaced ligands at positions 1 and 2; in these sites, position 3 is always occupied as well, whereas positions 4 and 5 are usually vacant. Conversely, the “C-terminal” subtype is characterized by the presence of two closely spaced ligands at positions 4 and 5; in these sites, positions 1 and 3 are almost always occupied as well, whereas position 2 is most commonly vacant. The “central” subtype comprises all the other cases, including the Zn-site of wheat EC metallothionein (PDB code 2kak [42]), where all five positions are occupied.

In the classical classification of zinc fingers given by Grishin [17], some of the Zn-sites belonging to this cluster were placed into two different groups, i.e., the “TAZ2 domain-like” group (including, e.g., a zinc necklace of the transcriptional adaptor protein CBP, PDB code 1f81 [43]) and the “short zinc-binding loops” group (including, e.g., a zinc necklace of RNA polymerase II, PDB code 1twf [41]). The “TAZ2 domain-like” sites were defined as having two zinc ligands each from the termini of two α-helices, and the “short zinc-binding loops” sites as having at least three closely spaced zinc ligands from a loop. We suggest that these two groups are better viewed as two variants of the zinc necklace motif, resulting from the presence (“TAZ2 domain-like” case) or the absence (“short zinc-binding loops” case) of α-helices in correspondence of positions 1 and 5. Indeed, the demarcation line between the two groups was somehow blurred even in the original classification, where a Zn-site of DNA polymerase III was classified once among “TAZ2 domain-like” sites (when taken from the PDB structure 1jr3) and once among “short zinc-binding loops” sites (when taken from the PDB structure 1a5t).

The majority of the Zn-superfamilies included in this cluster (32 out of 49) have a structural function, but there are also two with a substrate, two with a regulatory, and one with a catalytic function. Predicting the role of the 12 Zn-superfamilies with unknown functions is therefore less straightforward with respect to the above discussed zinc ribbons and treble clefs, where both structural and functional homogeneity is higher.

### *4*. Zinc rafts

The Zn-sites that belong to this cluster share a common structural scaffold consisting of three adjacent β-strands, which we refer to as a “zinc raft”. Zinc rafts harbour either two or three zinc ligands. Over 70% of them are His residues. The central β-strand always provides at least one ligand, and most often contains two ligands spaced by a single residue (69% of the cases), whereas only one of the two lateral β-strands typically provides a ligand (76% of the cases). The position of zinc with respect to the raft therefore varies depending on which of the two lateral β-strands contains the ligand. In the alignment of Figure 3, the Zn-sites included in the cluster are superimposed so as to have zinc always on the same side of the raft. In this view, the positions occupied by zinc in the individual sites span an arch-shaped region whose central portion corresponds to sites where neither or both of the lateral β-strands provides a ligand.

The zinc raft motif is the most widespread among Zn-superfamilies with a catalytic function (there are 23 in the cluster), but it also occurs in Zn-superfamilies with structural (7 cases), regulatory (3 cases), and substrate functions (1 case). This suggests that this motif, while being best suited for catalytic sites, constitutes a versatile scaffold for zinc sites with diverse roles. The vast majority (i.e., over 80%) of catalytic Zn-sites in the cluster have three protein ligands, whereas all of the structural and regulatory Zn-sites in the cluster have four (or five in the case of glutaminyl cyclase, PDB code 2faw [44]). Out of the 11 Zn-superfamilies with unknown function included in the cluster, therefore, the 7 of them that have three protein ligands are most likely to have a catalytic function.

### *5*. C2H2 zinc fingers

The structural motif shared by the Zn-sites included in this cluster was the first zinc finger to be discovered, and is referred to as a “C2H2” zinc finger from the zinc ligands (i.e., two Cys and two His residues) present in the *Xenopus laevis* transcription factor IIIA where it was originally identified [45]. In its archetypal form, this motif consists of a β-hairpin followed by a α-helix, which provide two zinc ligands each. Although these same structural elements are found in treble clefs (see above), the Zn-sites that belong to this cluster are structurally distinct from treble clefs, in agreement with the classical classification of zinc fingers given by Grishin [17]. Treble clefs and C2H2 zinc fingers are in fact not superimposable on each other, as by superimposing the β-hairpins, the α-helices do not overlap but are translated relative to each other along their axes.

In C2H2 zinc fingers, Cys residues are the most common ligands in the β-hairpin (87% of the cases), whereas His residues are most frequent in the α-helix (74% of the cases). The spacing between the two ligands on the α-helix is typically three residues, but there are variants (30% of the cases) where the spacing is five or six residues, and the C-terminal ligand is found downstream of the helix. In the extreme case of a Zn-site of *Thermus thermophilus* GTP cyclohydrolase I (PDB code 1wur [46]) the C-terminal ligand is absent altogether, and the function of the C2H2 zinc finger is catalytic. All the other Zn-superfamilies included in the cluster have instead a structural function.

### *6*. Loosened zinc ribbons

The Zn-sites included in this cluster have a structure that can be regarded as a variant of the zinc ribbon motif (see above), in which one of the two β-hairpins is replaced by an extended coil. We thus use the term “loosened zinc ribbons” to indicate these Zn-sites. The extended coil typically harbours two zinc ligands spaced by one residue (81% of the cases), and its backbone trace is oriented nearly parallel to the axis of the β-hairpin. Exceptions are the Zn-sites of two viral proteases (PDB codes 2hrv [47] and 3ifu [48]), which are not shown in the alignment of Figure 3 as the extended coil is oriented perpendicular to the axis of the β-hairpin. Similarly to zinc ribbons, zinc ligands in these sites are most commonly Cys (76% of the cases), and the majority of the Zn-superfamilies included in the cluster have a structural function (15 out of 16, the only exception being that of *Bacillus subtilis* Hsp33, which has a regulatory function).

### *7*. Helical anchors

The Zn-sites that belong to this cluster are characterized by the presence of a α-helix providing two zinc ligands, which are most often His (83% of the cases) and are almost always spaced by three residues (93% of the cases). This structural element, which we refer to as a “helical anchor”, is complemented by a variable structural element providing one or, in some cases, two additional zinc ligands. Each additional zinc ligand can be found at one of three possible positions, of which only the most common (occupied in 73% of the cases) is shown in Figure 3. Helical anchors are also present in other Zn-sites, and in C2H2 zinc fingers in particular, where they are complemented by a β-hairpin element (see above). However, C2H2 zinc fingers are not superimposable to the Zn-sites of this cluster.

The majority of the Zn-superfamilies included in this cluster (9 out of 15) have a catalytic function. Indeed, helical anchors represent the most common motif among catalytic Zn-superfamilies after zinc rafts (see above). Similarly to zinc rafts, however, other functions are also possible for helical anchors, as this cluster includes two Zn-superfamilies with a structural and a regulatory function, respectively (as well as four others with unknown functions).

### *8*. Small clusters: shuffled zinc ribbons and Zn2Cys6 zinc fingers

In addition to the large and medium Zn-clusters described above, which altogether comprise about 73% of all Zn-superfamilies, a few additional small Zn-clusters altogether comprising about 5% of all Zn-superfamilies were identified. The largest of these clusters contains eight Zn-sites whose structure consists of two two-stranded β-sheets that approximately lie on the same plane. The zinc ligands are provided by short loops that connect one β-strand of a β-sheet with one β-strand of the other β-sheet. Each loop almost invariably contains two Cys ligands spaced by two residues (94% of the cases). This motif can be described as resulting from a rearrangement of the classical zinc ribbon (see above), in which the pairing of the β-strands is different (i.e., β1–β4 and β2–β3 instead of β1–β2 and β3–β4), and is thus referred to here as a “shuffled zinc ribbon”. We use the same term to indicate another, smaller cluster, which contains two Zn-sites sharing a structural motif similar to that described above, except that one of the two β-sheets is formed by three β-strands, and the loops connecting the two β-sheets harbour only one zinc ligand each (one His and one Cys residue). Two other zinc ligands (two adjacent Cys residues) are instead found on the loop connecting the two C-terminal β-strands of the three-stranded β-sheet. In Grishin's work, both of the above motifs were classified among zinc ribbons (in “DnaJ” and “Btk” subgroups, respectively) [17], however they are neither superimposable on each other, nor on classical zinc ribbons. We therefore suggest to classify them separately as type I (or DnaJ-like) and type II (or Btk-like) shuffled zinc ribbons, respectively. Finally, we identified a small cluster containing seven Zn-sites, whose structure consists of a α-helix (almost invariably harbouring two Cys ligands spaced by two residues) followed by an extended coil resembling that found in loosened zinc ribbons (see above). This motif corresponds to the “Zn2Cys6 zinc finger” group in Grishin's classification, therefore we retain this term to indicate this cluster.

All the Zn-superfamilies included in the small clusters described above have a structural function, except for two (one in type I and one in type II shuffled zinc ribbons) with unknown functions and for that of the *Escherichia coli* Ada protein (PDB code 1adn [49]), whose catalytic site is best described as a type I shuffled zinc ribbon.

### *9*. Unclustered sites: grouping into pseudo-clusters

**Figure 5:**
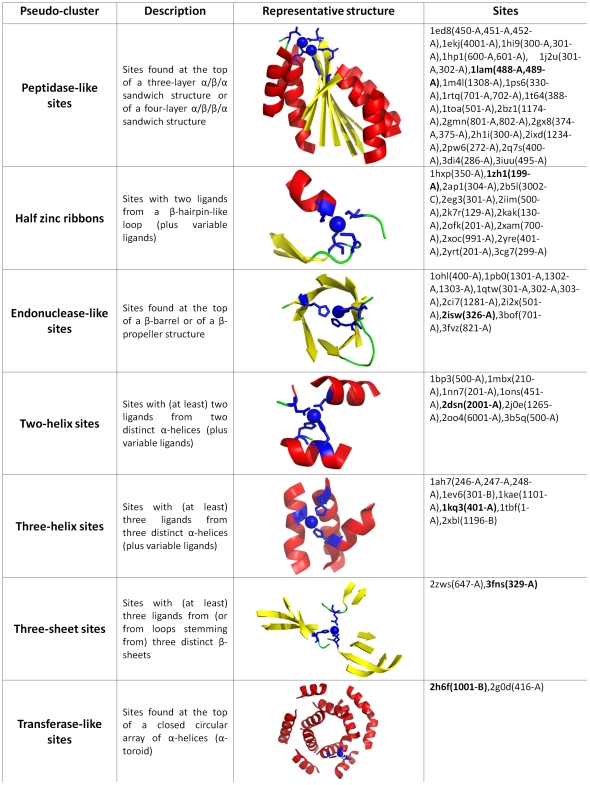
Pseudo-clusters grouping part of the unclustered zinc sites. For each pseudo-cluster, a short description of the criterion used to group the sites, a picture of an example structure (shown as a cartoon representation with zinc atoms as blue spheres and zinc ligands as blue sticks), and a list of the sites included in the pseudo-cluster are given (with the example structure in bold).

A total of 83 representative Zn-sites could not be included in any of the clusters described above. Furthermore, the MFS templates describing the structures of these sites could not be superimposed on one another, meaning that each of them should be considered a unique type of zinc-binding motif. Nonetheless, most of them (i.e., 60 out of 83) could be conveniently grouped under a limited number of categories (which we refer to as “pseudo-clusters”) by using some broader criteria for defining structural similarity, as shown in Figure 5. The largest of these categories (“peptidase-like sites” in Figure 5), for example, includes 17 Zn-sites that are all found at the top of a three-layer sandwich structure with a β-sheet in the central layer and α-helices in the outer layers (α/β/α), as well as 3 Zn-sites found at the top of an analogous, four-layer α/β/β/α structure. Despite being found in protein domains with similar folds, the local structures of these sites differ because the position and arrangement of the zinc ligands, which are mostly provided by loops connecting the β- and the α-layers, are highly variable. The majority of these sites are catalytic, and include those of “classic” zinc enzymes such as carboxypeptidase, aminopeptidase and alkaline phosphatase [15]. The second largest pseudo-cluster (“half zinc ribbons” in Figure 5), instead, contains 14 Zn-sites that are mostly structural, and all have two nearby (in sequence) zinc ligands on a β-hairpin-like loop closely resembling a half-site of zinc ribbons (see above). At variance with zinc ribbons, however, the other half of these sites is highly variable, consisting of two further ligands that can be found in various positions around the β-hairpin-like loop. Altogether, we defined 7 pseudo-clusters which provide at least a coarse-grained classification of the zinc sites that could not be placed in the detailed classification represented by the clusters, ultimately leaving out only 6% of all Zn-superfamilies.

### Concluding remarks

The number of protein structures deposited at the PDB is growing at a rate of about 150 structures per week. On average, 14 of these structures contain zinc but only 11 are true zinc proteins (i.e., they naturally bind zinc for their activity and/or stability). These few statistics exemplify the continuing expansion of our knowledge on the atomic-level interactions between proteins and one of their major inorganic partners (i.e., zinc) but also warn that a significant fraction of these interactions are not relevant to biological function. We thus embarked upon a systematic study of zinc sites in proteins with known structure with the aim of providing an accurate and up-to-date classification that helps researchers to best use the information available in structural databases.

**Figure 6:**
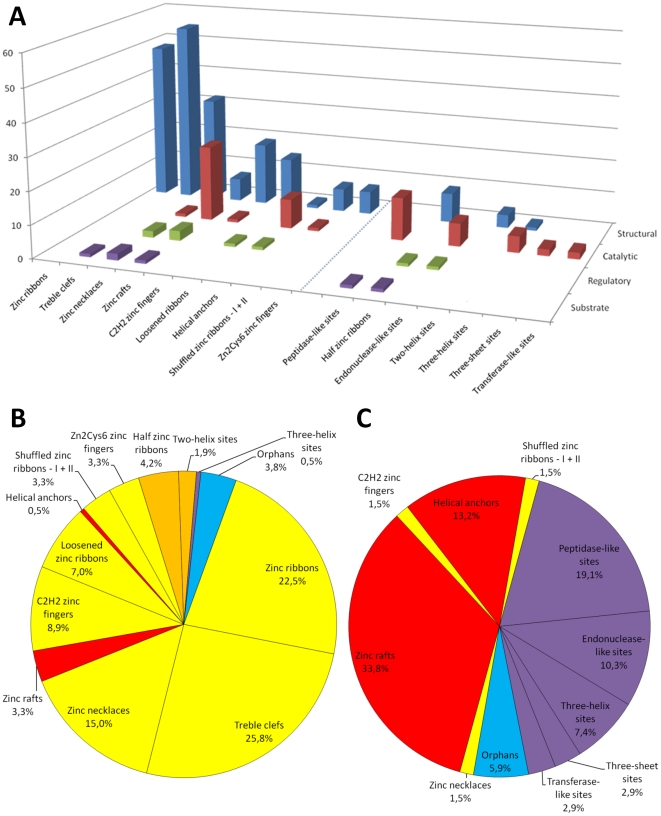
Summary charts showing how zinc sites with specific functions are distributed across clusters and pseudo-clusters. Histogram (A) shows the occurrence of zinc sites with structural, catalytic, regulatory and substrate functions in clusters (left) and pseudo-clusters (right). Pie charts show the shares of structural (B) and catalytic (C) zinc sites occurring in specific clusters and pseudo-clusters, as well as those that remained unassigned (“orphans”). Sectors in pie charts are coloured according to whether clusters and pseudo-clusters contain exclusively or predominantly structural (yellow for clusters and orange for pseudo-clusters) or catalytic sites (red for clusters and purple for pseudo-clusters).

By using a method based on the definition of minimal functional sites as three-dimensional templates encompassing the local structural environment of metals in proteins, we classified 77% of a non-redundant set of zinc sites into 10 clusters (Table 1 and Figure 3), each representing a zinc-binding motif conserved across different protein superfamilies. An additional 16% were classified into 7 broader categories (pseudo-clusters), each representing a set of general structural features (e.g., the secondary structures of zinc ligands) describing the zinc site. A picture of how zinc sites with specific functions are distributed across clusters and pseudo-clusters is given in Figure 6. This Figure shows that structural zinc sites are the majority in eight clusters and in two pseudo-clusters (Figure 6A and 6B), while catalytic zinc sites are predominant in two clusters and in five pseudo-clusters (Figure 6A and 6C). From another point of view, this indicates that, with a few exceptions, only ten types of structural and seven types of catalytic zinc sites appear to occur in proteins. Eight of the ten structural types are indeed well-defined zinc-binding motifs, covering almost 90% of structural zinc sites (Figure 6B). This is the case, instead, only for two of the seven catalytic types (zinc rafts and helical anchors), and catalytic zinc sites are divided almost equally between these two (52%) and the other, less well-defined five types (43%) (Figure 6C). No particular dominant types emerged for regulatory and substrate zinc sites, which appear to resemble more closely catalytic or structural sites depending on the specific case. Clearly, more structural information is needed to understand if there are some structural motifs that can be recognized as characteristic of these sites. Even so, MFSs appear to constitute a helpful conceptual and methodological basis for structure-function studies of zinc proteins, with applications in various areas such as biochemistry, molecular pharmacology and *de novo* protein design.

## Supporting Information

Table S1 List of the non-physiological Zn-sites found in PDB structures and removed from the dataset.
(PDF)

Table S2 Summary of the relevant information on Zn-superfamilies.
(PDF)

Table S3 Lists of the Zn-sites belonging to each Zn-superfamily.
(PDF)

Table S4 Schematic picture of the structures of the representative Zn-sites included in each Zn-cluster.
(PDF)

Table S5 Results of the clustering of representative Zn-sites using different distance threshold values for defining spatially proximal residues in building the MFS templates.
(PDF)
