# Supplementary material for: Minimal Functional Sites Allow a Classification of Zinc Sites in Proteins
Source: PLoS One. 2011 Oct 17;6(10):e26325. doi: 10.1371/journal.pone.0026325 (PMC3197139; doi:10.1371/journal.pone.0026325)
Supplement: Table S1 — List of the non-physiological Zn-sites found in PDB structures and removed from the dataset. (PDF) [file pone.0026325.s001.pdf]

Table S1. List of the non-physiological Zn-sites found in PDB structures and removed from the dataset. Each Zn atom in a Zn-site is identified by six fields (separated by underscores) as (i) PDB code, (ii) chain identifier, (iii) residue name, (iv) residue number, (v) atom name, and (vi) atom number. Multiple Zn atoms in the same Zn-site are separated by commas.

```
1a0b_A_ZN_800_ZN_921
1a0q_H_ZN_212_ZN_3187
1a0q_H_ZN_213_ZN_3188
1a0q_L_ZN_214_ZN_3186
1a1q_A_ZN_901_ZN_535
1a1q_B_ZN_901_ZN_536
1a1q_C_ZN_901_ZN_537
1a2p_A_ZN_112_ZN_2628
1a2p_B_ZN_112_ZN_2629
1a2p_C_ZN_112_ZN_2630
1a6f_A_ZN_201_ZN_952
1a6f_A_ZN_202_ZN_953
1a7w_A_ZN_101_ZN_535
1a8l_A_ZN_440_ZN_1813
1aa0_A_ZN_565_ZN_1039
1agn_C_ZN_401_ZN_11287
1agn_D_ZN_401_ZN_11338
1agn_D_ZN_402_ZN_11339
1agn_D_ZN_403_ZN_11340
1agn_A_ZN_401_ZN_11163
1agn_B_ZN_401_ZN_11222
1agn_B_ZN_402_ZN_11224
1agn_B_ZN_403_ZN_11223
1ak0_A_ZN_274_ZN_2082
1aol_A_ZN_430_ZN_1815,1aol_A_ZN_431_ZN_1816
1aol_A_ZN_432_ZN_1817
1aw5_A_ZN_401_ZN_2530
1aym_1_ZN_6000_ZN_6417
1ayn_1_ZN_6000_ZN_6338
1b0n_A_ZN_1001_ZN_1115
1b0n_A_ZN_1002_ZN_1116
1b0n_A_ZN_1003_ZN_1117
1b0n_A_ZN_1004_ZN_1118
1b0n_A_ZN_1005_ZN_1119
1b1b_A_ZN_301_ZN_1302
1b1b_A_ZN_302_ZN_1303
1b20_A_ZN_377_ZN_2615
1b20_B_ZN_434_ZN_2616
1b20_C_ZN_111_ZN_2617
1b21_C_ZN_341_ZN_2594
1b2x_C_ZN_384_ZN_2608
1b2z_C_ZN_359_ZN_2589
1b4e_A_ZN_401_ZN_2498
1b4e_A_ZN_402_ZN_2496
1b55_B_ZN_171_ZN_2742
1b57_A_ZN_362_ZN_5253
1b57_A_ZN_363_ZN_5254
```

1b57_B_ZN_362_ZN_5269
1b71_A_ZN_194_ZN_1562
1bao_C_ZN_111_ZN_2542
1baw_A_ZN_107_ZN_2420
1baw_B_ZN_107_ZN_2422
1baw_C_ZN_107_ZN_2424
1bc8_B_ZN_2_ZN_1198
1bc8_C_ZN_94_ZN_1199
1bfv_H_ZN_201_ZN_1799
1bfv_L_ZN_200_ZN_1797,1bfv_L_ZN_201_ZN_1798
1bi0_A_ZN_291_ZN_1653
1bi3_A_ZN_291_ZN_2658
1bi3_B_ZN_1291_ZN_2664
1brg_C_ZN_1_ZN_2530
1brh_C_ZN_111_ZN_2531
1brj_C_ZN_111_ZN_2504
1brk_A_ZN_111_ZN_2554
1brk_B_ZN_111_ZN_2555
1brk_C_ZN_111_ZN_2556
1bs4_A_ZN_2001_ZN_4126
1bs4_B_ZN_2002_ZN_4160
1bs4_C_ZN_2003_ZN_4194
1bs5_A_ZN_2001_ZN_4042
1bs5_B_ZN_2001_ZN_4053
1bs5_C_ZN_2001_ZN_4054
1bs8_A_ZN_2001_ZN_4174
1bs8_B_ZN_2001_ZN_4185
1bs8_C_ZN_2001_ZN_4186
1bsk_A_ZN_169_ZN_1331
1bt0_A_ZN_201_ZN_574
1bt0_A_ZN_202_ZN_575
1btg_B_ZN_902_ZN_2512
1btg_C_ZN_900_ZN_2513,1btg_C_ZN_901_ZN_2514
1bxz_A_ZN_354_ZN_10577
1bxz_B_ZN_354_ZN_10585
1bxz_C_ZN_354_ZN_10593
1bxz_D_ZN_354_ZN_10601
1byf_A_ZN_301_ZN_1932
1byf_A_ZN_302_ZN_1933
1byf_A_ZN_303_ZN_1944
1byf_A_ZN_304_ZN_1943
1byf_B_ZN_302_ZN_1962
1byf_B_ZN_303_ZN_1960
1byf_B_ZN_304_ZN_1963
1c1n_A_ZN_409_ZN_3317
1c1r_A_ZN_409_ZN_3321
1c1u_H_ZN_254_ZN_4814
1c1v_H_ZN_254_ZN_4809
1c1v_H_ZN_255_ZN_4810
1c1w_H_ZN_254_ZN_4809
1c1w_H_ZN_255_ZN_4810
1c1w_L_ZN_249_ZN_4808
1c2d_A_ZN_409_ZN_3304
1c2d_A_ZN_410_ZN_3305
1c2e_A_ZN_409_ZN_3310
1c2f_A_ZN_409_ZN_3298
1c2f_A_ZN_410_ZN_3299,1c2f_A_ZN_411_ZN_3300
1c2g_A_ZN_409_ZN_3313

1c2g_A_ZN_410_ZN_3314,1c2g_A_ZN_411_ZN_3315
1c2h_A_ZN_409_ZN_3288
1c2h_A_ZN_410_ZN_3289,1c2h_A_ZN_411_ZN_3290
1c2i_A_ZN_409_ZN_3319
1c2i_A_ZN_410_ZN_3320,1c2i_A_ZN_411_ZN_3321
1c2j_A_ZN_409_ZN_3326
1c2k_A_ZN_258_ZN_3335
1c2k_A_ZN_261_ZN_3336
1c2l_A_ZN_409_ZN_3324
1c2l_A_ZN_410_ZN_3325
1c2m_A_ZN_409_ZN_3300
1c8m_1_ZN_6000_ZN_6315
1c8y_A_ZN_271_ZN_2015
1cak_A_ZN_362_ZN_2078
1caz_A_ZN_362_ZN_2078
1ces_A_ZN_238_ZN_3412
1ces_B_ZN_238_ZN_3413
1cfv_L_ZN_200_ZN_1803,1cfv_L_ZN_202_ZN_1805
1cfv_L_ZN_201_ZN_1804
1cg2_A_ZN_502_ZN_11129
1cg2_A_ZN_503_ZN_11130
1cg2_B_ZN_502_ZN_11133
1cg2_C_ZN_502_ZN_11136
1cg2_D_ZN_502_ZN_11139
1ci3_M_ZN_256_ZN_1888
1ci3_M_ZN_257_ZN_1889
1cjv_A_ZN_581_ZN_5637
1cjv_C_ZN_405_ZN_5711
1cko_A_ZN_888_ZN_2563
1clc_A_ZN_653_ZN_4218
1cnq_A_ZN_341_ZN_2571,1cnq_A_ZN_342_ZN_2572,1cnq_A_ZN_343_ZN_2573
1cpr_A_ZN_130_ZN_921,1cpr_A_ZN_131_ZN_922
1cpr_A_ZN_132_ZN_923
1cpr_A_ZN_133_ZN_924
1cpr_A_ZN_134_ZN_925
1cpr_A_ZN_135_ZN_926
1cvr_A_ZN_673_ZN_3389
1cvr_A_ZN_731_ZN_3387
1cy5_A_ZN_101_ZN_747,1cy5_A_ZN_101_ZN_748
1cy5_A_ZN_102_ZN_749
1cy5_A_ZN_103_ZN_750
1cy5_A_ZN_104_ZN_751
1cy5_A_ZN_105_ZN_752
1cyy_A_ZN_1001_ZN_3953
1czf_A_ZN_501_ZN_4935
1czf_A_ZN_502_ZN_4936
1czf_A_ZN_503_ZN_4937
1czf_B_ZN_504_ZN_4952
1czf_B_ZN_505_ZN_4953
1czf_B_ZN_506_ZN_4954
1d0g_A_ZN_300_ZN_6297
1d1s_B_ZN_405_ZN_11235
1d1s_B_ZN_406_ZN_11236
1d1s_C_ZN_407_ZN_11311
1d1s_D_ZN_409_ZN_11366
1d1s_D_ZN_410_ZN_11367
1d1s_D_ZN_411_ZN_11368
1d1s_A_ZN_401_ZN_11163

1d1s_A_ZN_402_ZN_11164
1d1s_A_ZN_403_ZN_11165
1d1s_A_ZN_408_ZN_11166
1d1s_B_ZN_404_ZN_11234
1d1t_B_ZN_405_ZN_11235
1d1t_B_ZN_406_ZN_11236
1d1t_C_ZN_407_ZN_11311
1d1t_D_ZN_409_ZN_11366
1d1t_D_ZN_410_ZN_11367
1d1t_D_ZN_411_ZN_11368
1d1t_A_ZN_401_ZN_11163
1d1t_A_ZN_402_ZN_11164
1d1t_A_ZN_403_ZN_11165
1d1t_A_ZN_408_ZN_11166
1d1t_B_ZN_404_ZN_11234
1d8y_A_ZN_3_ZN_4809
1d9d_A_ZN_1_ZN_4862
1d9d_A_ZN_320_ZN_4864
1d9d_A_ZN_322_ZN_4866
1d9d_A_ZN_3_ZN_4863
1d9f_A_ZN_1_ZN_4878
1d9x_A_ZN_701_ZN_4634
1d9x_A_ZN_702_ZN_4635
1d9z_A_ZN_701_ZN_4635
1d9z_A_ZN_702_ZN_4636
1def_A_ZN_148_ZN_2353
1dff_A_ZN_165_ZN_1317
1dg6_A_ZN_300_ZN_1251
1dk4_A_ZN_290_ZN_4039,1dk4_A_ZN_291_ZN_4040,1dk4_A_ZN_292_ZN_4041
1dk4_B_ZN_590_ZN_4047,1dk4_B_ZN_591_ZN_4048,1dk4_B_ZN_592_ZN_4049
1dkh_A_ZN_201_ZN_1253
1dkh_A_ZN_202_ZN_1254,1dkh_A_ZN_203_ZN_1255
1dq3_A_ZN_901_ZN_3757
1du3_E_ZN_1_ZN_11957
1du3_K_ZN_2_ZN_11958
1dv6_H_ZN_1010_ZN_13480
1dv6_T_ZN_2010_ZN_13915
1dvb_A_ZN_194_ZN_1576
1dvf_B_ZN_1160_ZN_3500
1dvf_C_ZN_1159_ZN_3501
1dvf_D_ZN_1158_ZN_3499
1dwa_W_ZN_1_ZN_4363
1dwf_M_ZN_1524_ZN_4363
1dwg_W_ZN_1_ZN_4360
1dwh_W_ZN_1_ZN_4363
1dwi_W_ZN_1_ZN_4363
1dwj_W_ZN_1_ZN_4363
1dx8_A_ZN_71_ZN_1065
1dy0_A_ZN_402_ZN_1403
1dy1_A_ZN_402_ZN_1423
1e08_D_ZN_12_ZN_5015
1e4m_M_ZN_1502_ZN_4334
1e67_A_ZN_129_ZN_3901
1e67_B_ZN_129_ZN_3906
1e67_C_ZN_129_ZN_3907
1e67_D_ZN_129_ZN_3908
1e6q_M_ZN_1520_ZN_4402
1e6s_M_ZN_1502_ZN_4292

1e6x_M_ZN_1502_ZN_4335
1e70_M_ZN_1502_ZN_4338
1e71_M_ZN_1503_ZN_4352
1e72_M_ZN_1502_ZN_4348
1e73_M_ZN_1515_ZN_4422
1e87_A_ZN_1001_ZN_1030
1ear_A_ZN_1143_ZN_1220
1eb0_A_ZN_1144_ZN_1189
1ebo_A_ZN_150_ZN_5335
1ebo_D_ZN_150_ZN_5333
1ebo_F_ZN_150_ZN_5334
1ec5_A_ZN_50_ZN_1252
1ec5_B_ZN_50_ZN_1253,1ec5_C_ZN_50_ZN_1254
1efq_A_ZN_200_ZN_862
1ekb_B_ZN_310_ZN_1915
1ekb_B_ZN_311_ZN_1916
1ekm_A_ZN_701_ZN_15595
1ekm_B_ZN_701_ZN_15596
1ekm_C_ZN_701_ZN_15597
1et5_A_ZN_504_ZN_2564
1et5_A_ZN_505_ZN_2565
1et8_A_ZN_504_ZN_2586
1et8_A_ZN_505_ZN_2587
1ete_A_ZN_135_ZN_4276
1ete_A_ZN_136_ZN_4277
1ete_A_ZN_137_ZN_4278
1ete_B_ZN_1135_ZN_4279
1ete_B_ZN_1136_ZN_4280
1ete_B_ZN_1137_ZN_4281
1ete_C_ZN_2135_ZN_4282
1ete_C_ZN_2136_ZN_4283
1ete_D_ZN_3135_ZN_4284
1euc_B_ZN_400_ZN_5257
1exi_A_ZN_501_ZN_2542
1exj_A_ZN_501_ZN_2529
1eyk_A_ZN_339_ZN_5011
1eyk_B_ZN_344_ZN_5056
1f1m_A_ZN_801_ZN_4901
1f1m_B_ZN_802_ZN_4902
1f1m_B_ZN_803_ZN_4903
1f1m_C_ZN_804_ZN_4904
1f1m_D_ZN_805_ZN_4905
1f1m_D_ZN_806_ZN_4906
1f30_A_ZN_201_ZN_14845
1f30_J_ZN_201_ZN_14910
1f30_K_ZN_201_ZN_14927
1f30_L_ZN_201_ZN_14936
1f30_B_ZN_201_ZN_14862
1f30_C_ZN_201_ZN_14863
1f30_D_ZN_201_ZN_14872
1f30_E_ZN_201_ZN_14881
1f30_F_ZN_201_ZN_14890
1f30_G_ZN_201_ZN_14899
1f30_H_ZN_201_ZN_14900
1f30_I_ZN_201_ZN_14901
1f35_A_ZN_301_ZN_2641,1f35_A_ZN_302_ZN_2651
1f35_A_ZN_304_ZN_2642
1f35_A_ZN_305_ZN_2643

1f35_A_ZN_306_ZN_2644
1f35_A_ZN_307_ZN_2645
1f35_B_ZN_303_ZN_2655
1f35_B_ZN_308_ZN_2652,1f35_B_ZN_309_ZN_2653
1f35_B_ZN_310_ZN_2654
1f3z_A_ZN_300_ZN_1109
1f4t_A_ZN_369_ZN_6039
1f5f_A_ZN_252_ZN_1370
1f88_A_ZN_907_ZN_5129
1f88_A_ZN_910_ZN_5130
1f88_B_ZN_908_ZN_5221
1f88_B_ZN_909_ZN_5222
1fbe_A_ZN_341_ZN_5862,1fbe_A_ZN_342_ZN_5863
1fbe_B_ZN_341_ZN_5864,1fbe_B_ZN_342_ZN_5865
1fd9_A_ZN_401_ZN_1549,1fd9_A_ZN_402_ZN_1550
1ffy_A_ZN_1992_ZN_9025
1fio_A_ZN_501_ZN_1555
1fio_A_ZN_502_ZN_1556
1fj6_A_ZN_403_ZN_2523,1fj6_A_ZN_404_ZN_2524,1fj6_A_ZN_405_ZN_2525
1fj9_A_ZN_403_ZN_4901,1fj9_A_ZN_404_ZN_4902
1fj9_B_ZN_413_ZN_4947,1fj9_B_ZN_414_ZN_4948
1fr9_A_ZN_196_ZN_1449
1frp_A_ZN_337_ZN_6005
1frp_B_ZN_337_ZN_6051
1frw_A_ZN_196_ZN_1461
1fss_A_ZN_901_ZN_4706
1fss_A_ZN_902_ZN_4707
1ftj_A_ZN_269_ZN_5892
1ftj_B_ZN_270_ZN_5903
1ftj_B_ZN_272_ZN_5904
1ftj_C_ZN_271_ZN_5915
1ftj_C_ZN_273_ZN_5916
1ftm_A_ZN_422_ZN_5870
1ftm_B_ZN_423_ZN_5884
1ftm_B_ZN_425_ZN_5885
1ftm_C_ZN_424_ZN_5899
1ftm_C_ZN_426_ZN_5900
1fuk_A_ZN_900_ZN_1266
1fwz_A_ZN_301_ZN_1588
1fxu_A_ZN_302_ZN_2170
1g3y_A_ZN_301_ZN_1630
1g43_A_ZN_201_ZN_1215,1g43_A_ZN_202_ZN_1216
1g6d_A_ZN_11_ZN_208
1g6d_A_ZN_12_ZN_209
1g6d_A_ZN_13_ZN_210
1g9a_A_ZN_1294_ZN_10589
1g9b_A_ZN_1294_ZN_10589
1g9c_A_ZN_1294_ZN_10589
1g9d_A_ZN_1294_ZN_10589
1gen_A_ZN_301_ZN_1959
1ghq_A_ZN_802_ZN_4438
1ghq_B_ZN_801_ZN_4454
1ghx_L_ZN_859_ZN_4833
1ghy_H_ZN_247_ZN_4836
1ghy_H_ZN_248_ZN_4837
1ghy_L_ZN_580_ZN_4835
1gi4_A_ZN_409_ZN_3369
1gl4_A_ZN_1632_ZN_2806

1glc_F_ZN_169_ZN_4912
1gle_F_ZN_169_ZN_4912
1gle_G_ZN_502_ZN_4913
1gr0_A_ZN_1200_ZN_2552
1gs7_A_ZN_1338_ZN_2579
1gs7_A_ZN_1339_ZN_2580
1gs8_A_ZN_1339_ZN_2590
1gs8_A_ZN_1340_ZN_2591
1gs8_A_ZN_1341_ZN_2592
1gud_A_ZN_1289_ZN_4268
1gud_A_ZN_1290_ZN_4269
1gud_A_ZN_1291_ZN_4270
1gud_A_ZN_1292_ZN_4271
1gud_B_ZN_1289_ZN_4272
1gud_B_ZN_1290_ZN_4273
1gud_B_ZN_1291_ZN_4274
1gud_B_ZN_1292_ZN_4275
1gvy_A_ZN_1423_ZN_3041
1gvy_A_ZN_1425_ZN_3043
1gw1_A_ZN_1422_ZN_3031
1gw1_A_ZN_1424_ZN_3033
1gzm_A_ZN_1349_ZN_5476
1gzm_B_ZN_1349_ZN_5743
1h1o_A_ZN_1187_ZN_2712
1h1o_A_ZN_1188_ZN_2713
1h1o_A_ZN_1189_ZN_2714
1h1o_B_ZN_1388_ZN_2812
1h1o_B_ZN_1389_ZN_2813
1h1o_B_ZN_1390_ZN_2814
1h2b_A_ZN_1363_ZN_5389
1h2m_A_ZN_1350_ZN_2862
1h7v_A_ZN_61_ZN_922
1hbm_D_ZN_1556_ZN_19541
1hbn_A_ZN_1557_ZN_19413
1hbo_A_ZN_1556_ZN_19483
1hbu_A_ZN_1557_ZN_19388
1hfe_S_ZN_500_ZN_7780
1hk8_A_ZN_1589_ZN_4475
1hkk_A_ZN_1392_ZN_2959
1hkk_A_ZN_1393_ZN_2960
1hkk_A_ZN_1394_ZN_2961
1hml_A_ZN_125_ZN_986
1hp7_A_ZN_401_ZN_2987
1hp7_A_ZN_402_ZN_2988
1hp7_A_ZN_403_ZN_2989
1hp7_A_ZN_404_ZN_2990
1hp7_A_ZN_405_ZN_2991
1hw1_A_ZN_506_ZN_3614
1hwt_D_ZN_138_ZN_4008
1hz5_A_ZN_101_ZN_1135
1hz5_B_ZN_110_ZN_1144
1hz5_A_ZN_102_ZN_1136
1hz5_A_ZN_103_ZN_1137
1hz5_A_ZN_104_ZN_1138
1hz5_A_ZN_106_ZN_1139
1hz5_A_ZN_108_ZN_1140
1hz5_B_ZN_105_ZN_1141
1hz5_B_ZN_107_ZN_1142

1hz5_B_ZN_109_ZN_1143
1hzx_A_ZN_957_ZN_5261
1hzx_A_ZN_959_ZN_5262
1hzx_A_ZN_962_ZN_5263
1hzx_A_ZN_964_ZN_5264
1hzx_B_ZN_956_ZN_5482
1hzx_B_ZN_958_ZN_5483
1hzx_B_ZN_963_ZN_5484
1i27_A_ZN_999_ZN_618
1i54_A_ZNH_1105_ZN_1646
1i54_B_ZNH_1107_ZN_1732
1i55_A_ZNH_1105_ZN_1646
1i55_B_ZNH_1107_ZN_1732
1i6h_A_ZN_1734_ZN_28439
1i6v_D_ZN_1601_ZN_21238
1i7w_A_ZN_1_ZN_8686
1i7w_C_ZN_2_ZN_8691
1i94_D_ZN_212_ZN_44491
1i95_D_ZN_212_ZN_35042
1i95_N_ZN_76_ZN_35041
1i96_D_ZN_212_ZN_35129
1i96_N_ZN_76_ZN_35128
1i97_D_ZN_212_ZN_35042
1i97_N_ZN_76_ZN_35041
1i9r_H_ZN_498_ZN_13177
1i9r_K_ZN_499_ZN_13178
1i9r_X_ZN_500_ZN_13179
1ia6_A_ZN_1264_ZN_3380
1ia7_A_ZN_1333_ZN_3433
1iau_A_ZN_501_ZN_1920
1iau_A_ZN_502_ZN_1921
1iau_A_ZN_503_ZN_1922
1iau_A_ZN_504_ZN_1923
1iau_A_ZN_505_ZN_1924
1ibq_A_ZN_1451_ZN_4859
1ibq_A_ZN_1453_ZN_4860
1ibq_A_ZN_1455_ZN_4861
1ibq_A_ZN_1457_ZN_4862
1ibq_B_ZN_1452_ZN_4885
1ibq_B_ZN_1454_ZN_4886
1ibq_B_ZN_1456_ZN_4887
1ibq_B_ZN_1458_ZN_4888
1ij0_A_ZN_201_ZN_781
1ij0_B_ZN_202_ZN_782
1ij0_C_ZN_203_ZN_783
1ij1_A_ZN_201_ZN_784
1ij1_B_ZN_202_ZN_785
1ij1_C_ZN_203_ZN_786
1ijl_A_ZN_201_ZN_1926
1io0_A_ZN_1001_ZN_1299
1iqb_A_ZN_90_ZN_1295
1iqb_B_ZN_91_ZN_1296
1irn_A_ZN_55_ZN_764
1irx_B_ZN_602_ZN_8508
1iuj_A_ZN_2007_ZN_1681
1iuj_B_ZN_2001_ZN_1682,1iuj_B_ZN_2002_ZN_1683,1iuj_B_ZN_2003_ZN_1684,1iuj
_B_ZN_2004_ZN_1685,1iuj_B_ZN_2005_ZN_1686
1iuj_B_ZN_2006_ZN_1687

1iwl_A_ZN_191_ZN_1411
1iwl_A_ZN_192_ZN_1412
1iwl_A_ZN_193_ZN_1413
1ix1_A_ZN_201_ZN_2723
1ix1_B_ZN_201_ZN_2764
1j2x_A_ZN_1000_ZN_720
1j30_A_ZN_405_ZN_2197
1j30_B_ZN_402_ZN_2200
1j9y_A_ZN_1001_ZN_2726
1j9y_A_ZN_1002_ZN_2727
1j9y_A_ZN_1003_ZN_2728
1j9y_A_ZN_1004_ZN_2729
1jaz_A_ZN_401_ZN_4603
1jaz_B_ZN_402_ZN_4604
1jaz_B_ZN_403_ZN_4605
1jcc_A_ZN_81_ZN_1098
1jcc_B_ZN_82_ZN_1099
1jcc_C_ZN_83_ZN_1100
1jf1_A_ZN_9001_ZN_3148
1jff_A_ZN_900_ZN_6581
1jje_A_ZN_261_ZN_3449
1jje_B_ZN_261_ZN_3481
1jjt_A_ZN_261_ZN_3449
1jjt_B_ZN_261_ZN_3484
1jk0_A_ZN_501_ZN_4945
1jk9_B_ZN_303_ZN_5965
1jk9_D_ZN_304_ZN_5972
1jke_A_ZN_906_ZN_4464
1jke_D_ZN_903_ZN_4473
1jke_D_ZN_904_ZN_4474
1jke_D_ZN_907_ZN_4475
1jke_A_ZN_908_ZN_4465
1jke_A_ZN_909_ZN_4466
1jke_B_ZN_912_ZN_4467
1jke_C_ZN_905_ZN_4468
1jke_C_ZN_910_ZN_4469
1jke_C_ZN_911_ZN_4470
1jke_D_ZN_901_ZN_4471
1jke_D_ZN_902_ZN_4472
1jml_A_ZN_101_ZN_561
1jml_A_ZN_102_ZN_562
1jml_A_ZN_103_ZN_563
1job_A_ZN_301_ZN_1321
1job_A_ZN_302_ZN_1322
1job_A_ZN_303_ZN_1323
1job_A_ZN_304_ZN_1324,1job_A_ZN_305_ZN_1325
1job_A_ZN_306_ZN_1326
1jod_A_ZN_301_ZN_2613,1jod_A_ZN_313_ZN_2619
1jod_A_ZN_318_ZN_2624,1jod_A_ZN_319_ZN_2625
1jod_A_ZN_321_ZN_2626
1jod_B_ZN_306_ZN_2642
1jod_B_ZN_307_ZN_2643
1jod_B_ZN_308_ZN_2644
1jod_B_ZN_309_ZN_2645
1jod_B_ZN_310_ZN_2646
1jod_B_ZN_312_ZN_2647
1jod_B_ZN_320_ZN_2648
1jod_A_ZN_302_ZN_2614

1jod_A_ZN_303_ZN_2615,1jod_B_ZN_322_ZN_2649
1jod_A_ZN_304_ZN_2616
1jod_A_ZN_305_ZN_2617
1jod_A_ZN_311_ZN_2618
1jod_A_ZN_314_ZN_2620,1jod_A_ZN_315_ZN_2621
1jod_A_ZN_316_ZN_2622
1jod_A_ZN_317_ZN_2623
1jpu_A_ZN_372_ZN_2788
1jq5_A_ZN_372_ZN_2830
1jqa_A_ZN_372_ZN_2735
1jr9_A_ZN_205_ZN_1585
1jv0_A_ZN_262_ZN_3999
1jv0_A_ZN_264_ZN_4001
1jv0_B_ZN_262_ZN_4007
1jv0_B_ZN_264_ZN_4009
1jyb_A_ZN_601_ZN_1518
1k24_A_ZN_401_ZN_1954
1k24_A_ZN_402_ZN_1955
1k24_A_ZN_403_ZN_1956
1k2f_A_ZN_603_ZN_3000
1k2f_A_ZN_604_ZN_3001
1k2y_X_ZN_500_ZN_3460
1k35_A_ZN_500_ZN_3382
1k4p_A_ZN_1003_ZN_1643
1k4p_A_ZN_1004_ZN_1644,1k4p_A_ZN_1005_ZN_1645,1k4p_A_ZN_1006_ZN_1646,1k4p
_A_ZN_1007_ZN_1647
1k51_A_ZN_1001_ZN_554
1k51_A_ZN_1002_ZN_555
1k51_A_ZN_1003_ZN_556
1k52_A_ZN_1001_ZN_1125
1k52_A_ZN_1002_ZN_1126
1k52_A_ZN_1003_ZN_1127
1k52_A_ZN_1006_ZN_1128
1k52_A_ZN_1008_ZN_1129
1k52_B_ZN_1004_ZN_1130
1k52_B_ZN_1005_ZN_1131
1k52_B_ZN_1007_ZN_1132
1k53_A_ZN_1001_ZN_1141
1k53_A_ZN_1002_ZN_1142
1k53_A_ZN_1003_ZN_1143
1k53_A_ZN_1004_ZN_1144
1k53_A_ZN_1005_ZN_1145
1k53_A_ZN_1007_ZN_1146
1k53_B_ZN_1006_ZN_1147
1k53_B_ZN_1008_ZN_1148
1k9z_A_ZN_401_ZN_2727,1k9z_A_ZN_402_ZN_2728,1k9z_A_ZN_403_ZN_2729
1k9z_A_ZN_404_ZN_2730
1k9z_A_ZN_405_ZN_2731
1k9z_A_ZN_406_ZN_2732
1k9z_A_ZN_407_ZN_2733
1k9z_A_ZN_408_ZN_2734
1k9z_A_ZN_409_ZN_2735
1kea_A_ZN_1001_ZN_1784
1kfi_A_ZN_700_ZN_8983
1kfi_B_ZN_700_ZN_8994
1kfs_A_ZN_1_ZN_4820
1kfs_A_ZN_320_ZN_4822
1kfs_A_ZN_3_ZN_4821

1kl9_A_ZN_501_ZN_1304
1kl9_A_ZN_502_ZN_1305
1kl9_A_ZN_503_ZN_1306
1kl9_A_ZN_504_ZN_1307
1kn2_L_ZN_553_ZN_3392
1kn2_L_ZN_554_ZN_3393,1kn2_L_ZN_559_ZN_3398
1kn2_L_ZN_555_ZN_3394,1kn2_L_ZN_556_ZN_3395,1kn2_L_ZN_557_ZN_3396,1kn2_L_ZN_558_ZN_3397
1kn4_L_ZN_553_ZN_3392
1kn4_L_ZN_554_ZN_3393,1kn4_L_ZN_559_ZN_3398
1kn4_L_ZN_555_ZN_3394,1kn4_L_ZN_556_ZN_3395,1kn4_L_ZN_557_ZN_3396,1kn4_L_ZN_558_ZN_3397
1kno_A_ZN_215_ZN_9913
1ko2_A_ZN_301_ZN_1739
1ko3_A_ZN_3_ZN_1737
1kq0_A_ZN_479_ZN_2784,1kq0_A_ZN_480_ZN_2785
1kq9_A_ZN_479_ZN_2724,1kq9_A_ZN_480_ZN_2725
1krp_A_ZN_1_ZN_4818,1krp_B_ZN_2_ZN_4817
1krp_A_ZN_320_ZN_4819
1krp_A_ZN_3_ZN_4820
1ksp_A_ZN_320_ZN_4817
1ksp_A_ZN_3_ZN_4818
1kys_A_ZN_301_ZN_1799
1kys_A_ZN_304_ZN_1796
1kys_A_ZN_306_ZN_1797
1kys_A_ZN_308_ZN_1798
1l0h_A_ZN_80_ZN_593
1l0h_A_ZN_81_ZN_594
1l0i_A_ZN_1002_ZN_618
1l0i_A_ZN_1004_ZN_624
1l0i_A_ZN_1005_ZN_625,1l0i_A_ZN_1006_ZN_626,1l0i_A_ZN_1007_ZN_627,1l0i_A_ZN_1008_ZN_628
1l0i_A_ZN_1009_ZN_629
1l0y_A_ZN_701_ZN_7098
1l0y_B_ZN_706_ZN_7106
1l0y_C_ZN_703_ZN_7107
1l0y_D_ZN_705_ZN_7109
1l3f_E_ZN_330_ZN_2435
1l7o_A_ZN_302_ZN_3202
1l7o_B_ZN_301_ZN_3207
1l7o_B_ZN_303_ZN_3208
1l9h_A_ZN_2011_ZN_5267
1l9h_A_ZN_957_ZN_5264
1l9h_A_ZN_959_ZN_5265
1l9h_A_ZN_962_ZN_5266
1l9h_B_ZN_956_ZN_5505
1l9h_B_ZN_958_ZN_5506
1l9h_B_ZN_963_ZN_5507
1l9u_D_ZN_2002_ZN_18764
1l9u_D_ZN_2003_ZN_18765
1l9u_M_ZN_3002_ZN_18767
1l9u_M_ZN_3003_ZN_18768
1l9z_D_ZN_2002_ZN_10085
1l9z_D_ZN_2003_ZN_10086
1lam_A_ZN_490_ZN_3709
1lan_A_ZN_490_ZN_3716
1lbc_A_ZN_321_ZN_5868
1lbc_B_ZN_322_ZN_5903

1lbc_B_ZN_324_ZN_5904
1lbc_C_ZN_323_ZN_5939
1lbc_C_ZN_325_ZN_5940
1lcp_A_ZN_490_ZN_7509
1lcp_B_ZN_490_ZN_7546
1ld3_A_ZN_500_ZN_2487
1lhr_A_ZN_403_ZN_4806
1lhr_B_ZN_404_ZN_4839
1liq_A_ZN_28_ZN_424
1lmh_A_ZN_185_ZN_1453
1lqw_A_ZN_202_ZN_2878
1lqw_B_ZN_201_ZN_2879
1lr0_A_ZN_130_ZN_970
1lr0_A_ZN_131_ZN_971
1lru_A_ZN_601_ZN_3883
1lru_B_ZN_602_ZN_3911
1lru_C_ZN_603_ZN_3949
1lry_A_ZN_168_ZN_1334
1lu0_A_ZN_201_ZN_479
1m2a_A_ZN_301_ZN_1624
1m2a_A_ZN_302_ZN_1625
1m2a_A_ZN_303_ZN_1626
1m2a_A_ZN_304_ZN_1627
1m2x_A_ZN_900_ZN_7229
1m2x_B_ZN_900_ZN_7252
1m2x_C_ZN_900_ZN_7275
1m2x_D_ZN_900_ZN_7298
1m55_A_ZN_205_ZN_3164
1m55_B_ZN_203_ZN_3167
1m55_B_ZN_204_ZN_3168
1m55_B_ZN_206_ZN_3169
1m5b_A_ZN_2103_ZN_6136
1m5b_A_ZN_2104_ZN_6137
1m5b_B_ZN_2105_ZN_6156
1m5b_B_ZN_2106_ZN_6157
1m5b_C_ZN_2107_ZN_6176
1m5e_A_ZN_1704_ZN_6150
1m5e_A_ZN_1705_ZN_6151,1m5e_A_ZN_1710_ZN_6152,1m5e_B_ZN_1711_ZN_6175
1m5e_B_ZN_1706_ZN_6172
1m5e_B_ZN_1707_ZN_6173
1m5e_B_ZN_1709_ZN_6174
1m5e_C_ZN_1708_ZN_6191
1m5f_A_ZN_1204_ZN_6097
1m5f_B_ZN_1205_ZN_6117,1m5f_B_ZN_1209_ZN_6120
1m5f_B_ZN_1206_ZN_6118
1m5f_B_ZN_1207_ZN_6119
1m5f_C_ZN_1208_ZN_6136
1m60_A_HES_105_ZN_1672
1mey_G_ZN_91_ZN_2647
1mft_A_ZN_60_ZN_885,1mft_B_ZN_60_ZN_886
1mh2_A_ZN_201_ZN_1819
1mkm_A_ZN_402_ZN_3971
1mkm_B_ZN_401_ZN_3975
1ml2_A_ZEM_296_ZN_2386
1mm7_A_ZN_901_ZN_5855
1mm7_B_ZN_902_ZN_5869
1mm7_B_ZN_904_ZN_5870
1mm7_C_ZN_903_ZN_5884

1mm7_C_ZN_905_ZN_5885
1mqj_A_ZN_401_ZN_1960
1mrc_L_ZN_600_ZN_3138
1mrd_L_ZN_600_ZN_3172
1mre_L_ZN_600_ZN_3210
1mrf_L_ZN_600_ZN_3194
1mro_A_ZN_801_ZN_19304
1ms7_A_ZN_3002_ZN_6055
1ms7_A_ZN_3003_ZN_6056
1ms7_B_ZN_3001_ZN_6069
1ms7_B_ZN_3004_ZN_6070
1ms7_B_ZN_3005_ZN_6071,1ms7_B_ZN_3006_ZN_6072
1mwo_A_ZN_436_ZN_3571
1mwo_A_ZN_437_ZN_3572
1mwo_A_ZN_438_ZN_3573
1mwo_A_ZN_439_ZN_3574
1mwo_A_ZN_440_ZN_3575
1mwo_A_ZN_441_ZN_3576
1mwq_A_ZN_301_ZN_1708
1mwq_A_ZN_303_ZN_1709
1mwq_B_ZN_302_ZN_1738
1mwq_B_ZN_304_ZN_1739
1mxd_A_ZN_725_ZN_3709
1mxd_A_ZN_726_ZN_3710
1mxd_A_ZN_727_ZN_3711
1mxd_A_ZN_728_ZN_3712
1mxd_A_ZN_729_ZN_3713
1mxd_A_ZN_730_ZN_3714
1mxg_A_ZN_437_ZN_3732
1mxu_A_ZN_701_ZN_5885
1mxu_B_ZN_702_ZN_5901
1mxu_B_ZN_704_ZN_5902
1mxu_C_ZN_703_ZN_5918
1mxu_C_ZN_705_ZN_5919
1mxv_A_ZN_701_ZN_5885
1mxv_B_ZN_702_ZN_5886
1mxv_B_ZN_704_ZN_5887
1mxv_C_ZN_703_ZN_5888
1mxv_C_ZN_705_ZN_5889
1mxw_A_ZN_701_ZN_5885
1mxw_B_ZN_702_ZN_5886
1mxw_B_ZN_704_ZN_5887
1mxw_C_ZN_703_ZN_5888
1mxw_C_ZN_705_ZN_5889
1mxx_A_ZN_701_ZN_5885
1mxx_B_ZN_702_ZN_5886
1mxx_B_ZN_704_ZN_5887
1mxx_C_ZN_703_ZN_5888
1mxx_C_ZN_705_ZN_5889
1mxy_A_ZN_701_ZN_5886
1mxy_B_ZN_702_ZN_5887
1mxy_B_ZN_704_ZN_5888
1mxy_C_ZN_703_ZN_5889
1mxy_C_ZN_705_ZN_5890
1mxz_A_ZN_701_ZN_6053
1mxz_B_ZN_702_ZN_6054
1mxz_B_ZN_704_ZN_6055
1mxz_C_ZN_703_ZN_6056

1mxz_C_ZN_705_ZN_6057
1my0_A_ZN_701_ZN_6053
1my0_B_ZN_702_ZN_6054
1my0_B_ZN_704_ZN_6055
1my0_C_ZN_703_ZN_6056
1my0_C_ZN_705_ZN_6057
1my1_A_ZN_701_ZN_6053
1my1_B_ZN_702_ZN_6054
1my1_B_ZN_704_ZN_6055
1my1_C_ZN_703_ZN_6056
1my1_C_ZN_705_ZN_6057
1my2_A_ZN_701_ZN_6053
1my2_B_ZN_702_ZN_6067
1my2_B_ZN_704_ZN_6068
1my2_C_ZN_703_ZN_6082
1my2_C_ZN_705_ZN_6083
1my3_A_ZN_701_ZN_5885
1my3_B_ZN_702_ZN_5901
1my3_B_ZN_704_ZN_5902
1my3_C_ZN_703_ZN_5918
1my3_C_ZN_705_ZN_5919
1my4_A_ZN_701_ZN_5830
1my4_B_ZN_702_ZN_5846
1my4_B_ZN_704_ZN_5847
1my4_C_ZN_703_ZN_5863
1my4_C_ZN_705_ZN_5864
1myr_A_ZN_992_ZN_4269
1mzb_A_ZN_202_ZN_1041
1mzb_A_ZN_203_ZN_1042
1mzb_A_ZN_204_ZN_1043
1n08_A_ZN_501_ZN_2534
1n08_B_ZN_601_ZN_2562
1n34_N_ZN_307_ZN_51779
1n5n_A_ZN_401_ZN_2678
1n5n_B_ZN_402_ZN_2691
1ncr_A_ZN_6000_ZN_6341
1nd2_A_ZN_6000_ZN_6338
1nd3_A_ZN_6000_ZN_6341
1nik_A_ZN_1734_ZN_28301
1njq_A_ZN_64_ZN_631
1nkx_A_ZN_691_ZN_2721
1nkx_A_ZN_692_ZN_2722
1nl5_A_ZN_500_ZN_2857
1nlc_A_ZN_50_ZN_1052
1nlc_A_ZN_54_ZN_1051
1nlc_B_ZN_51_ZN_1055
1nlc_B_ZN_52_ZN_1056
1nlc_B_ZN_53_ZN_1053
1nlc_B_ZN_55_ZN_1054
1nlx_A_ZN_5001_ZN_11131,1nlx_B_ZN_5002_ZN_11132
1nlx_G_ZN_5007_ZN_11137,1nlx_H_ZN_5008_ZN_11138
1nlx_G_ZN_6007_ZN_11151
1nlx_H_ZN_6008_ZN_11152
1nlx_I_ZN_5009_ZN_11139,1nlx_J_ZN_5010_ZN_11140
1nlx_I_ZN_6009_ZN_11153
1nlx_J_ZN_6010_ZN_11154
1nlx_K_ZN_5011_ZN_11141,1nlx_L_ZN_5012_ZN_11142
1nlx_K_ZN_6011_ZN_11155

1nlx_L_ZN_6012_ZN_11156
1nlx_M_ZN_5013_ZN_11143,1nlx_N_ZN_5014_ZN_11144
1nlx_A_ZN_6001_ZN_11145
1nlx_M_ZN_6013_ZN_11157
1nlx_N_ZN_6014_ZN_11158
1nlx_B_ZN_6002_ZN_11146
1nlx_C_ZN_5003_ZN_11133,1nlx_D_ZN_5004_ZN_11134
1nlx_C_ZN_6003_ZN_11147
1nlx_D_ZN_6004_ZN_11148
1nlx_E_ZN_5005_ZN_11135,1nlx_F_ZN_5006_ZN_11136
1nlx_E_ZN_6005_ZN_11149
1nlx_F_ZN_6006_ZN_11150
1nnk_A_ZN_451_ZN_2060
1nnk_A_ZN_452_ZN_2061
1nnq_A_ZN_200_ZN_2617
1nnq_A_ZN_201_ZN_2618,1nnq_B_ZN_202_ZN_2622
1nnq_A_ZN_202_ZN_2619,1nnq_B_ZN_201_ZN_2621
1nnq_B_ZN_200_ZN_2620
1no5_A_ZN_501_ZN_1656,1no5_A_ZN_511_ZN_1657,1no5_A_ZN_521_ZN_1658,1no5_A_
ZN_531_ZN_1659
1no5_A_ZN_561_ZN_1660
1no5_B_ZN_541_ZN_1667,1no5_B_ZN_551_ZN_1668
1no5_B_ZN_571_ZN_1669
1noy_B_ZN_2000_ZN_5953
1nvt_A_ZN_290_ZN_4403
1nvt_B_ZN_291_ZN_4452
1nw2_A_ZN_6001_ZN_6489
1nw2_B_ZN_6002_ZN_6498
1nw2_C_ZN_6003_ZN_6507
1nw2_D_ZN_6004_ZN_6522,1nw2_D_ZN_6009_ZN_6523
1nw2_E_ZN_6005_ZN_6524
1nw2_F_ZN_6006_ZN_6529
1nw2_G_ZN_6007_ZN_6534
1nw2_H_ZN_6008_ZN_6539
1nwp_A_ZN_401_ZN_1920
1nwp_A_ZN_402_ZN_1921
1nwp_B_ZN_403_ZN_1923
1nwp_B_ZN_404_ZN_1924
1nze_A_ZN_150_ZN_878
1o06_A_ZN_101_ZN_174,1o06_A_ZN_102_ZN_175
1o06_A_ZN_103_ZN_176,1o06_A_ZN_103_ZN_177
1oah_A_ZN_1528_ZN_7931
1oah_A_ZN_1529_ZN_7932
1oao_C_ZN_1731_ZN_21981
1ocy_A_ZN_1531_ZN_1585
1odz_A_ZN_1423_ZN_6104
1odz_B_ZN_1423_ZN_6137
1oek_A_ZN_1196_ZN_1518
1oek_A_ZN_1197_ZN_1519
1oep_A_ZN_500_ZN_3213
1oep_A_ZN_501_ZN_3214
1ows_A_ZN_1001_ZN_1798
1ows_A_ZN_1002_ZN_1799
1ozu_A_ZN_354_ZN_2000
1ozu_B_ZN_356_ZN_2007
1p1q_A_ZN_501_ZN_5892
1p1q_B_ZN_502_ZN_5906
1p1q_B_ZN_504_ZN_5907

1p1q_C_ZN_503_ZN_5921
1p1q_C_ZN_505_ZN_5922
1p1v_A_ZN_201_ZN_3189
1p1v_B_ZN_203_ZN_3196
1p1v_C_ZN_205_ZN_3203
1p26_A_ZN_101_ZN_606
1p26_B_ZN_102_ZN_607
1p26_C_ZN_103_ZN_608
1p26_D_ZN_104_ZN_609
1p26_E_ZN_105_ZN_610
1p42_A_ZN_503_ZN_4407
1p42_A_ZN_504_ZN_4408
1p42_B_ZN_507_ZN_4427
1p5d_X_ZN_500_ZN_3563
1p5g_X_ZN_500_ZN_3540
1pcj_X_ZN_500_ZN_3479
1pcm_X_ZN_500_ZN_3420
1peg_B_ZN_5_ZN_3567,1peg_B_ZN_6_ZN_3568,1peg_B_ZN_7_ZN_3569
1pgu_A_ZN_616_ZN_9371
1pgu_B_ZN_616_ZN_9372
1pi6_A_ZN_650_ZN_4677
1pjp_A_ZN_1000_ZN_1815
1pp7_E_ZN_74_ZN_1437
1pp7_F_ZN_72_ZN_1438
1pp7_F_ZN_73_ZN_1439,1pp7_F_ZN_77_ZN_1441
1pp7_F_ZN_76_ZN_1440
1pp7_U_ZN_132_ZN_1442
1pp7_U_ZN_133_ZN_1443
1pqv_A_ZN_1735_ZN_4046
1pqv_A_ZN_1736_ZN_4047
1pqv_B_ZN_1300_ZN_4048
1pqv_C_ZN_319_ZN_4049
1pqv_I_ZN_200_ZN_4050
1pqv_I_ZN_201_ZN_4051
1pqv_J_ZN_100_ZN_4052
1pqv_L_ZN_100_ZN_4053
1pqv_S_ZN_400_ZN_4054
1pv8_A_ZN_401_ZN_4241
1pv8_B_ZN_401_ZN_4258
1pv9_A_ZN_401_ZN_5199,1pv9_A_ZN_402_ZN_5200
1pv9_B_ZN_401_ZN_5201,1pv9_B_ZN_402_ZN_5202
1pvw_A_ZN_401_ZN_5221
1pvw_B_ZN_501_ZN_5228
1pvy_A_ZN_502_ZN_3487
1pvy_A_ZN_703_ZN_3491
1pvy_B_ZN_602_ZN_3506
1py0_A_ZN_124_ZN_923
1py2_B_ZN_601_ZN_3837
1py2_B_ZN_801_ZN_3839
1py2_C_ZN_701_ZN_3838
1q0a_A_ZN_405_ZN_1451
1q1y_A_ZN_350_ZN_1474
1q7h_A_ZN_201_ZN_1169
1q9l_A_ZN_214_ZN_6805
1q9l_C_ZN_214_ZN_6807
1q9l_C_ZN_215_ZN_6808
1q9q_A_ZN_218_ZN_3406
1q9r_B_ZN_306_ZN_3438

1q9t_A_ZN_218_ZN_3420
1q9u_A_ZN_401_ZN_2101
1q9u_B_ZN_408_ZN_2112
1q9u_B_ZN_409_ZN_2113
1q9u_B_ZN_410_ZN_2114
1q9u_B_ZN_414_ZN_2115
1q9u_B_ZN_415_ZN_2116
1q9u_B_ZN_416_ZN_2117
1q9u_A_ZN_403_ZN_2102,1q9u_A_ZN_411_ZN_2104
1q9u_A_ZN_407_ZN_2103,1q9u_A_ZN_413_ZN_2106
1q9u_A_ZN_412_ZN_2105
1q9u_A_ZN_417_ZN_2107
1q9u_B_ZN_402_ZN_2108
1q9u_B_ZN_404_ZN_2109
1q9u_B_ZN_405_ZN_2110
1q9u_B_ZN_406_ZN_2111
1q9v_B_ZN_304_ZN_3421
1qe3_A_ZN_500_ZN_3650
1qhd_A_ZN_501_ZN_3178
1qhw_A_ZN_435_ZN_2415
1qhw_A_ZN_436_ZN_2416
1qju_1_ZN_6000_ZN_6341
1qjx_1_ZN_6000_ZN_6341
1qjy_1_ZN_6000_ZN_6341
1qp9_B_ZN_133_ZN_4022
1qu2_A_ZN_1992_ZN_9025
1qu3_A_ZN_992_ZN_8719
1qvn_A_ZN_701_ZN_3922
1qvn_B_ZN_601_ZN_3921
1qvn_B_ZN_801_ZN_3923
1qvn_C_ZN_901_ZN_3924
1qx2_B_ZN_1003_ZN_1234
1qx2_B_ZN_1004_ZN_1235
1qyb_A_ZN_401_ZN_1519
1r1v_A_ZN_502_ZN_1520
1r1v_A_ZN_503_ZN_1521
1r1v_A_ZN_504_ZN_1522
1r4m_H_ZN_4_ZN_31632
1r4m_J_ZN_2_ZN_31630
1r4n_H_ZN_4_ZN_31632
1r4n_J_ZN_2_ZN_31630
1r4v_A_ZN_201_ZN_1228
1r4v_A_ZN_202_ZN_1229
1r4v_A_ZN_204_ZN_1235
1r5u_A_ZN_1734_ZN_28302
1r5u_I_ZN_203_ZN_28306
1r5u_M_ZN_209_ZN_28310
1r7o_A_ZN_1001_ZN_2918
1r7o_A_ZN_1002_ZN_2919
1r7o_A_ZN_1003_ZN_2920
1r7o_A_ZN_1004_ZN_2921,1r7o_A_ZN_1004_ZN_2922
1r85_A_ZN_901_ZN_3174
1r85_A_ZN_902_ZN_3175
1r85_A_ZN_903_ZN_3176,1r85_A_ZN_907_ZN_3180
1r85_A_ZN_904_ZN_3177
1r85_A_ZN_905_ZN_3178
1r85_A_ZN_906_ZN_3179
1r86_A_ZN_901_ZN_3146

1r86_A_ZN_902_ZN_3147
1r86_A_ZN_903_ZN_3148,1r86_A_ZN_904_ZN_3149
1r86_A_ZN_905_ZN_3150
1r86_A_ZN_906_ZN_3151
1r86_A_ZN_907_ZN_3152
1r87_A_ZN_901_ZN_3136
1r87_A_ZN_902_ZN_3137
1r87_A_ZN_903_ZN_3138,1r87_A_ZN_904_ZN_3139
1r87_A_ZN_905_ZN_3140
1r87_A_ZN_906_ZN_3141
1r87_A_ZN_907_ZN_3142
1r8q_E_ZN_601_ZN_6093
1r9p_A_ZN_135_ZN_2011
1r9s_A_ZN_1734_ZN_28465
1rft_A_ZN_403_ZN_2415
1rfu_A_ZN_403_ZN_19521
1rfu_B_ZN_1403_ZN_19564
1rfu_C_ZN_2403_ZN_19607
1rfu_D_ZN_3403_ZN_19650
1rfu_E_ZN_4403_ZN_19693
1rfu_F_ZN_5403_ZN_19736
1rfu_G_ZN_6403_ZN_19779
1rfu_H_ZN_7403_ZN_19822
1rfv_A_ZN_402_ZN_4801
1rfv_B_ZN_1402_ZN_4829
1rh2_A_ZN_1204_ZN_797
1rh2_B_ZN_1201_ZN_794
1rh2_C_ZN_1203_ZN_796
1rh2_E_ZN_1202_ZN_795
1rhf_A_ZN_301_ZN_2734
1ro0_A_ZN_302_ZN_1706
1ro2_A_ZN_302_ZN_1706
1ro5_A_ZN_405_ZN_1592
1rp0_A_ZN_500_ZN_4165
1rp0_B_ZN_501_ZN_4214
1rpj_A_ZN_289_ZN_2135
1rqg_A_ZN_802_ZN_4976
1ruq_L_ZN_601_ZN_3311
1ruq_L_ZN_602_ZN_3312
1rur_L_ZN_601_ZN_3311
1rur_L_ZN_602_ZN_3312
1ryq_A_ZN_101_ZN_491
1s03_A_ZN_48_ZN_3934
1s03_B_ZN_50_ZN_3944,1s03_B_ZN_52_ZN_3946,1s03_B_ZN_53_ZN_3947
1s03_B_ZN_51_ZN_3945
1s03_G_ZN_130_ZN_3949
1s03_G_ZN_131_ZN_3950
1s03_H_ZN_130_ZN_3948
1s03_A_ZN_49_ZN_3935
1s03_A_ZN_50_ZN_3936
1s03_A_ZN_51_ZN_3937
1s03_A_ZN_52_ZN_3938
1s03_A_ZN_53_ZN_3939,1s03_A_ZN_55_ZN_3941
1s03_A_ZN_54_ZN_3940
1s03_B_ZN_48_ZN_3942
1s03_B_ZN_49_ZN_3943
1s2z_A_ZN_601_ZN_1517
1s30_A_ZN_601_ZN_1517

1s3q_A_ZN_901_ZN_16146,1s3q_A_ZN_902_ZN_16147
1s3q_J_ZN_919_ZN_16164,1s3q_J_ZN_920_ZN_16165
1s3q_K_ZN_921_ZN_16166,1s3q_K_ZN_922_ZN_16167
1s3q_L_ZN_923_ZN_16168,1s3q_L_ZN_924_ZN_16169
1s3q_B_ZN_903_ZN_16148,1s3q_B_ZN_904_ZN_16149
1s3q_C_ZN_905_ZN_16150,1s3q_C_ZN_906_ZN_16151
1s3q_D_ZN_907_ZN_16152,1s3q_D_ZN_908_ZN_16153
1s3q_E_ZN_909_ZN_16154,1s3q_E_ZN_910_ZN_16155
1s3q_F_ZN_911_ZN_16156,1s3q_F_ZN_912_ZN_16157
1s3q_G_ZN_913_ZN_16158,1s3q_G_ZN_914_ZN_16159
1s3q_H_ZN_915_ZN_16160,1s3q_H_ZN_916_ZN_16161
1s3q_I_ZN_917_ZN_16162,1s3q_I_ZN_918_ZN_16163
1s4i_A_ZN_802_ZN_4530
1s4i_B_ZN_801_ZN_4527
1s7d_A_ZN_202_ZN_1530
1s7d_A_ZN_203_ZN_1531
1s7d_A_ZN_204_ZN_1532
1s7g_A_ZN_602_ZN_9543
1s7g_B_ZN_604_ZN_9629
1s7g_C_ZN_606_ZN_9731
1s7g_D_ZN_608_ZN_9819
1s9z_A_ZN_102_ZN_145
1sdx_A_ZN_1002_ZN_2737
1sdx_A_ZN_1003_ZN_2738
1sdx_A_ZN_677_ZN_2736
1sed_A_ZN_501_ZN_2792
1sed_B_ZN_502_ZN_2854
1sed_C_ZN_503_ZN_2908
1sgf_A_ZN_1_ZN_8554
1sgf_X_ZN_501_ZN_8555
1shw_A_ZN_1001_ZN_2660
1slx_B_ZN_248_ZN_2682
1snn_A_ZN_402_ZN_3487
1snn_A_ZN_603_ZN_3491
1snn_B_ZN_502_ZN_3506
1su0_B_ZN_160_ZN_1028
1sv2_A_ZN_1001_ZN_2691
1sv2_B_ZN_1001_ZN_2700
1sw1_A_ZN_401_ZN_4325
1sw1_A_ZN_402_ZN_4326
1sw1_A_ZN_404_ZN_4327
1sw1_A_ZN_406_ZN_4328
1sw1_B_ZN_403_ZN_4339
1sw1_B_ZN_405_ZN_4340
1sw4_A_ZN_402_ZN_4325
1sw4_A_ZN_404_ZN_4326
1sw4_A_ZN_406_ZN_4327
1sw4_B_ZN_401_ZN_4333
1sw4_B_ZN_403_ZN_4334
1sw4_B_ZN_405_ZN_4335
1sw4_B_ZN_409_ZN_4337
1szz_A_ZN_716_ZN_10525
1szz_B_ZN_717_ZN_10553
1szz_C_ZN_718_ZN_10581
1szz_D_ZN_719_ZN_10609
1szz_E_ZN_720_ZN_10637
1szz_F_ZN_721_ZN_10665
1szz_G_ZN_722_ZN_10693

1szz_H_ZN_723_ZN_10721
1t0b_A_ZN_3001_ZN_15720
1t0b_B_ZN_3002_ZN_15721
1t0b_C_ZN_3003_ZN_15722
1t0b_D_ZN_3008_ZN_15723
1t0b_E_ZN_3005_ZN_15724
1t0b_F_ZN_3006_ZN_15725
1t0b_G_ZN_3007_ZN_15726
1t0b_H_ZN_3004_ZN_15727
1t4k_A_ZN_304_ZN_6601
1t4k_A_ZN_305_ZN_6602
1t4k_B_ZN_303_ZN_6603
1t4k_B_ZN_308_ZN_6604
1t4k_B_ZN_309_ZN_6605
1t4k_C_ZN_301_ZN_6606
1t4k_C_ZN_302_ZN_6607
1t4k_C_ZN_306_ZN_6608
1t4k_D_ZN_307_ZN_6609
1t5l_A_ZN_659_ZN_9615
1t5l_A_ZN_660_ZN_9616
1t5l_B_ZN_659_ZN_9617
1t5l_B_ZN_660_ZN_9618
1t8k_A_ZN_201_ZN_609
1t8k_A_ZN_202_ZN_610
1t8k_A_ZN_203_ZN_611
1t8k_A_ZN_204_ZN_612
1t8k_A_ZN_205_ZN_613
1t8k_A_ZN_206_ZN_614,1t8k_A_ZN_207_ZN_615
1t8k_A_ZN_208_ZN_616
1t8k_A_ZN_209_ZN_617
1t8k_A_ZN_210_ZN_618
1t92_A_ZN_141_ZN_1689
1taf_A_ZN_2002_ZN_1088
1taf_A_ZN_2003_ZN_1089
1taf_A_ZN_2004_ZN_1090
1taf_A_ZN_2005_ZN_1091
1taf_B_ZN_2001_ZN_1092
1taf_B_ZN_2006_ZN_1093
1taf_B_ZN_2007_ZN_1094
1taq_A_ZN_900_ZN_6454
1teh_B_ZN_378_ZN_5595
1tlg_A_ZN_301_ZN_1942
1tlg_A_ZN_302_ZN_1943
1tlg_A_ZN_303_ZN_1944
1tlg_B_ZN_301_ZN_1959
1tlg_B_ZN_302_ZN_1960
1tq7_A_ZN_401_ZN_2413
1tq7_A_ZN_402_ZN_2414
1tq7_B_ZN_403_ZN_2429
1ttm_A_ZN_263_ZN_2061
1ty2_A_ZN_700_ZN_5166
1ty2_B_ZN_701_ZN_5167
1ty2_C_ZN_702_ZN_5168
1u05_A_ZN_500_ZN_3713
1u05_B_ZN_501_ZN_3714
1u0a_A_ZN_5005_ZN_6897
1u0a_A_ZN_5011_ZN_6898
1u0a_B_ZN_5006_ZN_6945

1u10_A_ZN_601_ZN_11230
1u10_D_ZN_602_ZN_11249
1u10_E_ZN_603_ZN_11256
1u19_A_ZN_2011_ZN_5574
1u19_A_ZN_957_ZN_5571
1u19_A_ZN_959_ZN_5572
1u19_A_ZN_962_ZN_5573
1u19_B_ZN_956_ZN_5745
1u19_B_ZN_958_ZN_5746
1u19_B_ZN_963_ZN_5747
1u1h_A_ZN_767_ZN_5786
1u1j_A_ZN_767_ZN_5786
1u1u_A_ZN_766_ZN_5785
1u1u_A_ZN_767_ZN_5786
1u22_A_ZN_767_ZN_5786
1u74_A_ZNH_1001_ZN_6449
1u74_C_ZNH_1201_ZN_6540
1u75_A_ZNH_1001_ZN_5582
1u75_C_ZNH_1201_ZN_5673
1u7j_A_ZN_50_ZN_1653,1u7j_B_ZN_150_ZN_1654
1u7m_A_ZN_54_ZN_1829,1u7m_B_ZN_154_ZN_1830
1u9k_A_ZN_135_ZN_1787
1u9k_A_ZN_136_ZN_1788
1u9k_B_ZN_135_ZN_1789
1ud9_A_ZN_501_ZN_7519,1ud9_A_ZN_502_ZN_7520
1ud9_B_ZN_522_ZN_7529
1ud9_B_ZN_523_ZN_7530
1ud9_C_ZN_509_ZN_7531
1ud9_C_ZN_511_ZN_7532
1ud9_C_ZN_513_ZN_7533
1ud9_C_ZN_520_ZN_7534
1ud9_C_ZN_521_ZN_7535
1ud9_D_ZN_507_ZN_7537
1ud9_D_ZN_514_ZN_7538
1ud9_D_ZN_517_ZN_7539
1ud9_A_ZN_503_ZN_7521
1ud9_D_ZN_518_ZN_7540
1ud9_D_ZN_519_ZN_7541
1ud9_A_ZN_505_ZN_7522,1ud9_D_ZN_504_ZN_7536
1ud9_A_ZN_506_ZN_7523
1ud9_A_ZN_512_ZN_7524
1ud9_A_ZN_515_ZN_7525
1ud9_A_ZN_516_ZN_7526
1ud9_B_ZN_508_ZN_7527
1ud9_B_ZN_510_ZN_7528
1udv_A_ZN_101_ZN_1411
1ue1_B_ZN_400_ZN_1728
1uqw_A_ZN_500_ZN_7635
1uqw_A_ZN_501_ZN_7636,1uqw_A_ZN_502_ZN_7637
1uqw_A_ZN_503_ZN_7638,1uqw_A_ZN_504_ZN_7639
1uqw_A_ZN_505_ZN_7640
1uqw_A_ZN_506_ZN_7641,1uqw_A_ZN_507_ZN_7642
1uqw_A_ZN_508_ZN_7643
1uqw_A_ZN_509_ZN_7644
1uqw_B_ZN_510_ZN_7657
1uqw_B_ZN_511_ZN_7658
1usn_A_ZN_262_ZN_1336
1ut8_A_ZN_303_ZN_4402

1ut8_B_ZN_303_ZN_4403
1uv0_A_ZN_1150_ZN_1079
1uv0_A_ZN_1151_ZN_1080
1uv0_A_ZN_1152_ZN_1081
1uvq_B_ZN_1192_ZN_3111
1uw1_A_ZN_1075_ZN_597
1uxa_A_ZN_1367_ZN_4385
1uxa_B_ZN_1367_ZN_4418
1uxa_C_ZN_1367_ZN_4455
1uxb_A_ZN_1367_ZN_4373
1uxb_B_ZN_1367_ZN_4414
1uxb_C_ZN_1366_ZN_4427
1uxe_A_ZN_1366_ZN_4353
1uxe_B_ZN_1366_ZN_4358
1uxe_C_ZN_1366_ZN_4359
1v15_E_ZN_1009_ZN_5138
1v15_G_ZN_1009_ZN_5139
1v15_I_ZN_1009_ZN_5140
1v15_K_ZN_1009_ZN_5141
1v48_A_ZN_301_ZN_2036
1v51_A_ZN_611_ZN_3609
1v51_A_ZN_603_ZN_3601
1v51_A_ZN_604_ZN_3602
1v51_A_ZN_605_ZN_3603
1v51_A_ZN_606_ZN_3604
1v51_A_ZN_607_ZN_3605
1v51_A_ZN_608_ZN_3606
1v51_A_ZN_609_ZN_3607
1v51_A_ZN_610_ZN_3608
1v5r_A_ZN_201_ZN_1490
1v72_A_ZN_401_ZN_2582
1v8d_A_ZN_1402_ZN_4245
1v8d_A_ZN_401_ZN_4244
1v8n_A_ZN_300_ZN_1208
1v8r_A_ZN_301_ZN_1222
1v8r_A_ZN_302_ZN_1223
1v8t_A_ZN_300_ZN_1232
1v8t_A_ZN_301_ZN_1233
1v8w_A_ZN_300_ZN_1240
1v8y_A_ZN_300_ZN_1208
1v8y_A_ZN_301_ZN_1209
1vcv_A_ZN_1002_ZN_3463
1vcv_B_ZN_1001_ZN_3464
1ve0_A_ZN_136_ZN_1056
1vec_A_ZN_1001_ZN_3235
1vev_A_ZN_1513_ZN_2691
1vev_B_ZN_2513_ZN_2719
1vey_A_ZN_1513_ZN_2677
1vey_B_ZN_2514_ZN_2693
1vez_A_ZN_1513_ZN_2736
1vez_B_ZN_2513_ZN_2776
1vf2_B_ZN_6000_ZN_3681
1vfn_A_ZN_434_ZN_2105
1vk9_A_ZN_140_ZN_1153
1vk9_A_ZN_141_ZN_1154
1vk9_A_ZN_142_ZN_1155
1vk9_A_ZN_143_ZN_1156
1vk9_A_ZN_144_ZN_1157

1vk9_A_ZN_145_ZN_1158
1vli_A_ZN_374_ZN_2741
1vpy_A_ZN_278_ZN_2000
1vpy_A_ZN_279_ZN_2001
1vpy_A_ZN_280_ZN_2002
1vpy_A_ZN_281_ZN_2003
1vs0_A_ZN_9002_ZN_4874
1vs0_A_ZN_9004_ZN_4875
1vs0_B_ZN_9001_ZN_4879
1vs0_B_ZN_9003_ZN_4880
1vsh_A_ZN_280_ZN_1129,1vsh_A_ZN_281_ZN_1130
1vsh_A_ZN_285_ZN_1131
1vsh_A_ZN_289_ZN_1132
1vsl_A_ZN_200_ZN_1127
1vsl_A_ZN_201_ZN_1128
1vyk_A_ZN_1150_ZN_1037
1vyk_A_ZN_1151_ZN_1038
1w25_A_ZN_499_ZN_6965
1w7v_A_ZN_1440_ZN_14093
1w7v_B_ZN_1440_ZN_14096
1w7v_C_ZN_1440_ZN_14100
1w7v_D_ZN_1440_ZN_14103
1w9b_M_ZN_1502_ZN_4338
1w9d_M_ZN_1502_ZN_4314
1wa0_X_ZN_1339_ZN_2550
1wa0_X_ZN_1340_ZN_2551
1wa1_X_ZN_503_ZN_2563
1wa2_X_ZN_1339_ZN_2542
1wa2_X_ZN_1340_ZN_2543
1waa_A_ZN_1090_ZN_4222
1waa_B_ZN_1094_ZN_4231
1waa_C_ZN_1090_ZN_4232
1waa_C_ZN_1091_ZN_4233
1waa_C_ZN_1092_ZN_4234
1waa_C_ZN_1093_ZN_4235,1waa_E_ZN_1090_ZN_4241
1waa_C_ZN_1094_ZN_4236
1waa_D_ZN_1090_ZN_4237
1waa_D_ZN_1091_ZN_4238
1waa_D_ZN_1092_ZN_4239
1waa_E_ZN_1089_ZN_4240
1waa_A_ZN_1091_ZN_4223
1waa_E_ZN_1091_ZN_4242
1waa_F_ZN_1090_ZN_4243
1waa_F_ZN_1091_ZN_4244
1waa_F_ZN_1092_ZN_4245
1waa_A_ZN_1092_ZN_4224
1waa_A_ZN_1093_ZN_4225
1waa_A_ZN_1094_ZN_4226
1waa_B_ZN_1090_ZN_4227
1waa_B_ZN_1091_ZN_4228
1waa_B_ZN_1092_ZN_4229
1waa_B_ZN_1093_ZN_4230
1wae_A_ZN_503_ZN_2549
1wbq_A_ZN_1440_ZN_13973
1wbq_B_ZN_1440_ZN_13976
1wbq_C_ZN_1440_ZN_13980
1wbq_D_ZN_1440_ZN_13983
1wcz_A_ZN_300_ZN_1644

1wdk_A_ZN_716_ZN_16444
1wdm_A_ZN_716_ZN_16304
1wej_L_ZN_215_ZN_4170
1wfx_A_ZN_201_ZN_1444
1wfz_A_ZN_201_ZN_1897
1wo3_A_ZN_26_ZN_335
1wo4_A_ZN_26_ZN_332
1wo5_A_ZN_26_ZN_369
1wo6_A_ZN_26_ZN_372
1wo7_A_ZN_26_ZN_373
1wpp_A_ZN_601_ZN_4768,1wpp_A_ZN_602_ZN_4769
1wpp_B_ZN_603_ZN_4785,1wpp_B_ZN_604_ZN_4786
1ww1_A_ZN_301_ZN_4007
1ww1_B_ZN_302_ZN_4008
1wy2_A_ZN_405_ZN_5518,1wy2_A_ZN_406_ZN_5519
1wy2_B_ZN_407_ZN_5538,1wy2_B_ZN_408_ZN_5539
1x1c_A_ZN_5001_ZN_2657,1x1c_A_ZN_5004_ZN_2660
1x1c_A_ZN_5002_ZN_2658
1x1c_A_ZN_5003_ZN_2659
1x1c_A_ZN_5005_ZN_2661
1x1d_A_ZN_5001_ZN_2657
1x1v_A_ZN_901_ZN_2092
1x1v_A_ZN_903_ZN_2093
1x1v_B_ZN_902_ZN_2144
1x68_A_ZN_401_ZN_1045
1x6a_A_ZN_401_ZN_1149
1xaf_A_ZN_501_ZN_3691
1xaf_A_ZN_502_ZN_3692
1xaf_A_ZN_503_ZN_3693
1xaf_A_ZN_504_ZN_3694
1xaf_A_ZN_508_ZN_3695
1xaf_B_ZN_505_ZN_3710
1xaf_B_ZN_506_ZN_3711
1xaf_B_ZN_507_ZN_3712
1xb0_A_ZN_400_ZN_4833
1xb0_C_ZN_600_ZN_4840
1xb0_C_ZN_601_ZN_4841
1xb0_D_ZN_602_ZN_4843
1xb0_D_ZN_700_ZN_4844
1xb0_D_ZN_701_ZN_4845
1xb0_E_ZN_702_ZN_4847
1xb0_E_ZN_800_ZN_4848
1xb0_E_ZN_801_ZN_4849
1xb0_A_ZN_401_ZN_4832
1xb0_F_ZN_802_ZN_4851
1xb0_F_ZN_900_ZN_4852
1xb0_F_ZN_901_ZN_4853
1xb0_A_ZN_902_ZN_4834
1xb0_B_ZN_402_ZN_4835
1xb0_B_ZN_500_ZN_4836
1xb0_B_ZN_501_ZN_4837
1xb0_C_ZN_502_ZN_4839
1xb1_A_ZN_400_ZN_4807
1xb1_C_ZN_601_ZN_4816
1xb1_C_ZN_602_ZN_4817
1xb1_D_ZN_700_ZN_4819
1xb1_D_ZN_701_ZN_4820
1xb1_E_ZN_702_ZN_4822

1xb1_E_ZN_800_ZN_4823
1xb1_E_ZN_801_ZN_4824
1xb1_A_ZN_401_ZN_4808
1xb1_F_ZN_802_ZN_4826
1xb1_F_ZN_900_ZN_4827
1xb1_F_ZN_901_ZN_4828
1xb1_A_ZN_902_ZN_4810
1xb1_B_ZN_402_ZN_4811
1xb1_B_ZN_500_ZN_4812
1xb1_B_ZN_501_ZN_4813
1xb1_B_ZN_502_ZN_4814
1xb1_C_ZN_600_ZN_4815
1xb8_A_ZN_1001_ZN_1920
1xb8_C_ZN_5001_ZN_1921
1xem_A_ZN_401_ZN_1317
1xjs_A_ZN_150_ZN_2244
1xkf_A_ZN_1001_ZN_1816
1xkf_A_ZN_1002_ZN_1817
1xki_A_ZN_1001_ZN_984
1xki_A_ZN_1002_ZN_985
1xki_A_ZN_1003_ZN_986
1xoc_A_ZN_521_ZN_4197
1xoc_A_ZN_530_ZN_4206
1xoc_A_ZN_531_ZN_4207
1xoc_A_ZN_532_ZN_4208
1xoc_A_ZN_533_ZN_4209
1xoc_A_ZN_534_ZN_4210
1xoc_A_ZN_522_ZN_4198
1xoc_A_ZN_523_ZN_4199
1xoc_A_ZN_524_ZN_4200
1xoc_A_ZN_525_ZN_4201,1xoc_A_ZN_535_ZN_4211
1xoc_A_ZN_526_ZN_4202
1xoc_A_ZN_527_ZN_4203
1xoc_A_ZN_528_ZN_4204
1xoc_A_ZN_529_ZN_4205
1xpz_A_ZN_263_ZN_2061
1xq0_A_ZN_263_ZN_2061
1xru_A_ZN_501_ZN_4402
1xru_B_ZN_502_ZN_4419
1xtl_D_ZN_1330_ZN_4562
1xtl_A_ZN_1327_ZN_4555
1xtl_A_ZN_1331_ZN_4556
1xtl_B_ZN_1326_ZN_4551
1xtl_B_ZN_1329_ZN_4552
1xtl_C_ZN_1328_ZN_4559
1xtm_B_ZN_502_ZN_2279
1xtm_B_ZN_505_ZN_2280
1xtm_B_ZN_506_ZN_2281
1xuf_A_BAZ_246_ZN_2121
1xug_A_ZN_409_ZN_3379
1xuj_A_BOZ_246_ZN_2076
1xvh_A_ZN_1302_ZN_1747,1xvh_A_ZN_1305_ZN_1748
1xvh_B_ZN_1307_ZN_1762
1xvh_B_ZN_1309_ZN_1763
1xvh_B_ZN_1311_ZN_1764
1xvh_B_ZN_1312_ZN_1765
1xvh_B_ZN_1314_ZN_1766
1xvh_A_ZN_1306_ZN_1749

1xvh_A_ZN_1308_ZN_1750
1xvh_A_ZN_1310_ZN_1751
1xvh_A_ZN_1313_ZN_1752
1xvh_A_ZN_1315_ZN_1753
1xvh_A_ZN_1316_ZN_1754,1xvh_B_ZN_1301_ZN_1759
1xvh_B_ZN_1303_ZN_1760
1xvh_B_ZN_1304_ZN_1761
1xvx_A_ZN_314_ZN_2387
1xvx_A_ZN_315_ZN_2388
1xvx_A_ZN_316_ZN_2389
1xvx_A_ZN_317_ZN_2390
1xvx_A_ZN_318_ZN_2391
1xwy_A_ZN_401_ZN_2040
1xx4_A_ZN_400_ZN_2023
1xx4_A_ZN_401_ZN_2024,1xx4_A_ZN_402_ZN_2025
1xxw_A_ZN_201_ZN_1840
1y1w_A_ZN_1734_ZN_31809
1y6h_A_ZN_513_ZN_2806
1y6h_B_ZN_513_ZN_2816
1y75_A_ZN_401_ZN_1802
1y75_A_ZN_402_ZN_1803
1y77_A_ZN_1734_ZN_31810
1y77_I_ZN_204_ZN_31815
1y7p_A_ZN_301_ZN_4910
1y7p_B_ZN_303_ZN_4921
1y7p_C_ZN_302_ZN_4932
1y7w_B_ZN_285_ZN_4261
1y9a_A_ZN_1002_ZN_5429
1y9a_C_ZN_2002_ZN_5445
1yc2_A_ZN_402_ZN_9752
1yc2_B_ZN_404_ZN_9829
1yc2_C_ZN_406_ZN_9940
1yc2_D_ZN_408_ZN_10029
1ycf_A_ZN_502_ZN_12465
1ycf_A_ZN_503_ZN_12466
1ycf_B_ZN_512_ZN_12503
1ycg_A_ZN_401_ZN_12465
1ycg_C_ZN_422_ZN_12550
1ycg_C_ZN_423_ZN_12551
1ycg_D_ZN_431_ZN_12590
1ycg_D_ZN_432_ZN_12591
1ycg_D_ZN_433_ZN_12592
1ycg_A_ZN_402_ZN_12466
1ycg_A_ZN_403_ZN_12467
1ycg_A_ZN_404_ZN_12468
1ycg_B_ZN_411_ZN_12507
1ycg_B_ZN_412_ZN_12508
1ycg_B_ZN_413_ZN_12509
1ycg_B_ZN_414_ZN_12510
1ycg_C_ZN_421_ZN_12549
1ych_A_ZN_600_ZN_12465
1ych_A_ZN_601_ZN_12466
1ych_B_ZN_610_ZN_12501
1ych_B_ZN_611_ZN_12502
1ych_C_ZN_620_ZN_12537
1ych_C_ZN_621_ZN_12538
1ych_D_ZN_630_ZN_12573
1ych_D_ZN_631_ZN_12574

1yec_L_ZN_215_ZN_3391
1yec_L_ZN_216_ZN_3392,1yec_L_ZN_218_ZN_3394
1yec_L_ZN_217_ZN_3393
1yec_L_ZN_219_ZN_3395,1yec_L_ZN_220_ZN_3396,1yec_L_ZN_221_ZN_3397
1yef_L_ZN_215_ZN_3391
1yef_L_ZN_216_ZN_3392,1yef_L_ZN_218_ZN_3394,1yef_L_ZN_222_ZN_3398
1yef_L_ZN_217_ZN_3393
1yef_L_ZN_219_ZN_3395,1yef_L_ZN_220_ZN_3396,1yef_L_ZN_221_ZN_3397
1yeg_L_ZN_215_ZN_3391,1yeg_L_ZN_219_ZN_3395,1yeg_L_ZN_220_ZN_3396,1yeg_L_ZN_221_ZN_3397
1yeg_L_ZN_216_ZN_3392,1yeg_L_ZN_218_ZN_3394
1yeg_L_ZN_217_ZN_3393
1yeh_L_ZN_215_ZN_3391,1yeh_L_ZN_219_ZN_3395,1yeh_L_ZN_220_ZN_3396,1yeh_L_ZN_221_ZN_3397
1yeh_L_ZN_216_ZN_3392,1yeh_L_ZN_218_ZN_3394
1yeh_L_ZN_217_ZN_3393
1yei_L_ZN_601_ZN_3394
1yei_L_ZN_602_ZN_3395,1yei_L_ZN_607_ZN_3393
1yei_L_ZN_603_ZN_3392,1yei_L_ZN_604_ZN_3396,1yei_L_ZN_605_ZN_3397,1yei_L_ZN_606_ZN_3398
1yej_L_ZN_601_ZN_3391
1yej_L_ZN_602_ZN_3395,1yej_L_ZN_604_ZN_3393
1yej_L_ZN_603_ZN_3392
1yej_L_ZN_605_ZN_3394,1yej_L_ZN_606_ZN_3396,1yej_L_ZN_607_ZN_3397
1yek_L_ZN_601_ZN_3394,1yek_L_ZN_605_ZN_3392,1yek_L_ZN_606_ZN_3398,1yek_L_ZN_607_ZN_3393
1yek_L_ZN_602_ZN_3395,1yek_L_ZN_604_ZN_3397
1yek_L_ZN_603_ZN_3396
1yew_A_ZN_800_ZN_19771
1yew_C_ZN_661_ZN_19765
1yew_C_ZN_700_ZN_19773
1yew_E_ZN_701_ZN_19774
1yew_E_ZN_702_ZN_19775,1yew_E_ZN_900_ZN_19772
1yew_G_ZN_662_ZN_19767
1yew_K_ZN_663_ZN_19769
1ygd_A_ZNH_142_ZN_4381
1ygd_C_ZNH_142_ZN_4469
1yh8_A_ZN_503_ZN_4300
1yh8_A_ZN_504_ZN_4301
1yh8_B_ZN_507_ZN_4322
1yhc_A_ZN_603_ZN_4300
1yhc_A_ZN_606_ZN_4301
1yhc_B_ZN_607_ZN_4357
1yhu_B_ZN_190_ZN_26737
1yhu_R_ZN_191_ZN_26746
1yhu_V_ZN_190_ZN_26747
1yhu_V_ZN_191_ZN_26748
1yhu_B_ZN_191_ZN_26738
1yhu_F_ZN_190_ZN_26739
1yhu_F_ZN_191_ZN_26740
1yhu_J_ZN_190_ZN_26741
1yhu_J_ZN_191_ZN_26742
1yhu_N_ZN_190_ZN_26743
1yhu_N_ZN_191_ZN_26744
1yhu_R_ZN_190_ZN_26745
1yj0_A_ZN_401_ZN_2552
1yj0_A_ZN_402_ZN_2553
1yj6_A_ZN_219_ZN_5416

1yj6_B_ZN_219_ZN_5437
1yj6_C_ZN_219_ZN_5458
1yjo_A_ZN_7_ZN_57
1yn4_A_ZN_237_ZN_756
1yn4_A_ZN_238_ZN_757
1ynn_D_ZN_1525_ZN_24314
1yo7_A_ZN_201_ZN_1913
1yt3_A_ZN_1002_ZN_3096
1yt3_A_ZN_1003_ZN_3097
1yt3_A_ZN_1004_ZN_3098
1yt3_A_ZN_1005_ZN_3099
1yxp_A_ZN_100_ZN_983
1yxp_B_ZN_101_ZN_984
1z1n_X_ZN_701_ZN_3867
1z1n_X_ZN_702_ZN_3868
1z1n_X_ZN_703_ZN_3869
1z7h_A_ZN_444_ZN_3455
1z83_A_ZN_601_ZN_4499
1z83_A_ZN_641_ZN_4502
1z83_B_ZN_611_ZN_4500
1z83_B_ZN_642_ZN_4503
1z83_B_ZN_643_ZN_4504
1z83_C_ZN_621_ZN_4501
1z83_C_ZN_644_ZN_4505
1z83_C_ZN_645_ZN_4506
1z8i_A_ZN_702_ZN_2374
1z8i_B_ZN_701_ZN_2389
1z8i_B_ZN_703_ZN_2390
1z8i_B_ZN_704_ZN_2391
1z8j_A_ZN_15_ZN_2329
1z8j_B_ZN_1_ZN_2344
1z8j_B_ZN_4_ZN_2345
1z9y_A_ZN_400_ZN_2023
1zap_A_ZN_400_ZN_3105
1zat_A_ZN_467_ZN_1942
1zde_A_ZN_290_ZN_1269
1ze9_A_ZN_18_ZN_264
1zfp_E_ZN_1_ZN_870
1zgf_A_ZN_900_ZN_2040
1zjl_A_ZN_371_ZN_2874
1zjl_A_ZN_372_ZN_2875
1zjl_A_ZN_373_ZN_2876
1zjl_A_ZN_374_ZN_2877
1zkj_A_ZN_506_ZN_2629
1zkj_A_ZN_507_ZN_2630
1zkj_A_ZN_508_ZN_2631
1zkj_A_ZN_509_ZN_2632
1zkj_A_ZN_510_ZN_2633
1zkj_A_ZN_511_ZN_2634
1zkj_A_ZN_512_ZN_2635
1zkj_A_ZN_514_ZN_2636
1zlh_A_ZN_666_ZN_2905
1zlh_A_ZN_777_ZN_2906
1zlh_A_ZN_888_ZN_2907
1zlh_A_ZN_999_ZN_2908
1zqt_A_ZN_340_ZN_2916
1zr6_A_ZN_1481_ZN_3786
1zr6_A_ZN_1482_ZN_3787

1zr6_A_ZN_1483_ZN_3788
1zr6_A_ZN_1484_ZN_3789
1zrp_A_ZN_54_ZN_790
1zv8_G_ZN_204_ZN_3688
1zv8_H_ZN_203_ZN_3694
1zxz_A_ZN_198_ZN_2965
1zxz_B_ZN_198_ZN_2966
1zy0_A_ZN_198_ZN_2945
1zy0_B_ZN_198_ZN_2946
1zy1_A_ZN_198_ZN_2993
1zy1_B_ZN_198_ZN_2994
1zyr_D_ZN_9002_ZN_54056
1zyr_N_ZN_9003_ZN_54057
1zyr_N_ZN_9004_ZN_54058
1zzh_A_ZN_402_ZN_8904
1zzh_A_ZN_801_ZN_8905
1zzh_B_ZN_502_ZN_8993
1zzh_B_ZN_503_ZN_8994
1zzh_B_ZN_504_ZN_8995
1zzh_B_ZN_802_ZN_8996
1zzh_C_ZN_602_ZN_9084
1zzh_D_ZN_702_ZN_9172
1zzm_A_ZN_403_ZN_2043
1zzm_A_ZN_404_ZN_2044
258l_A_ZN_500_ZN_1310
2a03_A_ZN_600_ZN_3166
2a0b_A_ZN_800_ZN_957
2a0s_B_ZN_381_ZN_2745
2a21_A_ZN_1270_ZN_4086
2a21_B_ZN_2270_ZN_4102
2a2i_A_ZN_1270_ZN_4081
2a2i_B_ZN_2270_ZN_4106
2a2q_H_ZN_1011_ZN_4694
2a2q_H_ZN_1012_ZN_4695
2a5v_A_ZN_400_ZN_6171
2a5v_D_ZN_407_ZN_6186
2a5v_D_ZN_410_ZN_6187
2a5v_A_ZN_405_ZN_6169
2a5v_A_ZN_409_ZN_6170
2a5v_B_ZN_408_ZN_6176
2a5v_D_ZN_406_ZN_6185
2ab4_A_ZN_310_ZN_2841
2ac2_A_ZN_500_ZN_2487
2adw_E_ZN_401_ZN_1118
2aer_H_ZN_3011_ZN_4700
2aer_H_ZN_3012_ZN_4701
2agz_D_ZN_301_ZN_7355
2agz_H_ZN_300_ZN_7369
2ahj_A_ZN_303_ZN_6364
2ahj_C_ZN_303_ZN_6383
2aho_A_ZN_914_ZN_5181
2ai1_A_ZN_291_ZN_2113
2ai2_A_ZN_291_ZN_2119
2ai3_A_ZN_291_ZN_2141
2ajy_H_ZN_304_ZN_3393
2ajy_H_ZN_305_ZN_3394
2ajy_L_ZN_301_ZN_3376
2ajy_L_ZN_302_ZN_3377

2ajy_L_ZN_303_ZN_3378
2ak1_H_ZN_502_ZN_3378
2ak1_H_ZN_504_ZN_3379
2ak1_L_ZN_501_ZN_3376
2ak1_L_ZN_503_ZN_3377
2akf_A_ZN_1_ZN_802,2akf_A_ZN_1_ZN_803
2akf_B_ZN_2_ZN_804,2akf_B_ZN_3_ZN_805,2akf_B_ZN_3_ZN_806,2akf_B_ZN_4_ZN_8
07,2akf_B_ZN_4_ZN_808
2al4_A_ZN_821_ZN_11757
2al4_F_ZN_830_ZN_11903
2al4_B_ZN_822_ZN_11771
2al4_B_ZN_824_ZN_11772
2al4_C_ZN_823_ZN_11786
2al4_C_ZN_825_ZN_11787
2al4_D_ZN_826_ZN_11837
2al4_E_ZN_827_ZN_11851
2al4_E_ZN_829_ZN_11852
2al4_F_ZN_828_ZN_11902
2alu_A_ZN_302_ZN_2776
2alu_A_ZN_303_ZN_2777
2as9_A_ZN_1777_ZN_3047
2as9_A_ZN_333_ZN_3044
2as9_A_ZN_666_ZN_3045
2as9_A_ZN_888_ZN_3046
2as9_B_ZN_222_ZN_3048
2as9_B_ZN_444_ZN_3049
2as9_B_ZN_555_ZN_3050
2axr_A_ZN_500_ZN_3786
2axr_A_ZN_501_ZN_3787
2axr_A_ZN_502_ZN_3788
2axr_A_ZN_503_ZN_3789
2ayi_A_ZN_500_ZN_15471,2ayi_A_ZN_501_ZN_15472
2ayi_B_ZN_600_ZN_15473,2ayi_B_ZN_601_ZN_15474
2ayi_C_ZN_700_ZN_15475,2ayi_C_ZN_701_ZN_15476
2ayi_D_ZN_800_ZN_15477,2ayi_D_ZN_801_ZN_15478
2ayi_E_ZN_900_ZN_15479,2ayi_E_ZN_901_ZN_15480
2ays_A_ZN_1001_ZN_2735
2ays_A_ZN_1002_ZN_2736
2azh_A_ZN_150_ZN_2244
2b0z_A_ZNH_295_ZN_3217
2b10_A_ZNH_295_ZN_6429
2b10_C_ZNH_795_ZN_6515
2b11_A_ZNH_1001_ZN_6445
2b11_B_ZNH_1101_ZN_6488
2b12_A_ZNH_295_ZN_3221
2b3x_A_ZN_1001_ZN_6930
2b63_A_ZN_2456_ZN_31736
2b65_A_ZN_1001_ZN_2795
2b65_A_ZN_1002_ZN_2796
2b65_A_ZN_1003_ZN_2797
2b8k_I_ZN_124_ZN_31050
2b8o_H_ZN_512_ZN_4720
2b8o_H_ZN_513_ZN_4721
2bcn_A_ZNH_295_ZN_5593
2bcn_C_ZNH_295_ZN_5679
2bf9_A_ZN_37_ZN_588
2bh3_A_ZN_1001_ZN_3526,2bh3_A_ZN_1002_ZN_3527
2bh3_A_ZN_1007_ZN_3542

2bh3_A_ZN_1008_ZN_3543
2bhb_A_ZN_1001_ZN_3510,2bhb_A_ZN_1002_ZN_3511
2bhb_A_ZN_1006_ZN_3526
2bhb_A_ZN_1007_ZN_3527
2bn7_A_ZN_1445_ZN_3540
2bo0_A_ZN_502_ZN_5123
2bo0_A_ZN_504_ZN_5124
2bo0_A_ZN_505_ZN_5125
2boq_A_ZN_1323_ZN_2449
2boq_A_ZN_1324_ZN_2450
2boq_A_ZN_1325_ZN_2451,2boq_A_ZN_1326_ZN_2452,2boq_A_ZN_1327_ZN_2453
2boq_A_ZN_1358_ZN_2462
2boq_A_ZN_1359_ZN_2463
2boq_A_ZN_1360_ZN_2464
2bp0_A_ZN_1339_ZN_5091
2bp0_B_ZN_1342_ZN_5112
2bp0_B_ZN_1343_ZN_5113
2bp0_A_ZN_1340_ZN_5092
2bp0_A_ZN_1341_ZN_5093
2bp0_A_ZN_1342_ZN_5094
2bp0_A_ZN_1343_ZN_5095
2bp0_A_ZN_1344_ZN_5096
2bp0_B_ZN_1339_ZN_5109
2bp0_B_ZN_1340_ZN_5110
2bp0_B_ZN_1341_ZN_5111
2bp8_A_ZN_1339_ZN_5124
2bp8_B_ZN_1343_ZN_5145
2bp8_A_ZN_1340_ZN_5125
2bp8_A_ZN_1341_ZN_5126
2bp8_A_ZN_1342_ZN_5127
2bp8_A_ZN_1343_ZN_5128
2bp8_B_ZN_1339_ZN_5141
2bp8_B_ZN_1340_ZN_5142
2bp8_B_ZN_1341_ZN_5143
2bp8_B_ZN_1342_ZN_5144
2bq8_X_ZN_1307_ZN_2433
2bsf_A_ZN_1330_ZN_1343
2byo_A_ZN_1208_ZN_1348
2byo_A_ZN_1209_ZN_1349
2byo_A_ZN_1210_ZN_1350
2byo_A_ZN_1211_ZN_1351
2byo_A_ZN_1212_ZN_1352
2byo_A_ZN_1213_ZN_1353
2c1d_A_ZN_1293_ZN_12294
2c1d_G_ZN_1293_ZN_12690
2c1d_G_ZN_1294_ZN_12691
2c1d_G_ZN_1295_ZN_12692
2c1d_A_ZN_1294_ZN_12295
2c1d_A_ZN_1295_ZN_12296
2c1d_A_ZN_1296_ZN_12297
2c1d_C_ZN_1293_ZN_12427
2c1d_C_ZN_1294_ZN_12428
2c1d_C_ZN_1295_ZN_12429
2c1d_E_ZN_1293_ZN_12559
2c1d_E_ZN_1294_ZN_12560
2c1g_A_ZN_1465_ZN_3104
2c1g_A_ZN_1466_ZN_3105
2c1g_A_ZN_1467_ZN_3106

2c1i_A_ZN_1465_ZN_3099
2c1i_A_ZN_1466_ZN_3100
2c20_A_ZN_601_ZN_15621
2c20_B_ZN_601_ZN_15666
2c20_C_ZN_601_ZN_15711
2c20_D_ZN_601_ZN_15756
2c20_E_ZN_601_ZN_15801
2c20_F_ZN_601_ZN_15846
2c36_A_ZN_1311_ZN_4336
2c3a_A_ZN_1310_ZN_4099
2c3a_B_ZN_1313_ZN_4131
2c3a_B_ZN_1314_ZN_4132
2c3a_B_ZN_1315_ZN_4133
2c3a_B_ZN_1316_ZN_4134
2c5w_B_ZN_701_ZN_3173
2c5w_B_ZN_702_ZN_3174
2c5w_B_ZN_703_ZN_3175
2c5w_B_ZN_704_ZN_3176
2c5w_B_ZN_705_ZN_3177
2c6w_B_ZN_701_ZN_3128
2c6w_B_ZN_702_ZN_3129
2c6w_B_ZN_703_ZN_3130
2c9s_A_ZN_1155_ZN_2414
2c9s_A_ZN_1157_ZN_2416
2c9s_F_ZN_1157_ZN_2455
2c9s_F_ZN_1160_ZN_2461
2c9u_A_ZN_1159_ZN_2288
2c9u_F_ZN_1155_ZN_2294
2cb8_A_ZN_1089_ZN_1505
2cb8_A_ZN_1090_ZN_1506,2cb8_A_ZN_1093_ZN_1509,2cb8_A_ZN_1096_ZN_1542
2cb8_A_ZN_1091_ZN_1507
2cb8_A_ZN_1092_ZN_1508
2cb8_B_ZN_1090_ZN_1625
2cb8_B_ZN_1091_ZN_1626
2cbi_B_ZN_1625_ZN_9261
2cbi_B_ZN_1636_ZN_9282
2cbi_B_ZN_1637_ZN_9283
2cbi_B_ZN_1626_ZN_9262
2cbi_B_ZN_1627_ZN_9263
2cbi_B_ZN_1628_ZN_9264
2cbi_B_ZN_1631_ZN_9277
2cbi_B_ZN_1632_ZN_9278
2cbi_B_ZN_1633_ZN_9279
2cbi_B_ZN_1634_ZN_9280
2cbi_B_ZN_1635_ZN_9281
2cc0_A_ZN_200_ZN_3019
2cc0_B_ZN_200_ZN_3024
2ccv_A_ZN_1100_ZN_821
2cei_A_ZN_210_ZN_1415
2cei_A_ZN_211_ZN_1416
2cei_A_ZN_212_ZN_1417,2cei_A_ZN_213_ZN_1418
2cei_A_ZN_214_ZN_1419
2cei_A_ZN_215_ZN_1420
2cei_A_ZN_216_ZN_1421,2cei_A_ZN_220_ZN_1422
2cex_A_ZN_1306_ZN_9529
2cex_B_ZN_1310_ZN_9562
2cex_B_ZN_1311_ZN_9563
2cex_C_ZN_1306_ZN_9564

2cex_C_ZN_1307_ZN_9565
2cex_D_ZN_1307_ZN_9572
2cey_A_ZN_1307_ZN_2382
2cey_A_ZN_1308_ZN_2383
2cey_A_ZN_1309_ZN_2384
2ch9_A_ZN_1155_ZN_1095
2ch9_A_ZN_1156_ZN_1096
2ch9_A_ZN_1157_ZN_1097
2ch9_A_ZN_1158_ZN_1098
2ch9_A_ZN_1159_ZN_1099
2chi_A_ZN_212_ZN_1416
2chi_A_ZN_214_ZN_1417
2chu_A_ZN_1311_ZN_4347
2chu_B_ZN_1315_ZN_4434
2cih_A_ZN_210_ZN_1422
2cih_A_ZN_211_ZN_1423
2cih_A_ZN_212_ZN_1424,2cih_A_ZN_213_ZN_1425
2cih_A_ZN_214_ZN_1426
2cih_A_ZN_215_ZN_1427
2cih_A_ZN_216_ZN_1428,2cih_A_ZN_220_ZN_1430,2cih_A_ZN_220_ZN_1431
2cih_A_ZN_217_ZN_1429
2cjl_A_ZN_1216_ZN_3103
2cjl_A_ZN_1217_ZN_3104
2cjl_B_ZN_1216_ZN_3105
2cjl_B_ZN_1217_ZN_3106
2ckr_A_ZN_1433_ZN_5076
2ckr_A_ZN_1434_ZN_5077
2ckr_B_ZN_1437_ZN_5152
2ckr_B_ZN_1438_ZN_5153
2ckr_B_ZN_1439_ZN_5154
2cks_A_ZN_1432_ZN_5160
2cks_A_ZN_1433_ZN_5161
2cks_A_ZN_1434_ZN_5162
2cks_A_ZN_1435_ZN_5163
2cks_B_ZN_1432_ZN_5184
2cks_B_ZN_1433_ZN_5185
2cks_B_ZN_1434_ZN_5186
2cks_B_ZN_1435_ZN_5187
2clb_A_ZN_1175_ZN_10972
2clb_B_ZN_1175_ZN_10974
2clb_C_ZN_1175_ZN_10976
2clb_D_ZN_1175_ZN_10978
2clb_M_ZN_1175_ZN_10980
2clb_N_ZN_1175_ZN_10982
2clb_O_ZN_1175_ZN_10984
2clb_P_ZN_1175_ZN_10986
2clu_A_ZN_212_ZN_1416
2cn6_A_ZN_210_ZN_1414
2cn6_A_ZN_212_ZN_1416,2cn6_A_ZN_213_ZN_1417
2cn6_A_ZN_214_ZN_1418
2cn6_A_ZN_215_ZN_1419
2cn6_A_ZN_216_ZN_1420,2cn6_A_ZN_220_ZN_1421
2cua_A_ZN_171_ZN_1981
2cua_B_ZN_172_ZN_1984
2cye_A_ZN_1001_ZN_4147
2cye_B_ZN_1002_ZN_4148
2cye_C_ZN_1003_ZN_4245
2cye_D_ZN_1004_ZN_4342

2d0w_A_ZN_1205_ZN_2585
2d0w_B_ZN_1219_ZN_2637
2d0w_A_ZN_1206_ZN_2586
2d0w_A_ZN_1214_ZN_2587
2d0w_A_ZN_1220_ZN_2588
2d0w_A_ZN_1222_ZN_2589
2d0w_B_ZN_1203_ZN_2633
2d0w_B_ZN_1207_ZN_2634
2d0w_B_ZN_1211_ZN_2635
2d0w_B_ZN_1218_ZN_2636
2d1n_A_ZN_285_ZN_2652
2d1n_A_ZN_286_ZN_2653
2d3k_B_ZN_1001_ZN_1879
2d3k_B_ZN_1002_ZN_1880
2d5m_A_ZN_201_ZN_1441
2d5m_A_ZN_202_ZN_1442
2d5m_A_ZN_203_ZN_1443
2d8a_A_ZN_502_ZN_2543
2d8a_A_ZN_503_ZN_2544
2d8a_A_ZN_504_ZN_2545
2d8a_A_ZN_505_ZN_2546
2dge_A_ZN_1001_ZN_3191
2dge_B_ZN_1002_ZN_3235
2dh3_A_ZN_601_ZN_6507
2di3_A_ZN_1002_ZN_3525
2di3_B_ZN_1001_ZN_3526
2djw_B_ZN_2003_ZN_6230
2djw_E_ZN_2002_ZN_6231
2djw_G_ZN_2004_ZN_6232
2djw_J_ZN_2001_ZN_6233
2dkc_A_ZN_901_ZN_8388
2dkc_B_ZN_902_ZN_8413
2dkd_A_ZN_921_ZN_8383
2dkd_B_ZN_922_ZN_8408
2doj_A_ZN_787_ZN_2751
2doj_A_ZN_788_ZN_2752
2dp8_A_ZN_692_ZN_2814
2dp8_A_ZN_693_ZN_2815
2dqv_A_ZN_3016_ZN_2762
2dqv_A_ZN_3017_ZN_2763
2ds9_A_ZN_1101_ZN_2744
2ds9_A_ZN_1102_ZN_2745
2dsf_A_ZN_1101_ZN_2719
2dsf_A_ZN_1102_ZN_2720
2dt8_A_ZN_501_ZN_2170
2dvc_A_ZN_1004_ZN_2796
2dvc_A_ZN_1005_ZN_2797
2dwa_A_ZN_1003_ZN_2832
2dwa_A_ZN_1004_ZN_2833
2dwh_A_ZN_3_ZN_2728
2dwh_A_ZN_4_ZN_2729
2dwi_A_ZN_1003_ZN_2838
2dwi_A_ZN_1004_ZN_2839
2dwj_A_ZN_1101_ZN_2744
2dwj_A_ZN_1102_ZN_2745
2dxr_A_ZN_1005_ZN_2797
2dxr_A_ZN_1006_ZN_2798
2dxy_A_ZN_1101_ZN_2720

2dxy_A_ZN_1102_ZN_2721
2dyx_A_ZN_1004_ZN_2815
2dyx_A_ZN_1005_ZN_2816
2e0p_A_ZN_700_ZN_4087
2e0s_A_ZN_1004_ZN_2812
2e0s_A_ZN_1005_ZN_2813
2e18_A_ZN_1303_ZN_4053
2e18_A_ZN_1304_ZN_4054
2e18_A_ZN_1305_ZN_4055
2e18_A_ZN_1306_ZN_4056
2e18_B_ZN_1307_ZN_4072
2e18_B_ZN_1308_ZN_4073
2e1s_A_ZN_1004_ZN_2801
2e1s_A_ZN_1005_ZN_2802
2e26_A_ZN_603_ZN_5678
2e26_A_ZN_606_ZN_5681
2e2h_A_ZN_1734_ZN_28975
2e2i_A_ZN_1734_ZN_29665
2e4t_A_ZN_700_ZN_4099
2e6l_A_ZN_501_ZN_1472,2e6l_A_ZN_502_ZN_1473
2e84_A_ZN_557_ZN_3815
2e84_A_ZN_558_ZN_3816
2e84_A_ZN_559_ZN_3817
2e84_A_ZN_560_ZN_3818
2e88_A_ZN_501_ZN_2957
2ebn_A_ZN_301_ZN_2215
2eex_A_ZN_700_ZN_4070
2eh1_A_ZN_102_ZN_1336
2eh1_B_ZN_101_ZN_1345
2eh9_A_ZN_201_ZN_886,2eh9_A_ZN_202_ZN_887
2eh9_A_ZN_203_ZN_888
2eh9_A_ZN_204_ZN_889
2ehs_A_ZN_202_ZN_601
2ehs_A_ZN_203_ZN_602
2ehs_A_ZN_204_ZN_603
2ehs_A_ZN_205_ZN_604
2ehs_A_ZN_206_ZN_605
2ehs_A_ZN_207_ZN_606
2ehs_A_ZN_208_ZN_607
2eht_A_ZN_201_ZN_605
2eht_A_ZN_202_ZN_606
2eht_A_ZN_203_ZN_607
2eht_A_ZN_204_ZN_608
2eht_A_ZN_205_ZN_609,2eht_A_ZN_207_ZN_611
2eht_A_ZN_206_ZN_610
2eij_C_ZN_262_ZN_28949
2eij_P_ZN_1262_ZN_30176
2eim_A_ZN_603_ZN_28536
2eim_C_ZN_262_ZN_28916
2eim_N_ZN_1603_ZN_29650
2eim_P_ZN_1262_ZN_30127
2ein_B_ZN_607_ZN_28884
2ein_P_ZN_1262_ZN_30121
2ein_P_ZN_1604_ZN_30122
2ein_T_ZN_1608_ZN_30437
2ein_C_ZN_262_ZN_29001
2ein_D_ZN_605_ZN_29368
2ein_F_ZN_604_ZN_29370

2ein_G_ZN_608_ZN_29371
2ein_L_ZN_603_ZN_29583
2ein_N_ZN_1603_ZN_29683,2ein_N_ZN_1609_ZN_29684
2ein_O_ZN_1607_ZN_29970
2ej1_A_ZN_700_ZN_4202
2ejc_A_ZN_501_ZN_2302
2ejc_A_ZN_511_ZN_2312,2ejc_A_ZN_512_ZN_2313
2ejc_A_ZN_513_ZN_2314
2ejc_A_ZN_514_ZN_2315
2ejc_A_ZN_515_ZN_2316,2ejc_A_ZN_516_ZN_2317
2ejc_A_ZN_520_ZN_2321
2ejc_A_ZN_522_ZN_2323
2ejc_A_ZN_502_ZN_2303
2ejc_A_ZN_503_ZN_2304,2ejc_A_ZN_519_ZN_2320
2ejc_A_ZN_504_ZN_2305
2ejc_A_ZN_505_ZN_2306
2ejc_A_ZN_506_ZN_2307
2ejc_A_ZN_507_ZN_2308
2ejc_A_ZN_508_ZN_2309,2ejc_A_ZN_521_ZN_2322
2ejc_A_ZN_509_ZN_2310,2ejc_A_ZN_510_ZN_2311,2ejc_A_ZN_517_ZN_2318,2ejc_A_ZN_518_ZN_2319
2ek0_A_ZN_102_ZN_1351
2ek0_B_ZN_101_ZN_1352
2ek0_B_ZN_103_ZN_1353
2ek8_A_ZN_1003_ZN_3123,2ek8_A_ZN_1004_ZN_3124
2ek8_A_ZN_1005_ZN_3125
2ek8_A_ZN_1006_ZN_3126
2ek9_A_ZN_1003_ZN_3123,2ek9_A_ZN_1004_ZN_3124
2ek9_A_ZN_1005_ZN_3125
2ek9_A_ZN_1006_ZN_3126
2eo7_A_ZN_700_ZN_4132
2epf_A_ZN_301_ZN_6501
2epf_A_ZN_305_ZN_6502
2epf_B_ZN_302_ZN_6504
2epf_C_ZN_303_ZN_6505
2epf_D_ZN_304_ZN_6507
2eqd_A_ZN_700_ZN_4108
2esl_A_ZN_4_ZN_8840
2esl_D_ZN_5_ZN_8857
2eul_A_ZN_401_ZN_4813
2eul_C_ZN_407_ZN_4826
2eul_C_ZN_408_ZN_4827,2eul_C_ZN_423_ZN_4834
2eul_C_ZN_409_ZN_4828
2eul_C_ZN_411_ZN_4830,2eul_C_ZN_415_ZN_4832,2eul_C_ZN_421_ZN_4833,2eul_D_ZN_422_ZN_4837
2eul_C_ZN_412_ZN_4831
2eul_C_ZN_425_ZN_4835
2eul_D_ZN_418_ZN_4836
2eul_A_ZN_402_ZN_4814,2eul_A_ZN_413_ZN_4817,2eul_A_ZN_416_ZN_4818,2eul_A_ZN_417_ZN_4819,2eul_B_ZN_405_ZN_4822,2eul_B_ZN_414_ZN_4823
2eul_A_ZN_403_ZN_4815
2eul_A_ZN_404_ZN_4816
2eul_A_ZN_419_ZN_4820
2eul_A_ZN_424_ZN_4821
2eul_B_ZN_420_ZN_4824
2eul_C_ZN_400_ZN_4829
2eul_C_ZN_406_ZN_4825
2ev6_A_ZN_1151_ZN_2273

2ev6_B_ZN_2151_ZN_2286
2f3b_A_ZN_340_ZN_2516,2f3b_A_ZN_341_ZN_2517,2f3b_A_ZN_342_ZN_2518
2f3d_A_ZN_340_ZN_2526,2f3d_A_ZN_341_ZN_2527,2f3d_A_ZN_342_ZN_2528
2f4l_A_ZN_1400_ZN_8378,2f4l_A_ZN_1401_ZN_8379
2f4l_B_ZN_2400_ZN_8380,2f4l_B_ZN_2401_ZN_8381
2f4l_C_ZN_3400_ZN_8383,2f4l_C_ZN_3401_ZN_8384
2f4l_D_ZN_4400_ZN_8385,2f4l_D_ZN_4401_ZN_8386
2f4o_A_ZN_502_ZN_2950
2f4y_C_ZN_1001_ZN_2584
2f56_C_ZN_300_ZN_2620
2f5w_C_ZN_150_ZN_2596
2f6s_A_ZN_1101_ZN_2933
2f6s_B_ZN_5501_ZN_2939
2f92_F_ZN_1001_ZN_2776,2f92_F_ZN_1002_ZN_2777,2f92_F_ZN_1003_ZN_2778
2f94_F_ZN_1001_ZN_2767,2f94_F_ZN_1002_ZN_2768,2f94_F_ZN_1003_ZN_2769
2f9k_F_ZN_1001_ZN_2776,2f9k_F_ZN_1002_ZN_2777,2f9k_F_ZN_1003_ZN_2778
2fa7_A_ZN_302_ZN_2842
2fa7_A_ZN_303_ZN_2843
2fa7_A_ZN_304_ZN_2844
2fac_A_ZN_401_ZN_1197,2fac_B_ZN_410_ZN_1234
2fac_B_ZN_411_ZN_1235
2fac_A_ZN_402_ZN_1198
2fac_A_ZN_403_ZN_1199
2fac_A_ZN_404_ZN_1200
2fac_A_ZN_405_ZN_1201
2fac_A_ZN_406_ZN_1202
2fac_A_ZN_408_ZN_1203
2fac_B_ZN_407_ZN_1232
2fac_B_ZN_409_ZN_1233
2fad_A_ZN_402_ZN_1198
2fad_A_ZN_403_ZN_1199
2fad_A_ZN_404_ZN_1200
2fad_A_ZN_405_ZN_1201
2fad_A_ZN_406_ZN_1202
2fad_A_ZN_407_ZN_1203
2fad_A_ZN_408_ZN_1204
2fad_B_ZN_409_ZN_1234
2fae_A_ZN_401_ZN_1197
2fae_A_ZN_402_ZN_1198
2fae_A_ZN_403_ZN_1199
2fae_A_ZN_404_ZN_1200
2fae_A_ZN_405_ZN_1201
2fae_A_ZN_406_ZN_1202
2fae_A_ZN_408_ZN_1203
2fae_B_ZN_407_ZN_1236
2fbh_A_ZN_203_ZN_1142
2fbh_A_ZN_204_ZN_1143
2fbj_H_ZN_226_ZN_3388
2fir_H_ZN_1011_ZN_4726
2fir_H_ZN_1012_ZN_4727
2fj9_A_ZN_88_ZN_711
2fkf_A_ZN_500_ZN_3409
2fkm_X_ZN_500_ZN_3405
2fm6_A_ZN_403_ZN_4012
2fs5_A_ZN_701_ZN_3393
2fz6_A_ZN_201_ZN_1977
2fz6_A_ZN_202_ZN_1978
2fz6_B_ZN_203_ZN_1979

2fz6_C_ZN_204_ZN_1980
2g2n_A_ZN_1001_ZN_3643,2g2n_A_ZN_1005_ZN_3644,2g2n_A_ZN_1009_ZN_3645
2g2n_C_ZN_1015_ZN_3660
2g2n_C_ZN_1019_ZN_3661
2g2n_D_ZN_1004_ZN_3667
2g2n_D_ZN_1008_ZN_3668
2g2n_D_ZN_1016_ZN_3669
2g2n_D_ZN_1020_ZN_3670
2g2n_A_ZN_1013_ZN_3646
2g2n_A_ZN_1017_ZN_3647
2g2n_B_ZN_1002_ZN_3648
2g2n_B_ZN_1006_ZN_3649,2g2n_B_ZN_1010_ZN_3650
2g2n_B_ZN_1014_ZN_3651
2g2n_B_ZN_1018_ZN_3652
2g2n_C_ZN_1003_ZN_3658
2g2n_C_ZN_1007_ZN_3659
2g2p_A_ZN_1001_ZN_3653,2g2p_A_ZN_1005_ZN_3654,2g2p_A_ZN_1009_ZN_3655
2g2p_D_ZN_1008_ZN_3688,2g2p_D_ZN_1012_ZN_3689
2g2p_D_ZN_1016_ZN_3690
2g2p_A_ZN_1013_ZN_3656
2g2p_B_ZN_1002_ZN_3668
2g2p_B_ZN_1006_ZN_3669,2g2p_B_ZN_1010_ZN_3670
2g2p_B_ZN_1014_ZN_3671
2g2p_C_ZN_1003_ZN_3678
2g2p_C_ZN_1007_ZN_3679
2g2p_C_ZN_1015_ZN_3680
2g2p_D_ZN_1004_ZN_3687
2g7z_A_ZN_6001_ZN_4167
2g7z_A_ZN_6012_ZN_4176
2g7z_A_ZN_6013_ZN_4177
2g7z_B_ZN_6009_ZN_4212
2g7z_B_ZN_6010_ZN_4213
2g7z_B_ZN_6014_ZN_4214
2g7z_B_ZN_6015_ZN_4215
2g7z_B_ZN_6016_ZN_4216
2g7z_A_ZN_6002_ZN_4168
2g7z_A_ZN_6003_ZN_4169
2g7z_A_ZN_6004_ZN_4170
2g7z_A_ZN_6005_ZN_4171
2g7z_A_ZN_6006_ZN_4172
2g7z_A_ZN_6007_ZN_4173
2g7z_A_ZN_6008_ZN_4174
2g7z_A_ZN_6011_ZN_4175
2g87_A_ZN_2011_ZN_5574
2g87_A_ZN_957_ZN_5571
2g87_A_ZN_959_ZN_5572
2g87_A_ZN_962_ZN_5573
2g87_B_ZN_956_ZN_5745
2g87_B_ZN_958_ZN_5746
2g87_B_ZN_963_ZN_5747
2g93_A_ZN_690_ZN_2785
2g93_A_ZN_691_ZN_2786
2gbx_B_ZN_460_ZN_15055
2gbx_B_ZN_464_ZN_15056
2gbx_B_ZN_468_ZN_15057
2gbx_D_ZN_462_ZN_15075
2gbx_D_ZN_465_ZN_15076
2gbx_D_ZN_469_ZN_15077

2gbx_F_ZN_463_ZN_15095
2gbx_F_ZN_466_ZN_15096
2gbx_F_ZN_467_ZN_15097
2gc1_A_ZN_403_ZN_3047
2gfe_A_ZN_869_ZN_6212
2gfe_B_ZN_870_ZN_6223
2gfe_B_ZN_872_ZN_6224
2gfe_C_ZN_871_ZN_6235
2gfe_C_ZN_873_ZN_6236
2gjz_A_ZN_213_ZN_6680
2gjz_L_ZN_216_ZN_6676
2gjz_L_ZN_217_ZN_6677
2gjz_B_ZN_229_ZN_6681
2gjz_B_ZN_230_ZN_6682
2gjz_B_ZN_231_ZN_6683
2gjz_H_ZN_229_ZN_6678
2gjz_H_ZN_230_ZN_6679
2gjz_L_ZN_213_ZN_6673
2gjz_L_ZN_214_ZN_6674
2gjz_L_ZN_215_ZN_6675
2gmn_A_ZN_805_ZN_4043
2gmn_B_ZN_806_ZN_4046
2go3_A_ZN_604_ZN_4346
2go3_B_ZN_603_ZN_4399
2go3_B_ZN_605_ZN_4400
2go4_A_ZN_604_ZN_4302
2go4_A_ZN_605_ZN_4303
2go4_B_ZN_603_ZN_4336
2gpy_A_ZN_304_ZN_3179
2gpy_A_ZN_306_ZN_3180
2gpy_A_ZN_307_ZN_3181
2gpy_A_ZN_308_ZN_3182
2gpy_B_ZN_302_ZN_3184
2gpy_B_ZN_303_ZN_3185
2gpy_B_ZN_305_ZN_3186
2gpy_B_ZN_309_ZN_3187
2gpy_B_ZN_310_ZN_3188
2gtl_M_ZN_252_ZN_19139
2gu1_A_ZN_362_ZN_2555
2gvi_A_ZN_302_ZN_1603
2gvi_A_ZN_303_ZN_1604
2gvi_A_ZN_304_ZN_1605
2gvm_A_ZN_201_ZN_1973
2gvm_B_ZN_202_ZN_2006
2gyq_A_ZN_407_ZN_2774
2gyq_A_ZN_409_ZN_2775
2gyq_A_ZN_410_ZN_2776,2gyq_A_ZN_418_ZN_2777
2gyq_B_ZN_406_ZN_2789
2gyq_B_ZN_408_ZN_2790
2h1l_D_ZN_3_ZN_53923
2h2g_A_ZN_1002_ZN_1906
2h32_A_ZN_302_ZN_3431
2h32_H_ZN_301_ZN_3432
2h4i_A_ZN_697_ZN_2840
2h4i_A_ZN_698_ZN_2841
2h4l_X_ZN_500_ZN_3456
2h4n_A_ZN_263_ZN_2049
2h5a_X_ZN_700_ZN_3495

2h6s_A_ZN_602_ZN_2577
2h6t_A_ZN_602_ZN_2577
2hae_D_ZN_800_ZN_11577
2hap_D_ZN_136_ZN_2076
2hba_A_ZN_101_ZN_814
2hba_A_ZN_102_ZN_815
2hba_A_ZN_106_ZN_816
2hba_B_ZN_103_ZN_821
2hba_B_ZN_104_ZN_822
2hba_B_ZN_105_ZN_823
2hba_B_ZN_107_ZN_824
2hba_B_ZN_108_ZN_825
2hbb_A_ZN_101_ZN_392
2hbb_A_ZN_102_ZN_393
2hbb_A_ZN_103_ZN_394
2hbb_A_ZN_104_ZN_395
2hbl_A_ZN_1001_ZN_3218
2hbm_A_ZN_1001_ZN_3218
2hca_A_ZN_698_ZN_2784
2hca_A_ZN_699_ZN_2785
2hcm_A_ZN_367_ZN_1231,2hcm_A_ZN_368_ZN_1232
2hf8_A_ZN_401_ZN_3250,2hf8_B_ZN_402_ZN_3285
2hf8_A_ZN_403_ZN_3251
2hf8_B_ZN_404_ZN_3286
2hf9_A_ZN_302_ZN_3250
2hf9_B_ZN_302_ZN_3284
2hh5_A_ZN_701_ZN_3423
2hh5_A_ZN_702_ZN_3424
2hh5_A_ZN_703_ZN_3425
2hh5_B_ZN_601_ZN_3375
2hh5_B_ZN_602_ZN_3376
2hh5_B_ZN_603_ZN_3377
2hi7_A_ZN_190_ZN_2559
2hjg_A_ZN_900_ZN_3149
2hpy_A_ZN_2011_ZN_5574
2hpy_A_ZN_957_ZN_5571
2hpy_A_ZN_959_ZN_5572
2hpy_A_ZN_962_ZN_5573
2hpy_B_ZN_956_ZN_5764
2hpy_B_ZN_958_ZN_5765
2hpy_B_ZN_963_ZN_5766
2hqk_A_ZN_401_ZN_1812
2hqk_A_ZN_402_ZN_1813
2hqk_A_ZN_403_ZN_1814
2hue_A_ZN_403_ZN_2694
2hvf_A_ZN_101_ZN_411
2hvf_A_ZN_102_ZN_412
2hvf_A_ZN_103_ZN_413
2hvf_A_ZN_104_ZN_414
2hz8_A_ZN_116_ZN_1932,2hz8_A_ZN_117_ZN_1933
2hzc_A_ZN_302_ZN_715
2hzc_A_ZN_303_ZN_716
2i0m_A_ZN_217_ZN_1620
2i0m_A_ZN_218_ZN_1621
2i0m_A_ZN_219_ZN_1622
2i0o_A_ZN_579_ZN_2288,2i0o_A_ZN_580_ZN_2289,2i0o_A_ZN_581_ZN_2290
2i0o_A_ZN_582_ZN_2291
2i0o_A_ZN_583_ZN_2292

2i0o_A_ZN_584_ZN_2293
2i14_E_ZN_2393_ZN_18434
2i14_F_ZN_2793_ZN_18458
2i14_A_ZN_393_ZN_18338
2i14_B_ZN_793_ZN_18362
2i14_C_ZN_1393_ZN_18386
2i14_D_ZN_1793_ZN_18410
2i2x_M_ZN_523_ZN_44453
2i2x_A_ZN_524_ZN_43898
2i2x_E_ZN_522_ZN_44083
2i2x_I_ZN_521_ZN_44268
2i3v_A_ZN_301_ZN_8105
2i3v_A_ZN_302_ZN_8106
2i3v_B_ZN_303_ZN_8117
2i3v_B_ZN_304_ZN_8118
2i3v_B_ZN_305_ZN_8119
2i3v_C_ZN_306_ZN_8130
2i3v_D_ZN_307_ZN_8141
2i7v_A_ZN_483_ZN_3475
2i7v_A_ZN_484_ZN_3476
2ier_A_ZN_603_ZN_4318
2ier_A_ZN_605_ZN_4319
2ier_A_ZN_608_ZN_4321
2ier_B_ZN_604_ZN_4372
2ier_B_ZN_609_ZN_4374
2ies_A_ZN_602_ZN_4335
2ies_A_ZN_603_ZN_4336
2ies_A_ZN_604_ZN_4346
2ies_A_ZN_605_ZN_4366
2ies_A_ZN_606_ZN_4347
2igi_A_ZN_1001_ZN_2919,2igi_A_ZN_1004_ZN_2922
2igi_B_ZN_1007_ZN_2945,2igi_B_ZN_1010_ZN_2948
2ims_A_ZN_200_ZN_1302
2imt_A_ZN_200_ZN_1303
2imz_A_ZN_501_ZN_2258
2imz_B_ZN_502_ZN_2259
2in4_A_HE5_201_ZN_1249,2in4_A_HE5_201_ZN_1250
2iu2_A_ZN_210_ZN_1413
2iu2_A_ZN_211_ZN_1414
2iu2_A_ZN_214_ZN_1415
2iu2_A_ZN_215_ZN_1416
2iu2_A_ZN_219_ZN_1417
2iu2_A_ZN_220_ZN_1418
2iv0_A_ZN_1413_ZN_6454
2iv0_A_ZN_1414_ZN_6455
2iv0_A_ZN_1415_ZN_6456
2iv0_A_ZN_1416_ZN_6457
2iv0_B_ZN_1413_ZN_6460
2iv0_B_ZN_1414_ZN_6461
2iv0_B_ZN_1415_ZN_6462
2iv0_B_ZN_1416_ZN_6463
2iv0_B_ZN_1417_ZN_6464
2iw0_A_ZN_1255_ZN_1768
2iwe_A_ZN_1129_ZN_3912
2iwe_D_ZN_1129_ZN_3913
2iwe_G_ZN_1129_ZN_3914
2iwe_J_ZN_1129_ZN_3915
2iyb_E_ZN_1424_ZN_5398

2izo_A_ZN_1347_ZN_5729
2izo_B_ZN_1246_ZN_5732
2izo_C_ZN_1251_ZN_5735
2izo_C_ZN_1252_ZN_5736
2j13_A_ZN_1236_ZN_1760
2j13_A_ZN_1237_ZN_1761
2j13_A_ZN_1238_ZN_1762
2j13_A_ZN_1239_ZN_1763
2j1m_A_ZN_1457_ZN_7423
2j1m_A_ZN_1458_ZN_7424
2j1m_A_ZN_1459_ZN_7425
2j1m_A_ZN_1460_ZN_7426
2j1m_A_ZN_1461_ZN_7427
2j1m_B_ZN_1457_ZN_7479
2j1m_B_ZN_1458_ZN_7480
2j44_A_ZN_1224_ZN_1803
2j44_A_ZN_1225_ZN_1804
2j44_A_ZN_1226_ZN_1805
2j4s_B_ZN_1457_ZN_7392
2j65_A_ZN_303_ZN_4342
2j65_A_ZN_304_ZN_4343
2j65_B_ZN_303_ZN_4388
2j6e_A_ZN_1446_ZN_9827
2j6e_B_ZN_1446_ZN_9975
2j6x_D_ZN_1376_ZN_22449
2ja5_A_ZN_2461_ZN_31670
2ja5_A_ZN_2464_ZN_31673
2ja6_A_ZN_2461_ZN_32022
2ja7_A_ZN_2464_ZN_63944
2ja7_A_ZN_2465_ZN_63945
2ja8_A_ZN_2461_ZN_32011
2jd8_0_ZN_201_ZN_49898,2jd8_0_ZN_202_ZN_49899
2jd8_9_ZN_201_ZN_49980,2jd8_9_ZN_202_ZN_49981
2jd8_A_ZN_201_ZN_49983,2jd8_A_ZN_202_ZN_49984
2jd8_B_ZN_201_ZN_49996,2jd8_B_ZN_202_ZN_49997
2jd8_C_ZN_201_ZN_50009,2jd8_C_ZN_202_ZN_50010
2jd8_D_ZN_201_ZN_50022,2jd8_D_ZN_202_ZN_50023
2jd8_E_ZN_201_ZN_50030,2jd8_E_ZN_202_ZN_50031
2jd8_F_ZN_201_ZN_50038,2jd8_F_ZN_202_ZN_50039
2jd8_G_ZN_201_ZN_50046,2jd8_G_ZN_202_ZN_50047
2jd8_H_ZN_201_ZN_50054,2jd8_H_ZN_202_ZN_50055
2jd8_I_ZN_201_ZN_50062,2jd8_I_ZN_202_ZN_50063
2jd8_1_ZN_201_ZN_49901,2jd8_1_ZN_202_ZN_49902
2jd8_J_ZN_201_ZN_50075,2jd8_J_ZN_202_ZN_50076
2jd8_K_ZN_201_ZN_50093,2jd8_K_ZN_202_ZN_50094
2jd8_L_ZN_201_ZN_50106,2jd8_L_ZN_202_ZN_50107
2jd8_M_ZN_201_ZN_50109,2jd8_M_ZN_202_ZN_50110
2jd8_N_ZN_201_ZN_50117,2jd8_N_ZN_202_ZN_50118
2jd8_O_ZN_201_ZN_50120,2jd8_O_ZN_202_ZN_50121
2jd8_P_ZN_201_ZN_50133,2jd8_P_ZN_202_ZN_50134
2jd8_Q_ZN_201_ZN_50136,2jd8_Q_ZN_202_ZN_50137
2jd8_R_ZN_201_ZN_50144,2jd8_R_ZN_202_ZN_50145
2jd8_S_ZN_201_ZN_50147,2jd8_S_ZN_202_ZN_50148
2jd8_2_ZN_201_ZN_49909,2jd8_2_ZN_202_ZN_49910
2jd8_T_ZN_201_ZN_50160,2jd8_T_ZN_202_ZN_50161
2jd8_U_ZN_201_ZN_50168,2jd8_U_ZN_202_ZN_50169
2jd8_V_ZN_201_ZN_50171,2jd8_V_ZN_202_ZN_50172
2jd8_W_ZN_201_ZN_50184,2jd8_W_ZN_202_ZN_50185

2jd8_X_ZN_201_ZN_50202,2jd8_X_ZN_202_ZN_50203
2jd8_Y_ZN_201_ZN_50210,2jd8_Y_ZN_202_ZN_50211
2jd8_Z_ZN_201_ZN_50218,2jd8_Z_ZN_202_ZN_50219
2jd8_3_ZN_201_ZN_49917,2jd8_3_ZN_202_ZN_49918
2jd8_4_ZN_201_ZN_49935,2jd8_4_ZN_202_ZN_49936
2jd8_5_ZN_201_ZN_49943,2jd8_5_ZN_202_ZN_49944
2jd8_6_ZN_201_ZN_49956,2jd8_6_ZN_202_ZN_49957
2jd8_7_ZN_201_ZN_49964,2jd8_7_ZN_202_ZN_49965
2jd8_8_ZN_201_ZN_49972,2jd8_8_ZN_202_ZN_49973
2jew_A_ZN_1308_ZN_2438
2jgo_A_ZN_1031_ZN_752,2jgo_A_ZN_1032_ZN_753
2jgo_B_ZN_1030_ZN_754,2jgo_B_ZN_1031_ZN_755
2jho_A_ZN_1157_ZN_1279
2jho_A_ZN_1158_ZN_1280
2jig_A_ZN_1252_ZN_3228
2jig_A_ZN_1253_ZN_3229
2jig_B_ZN_1252_ZN_3253
2jig_B_ZN_1253_ZN_3254
2jjl_A_ZN_1330_ZN_1559
2jjl_A_ZN_1331_ZN_1560
2jjl_A_ZN_1332_ZN_1561
2jjz_A_ZN_1126_ZN_3411
2jjz_B_ZN_1128_ZN_3416
2jjz_C_ZN_1128_ZN_3418
2jjz_D_ZN_1123_ZN_3425
2jks_A_ZN_1316_ZN_2140
2jks_A_ZN_1317_ZN_2141
2jks_A_ZN_1318_ZN_2142,2jks_A_ZN_1322_ZN_2146
2jks_A_ZN_1319_ZN_2143
2jks_A_ZN_1320_ZN_2144
2jks_A_ZN_1321_ZN_2145
2jks_A_ZN_1323_ZN_2147
2jl0_A_ZN_503_ZN_5155
2jl0_C_ZN_503_ZN_5183
2jl3_A_ZN_503_ZN_5039
2jl3_A_ZN_888_ZN_5040
2jl3_B_ZN_502_ZN_5067
2jl3_B_ZN_888_ZN_5081
2k78_A_ZNH_151_ZN_1986
2kfn_A_ZN_1_ZN_4820
2kfn_A_ZN_320_ZN_4822
2kfn_A_ZN_3_ZN_4821
2kfz_A_ZN_1_ZN_4819,2kfz_A_ZN_2_ZN_4820
2kfz_A_ZN_320_ZN_4822
2kfz_A_ZN_3_ZN_4821
2kik_A_ZN_50_ZN_1643,2kik_B_ZN_50_ZN_1644
2kkh_A_ZN_201_ZN_1553
2kmn_A_ZN_148_ZN_2355
2kn9_A_ZN_82_ZN_1198
2kzm_A_ZN_1_ZN_4799
2kzz_A_ZN_1_ZN_4799,2kzz_B_ZN_2_ZN_4798
2kzz_A_ZN_3_ZN_4800
2nl9_A_ZN_1001_ZN_1396
2nl9_A_ZN_1002_ZN_1397
2nl9_A_ZN_1003_ZN_1398,2nl9_A_ZN_1004_ZN_1399
2nl9_A_ZN_1005_ZN_1400,2nl9_A_ZN_1006_ZN_1401
2nl9_B_ZN_1007_ZN_1403
2nnx_A_ZN_156_ZN_4452

2nnx_B_ZN_156_ZN_4454
2nnx_C_ZN_156_ZN_4456
2nnx_D_ZN_156_ZN_4458
2nsf_A_ZN_404_ZN_1874
2nuv_A_ZN_1_ZN_2729
2nuv_A_ZN_3002_ZN_2730
2nvv_A_ZN_507_ZN_22975
2nvv_B_ZN_507_ZN_22976
2nvv_C_ZN_507_ZN_22977
2nvv_D_ZN_507_ZN_22978
2nvv_E_ZN_507_ZN_22979
2nvv_E_ZN_508_ZN_22980
2nvx_B_ZN_1307_ZN_29387
2nvz_A_ZN_1734_ZN_28977
2nwj_A_ZN_1101_ZN_2752
2nwj_A_ZN_1102_ZN_2753
2nxf_A_ZN_403_ZN_2575
2nxf_A_ZN_404_ZN_2576
2o1l_A_ZN_689_ZN_2753
2o1l_A_ZN_690_ZN_2754
2o1q_B_ZN_146_ZN_2290
2o3z_A_ZN_502_ZN_4345
2o3z_B_ZN_504_ZN_4370
2o3z_B_ZN_506_ZN_4371
2o4m_C_ZN_3015_ZN_10319
2o4m_C_ZN_3016_ZN_10320
2o4m_C_ZN_3017_ZN_10321
2o4m_C_ZN_3020_ZN_10322
2o4m_P_ZN_3011_ZN_10346
2o4m_P_ZN_3012_ZN_10347
2o4m_P_ZN_3021_ZN_10348
2o4m_P_ZN_3022_ZN_10349
2o4m_A_ZN_3009_ZN_10284
2o4m_A_ZN_3013_ZN_10285
2o4m_A_ZN_3018_ZN_10286
2o4m_A_ZN_3024_ZN_10287
2o4m_B_ZN_3010_ZN_10299
2o4m_B_ZN_3014_ZN_10300
2o4m_B_ZN_3019_ZN_10301
2o51_A_ZN_696_ZN_2752
2o51_A_ZN_697_ZN_2753
2o6d_A_ZN_302_ZN_2596
2o6d_B_ZN_301_ZN_2613
2o6e_A_ZN_301_ZN_2569
2o6e_A_ZN_304_ZN_2570
2o6e_A_ZN_305_ZN_2571
2o6e_A_ZN_306_ZN_2572
2o6e_A_ZN_307_ZN_2573
2o6e_A_ZN_308_ZN_2574
2o6e_B_ZN_302_ZN_2589
2o6e_B_ZN_303_ZN_2590
2o6p_A_ZN_1004_ZN_2047
2o6p_A_ZN_1006_ZN_2048
2o6p_B_ZN_1001_ZN_2093
2o6p_B_ZN_1002_ZN_2095
2o6p_B_ZN_1003_ZN_2094
2o6p_B_ZN_1005_ZN_2096
2oaj_A_ZN_963_ZN_6832

2ocu_A_ZN_1002_ZN_2710
2ocu_A_ZN_1003_ZN_2711
2oh3_A_ZN_300_ZN_1217
2okl_A_ZN_601_ZN_2889
2okl_B_ZN_602_ZN_2957
2ooi_A_ZN_1_ZN_2595
2ooi_B_ZN_2_ZN_2596
2ou3_A_ZN_161_ZN_2601
2ou3_A_ZN_162_ZN_2602,2ou3_A_ZN_162_ZN_2603
2ou3_B_ZN_161_ZN_2619
2ou3_B_ZN_162_ZN_2620
2ou3_B_ZN_163_ZN_2621
2ou7_A_ZN_501_ZN_2372
2owb_A_ZN_501_ZN_2389
2ox8_A_ZN_1_ZN_4317
2ox8_B_ZN_5_ZN_4329
2ox8_C_ZN_1_ZN_4333
2ox8_C_ZN_2_ZN_4334
2ox8_C_ZN_9_ZN_4341
2ox8_D_ZN_1_ZN_4342
2ox8_D_ZN_2_ZN_4343
2ox8_D_ZN_9_ZN_4350
2ox8_A_ZN_2_ZN_4318
2ox8_A_ZN_3_ZN_4319
2ox8_A_ZN_4_ZN_4320
2ox8_A_ZN_5_ZN_4321
2ox8_B_ZN_1_ZN_4325
2ox8_B_ZN_2_ZN_4326
2ox8_B_ZN_3_ZN_4327
2ox8_B_ZN_4_ZN_4328
2oz4_L_ZN_2001_ZN_5311
2oz4_L_ZN_2002_ZN_5312
2p05_A_ZN_200_ZN_540
2p09_A_ZN_200_ZN_589
2p0x_A_ZN_1319_ZN_1037
2p1h_A_ZN_101_ZN_755
2p1h_A_ZN_102_ZN_756,2p1h_A_ZN_105_ZN_759
2p1h_A_ZN_103_ZN_757,2p1h_A_ZN_104_ZN_758
2p1h_A_ZN_106_ZN_760
2p1s_A_ZN_1001_ZN_2733
2p1s_A_ZN_1002_ZN_2734
2p2l_A_ZN_201_ZN_4145
2p2l_A_ZN_202_ZN_4146
2p2l_A_ZN_901_ZN_4147
2p2l_A_ZN_902_ZN_4148
2p2l_B_ZN_201_ZN_4177
2p2l_B_ZN_202_ZN_4178
2p2l_C_ZN_201_ZN_4207
2p2l_C_ZN_202_ZN_4208
2p46_A_ZN_201_ZN_3647,2p46_A_ZN_201_ZN_3648
2p46_D_ZN_211_ZN_3659
2p46_A_ZN_202_ZN_3649
2p46_A_ZN_203_ZN_3650,2p46_A_ZN_204_ZN_3651
2p46_A_ZN_205_ZN_3652
2p46_B_ZN_206_ZN_3653
2p46_C_ZN_207_ZN_3654,2p46_C_ZN_207_ZN_3655
2p46_C_ZN_208_ZN_3656
2p46_C_ZN_209_ZN_3657

2p46_C_ZN_210_ZN_3658
2p4n_A_ZN_900_ZN_9117
2p9x_A_ZN_2001_ZN_3050
2p9x_C_ZN_2002_ZN_3055
2p9x_C_ZN_2004_ZN_3056
2p9x_D_ZN_2003_ZN_3057
2pdo_B_ZN_601_ZN_9634
2pdo_B_ZN_604_ZN_9635
2pdo_E_ZN_602_ZN_9644
2pdo_G_ZN_603_ZN_9661,2pdo_H_ZN_605_ZN_9662
2ped_A_ZN_2011_ZN_5630
2ped_A_ZN_957_ZN_5575
2ped_A_ZN_959_ZN_5611
2ped_A_ZN_962_ZN_5628
2ped_B_ZN_956_ZN_5574
2ped_B_ZN_958_ZN_5593
2ped_B_ZN_963_ZN_5629
2pgq_A_ZN_501_ZN_2444
2pgq_A_ZN_504_ZN_2445
2pgq_B_ZN_502_ZN_2460
2ph1_A_ZN_301_ZN_1900
2pig_A_ZN_335_ZN_4889
2pig_A_ZN_336_ZN_4890
2pig_B_ZN_335_ZN_4891
2pjs_B_ZN_117_ZN_1737
2pkp_A_ZN_171_ZN_1288
2pli_A_ZN_703_ZN_2699
2pli_C_ZN_713_ZN_2736
2pli_D_ZN_702_ZN_2737
2pli_D_ZN_711_ZN_2738
2pli_D_ZN_712_ZN_2739
2pli_A_ZN_704_ZN_2700
2pli_A_ZN_705_ZN_2701
2pli_B_ZN_706_ZN_2722
2pli_B_ZN_707_ZN_2723
2pli_B_ZN_708_ZN_2724
2pli_B_ZN_709_ZN_2725
2pli_B_ZN_710_ZN_2726
2pli_C_ZN_701_ZN_2735
2pms_C_ZN_502_ZN_6975
2pms_D_ZN_501_ZN_6976
2ppt_B_ZN_302_ZN_2139
2pq3_A_ZN_201_ZN_650,2pq3_A_ZN_201_ZN_651
2pq3_A_ZN_202_ZN_652
2pqk_A_ZN_401_ZN_1361
2pqk_A_ZN_402_ZN_1362
2pqk_B_ZN_403_ZN_1364
2prs_A_ZN_503_ZN_4164
2ps0_A_ZN_504_ZN_4094
2ps0_B_ZN_503_ZN_4096
2psy_A_ZN_2001_ZN_1825
2psy_A_ZN_2002_ZN_1826
2ptw_A_ZN_500_ZN_3074
2ptw_A_ZN_501_ZN_3075
2ptx_A_ZN_500_ZN_3308,2ptx_A_ZN_501_ZN_3309
2pty_A_ZN_500_ZN_3294,2pty_A_ZN_501_ZN_3295
2ptz_A_ZN_500_ZN_3290,2ptz_A_ZN_501_ZN_3291
2ptz_A_ZN_550_ZN_3292

2pu0_A_ZN_501_ZN_3300,2pu0_A_ZN_502_ZN_3301
2pu0_A_ZN_505_ZN_3302
2pu1_A_ZN_500_ZN_3310,2pu1_A_ZN_501_ZN_3311
2pu1_A_ZN_550_ZN_3312
2pve_A_ZN_301_ZN_2493
2pve_B_ZN_302_ZN_2502
2pve_C_ZN_303_ZN_2507
2pvx_A_ZN_901_ZN_6601
2pvx_B_ZN_902_ZN_6602
2pvx_C_ZN_903_ZN_6603
2pvx_D_ZN_904_ZN_6604
2pvx_E_ZN_905_ZN_6605
2pvx_F_ZN_906_ZN_6606
2pvx_G_ZN_907_ZN_6607
2pvx_H_ZN_908_ZN_6608
2pw6_A_ZN_273_ZN_1872
2pw6_A_ZN_274_ZN_1873
2px1_A_ZN_301_ZN_2749
2px1_A_ZN_302_ZN_2750
2pxr_C_ZN_401_ZN_1195
2pxr_C_ZN_402_ZN_1196
2q02_A_ZN_301_ZN_8593
2q02_B_ZN_301_ZN_8600
2q08_H_ZN_995_ZN_40835
2q08_J_ZN_996_ZN_40838
2q08_A_ZN_993_ZN_40826
2q08_D_ZN_994_ZN_40830
2q0y_A_ZN_153_ZN_1220
2q1b_A_ZN_430_ZN_2108
2q4z_A_ZN_601_ZN_4865
2q5b_A_ZN_107_ZN_2437
2q5b_B_ZN_107_ZN_2445
2q5b_C_ZN_107_ZN_2448
2q5b_C_ZN_108_ZN_2449
2q8j_A_ZN_803_ZN_2704
2q8j_A_ZN_804_ZN_2705
2qa1_A_ZN_601_ZN_3720
2qdt_A_ZN_403_ZN_2017,2qdt_A_ZN_403_ZN_2018
2qfi_A_ZN_302_ZN_4414,2qfi_A_ZN_306_ZN_4418
2qfi_A_ZN_303_ZN_4415
2qfi_A_ZN_307_ZN_4419
2qfi_B_ZN_305_ZN_4417
2qha_A_ZN_2001_ZN_5395
2qha_B_ZN_2002_ZN_5414
2qin_B_ZN_3001_ZN_7950
2qje_A_ZN_690_ZN_2687
2qje_A_ZN_691_ZN_2688
2ql0_A_ZN_53_ZN_744
2ql1_A_ZN_1_ZN_1787
2ql1_A_ZN_2_ZN_1788
2ql1_A_ZN_3_ZN_1789
2ql1_A_ZN_4_ZN_1790
2qla_A_ZN_500_ZN_3324
2qla_B_ZN_502_ZN_3368
2qla_C_ZN_501_ZN_3412
2qla_D_ZN_503_ZN_3456
2qnw_A_ZN_83_ZN_663
2qnw_A_ZN_84_ZN_664

2qnw_A_ZN_86_ZN_666
2qnw_A_ZN_87_ZN_667
2qnw_A_ZN_88_ZN_668
2qq4_A_ZN_139_ZN_10248
2qq4_J_ZN_139_ZN_10257
2qq4_B_ZN_139_ZN_10249
2qq4_C_ZN_139_ZN_10250
2qq4_D_ZN_139_ZN_10251
2qq4_E_ZN_139_ZN_10252
2qq4_F_ZN_139_ZN_10253
2qq4_G_ZN_139_ZN_10254
2qq4_H_ZN_139_ZN_10255
2qq4_I_ZN_139_ZN_10256
2qqu_A_ZN_1020_ZN_4437
2qqu_A_ZN_1021_ZN_4438
2qqu_A_ZN_1022_ZN_4439
2qqu_A_ZN_1023_ZN_4440
2qqv_A_ZN_1101_ZN_4441
2qqv_A_ZN_1102_ZN_4442
2qqv_A_ZN_1103_ZN_4443
2qqv_A_ZN_1104_ZN_4444
2qqw_A_ZN_1101_ZN_4426
2qqw_A_ZN_1102_ZN_4427
2qqw_A_ZN_1103_ZN_4428
2qqw_A_ZN_1104_ZN_4429
2qsc_H_ZN_230_ZN_3360
2qsc_H_ZN_231_ZN_3361
2qsc_L_ZN_215_ZN_3357
2qsc_L_ZN_216_ZN_3358
2qsw_A_ZN_202_ZN_805
2qsw_A_ZN_203_ZN_806
2qvv_A_ZN_403_ZN_5045
2qvv_B_ZN_339_ZN_5071
2r1w_A_ZN_215_ZN_3361
2r1w_B_ZN_217_ZN_3377
2r1x_A_ZN_215_ZN_3352
2r1y_A_ZN_216_ZN_3350
2r23_B_ZN_217_ZN_3405
2r2b_A_ZN_214_ZN_3338
2r2b_B_ZN_217_ZN_3339
2r2h_B_ZN_215_ZN_3359
2r2v_C_ZN_35_ZN_2054
2r2v_D_ZN_35_ZN_2072
2r2z_A_ZN_91_ZN_744
2r2z_A_ZN_92_ZN_745
2r2z_A_ZN_93_ZN_746
2r2z_A_ZN_94_ZN_747
2r71_A_ZN_1001_ZN_2772
2r71_A_ZN_1002_ZN_2773
2r74_A_ZN_167_ZN_2353
2r74_B_ZN_167_ZN_2364
2r92_I_ZN_204_ZN_31622
2r9j_A_ZN_687_ZN_2676
2r9j_A_ZN_688_ZN_2677
2ra6_A_ZN_803_ZN_4729
2ra6_B_ZN_801_ZN_4732
2ra6_B_ZN_802_ZN_4733
2ra6_C_ZN_804_ZN_4737

2ra6_D_ZN_805_ZN_4741
2ra6_D_ZN_806_ZN_4740
2ra7_A_ZN_1050_ZN_1005
2rb4_A_ZN_481_ZN_2633
2rc5_A_ZN_317_ZN_9545
2rc6_A_ZN_315_ZN_9501
2rcc_B_ZN_400_ZN_7489,2rcc_B_ZN_401_ZN_7490
2rcc_C_ZN_400_ZN_7527,2rcc_C_ZN_401_ZN_7528
2rf3_B_ZN_308_ZN_495
2rgl_A_ZN_1001_ZN_7617
2rgm_A_ZN_1001_ZN_7627
2rgx_A_ZN_300_ZN_1620
2rgx_A_ZN_301_ZN_1621
2rgx_A_ZN_302_ZN_1622
2rgx_A_ZN_303_ZN_1623
2rjq_A_ZN_2_ZN_2267
2rkn_A_ZN_201_ZN_667
2rkn_A_ZN_202_ZN_668,2rkn_A_ZN_203_ZN_669
2rku_A_ZN_501_ZN_2413
2ush_A_ZN_707_ZN_8029
2ush_A_ZN_708_ZN_8030
2ush_A_ZN_709_ZN_8031
2ush_B_ZN_705_ZN_8044
2ush_B_ZN_706_ZN_8045
2usn_A_ZN_262_ZN_1319
2uur_A_ZN_601_ZN_1734
2ux1_A_ZN_1173_ZN_14560
2ux1_E_ZN_1174_ZN_14585
2ux1_F_ZN_1173_ZN_14586
2ux1_F_ZN_1174_ZN_14587
2ux1_G_ZN_1173_ZN_14588
2ux1_G_ZN_1174_ZN_14589
2ux1_H_ZN_1173_ZN_14592
2ux1_H_ZN_1174_ZN_14593
2ux1_I_ZN_1173_ZN_14610
2ux1_I_ZN_1174_ZN_14611
2ux1_J_ZN_1173_ZN_14627
2ux1_A_ZN_1174_ZN_14561
2ux1_J_ZN_1174_ZN_14628
2ux1_K_ZN_1173_ZN_14645
2ux1_K_ZN_1174_ZN_14646
2ux1_L_ZN_1173_ZN_14648
2ux1_L_ZN_1174_ZN_14649
2ux1_B_ZN_1173_ZN_14562
2ux1_B_ZN_1174_ZN_14563
2ux1_C_ZN_1173_ZN_14564
2ux1_C_ZN_1174_ZN_14565
2ux1_D_ZN_1173_ZN_14567
2ux1_D_ZN_1174_ZN_14568
2ux1_E_ZN_1173_ZN_14584
2uxa_A_ZN_1263_ZN_6119
2uxa_B_ZN_1262_ZN_6120
2uxa_B_ZN_1263_ZN_6121
2uxa_C_ZN_1263_ZN_6122
2uxa_C_ZN_1264_ZN_6123
2uyd_X_ZN_201_ZN_1348
2uyd_X_ZN_202_ZN_1349,2uyd_X_ZN_203_ZN_1350,2uyd_X_ZN_207_ZN_1354
2uyd_X_ZN_204_ZN_1351

2uyd_X_ZN_205_ZN_1352
2uyd_X_ZN_206_ZN_1353
2uyd_X_ZN_208_ZN_1355
2uzi_H_ZN_1115_ZN_3007
2uzi_H_ZN_1116_ZN_3008
2uzi_R_ZN_1169_ZN_3042
2uzi_R_ZN_1170_ZN_3043
2uzi_R_ZN_1171_ZN_3044
2v08_A_ZN_1090_ZN_1273
2v08_A_ZN_1091_ZN_1274
2v08_A_ZN_1092_ZN_1275
2v08_A_ZN_1093_ZN_1276
2v08_A_ZN_1094_ZN_1277
2v08_B_ZN_1090_ZN_1334
2v08_B_ZN_1091_ZN_1335
2v08_B_ZN_1092_ZN_1336
2v08_B_ZN_1093_ZN_1337
2v0g_D_ZN_1880_ZN_17606
2v0p_A_ZN_1235_ZN_3565
2v0p_A_ZN_1236_ZN_3566
2v0p_B_ZN_1235_ZN_3567
2v0p_B_ZN_1236_ZN_3568
2v1z_A_ZN_1271_ZN_2101
2v20_A_ZN_1270_ZN_2102
2v25_A_ZN_1235_ZN_3685
2v25_A_ZN_1236_ZN_3686
2v25_B_ZN_1235_ZN_3687
2v25_B_ZN_1236_ZN_3688
2v29_A_ZN_1273_ZN_4261
2v29_A_ZN_1276_ZN_4264
2v29_A_ZN_1277_ZN_4265
2v2a_A_ZN_1276_ZN_2271
2v2b_A_ZN_1275_ZN_2115
2v8l_A_ZN_1107_ZN_829
2v9e_A_ZN_1276_ZN_4576
2v9e_A_ZN_1277_ZN_4577
2v9e_B_ZN_1276_ZN_4595
2v9e_B_ZN_1277_ZN_4596
2v9f_A_ZN_1280_ZN_2145
2v9f_A_ZN_1281_ZN_2146
2v9i_A_ZN_1276_ZN_4244
2v9i_A_ZN_1277_ZN_4245
2v9m_A_ZN_1277_ZN_4707
2v9m_B_ZN_1276_ZN_4738
2v9o_A_ZN_1276_ZN_4299
2v9o_E_ZN_1276_ZN_4301
2v9o_E_ZN_1277_ZN_4302
2v9o_E_ZN_1278_ZN_4303
2vac_A_ZN_1138_ZN_1081
2vac_A_ZN_1139_ZN_1082
2vac_A_ZN_1140_ZN_1083
2vad_A_ZN_1224_ZN_1801
2vad_A_ZN_1225_ZN_1802
2vad_A_ZN_1226_ZN_1803
2vbs_A_ZN_1135_ZN_1063
2vcg_D_ZN_1378_ZN_11276
2ves_A_ZN_1297_ZN_6949
2ves_B_ZN_1291_ZN_6981

2ves_C_ZN_1302_ZN_7018
2ves_C_ZN_1303_ZN_7019
2vh3_A_ZN_1113_ZN_1801
2vh5_H_ZN_1115_ZN_3008
2vh5_H_ZN_1116_ZN_3009
2vh5_R_ZN_1169_ZN_3007
2vh9_A_ZN_1267_ZN_4301
2vh9_B_ZN_1268_ZN_4407
2vir_A_ZN_211_ZN_5341
2vir_A_ZN_212_ZN_5342
2vir_C_ZN_1_ZN_5340
2vis_A_ZN_211_ZN_5356
2vis_A_ZN_212_ZN_5357
2vis_C_ZN_1_ZN_5355
2vit_A_ZN_211_ZN_5342
2vit_A_ZN_212_ZN_5343
2vit_C_ZN_1_ZN_5341
2vm3_A_ZN_888_ZN_2557
2vm4_A_ZN_888_ZN_2572
2vme_A_ZN_501_ZN_12123
2vme_D_ZN_501_ZN_12183
2vmj_A_ZN_1328_ZN_2478
2vmj_A_ZN_1329_ZN_2479
2vmj_A_ZN_1330_ZN_2480
2vn3_A_ZN_403_ZN_2552
2vqf_Z_ZN_140_ZN_52187
2vqg_A_ZN_1089_ZN_5377,2vqg_A_ZN_1090_ZN_5378
2vqg_D_ZN_1088_ZN_5459,2vqg_D_ZN_1089_ZN_5460
2vqg_D_ZN_1090_ZN_5461,2vqg_D_ZN_1092_ZN_5463
2vqg_D_ZN_1091_ZN_5462,2vqg_D_ZN_1093_ZN_5464
2vqg_E_ZN_1090_ZN_5465,2vqg_E_ZN_1092_ZN_5504
2vqg_F_ZN_1088_ZN_5509,2vqg_F_ZN_1091_ZN_5512
2vqg_F_ZN_1089_ZN_5510,2vqg_F_ZN_1090_ZN_5511,2vqg_F_ZN_1092_ZN_5513
2vqg_G_ZN_1090_ZN_5514,2vqg_G_ZN_1091_ZN_5515
2vqg_G_ZN_1092_ZN_5516,2vqg_G_ZN_1094_ZN_5522
2vqg_H_ZN_1090_ZN_5531,2vqg_H_ZN_1091_ZN_5532
2vqg_I_ZN_1089_ZN_5541,2vqg_I_ZN_1092_ZN_5552
2vqg_A_ZN_1091_ZN_5379,2vqg_A_ZN_1095_ZN_5391
2vqg_A_ZN_1094_ZN_5390,2vqg_A_ZN_1098_ZN_5402,2vqg_A_ZN_1099_ZN_5403
2vqg_B_ZN_1088_ZN_5404,2vqg_B_ZN_1089_ZN_5405
2vqg_B_ZN_1090_ZN_5406,2vqg_B_ZN_1095_ZN_5419
2vqg_B_ZN_1091_ZN_5407,2vqg_B_ZN_1094_ZN_5418,2vqg_I_ZN_1093_ZN_5553
2vqg_C_ZN_1091_ZN_5432,2vqg_C_ZN_1092_ZN_5433
2vqg_C_ZN_1093_ZN_5434,2vqg_C_ZN_1097_ZN_5442
2vqg_C_ZN_1094_ZN_5435,2vqg_C_ZN_1096_ZN_5441,2vqg_C_ZN_1100_ZN_5453
2vqh_A_ZN_1089_ZN_1117,2vqh_B_ZN_1091_ZN_1134
2vqh_A_ZN_1090_ZN_1118
2vqh_A_ZN_1091_ZN_1119
2vqh_A_ZN_1092_ZN_1120,2vqh_A_ZN_1093_ZN_1121
2vqh_B_ZN_1089_ZN_1132
2vqh_B_ZN_1090_ZN_1133
2vqk_A_ZN_1093_ZN_548
2vqk_A_ZN_1094_ZN_549,2vqk_A_ZN_1095_ZN_550
2vqk_A_ZN_1096_ZN_551
2vqk_A_ZN_1097_ZN_552
2vqk_A_ZN_1098_ZN_553
2vql_A_ZN_1089_ZN_2235,2vql_A_ZN_1090_ZN_2236
2vql_D_ZN_1089_ZN_2266

2vql_A_ZN_1091_ZN_2237
2vql_A_ZN_1092_ZN_2238
2vql_B_ZN_1089_ZN_2249
2vql_B_ZN_1090_ZN_2250
2vql_B_ZN_1091_ZN_2251,2vql_B_ZN_1092_ZN_2252
2vql_C_ZN_1089_ZN_2263
2vql_C_ZN_1090_ZN_2264
2vql_D_ZN_1088_ZN_2265
2vr6_A_ZN_1155_ZN_2381
2vr6_F_ZN_1154_ZN_2391
2vr7_A_ZN_1156_ZN_2252
2vr7_A_ZN_1159_ZN_2259
2vr7_A_ZN_1162_ZN_2266
2vr7_F_ZN_1156_ZN_2268
2vr8_A_ZN_1157_ZN_2311
2vr8_A_ZN_1160_ZN_2318
2vr8_F_ZN_1157_ZN_2326
2vr8_F_ZN_1160_ZN_2333
2vrs_A_ZN_1328_ZN_4656
2vrs_A_ZN_1329_ZN_4657
2vrs_A_ZN_1330_ZN_4658
2vrs_A_ZN_1331_ZN_4659
2vrs_A_ZN_1332_ZN_4660
2vrs_B_ZN_1329_ZN_4673
2vrs_C_ZN_1329_ZN_4681
2vrt_A_ZN_1506_ZN_13193
2vrz_A_ZN_1097_ZN_1526
2vrz_A_ZN_1098_ZN_1527
2vrz_A_ZN_1099_ZN_1528,2vrz_A_ZN_1101_ZN_1530
2vrz_A_ZN_1100_ZN_1529
2vrz_B_ZN_1089_ZN_1531
2vrz_B_ZN_1090_ZN_1532
2vs0_A_ZN_1086_ZN_1392
2vs0_A_ZN_1087_ZN_1393
2vs0_A_ZN_1088_ZN_1394
2vs0_A_ZN_1089_ZN_1395
2vs0_B_ZN_1089_ZN_1401
2vtg_A_ZN_1126_ZN_898
2vtg_A_ZN_1127_ZN_899
2vu4_A_ZN_1187_ZN_1140
2vw4_A_ZN_503_ZN_5149
2vw4_B_ZN_503_ZN_5165
2vw6_A_ZN_503_ZN_5157
2vw6_B_ZN_503_ZN_5173
2vw7_A_ZN_503_ZN_5177
2vw7_B_ZN_503_ZN_5193
2vxi_A_ZN_201_ZN_15959,2vxi_A_ZN_202_ZN_15960
2vxi_J_ZN_201_ZN_16497,2vxi_J_ZN_202_ZN_16498
2vxi_K_ZN_201_ZN_16554,2vxi_K_ZN_202_ZN_16555
2vxi_L_ZN_201_ZN_16611,2vxi_L_ZN_202_ZN_16612
2vxi_B_ZN_201_ZN_16021,2vxi_B_ZN_202_ZN_16022
2vxi_C_ZN_201_ZN_16088,2vxi_C_ZN_202_ZN_16089
2vxi_D_ZN_201_ZN_16150,2vxi_D_ZN_202_ZN_16151
2vxi_E_ZN_201_ZN_16207,2vxi_E_ZN_202_ZN_16208
2vxi_F_ZN_201_ZN_16269,2vxi_F_ZN_202_ZN_16270
2vxi_G_ZN_201_ZN_16321,2vxi_G_ZN_202_ZN_16322
2vxi_H_ZN_201_ZN_16378,2vxi_H_ZN_202_ZN_16379
2vxi_I_ZN_201_ZN_16440,2vxi_I_ZN_202_ZN_16441

2vxx_A_ZN_201_ZN_5552,2vxx_A_ZN_202_ZN_5553
2vxx_B_ZN_201_ZN_5554,2vxx_B_ZN_202_ZN_5555
2vxx_C_ZN_201_ZN_5577,2vxx_C_ZN_202_ZN_5578
2vxx_D_ZN_201_ZN_5587,2vxx_D_ZN_202_ZN_5588
2vyo_A_ZN_1223_ZN_1613
2vyo_A_ZN_1224_ZN_1614
2vz5_A_ZN_1131_ZN_1751
2vz5_A_ZN_1132_ZN_1752
2w0d_A_ZN_1269_ZN_5077
2w0d_C_ZN_1269_ZN_5154
2w0m_A_ZN_1236_ZN_1789
2w0m_A_ZN_1237_ZN_1790
2w0m_A_ZN_1238_ZN_1791
2w0m_A_ZN_1239_ZN_1792
2w3z_A_ZN_1312_ZN_1868
2w57_A_ZN_201_ZN_2103
2w57_A_ZN_202_ZN_2104
2w57_B_ZN_201_ZN_2105
2w57_B_ZN_202_ZN_2106
2w88_A_ZN_107_ZN_2444
2w88_A_ZN_108_ZN_2445
2w88_B_ZN_107_ZN_2447
2w88_B_ZN_108_ZN_2448
2w88_C_ZN_107_ZN_2450
2w88_C_ZN_108_ZN_2451
2w88_C_ZN_109_ZN_2452
2w8c_A_ZN_107_ZN_1663
2w8c_A_ZN_108_ZN_1664
2w8c_B_ZN_107_ZN_1666
2w8c_B_ZN_108_ZN_1667
2w8s_A_ZN_1515_ZN_16371
2w8s_B_ZN_1515_ZN_16378
2w8s_C_ZN_1515_ZN_16385
2w8s_D_ZN_1515_ZN_16402
2w9m_A_ZN_1569_ZN_8347
2w9n_A_ZN_1150_ZN_1187
2w9n_A_ZN_1151_ZN_1188
2w9n_A_ZN_1152_ZN_1189
2w9n_A_ZN_1153_ZN_1190
2wad_A_ZN_700_ZN_13833
2wad_A_ZN_701_ZN_13834
2wad_A_ZN_702_ZN_13835
2wad_B_ZN_700_ZN_13836
2wad_B_ZN_701_ZN_13837
2wad_B_ZN_702_ZN_13838
2wad_C_ZN_700_ZN_13839
2wad_C_ZN_701_ZN_13840
2wad_C_ZN_702_ZN_13841
2wae_A_ZN_1680_ZN_4615
2waq_A_ZN_1360_ZN_26470
2waq_B_ZN_1400_ZN_26473
2waq_B_ZN_1500_ZN_26474
2waq_C_ZN_1000_ZN_26475
2wd2_A_ZN_1264_ZN_2058
2wdg_Z_ZN_744_ZN_57355
2wdh_Z_ZN_744_ZN_57355
2wdk_Z_ZN_744_ZN_57293
2wdm_Z_ZN_744_ZN_57293

2wgq_A_ZN_801_ZN_11383
2wgq_B_ZN_801_ZN_11386
2wgt_A_ZN_1367_ZN_4387
2wgt_B_ZN_1367_ZN_4410
2wgt_C_ZN_1366_ZN_4411
2wgu_A_ZN_2_ZN_4387
2wgu_B_ZN_2_ZN_4410
2wgu_C_ZN_2_ZN_4433
2wh1_Z_ZN_549_ZN_58236
2wh3_Z_ZN_549_ZN_58236
2whk_A_ZN_1342_ZN_2718
2whm_A_ZN_1424_ZN_3035
2wkn_A_ZN_411_ZN_25065,2wkn_A_ZN_412_ZN_25066
2wkn_B_ZN_411_ZN_25067,2wkn_B_ZN_412_ZN_25068
2wkn_C_ZN_411_ZN_25069,2wkn_C_ZN_412_ZN_25070
2wkn_D_ZN_411_ZN_25071,2wkn_D_ZN_412_ZN_25072
2wkn_E_ZN_411_ZN_25073,2wkn_E_ZN_412_ZN_25074
2wkn_F_ZN_411_ZN_25075,2wkn_F_ZN_412_ZN_25076
2wkn_G_ZN_411_ZN_25077,2wkn_G_ZN_412_ZN_25078
2wkn_H_ZN_411_ZN_25079,2wkn_H_ZN_412_ZN_25080
2wri_Z_ZN_2_ZN_60237
2wri_Z_ZN_3_ZN_60238
2wrk_Z_ZN_2_ZN_60237
2wrk_Z_ZN_3_ZN_60238
2wrn_1_ZN_2_ZN_59573
2wrn_1_ZN_5_ZN_59576
2wrq_1_ZN_2_ZN_59573
2wrq_1_ZN_5_ZN_59576
2wvk_A_ZN_1393_ZN_6092
2wvk_A_ZN_1394_ZN_6093
2wvk_B_ZN_1392_ZN_6094
2wvk_B_ZN_1393_ZN_6095
2wvk_B_ZN_1394_ZN_6096
2wvl_A_ZN_1392_ZN_6069
2wvl_A_ZN_1393_ZN_6070
2wvl_B_ZN_1392_ZN_6111
2wvl_B_ZN_1393_ZN_6112
2wvl_B_ZN_1394_ZN_6113
2wvm_A_ZN_1392_ZN_5923
2wvm_B_ZN_1392_ZN_5964
2wvm_B_ZN_1393_ZN_5965
2wwn_A_ZN_1162_ZN_2214
2wwn_A_ZN_1164_ZN_2216
2wwn_A_ZN_1165_ZN_2217
2wwn_A_ZN_1166_ZN_2218
2wwn_B_ZN_1162_ZN_2231
2wwo_A_ZN_1163_ZN_2247
2wwo_A_ZN_1164_ZN_2248
2wwo_A_ZN_1165_ZN_2249
2wwo_B_ZN_1163_ZN_2251
2wwo_B_ZN_1164_ZN_2252
2wxd_M_ZN_1502_ZN_4410
2wyt_A_ZN_154_ZN_4726
2wyt_F_ZN_154_ZN_4778
2wyz_A_ZN_154_ZN_2289
2wyz_F_ZN_154_ZN_2319
2wz0_A_ZN_154_ZN_2271
2wz5_A_ZN_154_ZN_2274

2wz6_A_ZN_154_ZN_2287
2wz6_F_ZN_154_ZN_2318
2x2o_A_ZN_1120_ZN_1020
2x2o_A_ZN_1121_ZN_1021,2x2o_A_ZN_1123_ZN_1023
2x2o_A_ZN_1122_ZN_1022
2x2p_A_ZN_1120_ZN_1005
2x2p_A_ZN_1121_ZN_1006,2x2p_A_ZN_1123_ZN_1008
2x2p_A_ZN_1122_ZN_1007
2x4h_A_ZN_1139_ZN_4207
2x4h_D_ZN_1141_ZN_4219,2x4h_D_ZN_1142_ZN_4220
2x4h_A_ZN_1140_ZN_4208,2x4h_A_ZN_1141_ZN_4209
2x4h_A_ZN_1142_ZN_4210
2x4h_B_ZN_1139_ZN_4211,2x4h_B_ZN_1140_ZN_4212
2x4h_B_ZN_1141_ZN_4213
2x4h_B_ZN_1142_ZN_4214
2x4h_C_ZN_1139_ZN_4215,2x4h_C_ZN_1140_ZN_4216
2x4h_D_ZN_1139_ZN_4217
2x4h_D_ZN_1140_ZN_4218
2x4k_A_ZN_1063_ZN_1011
2x4k_A_ZN_1064_ZN_1012
2x4k_A_ZN_1065_ZN_1013
2x4k_B_ZN_1064_ZN_1023
2x4k_B_ZN_1065_ZN_1024
2x5r_A_ZN_1125_ZN_969
2x5r_A_ZN_1127_ZN_971
2x5r_A_ZN_1128_ZN_972
2x6p_B_ZN_1030_ZN_703,2x6p_B_ZN_1031_ZN_704
2x6p_B_ZN_1032_ZN_705,2x6p_B_ZN_1033_ZN_706
2x6p_C_ZN_1030_ZN_707
2x6p_C_ZN_1031_ZN_708
2x7s_A_ZN_1266_ZN_2152
2x7t_A_ZN_1264_ZN_2088
2x7u_A_ZN_1262_ZN_2052
2xb3_A_ZN_1001_ZN_1206
2xb3_A_ZN_1002_ZN_1207
2xb3_A_ZN_1003_ZN_1208
2xb3_A_ZN_1004_ZN_1209
2xb3_A_ZN_1005_ZN_1210
2xbq_A_ZN_1107_ZN_1714
2xbq_A_ZN_1108_ZN_1715
2xc2_A_ZN_1108_ZN_891
2xev_A_ZN_1123_ZN_3019
2xev_B_ZN_1125_ZN_3020
2xev_B_ZN_1126_ZN_3021,2xev_B_ZN_1127_ZN_3022
2xev_C_ZN_1126_ZN_3024
2xev_C_ZN_1127_ZN_3025
2xg1_Z_ZN_185_ZN_55295
2xgw_A_ZN_1176_ZN_1324,2xgw_A_ZN_1176_ZN_1325,2xgw_A_ZN_1177_ZN_1326
2xgw_A_ZN_1178_ZN_1327
2xjk_A_ZN_156_ZN_1136
2xjk_A_ZN_157_ZN_1137
2xjk_A_ZN_158_ZN_1138
2xjl_A_ZN_156_ZN_1157
2xjl_A_ZN_157_ZN_1158
2xjl_A_ZN_158_ZN_1159
2xjz_C_ZN_204_ZN_6110
2xk5_A_ZN_1074_ZN_1117
2xk5_A_ZN_1075_ZN_1118

2xk5_A_ZN_1076_ZN_1119
2xk5_A_ZN_1077_ZN_1120
2xk5_B_ZN_1076_ZN_1121
2xk5_B_ZN_1077_ZN_1122
2xk5_B_ZN_1078_ZN_1123
2xl9_A_ZN_1269_ZN_3669
2xl9_A_ZN_1270_ZN_3670
2xl9_B_ZN_1269_ZN_3679
2xod_A_ZN_1120_ZN_1047
2xoe_A_ZN_1120_ZN_1008
2xoe_A_ZN_1121_ZN_1009,2xoe_A_ZN_1122_ZN_1010
2xq8_C_ZN_1317_ZN_12656
2xq8_C_ZN_1318_ZN_12657
2xq8_D_ZN_1317_ZN_12658
2xqc_A_ZN_1138_ZN_3451
2xqc_A_ZN_1139_ZN_3452
2xqc_A_ZN_1140_ZN_3453
2xqc_B_ZN_1038_ZN_3454
2xqc_C_ZN_1007_ZN_3455
2xqc_D_ZN_1141_ZN_3456
2xqc_D_ZN_1142_ZN_3457
2xqc_E_ZN_1038_ZN_3458
2xqc_E_ZN_1039_ZN_3459
2xqd_1_ZN_4_ZN_59825
2xqd_1_ZN_7_ZN_59827
2xsc_B_ZN_1070_ZN_2780
2xsc_C_ZN_1070_ZN_2781
2xsc_E_ZN_1070_ZN_2782
2xsn_A_ZN_1528_ZN_10725
2xsn_B_ZN_1529_ZN_10726
2xsn_C_ZN_1536_ZN_10727
2xsn_D_ZN_1536_ZN_10728
2xum_A_ZN_501_ZN_3014
2xwc_A_ZN_1320_ZN_1593
2y33_A_ZN_900_ZN_1693
2yr2_B_ZN_1001_ZN_2292
2yww_A_ZN_501_ZN_2243
2yww_B_ZN_502_ZN_2307
2yx1_A_ZN_501_ZN_5171
2yx1_B_ZN_510_ZN_5207
2yx1_A_ZN_502_ZN_5172
2yx1_A_ZN_503_ZN_5173
2yx1_A_ZN_504_ZN_5174
2yx1_B_ZN_505_ZN_5202
2yx1_B_ZN_506_ZN_5203
2yx1_B_ZN_507_ZN_5204
2yx1_B_ZN_508_ZN_5205
2yx1_B_ZN_509_ZN_5206
2z2y_A_ZN_2001_ZN_5642
2z2y_B_ZN_2002_ZN_5643
2z2y_B_ZN_2006_ZN_5644
2z2y_C_ZN_2003_ZN_5652
2z2y_D_ZN_2004_ZN_5653
2z2y_D_ZN_2005_ZN_5654
2z30_B_ZN_2001_ZN_2837
2z45_A_ZN_1001_ZN_1766
2z45_A_ZN_1002_ZN_1767
2z45_A_ZN_1003_ZN_1768

2z45_A_ZN_1007_ZN_1769
2z45_A_ZN_1008_ZN_1770
2z45_B_ZN_1004_ZN_1771
2z45_B_ZN_1005_ZN_1772
2z45_B_ZN_1006_ZN_1773
2z45_B_ZN_1009_ZN_1774
2z56_B_ZN_2001_ZN_2826
2z57_B_ZN_2001_ZN_2829
2z58_B_ZN_263_ZN_2842
2z6m_A_ZN_706_ZN_16853
2z6m_B_ZN_708_ZN_16855
2z6m_G_ZN_707_ZN_16889
2z6m_H_ZN_709_ZN_16891
2z6m_I_ZN_710_ZN_16901
2z8d_A_ZN_3_ZN_6048
2z8d_A_ZN_4_ZN_6049
2z8d_B_ZN_3_ZN_6088
2z8d_B_ZN_4_ZN_6089
2z8d_B_ZN_5_ZN_6090,2z8d_B_ZN_6_ZN_6091
2z8d_B_ZN_7_ZN_6092
2z8e_A_ZN_3_ZN_6048
2z8e_A_ZN_4_ZN_6049
2z8e_B_ZN_3_ZN_6088
2z8e_B_ZN_4_ZN_6089
2z8e_B_ZN_5_ZN_6090,2z8e_B_ZN_6_ZN_6091
2z8e_B_ZN_7_ZN_6092
2z8f_A_ZN_11_ZN_6070
2z8f_A_ZN_13_ZN_6071
2z8f_B_ZN_12_ZN_6134
2z8f_B_ZN_14_ZN_6135
2z8f_B_ZN_15_ZN_6136
2z8x_A_ZN_628_ZN_4565
2z8x_A_ZN_629_ZN_4566
2z8z_A_ZN_629_ZN_4566
2z8z_A_ZN_630_ZN_4567
2z94_A_ZN_901_ZN_2373
2z9j_A_DTZ_902_ZN_4698
2z9j_B_DTZ_901_ZN_4709
2z9k_A_DOZ_901_ZN_4743
2z9k_B_DOZ_902_ZN_4756
2z9l_A_DAZ_701_ZN_4667
2z9l_B_DAZ_702_ZN_4678
2zc0_A_ZN_408_ZN_12449
2zc0_B_ZN_408_ZN_12471
2zc0_C_ZN_408_ZN_12493
2zc0_D_ZN_408_ZN_12515
2zc2_A_ZN_501_ZN_1255
2zc2_A_ZN_504_ZN_1256
2zc2_A_ZN_505_ZN_1257
2zc2_A_ZN_507_ZN_1258
2zc2_B_ZN_502_ZN_1259
2zc2_B_ZN_503_ZN_1260
2zc2_B_ZN_506_ZN_1261
2zc2_B_ZN_508_ZN_1262
2zc2_B_ZN_509_ZN_1263
2zc5_B_ZN_801_ZN_6329,2zc5_B_ZN_804_ZN_6332
2zc5_B_ZN_802_ZN_6330
2zc5_B_ZN_803_ZN_6331

2zc5_D_ZN_805_ZN_6357
2zc5_D_ZN_806_ZN_6358
2zc5_D_ZN_807_ZN_6359
2zc5_D_ZN_808_ZN_6360
2zc6_B_ZN_801_ZN_6330,2zc6_B_ZN_804_ZN_6333
2zc6_B_ZN_802_ZN_6331
2zc6_B_ZN_803_ZN_6332
2zc6_D_ZN_805_ZN_6359,2zc6_D_ZN_808_ZN_6362
2zc6_D_ZN_806_ZN_6360
2zc6_D_ZN_807_ZN_6361
2zcb_A_ZN_77_ZN_1741
2zcb_A_ZN_78_ZN_1742
2zcb_A_ZN_79_ZN_1743
2zcb_B_ZN_77_ZN_1744
2zcb_B_ZN_78_ZN_1745
2zcc_A_ZN_77_ZN_1677
2zcc_A_ZN_78_ZN_1678
2zcc_A_ZN_79_ZN_1679
2zcc_B_ZN_77_ZN_1680
2zcc_B_ZN_78_ZN_1681
2zcc_B_ZN_79_ZN_1682
2ze7_A_ZN_301_ZN_1817
2ze7_A_ZN_302_ZN_1818
2zj6_A_ZN_627_ZN_4561
2zj6_A_ZN_628_ZN_4562
2zj7_A_ZN_628_ZN_4562
2zj7_A_ZN_629_ZN_4563
2zjs_L_ZN_223_ZN_6935
2zjs_Y_ZN_435_ZN_6936
2zmb_A_ZN_689_ZN_2773
2zmb_A_ZN_690_ZN_2774
2zn8_A_ZN_991_ZN_1384
2zn8_A_ZN_992_ZN_1385,2zn8_A_ZN_994_ZN_1387
2zn8_A_ZN_993_ZN_1386
2zne_A_ZN_991_ZN_3007
2zne_A_ZN_992_ZN_3008,2zne_A_ZN_994_ZN_3010
2zne_A_ZN_993_ZN_3009
2zne_B_ZN_991_ZN_3012
2zne_B_ZN_992_ZN_3013,2zne_B_ZN_994_ZN_3015
2zne_B_ZN_993_ZN_3014
2zrt_A_ZN_990_ZN_10976
2zrt_D_ZN_1_ZN_11011
2zrt_D_ZN_990_ZN_10989
2zrt_D_ZN_991_ZN_10990,2zrt_D_ZN_993_ZN_10992
2zrt_D_ZN_992_ZN_10991,2zrt_D_ZN_994_ZN_10993
2zrt_E_ZN_990_ZN_10994
2zrt_E_ZN_991_ZN_10995,2zrt_E_ZN_993_ZN_10997
2zrt_E_ZN_992_ZN_10996,2zrt_E_ZN_994_ZN_10998
2zrt_F_ZN_990_ZN_10999
2zrt_F_ZN_991_ZN_11000,2zrt_F_ZN_993_ZN_11002
2zrt_F_ZN_992_ZN_11001,2zrt_F_ZN_994_ZN_11003
2zrt_A_ZN_991_ZN_10977,2zrt_A_ZN_992_ZN_10978
2zrt_G_ZN_990_ZN_11004
2zrt_G_ZN_991_ZN_11005,2zrt_G_ZN_992_ZN_11006
2zrt_G_ZN_993_ZN_11007,2zrt_G_ZN_994_ZN_11008
2zrt_H_ZN_990_ZN_11009,2zrt_H_ZN_992_ZN_11010
2zrt_B_ZN_2_ZN_11012
2zrt_B_ZN_990_ZN_10979

2zrt_B_ZN_991_ZN_10980,2zrt_B_ZN_993_ZN_10982
2zrt_B_ZN_992_ZN_10981,2zrt_B_ZN_994_ZN_10983
2zrt_C_ZN_990_ZN_10984
2zrt_C_ZN_991_ZN_10985,2zrt_C_ZN_993_ZN_10987
2zrt_C_ZN_992_ZN_10986,2zrt_C_ZN_994_ZN_10988
2zsg_A_ZN_1001_ZN_5578
2ztg_A_ZN_901_ZN_5924
2ztx_A_DTZ_501_ZN_1401
2zu2_A_DTZ_5517_ZN_4630
2zu2_B_DTZ_5518_ZN_4633
2zum_A_ZN_460_ZN_3209,2zum_A_ZN_460_ZN_3210
2zum_A_ZN_461_ZN_3211,2zum_A_ZN_461_ZN_3212,2zum_A_ZN_463_ZN_3215,2zum_A_ZN_463_ZN_3216
2zum_A_ZN_462_ZN_3213,2zum_A_ZN_462_ZN_3214
2zum_A_ZN_464_ZN_3217,2zum_A_ZN_464_ZN_3218,2zum_A_ZN_464_ZN_3219
2zum_A_ZN_465_ZN_3220,2zum_A_ZN_465_ZN_3221,2zum_A_ZN_465_ZN_3222
2zum_A_ZN_466_ZN_3223,2zum_A_ZN_466_ZN_3224
2zum_A_ZN_467_ZN_3225
2zum_A_ZN_467_ZN_3226
2zvl_B_ZN_262_ZN_11946
2zvl_E_ZN_262_ZN_11945
2zvl_V_ZN_1_ZN_11941
2zvl_W_ZN_2_ZN_11942
2zvl_Y_ZN_3_ZN_11943
2zvl_Z_ZN_4_ZN_11944
2zwi_A_ZN_410_ZN_6121
2zwi_B_ZN_2_ZN_6169
2zwr_B_ZN_211_ZN_3075
2zwr_B_ZN_212_ZN_3076
2zwr_B_ZN_213_ZN_3077
2zwr_A_ZN_210_ZN_3066
2zwr_A_ZN_211_ZN_3067
2zwr_A_ZN_212_ZN_3068
2zwr_A_ZN_213_ZN_3069
2zwr_A_ZN_214_ZN_3070
2zwr_A_ZN_215_ZN_3071
2zwr_B_ZN_210_ZN_3074
2zzw_A_ZN_362_ZN_5283
2zzw_B_ZN_362_ZN_5290
3a1z_A_ZN_226_ZN_6472
3a1z_C_ZN_227_ZN_6514
3a1z_C_ZN_228_ZN_6515
3a1z_C_ZN_229_ZN_6516
3a1z_C_ZN_230_ZN_6517
3a1z_C_ZN_231_ZN_6518
3a1z_D_ZN_226_ZN_6535
3a1z_D_ZN_227_ZN_6536
3a1z_D_ZN_228_ZN_6537,3a1z_D_ZN_229_ZN_6538
3a1z_D_ZN_230_ZN_6539
3a1z_A_ZN_227_ZN_6473
3a1z_A_ZN_228_ZN_6474
3a1z_A_ZN_229_ZN_6475
3a1z_A_ZN_230_ZN_6476
3a1z_B_ZN_226_ZN_6493
3a1z_B_ZN_227_ZN_6494
3a1z_B_ZN_228_ZN_6495,3a1z_B_ZN_229_ZN_6496
3a1z_C_ZN_226_ZN_6513
3a3n_A_ZN_399_ZN_2815

3a3n_B_ZN_68_ZN_2816
3a3n_B_ZN_69_ZN_2817
3a3o_B_ZN_65_ZN_2787
3a3p_B_ZN_70_ZN_2820
3a7q_A_ZN_4005_ZN_5785
3aak_A_ZN_991_ZN_1387
3aak_A_ZN_992_ZN_1388
3aak_A_ZN_993_ZN_1389
3af5_A_ZN_663_ZN_5152
3af5_A_ZN_664_ZN_5153
3aht_A_ZN_1001_ZN_7628
3ahv_A_ZN_1001_ZN_7632
3b4n_A_ZN_711_ZN_4750,3b4n_A_ZN_716_ZN_4752
3b4n_A_ZN_714_ZN_4751
3b4n_B_ZN_712_ZN_4766
3b4n_B_ZN_713_ZN_4767,3b4n_B_ZN_715_ZN_4768
3b6p_A_ZN_800_ZN_6762
3b6p_B_ZN_800_ZN_6785
3b6p_C_ZN_800_ZN_6808
3b6p_D_ZN_800_ZN_6831
3b8y_B_ZN_711_ZN_4553
3b90_A_ZN_711_ZN_3334
3b90_A_ZN_712_ZN_3335
3b90_B_ZN_713_ZN_3342
3b90_B_ZN_714_ZN_3343
3b92_A_ZN_503_ZN_2055
3bb6_A_ZN_128_ZN_3532
3bb6_B_ZN_128_ZN_3533
3bft_A_ZN_1002_ZN_6094
3bft_A_ZN_1003_ZN_6095
3bft_B_ZN_1001_ZN_6115
3bft_B_ZN_1004_ZN_6116
3bft_B_ZN_1005_ZN_6117
3bgo_S_ZN_278_ZN_2400
3bgo_S_ZN_279_ZN_2401
3bgo_S_ZN_280_ZN_2402
3bgo_S_ZN_281_ZN_2403
3bkf_A_ZN_500_ZN_510,3bkf_A_ZN_501_ZN_511
3bkm_L_ZN_216_ZN_3420
3bkn_A_ZN_201_ZN_15595,3bkn_A_ZN_202_ZN_15596
3bkn_J_ZN_201_ZN_16068,3bkn_J_ZN_202_ZN_16069
3bkn_K_ZN_201_ZN_16086,3bkn_K_ZN_202_ZN_16087
3bkn_L_ZN_201_ZN_16132,3bkn_L_ZN_202_ZN_16133
3bkn_B_ZN_201_ZN_15684,3bkn_B_ZN_202_ZN_15685
3bkn_C_ZN_201_ZN_15689,3bkn_C_ZN_202_ZN_15690
3bkn_D_ZN_201_ZN_15778,3bkn_D_ZN_202_ZN_15779
3bkn_E_ZN_201_ZN_15795,3bkn_E_ZN_202_ZN_15796
3bkn_F_ZN_201_ZN_15884,3bkn_F_ZN_202_ZN_15885
3bkn_G_ZN_201_ZN_15887,3bkn_G_ZN_202_ZN_15888
3bkn_H_ZN_201_ZN_15975,3bkn_H_ZN_202_ZN_15976
3bkn_I_ZN_201_ZN_15978,3bkn_I_ZN_202_ZN_15979
3bkq_X_ZN_500_ZN_3472
3boc_A_ZN_1001_ZN_1570
3bpc_A_ZN_215_ZN_3318
3bpc_B_ZN_217_ZN_3333
3bpu_A_ZN_901_ZN_638
3bpu_A_ZN_902_ZN_639
3byr_A_ZN_504_ZN_738

3byw_A_ZN_14_ZN_9465
3byw_D_ZN_18_ZN_9494
3byw_D_ZN_1_ZN_9491
3byw_E_ZN_10_ZN_9509
3byw_E_ZN_19_ZN_9510
3byw_E_ZN_3_ZN_9507
3byw_E_ZN_9_ZN_9508
3byw_F_ZN_11_ZN_9524
3byw_F_ZN_4_ZN_9523
3byw_G_ZN_12_ZN_9526
3byw_G_ZN_5_ZN_9525
3byw_A_ZN_7_ZN_9464
3byw_B_ZN_15_ZN_9472
3byw_B_ZN_6_ZN_9470
3byw_B_ZN_8_ZN_9471
3byw_C_ZN_16_ZN_9486
3byw_C_ZN_2_ZN_9485
3byw_D_ZN_13_ZN_9492
3byw_D_ZN_17_ZN_9493
3c04_A_ZN_500_ZN_3454
3c2s_A_ZN_448_ZN_1787
3c2s_A_ZN_449_ZN_1788
3c2s_A_ZN_450_ZN_1789
3c2s_A_ZN_451_ZN_1790
3c3u_A_ZN_324_ZN_2594
3c62_A_ZN_107_ZN_3257
3c62_B_ZN_107_ZN_3302
3c62_C_ZN_107_ZN_3349
3c62_D_ZN_107_ZN_3393
3c63_A_ZN_107_ZN_3249
3c63_B_ZN_107_ZN_3294
3c63_C_ZN_107_ZN_3341
3c63_D_ZN_107_ZN_3385
3c9l_A_ZN_1349_ZN_2686
3cao_A_ZN_111_ZN_808
3cao_A_ZN_112_ZN_809
3cao_A_ZN_113_ZN_810
3cao_A_ZN_114_ZN_811
3car_A_ZN_111_ZN_788
3car_A_ZN_112_ZN_789
3car_A_ZN_113_ZN_790
3car_A_ZN_114_ZN_791
3ce2_A_ZN_701_ZN_4810
3ce2_A_ZN_702_ZN_4811
3ce2_A_ZN_704_ZN_4813
3ce2_A_ZN_705_ZN_4814
3ce2_A_ZN_706_ZN_4815
3cfl_A_ZN_3_ZN_2695
3cfl_A_ZN_4_ZN_2696
3cgh_A_ZN_600_ZN_4037
3chv_A_ZN_301_ZN_2262
3ci8_A_ZN_8_ZN_2681
3ci8_A_ZN_9_ZN_2682
3ciz_A_ZN_571_ZN_8748
3ciz_B_ZN_571_ZN_8760
3cjj_A_ZN_2_ZN_1706
3cjj_A_ZN_3_ZN_1707
3cjj_A_ZN_4_ZN_1708,3cjj_A_ZN_4_ZN_1709

3cjj_A_ZN_5_ZN_1710
3clh_A_ZN_344_ZN_4905
3clh_B_ZN_344_ZN_4950
3cne_A_ZN_301_ZN_5336
3cne_B_ZN_301_ZN_5363
3cne_C_ZN_301_ZN_5365
3cne_D_ZN_301_ZN_5392
3cnq_S_ZN_278_ZN_2404
3cnq_S_ZN_279_ZN_2405
3cnq_S_ZN_280_ZN_2406
3cnq_S_ZN_281_ZN_2407
3cnq_S_ZN_282_ZN_2408
3co0_S_ZN_278_ZN_2396
3co0_S_ZN_279_ZN_2397
3co0_S_ZN_280_ZN_2398
3co0_S_ZN_281_ZN_2399
3cp0_A_ZN_101_ZN_553
3cp0_A_ZN_102_ZN_554
3cp0_A_ZN_103_ZN_555
3cpm_A_ZN_2_ZN_1485
3cpm_A_ZN_3_ZN_1486
3cpm_A_ZN_4_ZN_1487
3cpm_A_ZN_5_ZN_1488
3cqj_A_ZN_286_ZN_4391
3cqj_B_ZN_286_ZN_4393
3cqk_A_ZN_286_ZN_4388
3cqk_B_ZN_286_ZN_4395
3cqq_A_ZN_155_ZN_1917
3cqq_B_ZN_154_ZN_1921
3crb_A_ZN_3_ZN_2695
3crb_A_ZN_4_ZN_2696
3csv_A_ZN_333_ZN_2558
3cvd_A_ZN_107_ZN_2479
3cvd_A_ZN_108_ZN_2480
3cvd_C_ZN_106_ZN_2482
3cvd_C_ZN_108_ZN_2484
3cw1_0_ZN_478_ZN_3404
3cw1_9_ZN_278_ZN_3403
3cw1_L_ZN_78_ZN_3402
3cw1_l_ZN_678_ZN_3405
3d0i_B_ZN_901_ZN_12540
3d1t_A_ZN_258_ZN_3830
3d1t_B_ZN_258_ZN_3832
3d2z_A_ZN_263_ZN_2075
3d71_A_ZN_501_ZN_2756
3d7v_A_ZN_1_ZN_1360
3d7v_A_ZN_2_ZN_1361
3d7v_A_ZN_3_ZN_1362
3d7v_A_ZN_4_ZN_1363
3d8c_A_ZN_350_ZN_2825
3dci_A_ZN_219_ZN_4625
3dco_A_ZN_900_ZN_8891
3dfm_A_ZN_403_ZN_2078
3dfm_A_ZN_404_ZN_2079
3dgd_A_ZN_128_ZN_4205
3dgd_C_ZN_129_ZN_4258
3dgd_C_ZN_134_ZN_4279
3dgd_D_ZN_128_ZN_4280

3dgd_D_ZN_129_ZN_4281
3dgd_D_ZN_130_ZN_4282
3dgd_D_ZN_131_ZN_4283
3dgd_A_ZN_129_ZN_4206
3dgd_A_ZN_130_ZN_4207
3dgd_A_ZN_133_ZN_4220
3dgd_B_ZN_128_ZN_4221
3dgd_B_ZN_129_ZN_4222
3dgd_B_ZN_130_ZN_4223
3dgd_B_ZN_134_ZN_4256
3dgd_C_ZN_128_ZN_4257
3dgl_A_ZN_80_ZN_590
3dgm_A_ZN_80_ZN_585
3dgn_A_ZN_80_ZN_616
3dgo_A_ZN_80_ZN_584
3did_A_ZN_128_ZN_3832
3did_D_ZN_128_ZN_3873
3did_D_ZN_129_ZN_3874
3did_D_ZN_130_ZN_3875
3did_A_ZN_129_ZN_3833
3did_A_ZN_130_ZN_3834
3did_B_ZN_128_ZN_3857
3did_B_ZN_129_ZN_3858
3did_B_ZN_130_ZN_3859
3did_C_ZN_128_ZN_3866
3did_C_ZN_129_ZN_3867
3did_C_ZN_130_ZN_3868
3dln_A_ZN_301_ZN_2043
3dow_A_ZN_201_ZN_1039
3dp4_A_ZN_301_ZN_2033
3dp4_A_ZN_302_ZN_2034
3dp6_A_ZN_301_ZN_6088
3dp6_A_ZN_302_ZN_6089
3dp6_B_ZN_301_ZN_6090
3dp6_B_ZN_302_ZN_6091
3dp6_C_ZN_301_ZN_6092
3dp6_C_ZN_302_ZN_6093
3dtk_A_ZN_283_ZN_1876,3dtk_A_ZN_284_ZN_1877
3dug_D_ZN_426_ZN_24255
3dug_D_ZN_427_ZN_24256
3dug_E_ZN_426_ZN_24271
3dug_E_ZN_427_ZN_24272
3dug_F_ZN_426_ZN_24275
3dug_F_ZN_427_ZN_24276
3dug_A_ZN_426_ZN_24177
3dug_G_ZN_426_ZN_24285
3dug_G_ZN_427_ZN_24286
3dug_H_ZN_426_ZN_24295
3dug_H_ZN_427_ZN_24296
3dug_A_ZN_427_ZN_24190
3dug_B_ZN_426_ZN_24229
3dug_B_ZN_427_ZN_24230
3dug_C_ZN_426_ZN_24245
3dug_C_ZN_427_ZN_24246
3dxs_X_ZN_75_ZN_631
3dyc_A_ZN_453_ZN_6578
3dyc_A_ZN_454_ZN_6579
3dza_A_ZN_501_ZN_5572

3dza_D_ZN_502_ZN_5657
3dza_A_ZN_502_ZN_5573
3dza_A_ZN_503_ZN_5574
3dza_B_ZN_502_ZN_5595
3dza_B_ZN_503_ZN_5596
3dza_C_ZN_501_ZN_5633
3dza_C_ZN_503_ZN_5634
3dza_C_ZN_504_ZN_5635
3dza_C_ZN_505_ZN_5636
3e1o_A_ZN_300_ZN_15654,3e1o_A_ZN_301_ZN_15655
3e1o_J_ZN_300_ZN_15952,3e1o_J_ZN_301_ZN_15953
3e1o_K_ZN_300_ZN_15959,3e1o_K_ZN_301_ZN_15960
3e1o_L_ZN_300_ZN_16009,3e1o_L_ZN_301_ZN_16010
3e1o_B_ZN_300_ZN_15709,3e1o_B_ZN_301_ZN_15710
3e1o_C_ZN_300_ZN_15716,3e1o_C_ZN_301_ZN_15717
3e1o_D_ZN_300_ZN_15728,3e1o_D_ZN_301_ZN_15729
3e1o_E_ZN_300_ZN_15778,3e1o_E_ZN_301_ZN_15779
3e1o_F_ZN_300_ZN_15828,3e1o_F_ZN_301_ZN_15829
3e1o_G_ZN_300_ZN_15840,3e1o_G_ZN_301_ZN_15841
3e1o_H_ZN_300_ZN_15852,3e1o_H_ZN_301_ZN_15853
3e1o_I_ZN_300_ZN_15902,3e1o_I_ZN_301_ZN_15903
3e1p_A_ZN_300_ZN_15810,3e1p_A_ZN_301_ZN_15811
3e1p_J_ZN_300_ZN_16074,3e1p_J_ZN_301_ZN_16075
3e1p_K_ZN_300_ZN_16125,3e1p_K_ZN_301_ZN_16126
3e1p_L_ZN_300_ZN_16176,3e1p_L_ZN_301_ZN_16177
3e1p_B_ZN_300_ZN_15866,3e1p_B_ZN_301_ZN_15867
3e1p_C_ZN_300_ZN_15879,3e1p_C_ZN_301_ZN_15880
3e1p_D_ZN_300_ZN_15930,3e1p_D_ZN_301_ZN_15931
3e1p_E_ZN_300_ZN_15938,3e1p_E_ZN_301_ZN_15939
3e1p_F_ZN_300_ZN_15946,3e1p_F_ZN_301_ZN_15947
3e1p_G_ZN_300_ZN_16002,3e1p_G_ZN_301_ZN_16003
3e1p_H_ZN_300_ZN_16015,3e1p_H_ZN_301_ZN_16016
3e1p_I_ZN_300_ZN_16066,3e1p_I_ZN_301_ZN_16067
3e1z_A_ZN_111_ZN_2560
3e2c_A_ZN_200_ZN_2727
3e2c_B_ZN_200_ZN_2746
3e7l_A_ZN_2_ZN_2032
3e7l_B_ZN_3_ZN_2033
3e7l_B_ZN_4_ZN_2034
3e7l_C_ZN_1_ZN_2035
3e7m_A_ZN_900_ZN_6813
3e9q_B_ZN_256_ZN_4308
3e9x_A_ZN_689_ZN_2738
3e9x_A_ZN_690_ZN_2739
3ea1_A_ZN_299_ZN_4819
3ea1_A_ZN_300_ZN_4820
3ea1_A_ZN_301_ZN_4821
3ea1_A_ZN_302_ZN_4822
3ea1_B_ZN_299_ZN_4823
3ea1_B_ZN_300_ZN_4824
3ea2_A_ZN_901_ZN_4831
3ea2_A_ZN_902_ZN_4832
3ea2_A_ZN_903_ZN_4833
3ea2_A_ZN_905_ZN_4834
3ea2_B_ZN_904_ZN_4847
3ea2_B_ZN_906_ZN_4848
3ebz_A_ZN_403_ZN_1578
3ebz_A_ZN_405_ZN_1579

3ebz_A_ZN_406_ZN_1580
3ebz_B_ZN_401_ZN_1641
3ebz_B_ZN_402_ZN_1642,3ebz_B_ZN_409_ZN_1646
3ebz_B_ZN_404_ZN_1643
3ebz_B_ZN_407_ZN_1644
3ebz_B_ZN_408_ZN_1645
3ec0_A_ZN_403_ZN_1615
3ec0_A_ZN_405_ZN_1616
3ec0_A_ZN_406_ZN_1617
3ec0_A_ZN_410_ZN_1618
3ec0_B_ZN_401_ZN_1642
3ec0_B_ZN_402_ZN_1643,3ec0_B_ZN_409_ZN_1647
3ec0_B_ZN_404_ZN_1644
3ec0_B_ZN_407_ZN_1645
3ec0_B_ZN_408_ZN_1646
3ecg_A_ZN_402_ZN_1612
3ecg_B_ZN_409_ZN_1645
3ecg_A_ZN_404_ZN_1613
3ecg_A_ZN_406_ZN_1614
3ecg_A_ZN_407_ZN_1615
3ecg_A_ZN_411_ZN_1616
3ecg_B_ZN_401_ZN_1641
3ecg_B_ZN_403_ZN_1642,3ecg_B_ZN_410_ZN_1646
3ecg_B_ZN_405_ZN_1643
3ecg_B_ZN_408_ZN_1644
3edg_A_ZN_211_ZN_1616
3edh_A_ZN_211_ZN_1699
3edi_A_ZN_211_ZN_1604
3edl_A_ZN_900_ZN_15409
3ee6_A_ZN_576_ZN_8217
3ee6_A_ZN_577_ZN_8218
3ee6_A_ZN_578_ZN_8219
3ee6_A_ZN_579_ZN_8220
3ee6_B_ZN_576_ZN_8280
3ee6_B_ZN_577_ZN_8281
3ee6_B_ZN_578_ZN_8282
3ee6_B_ZN_579_ZN_8283
3eer_A_ZN_2001_ZN_1060
3eer_A_ZN_2002_ZN_1061
3eer_A_ZN_2003_ZN_1062
3eer_A_ZN_2004_ZN_1063
3eer_A_ZN_2005_ZN_1064
3eer_A_ZN_2006_ZN_1065
3ef7_A_ZN_484_ZN_7273
3ef7_B_ZN_487_ZN_7305
3ef7_B_ZN_488_ZN_7306
3ef7_B_ZN_489_ZN_7307
3ef7_B_ZN_491_ZN_7309
3ef7_A_ZN_485_ZN_7274
3ef7_A_ZN_486_ZN_7275
3ef7_A_ZN_489_ZN_7304
3ef7_A_ZN_490_ZN_7308
3ef7_B_ZN_483_ZN_7272
3ef7_B_ZN_484_ZN_7301
3ef7_B_ZN_485_ZN_7302
3ef7_B_ZN_486_ZN_7303
3ehv_A_ZN_101_ZN_1707
3eii_A_ZN_301_ZN_6401

3eii_B_ZN_301_ZN_6431
3eii_B_ZN_352_ZN_6456
3eii_C_ZN_301_ZN_6467
3eii_C_ZN_351_ZN_6493
3eii_D_ZN_301_ZN_6499
3eip_A_ZN_285_ZN_1389
3ekl_A_ZN_353_ZN_2741
3ekz_A_ZN_355_ZN_2612
3elf_A_ZN_353_ZN_2725
3enr_A_ZN_1005_ZN_3620
3enr_B_ZN_1006_ZN_3623
3eob_I_ZN_1_ZN_9459
3eob_J_ZN_2_ZN_9460
3epm_A_ZN_613_ZN_8063
3epm_B_ZN_613_ZN_8079
3eqn_A_ZN_756_ZN_11264
3eqn_A_ZN_757_ZN_11265
3eqn_A_ZN_758_ZN_11266
3eqn_B_ZN_756_ZN_11354
3eqn_B_ZN_757_ZN_11355
3eqn_B_ZN_758_ZN_11356
3eqo_A_ZN_756_ZN_11264
3eqo_B_ZN_756_ZN_11316
3erp_A_ZN_333_ZN_5562
3erp_B_ZN_333_ZN_5607
3erz_A_ZN_706_ZN_16847
3erz_B_ZN_708_ZN_16865
3erz_G_ZN_707_ZN_16904
3erz_H_ZN_709_ZN_16922
3erz_I_ZN_710_ZN_16932
3ewf_A_ZN_413_ZN_11540
3ewf_C_ZN_412_ZN_11547
3eyv_A_ZN_220_ZN_6745
3eyv_A_ZN_221_ZN_6746
3eyv_L_ZN_220_ZN_6697
3eyv_L_ZN_221_ZN_6698
3eyw_A_ZN_2402_ZN_5589
3eyw_B_ZN_2402_ZN_5590
3eyy_A_ZN_147_ZN_1964
3eyy_B_ZN_147_ZN_1991
3ez5_A_ZN_4_ZN_10194,3ez5_A_ZN_5_ZN_10195
3ez5_D_ZN_1_ZN_10258,3ez5_D_ZN_2_ZN_10259
3ez5_D_ZN_3_ZN_10260
3ez8_A_ZN_540_ZN_4080
3f3q_A_ZN_104_ZN_850
3f3q_A_ZN_105_ZN_851
3f3q_A_ZN_106_ZN_852
3f4v_A_ZN_1001_ZN_7621
3f5j_A_ZN_1001_ZN_7666
3f5k_A_ZN_1001_ZN_7677
3f5l_A_ZN_1001_ZN_7644
3f6g_A_ZN_359_ZN_1860
3f6g_B_ZN_358_ZN_1830
3f6h_B_ZN_358_ZN_1938
3f9o_A_ZN_310_ZN_1888
3fai_A_ZN_3_ZN_1886
3fav_A_ZN_101_ZN_2325
3fav_A_ZN_102_ZN_2326

3fav_B_ZN_1_ZN_2327
3fav_B_ZN_96_ZN_2328
3fav_C_ZN_101_ZN_2334
3fav_C_ZN_102_ZN_2335
3fav_D_ZN_96_ZN_2336
3fc2_A_ZN_8_ZN_2460
3fca_A_ZN_500_ZN_4379
3fca_B_ZN_500_ZN_4380
3fgg_A_ZN_161_ZN_2481
3fgg_A_ZN_162_ZN_2482
3fgg_A_ZN_163_ZN_2483
3fgg_A_ZN_164_ZN_2484
3fgg_B_ZN_161_ZN_2509
3fgg_B_ZN_162_ZN_2510
3fgg_B_ZN_163_ZN_2511
3fgg_B_ZN_164_ZN_2512
3fid_A_ZN_297_ZN_4591,3fid_A_ZN_298_ZN_4592,3fid_B_ZN_297_ZN_4682,3fid_B_ZN_298_ZN_4683
3fid_A_ZN_299_ZN_4593
3fid_A_ZN_300_ZN_4594
3fid_A_ZN_301_ZN_4595
3fid_B_ZN_299_ZN_4684
3fid_B_ZN_300_ZN_4685
3fju_A_ZN_501_ZN_2970
3fju_A_ZN_502_ZN_2971
3fju_A_ZN_503_ZN_2972
3fju_A_ZN_505_ZN_2973
3fju_A_ZN_506_ZN_2974,3fju_A_ZN_507_ZN_2975
3fju_A_ZN_508_ZN_2976
3fju_A_ZN_509_ZN_2977
3fju_B_ZN_504_ZN_3023
3fkg_A_ZN_332_ZN_2465
3fkg_A_ZN_333_ZN_2466
3fkg_A_ZN_334_ZN_2467,3fkg_A_ZN_338_ZN_2471
3fkg_A_ZN_336_ZN_2469
3fkg_A_ZN_337_ZN_2470,3fkg_A_ZN_339_ZN_2472
3fki_I_ZN_124_ZN_31421
3fm1_A_ZN_332_ZN_2479
3fm1_A_ZN_333_ZN_2480
3fm1_A_ZN_335_ZN_2482
3fm2_A_ZN_135_ZN_2090
3fm2_B_ZN_135_ZN_2099
3fm4_A_ZN_332_ZN_2413
3fm4_A_ZN_333_ZN_2414
3fm4_A_ZN_334_ZN_2415,3fm4_A_ZN_337_ZN_2418
3fm4_A_ZN_336_ZN_2417
3fm4_A_ZN_338_ZN_2419
3fm6_A_ZN_332_ZN_4771
3fm6_A_ZN_333_ZN_4772
3fm6_A_ZN_335_ZN_4774
3fm6_A_ZN_336_ZN_4775,3fm6_A_ZN_337_ZN_4776
3fmu_A_ZN_332_ZN_4865
3fmu_A_ZN_333_ZN_4866
3fmu_A_ZN_334_ZN_4867,3fmu_A_ZN_338_ZN_4871
3fmu_A_ZN_336_ZN_4869
3fmu_A_ZN_337_ZN_4870,3fmu_A_ZN_339_ZN_4872,3fmu_A_ZN_340_ZN_4873
3fns_A_ZN_330_ZN_5184
3fns_A_ZN_331_ZN_5185

3fns_A_ZN_332_ZN_5186
3fns_B_ZN_330_ZN_5188
3fp5_A_ZN_107_ZN_874
3fsr_A_ZN_354_ZN_10698
3fsr_B_ZN_353_ZN_10711
3fsr_C_ZN_355_ZN_10721
3fsr_D_ZN_353_ZN_10722
3fu1_A_ZN_201_ZN_1783
3fu1_B_ZN_1_ZN_1787
3fu1_B_ZN_201_ZN_1785
3fw6_A_ZN_1_ZN_4006
3fw6_A_ZN_2_ZN_4007
3fw6_A_ZN_3_ZN_4008
3fwx_A_ZN_170_ZN_2623
3fwx_B_ZN_170_ZN_2624
3fym_A_ZN_2001_ZN_702
3g04_A_ZN_304_ZN_5054
3g04_A_ZN_305_ZN_5055
3g04_C_ZN_301_ZN_5126
3g04_C_ZN_302_ZN_5127
3g04_C_ZN_303_ZN_5128
3g4h_A_ZN_300_ZN_4610
3g4h_B_ZN_300_ZN_4609
3g64_A_ZN_278_ZN_6412
3g64_A_ZN_279_ZN_6413
3g7l_A_ZN_2_ZN_1065,3g7l_A_ZN_76_ZN_1064
3g7l_A_ZN_3_ZN_1066
3g7l_A_ZN_4_ZN_1067
3g8r_A_ZN_501_ZN_5296
3g8r_B_ZN_501_ZN_5297
3gak_A_ZN_331_ZN_4517
3gc9_B_ZN_603_ZN_5425
3gdf_A_ZN_1003_ZN_7997
3gdf_A_ZN_1004_ZN_7998
3gdf_B_ZN_1001_ZN_7999
3gdf_B_ZN_1002_ZN_8000
3geh_A_ZN_462_ZN_3428
3geh_A_ZN_463_ZN_3429
3ggl_A_ZN_1_ZN_1245
3git_A_ZN_739_ZN_19757
3git_B_ZN_739_ZN_19798
3git_C_ZN_739_ZN_19834
3git_D_ZN_739_ZN_19875
3git_E_ZN_739_ZN_19906
3git_F_ZN_739_ZN_19942
3giu_A_ZN_213_ZN_3585
3giu_B_ZN_213_ZN_3628
3giz_L_ZN_212_ZN_3264
3giz_L_ZN_213_ZN_3265
3giz_L_ZN_214_ZN_3266
3gj9_A_ZN_125_ZN_1754
3gj9_B_ZN_125_ZN_1755
3gj9_B_ZN_126_ZN_1756
3gj9_B_ZN_127_ZN_1757
3gl6_A_ZN_1_ZN_469
3go9_A_ZN_601_ZN_3726
3go9_A_ZN_602_ZN_3727
3go9_A_ZN_604_ZN_3729

3go9_A_ZN_605_ZN_3730
3go9_A_ZN_606_ZN_3731
3gps_A_ZN_128_ZN_3865
3gps_D_ZN_128_ZN_3936
3gps_D_ZN_129_ZN_3937
3gps_D_ZN_130_ZN_3938
3gps_A_ZN_129_ZN_3866
3gps_A_ZN_130_ZN_3867
3gps_B_ZN_128_ZN_3882
3gps_B_ZN_129_ZN_3883
3gps_B_ZN_130_ZN_3884
3gps_C_ZN_128_ZN_3915
3gps_C_ZN_129_ZN_3916
3gps_C_ZN_130_ZN_3917
3grb_A_ZN_129_ZN_3795
3grb_D_ZN_128_ZN_3833
3grb_D_ZN_129_ZN_3834
3grb_D_ZN_133_ZN_3853
3grb_A_ZN_130_ZN_3796
3grb_A_ZN_134_ZN_3815
3grb_B_ZN_128_ZN_3816
3grb_B_ZN_129_ZN_3817
3grb_B_ZN_130_ZN_3818
3grb_C_ZN_128_ZN_3860
3grb_C_ZN_129_ZN_3861
3grb_C_ZN_133_ZN_3880
3grg_A_ZN_129_ZN_3778
3grg_D_ZN_128_ZN_3812
3grg_D_ZN_129_ZN_3813
3grg_D_ZN_130_ZN_3814
3grg_A_ZN_130_ZN_3779
3grg_A_ZN_131_ZN_3780
3grg_B_ZN_128_ZN_3795
3grg_B_ZN_129_ZN_3796
3grg_B_ZN_130_ZN_3797
3grg_C_ZN_128_ZN_3845
3grg_C_ZN_129_ZN_3846
3grg_C_ZN_130_ZN_3847
3gs2_A_ZN_334_ZN_2232
3gsh_A_ZN_101_ZN_1412,3gsh_B_ZN_104_ZN_1445
3gsh_A_ZN_102_ZN_1413
3gsh_A_ZN_103_ZN_1414
3gtl_A_ZN_1734_ZN_29261
3gtm_S_ZN_310_ZN_30574
3gto_A_ZN_1734_ZN_29264
3gtp_A_ZN_1734_ZN_29284
3gtq_A_ZN_1734_ZN_28630
3gug_A_ZN_324_ZN_2621
3gyy_A_ZN_317_ZN_9825
3gyy_B_ZN_319_ZN_9834
3gyy_B_ZN_320_ZN_9835
3gyy_B_ZN_321_ZN_9836
3gyy_B_ZN_322_ZN_9837,3gyy_B_ZN_329_ZN_9844
3gyy_B_ZN_323_ZN_9838
3gyy_B_ZN_324_ZN_9839
3gyy_B_ZN_325_ZN_9840
3gyy_B_ZN_326_ZN_9841
3gyy_B_ZN_327_ZN_9842

3gyy_B_ZN_328_ZN_9843
3gyy_A_ZN_318_ZN_9826
3gyy_B_ZN_330_ZN_9845
3gyy_C_ZN_317_ZN_9846
3gyy_C_ZN_318_ZN_9847
3gyy_C_ZN_319_ZN_9848
3gyy_C_ZN_320_ZN_9849
3gyy_C_ZN_321_ZN_9850
3gyy_C_ZN_322_ZN_9851
3gyy_C_ZN_323_ZN_9852
3gyy_D_ZN_317_ZN_9853
3gyy_D_ZN_318_ZN_9854
3gyy_A_ZN_320_ZN_9827
3gyy_D_ZN_319_ZN_9855
3gyy_D_ZN_320_ZN_9856
3gyy_D_ZN_321_ZN_9857
3gyy_D_ZN_322_ZN_9858
3gyy_D_ZN_323_ZN_9859
3gyy_D_ZN_324_ZN_9860
3gyy_A_ZN_321_ZN_9828
3gyy_A_ZN_322_ZN_9829
3gyy_A_ZN_323_ZN_9830
3gyy_A_ZN_324_ZN_9831
3gyy_B_ZN_317_ZN_9832
3gyy_B_ZN_318_ZN_9833
3gze_A_ZN_10_ZN_6458
3gze_C_ZN_3_ZN_6474
3gze_C_ZN_6_ZN_6475
3gze_C_ZN_9_ZN_6476
3gze_D_ZN_13_ZN_6483
3gze_D_ZN_4_ZN_6482
3gze_A_ZN_1_ZN_6454
3gze_A_ZN_5_ZN_6455
3gze_A_ZN_7_ZN_6456
3gze_A_ZN_8_ZN_6457
3gze_B_ZN_12_ZN_6464
3gze_B_ZN_14_ZN_6465
3gze_B_ZN_2_ZN_6463
3gze_C_ZN_11_ZN_6477
3gzk_A_ZN_539_ZN_4115
3gzu_C_ZN_398_ZN_54007
3gzu_G_ZN_398_ZN_54008
3gzu_J_ZN_398_ZN_54009
3gzu_N_ZN_398_ZN_54010
3gzu_O_ZN_398_ZN_54011
3h03_A_ZN_1_ZN_8064
3h03_A_ZN_262_ZN_8065
3h03_B_ZN_262_ZN_8086
3h03_B_ZN_3_ZN_8085
3h03_D_ZN_262_ZN_8107
3h03_D_ZN_2_ZN_8106
3h03_G_ZN_262_ZN_8127
3h03_G_ZN_263_ZN_8128
3h0g_O_ZN_1269_ZN_62890
3h0g_C_ZN_1269_ZN_62881
3h1k_C_ZN_2012_ZN_32080
3h1k_P_ZN_3012_ZN_32497
3h2p_A_ZN_154_ZN_1859

3h2p_A_ZN_155_ZN_1860
3h2p_B_ZN_154_ZN_1871
3h2q_A_ZN_154_ZN_3777
3h2q_B_ZN_154_ZN_3783
3h2q_C_ZN_154_ZN_3794
3h2q_D_ZN_154_ZN_3800
3h2r_A_ZN_154_ZN_1823
3h2r_B_ZN_154_ZN_1832
3h2w_A_ZN_539_ZN_4124
3h3k_A_ZN_539_ZN_4146
3h44_B_ZN_1_ZN_15789
3h44_C_ZN_42_ZN_15808
3h50_A_ZN_115_ZN_868
3h50_A_ZN_116_ZN_869
3h50_A_ZN_117_ZN_870
3h5f_A_ZN_32_ZN_746
3h5f_A_ZN_33_ZN_747
3h5f_C_ZN_32_ZN_750,3h5f_C_ZN_33_ZN_751
3h5g_A_ZN_31_ZN_726,3h5g_A_ZN_34_ZN_729,3h5g_A_ZN_35_ZN_730
3h5g_A_ZN_32_ZN_727
3h5g_A_ZN_33_ZN_728
3h5g_B_ZN_31_ZN_731
3h5g_C_ZN_31_ZN_732
3h66_A_ZN_500_ZN_5059,3h66_A_ZN_501_ZN_5060
3h66_B_ZN_500_ZN_5061,3h66_B_ZN_501_ZN_5062
3h67_A_ZN_500_ZN_5065,3h67_A_ZN_501_ZN_5066
3h67_D_ZN_500_ZN_5097,3h67_D_ZN_501_ZN_5098
3h68_A_ZN_500_ZN_5059,3h68_A_ZN_501_ZN_5060
3h68_D_ZN_500_ZN_5091,3h68_D_ZN_501_ZN_5092
3h69_A_ZN_500_ZN_5059,3h69_A_ZN_501_ZN_5060
3h69_D_ZN_500_ZN_5077,3h69_D_ZN_501_ZN_5078
3h6t_A_ZN_266_ZN_6181
3h6t_C_ZN_267_ZN_6301
3h6t_A_ZN_267_ZN_6182
3h6t_A_ZN_268_ZN_6183
3h6t_B_ZN_266_ZN_6238
3h6t_B_ZN_267_ZN_6239
3h6t_B_ZN_268_ZN_6240
3h6t_B_ZN_269_ZN_6241
3h6t_B_ZN_270_ZN_6242
3h6t_C_ZN_266_ZN_6300
3h7h_A_ZN_119_ZN_1757
3h7s_A_ZN_101_ZN_1219
3h7s_A_ZN_102_ZN_1220
3h7s_A_ZN_103_ZN_1221
3h7s_A_ZN_104_ZN_1222,3h7s_A_ZN_104_ZN_1223
3h7s_A_ZN_105_ZN_1224
3h7s_A_ZN_106_ZN_1225,3h7s_B_ZN_104_ZN_1229
3h7s_B_ZN_101_ZN_1226
3h7s_B_ZN_102_ZN_1227
3h7s_B_ZN_103_ZN_1228
3h7t_A_ZN_236_ZN_3543,3h7t_A_ZN_237_ZN_3544,3h7t_A_ZN_241_ZN_3548
3h7t_A_ZN_238_ZN_3545,3h7t_A_ZN_242_ZN_3549
3h7t_A_ZN_239_ZN_3546
3h7t_A_ZN_240_ZN_3547,3h7t_A_ZN_243_ZN_3550
3h7t_B_ZN_236_ZN_3551
3hbu_P_ZN_488_ZN_3560
3hbv_P_ZN_488_ZN_2940

3hc3_L_ZN_214_ZN_3353
3hc3_L_ZN_215_ZN_3354
3hc4_L_ZN_401_ZN_3447
3hc4_L_ZN_402_ZN_3448
3hcu_C_ZN_306_ZN_4069
3hff_A_ZN_154_ZN_821
3hff_A_ZN_155_ZN_822
3hff_A_ZN_156_ZN_823
3hff_A_ZN_158_ZN_824
3hff_A_ZN_159_ZN_825
3hft_A_ZN_257_ZN_2027
3hk5_A_ZN_431_ZN_6784
3hk5_B_ZN_431_ZN_6803
3hk8_A_ZN_431_ZN_6763
3hk8_B_ZN_431_ZN_6783
3hka_C_ZN_430_ZN_10220
3hm3_A_ZN_600_ZN_2543,3hm3_A_ZN_600_ZN_2544
3hm3_A_ZN_700_ZN_2545,3hm3_A_ZN_700_ZN_2546
3hm3_B_ZN_400_ZN_2547,3hm3_B_ZN_400_ZN_2548
3hm3_C_ZN_100_ZN_2549,3hm3_C_ZN_100_ZN_2550
3hm3_C_ZN_200_ZN_2551,3hm3_C_ZN_200_ZN_2552
3hm3_D_ZN_900_ZN_2553,3hm3_D_ZN_900_ZN_2554
3hmf_A_ZN_1_ZN_942
3hmf_A_ZN_2_ZN_943
3hn8_A_ZN_700_ZN_6661
3hn8_B_ZN_700_ZN_6668
3hn8_C_ZN_700_ZN_6675
3hni_A_ZN_107_ZN_6642
3hni_B_ZN_107_ZN_6686
3hni_C_ZN_107_ZN_6730
3hni_D_ZN_107_ZN_6774
3hni_E_ZN_107_ZN_6818
3hni_F_ZN_107_ZN_6862
3hni_G_ZN_107_ZN_6906
3hni_H_ZN_107_ZN_6950
3hnj_A_ZN_107_ZN_3360
3hnj_B_ZN_107_ZN_3404
3hnj_B_ZN_108_ZN_3405
3hnj_B_ZN_109_ZN_3406
3hnj_C_ZN_107_ZN_3450
3hnj_D_ZN_107_ZN_3494
3hnj_D_ZN_108_ZN_3495
3hnj_D_ZN_109_ZN_3496
3ho5_A_ZN_902_ZN_8258
3hpi_A_ZN_372_ZN_5747
3hpi_A_ZN_374_ZN_5775
3hpi_B_ZN_371_ZN_5778
3hpi_B_ZN_372_ZN_5779
3hpi_B_ZN_373_ZN_5776
3hpi_B_ZN_374_ZN_5777
3hq2_A_ZN_504_ZN_8167
3hq2_A_ZN_506_ZN_8170,3hq2_A_ZN_508_ZN_8172
3hq2_B_ZN_504_ZN_8180
3hq2_B_ZN_507_ZN_8182
3hqh_A_ZN_1_ZN_1133,3hqh_A_ZN_2_ZN_1134
3hqh_A_ZN_3_ZN_1135
3hru_A_ZN_216_ZN_3353
3hru_B_ZN_216_ZN_3354

3hsu_A_ZN_503_ZN_3832
3hsv_A_ZN_2_ZN_2330,3hsv_A_ZN_2_ZN_2331
3hsv_B_ZN_3_ZN_2332,3hsv_B_ZN_3_ZN_2333
3hsv_M_ZN_1_ZN_2339
3htr_A_ZN_118_ZN_1485
3htr_A_ZN_119_ZN_1486
3htr_B_ZN_118_ZN_1495
3htr_B_ZN_119_ZN_1496
3hxs_A_ZN_142_ZN_1938
3hxs_A_ZN_143_ZN_1939
3hyp_A_ZN_142_ZN_1910
3hyp_B_ZN_142_ZN_1911
3hzk_B_ZN_215_ZN_3392
3hzm_B_ZN_215_ZN_3412
3hzv_B_ZN_213_ZN_3427
3hzy_A_ZN_214_ZN_3347
3hzy_A_ZN_215_ZN_3348
3hzy_A_ZN_218_ZN_3346
3hzy_B_ZN_215_ZN_3398
3i31_A_ZN_1_ZN_583
3i3w_A_ZN_900_ZN_6762
3i3w_B_ZN_900_ZN_6763
3i8g_P_ZN_127_ZN_58444
3i8h_P_ZN_127_ZN_60632
3i9b_4_ZN_3_ZN_56867
3i9d_4_ZN_3_ZN_58416
3i9f_A_ZN_1_ZN_2721
3i9f_A_ZN_2_ZN_2722
3i9f_A_ZN_5_ZN_2723
3i9f_B_ZN_3_ZN_2724
3i9f_B_ZN_4_ZN_2725
3i9f_B_ZN_6_ZN_2726
3iab_B_ZN_141_ZN_3038
3iab_B_ZN_142_ZN_3039,3iab_B_ZN_143_ZN_3040
3iaz_A_ZN_787_ZN_2785
3iaz_A_ZN_788_ZN_2786
3ib0_A_ZN_688_ZN_2645
3ib0_A_ZN_689_ZN_2646
3ib1_A_ZN_302_ZN_2606
3ib1_A_ZN_303_ZN_2607
3ib2_A_ZN_4_ZN_2722
3ib2_A_ZN_692_ZN_2721
3ici_A_ZN_401_ZN_3021
3ici_B_ZN_401_ZN_3034
3idv_A_ZN_501_ZN_1859
3idv_A_ZN_502_ZN_1860
3ie4_A_ZN_108_ZN_1618
3ie4_A_ZN_200_ZN_1615
3ie4_A_ZN_201_ZN_1616
3ie4_A_ZN_202_ZN_1617
3ie4_B_ZN_200_ZN_1619
3ie4_B_ZN_201_ZN_1620
3ie9_A_ZN_108_ZN_813
3iea_A_ZN_108_ZN_813
3iet_A_ZN_215_ZN_6686
3iet_B_ZN_216_ZN_6687
3iet_C_ZN_218_ZN_6688
3iet_D_ZN_214_ZN_6689

3if1_A_ZN_213_ZN_6523
3if1_A_ZN_215_ZN_6524
3if1_A_ZN_222_ZN_6525
3if1_C_ZN_218_ZN_6543
3if1_D_ZN_221_ZN_6547
3ifj_A_ZN_201_ZN_2177
3ifj_B_ZN_202_ZN_2178
3ifu_A_ZN_181_ZN_1369
3igd_A_ZN_201_ZN_1092
3ii1_A_ZN_571_ZN_4039
3ii1_A_ZN_572_ZN_4040
3ii1_A_ZN_573_ZN_4041
3ijo_E_ZN_700_ZN_5974
3ijo_E_ZN_701_ZN_5975
3ijo_E_ZN_702_ZN_5976
3ijo_H_ZN_401_ZN_5978
3ijo_H_ZN_700_ZN_5977
3ijx_B_ZN_262_ZN_5934
3ijx_D_ZN_1_ZN_5979
3ijx_H_ZN_262_ZN_5982
3ijx_H_ZN_2_ZN_5980,3ijx_H_ZN_3_ZN_5981
3ik6_B_ZN_2_ZN_6080
3ik6_E_ZN_262_ZN_6082
3ik6_E_ZN_3_ZN_6081
3ik6_H_ZN_1_ZN_6100
3ik6_H_ZN_2_ZN_6686
3il1_B_ZN_263_ZN_6061
3il1_E_ZN_1_ZN_6062
3il1_E_ZN_262_ZN_6063
3il1_H_ZN_2_ZN_6064
3il1_H_ZN_3_ZN_6065
3ilt_B_ZN_1_ZN_5908
3ilt_B_ZN_2_ZN_5919
3ilt_E_ZN_262_ZN_5920
3ilt_H_ZN_262_ZN_5922
3ilt_H_ZN_3_ZN_5921
3ilu_B_ZN_262_ZN_6076
3ilu_E_ZN_1_ZN_6107
3ilu_E_ZN_262_ZN_6109
3ilu_E_ZN_3_ZN_6108
3ilu_H_ZN_2_ZN_6130
3im4_A_ZN_701_ZN_1027,3im4_A_ZN_705_ZN_1029
3im4_A_ZN_702_ZN_1028
3im4_B_ZN_703_ZN_1030
3im4_B_ZN_704_ZN_1031
3io4_A_ZN_450_ZN_9323
3io4_A_ZN_451_ZN_9324
3io4_A_ZN_452_ZN_9326
3io4_B_ZN_450_ZN_9321
3io4_B_ZN_451_ZN_9322
3io4_B_ZN_452_ZN_9328
3io4_C_ZN_450_ZN_9325
3io4_C_ZN_451_ZN_9327
3io6_A_ZN_450_ZN_9255
3io6_B_ZN_450_ZN_9256
3io6_B_ZN_451_ZN_9259
3io6_C_ZN_450_ZN_9257
3io6_C_ZN_451_ZN_9258

3io8_A_ZN_210_ZN_2691
3io8_C_ZN_210_ZN_2692
3io8_C_ZN_211_ZN_2693
3io9_A_ZN_1_ZN_1356
3io9_A_ZN_2_ZN_1357
3io9_A_ZN_3_ZN_1358
3io9_B_ZN_4_ZN_1359
3ior_A_ZN_450_ZN_9160
3ior_A_ZN_451_ZN_9163
3ior_B_ZN_450_ZN_9158
3ior_B_ZN_451_ZN_9159
3ior_B_ZN_452_ZN_9161
3ior_C_ZN_450_ZN_9162
3iot_A_ZN_450_ZN_9181
3iot_A_ZN_451_ZN_9183
3iot_A_ZN_452_ZN_9185
3iot_B_ZN_450_ZN_9182
3iot_B_ZN_451_ZN_9186
3iot_B_ZN_452_ZN_9189
3iot_C_ZN_445_ZN_9184
3iot_C_ZN_446_ZN_9187
3iot_C_ZN_447_ZN_9188
3iou_A_ZN_450_ZN_9083,3iou_A_ZN_453_ZN_9087
3iou_A_ZN_451_ZN_9084
3iou_A_ZN_452_ZN_9086
3iou_B_ZN_450_ZN_9080
3iou_B_ZN_451_ZN_9081
3iou_B_ZN_452_ZN_9082
3iou_C_ZN_450_ZN_9085
3iov_A_ZN_442_ZN_9205
3iov_A_ZN_443_ZN_9206
3iov_B_ZN_450_ZN_9202
3iov_B_ZN_451_ZN_9203
3iov_B_ZN_452_ZN_9204
3iov_C_ZN_428_ZN_9207
3iow_A_ZN_449_ZN_9267
3iow_A_ZN_450_ZN_9268
3iow_A_ZN_451_ZN_9273
3iow_B_ZN_450_ZN_9269
3iow_B_ZN_451_ZN_9270
3iow_C_ZN_450_ZN_9271
3iow_C_ZN_451_ZN_9272
3ipj_B_ZN_96_ZN_1511
3ipj_B_ZN_97_ZN_1512
3iq6_A_ZN_201_ZN_6596
3iq6_B_ZN_201_ZN_6640
3iq6_C_ZN_201_ZN_6684
3iq6_D_ZN_201_ZN_6728
3iq6_E_ZN_201_ZN_6772
3iq6_F_ZN_201_ZN_6816
3iq6_G_ZN_201_ZN_6860
3iq6_H_ZN_201_ZN_6904
3ir2_A_ZN_1101_ZN_2990
3ir2_B_ZN_1101_ZN_2994
3iso_A_ZN_220_ZN_3563
3iso_B_ZN_220_ZN_3609
3iu6_A_ZN_950_ZN_1232
3ivb_A_ZN_1_ZN_1208,3ivb_A_ZN_4_ZN_1211,3ivb_A_ZN_5_ZN_1212

3ivb_A_ZN_2_ZN_1209
3ivb_A_ZN_3_ZN_1210
3iz0_A_ZN_900_ZN_12037
3jpy_A_ZN_901_ZN_2642
3jpy_A_ZN_902_ZN_2643
3jpy_A_ZN_903_ZN_2644
3jpy_A_ZN_904_ZN_2645
3jpy_A_ZN_905_ZN_2646
3k0v_A_ZN_81_ZN_2751
3k0v_A_ZN_82_ZN_2752
3k6d_A_ZN_201_ZN_762
3k6i_A_ZN_201_ZN_762
3k6i_A_ZN_202_ZN_763
3k6j_A_ZN_800_ZN_3458
3k7a_A_ZN_1734_ZN_29032
3k7a_M_ZN_346_ZN_29040
3k7l_A_ZN_704_ZN_3223
3kao_A_ZN_329_ZN_2772
3kao_A_ZN_330_ZN_2773
3kao_A_ZN_331_ZN_2774
3kb7_A_ZN_346_ZN_2388
3kdk_A_ZN_2_ZN_3046
3kdk_B_ZN_4_ZN_3048
3kfl_A_ZN_804_ZN_4326
3kiq_m_ZN_608_ZN_55998
3kis_m_ZN_608_ZN_55992
3kiu_m_ZN_127_ZN_56008
3kix_m_ZN_608_ZN_56008
3kj1_A_ZN_1_ZN_1427
3kj1_A_ZN_2_ZN_1428
3kj1_A_ZN_3_ZN_1429
3kj1_A_ZN_5_ZN_1430
3kj1_B_ZN_23_ZN_1436
3kj2_A_ZN_2_ZN_1412
3kj2_A_ZN_3_ZN_1413
3kj2_A_ZN_4_ZN_1414
3kj2_B_ZN_23_ZN_1419
3kj4_C_ZN_221_ZN_11152
3kj4_H_ZN_221_ZN_11151
3kj7_A_ZN_81_ZN_2751
3kj7_A_ZN_82_ZN_2752
3kjh_A_ZN_255_ZN_1961
3kji_A_ZN_255_ZN_3921
3kji_B_ZN_255_ZN_3949
3kl7_A_ZN_302_ZN_1762,3kl7_A_ZN_303_ZN_1763
3km1_A_ZN_155_ZN_1781
3km1_B_ZN_155_ZN_1788
3kve_A_ZN_489_ZN_15534
3kve_B_ZN_489_ZN_15602
3kve_C_ZN_489_ZN_15670
3kve_D_ZN_489_ZN_15738
3kwo_A_ZN_156_ZN_4875,3kwo_C_ZN_161_ZN_4931,3kwo_C_ZN_162_ZN_4932
3kwo_B_ZN_165_ZN_4912
3kwo_B_ZN_166_ZN_4913
3kwo_B_ZN_167_ZN_4914
3kwo_C_ZN_165_ZN_4934
3kwo_C_ZN_166_ZN_4935
3kwo_D_ZN_161_ZN_4976,3kwo_D_ZN_162_ZN_4977,3kwo_D_ZN_163_ZN_4978

3kwo_D_ZN_164_ZN_4979
3kwo_D_ZN_165_ZN_4980
3kwo_D_ZN_166_ZN_4981
3kwo_A_ZN_161_ZN_4872,3kwo_A_ZN_162_ZN_4873,3kwo_C_ZN_164_ZN_4933
3kwo_A_ZN_163_ZN_4874
3kwo_A_ZN_164_ZN_4876
3kwo_A_ZN_165_ZN_4877
3kwo_A_ZN_167_ZN_4878
3kwo_A_ZN_168_ZN_4879
3kwo_B_ZN_161_ZN_4908,3kwo_B_ZN_162_ZN_4909,3kwo_B_ZN_163_ZN_4910
3kwo_B_ZN_164_ZN_4911
3kya_A_ZN_22_ZN_3985
3kya_A_ZN_519_ZN_3986
3kya_A_ZN_520_ZN_3987
3kya_A_ZN_521_ZN_3988,3kya_A_ZN_522_ZN_3989
3kya_A_ZN_523_ZN_3990
3kya_A_ZN_524_ZN_3991
3kya_A_ZN_525_ZN_3992
3kya_A_ZN_526_ZN_3993
3kz0_A_ZN_1_ZN_2676
3kz0_A_ZN_2_ZN_2678
3kz0_A_ZN_4_ZN_2677
3kz0_B_ZN_1_ZN_2687
3kz0_B_ZN_2_ZN_2684
3kz0_B_ZN_3_ZN_2685
3kz0_B_ZN_5_ZN_2686
3kz4_C_ZN_398_ZN_54120
3kz4_F_ZN_398_ZN_54121
3kz4_I_ZN_398_ZN_54122
3kz4_L_ZN_398_ZN_54123
3kz4_O_ZN_398_ZN_54124
3l1o_L_ZN_215_ZN_3354
3l22_A_ZN_1_ZN_3481
3l22_A_ZN_2_ZN_3482
3l22_A_ZN_3_ZN_3483
3l22_A_ZN_4_ZN_3484
3l22_A_ZN_5_ZN_3485,3l22_A_ZN_5_ZN_3486
3l22_A_ZN_6_ZN_3487
3l22_A_ZN_7_ZN_3488
3l22_A_ZN_8_ZN_3489
3l22_A_ZN_9_ZN_3490
3l4k_A_ZN_1_ZN_6986
3l4k_A_ZN_3_ZN_6987,3l4k_A_ZN_4_ZN_6988
3l4k_A_ZN_5_ZN_6989
3l4k_A_ZN_6_ZN_6990
3l4k_A_ZN_7_ZN_6991
3l4k_A_ZN_8_ZN_6992
3l4k_E_ZN_16_ZN_7013
3l53_A_ZN_225_ZN_10085,3l53_A_ZN_227_ZN_10087
3l53_F_ZN_225_ZN_10188
3l53_F_ZN_226_ZN_10189
3l53_F_ZN_227_ZN_10190
3l53_A_ZN_226_ZN_10086
3l53_B_ZN_225_ZN_10102
3l53_B_ZN_226_ZN_10103
3l53_C_ZN_225_ZN_10118
3l53_D_ZN_225_ZN_10145
3l53_D_ZN_226_ZN_10146

3l53_D_ZN_227_ZN_10147
3l53_E_ZN_225_ZN_10173
3l6s_A_ZN_486_ZN_3587,3l6s_A_ZN_487_ZN_3588
3l8y_A_ZN_301_ZN_1385
3l9w_A_ZN_2402_ZN_5617
3l9w_B_ZN_2402_ZN_5618
3l9x_A_ZN_2402_ZN_5432
3l9x_B_ZN_2402_ZN_5467
3lat_B_ZN_220_ZN_3440,3lat_B_ZN_221_ZN_3441
3lat_A_ZN_217_ZN_3419
3lat_A_ZN_218_ZN_3420
3lat_A_ZN_219_ZN_3421
3lat_A_ZN_220_ZN_3422,3lat_A_ZN_221_ZN_3423
3lat_B_ZN_217_ZN_3437
3lat_B_ZN_218_ZN_3438
3lat_B_ZN_219_ZN_3439
3lec_A_ZN_228_ZN_1766
3ley_H_ZN_222_ZN_3406
3ley_H_ZN_223_ZN_3407
3ley_L_ZN_233_ZN_3408
3lgb_B_ZN_515_ZN_3203
3lgb_B_ZN_516_ZN_3204
3li2_A_ZN_1_ZN_4686
3li2_A_ZN_2_ZN_4687
3li2_A_ZN_3_ZN_4688
3li2_A_ZN_4_ZN_4689
3lix_A_ZN_117_ZN_1777
3liz_A_ZN_334_ZN_5963,3liz_L_ZN_212_ZN_5991
3liz_A_ZN_335_ZN_5964
3liz_A_ZN_336_ZN_5965
3liz_A_ZN_337_ZN_5966
3liz_L_ZN_213_ZN_5992
3ljm_A_ZN_31_ZN_832
3ljm_A_ZN_32_ZN_833
3ljm_B_ZN_31_ZN_842
3ljm_B_ZN_32_ZN_843
3ljm_B_ZN_33_ZN_844
3lkv_A_ZN_401_ZN_2187
3lkv_A_ZN_402_ZN_2188
3lkv_A_ZN_403_ZN_2189
3llx_A_ZN_376_ZN_3199
3lpe_B_ZN_60_ZN_4401
3lpe_D_ZN_60_ZN_4402
3lpe_F_ZN_60_ZN_4403
3lpe_H_ZN_60_ZN_4404
3ls1_A_ZN_1_ZN_2073
3ls1_A_ZN_3_ZN_2074
3ls1_B_ZN_2_ZN_2075
3ls1_B_ZN_4_ZN_2076
3ls6_A_ZN_303_ZN_3085
3ls6_B_ZN_314_ZN_3112
3ls6_A_ZN_308_ZN_3086
3ls6_A_ZN_309_ZN_3087
3ls6_A_ZN_311_ZN_3088
3ls6_B_ZN_304_ZN_3106
3ls6_B_ZN_305_ZN_3107
3ls6_B_ZN_306_ZN_3108
3ls6_B_ZN_307_ZN_3109,3ls6_B_ZN_313_ZN_3111

3ls6_B_ZN_310_ZN_3110
3lsf_E_ZN_1_ZN_6171
3lsf_E_ZN_3_ZN_6172
3lsf_E_ZN_401_ZN_6173
3lsf_H_ZN_2_ZN_6174
3lsf_H_ZN_401_ZN_6175
3lsl_A_ZN_1_ZN_6205
3lsl_A_ZN_2_ZN_6206
3lsl_D_ZN_3_ZN_6207
3lsl_D_ZN_400_ZN_6208
3lsl_G_ZN_401_ZN_6209
3lsw_A_ZN_1_ZN_2080
3lsw_A_ZN_2_ZN_2081
3lt8_A_ZN_80_ZN_585
3lt9_A_ZN_80_ZN_585
3lta_A_ZN_80_ZN_584
3ltb_A_ZN_80_ZN_585
3ltc_A_ZN_80_ZN_601
3ltd_A_ZN_81_ZN_592
3lu0_D_ZN_1409_ZN_25414
3lu2_A_ZN_309_ZN_4971,3lu2_A_ZN_310_ZN_4972
3lu2_B_ZN_309_ZN_4973,3lu2_B_ZN_310_ZN_4974
3luo_A_ZN_159_ZN_1163
3luo_A_ZN_160_ZN_1164
3luo_A_ZN_161_ZN_1165
3luo_A_ZN_162_ZN_1166
3luu_A_ZN_101_ZN_725
3lve_A_ZN_200_ZN_893
3lwo_B_ZN_61_ZN_5401
3lwp_B_ZN_61_ZN_5400
3lwr_B_ZN_61_ZN_5380
3lwv_B_ZN_61_ZN_5357
3lzn_A_ZN_200_ZN_2501
3lzn_B_ZN_200_ZN_2507
3m1v_A_ZN_558_ZN_20379
3m2r_A_ZN_558_ZN_20208
3m2u_A_ZN_560_ZN_20098
3m2v_D_ZN_556_ZN_20005
3m30_A_ZN_558_ZN_20189
3m32_A_ZN_558_ZN_20048
3m3f_A_ZN_401_ZN_2097
3m3f_A_ZN_402_ZN_2098
3m3k_A_ZN_401_ZN_6129
3m3k_A_ZN_402_ZN_6130
3m3k_C_ZN_401_ZN_6131
3m3k_E_ZN_401_ZN_6132
3m3l_A_ZN_262_ZN_6120
3m3l_D_ZN_1_ZN_6131
3m3l_D_ZN_262_ZN_6133
3m3l_D_ZN_2_ZN_6132
3m3l_G_ZN_3_ZN_6170
3m4g_A_ZN_83_ZN_6575
3m4g_H_ZN_83_ZN_6584
3m4g_I_ZN_83_ZN_6585
3m4g_J_ZN_83_ZN_6586
3m4g_K_ZN_83_ZN_6587
3m4g_L_ZN_83_ZN_6588
3m4g_B_ZN_83_ZN_6576

3m4g_B_ZN_84_ZN_6577
3m4g_C_ZN_83_ZN_6578
3m4g_D_ZN_83_ZN_6579
3m4g_D_ZN_84_ZN_6580
3m4g_E_ZN_83_ZN_6581
3m4g_F_ZN_83_ZN_6582
3m4g_G_ZN_83_ZN_6583
3m4w_A_ZN_1_ZN_10117
3m4w_A_ZN_5_ZN_10118
3m4w_B_ZN_2_ZN_10119
3m4w_B_ZN_6_ZN_10120
3m4w_E_ZN_4_ZN_10121
3m4w_F_ZN_3_ZN_10122
3m52_A_ZN_116_ZN_1787
3m52_A_ZN_117_ZN_1788
3m52_A_ZN_118_ZN_1789
3m52_A_ZN_119_ZN_1790
3m52_B_ZN_116_ZN_1799
3m52_B_ZN_117_ZN_1800
3m79_A_ZN_107_ZN_6596
3m79_B_ZN_107_ZN_6640
3m79_C_ZN_107_ZN_6684
3m79_D_ZN_107_ZN_6728
3m79_E_ZN_107_ZN_6772
3m79_F_ZN_107_ZN_6816
3m79_G_ZN_107_ZN_6860
3m79_H_ZN_107_ZN_6904
3m7p_A_ZN_953_ZN_2437
3m7p_A_ZN_954_ZN_2438
3m7p_A_ZN_955_ZN_2443
3m7p_A_ZN_956_ZN_2439
3m7p_A_ZN_957_ZN_2440
3m7p_A_ZN_958_ZN_2441
3m7p_A_ZN_959_ZN_2442
3m97_X_ZN_142_ZN_911
3m97_X_ZN_143_ZN_912
3m97_X_ZN_144_ZN_913
3m97_X_ZN_145_ZN_914
3m9g_A_ZN_2000_ZN_1574
3m9g_A_ZN_2001_ZN_1575
3m9g_A_ZN_2002_ZN_1576
3m9g_A_ZN_2003_ZN_1577
3m9g_A_ZN_2004_ZN_1578
3m9g_A_ZN_2005_ZN_1579
3m9g_A_ZN_2006_ZN_1580
3mbg_A_ZN_1_ZN_2878
3mbg_C_ZN_4_ZN_3033
3mbg_A_ZN_208_ZN_2876
3mbg_A_ZN_2_ZN_2874
3mbg_A_ZN_3_ZN_2875
3mbg_B_ZN_1_ZN_3009
3mbg_B_ZN_206_ZN_3018
3mbg_B_ZN_2_ZN_3019
3mbg_C_ZN_1_ZN_2994
3mbg_C_ZN_2_ZN_3024
3mcx_A_ZN_1_ZN_3761
3mcx_A_ZN_2_ZN_3762
3mcx_A_ZN_3_ZN_3763

3mcx_A_ZN_4_ZN_3764
3mcx_A_ZN_5_ZN_3765
3mcx_A_ZN_6_ZN_3766
3mhx_A_ZN_2002_ZN_1281
3mhx_A_ZN_2007_ZN_1283
3mhx_A_ZN_2008_ZN_1284
3mhx_A_ZN_2009_ZN_1285
3mit_A_ZN_903_ZN_2076
3mit_B_ZN_902_ZN_2137
3miu_A_ZN_142_ZN_2108
3miu_B_ZN_142_ZN_2164
3miu_B_ZN_143_ZN_2165
3mjn_A_ZN_1003_ZN_2706
3mjn_A_ZN_1004_ZN_2707
3mpv_A_ZN_901_ZN_2949
3mpv_A_ZN_902_ZN_2950
3mpv_A_ZN_903_ZN_2951
3mpv_B_ZN_904_ZN_2952
3mpv_B_ZN_905_ZN_2953
3msu_A_ZN_431_ZN_6903
3msu_B_ZN_434_ZN_6932
3mwp_A_ZN_570_ZN_12003
3mwp_B_ZN_570_ZN_12004
3mwp_C_ZN_570_ZN_12005
3mwt_A_ZN_690_ZN_11936
3mwt_B_ZN_690_ZN_11938
3mwt_C_ZN_690_ZN_11940
3mx2_A_ZN_570_ZN_12106
3mx2_B_ZN_570_ZN_12136
3mx2_C_ZN_570_ZN_12166
3mx5_A_ZN_570_ZN_12104
3mx5_B_ZN_570_ZN_12134
3mx5_C_ZN_570_ZN_12135
3mz8_A_ZN_500_ZN_3479
3mz8_B_ZN_501_ZN_3480
3n09_C_ZN_398_ZN_54059
3n09_F_ZN_398_ZN_54060
3n09_K_ZN_398_ZN_54061
3n09_N_ZN_398_ZN_54062
3n09_O_ZN_398_ZN_54063
3n0i_A_ZN_10_ZN_4377
3n0i_B_ZN_10_ZN_4466
3n0i_C_ZN_10_ZN_4467
3n12_A_ZN_1_ZN_2560
3n12_A_ZN_2_ZN_2561
3n12_A_ZN_3_ZN_2562,3n12_A_ZN_4_ZN_2563
3n3u_A_ZN_401_ZN_2295
3n3u_A_ZN_402_ZN_2296
3n55_A_ZN_503_ZN_970
3n6w_A_ZN_401_ZN_2968
3na9_H_ZN_218_ZN_3425
3na9_H_ZN_219_ZN_3426
3na9_H_ZN_220_ZN_3427
3na9_H_ZN_221_ZN_3428
3na9_L_ZN_215_ZN_3414
3na9_L_ZN_216_ZN_3415
3na9_L_ZN_217_ZN_3416
3naa_H_ZN_218_ZN_3398

3naa_H_ZN_219_ZN_3399
3naa_L_ZN_215_ZN_3387
3nab_H_ZN_215_ZN_3292
3nab_H_ZN_216_ZN_3293
3nab_L_ZN_215_ZN_3286
3nab_L_ZN_216_ZN_3287
3nac_H_ZN_218_ZN_3420
3nac_H_ZN_219_ZN_3421
3nac_H_ZN_220_ZN_3422
3nac_H_ZN_221_ZN_3423
3nac_H_ZN_222_ZN_3424
3nac_L_ZN_215_ZN_3408
3nac_L_ZN_216_ZN_3409
3nat_A_ZN_165_ZN_2455
3ncj_H_ZN_218_ZN_3405
3ncj_H_ZN_219_ZN_3406
3ncj_L_ZN_215_ZN_3393
3ncj_L_ZN_216_ZN_3394
3ngj_A_ZN_250_ZN_6485
3ngj_A_ZN_251_ZN_6486
3ngj_B_ZN_250_ZN_6488
3ngj_B_ZN_251_ZN_6489
3ngj_C_ZN_250_ZN_6495
3ngj_C_ZN_251_ZN_6496
3ngj_D_ZN_250_ZN_6506
3ngj_D_ZN_251_ZN_6507
3nm8_A_ZN_304_ZN_4695
3nm8_A_ZN_503_ZN_4690
3nm8_A_ZN_504_ZN_4691
3nm8_A_ZN_505_ZN_4692
3nm8_B_ZN_503_ZN_4698
3nm8_B_ZN_504_ZN_4699
3nm8_B_ZN_506_ZN_4700
3nmk_B_ZN_152_ZN_3319
3nmk_C_ZN_152_ZN_3320
3npy_A_ZN_505_ZN_4644
3npy_A_ZN_506_ZN_4645
3npy_A_ZN_507_ZN_4646
3npy_A_ZN_508_ZN_4647
3npy_A_ZN_509_ZN_4648
3npy_B_ZN_505_ZN_4657
3npy_B_ZN_506_ZN_4658
3npy_B_ZN_507_ZN_4746
3nq1_A_ZN_503_ZN_4647
3nq1_B_ZN_504_ZN_4678
3nq1_B_ZN_505_ZN_4679
3nq1_B_ZN_506_ZN_4680
3nq1_B_ZN_507_ZN_4681
3nq1_A_ZN_504_ZN_4658
3nq1_A_ZN_505_ZN_4659
3nq1_A_ZN_506_ZN_4660
3nq1_A_ZN_507_ZN_4661
3nq1_A_ZN_508_ZN_4662
3nq1_A_ZN_509_ZN_4663
3nq1_A_ZN_510_ZN_4664
3nq1_B_ZN_503_ZN_4667
3nq5_A_ZN_503_ZN_4652
3nq5_B_ZN_506_ZN_4663

3nq5_A_ZN_504_ZN_4653
3nq5_A_ZN_505_ZN_4654
3nq5_A_ZN_506_ZN_4655
3nq5_A_ZN_507_ZN_4656
3nq5_A_ZN_508_ZN_4657
3nq5_B_ZN_503_ZN_4660
3nq5_B_ZN_504_ZN_4661
3nq5_B_ZN_505_ZN_4662
3nyh_A_ZN_628_ZN_4969
3o14_A_ZN_250_ZN_3505
3o14_B_ZN_250_ZN_3565
3o2g_A_ZN_388_ZN_3194
3o2g_A_ZN_389_ZN_3195
3o97_A_ZN_302_ZN_2689
3o97_A_ZN_303_ZN_2690
3obc_A_ZN_107_ZN_1854
3obc_B_ZN_108_ZN_1856
3oca_A_ZN_300_ZN_2779
3oca_B_ZN_300_ZN_2782
3ofq_4_ZN_781_ZN_89822
3ogw_A_ZN_617_ZN_4777
3ol7_A_ZN_2001_ZN_17845
3ol7_E_ZN_2002_ZN_17886
3ome_A_ZN_300_ZN_11244
3ome_B_ZN_300_ZN_11245
3ome_C_ZN_300_ZN_11246
3ome_D_ZN_300_ZN_11247
3ome_E_ZN_300_ZN_11248
3ome_F_ZN_300_ZN_11249
3orj_A_ZN_468_ZN_3286
3p2a_A_ZN_151_ZN_4463
3p2a_B_ZN_151_ZN_4467
3p2a_C_ZN_151_ZN_4468
3p2a_D_ZN_151_ZN_4469
3p3e_A_ZN_401_ZN_4637
3pa0_C_ZN_12_ZN_831
3pa0_D_ZN_12_ZN_836
3pd8_A_ZN_260_ZN_6100
3pd8_B_ZN_260_ZN_6124
3pd8_B_ZN_261_ZN_6125
3pd8_C_ZN_260_ZN_6139
3pd8_C_ZN_261_ZN_6140
3pe8_A_ZN_254_ZN_1651,3pe8_A_ZN_254_ZN_1652
3pfe_A_ZN_503_ZN_3974
3pfe_A_ZN_504_ZN_3975
3pfs_A_ZN_1200_ZN_2096
3pfs_B_ZN_1200_ZN_2102
3plq_A_ZN_302_ZN_1015
3plw_A_ZN_187_ZN_926
3plw_A_ZN_188_ZN_927
3pm6_A_ZN_319_ZN_4136
3pm6_B_ZN_320_ZN_4154
3pso_A_ZN_601_ZN_2895,3pso_A_ZN_602_ZN_2896
3pso_A_ZN_603_ZN_2897
3pso_A_ZN_604_ZN_2898
3pso_B_ZN_701_ZN_2899,3pso_B_ZN_702_ZN_2900
3pso_B_ZN_703_ZN_2901
3pw3_A_ZN_406_ZN_17339

3pw3_B_ZN_406_ZN_17367
3pw3_C_ZN_406_ZN_17386
3pw3_D_ZN_406_ZN_17397
3pw3_E_ZN_406_ZN_17405
3pw3_F_ZN_406_ZN_17411
4enl_A_ZN_438_ZN_3291
4kmb_1_ZN_7_ZN_3528
5lve_A_ZN_200_ZN_885
6enl_A_ZN_438_ZN_3300
7ico_A_ZN_340_ZN_2875
7ico_A_ZN_343_ZN_2878
7icp_A_ZN_340_ZN_2875
7icp_A_ZN_343_ZN_2878
7icq_A_ZN_343_ZN_2877
7icr_A_ZN_340_ZN_2875
7icr_A_ZN_343_ZN_2878
7ict_A_ZN_343_ZN_2877
7mdh_A_ZN_501_ZN_10855
7mdh_C_ZN_501_ZN_10864
7mdh_C_ZN_502_ZN_10865
7mdh_C_ZN_503_ZN_10866
7mdh_C_ZN_504_ZN_10867
7mdh_C_ZN_505_ZN_10868
7mdh_D_ZN_501_ZN_10869
7mdh_D_ZN_502_ZN_10870
7mdh_D_ZN_503_ZN_10871
7mdh_A_ZN_502_ZN_10856
7mdh_A_ZN_503_ZN_10857
7mdh_A_ZN_504_ZN_10858
7mdh_B_ZN_501_ZN_10859
7mdh_B_ZN_502_ZN_10860
7mdh_B_ZN_503_ZN_10861
7mdh_B_ZN_504_ZN_10862
7mdh_B_ZN_505_ZN_10863
8rnt_A_ZN_105_ZN_779
9icf_A_ZN_339_ZN_2926
9icf_A_ZN_343_ZN_2929
9icg_A_ZN_339_ZN_2916,9icg_A_ZN_340_ZN_2917
9icg_A_ZN_343_ZN_2920
9ich_A_ZN_339_ZN_2916,9ich_A_ZN_340_ZN_2917
9ich_A_ZN_343_ZN_2920
9ici_A_ZN_340_ZN_2920
9ici_A_ZN_343_ZN_2923
9icv_A_ZN_339_ZN_2920,9icv_A_ZN_340_ZN_2921
9icv_A_ZN_343_ZN_2924
