# Supplementary material for: Minimal Functional Sites Allow a Classification of Zinc Sites in Proteins
Source: PLoS One. 2011 Oct 17;6(10):e26325. doi: 10.1371/journal.pone.0026325 (PMC3197139; doi:10.1371/journal.pone.0026325)
Supplement: Table S2 — Summary of the relevant information on Zn-superfamilies. (PDF) [file pone.0026325.s002.pdf]

Table S2. Summary of the relevant information on Zn-superfamilies.

Zn-superfamily	Cluster	Zinc function	# of Zn-sites	# of non-redundant proteins	Representative Zn-site
SF_1	Zinc Ribbons	Structural	42	30	3fl2_A_ZN_1002_ZN_910
SF_2	Zinc Ribbons	Structural	137	18	2hf1_A_ZN_102_ZN_928
SF_3	Zinc Ribbons	Structural	79	14	1yc5_A_ZN_1001_ZN_1944
SF_4	Zinc Ribbons	Structural	23	12	2con_A_ZN_201_ZN_1182
SF_5	Zinc Ribbons	Structural	14	10	1nj3_A_ZN_32_ZN_439
SF_6	Zinc Ribbons	Structural	38	8	1a8h_A_ZN_1000_ZN_4103
SF_7	Zinc Ribbons	Structural	9	7	1x4j_A_ZN_401_ZN_1123
SF_8	Zinc Ribbons	Structural	120	6	1twf_A_ZN_3006_ZN_28295
SF_9	Zinc Ribbons	Structural	12	6	1wge_A_ZN_201_ZN_1180
SF_10	Zinc Ribbons	Structural	27	6	2gag_D_ZN_101_ZN_12661
SF_11	Zinc Ribbons	Structural	35	5	1hxr_A_ZN_200_ZN_1679
SF_12	Zinc Ribbons	Structural	47	5	1k3x_A_ZN_501_ZN_2464
SF_13	Zinc Ribbons	Structural	26	5	1m2o_A_ZN_800_ZN_13889
SF_14	Zinc Ribbons	Structural	70	5	1twf_L_ZN_3005_ZN_28294
SF_15	Zinc Ribbons	Unknown	11	5	2kv1_A_ZN_125_ZN_1878
SF_16	Zinc Ribbons	Structural	6	5	2yu4_A_ZN_201_ZN_1482
SF_17	Zinc Ribbons	Structural	7	4	1vk6_A_ZN_301_ZN_2074
SF_18	Zinc Ribbons	Structural	30	4	1zin_A_ZN_219_ZN_1689
SF_19	Zinc Ribbons	Structural	5	4	2ckl_B_ZN_1115_ZN_1568
SF_20	Zinc Ribbons	Structural	4	4	2ea6_A_ZN_401_ZN_997
SF_21	Zinc Ribbons	Structural	16	4	3gj3_B_ZN_300_ZN_1856
SF_22	Zinc Ribbons	Structural	4	3	1k81_A_ZN_144_ZN_587
SF_23	Zinc Ribbons	Structural	108	3	2air_B_ZN_800_ZN_7255
SF_24	Zinc Ribbons	Structural	10	3	2ayj_A_ZN_57_ZN_956
SF_25	Zinc Ribbons	Structural	157	2	1d0c_A_ZN_900_ZN_6606
SF_26	Zinc Ribbons	Structural	5	2	1dgs_B_ZN_2701_ZN_9412
SF_27	Zinc Ribbons	Unknown	3	2	2f9y_B_ZN_305_ZN_4261
SF_28	Zinc Ribbons	Unknown	7	2	2i1o_A_ZN_701_ZN_3043-2i1o_A_ZN_702_ZN_3044
SF_29	Zinc Ribbons	Structural	2	2	2kkr_A_ZN_500_ZN_987
SF_30	Zinc Ribbons	Structural	1	1	1fre_A_ZN_43_ZN_315
SF_31	Zinc Ribbons	Structural	1	1	1odh_A_ZN_1172_ZN_1809
SF_32	Zinc Ribbons	Structural	2	1	1p91_A_ZN_1301_ZN_4179
SF_33	Zinc Ribbons	Structural	12	1	1pfv_A_ZN_552_ZN_4392
SF_34	Zinc Ribbons	Structural	16	1	1qf8_A_ZN_216_ZN_2707
SF_35	Zinc Ribbons	Unknown	1	1	1t8h_A_ZN_275_ZN_2098
SF_36	Zinc Ribbons	Structural	2	1	1zt2_A_ZN_999_ZN_8595
SF_37	Zinc Ribbons	Structural	26	1	2a6h_D_ZN_7458_ZN_53474
SF_38	Zinc Ribbons	Structural	9	1	2apo_B_ZN_501_ZN_2846
SF_39	Zinc Ribbons	Structural	9	1	2ba1_A_ZN_201_ZN_15664
SF_40	Zinc Ribbons	Structural	1	1	2d8s_A_ZN_401_ZN_1148

SF_41	Zinc Ribbons	Structural	1	1	2dkt_A_ZN_291_ZN_2166
SF_42	Zinc Ribbons	Unknown	1	1	2exu_A_ZN_501_ZN_1505
SF_43	Zinc Ribbons	Structural	2	1	2hdp_A_ZN_493_ZN_1998
SF_44	Zinc Ribbons	Structural	2	1	2hu9_A_ZN_132_ZN_2069
SF_45	Zinc Ribbons	Unknown	4	1	2jne_A_ZN_150_ZN_1090
SF_46	Zinc Ribbons	Structural	1	1	2jox_A_ZN_108_ZN_1639-2jox_A_ZN_109_ZN_1640
SF_47	Zinc Ribbons	Unknown	1	1	2k5c_A_ZN_96_ZN_1469
SF_48	Zinc Ribbons	Structural	2	1	2r6f_A_ZN_1004_ZN_13925
SF_49	Zinc Ribbons	Structural	13	1	2rf5_A_ZN_1_ZN_1666
SF_50	Zinc Ribbons	Structural	2	1	2riq_A_ZN_1_ZN_1066
SF_51	Zinc Ribbons	Structural	8	1	2xoc_A_ZN_992_ZN_3435-2xoc_A_ZN_993_ZN_3436
SF_52	Zinc Ribbons	Structural	5	1	2zae_B_ZN_121_ZN_3407
SF_53	Zinc Ribbons	Unknown	2	1	3bvo_A_ZN_301_ZN_3099
SF_54	Zinc Ribbons	Unknown	4	1	3cng_A_ZN_508_ZN_5811
SF_55	Zinc Ribbons	Unknown	3	1	3e9s_A_ZN_318_ZN_2520
SF_56	Zinc Ribbons	Unknown	2	1	3epz_A_ZN_701_ZN_3449
SF_57	Zinc Ribbons	Structural	3	1	3f2b_A_ZN_4_ZN_8465
SF_58	Zinc Ribbons	Structural	4	1	3flo_B_ZN_1_ZN_19830
SF_59	Zinc Ribbons	Structural	4	1	3flo_B_ZN_2_ZN_19831
SF_60	Zinc Ribbons	Unknown	2	1	3ir9_A_ZN_501_ZN_2441
SF_61	Zinc Ribbons	Unknown	2	1	3irb_A_ZN_201_ZN_2190
SF_62	Treble Clefs	Structural	399	100	1vfy_A_ZN_300_ZN_534
SF_63	Treble Clefs	Structural	470	69	1rut_X_ZN_601_ZN_1252
SF_64	Treble Clefs	Structural	52	28	1ptq_A_ZN_2_ZN_405
SF_65	Treble Clefs	Structural	40	13	2d8q_A_ZN_201_ZN_1009
SF_66	Treble Clefs	Structural	51	9	1a1t_A_ZN_56_ZN_1517
SF_67	Treble Clefs	Structural	100	8	3g9m_A_ZN_527_ZN_1900
SF_68	Treble Clefs	Structural	69	6	1jw9_B_ZN_250_ZN_2410
SF_69	Treble Clefs	Structural	26	6	3eb5_A_ZN_1001_ZN_525
SF_70	Treble Clefs	Structural	18	5	1wfe_A_ZN_201_ZN_1286
SF_71	Treble Clefs	Structural	10	5	2csv_A_ZN_200_ZN_1066
SF_72	Treble Clefs	Structural	6	4	2d8r_A_ZN_401_ZN_1532
SF_73	Treble Clefs	Structural	15	3	1en7_A_ZN_401_ZN_2547
SF_74	Treble Clefs	Structural	67	3	1twf_J_ZN_3001_ZN_28290
SF_75	Treble Clefs	Structural	28	3	2d6f_D_ZN_1900_ZN_17642
SF_76	Treble Clefs	Structural	7	3	2i9w_A_ZN_201_ZN_1393
SF_77	Treble Clefs	Structural	120	3	2ioi_A_ZN_3001_ZN_1473
SF_78	Treble Clefs	Structural	16	2	1hc7_A_ZN_490_ZN_14993
SF_79	Treble Clefs	Substrate	2	2	1jjd_A_ZN_101_ZN_690-1jjd_A_ZN_102_ZN_691-1jjd_A_ZN_103_ZN_692-1jjd_A_ZN_104_ZN_693
SF_80	Treble Clefs	Structural	4	2	2cs8_A_ZN_601_ZN_1544
SF_81	Treble Clefs	Structural	3	2	2x7m_A_ZN_1175_ZN_2888
SF_82	Treble Clefs	Structural	5	2	3ebe_A_ZN_500_ZN_4132
SF_83	Treble Clefs	Structural	11	2	3gl6_A_ZN_3_ZN_471

SF_84	Treble Clefs	Structural	2	1	1ffy_A_ZN_1001_ZN_9024
SF_85	Treble Clefs	Structural	2	1	1i3j_A_ZN_100_ZN_1641
SF_86	Treble Clefs	Structural	2	1	1irx_A_ZN_600_ZN_8505
SF_87	Treble Clefs	Structural	5	1	1ul4_A_ZN_138_ZN_1328
SF_88	Treble Clefs	Structural	2	1	1urj_A_ZN_2131_ZN_15696
SF_89	Treble Clefs	Structural	6	1	1vdd_A_ZN_230_ZN_6037
SF_90	Treble Clefs	Structural	1	1	1z60_A_ZN_1_ZN_878
SF_91	Treble Clefs	Unknown	4	1	2a1k_A_ZN_1_ZN_3437
SF_92	Treble Clefs	Structural	3	1	2ac3_A_ZN_531_ZN_2217
SF_93	Treble Clefs	Structural	2	1	2avu_E_ZN_400_ZN_5322
SF_94	Treble Clefs	Unknown	2	1	2dkt_A_ZN_191_ZN_2164
SF_95	Treble Clefs	Structural	2	1	2e61_A_ZN_201_ZN_1012
SF_96	Treble Clefs	Structural	1	1	2ea5_A_ZN_201_ZN_966
SF_97	Treble Clefs	Structural	6	1	2fe3_A_ZN_201_ZN_2275
SF_98	Treble Clefs	Structural	8	1	2hqh_E_ZN_1500_ZN_2934
SF_99	Treble Clefs	Unknown	1	1	2hye_B_ZN_3002_ZN_16944
SF_100	Treble Clefs	Structural	2	1	2inp_A_ZN_2_ZN_16003
SF_101	Treble Clefs	Structural	68	1	2j02_Z_ZN_639_ZN_56061
SF_102	Treble Clefs	Structural	2	1	2jmi_A_ZN_201_ZN_926
SF_103	Treble Clefs	Structural	2	1	2k0a_A_ZN_108_ZN_1716
SF_104	Treble Clefs	Structural	1	1	2k0a_A_ZN_109_ZN_1717
SF_105	Treble Clefs	Structural	4	1	2k17_A_ZN_940_ZN_1356
SF_106	Treble Clefs	Structural	1	1	2kdp_A_ZN_1_ZN_1157
SF_107	Treble Clefs	Structural	3	1	2kgg_A_ZN_54_ZN_748
SF_108	Treble Clefs	Structural	1	1	2kgo_A_ZN_109_ZN_1334
SF_109	Treble Clefs	Structural	1	1	2o03_A_ZN_202_ZN_957
SF_110	Treble Clefs	Structural	1	1	2ro1_A_ZN_201_ZN_2907
SF_111	Treble Clefs	Structural	7	1	2v89_B_ZN_1488_ZN_2726
SF_112	Treble Clefs	Structural	10	1	2v89_B_ZN_1489_ZN_2727
SF_113	Treble Clefs	Structural	1	1	2v9k_A_ZN_1533_ZN_3708
SF_114	Treble Clefs	Structural	14	1	2wb0_X_ZN_601_ZN_2479
SF_115	Treble Clefs	Structural	5	1	2wjy_A_ZN_1_ZN_6170
SF_116	Treble Clefs	Structural	5	1	2wjy_A_ZN_3_ZN_6172
SF_117	Treble Clefs	Unknown	1	1	2x5r_A_ZN_1126_ZN_970
SF_118	Treble Clefs	Structural	5	1	2zze_A_ZN_753_ZN_12283
SF_119	Treble Clefs	Structural	4	1	3gox_A_ZN_301_ZN_3917
SF_120	Treble Clefs	Unknown	1	1	3h0n_A_ZN_201_ZN_1501
SF_121	Treble Clefs	Structural	4	1	3kno_4_ZN_1_ZN_90400
SF_122	Treble Clefs	Structural	4	1	3pt9_A_ZN_2_ZN_6501
SF_123	Zinc Necklaces	Catalytic	150	24	1p6o_B_ZN_400_ZN_2523
SF_124	Zinc Necklaces	Structural	236	19	1n8k_A_ZN_376_ZN_5609
SF_125	Zinc Necklaces	Unknown	38	8	1ml9_A_ZN_1_ZN_1925-1ml9_A_ZN_2_ZN_1926-1ml9_A_ZN_3_ZN_1927
SF_126	Zinc Necklaces	Unknown	33	6	2rfi_A_ZN_504_ZN_4330

SF_127	Zinc Necklaces	Structural	57	4	1oyw_A_ZN_801_ZN_4066
SF_128	Zinc Necklaces	Structural	6	4	1t9h_A_ZN_411_ZN_2348
SF_129	Zinc Necklaces	Structural	29	4	2e5s_A_ZN_401_ZN_1472
SF_130	Zinc Necklaces	Structural	6	3	1m9o_A_ZN_78_ZN_621
SF_131	Zinc Necklaces	Structural	75	3	1r5y_A_ZN_400_ZN_2969
SF_132	Zinc Necklaces	Regulatory	7	3	1u2w_A_ZN_501_ZN_3156
SF_133	Zinc Necklaces	Structural	30	2	1f81_A_ZN_88_ZN_1374
SF_134	Zinc Necklaces	Structural	16	2	1ia9_A_ZN_2001_ZN_4494
SF_135	Zinc Necklaces	Regulatory	5	2	1q08_A_ZN_401_ZN_1461-1q08_A_ZN_402_ZN_1462
SF_136	Zinc Necklaces	Structural	60	2	1twf_C_ZN_3002_ZN_28291
SF_137	Zinc Necklaces	Unknown	6	2	1xto_A_ZN_312_ZN_2346
SF_138	Zinc Necklaces	Structural	33	2	2a6h_D_ZN_7412_ZN_53475
SF_139	Zinc Necklaces	Unknown	3	2	2glz_A_ZN_200_ZN_2445
SF_140	Zinc Necklaces	Structural	16	1	1a73_A_ZN_201_ZN_3354
SF_141	Zinc Necklaces	Structural	16	1	1a73_A_ZN_202_ZN_3355
SF_142	Zinc Necklaces	Structural	2	1	1kwg_A_ZN_806_ZN_5173
SF_143	Zinc Necklaces	Structural	1	1	1lpv_A_ZN_53_ZN_862
SF_144	Zinc Necklaces	Substrate	1	1	1mwz_A_ZN_75_ZN_1102
SF_145	Zinc Necklaces	Structural	2	1	1oqj_A_ZN_183_ZN_1411
SF_146	Zinc Necklaces	Structural	4	1	1rni_A_ZN_256_ZN_1708
SF_147	Zinc Necklaces	Structural	24	1	1svm_A_ZN_700_ZN_17611
SF_148	Zinc Necklaces	Structural	1	1	1t3k_A_ZN_201_ZN_2060
SF_149	Zinc Necklaces	Structural	3	1	1vsr_A_ZN_201_ZN_1099
SF_150	Zinc Necklaces	Structural	1	1	1z3i_X_ZN_900_ZN_5054
SF_151	Zinc Necklaces	Structural	4	1	2a5h_A_ZN_421_ZN_13155
SF_152	Zinc Necklaces	Structural	3	1	2b5l_C_ZN_3001_ZN_20400
SF_153	Zinc Necklaces	Unknown	2	1	2bjr_A_ZN_1369_ZN_5639
SF_154	Zinc Necklaces	Structural	3	1	2f44_A_ZN_255_ZN_1597
SF_155	Zinc Necklaces	Structural	2	1	2fea_A_ZN_1302_ZN_3656
SF_156	Zinc Necklaces	Structural	49	1	2fyg_A_ZN_302_ZN_963
SF_157	Zinc Necklaces	Structural	1	1	2iwj_A_ZN_1050_ZN_420
SF_158	Zinc Necklaces	Structural	6	1	2j2s_A_ZN_2215_ZN_1136
SF_159	Zinc Necklaces	Substrate	1	1	2kak_A_ZN_150_ZN_636-2kak_A_ZN_170_ZN_637-2kak_A_ZN_190_ZN_638
SF_160	Zinc Necklaces	Unknown	1	1	2kr1_A_ZN_65_ZN_993
SF_161	Zinc Necklaces	Unknown	7	1	2pg3_A_ZN_300_ZN_1668
SF_162	Zinc Necklaces	Structural	12	1	2pkg_C_ZN_175_ZN_10359
SF_163	Zinc Necklaces	Unknown	1	1	2yre_A_ZN_501_ZN_1428
SF_164	Zinc Necklaces	Structural	12	1	3c0y_B_ZN_404_ZN_8438
SF_165	Zinc Necklaces	Unknown	1	1	3f07_A_ZN_409_ZN_8384
SF_166	Zinc Necklaces	Unknown	1	1	3hko_A_ZN_701_ZN_2604
SF_167	Zinc Necklaces	Unknown	1	1	3l0a_A_ZN_266_ZN_2147
SF_168	Zinc Necklaces	Structural	3	1	3mhs_A_ZN_475_ZN_6614
SF_169	Zinc Necklaces	Structural	3	1	3mhs_A_ZN_476_ZN_6615

SF_170	Zinc Necklaces	Unknown	2	1	3mi9_C_ZN_88_ZN_5060
SF_171	Zinc Necklaces	Structural	9	1	3mln_A_ZN_501_ZN_5634
SF_172	Zinc Rafts	Catalytic	50	15	2o1q_A_ZN_145_ZN_2234
SF_173	Zinc Rafts	Unknown	15	9	1dyq_A_ZN_234_ZN_1884
SF_174	Zinc Rafts	Catalytic	30	9	1zsw_A_ZN_315_ZN_2857
SF_175	Zinc Rafts	Catalytic	365	8	1lug_A_ZN_1001_ZN_2122
SF_176	Zinc Rafts	Catalytic	97	7	1t0a_A_ZN_661_ZN_3559
SF_177	Zinc Rafts	Catalytic	42	7	1vhh_A_ZN_400_ZN_1559
SF_178	Zinc Rafts	Catalytic	37	5	1evl_A_ZN_1_ZN_13067
SF_179	Zinc Rafts	Catalytic	13	5	1im5_A_ZN_400_ZN_1439
SF_180	Zinc Rafts	Catalytic	18	5	1sr9_B_ZN_703_ZN_8681
SF_181	Zinc Rafts	Catalytic	17	5	1thj_A_ZN_214_ZN_4834
SF_182	Zinc Rafts	Catalytic	26	5	2g64_A_ZN_2001_ZN_1190
SF_183	Zinc Rafts	Catalytic	78	5	2v9l_A_ZN_1275_ZN_4535
SF_184	Zinc Rafts	Structural	269	4	1i76_A_ZN_998_ZN_1318
SF_185	Zinc Rafts	Catalytic	10	4	2p6y_A_ZN_201_ZN_1019
SF_186	Zinc Rafts	Catalytic	16	3	2fli_B_ZN_1982_ZN_19766
SF_187	Zinc Rafts	Substrate	15	3	3byr_A_ZN_501_ZN_735-3byr_A_ZN_502_ZN_736
SF_188	Zinc Rafts	Catalytic	3	2	1m55_B_ZN_201_ZN_3166
SF_189	Zinc Rafts	Catalytic	7	2	1oi0_A_ZN_1122_ZN_3386
SF_190	Zinc Rafts	Catalytic	13	2	1qwy_A_ZN_400_ZN_1824
SF_191	Zinc Rafts	Unknown	22	2	2aq2_B_ZN_1001_ZN_2767
SF_192	Zinc Rafts	Catalytic	6	2	2hsi_A_ZN_283_ZN_3487
SF_193	Zinc Rafts	Catalytic	7	2	2nly_A_ZN_300_ZN_1652
SF_194	Zinc Rafts	Regulatory	2	2	2o03_A_ZN_203_ZN_958
SF_195	Zinc Rafts	Catalytic	11	2	3bq5_A_ZN_800_ZN_11233
SF_196	Zinc Rafts	Unknown	16	2	3chv_A_ZN_302_ZN_2263
SF_197	Zinc Rafts	Catalytic	2	1	1ef0_A_ZN_701_ZN_6479
SF_198	Zinc Rafts	Structural	10	1	1eh6_A_ZN_208_ZN_1274
SF_199	Zinc Rafts	Regulatory	7	1	1enr_A_ZN_239_ZN_1826
SF_200	Zinc Rafts	Unknown	3	1	1r61_A_ZN_1001_ZN_3256
SF_201	Zinc Rafts	Unknown	3	1	1txl_A_ZN_216_ZN_1539
SF_202	Zinc Rafts	Structural	4	1	1yg9_A_ZN_401_ZN_2611
SF_203	Zinc Rafts	Catalytic	2	1	1yt3_A_ZN_1001_ZN_3094
SF_204	Zinc Rafts	Unknown	3	1	2cs7_C_ZN_203_ZN_1301
SF_205	Zinc Rafts	Structural	2	1	2faw_A_ZN_1001_ZN_4176
SF_206	Zinc Rafts	Catalytic	2	1	2fgy_B_ZN_721_ZN_7376
SF_207	Zinc Rafts	Structural	24	1	2ijd_1_ZN_645_ZN_10123
SF_208	Zinc Rafts	Structural	2	1	2j7u_A_ZN_1884_ZN_4703
SF_209	Zinc Rafts	Structural	1	1	2jox_A_ZN_110_ZN_1641
SF_210	Zinc Rafts	Unknown	1	1	2l0z_A_ZN_486_ZN_670
SF_211	Zinc Rafts	Regulatory	1	1	2o03_A_ZN_201_ZN_956
SF_212	Zinc Rafts	Unknown	6	1	2oog_B_ZN_401_ZN_13372

SF_213	Zinc Rafts	Unknown	2	1	2oso_A_ZN_163_ZN_1323
SF_214	Zinc Rafts	Unknown	2	1	2peb_A_ZN_200_ZN_1875
SF_215	Zinc Rafts	Unknown	2	1	2q1z_B_ZN_197_ZN_5181
SF_216	Zinc Rafts	Catalytic	58	1	3hka_C_ZN_429_ZN_10219
SF_217	C2H2 Zinc Fingers	Structural	385	75	1llm_C_ZN_301_ZN_2012
SF_218	C2H2 Zinc Fingers	Structural	140	11	2qfa_A_ZN_143_ZN_2093
SF_219	C2H2 Zinc Fingers	Structural	11	6	2csv_A_ZN_400_ZN_1067
SF_220	C2H2 Zinc Fingers	Structural	73	5	1k6y_A_ZN_401_ZN_5929
SF_221	C2H2 Zinc Fingers	Catalytic	55	5	1wur_A_ZN_1001_ZN_7332
SF_222	C2H2 Zinc Fingers	Structural	36	5	2dip_A_ZN_401_ZN_1515
SF_223	C2H2 Zinc Fingers	Structural	8	5	3c5k_A_ZN_201_ZN_842
SF_224	C2H2 Zinc Fingers	Structural	19	4	1k2f_B_ZN_606_ZN_3016
SF_225	C2H2 Zinc Fingers	Structural	11	3	3hct_A_ZN_303_ZN_2021
SF_226	C2H2 Zinc Fingers	Structural	2	2	1pi1_A_ZN_196_ZN_1517
SF_227	C2H2 Zinc Fingers	Structural	6	2	1zw8_A_ZN_66_ZN_1000
SF_228	C2H2 Zinc Fingers	Structural	16	2	2q1z_B_ZN_196_ZN_5180
SF_229	C2H2 Zinc Fingers	Structural	9	2	3eph_A_ZN_1_ZN_9627
SF_230	C2H2 Zinc Fingers	Structural	1	1	1ej6_C_ZN_2000_ZN_34492
SF_231	C2H2 Zinc Fingers	Structural	1	1	2bai_A_ZN_110_ZN_496
SF_232	C2H2 Zinc Fingers	Structural	2	1	2dkt_A_ZN_241_ZN_2165
SF_233	C2H2 Zinc Fingers	Structural	2	1	2dmi_A_ZN_300_ZN_1767
SF_234	C2H2 Zinc Fingers	Structural	5	1	2giv_A_ZN_501_ZN_2108
SF_235	C2H2 Zinc Fingers	Structural	2	1	2k9h_A_ZN_101_ZN_842
SF_236	C2H2 Zinc Fingers	Structural	5	1	2wjy_A_ZN_2_ZN_6171
SF_237	Loosened Zinc Ribbons	Structural	27	13	1ptq_A_ZN_1_ZN_404
SF_238	Loosened Zinc Ribbons	Structural	75	5	1twf_A_ZN_3008_ZN_28297
SF_239	Loosened Zinc Ribbons	Structural	4	4	1bor_A_ZN_58_ZN_424
SF_240	Loosened Zinc Ribbons	Regulatory	6	3	1vzy_A_ZN_1291_ZN_4385
SF_241	Loosened Zinc Ribbons	Structural	26	3	2gmw_A_ZN_300_ZN_3050
SF_242	Loosened Zinc Ribbons	Structural	88	3	2hrv_A_ZN_143_ZN_2227
SF_243	Loosened Zinc Ribbons	Structural	2	2	1q68_A_ZN_201_ZN_1112
SF_244	Loosened Zinc Ribbons	Structural	3	2	1wj2_A_ZN_470_ZN_1123
SF_245	Loosened Zinc Ribbons	Structural	50	2	2fyg_A_ZN_303_ZN_964
SF_246	Loosened Zinc Ribbons	Structural	6	1	1dy0_A_ZN_401_ZN_1402
SF_247	Loosened Zinc Ribbons	Structural	5	1	1fn9_A_ZN_1001_ZN_5767
SF_248	Loosened Zinc Ribbons	Structural	2	1	1mr1_C_ZN_601_ZN_4671
SF_249	Loosened Zinc Ribbons	Structural	1	1	1odh_A_ZN_1171_ZN_1808
SF_250	Loosened Zinc Ribbons	Structural	44	1	1v54_F_ZN_99_ZN_29334
SF_251	Loosened Zinc Ribbons	Structural	4	1	2dkt_A_ZN_441_ZN_2169
SF_252	Loosened Zinc Ribbons	Structural	1	1	3ifu_A_ZN_182_ZN_1370
SF_253	Helical Anchors	Catalytic	758	74	1c7k_A_ZN_133_ZN_1017
SF_254	Helical Anchors	Catalytic	66	15	1j98_A_ZN_300_ZN_1239
SF_255	Helical Anchors	Regulatory	20	5	2psr_A_ZN_103_ZN_772

SF_256	Helical Anchors	Catalytic	15	4	1v4p_B_ZN_1002_ZN_3729
SF_257	Helical Anchors	Catalytic	26	2	2ves_A_ZN_1295_ZN_6918
SF_258	Helical Anchors	Catalytic	7	2	1u0b_B_ZN_462_ZN_5097
SF_259	Helical Anchors	Catalytic	17	2	2ce7_A_ZN_1603_ZN_19198
SF_260	Helical Anchors	Unknown	2	2	3fvy_A_ZN_1000_ZN_5642
SF_261	Helical Anchors	Structural	1	1	1au1_B_ZN_169_ZN_3625
SF_262	Helical Anchors	Catalytic	35	1	1fr2_B_ZN_301_ZN_1697
SF_263	Helical Anchors	Unknown	1	1	1oek_A_ZN_1195_ZN_1517
SF_264	Helical Anchors	Catalytic	31	1	1sg0_A_ZN_231_ZN_3651
SF_265	Helical Anchors	Unknown	7	1	2j7u_A_ZN_1885_ZN_4704
SF_266	Helical Anchors	Unknown	12	1	2zh0_B_ZN_4002_ZN_12805
SF_267	Helical Anchors	Catalytic	2	1	3hwp_A_ZN_295_ZN_4707
SF_268	Shuffled Zinc Ribbons - type I	Structural	6	3	1nlt_A_ZN_351_ZN_1816
SF_269	Shuffled Zinc Ribbons - type I	Structural	14	2	2r6f_A_ZN_1005_ZN_13926
SF_270	Shuffled Zinc Ribbons - type I	Structural	6	2	2vmk_B_ZN_1514_ZN_14180
SF_271	Shuffled Zinc Ribbons - type I	Catalytic	5	1	1adn_A_ZN_93_ZN_733
SF_272	Shuffled Zinc Ribbons - type I	Unknown	2	1	1p9r_A_ZN_601_ZN_2934
SF_273	Shuffled Zinc Ribbons - type I	Structural	42	1	2i2t_4_ZN_101_ZN_89790
SF_274	Shuffled Zinc Ribbons - type I	Structural	2	1	2rhq_A_ZN_1_ZN_8338
SF_275	Shuffled Zinc Ribbons - type I	Structural	78	1	2zp8_E_ZN_54_ZN_4504
SF_276	Shuffled Zinc Ribbons - type II	Structural	11	3	1btk_A_ZN_1_ZN_2675
SF_277	Shuffled Zinc Ribbons - type II	Unknown	3	2	2yrt_A_ZN_401_ZN_1093
SF_278	Zn2Cys6 Zinc Fingers	Structural	31	6	1hwt_C_ZN_136_ZN_4004-1hwt_C_ZN_137_ZN_4005
SF_279	Zn2Cys6 Zinc Fingers	Structural	9	3	1ldj_B_ZN_203_ZN_6670
SF_280	Zn2Cys6 Zinc Fingers	Structural	8	2	3h84_A_ZN_355_ZN_5076
SF_281	Zn2Cys6 Zinc Fingers	Structural	1	1	1co4_A_ZN_43_ZN_591
SF_282	Zn2Cys6 Zinc Fingers	Structural	1	1	1lpv_A_ZN_54_ZN_863
SF_283	Zn2Cys6 Zinc Fingers	Structural	4	1	1v33_A_ZN_1000_ZN_2859
SF_284	Zn2Cys6 Zinc Fingers	Structural	34	1	2ox0_A_ZN_502_ZN_5784
SF_285	Unclustered – Peptidase-like sites	Catalytic	222	33	2gmn_A_ZN_801_ZN_4041-2gmn_A_ZN_802_ZN_4042
SF_286	Unclustered – Peptidase-like sites	Catalytic	191	29	1rtq_A_ZN_701_ZN_4308-1rtq_A_ZN_702_ZN_4309
SF_287	Unclustered – Peptidase-like sites	Catalytic	161	21	1m4l_A_ZN_1308_ZN_2464
SF_288	Unclustered – Peptidase-like sites	Catalytic	104	11	1ekj_A_ZN_4001_ZN_13182
SF_289	Unclustered – Peptidase-like sites	Catalytic	107	10	1ed8_A_ZN_450_ZN_6623-1ed8_A_ZN_451_ZN_6624-1ed8_A_ZN_452_ZN_6625
SF_290	Unclustered – Peptidase-like sites	Catalytic	40	9	1hp1_A_ZN_600_ZN_4029-1hp1_A_ZN_601_ZN_4030
SF_291	Unclustered – Peptidase-like sites	Catalytic	77	6	1t64_A_ZN_388_ZN_5631
SF_292	Unclustered – Peptidase-like sites	Catalytic	84	5	1lam_A_ZN_488_ZN_3707-1lam_A_ZN_489_ZN_3708
SF_293	Unclustered – Peptidase-like sites	Substrate	20	5	1toa_A_ZN_501_ZN_4295
SF_294	Unclustered – Peptidase-like sites	Catalytic	12	3	2ixd_A_ZN_1234_ZN_3723
SF_295	Unclustered – Peptidase-like sites	Catalytic	84	2	1j2u_A_ZN_301_ZN_11936-1j2u_A_ZN_302_ZN_11935
SF_296	Unclustered – Peptidase-like sites	Unknown	7	2	2gx8_A_ZN_374_ZN_8382-2gx8_A_ZN_375_ZN_8383
SF_297	Unclustered – Peptidase-like sites	Unknown	3	2	3di4_A_ZN_286_ZN_4346
SF_298	Unclustered – Peptidase-like sites	Catalytic	5	1	1hi9_A_ZN_300_ZN_10576-1hi9_A_ZN_301_ZN_10577

SF_299	Unclustered – Peptidase-like sites	Catalytic	8	1	1ps6_A_ZN_330_ZN_4807
SF_300	Unclustered – Peptidase-like sites	Catalytic	3	1	2bz1_A_ZN_1174_ZN_1350
SF_301	Unclustered – Peptidase-like sites	Unknown	3	1	2h1i_A_ZN_300_ZN_4873
SF_302	Unclustered – Peptidase-like sites	Unknown	1	1	2pw6_A_ZN_272_ZN_1871
SF_303	Unclustered – Peptidase-like sites	Unknown	2	1	2q7s_A_ZN_400_ZN_4407
SF_304	Unclustered – Peptidase-like sites	Unknown	1	1	3iuu_A_ZN_495_ZN_3828
SF_305	Unclustered – Half zinc ribbons	Structural	53	9	1hxp_A_ZN_350_ZN_5357
SF_306	Unclustered – Half zinc ribbons	Structural	20	7	2ap1_A_ZN_304_ZN_2392
SF_307	Unclustered – Half zinc ribbons	Structural	6	2	2ofk_A_ZN_201_ZN_2904
SF_308	Unclustered – Half zinc ribbons	Structural	3	2	2yrt_A_ZN_201_ZN_1092
SF_309	Unclustered – Half zinc ribbons	Structural	6	1	1zh1_A_ZN_199_ZN_2527
SF_310	Unclustered – Half zinc ribbons	Structural	2	1	2b5l_C_ZN_3002_ZN_20401
SF_311	Unclustered – Half zinc ribbons	Unknown	4	1	2eg3_A_ZN_301_ZN_3589
SF_312	Unclustered – Half zinc ribbons	Regulatory	1	1	2iim_A_ZN_500_ZN_508
SF_313	Unclustered – Half zinc ribbons	Structural	1	1	2k7r_A_ZN_129_ZN_1735
SF_314	Unclustered – Half zinc ribbons	Substrate	1	1	2kak_A_ZN_130_ZN_635
SF_315	Unclustered – Half zinc ribbons	Structural	10	1	2xam_A_ZN_700_ZN_6760
SF_316	Unclustered – Half zinc ribbons	Unknown	8	1	2xoc_A_ZN_991_ZN_3434
SF_317	Unclustered – Half zinc ribbons	Unknown	1	1	2yre_A_ZN_401_ZN_1427
SF_318	Unclustered – Half zinc ribbons	Structural	6	1	3cg7_A_ZN_299_ZN_4825
SF_319	Unclustered – Endonuclease-like sites	Catalytic	377	68	1pb0_A_ZN_1301_ZN_5439-1pb0_A_ZN_1302_ZN_5440-1pb0_A_ZN_1303_ZN_5441
SF_320	Unclustered – Endonuclease-like sites	Catalytic	52	11	1qtw_A_ZN_301_ZN_4400-1qtw_A_ZN_302_ZN_4401-1qtw_A_ZN_303_ZN_4402
SF_321	Unclustered – Endonuclease-like sites	Catalytic	37	5	2isw_A_ZN_326_ZN_4551
SF_322	Unclustered – Endonuclease-like sites	Catalytic	32	3	1ohl_A_ZN_400_ZN_2668
SF_323	Unclustered – Endonuclease-like sites	Catalytic	10	2	3bof_A_ZN_701_ZN_8766
SF_324	Unclustered – Endonuclease-like sites	Regulatory	2	1	2ci7_A_ZN_1281_ZN_2150
SF_325	Unclustered – Endonuclease-like sites	Catalytic	8	1	2i2x_A_ZN_501_ZN_43897
SF_326	Unclustered – Endonuclease-like sites	Catalytic	1	1	3fvz_A_ZN_821_ZN_2726
SF_327	Unclustered – Two-helix sites	Structural	14	3	1nn7_A_ZN_201_ZN_889
SF_328	Unclustered – Two-helix sites	Structural	11	2	2dsn_A_ZN_2001_ZN_6109
SF_329	Unclustered – Two-helix sites	Structural	1	1	1bp3_A_ZN_500_ZN_3125
SF_330	Unclustered – Two-helix sites	Unknown	4	1	1mbx_A_ZN_210_ZN_3636
SF_331	Unclustered – Two-helix sites	Unknown	1	1	1ons_A_ZN_451_ZN_2168
SF_332	Unclustered – Two-helix sites	Structural	6	1	2j0e_A_ZN_1265_ZN_3943
SF_333	Unclustered – Two-helix sites	Unknown	1	1	2oo4_A_ZN_6001_ZN_3417
SF_334	Unclustered – Two-helix sites	Unknown	2	1	3b5q_A_ZN_500_ZN_7285
SF_335	Unclustered – Three-helix sites	Catalytic	227	12	1tbf_A_ZN_1_ZN_2659
SF_336	Unclustered – Three-helix sites	Catalytic	49	9	1kq3_A_ZN_401_ZN_2794
SF_337	Unclustered – Three-helix sites	Catalytic	30	3	1ah7_A_ZN_246_ZN_2034-1ah7_A_ZN_247_ZN_2035-1ah7_A_ZN_248_ZN_2036
SF_338	Unclustered – Three-helix sites	Structural	154	3	1ev6_B_ZN_301_ZN_2437
SF_339	Unclustered – Three-helix sites	Catalytic	6	1	1kae_A_ZN_1101_ZN_6497
SF_340	Unclustered – Three-helix sites	Catalytic	12	1	2xbl_B_ZN_1196_ZN_5787
SF_341	Unclustered – Three-sheet sites	Catalytic	3	1	2zws_A_ZN_647_ZN_5238

SF_342	Unclustered – Three-sheet sites	Catalytic	2	1	3fns_A_ZN_329_ZN_5183
SF_343	Unclustered – Transferase-like sites	Catalytic	125	6	2h6f_B_ZN_1001_ZN_5978
SF_344	Unclustered – Transferase-like sites	Catalytic	8	3	2g0d_A_ZN_416_ZN_3224
SF_345	Unclustered	Catalytic	286	20	1n8k_A_ZN_375_ZN_5608
SF_346	Unclustered	Catalytic	19	7	2wkx_A_ZN_300_ZN_2087
SF_347	Unclustered	Structural	409	6	1mfm_A_ZN_154_ZN_1154
SF_348	Unclustered	Catalytic	62	3	2ow6_A_ZN_3001_ZN_8403
SF_349	Unclustered	Unknown	3	2	1nzj_A_ZN_700_ZN_2165
SF_350	Unclustered	Regulatory	3	2	1ton_A_ZN_247_ZN_1736
SF_351	Unclustered	Structural	6	2	2hvw_A_ZN_1001_ZN_3524
SF_352	Unclustered	Structural	14	2	2xoc_A_ZN_995_ZN_3438
SF_353	Unclustered	Structural	33	2	3ny3_A_ZN_1_ZN_579-3ny3_A_ZN_2_ZN_580
SF_354	Unclustered	Regulatory	3	1	1f5f_A_ZN_251_ZN_1369
SF_355	Unclustered	Structural	4	1	1ozj_A_ZN_145_ZN_2686
SF_356	Unclustered	Regulatory	1	1	1uns_A_ZN_1238_ZN_1896
SF_357	Unclustered	Catalytic	4	1	1x6m_A_ZN_201_ZN_5940
SF_358	Unclustered	Structural	8	1	1xer_A_ZN_106_ZN_772
SF_359	Unclustered	Unknown	2	1	2c2u_A_ZN_1209_ZN_1512
SF_360	Unclustered	Structural	9	1	2fuq_A_ZN_1_ZN_12000
SF_361	Unclustered	Unknown	1	1	2l0z_A_ZN_487_ZN_671
SF_362	Unclustered	Unknown	1	1	2qsw_A_ZN_201_ZN_804
SF_363	Unclustered	Regulatory	2	1	3kdk_A_ZN_1_ZN_3045
SF_364	Unclustered	Regulatory	1	1	3lnn_A_ZN_360_ZN_4347
SF_365	Unclustered	Unknown	2	1	3mi9_C_ZN_87_ZN_5059
SF_366	Unclustered	Unknown	2	1	3mo0_A_ZN_10_ZN_3923
SF_367	Unclustered	Structural	10	1	3pt9_A_ZN_1_ZN_6500
