# Supplementary material for: Minimal Functional Sites Allow a Classification of Zinc Sites in Proteins
Source: PLoS One. 2011 Oct 17;6(10):e26325. doi: 10.1371/journal.pone.0026325 (PMC3197139; doi:10.1371/journal.pone.0026325)
Supplement: Table S3 — Lists of the Zn-sites belonging to each Zn-superfamily. (PDF) [file pone.0026325.s003.pdf]

Table S3. Lists of the Zn-sites belonging to each Zn-superfamily (SF). Each Zn atom in a Zn-site is identified by six fields (separated by underscores) as (i) PDB code, (ii) chain identifier, (iii) residue name, (iv) residue number, (v) atom name, and (vi) atom number. Multiple Zn atoms in the same Zn-site are separated by commas.

```
------- SF_1 --------
1chc_A_ZN_70_ZN_1043
1e4u_A_ZN_80_ZN_1232
1fbv_A_ZN_1002_ZN_4371
1g25_A_ZN_67_ZN_1002
1iym_A_ZN_183_ZN_796
1jm7_A_ZN_124_ZN_3162
1jm7_B_ZN_144_ZN_3164
1ldj_B_ZN_202_ZN_6669
1rmd_A_ZN_119_ZN_915
1u6g_B_ZN_1230_ZN_15495
1ur6_B_ZN_80_ZN_3016
1v87_A_ZN_401_ZN_1678
1vd4_A_ZN_175_ZN_949
1weo_A_ZN_401_ZN_1345
1wim_A_ZN_401_ZN_1406
2cs3_A_ZN_400_ZN_1295
2csy_A_ZN_401_ZN_1203
2ct2_A_ZN_401_ZN_1324
2ct7_A_ZN_201_ZN_1339
2e2z_A_ZN_101_ZN_1574
2ect_A_ZN_401_ZN_1098
2egp_A_ZN_400_ZN_1117
2h0d_B_ZN_204_ZN_1595
2hye_D_ZN_4002_ZN_16947
2jmo_A_ZN_201_ZN_1134
2ko5_A_ZN_101_ZN_1501
2ppt_A_ZN_300_ZN_2137
2ppt_B_ZN_301_ZN_2138
2xeu_A_ZN_1066_ZN_510
2yur_A_ZN_401_ZN_1008
3dpl_R_ZN_202_ZN_3860
3dqv_R_ZN_4006_ZN_8842
3dqv_Y_ZN_4001_ZN_8843
3fl2_A_ZN_1002_ZN_910
3hcs_A_ZN_302_ZN_2522
3hcs_B_ZN_307_ZN_2527
3hct_A_ZN_302_ZN_2020
3k1l_A_ZN_382_ZN_6013
3k1l_B_ZN_382_ZN_5967
3l11_A_ZN_601_ZN_843
3ng2_A_ZN_1004_ZN_1042
3ng2_B_ZN_1002_ZN_1049

------- SF_2 --------
1d0q_A_ZN_151_ZN_1585
1d0q_B_ZN_151_ZN_1586
```

1dl6_A_ZN_60_ZN_872
1i3q_I_ZN_2003_ZN_28165
1i3q_I_ZN_2004_ZN_28166
1i50_I_ZN_3003_ZN_28295
1i50_I_ZN_3004_ZN_28296
1i6h_I_ZN_203_ZN_28436
1i6h_I_ZN_204_ZN_28437
1k83_I_ZN_3003_ZN_27841
1k83_I_ZN_3004_ZN_27842
1l1o_C_ZN_1_ZN_6574
1l1o_F_ZN_2_ZN_6575
1nik_I_ZN_203_ZN_28305
1nik_I_ZN_204_ZN_28306
1nui_A_ZN_501_ZN_3766
1nui_B_ZN_502_ZN_3769
1pft_A_ZN_51_ZN_767
1qyp_A_ZN_58_ZN_897
1r5u_I_ZN_204_ZN_28307
1r9s_I_ZN_203_ZN_28469
1r9s_I_ZN_204_ZN_28470
1r9t_I_ZN_203_ZN_29225
1r9t_I_ZN_204_ZN_29226
1rly_A_ZN_61_ZN_868
1sfo_I_ZN_203_ZN_28655
1sfo_I_ZN_204_ZN_28656
1tfi_A_ZN_51_ZN_773
1twa_I_ZN_3003_ZN_27731
1twa_I_ZN_3004_ZN_27732
1twc_I_ZN_3003_ZN_27755
1twc_I_ZN_3004_ZN_27756
1twf_I_ZN_3003_ZN_28292
1twf_I_ZN_3004_ZN_28293
1twg_I_ZN_3003_ZN_27733
1twg_I_ZN_3004_ZN_27734
1twh_I_ZN_3003_ZN_27702
1twh_I_ZN_3004_ZN_27703
1wcm_I_ZN_1121_ZN_30954
1wcm_I_ZN_1122_ZN_30955
1wii_A_ZN_201_ZN_1251
1y1v_I_ZN_203_ZN_31812
1y1v_I_ZN_204_ZN_31813
1y1v_S_ZN_310_ZN_31816
1y1w_I_ZN_203_ZN_31813
1y1w_I_ZN_204_ZN_31814
2akl_A_ZN_117_ZN_1738
2au3_A_ZN_501_ZN_3262
2b63_I_ZN_1121_ZN_31741
2b63_I_ZN_1122_ZN_31742
2b8k_I_ZN_123_ZN_31049
2e2h_I_ZN_203_ZN_28979
2e2h_I_ZN_204_ZN_28980
2e2i_I_ZN_203_ZN_29669
2e2i_I_ZN_204_ZN_29670
2e2j_I_ZN_203_ZN_29182
2e2j_I_ZN_204_ZN_29183
2hf1_A_ZN_102_ZN_928
2hf1_B_ZN_101_ZN_929
2ja5_A_ZN_2460_ZN_31669

2ja6_A_ZN_2460_ZN_32021
2ja7_A_ZN_2463_ZN_63943
2ja7_A_ZN_2466_ZN_63946
2ja8_A_ZN_2460_ZN_32010
2nvq_I_ZN_203_ZN_29376
2nvq_I_ZN_204_ZN_29377
2nvt_I_ZN_203_ZN_29144
2nvt_I_ZN_204_ZN_29145
2nvx_I_ZN_203_ZN_29389
2nvx_I_ZN_204_ZN_29390
2nvy_I_ZN_3003_ZN_28292
2nvy_I_ZN_3004_ZN_28293
2nvz_I_ZN_203_ZN_28981
2nvz_I_ZN_204_ZN_28982
2qkd_A_ZN_501_ZN_3023
2qkd_A_ZN_502_ZN_3024
2r7z_I_ZN_203_ZN_31815
2r7z_I_ZN_204_ZN_31816
2r92_I_ZN_203_ZN_31621
2r93_I_ZN_203_ZN_31509
2r93_I_ZN_204_ZN_31510
2vum_I_ZN_1121_ZN_32096
2vum_I_ZN_1122_ZN_32097
2x5c_A_ZN_1128_ZN_3196
2x5c_B_ZN_1131_ZN_3211
2yu9_I_ZN_203_ZN_29480
2yu9_I_ZN_204_ZN_29481
3cqz_I_ZN_3003_ZN_27331
3cqz_I_ZN_3004_ZN_27332
3fki_I_ZN_123_ZN_31420
3ga8_A_ZN_121_ZN_536
3gn5_A_ZN_132_ZN_2055
3gn5_B_ZN_132_ZN_2062
3gtg_I_ZN_203_ZN_30075
3gtg_I_ZN_204_ZN_30076
3gtj_I_ZN_203_ZN_29983
3gtk_I_ZN_203_ZN_30121
3gtl_I_ZN_203_ZN_29265
3gtl_I_ZN_204_ZN_29266
3gtm_I_ZN_203_ZN_30570
3gtm_I_ZN_204_ZN_30571
3gto_I_ZN_203_ZN_29268
3gto_I_ZN_204_ZN_29269
3gtp_I_ZN_203_ZN_29288
3gtp_I_ZN_204_ZN_29289
3gtq_I_ZN_203_ZN_28634
3gtq_I_ZN_204_ZN_28635
3h0g_I_ZN_1121_ZN_62882
3h0g_I_ZN_1122_ZN_62883
3h0g_U_ZN_1121_ZN_62891
3h0g_U_ZN_1122_ZN_62892
3h3v_J_ZN_123_ZN_31788
3h3v_J_ZN_124_ZN_31789
3hi2_A_ZN_121_ZN_2500
3hi2_C_ZN_121_ZN_2513
3hou_I_ZN_9988_ZN_63683
3hou_I_ZN_9989_ZN_63684
3hov_I_ZN_1121_ZN_31789

3hov_I_ZN_1122_ZN_31790
3how_I_ZN_1121_ZN_31888
3how_I_ZN_1122_ZN_31889
3hox_I_ZN_1121_ZN_31930
3hoy_I_ZN_1121_ZN_31815
3hoz_I_ZN_1121_ZN_31973
3i4m_I_ZN_1121_ZN_32367
3i4m_I_ZN_1122_ZN_32368
3i4n_I_ZN_1121_ZN_32319
3i4n_I_ZN_1122_ZN_32320
3k1f_I_ZN_3003_ZN_32341
3k1f_I_ZN_3004_ZN_32342
3k1f_M_ZN_3009_ZN_32345
3k7a_I_ZN_203_ZN_29036
3k7a_I_ZN_204_ZN_29037
3ndi_A_ZN_415_ZN_3232
3ndj_A_ZN_416_ZN_3237
3o9x_A_ZN_132_ZN_3102
3o9x_B_ZN_132_ZN_3103

------- SF_3 --------
1ici_A_ZN_2001_ZN_3901
1ici_B_ZN_2002_ZN_3946
1j8f_A_ZN_1001_ZN_8922
1j8f_B_ZN_2001_ZN_8923
1j8f_C_ZN_3001_ZN_8924
1m2g_A_ZN_999_ZN_1943
1m2h_A_ZN_999_ZN_1942
1m2j_A_ZN_999_ZN_1941
1m2k_A_ZN_999_ZN_1937
1m2n_A_ZN_999_ZN_3853
1m2n_B_ZN_999_ZN_3854
1ma3_A_ZN_1001_ZN_1984
1q14_A_ZN_2003_ZN_2298
1q17_A_ZN_401_ZN_6988
1q17_B_ZN_401_ZN_6989
1q17_C_ZN_401_ZN_6990
1q1a_A_ZN_701_ZN_2358
1s5p_A_ZN_1001_ZN_1817
1s7g_A_ZN_601_ZN_9542
1s7g_B_ZN_603_ZN_9628
1s7g_C_ZN_605_ZN_9730
1s7g_D_ZN_607_ZN_9818
1s7g_E_ZN_609_ZN_9843
1szc_A_ZN_701_ZN_2380
1szd_A_ZN_701_ZN_2381
1yc2_A_ZN_401_ZN_9751
1yc2_B_ZN_403_ZN_9828
1yc2_C_ZN_405_ZN_9939
1yc2_D_ZN_407_ZN_10028
1yc2_E_ZN_409_ZN_10111
1yc5_A_ZN_1001_ZN_1944
1z6u_A_ZN_1_ZN_2050
1z6u_B_ZN_3_ZN_2052
2b4y_A_ZN_1001_ZN_8020
2b4y_B_ZN_2001_ZN_8072
2b4y_C_ZN_3001_ZN_8124
2b4y_D_ZN_4001_ZN_8176

2ckl_A_ZN_1105_ZN_1566
2ecl_A_ZN_601_ZN_1220
2ecw_A_ZN_401_ZN_1252
2h0d_A_ZN_202_ZN_1593
2h2d_A_ZN_1001_ZN_1950
2h2f_A_ZN_1001_ZN_1899
2h2g_A_ZN_1001_ZN_1905
2h2h_A_ZN_1001_ZN_1898
2h2i_A_ZN_1000_ZN_1940
2h4f_A_ZN_1001_ZN_1936
2h4h_A_ZN_1001_ZN_1911
2h4j_A_ZN_1001_ZN_1939
2h59_A_ZN_1001_ZN_3957
2h59_B_ZN_1001_ZN_3994
2hjh_A_ZN_800_ZN_5137
2hjh_B_ZN_800_ZN_5185
2nyr_A_ZN_501_ZN_3950
2nyr_B_ZN_501_ZN_4037
2od2_A_ZN_701_ZN_2370
2od7_A_ZN_701_ZN_2349
2od9_A_ZN_701_ZN_2369
2qqf_A_ZN_701_ZN_2349
2qqg_A_ZN_701_ZN_2369
3d4b_A_ZN_1001_ZN_1854
3d81_A_ZN_1001_ZN_1959
3glr_A_ZN_1_ZN_2243
3gls_A_ZN_2_ZN_12861
3gls_B_ZN_4_ZN_12869
3gls_C_ZN_5_ZN_12870
3gls_D_ZN_6_ZN_12871
3gls_E_ZN_1_ZN_12872
3gls_F_ZN_3_ZN_12878
3glt_A_ZN_1_ZN_2234
3glu_A_ZN_400_ZN_2185
3jr3_A_ZN_1001_ZN_1920
3jwp_A_ZN_2001_ZN_1913
3k35_A_ZN_317_ZN_12762
3k35_B_ZN_317_ZN_12822
3k35_C_ZN_317_ZN_12872
3k35_D_ZN_317_ZN_12917
3k35_E_ZN_317_ZN_12967
3k35_F_ZN_317_ZN_13017

------- SF_4 --------
1ltl_A_ZN_275_ZN_11634
1ltl_B_ZN_275_ZN_11636
1ltl_C_ZN_275_ZN_11635
1ltl_D_ZN_275_ZN_11637
1ltl_E_ZN_275_ZN_11639
1ltl_F_ZN_275_ZN_11638
2con_A_ZN_201_ZN_1182
2d9g_A_ZN_201_ZN_742
2ea5_A_ZN_401_ZN_967
2k0c_A_ZN_54_ZN_515
2k1p_A_ZN_96_ZN_498
2k2c_A_ZN_140_ZN_2126
2k2d_A_ZN_80_ZN_715
2kdx_A_ZN_120_ZN_1856

2vl6_A_ZN_1266_ZN_6352
2vl6_B_ZN_1266_ZN_6353
2vl6_C_ZN_1266_ZN_6354
3a43_C_ZN_701_ZN_1986
3a43_C_ZN_702_ZN_1987
3a44_A_ZN_140_ZN_3853
3a44_B_ZN_140_ZN_3854
3a44_C_ZN_140_ZN_3855
3a44_C_ZN_141_ZN_3856

------- SF_5 --------
1n0z_A_ZN_46_ZN_680
1nj3_A_ZN_32_ZN_439
1q5w_A_ZN_32_ZN_1673
2c6a_A_ZN_336_ZN_719
2c6b_A_ZN_336_ZN_719
2cr8_A_ZN_401_ZN_789
2crc_A_ZN_401_ZN_710
2ebq_A_ZN_201_ZN_668
2ebr_A_ZN_201_ZN_638
2ebv_A_ZN_201_ZN_763
2gqe_A_ZN_32_ZN_474
2j9u_B_ZN_1162_ZN_2247
2j9u_D_ZN_1162_ZN_2248
3g9y_A_ZN_1_ZN_397

------- SF_6 --------
1a8h_A_ZN_1000_ZN_4103
1gax_A_ZN_998_ZN_17151
1gax_A_ZN_999_ZN_17152
1gax_B_ZN_998_ZN_17183
1gax_B_ZN_999_ZN_17184
1h3n_A_ZN_1817_ZN_6675
1h3n_A_ZN_1818_ZN_6676
1ile_A_ZN_1101_ZN_6700
1ile_A_ZN_1102_ZN_6701
1irx_A_ZN_601_ZN_8506
1jzq_A_ZN_1101_ZN_6700
1jzq_A_ZN_1102_ZN_6701
1jzs_A_ZN_1101_ZN_6700
1jzs_A_ZN_1102_ZN_6701
1obc_A_ZN_1812_ZN_6633
1obc_A_ZN_1813_ZN_6634
1rqg_A_ZN_801_ZN_4975
1v47_A_ZN_601_ZN_5385
1v47_B_ZN_602_ZN_5420
1woy_A_ZN_601_ZN_4102
2bte_A_ZN_1877_ZN_17619
2bte_A_ZN_1878_ZN_17620
2bte_D_ZN_1877_ZN_17708
2bte_D_ZN_1878_ZN_17709
2byt_A_ZN_1302_ZN_17621
2byt_A_ZN_1303_ZN_17622
2byt_D_ZN_1602_ZN_17669
2byt_D_ZN_1603_ZN_17670
2d54_A_ZN_1501_ZN_4095
2d5b_A_ZN_501_ZN_4102
2v0c_A_ZN_1815_ZN_6641

2v0c_A_ZN_1816_ZN_6642
2v0g_A_ZN_1877_ZN_17553
2v0g_A_ZN_1878_ZN_17554
2v0g_D_ZN_1881_ZN_17607
2znr_A_ZN_1_ZN_1434
2znv_A_ZN_1_ZN_5079
2znv_D_ZN_2_ZN_5088

------- SF_7 --------
1x4j_A_ZN_401_ZN_1123
2ct0_A_ZN_401_ZN_1079
2d8t_A_ZN_401_ZN_1010
2ecj_A_ZN_401_ZN_861
2ecy_A_ZN_401_ZN_883
2l0b_A_ZN_161_ZN_1371
2ysl_A_ZN_401_ZN_1093
3na7_A_ZN_300_ZN_1930
3nw0_A_ZN_2_ZN_3482

------- SF_8 --------
1i3q_A_ZN_2006_ZN_28168
1i3q_B_ZN_2007_ZN_28169
1i50_A_ZN_3006_ZN_28290
1i50_B_ZN_3007_ZN_28293
1i6h_B_ZN_1307_ZN_28440
1k83_A_ZN_3006_ZN_27836
1k83_B_ZN_3007_ZN_27839
1nik_B_ZN_1307_ZN_28303
1r5u_B_ZN_1307_ZN_28304
1r9s_B_ZN_1307_ZN_28467
1r9t_A_ZN_1734_ZN_29221
1r9t_B_ZN_1307_ZN_29223
1sfo_A_ZN_1734_ZN_28651
1sfo_B_ZN_1307_ZN_28653
1twa_A_ZN_3006_ZN_27734
1twa_B_ZN_3007_ZN_27735
1twc_A_ZN_3006_ZN_27717
1twc_B_ZN_3007_ZN_27721
1twf_A_ZN_3006_ZN_28295
1twf_B_ZN_3007_ZN_28296
1twg_A_ZN_3006_ZN_27700
1twg_B_ZN_3007_ZN_27702
1twh_A_ZN_3006_ZN_27705
1twh_B_ZN_3007_ZN_27706
1wcm_A_ZN_2456_ZN_30949
1wcm_B_ZN_2225_ZN_30952
1y1v_A_ZN_1734_ZN_31808
1y1v_B_ZN_1307_ZN_31810
1y1w_B_ZN_1307_ZN_31811
1y77_B_ZN_1307_ZN_31812
2b63_B_ZN_2225_ZN_31739
2b8k_A_ZN_1734_ZN_31045
2b8k_B_ZN_1225_ZN_31047
2e2h_B_ZN_1307_ZN_28977
2e2i_B_ZN_1307_ZN_29667
2e2j_A_ZN_1734_ZN_29178
2e2j_B_ZN_1307_ZN_29180
2ja5_A_ZN_2463_ZN_31672

2ja6_A_ZN_2463_ZN_32024
2ja6_A_ZN_2464_ZN_32025
2ja7_A_ZN_2469_ZN_63949
2ja7_A_ZN_2470_ZN_63950
2ja7_A_ZN_2471_ZN_63951
2ja7_M_ZN_2457_ZN_63953
2ja8_A_ZN_2463_ZN_32013
2ja8_A_ZN_2464_ZN_32014
2nvq_A_ZN_1734_ZN_29372
2nvq_B_ZN_1307_ZN_29374
2nvt_A_ZN_1734_ZN_29140
2nvt_B_ZN_1307_ZN_29142
2nvx_A_ZN_1734_ZN_29385
2nvy_A_ZN_3006_ZN_28295
2nvy_B_ZN_3007_ZN_28296
2nvz_B_ZN_1307_ZN_28979
2pmz_B_ZN_2001_ZN_48123
2pmz_R_ZN_2001_ZN_48128
2r7z_A_ZN_1734_ZN_31811
2r7z_B_ZN_1307_ZN_31813
2r92_A_ZN_1506_ZN_31617
2r92_B_ZN_1307_ZN_31619
2r93_A_ZN_1506_ZN_31505
2r93_B_ZN_1307_ZN_31507
2vum_A_ZN_2456_ZN_32091
2vum_B_ZN_2225_ZN_32094
2waq_A_ZN_1350_ZN_26469
2wb1_A_ZN_1881_ZN_52740
2wb1_B_ZN_2123_ZN_52742
2wb1_R_ZN_2123_ZN_52753
2wb1_W_ZN_1881_ZN_52762
2yu9_A_ZN_1734_ZN_29476
2yu9_B_ZN_1307_ZN_29478
3cqz_A_ZN_3006_ZN_27327
3cqz_B_ZN_3007_ZN_27329
3fki_A_ZN_1734_ZN_31416
3fki_B_ZN_1225_ZN_31418
3gtg_A_ZN_1734_ZN_30071
3gtg_B_ZN_1307_ZN_30073
3gtj_A_ZN_1734_ZN_29979
3gtj_B_ZN_1307_ZN_29981
3gtk_A_ZN_1734_ZN_30117
3gtk_B_ZN_1307_ZN_30119
3gtl_B_ZN_1307_ZN_29263
3gtm_A_ZN_1735_ZN_30566
3gtm_B_ZN_1307_ZN_30568
3gto_B_ZN_1307_ZN_29266
3gtp_B_ZN_1307_ZN_29286
3gtq_B_ZN_1307_ZN_28632
3h0g_A_ZN_2456_ZN_62877
3h0g_B_ZN_2225_ZN_62880
3h0g_M_ZN_2456_ZN_62886
3h0g_N_ZN_2225_ZN_62889
3h3v_B_ZN_1734_ZN_31784
3h3v_C_ZN_1225_ZN_31786
3hkz_A_ZN_1002_ZN_53074
3hkz_B_ZN_2001_ZN_53076
3hkz_I_ZN_1002_ZN_53087

3hkz_J_ZN_2001_ZN_53089
3hou_A_ZN_9984_ZN_63679
3hou_B_ZN_9986_ZN_63681
3hou_M_ZN_9992_ZN_63687
3hou_N_ZN_9994_ZN_63689
3hou_U_ZN_9996_ZN_63691
3hou_U_ZN_9997_ZN_63692
3hov_A_ZN_2456_ZN_31784
3hov_B_ZN_2225_ZN_31787
3how_A_ZN_2456_ZN_31883
3how_B_ZN_2225_ZN_31886
3hox_A_ZN_2456_ZN_31925
3hox_B_ZN_2225_ZN_31928
3hoy_A_ZN_2456_ZN_31810
3hoy_B_ZN_2225_ZN_31813
3hoz_A_ZN_2456_ZN_31968
3hoz_B_ZN_2225_ZN_31971
3i4m_A_ZN_2456_ZN_32363
3i4m_B_ZN_2225_ZN_32365
3i4n_A_ZN_2456_ZN_32315
3i4n_B_ZN_2225_ZN_32317
3k1f_A_ZN_3006_ZN_32337
3k1f_B_ZN_3007_ZN_32339
3k7a_B_ZN_1307_ZN_29034

------- SF_9 --------
1wge_A_ZN_201_ZN_1180
1yop_A_ZN_84_ZN_1259
1yws_A_ZN_83_ZN_1242
2jr7_A_ZN_85_ZN_1276
2jz8_A_ZN_150_ZN_1377
2kpi_A_ZN_150_ZN_838
2odx_A_ZN_156_ZN_829
3gtj_I_ZN_204_ZN_29984
3gtk_I_ZN_204_ZN_30122
3hox_I_ZN_1122_ZN_31931
3hoy_I_ZN_1122_ZN_31816
3hoz_I_ZN_1122_ZN_31974

------- SF_10 --------
1vrq_D_ZN_1006_ZN_12733
1x31_D_ZN_1006_ZN_12679
1x3w_A_ZN_999_ZN_3172
1x3z_A_ZN_999_ZN_3171
2f4m_A_ZN_501_ZN_2956
2f4o_A_ZN_501_ZN_2949
2fe8_A_ZN_316_ZN_7425
2fe8_B_ZN_316_ZN_7434
2fe8_C_ZN_316_ZN_7443
2gag_D_ZN_101_ZN_12661
2gah_D_ZN_101_ZN_12643
2gfo_A_ZN_1200_ZN_2748
2hd5_A_ZN_201_ZN_3129
2ibi_A_ZN_1_ZN_3321
3ad7_D_ZN_100_ZN_12698
3ad8_D_ZN_100_ZN_12715
3ad9_D_ZN_100_ZN_12663
3ada_D_ZN_100_ZN_12640

3esw_A_ZN_344_ZN_3215
3i3t_A_ZN_700_ZN_12328
3i3t_C_ZN_700_ZN_12329
3i3t_E_ZN_700_ZN_12330
3i3t_G_ZN_700_ZN_12331
3mtn_A_ZN_700_ZN_6293
3mtn_C_ZN_700_ZN_6303
3n3k_A_ZN_1_ZN_3491
3nhe_A_ZN_1_ZN_3444

------- SF_11 --------
1hxr_A_ZN_200_ZN_1679
1hxr_B_ZN_201_ZN_1680
1x6m_A_ZN_200_ZN_5939
1x6m_B_ZN_200_ZN_5946
1x6m_C_ZN_200_ZN_5971
1x6m_D_ZN_200_ZN_5989
1xa8_A_ZN_200_ZN_5940
1xa8_B_ZN_200_ZN_5966
1xa8_C_ZN_200_ZN_5998
1xa8_D_ZN_200_ZN_6030
2fu5_A_ZN_502_ZN_4167
2fu5_B_ZN_501_ZN_4172
2qfb_A_ZN_1001_ZN_9991
2qfb_B_ZN_1002_ZN_9992
2qfb_C_ZN_1003_ZN_9993
2qfb_D_ZN_1004_ZN_9994
2qfb_E_ZN_1005_ZN_9995
2qfb_F_ZN_1006_ZN_9996
2qfb_G_ZN_1007_ZN_9997
2qfb_H_ZN_1008_ZN_9998
2qfb_I_ZN_1009_ZN_9999
2qfb_J_ZN_1010_ZN_10000
2rqa_A_ZN_679_ZN_2172
2rqb_A_ZN_1026_ZN_2160
3eqt_A_ZN_1_ZN_2509
3eqt_B_ZN_2_ZN_2510
3ga3_A_ZN_1_ZN_1093
3lrn_A_ZN_1_ZN_2623
3lrn_B_ZN_2_ZN_2624
3lrr_A_ZN_1_ZN_2525
3lrr_B_ZN_2_ZN_2526
3ncu_A_ZN_1_ZN_2505
3ncu_B_ZN_2_ZN_2506
3og8_A_ZN_2_ZN_2699
3og8_B_ZN_1_ZN_2700

------- SF_12 --------
1ee8_A_ZN_501_ZN_4211
1ee8_B_ZN_502_ZN_4212
1k3w_A_ZN_501_ZN_2465
1k3x_A_ZN_501_ZN_2464
1k82_A_ZN_450_ZN_10226
1k82_B_ZN_450_ZN_10227
1k82_C_ZN_450_ZN_10228
1k82_D_ZN_450_ZN_10229
1kfv_A_ZN_300_ZN_5283
1kfv_B_ZN_301_ZN_5290,1kfv_B_ZN_301_ZN_5291

1l1t_A_ZN_300_ZN_2442
1l1z_A_ZN_300_ZN_2465
1l2b_A_ZN_300_ZN_2466
1l2c_A_ZN_300_ZN_2441
1l2d_A_ZN_300_ZN_2444
1nnj_A_ZN_300_ZN_2743
1pji_A_ZN_300_ZN_2766
1pjj_A_ZN_300_ZN_2740
1pm5_A_ZN_300_ZN_2795
1q39_A_ZN_301_ZN_2028
1q3b_A_ZN_301_ZN_2193
1q3c_A_ZN_301_ZN_2067
1r2y_A_ZN_300_ZN_2599
1r2z_A_ZN_320_ZN_2593
1tdz_A_ZN_1001_ZN_2701
1xc8_A_ZN_5001_ZN_2701
2ea0_A_ZN_501_ZN_2583
2f5n_A_ZN_300_ZN_2511
2f5o_A_ZN_300_ZN_2543
2f5p_A_ZN_300_ZN_2520
2f5q_A_ZN_300_ZN_2524
2f5s_A_ZN_300_ZN_2579
2opf_A_ZN_501_ZN_2635
2oq4_A_ZN_501_ZN_4871
2oq4_B_ZN_501_ZN_4877
3c58_A_ZN_401_ZN_2727
3go8_A_ZN_300_ZN_2564
3gp1_A_ZN_300_ZN_2518
3gpp_A_ZN_300_ZN_2492
3gpu_A_ZN_300_ZN_2514
3gpx_A_ZN_300_ZN_2473
3gpy_A_ZN_300_ZN_2781
3gq3_A_ZN_300_ZN_2535
3gq4_A_ZN_300_ZN_2625
3gq5_A_ZN_300_ZN_2421
3jr4_A_ZN_300_ZN_2489
3jr5_A_ZN_300_ZN_2735

------- SF_13 --------
1m2o_A_ZN_800_ZN_13889
1m2o_C_ZN_800_ZN_13923
1m2v_A_ZN_800_ZN_11509
1m2v_B_ZN_950_ZN_11510
1pcx_A_ZN_950_ZN_5954
1pd0_A_ZN_950_ZN_5902
1pd1_A_ZN_950_ZN_5895
2nup_A_ZN_800_ZN_12423
2nup_B_ZN_1100_ZN_12424
2nut_A_ZN_800_ZN_12479
2nut_B_ZN_1100_ZN_12480
2qtv_A_ZN_800_ZN_7358
2yrc_A_ZN_200_ZN_879
2yrd_A_ZN_200_ZN_873
3efo_A_ZN_766_ZN_11779
3efo_B_ZN_1034_ZN_11780
3eg9_A_ZN_800_ZN_11851
3eg9_B_ZN_1100_ZN_11852
3egd_A_ZN_765_ZN_12594

3egd_B_ZN_1094_ZN_12595
3egx_A_ZN_765_ZN_12526
3egx_B_ZN_1094_ZN_12527
3eh1_A_ZN_1269_ZN_5816
3eh2_A_ZN_800_ZN_17551
3eh2_B_ZN_800_ZN_17552
3eh2_C_ZN_800_ZN_17553

------- SF_14 --------
1i3q_L_ZN_2005_ZN_28167
1i50_L_ZN_3005_ZN_28298
1i6h_L_ZN_105_ZN_28438
1k83_L_ZN_3005_ZN_27844
1r5u_L_ZN_105_ZN_28309
1r9s_L_ZN_105_ZN_28472
1r9t_L_ZN_105_ZN_29228
1sfo_L_ZN_105_ZN_28658
1twa_L_ZN_3005_ZN_27733
1twc_L_ZN_3005_ZN_27758
1twf_L_ZN_3005_ZN_28294
1twg_L_ZN_3005_ZN_27736
1twh_L_ZN_3005_ZN_27704
1wcm_L_ZN_1071_ZN_30957
1y1v_L_ZN_105_ZN_31815
1y1w_L_ZN_105_ZN_31816
1y77_L_ZN_105_ZN_31817
2b63_L_ZN_1071_ZN_31744
2b8k_L_ZN_71_ZN_31052
2e2h_L_ZN_105_ZN_28982
2e2i_L_ZN_105_ZN_29672
2e2j_L_ZN_105_ZN_29185
2ja5_A_ZN_2459_ZN_31668
2ja6_A_ZN_2459_ZN_32020
2ja7_A_ZN_2461_ZN_63941
2ja7_A_ZN_2462_ZN_63942
2ja8_A_ZN_2459_ZN_32009
2nvq_L_ZN_105_ZN_29379
2nvt_L_ZN_105_ZN_29147
2nvx_L_ZN_105_ZN_29392
2nvy_L_ZN_3005_ZN_28294
2nvz_L_ZN_105_ZN_28984
2pmz_P_ZN_1001_ZN_48125
2pmz_Z_ZN_1001_ZN_48130
2r7z_L_ZN_105_ZN_31818
2r92_L_ZN_105_ZN_31624
2r93_L_ZN_105_ZN_31512
2vum_L_ZN_1071_ZN_32099
2waq_P_ZN_50_ZN_26484
2wb1_P_ZN_1049_ZN_52752
2wb1_X_ZN_1049_ZN_52764
2yu9_L_ZN_105_ZN_29483
3cqz_L_ZN_3005_ZN_27334
3fki_L_ZN_71_ZN_31423
3gtg_L_ZN_105_ZN_30078
3gtj_L_ZN_105_ZN_29986
3gtk_L_ZN_105_ZN_30124
3gtl_L_ZN_105_ZN_29268
3gtm_L_ZN_105_ZN_30573

3gto_L_ZN_105_ZN_29271
3gtp_L_ZN_105_ZN_29291
3gtq_L_ZN_105_ZN_28637
3h0g_L_ZN_1071_ZN_62885
3h0g_X_ZN_1071_ZN_62894
3h3v_M_ZN_71_ZN_31791
3hou_L_ZN_9991_ZN_63686
3hou_X_ZN_9999_ZN_63694
3hov_L_ZN_1071_ZN_31792
3how_L_ZN_1071_ZN_31891
3hox_L_ZN_1071_ZN_31933
3hoy_L_ZN_1071_ZN_31818
3hoz_L_ZN_1071_ZN_31976
3i4m_L_ZN_1071_ZN_32370
3i4n_L_ZN_1071_ZN_32322
3k1f_L_ZN_3005_ZN_32344
3k7a_L_ZN_105_ZN_29039
3lrq_A_ZN_101_ZN_2719
3lrq_B_ZN_101_ZN_2721
3lrq_C_ZN_101_ZN_2723
3lrq_D_ZN_101_ZN_2725

------- SF_15 --------
2k8d_A_ZN_155_ZN_2368
2kv1_A_ZN_125_ZN_1878
2l1u_A_ZN_144_ZN_2188
3cez_A_ZN_201_ZN_2041
3cez_B_ZN_201_ZN_2046
3cxk_A_ZN_201_ZN_2085
3cxk_B_ZN_201_ZN_2090
3hci_A_ZN_1000_ZN_2353
3hci_B_ZN_1000_ZN_2368
3hcj_A_ZN_1000_ZN_2435
3hcj_B_ZN_1000_ZN_2436

------- SF_16 --------
2ecv_A_ZN_401_ZN_1252
2yu4_A_ZN_201_ZN_1482
3hcu_A_ZN_302_ZN_4065
3hcu_C_ZN_305_ZN_4068
3htk_C_ZN_268_ZN_3108
3i2d_A_ZN_1_ZN_2237

------- SF_17 --------
1vk6_A_ZN_301_ZN_2074
2d74_B_ZN_1002_ZN_4220
2dcu_B_ZN_3002_ZN_4283
2e9h_A_ZN_301_ZN_2431
2gb5_A_ZN_301_ZN_4219
2gb5_B_ZN_301_ZN_4220
2qmu_C_ZN_414_ZN_4993

------- SF_18 --------
1kjz_A_ZN_411_ZN_3050
1kk0_A_ZN_411_ZN_3042
1kk1_A_ZN_411_ZN_3017
1kk2_A_ZN_411_ZN_3019
1kk3_A_ZN_411_ZN_3029

1p3j_A_ZN_218_ZN_1672
1s0u_A_ZN_1_ZN_2838
1s3g_A_ZN_219_ZN_1686
1zin_A_ZN_219_ZN_1689
1zio_A_ZN_219_ZN_1689
1zip_A_ZN_219_ZN_1689
2d74_A_ZN_1001_ZN_4219
2dcu_A_ZN_3001_ZN_4254
2eu8_A_ZN_220_ZN_3369
2eu8_B_ZN_221_ZN_3428
2oo7_A_ZN_920_ZN_3360
2oo7_B_ZN_921_ZN_3419
2ori_A_ZN_420_ZN_3371
2ori_B_ZN_421_ZN_3430
2osb_A_ZN_1220_ZN_3367
2osb_B_ZN_1221_ZN_3426
2p3s_A_ZN_320_ZN_1691
2qaj_A_ZN_620_ZN_3377
2qaj_B_ZN_621_ZN_3436
2xb4_A_ZN_1224_ZN_1734
3dkv_A_ZN_218_ZN_1693
3dl0_A_ZN_219_ZN_3460
3dl0_B_ZN_219_ZN_3519
3fb4_A_ZN_217_ZN_1675
3kb1_A_ZN_302_ZN_3794

------- SF_19 --------
1z60_A_ZN_2_ZN_879
2ckl_B_ZN_1115_ZN_1568
2djb_A_ZN_401_ZN_1069
2eci_A_ZN_401_ZN_1317
2jmd_A_ZN_66_ZN_977

------- SF_20 --------
2ea6_A_ZN_401_ZN_997
2ecm_A_ZN_401_ZN_787
2kiz_A_ZN_71_ZN_1080
2ysj_A_ZN_401_ZN_917

------- SF_21 --------
2wwz_C_ZN_1694_ZN_1515
2wx0_C_ZN_1694_ZN_2800
2wx0_G_ZN_1694_ZN_2801
3a9j_C_ZN_1_ZN_1504
3a9k_C_ZN_1_ZN_1472
3ch5_B_ZN_800_ZN_1920
3gj3_B_ZN_300_ZN_1856
3gj4_B_ZN_300_ZN_3626
3gj4_D_ZN_300_ZN_3656
3gj5_B_ZN_300_ZN_3628
3gj5_D_ZN_300_ZN_3658
3gj6_B_ZN_300_ZN_1853
3gj7_B_ZN_300_ZN_3661
3gj7_D_ZN_300_ZN_3693
3gj8_B_ZN_300_ZN_3687
3gj8_D_ZN_300_ZN_3718

------- SF_22 --------

1k81_A_ZN_144_ZN_587,1k81_A_ZN_144_ZN_588
1nee_A_ZN_136_ZN_2205
1y77_I_ZN_203_ZN_31814
2j6a_A_ZN_1138_ZN_1071

------- SF_23 --------
1acm_B_ZN_154_ZN_7115
1acm_D_ZN_154_ZN_7132
1at1_B_ZN_154_ZN_7125
1at1_D_ZN_154_ZN_7126
1d09_B_ZN_1313_ZN_7253
1d09_D_ZN_1314_ZN_7270
1ezz_B_ZN_313_ZN_7233
1ezz_D_ZN_314_ZN_7234
1f1b_B_ZN_313_ZN_7249
1f1b_D_ZN_314_ZN_7266
1i5o_B_ZN_309_ZN_7225
1i5o_D_ZN_409_ZN_7242
1nbe_B_ZN_154_ZN_7251
1nbe_D_ZN_154_ZN_7270
1pg5_B_ZN_500_ZN_3502
1q95_G_ZN_2002_ZN_21805
1q95_H_ZN_2001_ZN_21806
1q95_I_ZN_2003_ZN_21807
1q95_J_ZN_2004_ZN_21808
1q95_K_ZN_2005_ZN_21809
1q95_L_ZN_2006_ZN_21810
1r0b_G_ZN_1001_ZN_21763
1r0b_H_ZN_1002_ZN_21764
1r0b_I_ZN_1003_ZN_21765
1r0b_J_ZN_1004_ZN_21820
1r0b_K_ZN_1005_ZN_21821
1r0b_L_ZN_1006_ZN_21822
1r0c_B_ZN_1001_ZN_7254
1r0c_H_ZN_1002_ZN_7272
1raa_B_ZN_154_ZN_7227
1raa_D_ZN_154_ZN_7257
1rab_B_ZN_154_ZN_7227
1rab_D_ZN_154_ZN_7257
1rac_B_ZN_154_ZN_7227
1rac_D_ZN_154_ZN_7257
1rad_B_ZN_154_ZN_7227
1rad_D_ZN_154_ZN_7257
1rae_B_ZN_154_ZN_7227
1rae_D_ZN_154_ZN_7257
1raf_B_ZN_154_ZN_7227
1raf_D_ZN_154_ZN_7257
1rag_B_ZN_154_ZN_7227
1rag_D_ZN_154_ZN_7257
1rah_B_ZN_154_ZN_7227
1rah_D_ZN_154_ZN_7257
1rai_B_ZN_154_ZN_7174
1rai_D_ZN_154_ZN_7204
1sku_B_ZN_315_ZN_7159
1sku_D_ZN_314_ZN_7167
1tth_B_ZN_154_ZN_7244
1tth_D_ZN_155_ZN_7261
1tu0_B_ZN_154_ZN_7237

1tu0_D_ZN_155_ZN_7246
1tug_B_ZN_154_ZN_7244
1tug_D_ZN_155_ZN_7289
1xjw_B_ZN_1313_ZN_7245
1xjw_D_ZN_1314_ZN_7262
1za1_B_ZN_154_ZN_7220
1za1_D_ZN_154_ZN_7250
1za2_B_ZN_154_ZN_7106
1za2_D_ZN_154_ZN_7152
2a0f_B_ZN_154_ZN_7113
2a0f_D_ZN_154_ZN_7114
2air_B_ZN_800_ZN_7255
2air_H_ZN_801_ZN_7274
2at1_B_ZN_154_ZN_7125
2at1_D_ZN_154_ZN_7126
2be7_D_ZN_1108_ZN_9927
2be7_E_ZN_1108_ZN_9928
2be7_F_ZN_1108_ZN_9929
2be9_B_ZN_1113_ZN_3497
2fzc_B_ZN_154_ZN_7235
2fzc_D_ZN_154_ZN_7283
2fzg_B_ZN_154_ZN_7243
2fzg_D_ZN_154_ZN_7295
2fzk_B_ZN_154_ZN_7246
2fzk_D_ZN_154_ZN_7301
2h3e_B_ZN_159_ZN_7119
2h3e_D_ZN_159_ZN_7136
2hse_B_ZN_954_ZN_7137
2hse_D_ZN_955_ZN_7155
2ipo_B_ZN_201_ZN_7253
2ipo_D_ZN_201_ZN_7286
2qg9_B_ZN_160_ZN_7231
2qg9_D_ZN_160_ZN_7232
2qgf_B_ZN_160_ZN_7227
2qgf_D_ZN_160_ZN_7228
2yww_A_ZN_504_ZN_2244
2yww_B_ZN_503_ZN_2308
3at1_B_ZN_154_ZN_7111
3at1_D_ZN_154_ZN_7112
3d7s_B_ZN_313_ZN_7237
3d7s_D_ZN_314_ZN_7238
3mpu_B_ZN_154_ZN_10613
3mpu_D_ZN_154_ZN_10624
3mpu_F_ZN_154_ZN_10635
4at1_B_ZN_154_ZN_7111
4at1_D_ZN_154_ZN_7112
5at1_B_ZN_154_ZN_7111
5at1_D_ZN_154_ZN_7112
6at1_B_ZN_154_ZN_7111
6at1_D_ZN_154_ZN_7112
7at1_B_ZN_154_ZN_7125
7at1_D_ZN_154_ZN_7126
8at1_B_ZN_154_ZN_7125
8at1_D_ZN_154_ZN_7126
8atc_B_ZN_154_ZN_7127
8atc_D_ZN_154_ZN_7144

------- SF_24 --------

2ayj_A_ZN_57_ZN_956
2k4x_A_ZN_56_ZN_900
2zjp_Y_ZN_61_ZN_84391
3dll_Y_ZN_61_ZN_83627
3hux_5_ZN_61_ZN_90791
3huz_5_ZN_61_ZN_90531
3kni_5_ZN_61_ZN_90945
3knk_5_ZN_61_ZN_90779
3knm_5_ZN_61_ZN_90731
3kno_5_ZN_61_ZN_90401

------- SF_25 --------
1d0c_A_ZN_900_ZN_6606
1d0o_A_ZN_900_ZN_6604
1d1v_A_ZN_900_ZN_6604
1d1w_A_ZN_900_ZN_6604
1d1x_A_ZN_901_ZN_6604
1d1y_B_ZN_900_ZN_6692
1df1_A_ZN_900_ZN_6849
1df1_B_ZN_900_ZN_6916
1dm6_A_ZN_900_ZN_6607
1dm7_B_ZN_2900_ZN_6684
1dm8_A_ZN_900_ZN_6607
1dmi_A_ZN_900_ZN_6607
1dmj_A_ZN_900_ZN_6607
1dmk_A_ZN_900_ZN_6611
1dwv_A_ZN_903_ZN_6911
1dwv_B_ZN_903_ZN_7005
1dww_A_ZN_903_ZN_6911
1dww_B_ZN_903_ZN_7005
1ed4_A_ZN_900_ZN_6604
1ed5_B_ZN_900_ZN_6677
1ed6_A_ZN_900_ZN_6600
1foi_A_ZN_900_ZN_6603
1foj_A_ZN_900_ZN_6607
1fol_B_ZN_900_ZN_6671
1foo_A_ZN_900_ZN_6603
1fop_B_ZN_900_ZN_6690
1i83_A_ZN_900_ZN_6610
1k2r_B_ZN_900_ZN_6740
1k2s_A_ZN_900_ZN_6661
1k2t_A_ZN_900_ZN_6661
1k2u_A_ZN_900_ZN_6661
1lzx_A_ZN_900_ZN_6660
1lzz_A_ZN_900_ZN_6660
1m00_A_ZN_900_ZN_6660
1m9j_A_ZN_903_ZN_6371
1m9k_A_ZN_903_ZN_6371
1m9m_A_ZN_903_ZN_6357
1m9q_A_ZN_903_ZN_6357
1m9r_A_ZN_904_ZN_6371
1mmv_A_ZN_900_ZN_6654
1mmw_A_ZN_900_ZN_6661
1n2n_A_ZN_950_ZN_6835
1n2n_B_ZN_950_ZN_6907
1nse_A_ZN_900_ZN_6607
1nsi_A_ZN_901_ZN_13696
1nsi_C_ZN_902_ZN_13873

1om4_A_ZN_900_ZN_6661
1om5_A_ZN_900_ZN_6661
1p6h_A_ZN_900_ZN_6661
1p6i_A_ZN_900_ZN_6661
1p6j_A_ZN_900_ZN_6661
1p6k_A_ZN_900_ZN_6700
1p6l_B_ZN_900_ZN_6521
1p6m_B_ZN_900_ZN_6513
1p6n_B_ZN_900_ZN_6541
1q2o_A_ZN_900_ZN_6449
1qw4_A_ZN_950_ZN_6833
1qw4_B_ZN_950_ZN_6909
1qw5_A_ZN_950_ZN_6833
1qw5_B_ZN_950_ZN_6907
1qw6_A_ZN_950_ZN_3420
1qwc_A_ZN_950_ZN_3420
1rs6_A_ZN_900_ZN_6677
1rs7_A_ZN_900_ZN_6661
1rs8_A_ZN_900_ZN_6453
1rs9_B_ZN_900_ZN_6552
1vaf_A_ZN_950_ZN_6833
1vaf_B_ZN_950_ZN_6944
1vag_A_ZN_950_ZN_3420
1zvi_A_ZN_902_ZN_3395
1zvl_A_ZN_950_ZN_6839
1zzq_A_ZN_900_ZN_6688
1zzr_A_ZN_900_ZN_6659
1zzs_A_ZN_900_ZN_6452
1zzt_A_ZN_900_ZN_6454
1zzu_A_ZN_900_ZN_6678
2g6h_A_ZN_900_ZN_6665
2g6i_A_ZN_900_ZN_6683
2g6j_A_ZN_900_ZN_6683
2g6k_A_ZN_900_ZN_6683
2g6l_A_ZN_900_ZN_6683
2g6m_A_ZN_900_ZN_6665
2g6n_A_ZN_900_ZN_6683
2g6o_A_ZN_900_ZN_6609
2hx2_A_ZN_900_ZN_6418
2hx3_A_ZN_900_ZN_6665
2hx4_A_ZN_900_ZN_6665
2nse_B_ZN_900_ZN_6670
3b3m_A_ZN_900_ZN_6747
3b3n_A_ZN_900_ZN_6742
3b3o_A_ZN_900_ZN_6769
3b3p_A_ZN_900_ZN_6769
3dqr_B_ZN_900_ZN_6821
3dqs_A_ZN_900_ZN_6525
3dqt_B_ZN_900_ZN_6640
3e7g_A_ZN_3001_ZN_13699
3e7g_C_ZN_3002_ZN_13858
3e7i_A_ZN_904_ZN_6856
3e7i_B_ZN_2903_ZN_6951
3e7m_B_ZN_1900_ZN_6901
3e7s_A_ZN_903_ZN_6376
3eah_A_ZN_862_ZN_6374
3ej8_A_ZN_1903_ZN_13729
3ej8_C_ZN_3903_ZN_13860

3fc5_A_ZN_900_ZN_6708
3hsn_A_ZN_900_ZN_6900
3hso_A_ZN_900_ZN_6863
3hsp_A_ZN_900_ZN_6835
3jt3_A_ZN_900_ZN_6739
3jt4_A_ZN_900_ZN_6784
3jt5_A_ZN_900_ZN_6752
3jt6_A_ZN_900_ZN_6753
3jt7_A_ZN_900_ZN_6766
3jt8_A_ZN_900_ZN_6769
3jt9_A_ZN_900_ZN_6778
3jta_A_ZN_900_ZN_6753
3jws_A_ZN_900_ZN_6746
3jwt_A_ZN_900_ZN_6746
3jwu_A_ZN_900_ZN_6749
3jwv_A_ZN_900_ZN_6746
3jww_A_ZN_900_ZN_6558
3jwx_A_ZN_900_ZN_6575
3jwy_A_ZN_900_ZN_6513
3jwz_B_ZN_900_ZN_6624
3jx0_A_ZN_900_ZN_6753
3jx1_A_ZN_900_ZN_6747
3jx2_A_ZN_900_ZN_6774
3jx3_A_ZN_900_ZN_6750
3jx4_A_ZN_900_ZN_6750
3jx5_A_ZN_900_ZN_6750
3jx6_A_ZN_900_ZN_6736
3n2r_A_ZN_900_ZN_6764
3nld_A_ZN_900_ZN_6514
3nle_A_ZN_900_ZN_6521
3nlf_A_ZN_900_ZN_6521
3nlg_A_ZN_900_ZN_6528
3nlh_A_ZN_900_ZN_6513
3nli_A_ZN_900_ZN_6532
3nlk_A_ZN_900_ZN_6746
3nlm_A_ZN_900_ZN_6796
3nln_A_ZN_900_ZN_6746
3nlo_A_ZN_900_ZN_6754
3nlp_A_ZN_900_ZN_6762
3nlq_A_ZN_900_ZN_6760
3nlr_A_ZN_900_ZN_6750
3nny_A_ZN_900_ZN_6750
3nnz_A_ZN_900_ZN_6763
3nos_A_ZN_1000_ZN_6399
3nse_B_ZN_900_ZN_6691
4nos_A_ZN_3000_ZN_13685
4nos_C_ZN_2000_ZN_13818
4nse_B_ZN_900_ZN_6677
5nse_A_ZN_900_ZN_6600
6nse_A_ZN_900_ZN_6600
7nse_B_ZN_900_ZN_6676
8nse_A_ZN_900_ZN_6596
9nse_A_ZN_900_ZN_6604

------- SF_26 --------
1dgs_A_ZN_701_ZN_9389
1dgs_B_ZN_2701_ZN_9412
1v9p_A_ZN_701_ZN_9487

1v9p_B_ZN_2701_ZN_9510
2owo_A_ZN_672_ZN_5655

------- SF_27 --------
2f9i_B_ZN_601_ZN_8690
2f9i_D_ZN_602_ZN_8691
2f9y_B_ZN_305_ZN_4261

------- SF_28 --------
2i14_A_ZN_391_ZN_18337
2i14_B_ZN_791_ZN_18361
2i14_C_ZN_1391_ZN_18385
2i14_D_ZN_1791_ZN_18409
2i14_E_ZN_2391_ZN_18433
2i14_F_ZN_2791_ZN_18457
2i1o_A_ZN_701_ZN_3043,2i1o_A_ZN_702_ZN_3044

------- SF_29 --------
2kkr_A_ZN_500_ZN_987
2kkt_A_ZN_500_ZN_840

------- SF_30 --------
1fre_@_ZN_43_ZN_413

------- SF_31 --------
1odh_A_ZN_1172_ZN_1809

------- SF_32 --------
1p91_A_ZN_1301_ZN_4179
1p91_B_ZN_2301_ZN_4212

------- SF_33 --------
1f4l_A_ZN_701_ZN_4448
1med_A_ZN_29_ZN_392
1p7p_A_ZN_552_ZN_4378
1pfu_A_ZN_552_ZN_4396
1pfv_A_ZN_552_ZN_4392
1pfw_A_ZN_552_ZN_4391
1pfy_A_ZN_552_ZN_4392
1qqt_A_ZN_552_ZN_4383
3h97_A_ZN_601_ZN_4400
3h99_A_ZN_601_ZN_4397
3h9b_A_ZN_601_ZN_4384
3h9c_A_ZN_601_ZN_4377

------- SF_34 --------
1jwh_C_ZN_357_ZN_9000
1jwh_D_ZN_358_ZN_9016
1qf8_A_ZN_216_ZN_2707
1qf8_B_ZN_216_ZN_2709
1rqf_A_ZN_179_ZN_11146
1rqf_B_ZN_179_ZN_11147
1rqf_D_ZN_201_ZN_11148
1rqf_E_ZN_202_ZN_11149
1rqf_G_ZN_301_ZN_11156
1rqf_H_ZN_302_ZN_11163
1rqf_J_ZN_401_ZN_11164
1rqf_K_ZN_402_ZN_11165

2r6m_A_ZN_216_ZN_2892
2r6m_B_ZN_216_ZN_2893
3eed_A_ZN_194_ZN_3003
3eed_B_ZN_194_ZN_3014

------- SF_35 --------
1t8h_A_ZN_275_ZN_2098

------- SF_36 --------
1zt2_A_ZN_999_ZN_8595
1zt2_C_ZN_999_ZN_8661

------- SF_37 --------
1smy_D_ZN_9102_ZN_53708
1smy_N_ZN_9104_ZN_53927
1ynj_D_ZN_1525_ZN_24374
1zyr_D_ZN_9001_ZN_54055
2a68_D_ZN_7058_ZN_53765
2a68_N_ZN_7059_ZN_54121
2a69_D_ZN_7058_ZN_53759
2a69_N_ZN_7059_ZN_54064
2a6e_D_ZN_7058_ZN_53569
2a6e_N_ZN_7059_ZN_53572
2a6h_D_ZN_7458_ZN_53474
2a6h_N_ZN_7459_ZN_53520
2be5_D_ZN_9058_ZN_53570
2be5_N_ZN_9059_ZN_53599
2cw0_D_ZN_1525_ZN_53969
2cw0_N_ZN_1525_ZN_53971
2o5i_D_ZN_4058_ZN_49167
2o5i_N_ZN_5058_ZN_49170
2o5j_D_ZN_4058_ZN_48527
2o5j_N_ZN_5058_ZN_48562
2ppb_D_ZN_7058_ZN_49396
2ppb_N_ZN_7158_ZN_49505
3dxj_D_ZN_1525_ZN_56083
3dxj_N_ZN_1525_ZN_56099
3eql_D_ZN_1525_ZN_52791
3eql_N_ZN_1525_ZN_52824

------- SF_38 --------
2apo_B_ZN_501_ZN_2846
2aqc_A_ZN_100_ZN_1067
2aus_B_ZN_1070_ZN_5797
2aus_D_ZN_1071_ZN_5786
2ey4_E_ZN_201_ZN_7285
2ey4_F_ZN_301_ZN_7286
2hvy_C_ZN_201_ZN_5784
3hax_C_ZN_201_ZN_5560
3hjw_B_ZN_1_ZN_5485

------- SF_39 --------
2ba1_A_ZN_201_ZN_15664
2ba1_B_ZN_202_ZN_15666
2ba1_C_ZN_203_ZN_15665
3m7n_A_ZN_180_ZN_16153
3m7n_B_ZN_180_ZN_16154
3m7n_C_ZN_180_ZN_16155

3m85_A_ZN_180_ZN_16114
3m85_B_ZN_180_ZN_16115
3m85_C_ZN_180_ZN_16116

------- SF_40 --------
2d8s_A_ZN_401_ZN_1148

------- SF_41 --------
2dkt_A_ZN_291_ZN_2166

------- SF_42 --------
2exu_A_ZN_501_ZN_1505

------- SF_43 --------
2hdp_A_ZN_493_ZN_1998
2hdp_B_ZN_493_ZN_2000

------- SF_44 --------
2hu9_A_ZN_132_ZN_2069
2hu9_B_ZN_132_ZN_2078

------- SF_45 --------
2jne_A_ZN_150_ZN_1090
2jne_A_ZN_200_ZN_1091
2jrp_A_ZN_150_ZN_1245
2jrp_A_ZN_200_ZN_1246

------- SF_46 --------
2jox_A_ZN_108_ZN_1639,2jox_A_ZN_109_ZN_1640

------- SF_47 --------
2k5c_A_ZN_96_ZN_1469

------- SF_48 --------
2r6f_A_ZN_1004_ZN_13925
2r6f_B_ZN_1007_ZN_13982

------- SF_49 --------
2rf5_A_ZN_1_ZN_1666
3kr7_A_ZN_1_ZN_1687
3kr8_A_ZN_1_ZN_3325
3kr8_C_ZN_1_ZN_3363
3mhj_A_ZN_1_ZN_3394
3mhj_B_ZN_1_ZN_3425
3mhk_A_ZN_1_ZN_1688
3p0n_A_ZN_1_ZN_3349
3p0n_C_ZN_1_ZN_3380
3p0p_A_ZN_1_ZN_3320
3p0p_C_ZN_1_ZN_3352
3p0q_A_ZN_1_ZN_3347
3p0q_C_ZN_1_ZN_3379

------- SF_50 --------
2jvn_A_ZN_359_ZN_2064,2jvn_A_ZN_400_ZN_2064
2riq_A_ZN_1_ZN_1066

------- SF_51 --------
2xoc_A_ZN_992_ZN_3435,2xoc_A_ZN_993_ZN_3436

2xoc_B_ZN_992_ZN_3477,2xoc_B_ZN_993_ZN_3478
2xoy_A_ZN_992_ZN_3388,2xoy_A_ZN_993_ZN_3389
2xoy_B_ZN_992_ZN_3393,2xoy_B_ZN_993_ZN_3394
2xoz_A_ZN_992_ZN_3423,2xoz_A_ZN_993_ZN_3424
2xoz_B_ZN_992_ZN_3428,2xoz_B_ZN_993_ZN_3429
2xp0_A_ZN_992_ZN_3410,2xp0_A_ZN_993_ZN_3411
2xp0_B_ZN_992_ZN_3415,2xp0_B_ZN_993_ZN_3416

------- SF_52 --------
1x0t_A_ZN_150_ZN_907
2k3r_A_ZN_124_ZN_1611
2ki7_B_ZN_124_ZN_3591
2zae_B_ZN_121_ZN_3407
2zae_D_ZN_121_ZN_3430

------- SF_53 --------
3bvo_A_ZN_301_ZN_3099
3bvo_B_ZN_301_ZN_3100

------- SF_54 --------
3cng_A_ZN_508_ZN_5811
3cng_B_ZN_509_ZN_5822
3cng_C_ZN_510_ZN_5836
3cng_D_ZN_511_ZN_5848

------- SF_55 --------
3e9s_A_ZN_318_ZN_2520
3mj5_A_ZN_901_ZN_4557
3mj5_B_ZN_901_ZN_4589

------- SF_56 --------
3epz_A_ZN_701_ZN_3449
3epz_B_ZN_701_ZN_3469

------- SF_57 --------
3f2b_A_ZN_4_ZN_8465
3f2c_A_ZN_4_ZN_8434
3f2d_A_ZN_4_ZN_8434

------- SF_58 --------
3flo_B_ZN_1_ZN_19830
3flo_D_ZN_1_ZN_19917
3flo_F_ZN_1_ZN_19999
3flo_H_ZN_1_ZN_20076

------- SF_59 --------
3flo_B_ZN_2_ZN_19831
3flo_D_ZN_2_ZN_19918
3flo_F_ZN_2_ZN_20000
3flo_H_ZN_2_ZN_20077

------- SF_60 --------
3ir9_A_ZN_501_ZN_2441
3ir9_B_ZN_501_ZN_2442

------- SF_61 --------
3irb_A_ZN_201_ZN_2190
3irb_B_ZN_201_ZN_2211

------- SF_62 --------
1bor_A_ZN_57_ZN_423
1chc_A_ZN_71_ZN_1044
1dcq_A_ZN_600_ZN_2075
1dvp_A_ZN_401_ZN_1730
1dvp_A_ZN_402_ZN_1731
1e4u_A_ZN_79_ZN_1231
1f62_A_ZN_52_ZN_795
1f62_A_ZN_53_ZN_796
1fbv_A_ZN_1001_ZN_4370
1fp0_A_ZN_89_ZN_1318
1fp0_A_ZN_90_ZN_1319
1g25_A_ZN_66_ZN_1001
1hyi_A_ZN_66_ZN_985
1hyi_A_ZN_67_ZN_986
1hyj_A_ZN_66_ZN_985
1hyj_A_ZN_67_ZN_986
1iym_A_ZN_182_ZN_795
1jm7_A_ZN_123_ZN_3161
1jm7_B_ZN_143_ZN_3163
1joc_A_ZN_300_ZN_1928
1joc_A_ZN_301_ZN_1929
1joc_B_ZN_302_ZN_1950
1joc_B_ZN_303_ZN_1951
1ldj_B_ZN_201_ZN_6668
1ldk_C_ZN_4001_ZN_7925
1mm2_A_ZN_62_ZN_896
1mm2_A_ZN_63_ZN_897
1mm3_A_ZN_62_ZN_903
1mm3_A_ZN_63_ZN_904
1rmd_A_ZN_117_ZN_913,1rmd_A_ZN_118_ZN_914
1tot_A_ZN_54_ZN_829
1u5k_A_ZN_300_ZN_3589
1u5k_B_ZN_300_ZN_3590
1u6g_B_ZN_1229_ZN_15494
1ur6_B_ZN_79_ZN_3015
1v87_A_ZN_201_ZN_1677
1vfy_A_ZN_300_ZN_534
1vfy_A_ZN_301_ZN_535
1vyx_A_ZN_1061_ZN_915
1w3s_A_ZN_1238_ZN_3535
1w3s_B_ZN_1239_ZN_3536
1wd2_A_ZN_71_ZN_930
1we9_A_ZN_201_ZN_895
1we9_A_ZN_401_ZN_896
1wee_A_ZN_201_ZN_1016
1wee_A_ZN_401_ZN_1017
1wem_A_ZN_201_ZN_1101
1wem_A_ZN_401_ZN_1102
1wen_A_ZN_201_ZN_1000
1wen_A_ZN_401_ZN_1001
1weo_A_ZN_201_ZN_1344
1wep_A_ZN_201_ZN_1134
1wep_A_ZN_401_ZN_1135
1weq_A_ZN_201_ZN_1240
1weq_A_ZN_401_ZN_1241
1wes_A_ZN_201_ZN_1019

1wes_A_ZN_401_ZN_1020
1weu_A_ZN_201_ZN_1284
1weu_A_ZN_401_ZN_1285
1wev_A_ZN_201_ZN_1307
1wev_A_ZN_401_ZN_1308
1wew_A_ZN_201_ZN_1123
1wew_A_ZN_401_ZN_1124
1wfk_A_ZN_200_ZN_1271
1wfk_A_ZN_400_ZN_1272
1wil_A_ZN_201_ZN_1276
1wil_A_ZN_401_ZN_1277
1wim_A_ZN_201_ZN_1405
1x4i_A_ZN_201_ZN_996
1x4i_A_ZN_401_ZN_997
1x4u_A_ZN_201_ZN_1162
1x4u_A_ZN_401_ZN_1163
1xwh_A_ZN_355_ZN_955
1xwh_A_ZN_356_ZN_956
1y02_A_ZN_161_ZN_726
1y02_A_ZN_162_ZN_727
1zbd_B_ZN_300_ZN_2479
1zbd_B_ZN_301_ZN_2480
2a20_A_ZN_292_ZN_907
2a20_A_ZN_308_ZN_908
2b0o_E_ZN_698_ZN_7993
2b0o_F_ZN_698_ZN_7994
2b0o_G_ZN_698_ZN_7995
2b0o_H_ZN_698_ZN_7996
2b9d_A_ZN_1002_ZN_803
2b9d_B_ZN_1001_ZN_804
2cjs_C_ZN_201_ZN_3190
2cjs_C_ZN_202_ZN_3191
2ckl_A_ZN_1104_ZN_1565
2crr_A_ZN_401_ZN_2232
2crw_A_ZN_401_ZN_2221
2csy_A_ZN_201_ZN_1202
2csz_A_ZN_201_ZN_1120
2csz_A_ZN_401_ZN_1121
2ct2_A_ZN_201_ZN_1323
2ct7_A_ZN_401_ZN_1340
2d9l_A_ZN_401_ZN_2090
2dip_A_ZN_201_ZN_1514
2dx8_A_ZN_401_ZN_903
2dx8_A_ZN_402_ZN_904
2dx8_B_ZN_403_ZN_905
2dx8_B_ZN_404_ZN_906
2e5r_A_ZN_201_ZN_936
2e6r_A_ZN_201_ZN_1339
2e6r_A_ZN_401_ZN_1340
2e6s_A_ZN_201_ZN_1106
2e6s_A_ZN_401_ZN_1107
2e6s_A_ZN_601_ZN_1108
2ecl_A_ZN_201_ZN_1218
2ecm_A_ZN_201_ZN_786
2ecn_A_ZN_201_ZN_1007
2ecn_A_ZN_401_ZN_1008
2ect_A_ZN_201_ZN_1097
2ecw_A_ZN_201_ZN_1251

2egp_A_ZN_200_ZN_1116
2ewl_A_ZN_57_ZN_863
2f6j_A_ZN_1001_ZN_4191
2f6j_A_ZN_1002_ZN_4192
2f6j_B_ZN_1003_ZN_4193
2f6j_B_ZN_1004_ZN_4194
2f6j_C_ZN_1005_ZN_4195
2f6j_C_ZN_1006_ZN_4196
2f6n_A_ZN_1001_ZN_2721
2f6n_A_ZN_1002_ZN_2722
2f6n_B_ZN_1003_ZN_2723
2f6n_B_ZN_1004_ZN_2724
2f8b_A_ZN_57_ZN_1725
2f8b_B_ZN_57_ZN_1726
2fc7_A_ZN_201_ZN_1166
2fsa_A_ZN_501_ZN_4190
2fsa_A_ZN_502_ZN_4191
2fsa_B_ZN_503_ZN_4192
2fsa_B_ZN_504_ZN_4193
2fsa_C_ZN_505_ZN_4194
2fsa_C_ZN_506_ZN_4195
2fuu_A_ZN_201_ZN_1179
2fuu_A_ZN_202_ZN_1180
2g43_A_ZN_1_ZN_1760
2g43_B_ZN_131_ZN_1761
2g45_A_ZN_401_ZN_3039
2g45_D_ZN_402_ZN_3041
2g6q_A_ZN_300_ZN_491
2g6q_A_ZN_400_ZN_492
2h0d_A_ZN_201_ZN_1592
2h0d_B_ZN_203_ZN_1594
2hye_D_ZN_4001_ZN_16945
2i50_A_ZN_336_ZN_1926
2i50_A_ZN_338_ZN_1928
2ida_A_ZN_103_ZN_1551
2iqj_A_ZN_301_ZN_1944
2iqj_B_ZN_301_ZN_1950
2jm1_A_ZN_1_ZN_2192
2jm1_A_ZN_2_ZN_2193
2jm1_A_ZN_3_ZN_2194
2jmj_A_ZN_201_ZN_1095
2jmj_A_ZN_401_ZN_1096
2jmo_A_ZN_401_ZN_1135
2jrj_A_ZN_62_ZN_815
2jrj_A_ZN_63_ZN_816
2k1j_A_ZN_250_ZN_988
2k1j_A_ZN_251_ZN_989
2ke1_A_ZN_355_ZN_1129
2ke1_A_ZN_356_ZN_1130
2kft_A_ZN_401_ZN_1142
2kft_A_ZN_501_ZN_1143
2ko5_A_ZN_100_ZN_1500
2kwj_A_ZN_501_ZN_2024
2kwj_A_ZN_601_ZN_2025
2kwj_A_ZN_701_ZN_2026
2kwj_A_ZN_801_ZN_2027
2kwk_A_ZN_501_ZN_2020
2kwk_A_ZN_601_ZN_2021

2kwk_A_ZN_701_ZN_2022
2kwk_A_ZN_801_ZN_2023
2kwn_A_ZN_501_ZN_1948
2kwn_A_ZN_601_ZN_1949
2kwn_A_ZN_701_ZN_1950
2kwn_A_ZN_801_ZN_1951
2kwo_A_ZN_501_ZN_1996
2kwo_A_ZN_601_ZN_1997
2kwo_A_ZN_701_ZN_1998
2kwo_A_ZN_801_ZN_1999
2kyu_A_ZN_81_ZN_1010
2kyu_A_ZN_82_ZN_1011
2olm_A_ZN_201_ZN_1074
2owa_A_ZN_201_ZN_2034
2owa_B_ZN_201_ZN_2035
2p57_A_ZN_201_ZN_983
2pnx_A_ZN_300_ZN_974
2pnx_A_ZN_400_ZN_975
2pnx_C_ZN_300_ZN_976
2pnx_C_ZN_400_ZN_977
2puy_A_ZN_355_ZN_1078
2puy_A_ZN_356_ZN_1079
2puy_B_ZN_357_ZN_1080
2puy_B_ZN_358_ZN_1081
2pv0_A_ZN_502_ZN_7856
2pv0_A_ZN_503_ZN_7857
2pv0_B_ZN_505_ZN_7859
2pv0_B_ZN_506_ZN_7860
2pv0_C_ZN_509_ZN_7863
2pvc_A_ZN_602_ZN_8524
2pvc_A_ZN_603_ZN_8525
2pvc_B_ZN_605_ZN_8527
2pvc_B_ZN_606_ZN_8528
2pvc_C_ZN_608_ZN_8530
2pvc_C_ZN_609_ZN_8531
2qgp_A_ZN_113_ZN_2249
2qgp_B_ZN_113_ZN_2250
2qgp_C_ZN_113_ZN_2251
2qic_A_ZN_300_ZN_480
2qic_A_ZN_400_ZN_481
2ri7_A_ZN_501_ZN_1462
2ri7_A_ZN_502_ZN_1463
2ro1_A_ZN_301_ZN_2908
2uzg_A_ZN_131_ZN_1476
2uzg_A_ZN_132_ZN_1477
2v1c_C_ZN_266_ZN_4784
2v83_A_ZN_1487_ZN_1944
2v83_B_ZN_1488_ZN_1945
2v83_C_ZN_1484_ZN_1947
2v86_A_ZN_1488_ZN_1466
2v86_A_ZN_1489_ZN_1467
2v86_B_ZN_1488_ZN_1468
2v86_B_ZN_1489_ZN_1469
2v88_A_ZN_1488_ZN_1408
2v88_B_ZN_1488_ZN_1410
2v89_A_ZN_2488_ZN_2724
2v89_A_ZN_2489_ZN_2725
2vhf_A_ZN_1957_ZN_5250

2vhf_A_ZN_1958_ZN_5251
2vhf_B_ZN_1956_ZN_5252
2vhf_B_ZN_1957_ZN_5253
2vnf_A_ZN_1246_ZN_967
2vnf_A_ZN_1247_ZN_968
2vnf_C_ZN_1247_ZN_977
2vnf_C_ZN_1248_ZN_978
2vp7_A_ZN_1399_ZN_782
2vpb_A_ZN_1400_ZN_711
2vpd_A_ZN_1399_ZN_1457
2vpd_C_ZN_1398_ZN_1459
2vpd_C_ZN_1399_ZN_1460
2vpe_A_ZN_1400_ZN_1485
2vpe_C_ZN_1398_ZN_1487
2vpg_A_ZN_1400_ZN_1503
2vpg_C_ZN_1399_ZN_1505
2vpg_C_ZN_1400_ZN_1506
2xb1_A_ZN_1390_ZN_1513
2xb1_C_ZN_1390_ZN_1521
2xoc_A_ZN_994_ZN_3437
2xoc_B_ZN_994_ZN_3479
2xoy_A_ZN_994_ZN_3390
2xoy_B_ZN_994_ZN_3395
2xoz_A_ZN_994_ZN_3425
2xoz_B_ZN_994_ZN_3430
2xp0_A_ZN_994_ZN_3412
2xp0_B_ZN_994_ZN_3417
2yql_A_ZN_201_ZN_830
2yql_A_ZN_401_ZN_831
2yqm_A_ZN_201_ZN_1338
2yqm_A_ZN_401_ZN_1339
2ysm_A_ZN_301_ZN_1582
2ysm_A_ZN_501_ZN_1583
2ysm_A_ZN_701_ZN_1584
2ysm_A_ZN_901_ZN_1585
2yt5_A_ZN_201_ZN_964
2yt5_A_ZN_401_ZN_965
2yur_A_ZN_201_ZN_1007
2yw8_A_ZN_300_ZN_489
2yw8_A_ZN_301_ZN_490
2yyr_A_ZN_401_ZN_955
2yyr_A_ZN_402_ZN_956
2yyr_B_ZN_403_ZN_957
2yyr_B_ZN_404_ZN_958
2zet_C_ZN_301_ZN_5246
2zet_C_ZN_302_ZN_5247
2zet_D_ZN_301_ZN_5286
2zet_D_ZN_302_ZN_5287
3a1a_A_ZN_2_ZN_1044
3a1a_A_ZN_3_ZN_1045
3a1b_A_ZN_2_ZN_1184
3a1b_A_ZN_3_ZN_1185
3c5k_A_ZN_202_ZN_843
3c5k_A_ZN_203_ZN_844
3c6w_A_ZN_1_ZN_980
3c6w_A_ZN_2_ZN_981
3c6w_C_ZN_3_ZN_982
3c6w_C_ZN_4_ZN_983

3dpl_R_ZN_201_ZN_3859
3dqv_R_ZN_4005_ZN_8841
3dqv_Y_ZN_4002_ZN_8844
3dwd_A_ZN_501_ZN_1760
3dwd_B_ZN_501_ZN_1762
3feh_A_ZN_375_ZN_2859
3fl2_A_ZN_1001_ZN_909
3fm8_C_ZN_401_ZN_7171
3fm8_D_ZN_401_ZN_7183
3gv4_A_ZN_202_ZN_803
3gv4_A_ZN_203_ZN_804
3hcs_A_ZN_301_ZN_2521
3hcs_B_ZN_306_ZN_2526
3hct_A_ZN_301_ZN_2019
3hcu_A_ZN_301_ZN_4064
3hcu_C_ZN_304_ZN_4067
3ihp_A_ZN_836_ZN_11764
3ihp_B_ZN_836_ZN_11765
3jue_A_ZN_999_ZN_3815
3jue_B_ZN_999_ZN_3826
3k1l_A_ZN_383_ZN_6014
3k1l_B_ZN_383_ZN_5968
3knv_A_ZN_201_ZN_942
3knv_A_ZN_202_ZN_943
3kqi_A_ZN_71_ZN_625
3kqi_A_ZN_72_ZN_626
3kv4_A_ZN_448_ZN_3603
3kv4_A_ZN_449_ZN_3604
3kv5_A_ZN_489_ZN_7112
3kv5_A_ZN_490_ZN_7113
3kv5_D_ZN_489_ZN_7104
3kv5_D_ZN_490_ZN_7105
3kv6_A_ZN_489_ZN_7197
3kv6_A_ZN_490_ZN_7198
3kv6_D_ZN_489_ZN_7212
3kv6_D_ZN_490_ZN_7213
3l11_A_ZN_602_ZN_844
3ldy_A_ZN_143_ZN_1517
3ldy_A_ZN_144_ZN_1518
3lju_X_ZN_401_ZN_3070
3lqh_A_ZN_1001_ZN_1422
3lqh_A_ZN_1002_ZN_1423
3lqi_A_ZN_1_ZN_4533
3lqi_A_ZN_2_ZN_4534
3lqi_B_ZN_3_ZN_4535
3lqi_B_ZN_4_ZN_4536
3lqi_C_ZN_5_ZN_4537
3lqi_C_ZN_6_ZN_4538
3lqj_A_ZN_3_ZN_2983
3lqj_A_ZN_4_ZN_2984
3lqj_B_ZN_1_ZN_2985
3lqj_B_ZN_2_ZN_2986
3lvq_E_ZN_680_ZN_3264
3lvr_E_ZN_735_ZN_3259
3m7k_A_ZN_143_ZN_1522
3m7k_A_ZN_144_ZN_1523
3m99_A_ZN_472_ZN_5514
3m99_A_ZN_473_ZN_5515

3mdb_C_ZN_401_ZN_7071
3mdb_D_ZN_401_ZN_7072
3mhh_A_ZN_472_ZN_5732
3mhh_A_ZN_473_ZN_5733
3mhs_A_ZN_472_ZN_6611
3mhs_A_ZN_473_ZN_6612
3mhs_A_ZN_477_ZN_6616
3n9l_A_ZN_2_ZN_4053
3n9l_A_ZN_3_ZN_4054
3n9m_A_ZN_2_ZN_8110
3n9m_A_ZN_3_ZN_8111
3n9m_C_ZN_2_ZN_8113
3n9m_C_ZN_3_ZN_8114
3n9n_A_ZN_2_ZN_4136
3n9n_A_ZN_3_ZN_4137
3n9o_A_ZN_2_ZN_4097
3n9o_A_ZN_3_ZN_4098
3n9p_A_ZN_2_ZN_4133
3n9p_A_ZN_3_ZN_4134
3n9q_A_ZN_2_ZN_4100
3n9q_A_ZN_3_ZN_4101
3o33_A_ZN_1_ZN_5744
3o33_A_ZN_2_ZN_5745
3o33_B_ZN_1_ZN_5746
3o33_B_ZN_2_ZN_5747
3o33_C_ZN_1_ZN_5748
3o33_C_ZN_2_ZN_5749
3o33_D_ZN_1_ZN_5750
3o33_D_ZN_2_ZN_5751
3o34_A_ZN_1_ZN_1532
3o34_A_ZN_2_ZN_1533
3o35_A_ZN_1_ZN_2950
3o35_A_ZN_2_ZN_2951
3o35_B_ZN_1_ZN_2952
3o35_B_ZN_2_ZN_2953
3o36_A_ZN_1_ZN_2969
3o36_A_ZN_2_ZN_2970
3o36_B_ZN_1_ZN_2971
3o36_B_ZN_2_ZN_2972
3o37_A_ZN_1_ZN_6073
3o37_A_ZN_2_ZN_6074
3o37_B_ZN_1_ZN_6075
3o37_B_ZN_2_ZN_6076
3o37_C_ZN_1_ZN_6077
3o37_C_ZN_2_ZN_6078
3o37_D_ZN_1_ZN_6079
3o37_D_ZN_2_ZN_6080
3o47_A_ZN_501_ZN_4449
3o47_B_ZN_501_ZN_4478
3o70_A_ZN_500_ZN_457
3o70_A_ZN_501_ZN_458
3o7a_A_ZN_500_ZN_476
3o7a_A_ZN_501_ZN_477

------- SF_63 --------
1a6y_A_ZN_550_ZN_2101
1a6y_B_ZN_450_ZN_2103
1a7i_A_ZN_82_ZN_905

1a7i_A_ZN_83_ZN_906
1b8t_A_ZN_193_ZN_2771
1b8t_A_ZN_194_ZN_2772
1b8t_A_ZN_195_ZN_2773
1b8t_A_ZN_196_ZN_2774
1by4_A_ZN_1330_ZN_3833
1by4_B_ZN_1335_ZN_3835
1by4_C_ZN_2330_ZN_3837
1by4_D_ZN_2335_ZN_3839
1cit_A_ZN_398_ZN_1344
1ctl_A_ZN_86_ZN_1214
1ctl_A_ZN_87_ZN_1215
1cxx_A_ZN_1_ZN_868
1cxx_A_ZN_2_ZN_869
1dsz_A_ZN_1121_ZN_1856
1dsz_B_ZN_1221_ZN_1858
1fjg_N_ZN_190_ZN_51953
1g47_A_ZN_998_ZN_1057
1g47_A_ZN_999_ZN_1058
1ga5_A_ZN_450_ZN_4000
1ga5_B_ZN_550_ZN_4002
1ga5_E_ZN_450_ZN_4004
1ga5_F_ZN_550_ZN_4006
1gdc_A_ZN_73_ZN_1108
1glu_A_ZN_515_ZN_2031
1glu_B_ZN_515_ZN_2033
1gnf_A_ZN_244_ZN_710
1hcp_A_ZN_98_ZN_1170,1hcp_A_ZN_98_ZN_1171
1hcq_A_ZN_598_ZN_3707
1hcq_B_ZN_598_ZN_3709
1hcq_E_ZN_598_ZN_3711
1hcq_F_ZN_598_ZN_3713
1hlz_A_ZN_450_ZN_1979
1hlz_B_ZN_550_ZN_1981
1hnw_N_ZN_190_ZN_51890
1hnx_N_ZN_190_ZN_51891
1hnz_N_ZN_190_ZN_51891
1hr0_N_ZN_190_ZN_52387
1hra_A_ZN_81_ZN_654
1i94_N_ZN_76_ZN_44490
1ibi_A_ZN_195_ZN_882
1ibi_A_ZN_196_ZN_883
1ibk_N_ZN_307_ZN_51894
1ibl_N_ZN_307_ZN_52294
1ibm_N_ZN_507_ZN_52184
1iml_A_ZN_77_ZN_1150
1iml_A_ZN_78_ZN_1151
1j2o_A_ZN_115_ZN_1708
1j2o_A_ZN_116_ZN_1709
1j5e_N_ZN_307_ZN_51766
1kb2_A_ZN_150_ZN_2101
1kb2_B_ZN_350_ZN_2103
1kb4_A_ZN_150_ZN_2205
1kb4_B_ZN_350_ZN_2207
1kb6_A_ZN_150_ZN_2238
1kb6_B_ZN_350_ZN_2240
1lat_A_ZN_1514_ZN_1912
1lat_B_ZN_1514_ZN_1914

1lo1_A_ZN_195_ZN_1846
1lv3_A_ZN_66_ZN_991
1m3v_A_ZN_123_ZN_1749
1m3v_A_ZN_124_ZN_1750
1n32_N_ZN_307_ZN_52298
1n33_N_ZN_307_ZN_52163
1n36_N_ZN_307_ZN_51701
1nyp_A_ZN_67_ZN_1032
1nyp_A_ZN_68_ZN_1033
1ovx_A_ZN_61_ZN_1185
1ovx_B_ZN_61_ZN_1186
1pzw_A_ZN_100_ZN_644
1qli_A_ZN_195_ZN_882
1qli_A_ZN_196_ZN_883
1r0n_A_ZN_177_ZN_2035
1r0n_B_ZN_150_ZN_2037
1r0o_A_ZN_351_ZN_1962
1r0o_B_ZN_350_ZN_1964
1r4i_A_ZN_650_ZN_1814
1r4i_B_ZN_652_ZN_1816
1r4o_A_ZN_526_ZN_2013
1r4o_B_ZN_526_ZN_2015
1r4r_A_ZN_526_ZN_2315
1r4r_B_ZN_526_ZN_2317
1rgd_A_ZN_72_ZN_550
1rut_X_ZN_601_ZN_1252
1rut_X_ZN_602_ZN_1253
1rut_X_ZN_603_ZN_1254
1rut_X_ZN_604_ZN_1255
1rxr_A_ZN_213_ZN_1350
1tjl_A_ZN_200_ZN_11801
1tjl_B_ZN_200_ZN_11802
1tjl_C_ZN_200_ZN_11803
1tjl_D_ZN_200_ZN_11804
1tjl_E_ZN_200_ZN_11805
1tjl_F_ZN_200_ZN_11806
1tjl_G_ZN_200_ZN_11807
1tjl_H_ZN_200_ZN_11808
1tjl_I_ZN_200_ZN_11809
1tjl_J_ZN_200_ZN_11810
1u5s_B_ZN_138_ZN_2151
1u5s_B_ZN_139_ZN_2152
1uw0_A_ZN_1118_ZN_1865
1v6g_A_ZN_201_ZN_1163
1v6g_A_ZN_401_ZN_1164
1v9x_A_ZN_200_ZN_1711
1w4r_A_ZN_400_ZN_10270
1w4r_B_ZN_400_ZN_10329
1w4r_C_ZN_400_ZN_10388
1w4r_D_ZN_400_ZN_10447
1w4r_E_ZN_400_ZN_10506
1w4r_F_ZN_400_ZN_10573
1w4r_G_ZN_400_ZN_10632
1w4r_H_ZN_400_ZN_10691
1wig_A_ZN_201_ZN_1022
1wig_A_ZN_401_ZN_1023
1wyh_A_ZN_201_ZN_987
1wyh_A_ZN_401_ZN_988

1x3h_A_ZN_201_ZN_1150
1x3h_A_ZN_401_ZN_1151
1x4j_A_ZN_201_ZN_1122
1x4k_A_ZN_201_ZN_1066
1x4k_A_ZN_401_ZN_1067
1x4l_A_ZN_201_ZN_1004
1x4l_A_ZN_401_ZN_1005
1x61_A_ZN_201_ZN_969
1x61_A_ZN_401_ZN_970
1x62_A_ZN_201_ZN_1169
1x62_A_ZN_401_ZN_1170
1x63_A_ZN_201_ZN_1171
1x63_A_ZN_401_ZN_1172
1x64_A_ZN_201_ZN_1279
1x64_A_ZN_401_ZN_1280
1x68_A_ZN_201_ZN_1044
1x6a_A_ZN_201_ZN_1148
1xbt_A_ZN_1193_ZN_9999
1xbt_B_ZN_2193_ZN_10030
1xbt_C_ZN_3193_ZN_10061
1xbt_D_ZN_4193_ZN_10092
1xbt_E_ZN_5193_ZN_10123
1xbt_F_ZN_6193_ZN_10154
1xbt_G_ZN_7193_ZN_10185
1xbt_H_ZN_8193_ZN_10216
1xmo_N_ZN_307_ZN_52085
1xmq_N_ZN_307_ZN_52103
1xnq_N_ZN_307_ZN_52098
1xnr_N_ZN_307_ZN_52096
1xx6_A_ZN_302_ZN_2764
1xx6_B_ZN_402_ZN_2792
1y0j_A_ZN_244_ZN_1140
1ynw_A_ZN_150_ZN_2071
1ynw_B_ZN_350_ZN_2073
1zfo_A_ZN_31_ZN_489
2a66_A_ZN_401_ZN_1240
2b8t_A_ZN_1218_ZN_6385
2b8t_B_ZN_2218_ZN_6403
2b8t_C_ZN_3218_ZN_6421
2b8t_D_ZN_4218_ZN_6439
2c7a_A_ZN_1641_ZN_1898
2c7a_B_ZN_1639_ZN_1900
2c7m_A_ZN_500_ZN_1073
2c7n_A_ZN_499_ZN_6191
2c7n_C_ZN_499_ZN_6192
2c7n_E_ZN_499_ZN_6193
2c7n_G_ZN_499_ZN_6194
2c7n_I_ZN_499_ZN_6195
2c7n_K_ZN_499_ZN_6196
2co8_A_ZN_201_ZN_1159
2co8_A_ZN_401_ZN_1160
2cor_A_ZN_201_ZN_1150
2cor_A_ZN_401_ZN_1151
2cs2_A_ZN_200_ZN_2037
2cu8_A_ZN_201_ZN_1093
2cu8_A_ZN_401_ZN_1094
2cup_A_ZN_201_ZN_1514
2cup_A_ZN_401_ZN_1515

2cup_A_ZN_601_ZN_1516
2cuq_A_ZN_201_ZN_1137
2cuq_A_ZN_401_ZN_1138
2cur_A_ZN_201_ZN_982
2cur_A_ZN_401_ZN_983
2d8s_A_ZN_201_ZN_1147
2d8x_A_ZN_201_ZN_1018
2d8x_A_ZN_401_ZN_1019
2d8y_A_ZN_201_ZN_1368
2d8y_A_ZN_401_ZN_1369
2d8z_A_ZN_201_ZN_1019
2d8z_A_ZN_401_ZN_1020
2dar_A_ZN_201_ZN_1324
2dar_A_ZN_401_ZN_1325
2das_A_ZN_101_ZN_876
2dfy_C_ZN_601_ZN_2460
2dfy_C_ZN_602_ZN_2461
2dfy_C_ZN_603_ZN_2462
2dfy_C_ZN_604_ZN_2463
2dfy_X_ZN_601_ZN_2450
2dfy_X_ZN_602_ZN_2451
2dfy_X_ZN_603_ZN_2452
2dfy_X_ZN_604_ZN_2453
2dj7_A_ZN_201_ZN_1155
2dj7_A_ZN_401_ZN_1156
2dlo_A_ZN_201_ZN_1162
2dlo_A_ZN_401_ZN_1163
2dmj_A_ZN_200_ZN_1593
2ds5_A_ZN_100_ZN_677
2ds5_B_ZN_100_ZN_679
2ds6_A_ZN_100_ZN_654
2ds6_B_ZN_100_ZN_655
2ds7_A_ZN_100_ZN_293
2ds8_A_ZN_100_ZN_750
2ds8_B_ZN_100_ZN_751
2e5l_N_ZN_62_ZN_51918
2ea6_A_ZN_201_ZN_996
2ebl_A_ZN_191_ZN_1351
2ecv_A_ZN_201_ZN_1251
2egq_A_ZN_200_ZN_1148
2egq_A_ZN_300_ZN_1149
2ehe_A_ZN_200_ZN_1161
2ehe_A_ZN_300_ZN_1162
2env_A_ZN_200_ZN_1336
2ep4_A_ZN_200_ZN_1138
2ep4_A_ZN_300_ZN_1139
2eqe_A_ZN_201_ZN_648
2eqf_A_ZN_201_ZN_569
2eqg_A_ZN_201_ZN_688
2f4v_N_ZN_307_ZN_51570
2ff0_A_ZN_1001_ZN_2615
2fid_B_ZN_901_ZN_1089
2fif_B_ZN_901_ZN_3213
2fif_D_ZN_902_ZN_3214
2fif_F_ZN_903_ZN_3215
2fk4_A_ZN_101_ZN_1108
2gda_A_ZN_73_ZN_1108
2han_A_ZN_351_ZN_2165

2han_B_ZN_353_ZN_2167
2iyb_E_ZN_1422_ZN_5396
2iyb_E_ZN_1423_ZN_5397
2iyb_F_ZN_1421_ZN_5399
2iyb_F_ZN_1422_ZN_5400
2iyb_G_ZN_1421_ZN_5401
2iyb_G_ZN_1422_ZN_5402
2iyb_H_ZN_1421_ZN_5403
2iyb_H_ZN_1422_ZN_5404
2j00_Z_ZN_743_ZN_56165
2j02_Z_ZN_640_ZN_56062
2j87_A_ZN_400_ZN_5240
2j87_B_ZN_400_ZN_5271
2j87_C_ZN_400_ZN_5302
2j87_D_ZN_400_ZN_5333
2j9r_A_ZN_1194_ZN_1387
2ja1_A_ZN_1192_ZN_1501
2jtn_A_ZN_183_ZN_2748
2jtn_A_ZN_184_ZN_2749
2jtn_A_ZN_185_ZN_2750
2jtn_A_ZN_186_ZN_2751
2kae_A_ZN_175_ZN_2224
2kbx_B_ZN_298_ZN_3736
2kbx_B_ZN_299_ZN_3737
2kiz_A_ZN_70_ZN_1079
2kq9_A_ZN_113_ZN_1746
2l3k_A_ZN_124_ZN_1708
2l3k_A_ZN_125_ZN_1709
2nll_A_ZN_250_ZN_2116
2nll_B_ZN_450_ZN_2118
2o10_A_ZN_86_ZN_911
2o10_A_ZN_87_ZN_912
2o13_A_ZN_190_ZN_882
2o13_A_ZN_191_ZN_883
2orv_A_ZN_235_ZN_2482
2orv_B_ZN_235_ZN_2516
2orw_A_ZN_401_ZN_2697
2orw_B_ZN_402_ZN_2699
2pv0_A_ZN_501_ZN_7858
2pv0_B_ZN_504_ZN_7861
2pv0_C_ZN_507_ZN_7864
2pv0_C_ZN_508_ZN_7862
2pvc_A_ZN_601_ZN_8526
2pvc_B_ZN_604_ZN_8529
2pvc_C_ZN_607_ZN_8532
2qpo_A_ZN_201_ZN_4736
2qpo_B_ZN_202_ZN_4742
2qpo_C_ZN_204_ZN_4748
2qpo_D_ZN_203_ZN_4754
2qq0_A_ZN_400_ZN_2662
2qq0_B_ZN_402_ZN_2712
2qqe_A_ZN_400_ZN_2507
2qqe_B_ZN_402_ZN_2525
2rgt_A_ZN_205_ZN_2372
2rgt_A_ZN_206_ZN_2373
2rgt_A_ZN_207_ZN_2374
2rgt_A_ZN_208_ZN_2375
2rgt_B_ZN_200_ZN_2376

2rgt_B_ZN_201_ZN_2377
2rgt_B_ZN_203_ZN_2378
2rgt_B_ZN_204_ZN_2379
2uu9_Z_ZN_1122_ZN_52298
2uua_Z_ZN_139_ZN_52274
2uub_Z_ZN_1139_ZN_52270
2uuc_Z_ZN_1121_ZN_52274
2uxb_Z_ZN_144_ZN_52189
2uxc_Z_ZN_221_ZN_52302
2uxd_Z_ZN_81_ZN_51493
2uz3_A_ZN_1218_ZN_6390
2uz3_B_ZN_2218_ZN_6420
2uz3_C_ZN_3218_ZN_6451
2uz3_D_ZN_4218_ZN_6481
2v46_Z_ZN_743_ZN_55965
2v48_Z_ZN_743_ZN_55965
2vqe_Z_ZN_141_ZN_52097
2vqf_Z_ZN_141_ZN_52188
2vus_I_ZN_1713_ZN_22903
2vus_J_ZN_1713_ZN_22904
2vus_K_ZN_1712_ZN_22905
2vus_L_ZN_1713_ZN_22906
2vus_M_ZN_1713_ZN_22907
2vus_N_ZN_1713_ZN_22908
2vus_O_ZN_1712_ZN_22909
2vus_P_ZN_1713_ZN_22910
2vut_I_ZN_1713_ZN_23280
2vut_J_ZN_1713_ZN_23281
2vut_K_ZN_1712_ZN_23282
2vut_L_ZN_1713_ZN_23283
2vut_M_ZN_1713_ZN_23290
2vut_N_ZN_1713_ZN_23291
2vut_O_ZN_1712_ZN_23292
2vut_P_ZN_1713_ZN_23293
2vuu_I_ZN_1713_ZN_23283
2vuu_J_ZN_1713_ZN_23284
2vuu_K_ZN_1713_ZN_23285
2vuu_L_ZN_1713_ZN_23286
2vuu_M_ZN_1712_ZN_23287
2vuu_N_ZN_1713_ZN_23288
2vuu_O_ZN_1712_ZN_23289
2vuu_P_ZN_1713_ZN_23290
2wdg_Z_ZN_743_ZN_57354
2wdh_Z_ZN_743_ZN_57354
2wdk_Z_ZN_743_ZN_57292
2wdm_Z_ZN_743_ZN_57292
2wh1_Z_ZN_548_ZN_58235
2wh3_Z_ZN_548_ZN_58235
2wri_Z_ZN_4_ZN_60239
2wrk_Z_ZN_4_ZN_60239
2wrn_1_ZN_4_ZN_59575
2wrq_1_ZN_4_ZN_59575
2wvj_A_ZN_1193_ZN_10028
2wvj_B_ZN_1194_ZN_10058
2wvj_C_ZN_1193_ZN_10088
2wvj_D_ZN_1194_ZN_10119
2wvj_E_ZN_1193_ZN_10149
2wvj_F_ZN_1193_ZN_10179

2wvj_G_ZN_1194_ZN_10210
2wvj_H_ZN_1194_ZN_10241
2xfz_Z_ZN_5_ZN_55965
2xjy_A_ZN_201_ZN_1335
2xjy_A_ZN_202_ZN_1336
2xjy_A_ZN_203_ZN_1337
2xjy_A_ZN_204_ZN_1338
2xjz_A_ZN_201_ZN_6098
2xjz_A_ZN_202_ZN_6099
2xjz_A_ZN_203_ZN_6100
2xjz_A_ZN_204_ZN_6101
2xjz_B_ZN_201_ZN_6102
2xjz_B_ZN_202_ZN_6103
2xjz_B_ZN_203_ZN_6104
2xjz_B_ZN_204_ZN_6105
2xjz_C_ZN_201_ZN_6107
2xjz_C_ZN_202_ZN_6108
2xjz_C_ZN_203_ZN_6109
2xjz_D_ZN_201_ZN_6111
2xjz_D_ZN_202_ZN_6112
2xjz_D_ZN_203_ZN_6113
2xjz_D_ZN_204_ZN_6114
2xjz_E_ZN_201_ZN_6116
2xjz_E_ZN_202_ZN_6117
2xjz_E_ZN_203_ZN_6118
2xjz_E_ZN_204_ZN_6119
2xqd_N_ZN_1062_ZN_59870
2ysj_A_ZN_201_ZN_916
2zm6_N_ZN_62_ZN_51329
3a1a_A_ZN_1_ZN_1043
3a1b_A_ZN_1_ZN_1183
3cbb_A_ZN_1001_ZN_2090
3cbb_B_ZN_2001_ZN_2092
3d5a_N_ZN_62_ZN_57811
3d5c_N_ZN_62_ZN_57813
3dfv_C_ZN_30_ZN_1804
3dfv_D_ZN_30_ZN_1803
3dfx_A_ZN_30_ZN_1713
3dfx_B_ZN_30_ZN_1714
3dzu_A_ZN_7221_ZN_6160
3dzu_D_ZN_7121_ZN_6158
3dzy_A_ZN_7221_ZN_6276
3dzy_D_ZN_7121_ZN_6274
3e00_A_ZN_7221_ZN_6208
3e00_D_ZN_7121_ZN_6206
3e2i_A_ZN_200_ZN_1351
3f1e_N_ZN_5002_ZN_57773
3f1g_N_ZN_5002_ZN_57773
3f6q_B_ZN_301_ZN_2001
3f6q_B_ZN_302_ZN_2002
3fyl_A_ZN_526_ZN_1819
3fyl_B_ZN_526_ZN_1825
3g27_A_ZN_97_ZN_610
3g6p_A_ZN_1514_ZN_1922
3g6p_B_ZN_1512_ZN_1930
3g6q_A_ZN_526_ZN_1818
3g6q_B_ZN_526_ZN_1816
3g6r_A_ZN_1512_ZN_1905

3g6r_B_ZN_1514_ZN_1903
3g6t_A_ZN_527_ZN_1813
3g6t_B_ZN_527_ZN_1815
3g6u_A_ZN_526_ZN_1853
3g6u_B_ZN_526_ZN_1855
3g8u_A_ZN_526_ZN_1831
3g8u_B_ZN_526_ZN_1833
3g8x_A_ZN_526_ZN_1815
3g8x_B_ZN_526_ZN_1825
3g97_A_ZN_526_ZN_1766
3g97_B_ZN_526_ZN_1764
3g99_A_ZN_526_ZN_1823
3g99_B_ZN_526_ZN_1829
3g9i_A_ZN_526_ZN_1867
3g9i_B_ZN_526_ZN_1877
3g9j_A_ZN_1514_ZN_1913
3g9j_B_ZN_1512_ZN_1919
3g9m_A_ZN_526_ZN_1899
3g9m_B_ZN_526_ZN_1909
3g9o_A_ZN_526_ZN_1816
3g9o_B_ZN_526_ZN_1814
3g9p_A_ZN_526_ZN_1816
3g9p_B_ZN_526_ZN_1814
3gat_A_ZN_67_ZN_2091
3i8g_Q_ZN_62_ZN_58449
3i8h_Q_ZN_62_ZN_58256
3i9b_Q_ZN_62_ZN_56866
3i9d_Q_ZN_62_ZN_58415
3ixe_B_ZN_301_ZN_2002
3ixe_B_ZN_302_ZN_2003
3kiq_n_ZN_610_ZN_55999
3kis_n_ZN_610_ZN_55993
3kiu_n_ZN_62_ZN_56009
3kix_n_ZN_610_ZN_56009
3m9e_A_ZN_208_ZN_5078
3m9e_B_ZN_208_ZN_5080
3m9e_E_ZN_208_ZN_5082
3m9e_F_ZN_208_ZN_5084
3mr8_N_ZN_1_ZN_56070
3ms0_N_ZN_62_ZN_56114
3oge_N_ZN_62_ZN_51498
3ogy_N_ZN_62_ZN_51495
3ohc_N_ZN_62_ZN_51496
3ohd_N_ZN_62_ZN_51492
3ohy_N_ZN_62_ZN_51493
3oi0_N_ZN_62_ZN_51490
3oi2_N_ZN_62_ZN_51494
3oi4_N_ZN_62_ZN_51492
3oj3_I_ZN_901_ZN_6619
3oj3_J_ZN_902_ZN_6620
3oj3_K_ZN_903_ZN_6621
3oj3_L_ZN_904_ZN_6622
3oj3_M_ZN_905_ZN_6623
3oj3_N_ZN_906_ZN_6624
3oj3_O_ZN_907_ZN_6625
3oj3_P_ZN_908_ZN_6626
3oj4_C_ZN_900_ZN_3993
3oj4_F_ZN_900_ZN_3994

3oto_N_ZN_141_ZN_51660
5gat_A_ZN_67_ZN_1859
7gat_A_ZN_67_ZN_1857

------- SF_64 --------
1faq_A_ZN_1_ZN_846
1far_A_ZN_1_ZN_846
1kbe_A_ZN_2_ZN_779
1kbf_A_ZN_2_ZN_779
1ptq_A_ZN_2_ZN_405
1ptr_A_ZN_2_ZN_390
1r79_A_ZN_401_ZN_1210
1rfh_A_ZN_1_ZN_923,1rfh_A_ZN_1_ZN_924
1tbn_A_ZN_2_ZN_1027
1tbo_A_ZN_2_ZN_1027,1tbo_A_ZN_2_ZN_1031
1v5n_A_ZN_201_ZN_1357
1xa6_A_ZN_468_ZN_3257
1y8f_A_ZN_702_ZN_774
1z6u_A_ZN_2_ZN_2051
1z6u_B_ZN_4_ZN_2053
2ckl_B_ZN_1116_ZN_1569
2ct0_A_ZN_201_ZN_1078
2ctu_A_ZN_201_ZN_1099
2d8t_A_ZN_201_ZN_1009
2db6_A_ZN_401_ZN_1146
2djb_A_ZN_201_ZN_1068
2e73_A_ZN_201_ZN_1183
2eci_A_ZN_201_ZN_1316
2ecj_A_ZN_201_ZN_860
2ecy_A_ZN_201_ZN_882
2eli_A_ZN_401_ZN_1260
2enn_A_ZN_400_ZN_1168
2enz_A_ZN_400_ZN_974
2fnf_X_ZN_1_ZN_922
2gvi_A_ZN_301_ZN_1602
2jmd_A_ZN_65_ZN_976
2k2c_A_ZN_142_ZN_2128
2k2c_A_ZN_143_ZN_2129
2l0b_A_ZN_143_ZN_1370
2row_A_ZN_602_ZN_1377
2vrw_B_ZN_1566_ZN_4414
2xeu_A_ZN_1065_ZN_509
2ysl_A_ZN_201_ZN_1092
2yuu_A_ZN_401_ZN_1236
3bji_A_ZN_1_ZN_8364
3bji_B_ZN_3_ZN_8367
3cxl_A_ZN_501_ZN_3170
3d00_A_ZN_200_ZN_1410
3ky9_A_ZN_901_ZN_8812
3ky9_B_ZN_901_ZN_8814
3lrq_A_ZN_100_ZN_2718
3lrq_B_ZN_100_ZN_2720
3lrq_C_ZN_100_ZN_2722
3lrq_D_ZN_100_ZN_2724
3ng2_A_ZN_1003_ZN_1041
3ng2_B_ZN_1001_ZN_1043
3nw0_A_ZN_1_ZN_3481

------- SF_65 --------
1x4s_A_ZN_201_ZN_792
1x4s_A_ZN_401_ZN_793
2d8q_A_ZN_201_ZN_1009
2d8q_A_ZN_401_ZN_1010
2dan_A_ZN_201_ZN_862
2dan_A_ZN_401_ZN_863
2dj8_A_ZN_201_ZN_823
2dj8_A_ZN_401_ZN_824
2egm_A_ZN_200_ZN_836
2egm_A_ZN_300_ZN_837
2ffw_A_ZN_200_ZN_1168
2ffw_A_ZN_201_ZN_1169
2inn_A_ZN_514_ZN_16084
2inn_B_ZN_514_ZN_16087
2jun_A_ZN_220_ZN_1545
2jun_A_ZN_221_ZN_1546
2jun_A_ZN_222_ZN_1547
2jun_A_ZN_223_ZN_1548
2jw6_A_ZN_493_ZN_618
2jw6_A_ZN_494_ZN_619
2od1_A_ZN_901_ZN_732
2od1_A_ZN_902_ZN_733
2odd_A_ZN_1_ZN_926
2odd_A_ZN_2_ZN_927
2w0t_A_ZN_125_ZN_629
2yqp_A_ZN_201_ZN_902
2yqq_A_ZN_201_ZN_815
2yqq_A_ZN_401_ZN_816
2yrg_A_ZN_201_ZN_850
2yrg_A_ZN_401_ZN_851
2yvr_A_ZN_1001_ZN_745
2yvr_A_ZN_1002_ZN_746
2yvr_B_ZN_1003_ZN_747
2yvr_B_ZN_1004_ZN_748
3mek_A_ZN_500_ZN_3356
3mek_A_ZN_501_ZN_3357
3n71_A_ZN_495_ZN_3839
3n71_A_ZN_496_ZN_3840
3pdn_A_ZN_436_ZN_3539
3pdn_A_ZN_437_ZN_3540

------- SF_66 --------
1a1t_A_ZN_56_ZN_1517
1a1t_A_ZN_57_ZN_1518
1a6b_B_ZN_55_ZN_809
1aaf_A_ZN_56_ZN_893
1aaf_A_ZN_57_ZN_894
1bj6_A_ZN_54_ZN_811
1bj6_A_ZN_55_ZN_812
1cl4_A_ZN_81_ZN_493
1dsq_A_ZN_144_ZN_318
1dsv_A_ZN_171_ZN_488
1esk_A_ZN_54_ZN_656
1esk_A_ZN_55_ZN_657
1f6u_A_ZN_128_ZN_1498
1f6u_A_ZN_149_ZN_1499
1hvn_E_ZN_19_ZN_433

1hvo_E_ZN_19_ZN_433
1mfs_A_ZN_56_ZN_867
1mfs_A_ZN_57_ZN_868
1nc8_A_ZN_30_ZN_472
1ncp_C_ZN_61_ZN_536,1ncp_N_ZN_60_ZN_535
1q3y_A_ZN_1_ZN_646
1q3y_A_ZN_2_ZN_647
1q3z_A_ZN_1_ZN_646
1q3z_A_ZN_2_ZN_647
1u6p_A_ZN_57_ZN_4150
1wwd_A_ZN_57_ZN_1085
1wwe_A_ZN_57_ZN_1106
1wwf_A_ZN_57_ZN_1109
1wwg_A_ZN_57_ZN_1079
2a51_A_ZN_54_ZN_599
2a51_A_ZN_55_ZN_600
2bl6_A_ZN_1059_ZN_566
2bl6_A_ZN_1060_ZN_567
2cqf_A_ZN_330_ZN_860
2cqf_A_ZN_530_ZN_861
2di2_A_ZN_30_ZN_468
2e1x_A_ZN_50_ZN_420
2ec7_A_ZN_50_ZN_767
2ec7_A_ZN_51_ZN_768
2exf_A_ZN_56_ZN_680
2exf_A_ZN_57_ZN_681
2ihx_A_ZN_235_ZN_3181
2ihx_A_ZN_236_ZN_3182
2jzw_A_ZN_56_ZN_1122
2jzw_A_ZN_57_ZN_1123
2l4l_A_ZN_56_ZN_828
2l4l_A_ZN_57_ZN_829
2ysa_A_ZN_181_ZN_817
2znf_A_ZN_19_ZN_276
3nyb_B_ZN_2147_ZN_3082
3nyb_B_ZN_2148_ZN_3083

------- SF_67 --------
1a6y_A_ZN_551_ZN_2102
1a6y_B_ZN_451_ZN_2104
1by4_A_ZN_1331_ZN_3834
1by4_B_ZN_1336_ZN_3836
1by4_C_ZN_2331_ZN_3838
1by4_D_ZN_2336_ZN_3840
1cit_A_ZN_399_ZN_1345
1dsz_A_ZN_1122_ZN_1857
1dsz_B_ZN_1222_ZN_1859
1ga5_A_ZN_451_ZN_4001
1ga5_B_ZN_551_ZN_4003
1ga5_E_ZN_451_ZN_4005
1ga5_F_ZN_551_ZN_4007
1gdc_A_ZN_74_ZN_1109
1glu_A_ZN_516_ZN_2032
1glu_B_ZN_516_ZN_2034
1hcp_A_ZN_99_ZN_1171,1hcp_A_ZN_99_ZN_1172
1hcq_A_ZN_599_ZN_3708
1hcq_B_ZN_599_ZN_3710
1hcq_E_ZN_599_ZN_3712

1hcq_F_ZN_599_ZN_3714
1hlz_A_ZN_451_ZN_1980
1hlz_B_ZN_551_ZN_1982
1hra_A_ZN_82_ZN_655
1kb2_A_ZN_151_ZN_2102
1kb2_B_ZN_351_ZN_2104
1kb4_A_ZN_151_ZN_2206
1kb4_B_ZN_351_ZN_2208
1kb6_A_ZN_151_ZN_2239
1kb6_B_ZN_351_ZN_2241
1lat_A_ZN_1515_ZN_1913
1lat_B_ZN_1515_ZN_1915
1lo1_A_ZN_196_ZN_2257
1r0n_A_ZN_178_ZN_2036
1r0n_B_ZN_152_ZN_2038
1r0o_A_ZN_353_ZN_1963
1r0o_B_ZN_352_ZN_1965
1r4i_A_ZN_651_ZN_1815
1r4i_B_ZN_653_ZN_1817
1r4o_A_ZN_527_ZN_2014
1r4o_B_ZN_527_ZN_2016
1r4r_A_ZN_527_ZN_2316
1r4r_B_ZN_527_ZN_2318
1rgd_A_ZN_73_ZN_551
1rxr_A_ZN_214_ZN_1351
1ynw_A_ZN_151_ZN_2072
1ynw_B_ZN_351_ZN_2074
2a66_A_ZN_402_ZN_1241
2c7a_A_ZN_1642_ZN_1899
2c7a_B_ZN_1640_ZN_1901
2ebl_A_ZN_241_ZN_1352
2env_A_ZN_300_ZN_1337
2ff0_A_ZN_1002_ZN_2616
2gda_A_ZN_74_ZN_1109
2han_A_ZN_352_ZN_2166
2han_B_ZN_354_ZN_2168
2nll_A_ZN_251_ZN_2117
2nll_B_ZN_451_ZN_2119
3cbb_A_ZN_1002_ZN_2091
3cbb_B_ZN_2002_ZN_2093
3dzu_A_ZN_7222_ZN_6161
3dzu_D_ZN_7122_ZN_6159
3dzy_A_ZN_7222_ZN_6277
3dzy_D_ZN_7122_ZN_6275
3e00_A_ZN_7222_ZN_6209
3e00_D_ZN_7122_ZN_6207
3fyl_A_ZN_527_ZN_1820
3fyl_B_ZN_527_ZN_1826
3g6p_A_ZN_1515_ZN_1923
3g6p_B_ZN_1513_ZN_1931
3g6q_A_ZN_527_ZN_1819
3g6q_B_ZN_527_ZN_1817
3g6r_A_ZN_1513_ZN_1906
3g6r_B_ZN_1515_ZN_1904
3g6t_A_ZN_528_ZN_1814
3g6t_B_ZN_528_ZN_1816
3g6u_A_ZN_527_ZN_1854
3g6u_B_ZN_527_ZN_1856

3g8u_A_ZN_527_ZN_1832
3g8u_B_ZN_527_ZN_1834
3g8x_A_ZN_527_ZN_1816
3g8x_B_ZN_527_ZN_1826
3g97_A_ZN_527_ZN_1767
3g97_B_ZN_527_ZN_1765
3g99_A_ZN_527_ZN_1824
3g99_B_ZN_527_ZN_1830
3g9i_A_ZN_527_ZN_1868
3g9i_B_ZN_527_ZN_1878
3g9j_A_ZN_1515_ZN_1914
3g9j_B_ZN_1513_ZN_1920
3g9m_A_ZN_527_ZN_1900
3g9m_B_ZN_527_ZN_1910
3g9o_A_ZN_527_ZN_1817
3g9o_B_ZN_527_ZN_1815
3g9p_A_ZN_527_ZN_1817
3g9p_B_ZN_527_ZN_1815
3m9e_A_ZN_209_ZN_5079
3m9e_B_ZN_209_ZN_5081
3m9e_E_ZN_209_ZN_5083
3m9e_F_ZN_209_ZN_5085

------- SF_68 --------
1jw9_B_ZN_250_ZN_2410
1jwb_B_ZN_250_ZN_2406
1r4m_F_ZN_3_ZN_31631
1r4m_H_ZN_1_ZN_31629
1r4n_F_ZN_3_ZN_31631
1r4n_H_ZN_1_ZN_31629
1tt5_B_ZN_1014_ZN_14344
1tt5_D_ZN_1014_ZN_14345
1y8q_B_ZN_642_ZN_12937
1y8q_D_ZN_642_ZN_12970
1y8r_B_ZN_642_ZN_14320
1y8r_E_ZN_642_ZN_14353
1yov_B_ZN_443_ZN_14457
1yov_D_ZN_443_ZN_14458
1zfn_A_ZN_252_ZN_7192
1zfn_B_ZN_252_ZN_7193
1zfn_C_ZN_345_ZN_7194
1zfn_D_ZN_445_ZN_7195
1zkm_A_ZN_252_ZN_7190
1zkm_B_ZN_252_ZN_7191
1zkm_C_ZN_252_ZN_7192
1zkm_D_ZN_252_ZN_7193
1zud_1_ZN_501_ZN_4703
1zud_3_ZN_502_ZN_4706
2nvu_B_ZN_102_ZN_13019
3dbh_B_ZN_1_ZN_32587
3dbh_D_ZN_3_ZN_32589
3dbh_F_ZN_4_ZN_32590
3dbh_H_ZN_2_ZN_32588
3dbl_B_ZN_1_ZN_32586
3dbl_D_ZN_3_ZN_32588
3dbl_F_ZN_4_ZN_32589
3dbl_H_ZN_2_ZN_32587
3dbr_B_ZN_1_ZN_32433

3dbr_D_ZN_3_ZN_32435
3dbr_F_ZN_4_ZN_32436
3dbr_H_ZN_2_ZN_32434
3guc_A_ZN_401_ZN_3528
3guc_B_ZN_401_ZN_3556
3gzn_B_ZN_465_ZN_16055
3gzn_D_ZN_465_ZN_16056
3h5a_A_ZN_360_ZN_11065
3h5a_B_ZN_360_ZN_11066
3h5a_C_ZN_360_ZN_11067
3h5a_D_ZN_360_ZN_11068
3h5n_A_ZN_500_ZN_10429
3h5n_B_ZN_500_ZN_10467
3h5n_C_ZN_500_ZN_10500
3h5n_D_ZN_500_ZN_10533
3h5r_A_ZN_500_ZN_10444
3h5r_B_ZN_500_ZN_10445
3h5r_C_ZN_500_ZN_10451
3h5r_D_ZN_500_ZN_10457
3h8v_A_ZN_401_ZN_3426
3h8v_B_ZN_401_ZN_3458
3h9g_A_ZN_500_ZN_10776
3h9g_B_ZN_500_ZN_10782
3h9g_C_ZN_500_ZN_10793
3h9g_D_ZN_500_ZN_10804
3h9j_A_ZN_500_ZN_10492
3h9j_B_ZN_500_ZN_10524
3h9j_C_ZN_500_ZN_10561
3h9j_D_ZN_500_ZN_10624
3h9q_A_ZN_500_ZN_10419
3h9q_B_ZN_500_ZN_10420
3h9q_C_ZN_500_ZN_10431
3h9q_D_ZN_500_ZN_10432
3kyc_B_ZN_641_ZN_7408
3kyd_B_ZN_550_ZN_6736

------- SF_69 --------
2ecg_A_ZN_201_ZN_1168
2ecg_A_ZN_401_ZN_1169
2hdp_A_ZN_492_ZN_1997
2hdp_B_ZN_492_ZN_1999
2k4d_A_ZN_500_ZN_1259
2k4d_A_ZN_600_ZN_1260
2vje_A_ZN_1492_ZN_1891
2vje_A_ZN_1493_ZN_1892
2vje_B_ZN_1491_ZN_1893
2vje_B_ZN_1492_ZN_1894
2vje_C_ZN_1492_ZN_1895
2vje_C_ZN_1493_ZN_1896
2vje_D_ZN_1491_ZN_1897
2vje_D_ZN_1492_ZN_1898
2vjf_A_ZN_1492_ZN_1965
2vjf_A_ZN_1493_ZN_1966
2vjf_B_ZN_1491_ZN_1967
2vjf_B_ZN_1492_ZN_1968
2vjf_C_ZN_1492_ZN_1969
2vjf_C_ZN_1493_ZN_1970
2vjf_D_ZN_1491_ZN_1971

2vjf_D_ZN_1492_ZN_1972
3eb5_A_ZN_1001_ZN_525
3eb5_A_ZN_1002_ZN_526
3eb6_A_ZN_1001_ZN_1694
3eb6_A_ZN_1002_ZN_1695

------- SF_70 --------
1wfe_A_ZN_201_ZN_1286
1wfe_A_ZN_401_ZN_1287
1wff_A_ZN_201_ZN_1252
1wff_A_ZN_401_ZN_1253
1wfh_A_ZN_201_ZN_895
1wfh_A_ZN_401_ZN_896
1wfl_A_ZN_201_ZN_1078
1wfl_A_ZN_401_ZN_1079
1wfp_A_ZN_201_ZN_1006
1wfp_A_ZN_401_ZN_1007
1wg2_A_ZN_200_ZN_911
1wg2_A_ZN_400_ZN_912
1wys_A_ZN_201_ZN_1082
1wys_A_ZN_401_ZN_1083
1x4v_A_ZN_201_ZN_925
1x4v_A_ZN_401_ZN_926
1x4w_A_ZN_201_ZN_1000
1x4w_A_ZN_401_ZN_1001

------- SF_71 --------
2csv_A_ZN_200_ZN_1066
2d8u_A_ZN_201_ZN_910
2d8v_A_ZN_201_ZN_949
2did_A_ZN_201_ZN_734
2dif_A_ZN_201_ZN_734
2dja_A_ZN_201_ZN_1265
2dq5_A_ZN_1175_ZN_730
3ddt_A_ZN_46_ZN_1055
3ddt_B_ZN_46_ZN_1057
3ddt_C_ZN_46_ZN_1059

------- SF_72 --------
2d8r_A_ZN_401_ZN_1532
2jm3_A_ZN_92_ZN_1492
2jtg_A_ZN_88_ZN_1436
2ko0_A_ZN_88_ZN_2450
2l1g_A_ZN_88_ZN_2450
3kde_C_ZN_78_ZN_1005

------- SF_73 --------
1e7d_A_ZN_1158_ZN_2547
1e7d_B_ZN_1158_ZN_2550
1e7l_A_ZN_1165_ZN_2790
1e7l_B_ZN_1165_ZN_2831
1en7_A_ZN_401_ZN_2547
1en7_B_ZN_402_ZN_2549
1v0d_A_ZN_401_ZN_1938
2qnc_A_ZN_158_ZN_4444
2qnc_B_ZN_158_ZN_4454
2qnf_A_ZN_158_ZN_3847
2qnf_B_ZN_158_ZN_3848

3fc3_A_ZN_302_ZN_3792
3fc3_B_ZN_304_ZN_3808
3gox_A_ZN_302_ZN_3918
3gox_B_ZN_304_ZN_3937

------- SF_74 --------
1ef4_A_ZN_56_ZN_890
1i3q_J_ZN_2001_ZN_28163
1i50_J_ZN_3001_ZN_28297
1i6h_J_ZN_101_ZN_28434
1k83_J_ZN_3001_ZN_27843
1nik_J_ZN_101_ZN_28307
1r5u_J_ZN_101_ZN_28308
1r9s_J_ZN_101_ZN_28471
1r9t_J_ZN_101_ZN_29227
1sfo_J_ZN_101_ZN_28657
1twa_J_ZN_3001_ZN_27729
1twc_J_ZN_3001_ZN_27757
1twf_J_ZN_3001_ZN_28290
1twg_J_ZN_3001_ZN_27735
1twh_J_ZN_3001_ZN_27700
1wcm_J_ZN_1066_ZN_30956
1y1v_J_ZN_101_ZN_31814
1y1w_J_ZN_101_ZN_31815
1y77_J_ZN_101_ZN_31816
2b63_J_ZN_1066_ZN_31743
2b8k_J_ZN_71_ZN_31051
2e2h_J_ZN_101_ZN_28981
2e2i_J_ZN_101_ZN_29671
2e2j_J_ZN_101_ZN_29184
2ja5_A_ZN_2458_ZN_31667
2ja6_A_ZN_2458_ZN_32019
2ja7_A_ZN_2459_ZN_63939
2ja7_A_ZN_2460_ZN_63940
2ja8_A_ZN_2458_ZN_32008
2nvq_J_ZN_101_ZN_29378
2nvt_J_ZN_101_ZN_29146
2nvx_J_ZN_101_ZN_29391
2nvy_J_ZN_3001_ZN_28290
2nvz_J_ZN_101_ZN_28983
2pmz_N_ZN_1001_ZN_48124
2pmz_Y_ZN_1001_ZN_48129
2r7z_J_ZN_101_ZN_31817
2r92_J_ZN_101_ZN_31623
2r93_J_ZN_101_ZN_31511
2vum_J_ZN_1066_ZN_32098
2wb1_N_ZN_1065_ZN_52750
2wb1_O_ZN_1065_ZN_52751
2yu9_J_ZN_101_ZN_29482
3cqz_J_ZN_3001_ZN_27333
3fki_J_ZN_71_ZN_31422
3gtg_J_ZN_101_ZN_30077
3gtj_J_ZN_101_ZN_29985
3gtk_J_ZN_101_ZN_30123
3gtl_J_ZN_101_ZN_29267
3gtm_J_ZN_101_ZN_30572
3gto_J_ZN_101_ZN_29270
3gtp_J_ZN_101_ZN_29290

3gtq_J_ZN_101_ZN_28636
3h0g_J_ZN_1066_ZN_62884
3h0g_V_ZN_1066_ZN_62893
3h3v_K_ZN_71_ZN_31790
3hou_J_ZN_9990_ZN_63685
3hou_V_ZN_9998_ZN_63693
3hov_J_ZN_1066_ZN_31791
3how_J_ZN_1066_ZN_31890
3hox_J_ZN_1066_ZN_31932
3hoy_J_ZN_1066_ZN_31817
3hoz_J_ZN_1066_ZN_31975
3i4m_J_ZN_1066_ZN_32369
3i4n_J_ZN_1066_ZN_32321
3k1f_J_ZN_3001_ZN_32343
3k7a_J_ZN_101_ZN_29038

------- SF_75 --------
2d6f_C_ZN_900_ZN_17641
2d6f_D_ZN_1900_ZN_17642
3h0l_B_ZN_901_ZN_62901
3h0l_E_ZN_902_ZN_62930
3h0l_H_ZN_903_ZN_62959
3h0l_K_ZN_904_ZN_62988
3h0l_N_ZN_905_ZN_63017
3h0l_Q_ZN_906_ZN_63046
3h0l_T_ZN_907_ZN_63075
3h0l_W_ZN_908_ZN_63104
3h0m_B_ZN_901_ZN_62952
3h0m_E_ZN_902_ZN_62953
3h0m_H_ZN_903_ZN_62954
3h0m_K_ZN_904_ZN_62955
3h0m_N_ZN_905_ZN_62956
3h0m_Q_ZN_906_ZN_62957
3h0m_T_ZN_907_ZN_62958
3h0m_W_ZN_908_ZN_62959
3h0r_B_ZN_901_ZN_62929
3h0r_E_ZN_902_ZN_62966
3h0r_H_ZN_903_ZN_63001
3h0r_K_ZN_904_ZN_63035
3h0r_N_ZN_905_ZN_63109
3h0r_Q_ZN_906_ZN_63153
3h0r_T_ZN_907_ZN_63184
3h0r_W_ZN_908_ZN_63218
3kfu_F_ZN_673_ZN_33441
3kfu_I_ZN_467_ZN_33443

------- SF_76 --------
1ozb_I_ZN_50_ZN_9153
1ozb_J_ZN_51_ZN_9154
1sx1_A_ZN_23_ZN_335
1tm6_A_ZN_23_ZN_333
2i9w_A_ZN_200_ZN_1392
2i9w_A_ZN_201_ZN_1393
2jq5_A_ZN_129_ZN_1997

------- SF_77 --------
1gzh_A_ZN_1293_ZN_6513
1gzh_C_ZN_1290_ZN_6519

1hu8_A_ZN_501_ZN_4387
1hu8_B_ZN_502_ZN_4388
1hu8_C_ZN_503_ZN_4389
1kzy_A_ZN_1_ZN_6773
1kzy_B_ZN_1_ZN_6774
1t4w_A_ZN_201_ZN_1654
1tsr_A_ZN_1_ZN_5447
1tsr_B_ZN_1_ZN_5448
1tsr_C_ZN_1_ZN_5449
1tup_A_ZN_951_ZN_5447
1tup_B_ZN_952_ZN_5448
1tup_C_ZN_953_ZN_5449
1uol_A_ZN_1300_ZN_3075
1uol_B_ZN_1300_ZN_3076
1ycs_A_ZN_1_ZN_3018
2ac0_A_ZN_1_ZN_7198
2ac0_B_ZN_1_ZN_7199
2ac0_C_ZN_1_ZN_7200
2ac0_D_ZN_1_ZN_7201
2ady_A_ZN_1_ZN_3582
2ady_B_ZN_1_ZN_3583
2ahi_A_ZN_1_ZN_7065
2ahi_B_ZN_1_ZN_7066
2ahi_C_ZN_1_ZN_7067
2ahi_D_ZN_1_ZN_7068
2ata_A_ZN_1_ZN_7078
2ata_B_ZN_1_ZN_7079
2ata_C_ZN_1_ZN_7080
2ata_D_ZN_1_ZN_7081
2bim_A_ZN_1291_ZN_3073
2bim_B_ZN_1291_ZN_3079
2bin_A_ZN_1292_ZN_1456
2bio_A_ZN_1291_ZN_1482
2bip_A_ZN_1291_ZN_1467
2biq_A_ZN_1291_ZN_1472
2fej_A_ZN_1_ZN_3180
2geq_A_ZN_1_ZN_3584
2geq_B_ZN_2_ZN_3593
2h1l_M_ZN_13_ZN_53933
2h1l_N_ZN_14_ZN_53934
2h1l_O_ZN_15_ZN_53935
2h1l_P_ZN_16_ZN_53936
2h1l_Q_ZN_17_ZN_53937
2h1l_R_ZN_18_ZN_53938
2h1l_S_ZN_19_ZN_53939
2h1l_T_ZN_20_ZN_53940
2h1l_U_ZN_21_ZN_53941
2h1l_V_ZN_22_ZN_53942
2h1l_W_ZN_23_ZN_53943
2h1l_X_ZN_24_ZN_53944
2ioi_A_ZN_3001_ZN_1473
2iom_A_ZN_2001_ZN_1473
2ioo_A_ZN_3001_ZN_1473
2j1w_A_ZN_313_ZN_3097
2j1w_B_ZN_313_ZN_3098
2j1x_A_ZN_313_ZN_3101
2j1x_B_ZN_313_ZN_3102
2j1y_A_ZN_1290_ZN_6077

2j1y_B_ZN_1290_ZN_6079
2j1y_C_ZN_1290_ZN_6081
2j1y_D_ZN_1290_ZN_6083
2j1z_A_ZN_1291_ZN_3095
2j1z_B_ZN_1291_ZN_3096
2j20_A_ZN_1291_ZN_3073
2j20_B_ZN_1291_ZN_3079
2j21_A_ZN_1289_ZN_3009
2j21_B_ZN_1290_ZN_3010
2ocj_A_ZN_501_ZN_6097
2ocj_B_ZN_502_ZN_6098
2ocj_C_ZN_503_ZN_6099
2ocj_D_ZN_504_ZN_6100
2pcx_A_ZN_308_ZN_1692
2qvq_A_ZN_1_ZN_1547
2qxa_A_ZN_1_ZN_6123
2qxa_B_ZN_2_ZN_6124
2qxa_C_ZN_3_ZN_6125
2qxa_D_ZN_4_ZN_6126
2qxb_A_ZN_1_ZN_6105
2qxb_B_ZN_2_ZN_6106
2qxb_C_ZN_3_ZN_6107
2qxb_D_ZN_4_ZN_6108
2qxc_A_ZN_1_ZN_6117
2qxc_B_ZN_2_ZN_6118
2qxc_C_ZN_3_ZN_6119
2qxc_D_ZN_4_ZN_6120
2rmn_A_ZN_1_ZN_3593
2vuk_A_ZN_1300_ZN_3122
2vuk_B_ZN_1300_ZN_3141
2wgx_A_ZN_1300_ZN_3105
2wgx_B_ZN_1300_ZN_3106
2x0u_A_ZN_1300_ZN_3108
2x0u_B_ZN_1300_ZN_3123
2x0v_A_ZN_1300_ZN_3092
2x0v_B_ZN_1300_ZN_3117
2x0w_A_ZN_1300_ZN_3083
2x0w_B_ZN_1300_ZN_3098
2xwc_A_ZN_1319_ZN_1592
3d05_A_ZN_1_ZN_1446
3d06_A_ZN_1_ZN_1500
3d07_A_ZN_1_ZN_3058
3d07_B_ZN_1_ZN_3059
3d08_A_ZN_1_ZN_1511
3d09_A_ZN_1_ZN_1519
3d0a_A_ZN_1_ZN_7111
3d0a_B_ZN_1_ZN_7112
3d0a_C_ZN_1_ZN_7113
3d0a_D_ZN_1_ZN_7114
3exj_A_ZN_1_ZN_3540
3exj_B_ZN_2_ZN_3554
3exl_A_ZN_1_ZN_1739
3igk_A_ZN_1_ZN_1804
3igl_A_ZN_1_ZN_1788
3kmd_A_ZN_1_ZN_7067
3kmd_B_ZN_1_ZN_7068
3kmd_C_ZN_1_ZN_7070
3kmd_D_ZN_1_ZN_7069

3kz8_A_ZN_1_ZN_3472
3kz8_B_ZN_1_ZN_3474

------- SF_78 --------
1h4q_A_ZN_1479_ZN_8950
1h4q_B_ZN_1479_ZN_8994
1h4s_A_ZN_490_ZN_9083
1h4s_B_ZN_490_ZN_9124
1h4t_A_ZN_490_ZN_15025
1h4t_B_ZN_490_ZN_15026
1h4t_C_ZN_490_ZN_15027
1h4t_D_ZN_490_ZN_15028
1hc7_A_ZN_490_ZN_14993
1hc7_B_ZN_490_ZN_14994
1hc7_C_ZN_490_ZN_14995
1hc7_D_ZN_490_ZN_14996
1nj1_A_ZN_513_ZN_3784
1nj2_A_ZN_495_ZN_3695
1nj5_A_ZN_511_ZN_3784
1nj6_A_ZN_509_ZN_3784

------- SF_79 --------
1jjd_A_ZN_101_ZN_690,1jjd_A_ZN_102_ZN_691,1jjd_A_ZN_103_ZN_692,1jjd_A_ZN_
104_ZN_693
4mt2_A_ZN_67_ZN_410,4mt2_A_ZN_68_ZN_411

------- SF_80 --------
1pxe_A_ZN_64_ZN_724
2cs8_A_ZN_401_ZN_1543
2cs8_A_ZN_601_ZN_1544
2jyd_A_ZN_47_ZN_640

------- SF_81 --------
2x7m_A_ZN_1175_ZN_2888
3p1v_A_ZN_427_ZN_6593
3p1v_B_ZN_427_ZN_6624

------- SF_82 --------
2kwq_A_ZN_844_ZN_1474
3ebe_A_ZN_500_ZN_4132
3ebe_B_ZN_501_ZN_4133
3ebe_C_ZN_502_ZN_4134
3h15_A_ZN_500_ZN_1451

------- SF_83 --------
2kgg_A_ZN_53_ZN_747
2kgi_A_ZN_53_ZN_912
2vp7_A_ZN_1400_ZN_783
2vpb_A_ZN_1401_ZN_712
2vpd_A_ZN_1400_ZN_1458
2vpe_A_ZN_1401_ZN_1486
2vpe_C_ZN_1399_ZN_1488
2vpg_A_ZN_1401_ZN_1504
2xb1_A_ZN_1391_ZN_1514
2xb1_C_ZN_1391_ZN_1522
3gl6_A_ZN_3_ZN_471

------- SF_84 --------

1ffy_A_ZN_1001_ZN_9024
1qu2_A_ZN_1001_ZN_9024

------- SF_85 --------
1i3j_A_ZN_100_ZN_1641
1t2t_A_ZN_100_ZN_1628

------- SF_86 --------
1irx_A_ZN_600_ZN_8505
1irx_B_ZN_600_ZN_8507

------- SF_87 --------
1ul4_A_ZN_138_ZN_1328
1ul4_A_ZN_139_ZN_1329
1ul5_A_ZN_221_ZN_1396
1ul5_A_ZN_222_ZN_1397
1wj0_A_ZN_182_ZN_906

------- SF_88 --------
1urj_A_ZN_2131_ZN_15696
1urj_B_ZN_2131_ZN_15701

------- SF_89 --------
1vdd_A_ZN_230_ZN_6037
1vdd_B_ZN_231_ZN_6043
1vdd_C_ZN_232_ZN_6049
1vdd_D_ZN_233_ZN_6055
2v1c_A_ZN_230_ZN_4782
2v1c_B_ZN_230_ZN_4783

------- SF_90 --------
1z60_A_ZN_1_ZN_878

------- SF_91 --------
1gpc_A_ZN_1_ZN_2146
2a1k_A_ZN_1_ZN_3437
2a1k_B_ZN_2_ZN_3438
2atq_B_ZN_1_ZN_8610

------- SF_92 --------
2ac3_A_ZN_531_ZN_2217
2ac5_A_ZN_386_ZN_2243
2hw7_A_ZN_41_ZN_2165

------- SF_93 --------
2avu_E_ZN_400_ZN_5322
2avu_F_ZN_300_ZN_5321

------- SF_94 --------
2dkt_A_ZN_191_ZN_2164
2k2c_A_ZN_139_ZN_2125

------- SF_95 --------
2e61_A_ZN_201_ZN_1012
2rr4_A_ZN_501_ZN_1196

------- SF_96 --------
2ea5_A_ZN_201_ZN_966

------- SF_97 --------
2fe3_A_ZN_201_ZN_2275
2fe3_B_ZN_202_ZN_2276
2rgv_A_ZN_146_ZN_2278
2rgv_B_ZN_146_ZN_2279
3f8n_A_ZN_202_ZN_2165
3f8n_B_ZN_204_ZN_2167

------- SF_98 --------
2hqh_E_ZN_1500_ZN_2934
2hqh_F_ZN_1501_ZN_2935
2hqh_G_ZN_1502_ZN_2936
2hqh_H_ZN_1503_ZN_2937
3e2u_E_ZN_102_ZN_3032
3e2u_F_ZN_101_ZN_3033
3e2u_G_ZN_103_ZN_3034
3e2u_H_ZN_104_ZN_3035

------- SF_99 --------
2hye_B_ZN_3002_ZN_16944

------- SF_100 --------
2inp_A_ZN_2_ZN_16003
2inp_B_ZN_500_ZN_16007

------- SF_101 --------
1fjg_D_ZN_300_ZN_51954
1hnw_D_ZN_300_ZN_51891
1hnx_D_ZN_300_ZN_51892
1hnz_D_ZN_300_ZN_51892
1hr0_D_ZN_300_ZN_52388
1ibk_D_ZN_306_ZN_51893
1ibl_D_ZN_306_ZN_52293
1ibm_D_ZN_506_ZN_52183
1j5e_D_ZN_306_ZN_51765
1n32_D_ZN_306_ZN_52297
1n33_D_ZN_306_ZN_52162
1n34_D_ZN_306_ZN_51778
1n36_D_ZN_306_ZN_51700
1xmo_D_ZN_306_ZN_52084
1xmq_D_ZN_306_ZN_52102
1xnq_D_ZN_306_ZN_52097
1xnr_D_ZN_306_ZN_52095
2e5l_D_ZN_210_ZN_51917
2f4v_D_ZN_306_ZN_51569
2j00_Z_ZN_742_ZN_56164
2j02_Z_ZN_639_ZN_56061
2uu9_Z_ZN_1121_ZN_52297
2uua_Z_ZN_138_ZN_52273
2uub_Z_ZN_1138_ZN_52269
2uuc_Z_ZN_1120_ZN_52273
2uxb_Z_ZN_143_ZN_52188
2uxc_Z_ZN_220_ZN_52301
2uxd_Z_ZN_80_ZN_51492
2v46_Z_ZN_742_ZN_55964
2v48_Z_ZN_742_ZN_55964
2vqe_Z_ZN_140_ZN_52096

2wdg_Z_ZN_742_ZN_57353
2wdh_Z_ZN_742_ZN_57353
2wdk_Z_ZN_742_ZN_57291
2wdm_Z_ZN_742_ZN_57291
2wh1_Z_ZN_547_ZN_58234
2wh3_Z_ZN_547_ZN_58234
2wri_Z_ZN_5_ZN_60240
2wrk_Z_ZN_5_ZN_60240
2wrn_1_ZN_3_ZN_59574
2wrq_1_ZN_3_ZN_59574
2xfz_Z_ZN_1_ZN_55961
2xg1_Z_ZN_1_ZN_55151
2xqd_1_ZN_5_ZN_59826
2zm6_D_ZN_210_ZN_51328
3d5a_D_ZN_210_ZN_57810
3d5c_D_ZN_210_ZN_57812
3f1e_D_ZN_5001_ZN_57772
3f1g_D_ZN_5001_ZN_57772
3i8g_G_ZN_210_ZN_58408
3i8h_G_ZN_210_ZN_58219
3i9b_G_ZN_210_ZN_56865
3i9d_G_ZN_210_ZN_58414
3kiq_d_ZN_611_ZN_56000
3kis_d_ZN_611_ZN_55994
3kiu_d_ZN_210_ZN_56010
3kix_d_ZN_611_ZN_56010
3mr8_D_ZN_210_ZN_56071
3ms0_D_ZN_210_ZN_56115
3oge_D_ZN_210_ZN_51497
3ogy_D_ZN_210_ZN_51494
3ohc_D_ZN_210_ZN_51495
3ohd_D_ZN_210_ZN_51491
3ohy_D_ZN_210_ZN_51492
3oi0_D_ZN_210_ZN_51489
3oi2_D_ZN_210_ZN_51493
3oi4_D_ZN_210_ZN_51491
3oto_D_ZN_210_ZN_51659

------- SF_102 --------
2jmi_A_ZN_201_ZN_926
2jmi_A_ZN_401_ZN_927

------- SF_103 --------
2k0a_A_ZN_108_ZN_1716
2k0a_A_ZN_110_ZN_1718

------- SF_104 --------
2k0a_A_ZN_109_ZN_1717

------- SF_105 --------
2k16_A_ZN_940_ZN_1147
2k16_A_ZN_941_ZN_1148
2k17_A_ZN_940_ZN_1356
2k17_A_ZN_941_ZN_1357

------- SF_106 --------
2kdp_A_ZN_1_ZN_1157

------- SF_107 --------
2kgg_A_ZN_54_ZN_748
2kgi_A_ZN_54_ZN_913
3gl6_A_ZN_2_ZN_470

------- SF_108 --------
2kgo_A_ZN_109_ZN_1334

------- SF_109 --------
2o03_A_ZN_202_ZN_957

------- SF_110 --------
2ro1_A_ZN_201_ZN_2907

------- SF_111 --------
2jwo_A_ZN_488_ZN_1253
2v83_A_ZN_1486_ZN_1943
2v85_A_ZN_1488_ZN_1410
2v85_B_ZN_1487_ZN_1412
2v85_B_ZN_1488_ZN_1413
2v87_A_ZN_1487_ZN_1435
2v89_B_ZN_1488_ZN_2726

------- SF_112 --------
2jwo_A_ZN_489_ZN_1254
2v83_B_ZN_1489_ZN_1946
2v83_C_ZN_1485_ZN_1948
2v85_A_ZN_1489_ZN_1411
2v87_A_ZN_1488_ZN_1436
2v87_B_ZN_1488_ZN_1437
2v87_B_ZN_1489_ZN_1438
2v88_A_ZN_1489_ZN_1409
2v88_B_ZN_1489_ZN_1411
2v89_B_ZN_1489_ZN_2727

------- SF_113 --------
2v9k_A_ZN_1533_ZN_3708

------- SF_114 --------
1adu_A_ZN_530_ZN_4533
1adu_A_ZN_531_ZN_4534
1adu_B_ZN_530_ZN_4535
1adu_B_ZN_531_ZN_4536
1adv_A_ZN_530_ZN_4533
1adv_A_ZN_531_ZN_4534
1adv_B_ZN_530_ZN_4535
1adv_B_ZN_531_ZN_4536
1anv_A_ZN_530_ZN_2465
1anv_A_ZN_531_ZN_2466
2waz_X_ZN_601_ZN_2485
2waz_X_ZN_602_ZN_2486
2wb0_X_ZN_601_ZN_2479
2wb0_X_ZN_602_ZN_2480

------- SF_115 --------
2iyk_A_ZN_1163_ZN_2455
2iyk_B_ZN_1163_ZN_2458
2wjv_A_ZN_1_ZN_13071

2wjv_B_ZN_1_ZN_13139
2wjy_A_ZN_1_ZN_6170

------- SF_116 --------
2iyk_A_ZN_1165_ZN_2457
2iyk_B_ZN_1165_ZN_2460
2wjv_A_ZN_3_ZN_13073
2wjv_B_ZN_3_ZN_13141
2wjy_A_ZN_3_ZN_6172

------- SF_117 --------
2x5r_A_ZN_1126_ZN_970

------- SF_118 --------
2zze_A_ZN_753_ZN_12283
2zze_B_ZN_753_ZN_12284
2zzf_A_ZN_754_ZN_5929
2zzg_A_ZN_900_ZN_11996
2zzg_B_ZN_900_ZN_12026

------- SF_119 --------
3fc3_A_ZN_301_ZN_3791
3fc3_B_ZN_303_ZN_3807
3gox_A_ZN_301_ZN_3917
3gox_B_ZN_303_ZN_3936

------- SF_120 --------
3h0n_A_ZN_201_ZN_1501

------- SF_121 --------
3kni_4_ZN_5_ZN_90944
3knk_4_ZN_6_ZN_90778
3knm_4_ZN_1_ZN_90730
3kno_4_ZN_1_ZN_90400

------- SF_122 --------
3pt6_A_ZN_3_ZN_15518
3pt6_B_ZN_1_ZN_15520
3pt9_A_ZN_2_ZN_6501
3pta_A_ZN_3_ZN_7991

------- SF_123 --------
1af2_A_ZN_296_ZN_2222
1aln_A_ZN_296_ZN_2222
1ctt_A_ZN_296_ZN_2222
1ctu_A_ZN_296_ZN_2222
1jtk_A_ZN_137_ZN_1979
1jtk_B_ZN_137_ZN_1996
1mq0_A_ZN_147_ZN_1925
1mq0_B_ZN_147_ZN_1943
1ox7_A_ZN_401_ZN_2461,1ox7_A_ZN_402_ZN_2462
1ox7_B_ZN_400_ZN_2463,1ox7_B_ZN_403_ZN_2464
1p6o_A_ZN_401_ZN_2510
1p6o_B_ZN_400_ZN_2523
1r5t_A_ZN_150_ZN_4200
1r5t_B_ZN_151_ZN_4201
1r5t_C_ZN_152_ZN_4202
1r5t_D_ZN_153_ZN_4203

1rb7_A_ZN_400_ZN_2369,1rb7_A_ZN_402_ZN_2370
1rb7_B_ZN_401_ZN_2371,1rb7_B_ZN_403_ZN_2372
1tiy_A_ZN_200_ZN_2371
1tiy_B_ZN_201_ZN_2372
1uaq_A_ZN_200_ZN_2359
1uaq_B_ZN_200_ZN_2368
1uwz_A_ZN_1131_ZN_1963
1uwz_B_ZN_1131_ZN_1980
1ux0_A_ZN_1131_ZN_1971
1ux0_B_ZN_1131_ZN_1988
1ux1_A_ZN_1132_ZN_3961
1ux1_B_ZN_1131_ZN_3986
1ux1_C_ZN_1131_ZN_4003
1ux1_D_ZN_1132_ZN_4020
1vq2_A_ZN_701_ZN_1322
1wkq_A_ZN_201_ZN_2436
1wkq_B_ZN_202_ZN_2442
1wn5_A_ZN_1001_ZN_3609
1wn5_B_ZN_1002_ZN_3610
1wn5_C_ZN_1003_ZN_3616
1wn5_D_ZN_1004_ZN_3617
1wn6_A_ZN_1001_ZN_1834
1wn6_B_ZN_1002_ZN_1835
1wwr_A_ZN_201_ZN_4976
1wwr_B_ZN_202_ZN_4977
1wwr_C_ZN_203_ZN_4978
1wwr_D_ZN_204_ZN_4979
1ysb_A_ZN_501_ZN_2454,1ysb_A_ZN_503_ZN_2455
1ysb_B_ZN_500_ZN_2456,1ysb_B_ZN_502_ZN_2457
1ysd_A_ZN_500_ZN_2452,1ysd_A_ZN_501_ZN_2453
1ysd_B_ZN_502_ZN_2454,1ysd_B_ZN_503_ZN_2455
1z3a_A_ZN_301_ZN_2459
1z3a_B_ZN_302_ZN_2460
1zab_A_ZN_147_ZN_4257
1zab_B_ZN_147_ZN_4280
1zab_C_ZN_147_ZN_4298
1zab_D_ZN_147_ZN_4316
1zy7_A_ZN_801_ZN_5806
1zy7_B_ZN_802_ZN_5843
2a8n_A_ZN_301_ZN_2034
2a8n_B_ZN_302_ZN_2035
2b3j_A_ZN_2001_ZN_5672
2b3j_B_ZN_2002_ZN_5679
2b3j_C_ZN_2003_ZN_5686
2b3j_D_ZN_2004_ZN_5693
2b3z_A_ZN_1360_ZN_10975
2b3z_B_ZN_1360_ZN_10976
2b3z_C_ZN_1360_ZN_10977
2b3z_D_ZN_1360_ZN_10978
2d30_A_ZN_2001_ZN_1883
2d30_B_ZN_1001_ZN_1884
2d5n_A_ZN_1360_ZN_10975
2d5n_B_ZN_1360_ZN_10976
2d5n_C_ZN_1360_ZN_11025
2d5n_D_ZN_1360_ZN_11026
2fr5_A_ZN_147_ZN_4309
2fr5_B_ZN_147_ZN_4332
2fr5_C_ZN_147_ZN_4350

2fr5_D_ZN_147_ZN_4368
2fr6_A_ZN_147_ZN_4260
2fr6_B_ZN_147_ZN_4283
2fr6_C_ZN_147_ZN_4302
2fr6_D_ZN_147_ZN_4320
2g84_A_ZN_506_ZN_2885
2g84_B_ZN_507_ZN_2894
2hvv_A_ZN_501_ZN_2263
2hvv_B_ZN_503_ZN_2286
2hvw_A_ZN_1002_ZN_3525
2hvw_B_ZN_1004_ZN_3582
2hvw_C_ZN_1006_ZN_3639
2hxv_A_ZN_500_ZN_2718
2jyw_A_ZN_188_ZN_3004
2kbo_A_ZN_250_ZN_3129
2kem_A_ZN_195_ZN_3127
2nx8_A_ZN_999_ZN_1328
2nyt_A_ZN_2000_ZN_6001
2nyt_B_ZN_2001_ZN_6002
2nyt_C_ZN_2002_ZN_6003
2nyt_D_ZN_2003_ZN_6004
2o3k_A_ZN_401_ZN_2399
2o3k_B_ZN_400_ZN_2408
2rpz_A_ZN_301_ZN_3040
2w4l_A_ZN_1170_ZN_7284
2w4l_B_ZN_1171_ZN_7287
2w4l_C_ZN_1170_ZN_7289
2w4l_D_ZN_1170_ZN_7290
2w4l_E_ZN_1174_ZN_7291
2w4l_F_ZN_1171_ZN_7293
2z3g_A_ZN_2001_ZN_3706
2z3g_B_ZN_2002_ZN_3707
2z3g_C_ZN_2003_ZN_3731
2z3g_D_ZN_2004_ZN_3732
2z3h_A_ZN_2001_ZN_3708
2z3h_B_ZN_2002_ZN_3739
2z3h_C_ZN_2003_ZN_3770
2z3h_D_ZN_2004_ZN_3801
2z3i_A_ZN_2001_ZN_3823
2z3i_B_ZN_2002_ZN_3859
2z3i_C_ZN_2003_ZN_3895
2z3i_D_ZN_2004_ZN_3931
2z3j_A_ZN_2001_ZN_3643
2z3j_B_ZN_2002_ZN_3650
2z3j_C_ZN_2003_ZN_3656
2z3j_D_ZN_2004_ZN_3662
3dh1_A_ZN_201_ZN_5048
3dh1_B_ZN_201_ZN_5049
3dh1_C_ZN_201_ZN_5050
3dh1_D_ZN_201_ZN_5051
3dmo_A_ZN_131_ZN_3859
3dmo_B_ZN_131_ZN_3868
3dmo_C_ZN_131_ZN_3882
3dmo_D_ZN_131_ZN_3891
3e1u_A_ZN_1001_ZN_1525
3ex8_A_ZN_362_ZN_10975
3ex8_B_ZN_362_ZN_10976
3ex8_C_ZN_362_ZN_10977

3ex8_D_ZN_362_ZN_11001
3g8q_A_ZN_301_ZN_8592
3g8q_B_ZN_301_ZN_8594
3g8q_C_ZN_301_ZN_8596
3g8q_D_ZN_301_ZN_8599
3ijf_X_ZN_147_ZN_895
3iqs_A_ZN_400_ZN_1525
3ir2_A_ZN_1001_ZN_2989
3ir2_B_ZN_1001_ZN_2993
3mpz_A_ZN_300_ZN_3597
3mpz_B_ZN_300_ZN_3598
3mpz_C_ZN_300_ZN_3599
3mpz_D_ZN_300_ZN_3600
3ocq_A_ZN_184_ZN_1143
3oj6_A_ZN_150_ZN_3884
3oj6_B_ZN_150_ZN_3889
3oj6_C_ZN_150_ZN_3894
3oj6_D_ZN_150_ZN_3900

------- SF_124 --------
1a71_A_ZN_402_ZN_5576
1a71_B_ZN_402_ZN_5628
1a72_A_ZN_377_ZN_2789
1adb_A_ZN_376_ZN_6787
1adb_B_ZN_376_ZN_6846
1adc_A_ZN_376_ZN_6787
1adc_B_ZN_376_ZN_6846
1adf_A_ZN_377_ZN_3395
1adg_A_ZN_377_ZN_3395
1agn_A_ZN_375_ZN_11161
1agn_B_ZN_375_ZN_11220
1agn_C_ZN_375_ZN_11285
1agn_D_ZN_375_ZN_11336
1axe_A_ZN_402_ZN_5580
1axe_B_ZN_402_ZN_5632
1axg_A_ZN_402_ZN_11138
1axg_B_ZN_402_ZN_11190
1axg_C_ZN_402_ZN_11242
1axg_D_ZN_402_ZN_11294
1bto_A_ZN_376_ZN_11146
1bto_B_ZN_376_ZN_11202
1bto_C_ZN_376_ZN_11258
1bto_D_ZN_376_ZN_11314
1cdo_A_ZN_377_ZN_5610
1cdo_B_ZN_377_ZN_5656
1d1s_A_ZN_375_ZN_11161
1d1s_B_ZN_375_ZN_11232
1d1s_C_ZN_375_ZN_11309
1d1s_D_ZN_375_ZN_11364
1d1t_A_ZN_375_ZN_11161
1d1t_B_ZN_375_ZN_11232
1d1t_C_ZN_375_ZN_11309
1d1t_D_ZN_375_ZN_11364
1deh_A_ZN_375_ZN_5565
1deh_B_ZN_375_ZN_5617
1e3e_A_ZN_379_ZN_5626
1e3e_B_ZN_379_ZN_5672
1e3i_A_ZN_381_ZN_5637

1e3i_B_ZN_381_ZN_5692
1e3j_A_ZN_901_ZN_2588
1e3l_A_ZN_381_ZN_5634
1e3l_B_ZN_381_ZN_5680
1ee2_A_ZN_1301_ZN_5783
1ee2_B_ZN_1303_ZN_5858
1f8f_A_ZN_373_ZN_2675
1h2b_A_ZN_1364_ZN_5390
1h2b_B_ZN_1363_ZN_5446
1hdx_A_ZN_375_ZN_5565
1hdx_B_ZN_375_ZN_5618
1hdy_A_ZN_375_ZN_5563
1hdy_B_ZN_375_ZN_5615
1hdz_A_ZN_375_ZN_5551
1hdz_B_ZN_375_ZN_5597
1het_A_ZN_401_ZN_6110
1het_B_ZN_401_ZN_6182
1hld_A_ZN_376_ZN_5574
1hld_B_ZN_776_ZN_5642
1hso_A_ZN_1375_ZN_5553
1hso_B_ZN_2375_ZN_5605
1hsz_A_ZN_1375_ZN_5565
1hsz_B_ZN_2375_ZN_5611
1ht0_A_ZN_1375_ZN_5559
1ht0_B_ZN_2375_ZN_5605
1htb_A_ZN_375_ZN_5555
1htb_B_ZN_375_ZN_5607
1ju9_A_ZN_376_ZN_5570
1ju9_B_ZN_376_ZN_5599
1jvb_A_ZN_400_ZN_2623
1kol_A_ZN_1002_ZN_5872
1kol_B_ZN_1004_ZN_5923
1lde_A_ZN_376_ZN_11146
1lde_B_ZN_376_ZN_11200
1lde_C_ZN_376_ZN_11254
1lde_D_ZN_376_ZN_11308
1ldy_A_ZN_376_ZN_11146
1ldy_B_ZN_376_ZN_11201
1ldy_C_ZN_376_ZN_11256
1ldy_D_ZN_376_ZN_11311
1llu_A_ZN_344_ZN_20130
1llu_B_ZN_344_ZN_20184
1llu_C_ZN_344_ZN_20234
1llu_D_ZN_344_ZN_20288
1llu_E_ZN_344_ZN_20338
1llu_F_ZN_344_ZN_20392
1llu_G_ZN_344_ZN_20442
1llu_H_ZN_344_ZN_20496
1m6h_A_ZN_1375_ZN_5547
1m6h_B_ZN_1375_ZN_5565
1m6w_A_ZN_1375_ZN_5547
1m6w_B_ZN_1375_ZN_5580
1ma0_A_ZN_5375_ZN_5547
1ma0_B_ZN_6375_ZN_5618
1mc5_A_ZN_375_ZN_5547
1mc5_B_ZN_375_ZN_5626
1mg0_A_ZN_376_ZN_11142
1mg0_B_ZN_376_ZN_11207

1mg0_C_ZN_376_ZN_11272
1mg0_D_ZN_376_ZN_11337
1mgo_A_ZN_376_ZN_5564
1mgo_B_ZN_376_ZN_5610
1mp0_A_ZN_375_ZN_5547
1mp0_B_ZN_375_ZN_5604
1n8k_A_ZN_376_ZN_5609
1n8k_B_ZN_376_ZN_5668
1n92_A_ZN_376_ZN_5574
1n92_B_ZN_376_ZN_5634
1nto_A_ZN_400_ZN_15716
1nto_B_ZN_400_ZN_15718
1nto_C_ZN_400_ZN_15720
1nto_D_ZN_400_ZN_15722
1nto_E_ZN_400_ZN_15724
1nto_H_ZN_400_ZN_15726
1nvg_A_ZN_400_ZN_2622
1p0c_A_ZN_1501_ZN_5459
1p0c_B_ZN_2501_ZN_5472
1p0f_A_ZN_2502_ZN_5458
1p0f_B_ZN_2501_ZN_5514
1p1r_A_ZN_376_ZN_11146
1p1r_B_ZN_376_ZN_11202
1p1r_C_ZN_376_ZN_11266
1p1r_D_ZN_376_ZN_11322
1piw_A_ZN_1501_ZN_5563
1piw_B_ZN_1501_ZN_5565
1ps0_A_ZN_1501_ZN_2782
1q1n_A_ZN_1501_ZN_2782
1qlh_A_ZN_376_ZN_2788
1qlj_A_ZN_376_ZN_2788
1qv6_A_ZN_376_ZN_5576
1qv6_B_ZN_376_ZN_5640
1qv7_A_ZN_376_ZN_5576
1qv7_B_ZN_376_ZN_5632
1r37_A_ZN_400_ZN_5280
1r37_B_ZN_400_ZN_5332
1rjw_A_ZN_401_ZN_10221
1rjw_B_ZN_401_ZN_10235
1rjw_C_ZN_401_ZN_10261
1rjw_D_ZN_401_ZN_10281
1teh_A_ZN_375_ZN_5547
1teh_B_ZN_375_ZN_5593
1u3t_A_ZN_375_ZN_5553
1u3t_B_ZN_375_ZN_5611
1u3u_A_ZN_375_ZN_5565
1u3u_B_ZN_375_ZN_5621
1u3v_A_ZN_375_ZN_5565
1u3v_B_ZN_375_ZN_5621
1u3w_A_ZN_375_ZN_5559
1u3w_B_ZN_375_ZN_5616
1uuf_A_ZN_401_ZN_2585
1vj0_A_ZN_400_ZN_11307
1vj0_B_ZN_400_ZN_11312
1vj0_C_ZN_400_ZN_11316
1vj0_D_ZN_400_ZN_11322
1ye3_A_ZN_376_ZN_2788
1yqd_A_ZN_2000_ZN_5438

1yqd_B_ZN_3000_ZN_5495
1yqx_A_ZN_2000_ZN_5438
1yqx_B_ZN_3000_ZN_5487
2cd9_A_ZN_1368_ZN_5712
2cd9_B_ZN_1368_ZN_5715
2cda_A_ZN_1369_ZN_5709
2cda_B_ZN_1369_ZN_5759
2cdb_A_ZN_1371_ZN_11657
2cdb_B_ZN_1371_ZN_11723
2cdb_C_ZN_1371_ZN_11789
2cdb_D_ZN_1371_ZN_11856
2cdc_A_ZN_1372_ZN_11841
2cdc_B_ZN_1372_ZN_11915
2cdc_C_ZN_1372_ZN_11989
2cdc_D_ZN_1373_ZN_12073
2cf5_A_ZN_401_ZN_2667
2cf6_A_ZN_401_ZN_2667
2dfv_A_ZN_1001_ZN_7966
2dfv_B_ZN_1002_ZN_8025
2dfv_C_ZN_1003_ZN_8084
2dph_A_ZN_1001_ZN_6019
2dph_B_ZN_1003_ZN_6065
2dq4_A_ZN_502_ZN_5142
2dq4_B_ZN_504_ZN_5156
2eer_A_ZN_501_ZN_5273
2eer_B_ZN_502_ZN_5318
2eih_A_ZN_500_ZN_5135
2eih_B_ZN_501_ZN_5136
2ejv_A_ZN_502_ZN_5133
2ejv_B_ZN_502_ZN_5179
2fze_A_ZN_375_ZN_5557
2fze_B_ZN_375_ZN_5607
2fzw_A_ZN_375_ZN_5664
2fzw_B_ZN_375_ZN_5716
2h6e_A_ZN_500_ZN_2486
2hcy_A_ZN_349_ZN_10334
2hcy_B_ZN_349_ZN_10387
2hcy_C_ZN_349_ZN_10395
2hcy_D_ZN_349_ZN_10448
2jhg_A_ZN_402_ZN_6149
2jhg_B_ZN_402_ZN_6217
2ohx_A_ZN_402_ZN_5574
2ohx_B_ZN_402_ZN_5624
2oxi_A_ZN_376_ZN_5584
2oxi_B_ZN_376_ZN_5634
2xaa_A_ZN_1347_ZN_9784
2xaa_B_ZN_1348_ZN_9836
2xaa_C_ZN_1347_ZN_9882
2xaa_D_ZN_1348_ZN_9940
3bto_A_ZN_376_ZN_11146
3bto_B_ZN_376_ZN_11202
3bto_C_ZN_376_ZN_11258
3bto_D_ZN_376_ZN_11314
3cos_A_ZN_502_ZN_11233
3cos_B_ZN_502_ZN_11291
3cos_C_ZN_502_ZN_11353
3cos_D_ZN_502_ZN_11407
3hud_A_ZN_375_ZN_5565

3hud_B_ZN_375_ZN_5567
3i4c_A_ZN_400_ZN_14711
3i4c_B_ZN_400_ZN_14713
3i4c_C_ZN_400_ZN_14715
3i4c_D_ZN_400_ZN_14717
3i4c_E_ZN_400_ZN_14719
3i4c_H_ZN_400_ZN_14721
3jv7_A_ZN_501_ZN_9921
3jv7_B_ZN_501_ZN_9979
3jv7_C_ZN_501_ZN_10037
3jv7_D_ZN_501_ZN_10095
3m6i_A_ZN_402_ZN_5420
3m6i_B_ZN_402_ZN_5466
3meq_A_ZN_501_ZN_10057
3meq_B_ZN_501_ZN_10116
3meq_C_ZN_501_ZN_10150
3meq_D_ZN_501_ZN_10162
3oq6_A_ZN_376_ZN_5736
3oq6_B_ZN_376_ZN_5811
5adh_A_ZN_376_ZN_2788
6adh_A_ZN_376_ZN_5573
6adh_B_ZN_376_ZN_5575
7adh_A_ZN_376_ZN_2787
8adh_A_ZN_377_ZN_2788

------- SF_125 --------
1ml9_A_ZN_1_ZN_1925,1ml9_A_ZN_2_ZN_1926,1ml9_A_ZN_3_ZN_1927
1mvh_A_ZN_501_ZN_2143,1mvh_A_ZN_502_ZN_2144,1mvh_A_ZN_503_ZN_2145
1mvx_A_ZN_501_ZN_2143,1mvx_A_ZN_502_ZN_2144,1mvx_A_ZN_503_ZN_2145
1peg_A_ZN_1_ZN_3563,1peg_A_ZN_2_ZN_3564,1peg_A_ZN_3_ZN_3565
2igq_A_ZN_501_ZN_4085,2igq_A_ZN_502_ZN_4086,2igq_A_ZN_503_ZN_4087
2igq_B_ZN_501_ZN_4115,2igq_B_ZN_502_ZN_4116,2igq_B_ZN_503_ZN_4117
2o8j_A_ZN_1501_ZN_8332,2o8j_A_ZN_1502_ZN_8333,2o8j_A_ZN_1503_ZN_8334
2o8j_B_ZN_1505_ZN_8362,2o8j_B_ZN_1506_ZN_8363,2o8j_B_ZN_1507_ZN_8364
2o8j_C_ZN_1509_ZN_8392,2o8j_C_ZN_1510_ZN_8393,2o8j_C_ZN_1511_ZN_8394
2o8j_D_ZN_1513_ZN_8422,2o8j_D_ZN_1514_ZN_8423,2o8j_D_ZN_1515_ZN_8424
2r3a_A_ZN_301_ZN_2156,2r3a_A_ZN_302_ZN_2157,2r3a_A_ZN_303_ZN_2158
2rfi_A_ZN_501_ZN_4327,2rfi_A_ZN_502_ZN_4328,2rfi_A_ZN_503_ZN_4329
2rfi_B_ZN_501_ZN_4331,2rfi_B_ZN_502_ZN_4332,2rfi_B_ZN_503_ZN_4333
2w5y_A_ZN_4970_ZN_1365
2w5z_A_ZN_4970_ZN_1528
3bo5_A_ZN_301_ZN_2123,3bo5_A_ZN_302_ZN_2124,3bo5_A_ZN_303_ZN_2125
3fpd_A_ZN_1236_ZN_4239,3fpd_A_ZN_2_ZN_4240,3fpd_A_ZN_3_ZN_4241
3fpd_B_ZN_5_ZN_4305,3fpd_B_ZN_6_ZN_4306,3fpd_B_ZN_7_ZN_4307
3h6l_A_ZN_1088_ZN_1740,3h6l_A_ZN_1089_ZN_1741
3h6l_A_ZN_1090_ZN_1742
3hna_A_ZN_501_ZN_4580,3hna_A_ZN_502_ZN_4581,3hna_A_ZN_503_ZN_4582
3hna_B_ZN_501_ZN_4610,3hna_B_ZN_502_ZN_4611,3hna_B_ZN_503_ZN_4612
3k5k_A_ZN_1194_ZN_4224,3k5k_A_ZN_1195_ZN_4225,3k5k_A_ZN_1196_ZN_4226
3k5k_B_ZN_1194_ZN_4291,3k5k_B_ZN_1195_ZN_4293,3k5k_B_ZN_1196_ZN_4294
3mo0_A_ZN_1236_ZN_3919,3mo0_A_ZN_2_ZN_3920,3mo0_A_ZN_3_ZN_3921
3mo0_B_ZN_5_ZN_4028,3mo0_B_ZN_6_ZN_4029,3mo0_B_ZN_7_ZN_4030
3mo2_A_ZN_340_ZN_8099,3mo2_A_ZN_341_ZN_8100,3mo2_A_ZN_342_ZN_8101
3mo2_B_ZN_5_ZN_8143,3mo2_B_ZN_6_ZN_8144,3mo2_B_ZN_7_ZN_8145
3mo2_C_ZN_1_ZN_8187,3mo2_C_ZN_2_ZN_8188,3mo2_C_ZN_3_ZN_8189
3mo2_D_ZN_5_ZN_8257,3mo2_D_ZN_6_ZN_8258,3mo2_D_ZN_7_ZN_8259
3mo5_A_ZN_1_ZN_8149,3mo5_A_ZN_2_ZN_8150,3mo5_A_ZN_3_ZN_8151
3mo5_B_ZN_5_ZN_8192,3mo5_B_ZN_6_ZN_8193,3mo5_B_ZN_7_ZN_8194

3mo5_C_ZN_1_ZN_8235,3mo5_C_ZN_2_ZN_8236,3mo5_C_ZN_3_ZN_8237
3mo5_D_ZN_5_ZN_8304,3mo5_D_ZN_6_ZN_8305,3mo5_D_ZN_7_ZN_8306
3nni_A_ZN_1194_ZN_4057,3nni_A_ZN_1195_ZN_4058,3nni_A_ZN_1196_ZN_4059
3nni_B_ZN_1194_ZN_4130,3nni_B_ZN_1195_ZN_4131,3nni_B_ZN_1196_ZN_4132
3ooi_A_ZN_232_ZN_1950
3ooi_A_ZN_233_ZN_1951,3ooi_A_ZN_234_ZN_1952

------- SF_126 --------
1peg_A_ZN_4_ZN_3566
1peg_B_ZN_8_ZN_3570
2igq_A_ZN_504_ZN_4088
2igq_B_ZN_504_ZN_4118
2o8j_A_ZN_1504_ZN_8335
2o8j_B_ZN_1508_ZN_8365
2o8j_C_ZN_1512_ZN_8395
2o8j_D_ZN_1516_ZN_8425
2r3a_A_ZN_300_ZN_2155
2rfi_A_ZN_504_ZN_4330
2rfi_B_ZN_504_ZN_4334
3bo5_A_ZN_304_ZN_2126
3fpd_A_ZN_4_ZN_4242
3fpd_B_ZN_8_ZN_4308
3hna_A_ZN_504_ZN_4583
3hna_B_ZN_504_ZN_4613
3k5k_A_ZN_1197_ZN_4227
3k5k_B_ZN_1197_ZN_4295
3mek_A_ZN_502_ZN_3358
3mo0_A_ZN_4_ZN_3922
3mo0_B_ZN_8_ZN_4031
3mo2_A_ZN_343_ZN_8102
3mo2_B_ZN_8_ZN_8146
3mo2_C_ZN_4_ZN_8190
3mo2_D_ZN_8_ZN_8260
3mo5_A_ZN_4_ZN_8152
3mo5_B_ZN_8_ZN_8195
3mo5_C_ZN_4_ZN_8238
3mo5_D_ZN_8_ZN_8307
3n71_A_ZN_497_ZN_3841
3nni_A_ZN_1197_ZN_4060
3nni_B_ZN_1197_ZN_4133
3pdn_A_ZN_438_ZN_3541

------- SF_127 --------
1a5t_A_ZN_501_ZN_3064
1jr3_A_ZN_400_ZN_13832
1jr3_B_ZN_400_ZN_13838
1jr3_C_ZN_400_ZN_13844
1jr3_E_ZN_400_ZN_13850
1njf_A_ZN_401_ZN_7417
1njf_B_ZN_402_ZN_7449
1njf_C_ZN_403_ZN_7481
1njf_D_ZN_404_ZN_7509
1njg_A_ZN_401_ZN_3718
1njg_B_ZN_402_ZN_3729
1oyw_A_ZN_801_ZN_4066
1oyy_A_ZN_524_ZN_4039
1xxh_B_ZN_403_ZN_27607
1xxh_C_ZN_401_ZN_27605

1xxh_D_ZN_402_ZN_27606
1xxh_E_ZN_404_ZN_27608
1xxh_G_ZN_407_ZN_27611
1xxh_H_ZN_405_ZN_27609
1xxh_I_ZN_406_ZN_27610
1xxh_J_ZN_408_ZN_27612
1xxi_B_ZN_403_ZN_27612
1xxi_C_ZN_401_ZN_27610
1xxi_D_ZN_402_ZN_27611
1xxi_E_ZN_404_ZN_27613
1xxi_G_ZN_407_ZN_27616
1xxi_H_ZN_405_ZN_27614
1xxi_I_ZN_406_ZN_27615
1xxi_J_ZN_408_ZN_27617
2v1x_A_ZN_1595_ZN_8271
2v1x_B_ZN_1595_ZN_8304
2wwy_A_ZN_1595_ZN_9628
2wwy_B_ZN_1595_ZN_9643
3glf_B_ZN_418_ZN_28765
3glf_C_ZN_419_ZN_28766
3glf_D_ZN_420_ZN_28767
3glf_E_ZN_421_ZN_28768
3glf_G_ZN_422_ZN_28769
3glf_H_ZN_423_ZN_28770
3glf_I_ZN_424_ZN_28771
3glf_J_ZN_425_ZN_28772
3glg_B_ZN_418_ZN_28753
3glg_C_ZN_419_ZN_28754
3glg_D_ZN_420_ZN_28755
3glg_E_ZN_421_ZN_28756
3glg_G_ZN_422_ZN_28757
3glg_H_ZN_423_ZN_28758
3glg_I_ZN_424_ZN_28759
3glg_J_ZN_425_ZN_28760
3gli_B_ZN_418_ZN_29148
3gli_C_ZN_419_ZN_29149
3gli_D_ZN_420_ZN_29150
3gli_E_ZN_421_ZN_29151
3gli_G_ZN_422_ZN_29152
3gli_H_ZN_423_ZN_29153
3gli_I_ZN_424_ZN_29154
3gli_J_ZN_425_ZN_29155

------- SF_128 --------
1t9h_A_ZN_411_ZN_2348
1u0l_A_ZN_298_ZN_6667
1u0l_B_ZN_598_ZN_6696
1u0l_C_ZN_898_ZN_6725
2rcn_A_ZN_359_ZN_2177
2yv5_A_ZN_303_ZN_2369

------- SF_129 --------
2d9m_A_ZN_1085_ZN_1019
2d9n_A_ZN_256_ZN_1165
2d9n_A_ZN_456_ZN_1166
2e5s_A_ZN_201_ZN_1471
2e5s_A_ZN_401_ZN_1472
2rhk_C_ZN_501_ZN_2933

2rhk_C_ZN_502_ZN_2932
2rhk_D_ZN_501_ZN_2939
2rhk_D_ZN_502_ZN_2938
2rpp_A_ZN_201_ZN_1343
2rpp_A_ZN_401_ZN_1344
3d2n_A_ZN_101_ZN_612
3d2n_A_ZN_102_ZN_613
3d2q_A_ZN_303_ZN_2171
3d2q_A_ZN_304_ZN_2172
3d2q_B_ZN_303_ZN_2173
3d2q_B_ZN_304_ZN_2174
3d2q_C_ZN_303_ZN_2175
3d2q_C_ZN_304_ZN_2176
3d2q_D_ZN_303_ZN_2177
3d2q_D_ZN_304_ZN_2178
3d2s_A_ZN_303_ZN_2553
3d2s_A_ZN_304_ZN_2554
3d2s_B_ZN_303_ZN_2555
3d2s_B_ZN_304_ZN_2556
3d2s_C_ZN_303_ZN_2557
3d2s_C_ZN_304_ZN_2558
3d2s_D_ZN_303_ZN_2559
3d2s_D_ZN_304_ZN_2560

------- SF_130 --------
1m9o_A_ZN_78_ZN_621
1rgo_A_ZN_221_ZN_1128
1rgo_A_ZN_222_ZN_1129
2cqe_A_ZN_622_ZN_1437
2cqe_A_ZN_822_ZN_1438
2fc6_A_ZN_201_ZN_661

------- SF_131 --------
1efz_A_ZN_500_ZN_2902
1enu_A_ZN_500_ZN_2904
1f3e_A_ZN_500_ZN_2904
1iq8_A_ZN_600_ZN_9307
1iq8_B_ZN_600_ZN_9309
1it7_A_ZN_600_ZN_9307
1it7_B_ZN_600_ZN_9320
1it8_A_ZN_600_ZN_9307
1it8_B_ZN_600_ZN_9322
1j2b_A_ZN_600_ZN_12454
1j2b_B_ZN_600_ZN_12457
1k4g_A_ZN_400_ZN_2904
1k4h_A_ZN_400_ZN_2904
1n2v_A_ZN_400_ZN_2904
1ozm_A_ZN_400_ZN_2903
1ozq_A_ZN_400_ZN_2909
1p0b_A_ZN_400_ZN_2932
1p0d_A_ZN_400_ZN_2832
1p0e_A_ZN_400_ZN_2830
1pud_A_ZN_400_ZN_2904
1pxg_A_ZN_400_ZN_2847
1q2r_A_ZN_401_ZN_12505
1q2r_B_ZN_402_ZN_12517
1q2r_C_ZN_403_ZN_12529
1q2r_D_ZN_404_ZN_12541

1q2s_A_ZN_401_ZN_12518
1q2s_B_ZN_402_ZN_12519
1q2s_C_ZN_403_ZN_12520
1q2s_D_ZN_404_ZN_12532
1q4w_A_ZN_400_ZN_2852
1q63_A_ZN_400_ZN_2864
1q65_A_ZN_400_ZN_2884
1q66_A_ZN_400_ZN_2847
1r5y_A_ZN_400_ZN_2969
1s38_A_ZN_400_ZN_2867
1s39_A_ZN_400_ZN_2870
1wkd_A_ZN_400_ZN_2901
1wke_A_ZN_400_ZN_2901
1wkf_A_ZN_400_ZN_2908
1y5v_A_ZN_400_ZN_2882
1y5w_A_ZN_400_ZN_2834
1y5x_A_ZN_400_ZN_5571
1y5x_D_ZN_1400_ZN_5597
2ash_A_ZN_400_ZN_11153
2ash_B_ZN_400_ZN_11170
2ash_C_ZN_400_ZN_11175
2ash_D_ZN_400_ZN_11194
2bbf_A_ZN_400_ZN_2872
2nqz_A_ZN_400_ZN_2780
2nso_A_ZN_500_ZN_2785
2oko_A_ZN_500_ZN_2916
2pot_A_ZN_500_ZN_2698
2pwu_A_ZN_500_ZN_2738
2pwv_A_ZN_400_ZN_2811
2qii_A_ZN_400_ZN_2921
2qzr_A_ZN_400_ZN_2798
2z1v_A_ZN_400_ZN_2912
2z1w_A_ZN_500_ZN_2885
2z1x_A_ZN_500_ZN_2847
2z7k_A_ZN_390_ZN_2897
3bl3_A_ZN_500_ZN_2757
3bld_A_ZN_600_ZN_2702
3bll_A_ZN_600_ZN_2699
3blo_A_ZN_500_ZN_2684
3c2n_A_ZN_390_ZN_2710
3c2y_A_ZN_390_ZN_2810
3c2z_A_ZN_1402_ZN_2761
3eos_A_ZN_387_ZN_2746
3eou_A_ZN_390_ZN_2694
3gc4_A_ZN_399_ZN_2776
3gc5_A_ZN_390_ZN_2893
3ge7_A_ZN_387_ZN_2818
3gev_A_ZN_390_ZN_2725
3gfn_A_ZN_399_ZN_2730
3hfy_A_ZN_400_ZN_2618

------- SF_132 --------
1r1v_A_ZN_501_ZN_1519
1r22_A_ZN_502_ZN_1508
1r22_B_ZN_501_ZN_1509
1r23_B_ZN_501_ZN_1589
1u2w_A_ZN_501_ZN_3156
1u2w_B_ZN_502_ZN_3157

1u2w_C_ZN_503_ZN_3158

------- SF_133 --------
1f81_A_ZN_88_ZN_1374
1f81_A_ZN_89_ZN_1375
1f81_A_ZN_90_ZN_1376
1l3e_B_ZN_202_ZN_2207
1l3e_B_ZN_203_ZN_2208
1l3e_B_ZN_204_ZN_2209
1l8c_A_ZN_96_ZN_2281
1l8c_A_ZN_97_ZN_2282
1l8c_A_ZN_98_ZN_2283
1p4q_B_ZN_301_ZN_2371
1p4q_B_ZN_302_ZN_2372
1p4q_B_ZN_303_ZN_2373
1r8u_B_ZN_440_ZN_2382
1r8u_B_ZN_441_ZN_2383
1r8u_B_ZN_442_ZN_2384
1u2n_A_ZN_440_ZN_1568
1u2n_A_ZN_441_ZN_1569
1u2n_A_ZN_442_ZN_1570
2ka4_A_ZN_440_ZN_2447
2ka4_A_ZN_441_ZN_2448
2ka4_A_ZN_442_ZN_2449
2ka6_A_ZN_93_ZN_2146
2ka6_A_ZN_94_ZN_2147
2ka6_A_ZN_95_ZN_2148
2kje_A_ZN_500_ZN_2072
2kje_A_ZN_501_ZN_2073
2kje_A_ZN_502_ZN_2074
3io2_A_ZN_201_ZN_861
3io2_A_ZN_202_ZN_862
3io2_A_ZN_203_ZN_863

------- SF_134 --------
1ia9_A_ZN_2001_ZN_4494
1ia9_B_ZN_2001_ZN_4526
1iah_A_ZN_2001_ZN_4494
1iah_B_ZN_2001_ZN_4522
1iaj_A_ZN_2001_ZN_4135
1iaj_B_ZN_2001_ZN_4136
3lkm_A_ZN_904_ZN_1969
3lla_A_ZN_2002_ZN_4037
3lla_B_ZN_2001_ZN_4074
3lmh_A_ZN_1_ZN_3923
3lmh_B_ZN_2_ZN_3956
3lmi_A_ZN_1001_ZN_7966
3lmi_B_ZN_1002_ZN_8009
3lmi_C_ZN_1003_ZN_8047
3lmi_D_ZN_1004_ZN_8090
3pdt_A_ZN_1_ZN_1999

------- SF_135 --------
1q08_A_ZN_401_ZN_1461,1q08_A_ZN_402_ZN_1462
1q08_B_ZN_403_ZN_1469,1q08_B_ZN_404_ZN_1470
1q09_A_ZN_401_ZN_737,1q09_A_ZN_402_ZN_738
1q0a_A_ZN_401_ZN_1449,1q0a_A_ZN_402_ZN_1450
1q0a_B_ZN_403_ZN_1457,1q0a_B_ZN_404_ZN_1458

------- SF_136 --------
1i3q_C_ZN_2002_ZN_28164
1i50_C_ZN_3002_ZN_28294
1i6h_C_ZN_319_ZN_28435
1k83_C_ZN_3002_ZN_27840
1nik_C_ZN_319_ZN_28304
1r5u_C_ZN_319_ZN_28305
1r9s_C_ZN_319_ZN_28468
1r9t_C_ZN_319_ZN_29224
1sfo_C_ZN_319_ZN_28654
1twa_C_ZN_3002_ZN_27730
1twc_C_ZN_3002_ZN_27754
1twf_C_ZN_3002_ZN_28291
1twg_C_ZN_3002_ZN_27732
1twh_C_ZN_3002_ZN_27701
1wcm_C_ZN_1269_ZN_30953
1y1v_C_ZN_319_ZN_31811
1y1w_C_ZN_319_ZN_31812
1y77_C_ZN_319_ZN_31813
2b63_C_ZN_1269_ZN_31740
2b8k_C_ZN_319_ZN_31048
2e2h_C_ZN_319_ZN_28978
2e2i_C_ZN_319_ZN_29668
2e2j_C_ZN_319_ZN_29181
2ja5_A_ZN_2462_ZN_31671
2ja6_A_ZN_2462_ZN_32023
2ja7_A_ZN_2467_ZN_63947
2ja7_A_ZN_2468_ZN_63948
2ja8_A_ZN_2462_ZN_32012
2nvq_C_ZN_319_ZN_29375
2nvt_C_ZN_319_ZN_29143
2nvx_C_ZN_319_ZN_29388
2nvy_C_ZN_3002_ZN_28291
2nvz_C_ZN_319_ZN_28980
2r7z_C_ZN_319_ZN_31814
2r92_C_ZN_302_ZN_31620
2r93_C_ZN_302_ZN_31508
2vum_C_ZN_1269_ZN_32095
2yu9_C_ZN_319_ZN_29479
3cqz_C_ZN_3002_ZN_27330
3fki_C_ZN_319_ZN_31419
3gtg_C_ZN_319_ZN_30074
3gtj_C_ZN_319_ZN_29982
3gtk_C_ZN_319_ZN_30120
3gtl_C_ZN_319_ZN_29264
3gtm_C_ZN_319_ZN_30569
3gto_C_ZN_319_ZN_29267
3gtp_C_ZN_319_ZN_29287
3gtq_C_ZN_319_ZN_28633
3h3v_D_ZN_319_ZN_31787
3hou_C_ZN_9987_ZN_63682
3hou_O_ZN_9995_ZN_63690
3hov_C_ZN_1269_ZN_31788
3how_C_ZN_1269_ZN_31887
3hox_C_ZN_1269_ZN_31929
3hoy_C_ZN_1269_ZN_31814
3hoz_C_ZN_1269_ZN_31972

3i4m_C_ZN_1269_ZN_32366
3i4n_C_ZN_1269_ZN_32318
3k1f_C_ZN_3002_ZN_32340
3k7a_C_ZN_319_ZN_29035

------- SF_137 --------
1xto_A_ZN_312_ZN_2346
3g1p_A_ZN_300_ZN_3886
3g1p_B_ZN_301_ZN_3898
3jxp_A_ZN_320_ZN_2362
3p2u_A_ZN_262_ZN_4053
3p2u_B_ZN_262_ZN_4061

------- SF_138 --------
1smy_D_ZN_9103_ZN_53709
1smy_N_ZN_9105_ZN_53928
1ynj_D_ZN_1526_ZN_24375
1ynn_D_ZN_1526_ZN_24315
2a68_D_ZN_7112_ZN_53766
2a68_N_ZN_7113_ZN_54122
2a69_D_ZN_7112_ZN_53760
2a69_N_ZN_7113_ZN_54065
2a6e_D_ZN_7112_ZN_53570
2a6e_N_ZN_7113_ZN_53573
2a6h_D_ZN_7412_ZN_53475
2a6h_N_ZN_7413_ZN_53521
2be5_D_ZN_9112_ZN_53571
2be5_N_ZN_9113_ZN_53600
2cw0_D_ZN_1526_ZN_53970
2cw0_N_ZN_1526_ZN_53972
2o5i_D_ZN_6112_ZN_49168
2o5i_N_ZN_7112_ZN_49171
2o5j_D_ZN_6112_ZN_48528
2o5j_N_ZN_7112_ZN_48563
2ppb_D_ZN_8112_ZN_49397
2ppb_N_ZN_8212_ZN_49506
3aoh_D_ZN_2001_ZN_74265
3aoh_I_ZN_2002_ZN_74266
3aoh_N_ZN_2003_ZN_74267
3aoi_D_ZN_2002_ZN_73666
3aoi_I_ZN_2003_ZN_73667
3aoi_N_ZN_2001_ZN_73665
3dxj_D_ZN_1526_ZN_56084
3dxj_N_ZN_1526_ZN_56100
3eql_D_ZN_1526_ZN_52792
3eql_N_ZN_1526_ZN_52825
3lu0_D_ZN_1410_ZN_25415

------- SF_139 --------
2glz_A_ZN_200_ZN_2445
2glz_B_ZN_200_ZN_2471
2gvi_A_ZN_300_ZN_1601

------- SF_140 --------
1a73_A_ZN_201_ZN_3354
1a73_B_ZN_203_ZN_3356
1a74_A_ZN_601_ZN_3349
1a74_B_ZN_603_ZN_3351

1cyq_A_ZN_601_ZN_3341
1cyq_B_ZN_603_ZN_3343
1cz0_A_ZN_601_ZN_3351
1cz0_B_ZN_603_ZN_3353
1evw_A_ZN_501_ZN_6613
1evw_B_ZN_503_ZN_6615
1evw_C_ZN_505_ZN_6617
1evw_D_ZN_507_ZN_6619
1evx_A_ZN_1001_ZN_2493
1evx_B_ZN_1003_ZN_2500
2o6m_A_ZN_602_ZN_3348
2o6m_B_ZN_603_ZN_3349

------- SF_141 --------
1a73_A_ZN_202_ZN_3355
1a73_B_ZN_204_ZN_3357
1a74_A_ZN_602_ZN_3350
1a74_B_ZN_604_ZN_3352
1cyq_A_ZN_602_ZN_3342
1cyq_B_ZN_604_ZN_3344
1cz0_A_ZN_602_ZN_3352
1cz0_B_ZN_604_ZN_3354
1evw_A_ZN_502_ZN_6614
1evw_B_ZN_504_ZN_6616
1evw_C_ZN_506_ZN_6618
1evw_D_ZN_508_ZN_6620
1evx_A_ZN_1002_ZN_2494
1evx_B_ZN_1004_ZN_2501
2o6m_A_ZN_601_ZN_3347
2o6m_B_ZN_604_ZN_3350

------- SF_142 --------
1kwg_A_ZN_806_ZN_5173
1kwk_A_ZN_806_ZN_5185

------- SF_143 --------
1lpv_A_ZN_53_ZN_862,1lpv_A_ZN_53_ZN_863

------- SF_144 --------
1mwz_A_ZN_75_ZN_1102

------- SF_145 --------
1oqj_A_ZN_183_ZN_1411
1oqj_B_ZN_183_ZN_1412

------- SF_146 --------
1rni_A_ZN_256_ZN_1708
1ro0_A_ZN_301_ZN_1705
1ro2_A_ZN_301_ZN_1705
3m1m_A_ZN_1_ZN_2618

------- SF_147 --------
1n25_A_ZN_700_ZN_5869
1n25_B_ZN_700_ZN_5870
1svl_A_ZN_700_ZN_8806
1svl_B_ZN_700_ZN_8835
1svl_C_ZN_700_ZN_8864
1svm_A_ZN_700_ZN_17611

1svm_B_ZN_700_ZN_17644
1svm_C_ZN_700_ZN_17677
1svm_D_ZN_700_ZN_17710
1svm_E_ZN_700_ZN_17743
1svm_F_ZN_700_ZN_17776
1svo_A_ZN_700_ZN_5869
1svo_B_ZN_700_ZN_5870
2h1l_A_ZN_1_ZN_53921
2h1l_B_ZN_2_ZN_53922
2h1l_C_ZN_6_ZN_53926
2h1l_E_ZN_4_ZN_53924
2h1l_F_ZN_5_ZN_53925
2h1l_G_ZN_7_ZN_53927
2h1l_H_ZN_9_ZN_53929
2h1l_I_ZN_8_ZN_53928
2h1l_J_ZN_10_ZN_53930
2h1l_K_ZN_11_ZN_53931
2h1l_L_ZN_12_ZN_53932

------- SF_148 --------
1t3k_A_ZN_201_ZN_2060

------- SF_149 --------
1cw0_A_ZN_201_ZN_1754
1odg_A_ZN_200_ZN_1594
1vsr_A_ZN_201_ZN_1099

------- SF_150 --------
1z3i_X_ZN_900_ZN_5054

------- SF_151 --------
2a5h_A_ZN_421_ZN_13155
2a5h_B_ZN_421_ZN_13224
2a5h_C_ZN_421_ZN_13298
2a5h_D_ZN_421_ZN_13367

------- SF_152 --------
2b5l_C_ZN_3001_ZN_20400
2b5l_D_ZN_3003_ZN_20402
2hye_B_ZN_3001_ZN_16943

------- SF_153 --------
2bjr_A_ZN_1369_ZN_5639
2bjr_B_ZN_1369_ZN_5650

------- SF_154 --------
2f44_A_ZN_255_ZN_1597
2f44_B_ZN_256_ZN_1599
2f44_C_ZN_257_ZN_1601

------- SF_155 --------
2fea_A_ZN_1302_ZN_3656
2fea_B_ZN_2302_ZN_3674

------- SF_156 --------
2fyg_A_ZN_302_ZN_963
2g9t_A_ZN_998_ZN_20940
2g9t_B_ZN_998_ZN_20942

2g9t_C_ZN_998_ZN_20944
2g9t_D_ZN_998_ZN_20946
2g9t_E_ZN_998_ZN_20948
2g9t_F_ZN_998_ZN_20950
2g9t_G_ZN_998_ZN_20952
2g9t_H_ZN_998_ZN_20954
2g9t_I_ZN_998_ZN_20956
2g9t_J_ZN_998_ZN_20958
2g9t_K_ZN_998_ZN_20960
2g9t_L_ZN_998_ZN_20962
2g9t_M_ZN_998_ZN_20964
2g9t_N_ZN_998_ZN_20966
2g9t_O_ZN_998_ZN_20968
2g9t_P_ZN_998_ZN_20970
2g9t_Q_ZN_998_ZN_20972
2g9t_R_ZN_998_ZN_20974
2g9t_S_ZN_998_ZN_20976
2g9t_T_ZN_998_ZN_20978
2g9t_U_ZN_998_ZN_20980
2g9t_V_ZN_998_ZN_20982
2g9t_W_ZN_998_ZN_20984
2g9t_X_ZN_998_ZN_20986
2ga6_A_ZN_998_ZN_20991
2ga6_B_ZN_998_ZN_20993
2ga6_C_ZN_998_ZN_20995
2ga6_D_ZN_998_ZN_20997
2ga6_E_ZN_998_ZN_20999
2ga6_F_ZN_998_ZN_21001
2ga6_G_ZN_998_ZN_21003
2ga6_H_ZN_998_ZN_21005
2ga6_I_ZN_998_ZN_21007
2ga6_J_ZN_998_ZN_21009
2ga6_K_ZN_998_ZN_21011
2ga6_L_ZN_998_ZN_21013
2ga6_M_ZN_998_ZN_21015
2ga6_N_ZN_998_ZN_21017
2ga6_O_ZN_998_ZN_21019
2ga6_P_ZN_998_ZN_21021
2ga6_Q_ZN_998_ZN_21023
2ga6_R_ZN_998_ZN_21025
2ga6_S_ZN_998_ZN_21027
2ga6_T_ZN_998_ZN_21029
2ga6_U_ZN_998_ZN_21031
2ga6_V_ZN_998_ZN_21033
2ga6_W_ZN_998_ZN_21035
2ga6_X_ZN_998_ZN_21037

------- SF_157 --------
2iwj_A_ZN_1050_ZN_420

------- SF_158 --------
2j2s_A_ZN_2215_ZN_1136
2j2s_A_ZN_2216_ZN_1137
2jyi_A_ZN_2001_ZN_905
2jyi_A_ZN_2002_ZN_906
2kkf_A_ZN_2001_ZN_1660
2kkf_A_ZN_2002_ZN_1661

------- SF_159 --------
2kak_A_ZN_150_ZN_636,2kak_A_ZN_170_ZN_637,2kak_A_ZN_190_ZN_638

------- SF_160 --------
2kr1_A_ZN_65_ZN_993

------- SF_161 --------
2pg3_A_ZN_300_ZN_1668
3bl5_A_ZN_300_ZN_9233
3bl5_B_ZN_300_ZN_9240
3bl5_C_ZN_300_ZN_9247
3bl5_D_ZN_300_ZN_9254
3bl5_E_ZN_300_ZN_9261
3bl5_F_ZN_300_ZN_9268

------- SF_162 --------
2pf4_E_ZN_601_ZN_22803
2pf4_E_ZN_602_ZN_22804
2pf4_F_ZN_701_ZN_22805
2pf4_F_ZN_702_ZN_22806
2pf4_G_ZN_801_ZN_22807
2pf4_G_ZN_802_ZN_22808
2pf4_H_ZN_901_ZN_22809
2pf4_H_ZN_902_ZN_22810
2pkg_C_ZN_175_ZN_10359
2pkg_C_ZN_176_ZN_10360
2pkg_D_ZN_175_ZN_10361
2pkg_D_ZN_176_ZN_10362

------- SF_163 --------
2yre_A_ZN_501_ZN_1428

------- SF_164 --------
2vqj_A_ZN_1416_ZN_3076
2vqm_A_ZN_1412_ZN_2971
2vqw_G_ZN_1410_ZN_2873
3c0y_A_ZN_402_ZN_8434
3c0y_B_ZN_404_ZN_8438
3c0y_C_ZN_406_ZN_8442
3c0z_A_ZN_102_ZN_8405
3c0z_B_ZN_102_ZN_8417
3c0z_C_ZN_102_ZN_8430
3c10_A_ZN_102_ZN_8429
3c10_B_ZN_102_ZN_8455
3c10_C_ZN_102_ZN_8481

------- SF_165 --------
3f07_A_ZN_409_ZN_8384

------- SF_166 --------
3hko_A_ZN_701_ZN_2604

------- SF_167 --------
3l0a_A_ZN_266_ZN_2147

------- SF_168 --------
3m99_A_ZN_475_ZN_5517
3mhh_A_ZN_475_ZN_5735

3mhs_A_ZN_475_ZN_6614

------- SF_169 --------
3m99_A_ZN_476_ZN_5518
3mhh_A_ZN_476_ZN_5736
3mhs_A_ZN_476_ZN_6615

------- SF_170 --------
3mi9_C_ZN_88_ZN_5060
3mia_C_ZN_88_ZN_5045

------- SF_171 --------
3lyr_A_ZN_1_ZN_1683
3mln_A_ZN_501_ZN_5634
3mln_B_ZN_501_ZN_5635
3mlo_A_ZN_501_ZN_4312
3mlo_B_ZN_501_ZN_4313
3mlp_A_ZN_501_ZN_11751
3mlp_B_ZN_501_ZN_11778
3mlp_E_ZN_501_ZN_11792
3mlp_F_ZN_501_ZN_11806

------- SF_172 --------
1lr5_A_ZN_180_ZN_5144
1lr5_B_ZN_180_ZN_5217
1lr5_C_ZN_180_ZN_5290
1lr5_D_ZN_180_ZN_5363
1lrh_A_ZN_180_ZN_5153
1lrh_B_ZN_180_ZN_5240
1lrh_C_ZN_180_ZN_5327
1lrh_D_ZN_180_ZN_5414
1pmi_A_ZN_445_ZN_3433
1qwr_A_ZN_604_ZN_5114
1qwr_B_ZN_605_ZN_5127
1y9q_A_ZN_202_ZN_1414
2bnm_A_ZN_1199_ZN_3132
2bnm_B_ZN_1199_ZN_3168
2bnn_A_ZN_1200_ZN_2973
2bnn_B_ZN_1200_ZN_2982
2bno_A_ZN_1201_ZN_3018
2bno_B_ZN_1201_ZN_3041
2fqp_A_ZN_100_ZN_3001
2fqp_B_ZN_100_ZN_3006
2fqp_C_ZN_100_ZN_3011
2fqp_D_ZN_100_ZN_3032
2gc0_A_ZN_801_ZN_3023
2gc0_B_ZN_800_ZN_3040
2gc1_A_ZN_401_ZN_3046
2gc1_B_ZN_402_ZN_3064
2gc2_A_ZN_401_ZN_3023
2gc2_B_ZN_402_ZN_3042
2gc3_A_ZN_402_ZN_3023
2gc3_B_ZN_401_ZN_3040
2o1q_A_ZN_145_ZN_2234
2o1q_B_ZN_145_ZN_2289
2zkl_A_ZN_400_ZN_2918
3bal_A_ZN_154_ZN_4593
3bal_B_ZN_154_ZN_4594

3bal_C_ZN_154_ZN_4595
3bal_D_ZN_154_ZN_4596
3bb6_C_ZN_128_ZN_3534
3bb6_D_ZN_128_ZN_3535
3cew_A_ZN_201_ZN_3557
3cew_B_ZN_201_ZN_3558
3cew_C_ZN_201_ZN_3559
3cew_D_ZN_201_ZN_3560
3h1m_A_ZN_395_ZN_3005
3h1y_A_ZN_399_ZN_3032
3h50_A_ZN_114_ZN_867
3ht2_A_ZN_150_ZN_2275
3ht2_C_ZN_150_ZN_2276
3ibm_A_ZN_200_ZN_2361
3ibm_B_ZN_200_ZN_2363

------- SF_173 --------
1dyq_A_ZN_234_ZN_1884
1eu3_A_ZN_401_ZN_3419
1eu3_B_ZN_402_ZN_3436
1eu4_A_ZN_400_ZN_1668
1eu4_A_ZN_401_ZN_1669
1ewc_A_ZN_500_ZN_1733
1hxy_D_ZN_600_ZN_4744
1i4g_A_ZN_460_ZN_3692
1i4h_A_ZN_301_ZN_3692
1i4h_B_ZN_302_ZN_3693
1sxt_A_ZN_501_ZN_3642
1sxt_B_ZN_401_ZN_3641
2g9h_D_ZN_501_ZN_4903
2j4x_A_ZN_1211_ZN_1603
3ea6_A_ZN_1001_ZN_1838

------- SF_174 --------
1bh5_A_ZN_201_ZN_5707
1bh5_B_ZN_201_ZN_5734
1bh5_C_ZN_201_ZN_5761
1bh5_D_ZN_201_ZN_5788
1fa5_A_ZN_1200_ZN_2005
1fa5_A_ZN_1201_ZN_2006
1fro_A_ZN_201_ZN_5605
1fro_B_ZN_201_ZN_5633
1fro_C_ZN_201_ZN_5661
1fro_D_ZN_201_ZN_5689
1qin_A_ZN_401_ZN_2803
1qin_B_ZN_301_ZN_2835
1qip_A_ZN_902_ZN_5704
1qip_B_ZN_901_ZN_5742
1qip_C_ZN_904_ZN_5780
1qip_D_ZN_903_ZN_5818
1zsw_A_ZN_315_ZN_2857
2qh0_A_ZN_450_ZN_1037
2rjb_A_ZN_501_ZN_12726
2rjb_B_ZN_502_ZN_12727
2rjb_C_ZN_503_ZN_12728
2rjb_D_ZN_504_ZN_12729
2za0_A_ZN_301_ZN_2892
2za0_A_ZN_401_ZN_2893

3ct8_A_ZN_128_ZN_1099
3l7t_A_ZN_401_ZN_4417
3l7t_B_ZN_400_ZN_4418
3oa4_A_ZN_300_ZN_1081
3oaj_A_ZN_500_ZN_4988
3oaj_B_ZN_500_ZN_4994

------- SF_175 --------
12ca_A_ZN_262_ZN_2029
1a42_A_ZN_262_ZN_2041
1am6_A_ZN_262_ZN_2512
1avn_A_ZN_262_ZN_2040
1azm_A_ZN_261_ZN_2021
1bcd_A_ZN_262_ZN_2081
1bic_A_ZN_262_ZN_2080
1bn1_A_ZN_262_ZN_2493
1bn3_A_ZN_262_ZN_2496
1bn4_A_ZN_262_ZN_2493
1bnm_A_ZN_262_ZN_2489
1bnn_A_ZN_262_ZN_2488
1bnq_A_ZN_262_ZN_2041
1bnt_A_ZN_262_ZN_2041
1bnu_A_ZN_262_ZN_2041
1bnv_A_ZN_262_ZN_2488
1bnw_A_ZN_262_ZN_2488
1bv3_A_ZN_262_ZN_2045
1bzm_A_ZN_261_ZN_2036
1ca2_A_ZN_262_ZN_2041
1ca3_A_ZN_262_ZN_2031
1cai_A_ZN_262_ZN_2077
1caj_A_ZN_262_ZN_2074
1cak_A_ZN_262_ZN_2077
1cal_A_ZN_262_ZN_2073
1cam_A_ZN_262_ZN_2079
1cao_A_ZN_262_ZN_2076
1cay_A_ZN_262_ZN_2081
1caz_A_ZN_262_ZN_2077
1ccs_A_ZN_262_ZN_2032
1cct_A_ZN_262_ZN_2033
1ccu_A_ZN_262_ZN_2034
1cil_A_ZN_262_ZN_2041
1cim_A_ZN_262_ZN_2041
1cin_A_ZN_262_ZN_2041
1cnc_A_ZN_262_ZN_2027
1cng_A_ZN_1_ZN_2046
1cnh_A_ZN_1_ZN_2050
1cni_A_ZN_1_ZN_2046
1cnj_A_ZN_1_ZN_2049
1cnk_A_ZN_1_ZN_2049
1cnw_A_ZN_262_ZN_2041
1cnx_A_ZN_262_ZN_2041
1cny_A_ZN_262_ZN_2041
1cra_A_ZN_262_ZN_2082
1cva_A_ZN_262_ZN_2031
1cvb_A_ZN_262_ZN_2031
1cvc_A_ZN_262_ZN_2029
1cvd_A_ZN_262_ZN_2027
1cve_A_ZN_262_ZN_2029

1cvh_A_ZN_262_ZN_2027
1czm_A_ZN_261_ZN_2036
1dca_A_ZN_262_ZN_2030
1dcb_A_ZN_262_ZN_2030
1dmx_A_ZN_280_ZN_3813
1dmx_B_ZN_280_ZN_3814
1dmy_A_ZN_280_ZN_3813
1dmy_B_ZN_280_ZN_3827
1eou_A_ZN_300_ZN_2056
1f2w_A_ZN_262_ZN_2051
1flj_A_ZN_262_ZN_2537
1fql_A_ZN_262_ZN_2030
1fqm_A_ZN_262_ZN_2027
1fr7_A_ZN_262_ZN_4051
1fr7_B_ZN_263_ZN_4052
1g0e_A_ZN_262_ZN_2056
1g0f_A_ZN_262_ZN_2056
1g1d_A_ZN_262_ZN_2060
1g3z_A_ZN_262_ZN_2056
1g45_A_ZN_262_ZN_2056
1g46_A_ZN_262_ZN_2056
1g48_A_ZN_262_ZN_2056
1g4j_A_ZN_262_ZN_2056
1g4o_A_ZN_262_ZN_2056
1g52_A_ZN_262_ZN_2060
1g53_A_ZN_262_ZN_2060
1g54_A_ZN_262_ZN_2060
1h4n_A_ZN_262_ZN_2054
1h9n_A_ZN_262_ZN_2048
1h9q_A_ZN_262_ZN_2030
1hca_A_ZN_262_ZN_2041
1hcb_A_ZN_261_ZN_2025
1hea_A_ZN_262_ZN_2044
1heb_A_ZN_262_ZN_2032
1hec_A_ZN_262_ZN_2043
1hed_A_ZN_262_ZN_2028
1hug_A_ZN_261_ZN_2010
1huh_A_ZN_261_ZN_2018
1i8z_A_ZN_262_ZN_2060
1i90_A_ZN_262_ZN_2060
1i91_A_ZN_262_ZN_2060
1i9l_A_ZN_262_ZN_2056
1i9m_A_ZN_262_ZN_2056
1i9n_A_ZN_262_ZN_2056
1i9o_A_ZN_262_ZN_2056
1i9p_A_ZN_262_ZN_2056
1i9q_A_ZN_262_ZN_2056
1if4_A_ZN_262_ZN_2060
1if5_A_ZN_262_ZN_2060
1if6_A_ZN_262_ZN_2060
1if7_A_ZN_262_ZN_2060
1if8_A_ZN_262_ZN_2060
1if9_A_ZN_262_ZN_2060
1j9w_A_ZN_261_ZN_3983
1j9w_B_ZN_262_ZN_3988
1jcz_A_ZN_901_ZN_4179
1jcz_B_ZN_902_ZN_4184
1jd0_A_ZN_901_ZN_4170

1jd0_B_ZN_902_ZN_4184
1jv0_A_ZN_261_ZN_3998
1jv0_B_ZN_261_ZN_4006
1keq_A_ZN_280_ZN_3808
1keq_B_ZN_281_ZN_3836
1kop_A_ZN_301_ZN_3533
1kop_B_ZN_302_ZN_3546
1koq_A_ZN_301_ZN_3501
1koq_B_ZN_302_ZN_3502
1kwq_A_ZN_262_ZN_2060
1kwr_A_ZN_262_ZN_2060
1lg5_A_ZN_262_ZN_2051
1lg6_A_ZN_262_ZN_2051
1lgd_A_ZN_262_ZN_2051
1lug_A_ZN_1001_ZN_2122
1lzv_A_ZN_262_ZN_2038
1moo_A_ZN_262_ZN_2050
1mua_A_ZN_262_ZN_2039
1okl_A_ZN_262_ZN_2032
1okm_A_ZN_262_ZN_2052
1okn_A_ZN_262_ZN_2042
1oq5_A_ZN_600_ZN_2046
1ray_A_ZN_262_ZN_2081
1raz_A_ZN_262_ZN_2081
1rj5_A_ZN_601_ZN_4145
1rj5_B_ZN_601_ZN_4190
1rj6_A_ZN_601_ZN_4145
1rj6_B_ZN_601_ZN_4209
1t9n_A_ZN_262_ZN_2060
1tb0_X_ZN_262_ZN_2072
1tbt_X_ZN_262_ZN_2077
1te3_X_ZN_262_ZN_2066
1teq_X_ZN_262_ZN_2066
1teu_X_ZN_262_ZN_2077
1tg3_A_ZN_262_ZN_2057
1tg9_A_ZN_262_ZN_2057
1th9_A_ZN_262_ZN_2057
1thk_A_ZN_262_ZN_2057
1ttm_A_ZN_262_ZN_2060
1uga_A_ZN_262_ZN_2065
1ugb_A_ZN_262_ZN_2058
1ugc_A_ZN_262_ZN_2071
1ugd_A_ZN_262_ZN_2060
1uge_A_ZN_262_ZN_2063
1ugf_A_ZN_262_ZN_2068
1ugg_A_ZN_262_ZN_2060
1urt_A_ZN_280_ZN_1894
1v9e_A_ZN_260_ZN_4111
1v9e_B_ZN_260_ZN_4112
1xeg_A_ZN_262_ZN_2065
1xev_A_ZN_262_ZN_8253
1xev_B_ZN_562_ZN_8254
1xev_C_ZN_862_ZN_8255
1xev_D_ZN_999_ZN_8256
1xpz_A_ZN_262_ZN_2060
1xq0_A_ZN_262_ZN_2060
1y7w_A_ZN_283_ZN_4254
1y7w_B_ZN_284_ZN_4260

1yda_A_ZN_262_ZN_2031
1ydb_A_ZN_262_ZN_2033
1ydc_A_ZN_262_ZN_2033
1ydd_A_ZN_262_ZN_2044
1yo0_A_ZN_261_ZN_2058
1yo1_A_ZN_262_ZN_2064
1yo2_A_ZN_262_ZN_2058
1z93_A_ZN_268_ZN_2129
1z97_A_ZN_268_ZN_2126
1z9y_A_ZN_300_ZN_2022
1ze8_A_ZN_263_ZN_2061
1zfk_A_ZN_1300_ZN_2033
1zfq_A_ZN_600_ZN_2036
1zge_A_ZN_1000_ZN_2035
1zgf_A_ZN_400_ZN_2039
1zh9_A_ZN_600_ZN_2031
1znc_A_ZN_301_ZN_4947
1znc_B_ZN_302_ZN_4948
1zsb_A_ZN_262_ZN_2050
1zsc_A_ZN_262_ZN_2051
2abe_A_ZN_262_ZN_2050
2aw1_A_ZN_262_ZN_2086
2ax2_A_ZN_262_ZN_2060
2ca2_A_ZN_264_ZN_2045
2cba_A_ZN_262_ZN_2081
2cbb_A_ZN_262_ZN_2081
2cbc_A_ZN_262_ZN_2081
2cbd_A_ZN_262_ZN_2081
2eu2_A_ZN_262_ZN_2061
2eu3_A_ZN_262_ZN_2041
2ez7_A_ZN_262_ZN_2062
2f14_A_ZN_1262_ZN_2045
2fmg_A_ZN_262_ZN_2051
2fmz_A_ZN_262_ZN_2051
2fnk_A_ZN_262_ZN_2064
2fnm_A_ZN_262_ZN_2055
2fnn_A_ZN_262_ZN_2055
2foq_A_ZN_262_ZN_2056
2fos_A_ZN_262_ZN_2163
2fou_A_ZN_262_ZN_2125
2fov_A_ZN_262_ZN_2118
2foy_A_ZN_301_ZN_4027
2foy_B_ZN_302_ZN_4103
2fw4_A_ZN_561_ZN_4027
2fw4_B_ZN_562_ZN_4039
2gd8_A_ZN_262_ZN_2080
2geh_A_ZN_262_ZN_2051
2h15_A_ZN_262_ZN_2041
2h4n_A_ZN_262_ZN_2048
2hd6_A_ZN_262_ZN_2072
2hfw_A_ZN_262_ZN_2067
2hfx_A_ZN_261_ZN_2080
2hfy_A_ZN_262_ZN_2069
2hkk_A_ZN_261_ZN_2046
2hl4_A_ZN_262_ZN_2088
2hnc_A_ZN_263_ZN_2051
2hoc_A_ZN_263_ZN_2061
2ili_A_ZN_262_ZN_2067

2it4_A_ZN_561_ZN_4035
2it4_B_ZN_562_ZN_4043
2nmx_A_ZN_301_ZN_4094
2nmx_B_ZN_302_ZN_4111
2nn1_A_ZN_301_ZN_4072
2nn1_B_ZN_302_ZN_4096
2nn7_A_ZN_301_ZN_4054
2nn7_B_ZN_302_ZN_4076
2nng_A_ZN_262_ZN_2084
2nno_A_ZN_262_ZN_2118
2nns_A_ZN_262_ZN_2112
2nnv_A_ZN_262_ZN_2128
2nwo_A_ZN_262_ZN_2060
2nwp_A_ZN_262_ZN_2060
2nwy_A_ZN_262_ZN_2060
2nwz_A_ZN_262_ZN_2060
2nxr_A_ZN_262_ZN_2059
2nxs_A_ZN_262_ZN_2059
2nxt_A_ZN_261_ZN_2074
2o4z_A_ZN_262_ZN_2048
2osf_A_ZN_262_ZN_2065
2osm_A_ZN_262_ZN_2070
2pou_A_ZN_262_ZN_2060
2pov_A_ZN_262_ZN_2051
2pow_A_ZN_262_ZN_2050
2q1b_A_ZN_400_ZN_2107
2q1q_A_ZN_262_ZN_2067
2q38_A_ZN_501_ZN_2057
2qo8_A_ZN_262_ZN_2118
2qoa_A_ZN_262_ZN_2106
2qp6_A_ZN_262_ZN_2109
2vva_X_ZN_1267_ZN_2133
2vvb_X_ZN_1268_ZN_2198
2wd2_A_ZN_1262_ZN_2031
2wd3_A_ZN_1263_ZN_2100
2weg_A_ZN_1262_ZN_2060
2weh_A_ZN_1262_ZN_2060
2wej_A_ZN_1262_ZN_2060
2weo_A_ZN_1262_ZN_2050
2x7s_A_ZN_1265_ZN_2151
2x7t_A_ZN_1263_ZN_2087
2x7u_A_ZN_1261_ZN_2051
2znc_A_ZN_1_ZN_2465
3b4f_A_ZN_262_ZN_2064
3bet_A_ZN_262_ZN_2080
3bl0_A_ZN_262_ZN_2063
3bl1_A_ZN_262_ZN_2044
3c7p_A_ZN_262_ZN_2082
3ca2_A_ZN_264_ZN_2043
3caj_A_ZN_262_ZN_2090
3cyu_A_ZN_262_ZN_2051
3czv_A_ZN_262_ZN_4121
3czv_B_ZN_262_ZN_4135
3d0n_A_ZN_262_ZN_4280
3d0n_B_ZN_262_ZN_4285
3d8w_A_ZN_262_ZN_2070
3d92_A_ZN_262_ZN_2098
3d9z_A_ZN_262_ZN_2066

3da2_A_ZN_301_ZN_4091
3da2_B_ZN_301_ZN_4115
3daz_A_ZN_262_ZN_2106
3dbu_A_ZN_262_ZN_2130
3dc3_A_ZN_262_ZN_2112
3dc9_A_ZN_262_ZN_2084
3dcc_A_ZN_262_ZN_2112
3dcs_A_ZN_262_ZN_2083
3dcw_A_ZN_262_ZN_2124
3dd0_A_ZN_262_ZN_2075
3dd8_A_ZN_262_ZN_2044
3dv7_A_ZN_262_ZN_2057
3dvb_A_ZN_262_ZN_2059
3dvc_A_ZN_262_ZN_2066
3dvd_A_ZN_262_ZN_2060
3efi_A_ZN_262_ZN_2068
3eft_A_ZN_262_ZN_2080
3f4x_A_ZN_262_ZN_2067
3f7b_A_ZN_300_ZN_4005
3f7b_B_ZN_301_ZN_4034
3f7u_A_ZN_260_ZN_7957
3f7u_B_ZN_261_ZN_7983
3f7u_C_ZN_262_ZN_8009
3f7u_D_ZN_263_ZN_8035
3f8e_A_ZN_262_ZN_2061
3ffp_X_ZN_262_ZN_2055
3fw3_A_ZN_300_ZN_3965
3fw3_B_ZN_301_ZN_4001
3gz0_A_ZN_262_ZN_2089
3hfp_A_ZN_262_ZN_2065
3hkn_A_ZN_261_ZN_2051
3hkq_A_ZN_261_ZN_2051
3hkt_A_ZN_261_ZN_2051
3hku_A_ZN_261_ZN_2051
3hlj_A_ZN_272_ZN_2108
3hs4_A_ZN_301_ZN_2088
3iai_A_ZN_262_ZN_7970
3iai_B_ZN_262_ZN_8105
3iai_C_ZN_262_ZN_8229
3iai_D_ZN_262_ZN_8347
3ibi_A_ZN_262_ZN_2051
3ibl_A_ZN_262_ZN_2091
3ibn_A_ZN_262_ZN_2071
3ibu_A_ZN_262_ZN_2085
3ieo_A_ZN_262_ZN_2065
3igp_A_ZN_262_ZN_2082
3iqk_A_ZN_262_ZN_2153
3k2f_A_ZN_262_ZN_2041
3k7k_A_ZN_262_ZN_2089
3kig_A_ZN_500_ZN_2066
3kkx_A_ZN_262_ZN_4071
3ks3_A_ZN_262_ZN_2141
3kwa_A_ZN_262_ZN_2047
3l14_A_ZN_262_ZN_2140
3lxe_A_ZN_261_ZN_4019
3lxe_B_ZN_261_ZN_4042
3m1k_A_ZN_501_ZN_2081
3m3x_A_ZN_262_ZN_2193

3m40_A_ZN_262_ZN_2153
3m5e_A_ZN_262_ZN_2131
3m67_A_ZN_263_ZN_2129
3mdz_A_ZN_263_ZN_1996
3mhc_A_ZN_262_ZN_2071
3mhi_A_ZN_262_ZN_2106
3mhl_A_ZN_262_ZN_2060
3mhm_A_ZN_262_ZN_2175
3mho_A_ZN_262_ZN_2137
3mnh_A_ZN_262_ZN_2078
3mni_A_ZN_262_ZN_2073
3mnj_A_ZN_262_ZN_2077
3mnk_A_ZN_262_ZN_2083
3mnu_A_ZN_262_ZN_2096
3mwo_A_ZN_262_ZN_8358
3mwo_B_ZN_262_ZN_8359
3znc_A_ZN_1_ZN_2474
4ca2_A_ZN_262_ZN_2031
4cac_A_ZN_262_ZN_2041
5ca2_A_ZN_262_ZN_2030
5cac_A_ZN_262_ZN_2041
6ca2_A_ZN_262_ZN_2035
7ca2_A_ZN_262_ZN_2028
8ca2_A_ZN_262_ZN_2034
9ca2_A_ZN_262_ZN_2036

------- SF_176 --------
1gx1_A_ZN_1157_ZN_3529
1gx1_B_ZN_1159_ZN_3582
1gx1_C_ZN_1157_ZN_3614
1h47_A_ZN_1158_ZN_7038
1h47_B_ZN_1158_ZN_7081
1h47_C_ZN_1158_ZN_7103
1h47_D_ZN_1158_ZN_7161
1h47_E_ZN_1158_ZN_7204
1h47_F_ZN_1158_ZN_7226
1h48_A_ZN_900_ZN_7157
1h48_B_ZN_900_ZN_7195
1h48_C_ZN_900_ZN_7233
1h48_D_ZN_900_ZN_7272
1h48_E_ZN_900_ZN_7311
1h48_F_ZN_900_ZN_7349
1jy8_A_ZN_300_ZN_1403
1t0a_A_ZN_661_ZN_3559
1t0a_B_ZN_760_ZN_3585
1t0a_C_ZN_860_ZN_3587
1u3l_A_ZN_701_ZN_1160
1u3p_A_ZN_160_ZN_1159
1u40_A_ZN_160_ZN_1159
1w57_A_ZN_1371_ZN_2910
1yqn_A_ZN_400_ZN_1183
2amt_A_ZN_1900_ZN_7003
2amt_B_ZN_2900_ZN_7060
2amt_C_ZN_3900_ZN_7061
2amt_D_ZN_4900_ZN_7136
2amt_E_ZN_5900_ZN_7193
2amt_F_ZN_6900_ZN_7268
2gzl_A_ZN_900_ZN_1190

2pmp_A_ZN_500_ZN_1222
2uzh_A_ZN_1157_ZN_3409
2uzh_B_ZN_1157_ZN_3470
2uzh_C_ZN_1157_ZN_3555
3b6n_A_ZN_1157_ZN_1140
3elc_A_ZN_801_ZN_3550
3elc_B_ZN_802_ZN_3569
3elc_C_ZN_803_ZN_3588
3eor_A_ZN_800_ZN_1177
3ern_A_ZN_800_ZN_6982
3ern_B_ZN_800_ZN_7025
3ern_D_ZN_800_ZN_7071
3ern_E_ZN_800_ZN_7114
3esj_A_ZN_900_ZN_1217
3f0d_A_ZN_163_ZN_6812
3f0d_B_ZN_163_ZN_6813
3f0d_C_ZN_163_ZN_6814
3f0d_D_ZN_163_ZN_6815
3f0d_E_ZN_163_ZN_6816
3f0d_F_ZN_163_ZN_6817
3f0e_A_ZN_164_ZN_3356
3f0e_B_ZN_163_ZN_3357
3f0e_C_ZN_163_ZN_3358
3f0f_A_ZN_165_ZN_3519
3f0f_B_ZN_163_ZN_3520
3f0f_C_ZN_164_ZN_3542
3f0g_A_ZN_163_ZN_6856
3f0g_B_ZN_164_ZN_6878
3f0g_C_ZN_164_ZN_6900
3f0g_D_ZN_164_ZN_6922
3f0g_E_ZN_164_ZN_6944
3f0g_F_ZN_164_ZN_6966
3fba_A_ZN_900_ZN_1193
3ieq_A_ZN_163_ZN_3448
3ieq_B_ZN_164_ZN_3468
3ieq_C_ZN_163_ZN_3486
3iew_A_ZN_801_ZN_3417
3iew_B_ZN_801_ZN_3472
3iew_C_ZN_801_ZN_3504
3ike_A_ZN_201_ZN_3402
3ike_B_ZN_201_ZN_3421
3ike_C_ZN_201_ZN_3430
3ikf_A_ZN_201_ZN_3419
3ikf_B_ZN_201_ZN_3445
3ikf_C_ZN_201_ZN_3457
3jvh_A_ZN_163_ZN_3454
3jvh_B_ZN_163_ZN_3477
3jvh_C_ZN_163_ZN_3494
3k14_A_ZN_163_ZN_3388
3k14_B_ZN_163_ZN_3405
3k14_C_ZN_163_ZN_3420
3k2x_A_ZN_163_ZN_3364
3k2x_B_ZN_163_ZN_3384
3k2x_C_ZN_163_ZN_3419
3ke1_A_ZN_163_ZN_3257
3ke1_B_ZN_163_ZN_3284
3ke1_C_ZN_163_ZN_3310
3mbm_A_ZN_163_ZN_3433

3mbm_B_ZN_163_ZN_3468
3mbm_C_ZN_164_ZN_3497
3p0z_A_ZN_164_ZN_3432
3p0z_B_ZN_163_ZN_3462
3p0z_C_ZN_164_ZN_3498
3p10_A_ZN_164_ZN_3542
3p10_B_ZN_163_ZN_3573
3p10_C_ZN_164_ZN_3592

------- SF_177 --------
1lbu_A_ZN_214_ZN_1565
1r44_A_ZN_203_ZN_9913
1r44_B_ZN_203_ZN_9914
1r44_C_ZN_203_ZN_9915
1r44_D_ZN_203_ZN_9916
1r44_E_ZN_203_ZN_9917
1r44_F_ZN_203_ZN_9918
1u10_A_ZN_400_ZN_11224
1u10_B_ZN_400_ZN_11231
1u10_C_ZN_400_ZN_11237
1u10_D_ZN_400_ZN_11243
1u10_E_ZN_400_ZN_11250
1u10_F_ZN_400_ZN_11257
1vhh_A_ZN_400_ZN_1559
2vo9_A_ZN_501_ZN_3463
2vo9_B_ZN_501_ZN_3474
2vo9_C_ZN_501_ZN_3485
2wfq_A_ZN_1204_ZN_1322
2wfr_A_ZN_1204_ZN_1327
2wfx_A_ZN_1192_ZN_4468
2wg3_A_ZN_1199_ZN_8816
2wg3_B_ZN_1193_ZN_8818
2wg4_A_ZN_1190_ZN_4458
3d1m_A_ZN_1_ZN_3916
3d1m_B_ZN_2_ZN_3919
3ho5_H_ZN_400_ZN_8255
3m1n_A_ZN_200_ZN_2660
3m1n_B_ZN_700_ZN_2676
3mxw_A_ZN_400_ZN_4568
3n1f_A_ZN_2_ZN_7371
3n1f_B_ZN_1_ZN_7375
3n1g_A_ZN_191_ZN_4108
3n1g_B_ZN_192_ZN_4106
3n1m_B_ZN_194_ZN_4099
3n1o_A_ZN_194_ZN_3634
3n1o_B_ZN_194_ZN_3635
3n1o_C_ZN_194_ZN_3636
3n1p_B_ZN_194_ZN_2045
3n1q_A_ZN_190_ZN_5767
3n1q_B_ZN_190_ZN_5764
3n1q_E_ZN_190_ZN_5770
3n1r_A_ZN_196_ZN_1208

------- SF_178 --------
1evk_A_ZN_1_ZN_6549
1evk_B_ZN_2_ZN_6550
1evl_A_ZN_1_ZN_13067
1evl_B_ZN_1_ZN_13098

1evl_C_ZN_1_ZN_13129
1evl_D_ZN_1_ZN_13160
1fyf_A_ZN_650_ZN_6536
1fyf_B_ZN_650_ZN_6566
1kog_A_ZN_1_ZN_32521
1kog_B_ZN_1_ZN_32552
1kog_C_ZN_1_ZN_32583
1kog_D_ZN_1_ZN_32614
1kog_E_ZN_1_ZN_32645
1kog_F_ZN_1_ZN_32676
1kog_G_ZN_1_ZN_32707
1kog_H_ZN_1_ZN_32738
1nyq_A_ZN_1001_ZN_10461
1nyq_B_ZN_2001_ZN_10492
1nyr_A_ZN_1001_ZN_10390
1nyr_B_ZN_2001_ZN_10431
1qf6_A_ZN_1001_ZN_6828
2cim_A_ZN_1503_ZN_7866
2cim_B_ZN_1503_ZN_7869
2cj9_A_ZN_1503_ZN_7918
2cj9_B_ZN_1503_ZN_7949
2cja_A_ZN_1503_ZN_7901
2cja_B_ZN_1503_ZN_7934
2cjb_A_ZN_1503_ZN_7945
2cjb_B_ZN_1503_ZN_7947
3a31_A_ZN_743_ZN_3722
3a32_A_ZN_708_ZN_3736
3mey_A_ZN_1000_ZN_4535
3mey_B_ZN_1000_ZN_4568
3mf1_A_ZN_1000_ZN_4548
3mf1_B_ZN_1000_ZN_4576
3mf2_A_ZN_1000_ZN_4684
3mf2_B_ZN_1000_ZN_4720

------- SF_179 --------
1im5_A_ZN_400_ZN_1439
2h0r_A_ZN_301_ZN_12349
2h0r_B_ZN_301_ZN_12350
2h0r_C_ZN_301_ZN_12351
2h0r_D_ZN_301_ZN_12352
2h0r_E_ZN_301_ZN_12353
2h0r_F_ZN_301_ZN_12354
2h0r_G_ZN_301_ZN_12355
2wt9_A_ZN_1212_ZN_3356
2wt9_B_ZN_1212_ZN_3384
2wta_A_ZN_1215_ZN_1756
3eef_A_ZN_183_ZN_2636
3eef_B_ZN_183_ZN_2637

------- SF_180 --------
1sr9_A_ZN_704_ZN_8671
1sr9_B_ZN_703_ZN_8681
2nx9_A_ZN_601_ZN_7003
2nx9_B_ZN_602_ZN_7004
2qf7_A_ZN_1157_ZN_16187
2qf7_B_ZN_1155_ZN_16254
3ble_A_ZN_1003_ZN_2426
3blf_A_ZN_1003_ZN_2417

3bli_A_ZN_1003_ZN_2449
3fig_B_ZN_645_ZN_8780
3hps_A_ZN_701_ZN_8788
3hps_B_ZN_701_ZN_8804
3hpz_A_ZN_701_ZN_8607,3hpz_A_ZN_701_ZN_8608
3hpz_B_ZN_702_ZN_8616
3ivt_A_ZN_500_ZN_6152
3ivt_B_ZN_500_ZN_6164
3mi3_A_ZN_500_ZN_5822
3mi3_B_ZN_500_ZN_5824

------- SF_181 --------
1qrg_A_ZN_214_ZN_1561
1qrl_A_ZN_214_ZN_1626
1qrm_A_ZN_214_ZN_1557
1thj_A_ZN_214_ZN_4834
1thj_B_ZN_214_ZN_4835
1thj_C_ZN_214_ZN_4836
1v3w_A_ZN_3001_ZN_1364
1v67_A_ZN_3001_ZN_1338
2fko_A_ZN_3001_ZN_1338
3kwc_A_ZN_300_ZN_9401
3kwc_B_ZN_300_ZN_9407
3kwc_C_ZN_300_ZN_9412
3kwc_D_ZN_300_ZN_9417
3kwc_E_ZN_300_ZN_9419
3kwc_F_ZN_300_ZN_9432
3kwd_A_ZN_194_ZN_1337
3kwe_A_ZN_194_ZN_1360

------- SF_182 --------
1b66_A_ZN_401_ZN_2205
1b66_B_ZN_402_ZN_2223
1b6z_A_ZN_401_ZN_2204
1b6z_B_ZN_402_ZN_2205
1gtq_A_ZN_200_ZN_2703
1gtq_B_ZN_200_ZN_2704
1y13_A_ZN_174_ZN_3976
1y13_B_ZN_174_ZN_3992
1y13_C_ZN_174_ZN_4010
2a0s_A_ZN_281_ZN_2727
2g64_A_ZN_2001_ZN_1190
2oba_A_ZN_200_ZN_5970
2oba_B_ZN_200_ZN_5971
2oba_C_ZN_200_ZN_5972
2oba_D_ZN_200_ZN_5973
2oba_E_ZN_200_ZN_5974
2oba_F_ZN_200_ZN_5975
3jyg_A_ZN_200_ZN_9009
3jyg_B_ZN_200_ZN_9010
3jyg_C_ZN_200_ZN_9011
3jyg_D_ZN_200_ZN_9012
3jyg_E_ZN_200_ZN_9013
3jyg_F_ZN_200_ZN_9014
3lx3_A_ZN_201_ZN_1398
3lze_A_ZN_201_ZN_1376
3m0n_A_ZN_201_ZN_1366

------- SF_183 --------
1dzu_P_ZN_999_ZN_1635
1dzv_P_ZN_999_ZN_1603
1dzw_P_ZN_999_ZN_1603
1dzx_P_ZN_999_ZN_1625
1dzy_P_ZN_999_ZN_1609
1dzz_P_ZN_999_ZN_1624
1e46_S_ZN_999_ZN_1616
1e47_S_ZN_999_ZN_1634
1e48_S_ZN_999_ZN_1637
1e49_S_ZN_999_ZN_1618
1e4a_S_ZN_999_ZN_1619
1e4b_S_ZN_999_ZN_1625
1e4c_S_ZN_999_ZN_1640
1fua_A_ZN_216_ZN_1610
1gt7_A_ZN_275_ZN_42501
1gt7_B_ZN_275_ZN_42512
1gt7_C_ZN_275_ZN_42523
1gt7_D_ZN_275_ZN_42534
1gt7_E_ZN_275_ZN_42545
1gt7_F_ZN_275_ZN_42556
1gt7_G_ZN_275_ZN_42567
1gt7_H_ZN_275_ZN_42578
1gt7_I_ZN_275_ZN_42589
1gt7_J_ZN_275_ZN_42600
1gt7_K_ZN_275_ZN_42611
1gt7_L_ZN_275_ZN_42622
1gt7_M_ZN_275_ZN_42633
1gt7_N_ZN_275_ZN_42644
1gt7_O_ZN_275_ZN_42655
1gt7_P_ZN_275_ZN_42666
1gt7_Q_ZN_275_ZN_42677
1gt7_R_ZN_275_ZN_42688
1gt7_S_ZN_275_ZN_42699
1gt7_T_ZN_275_ZN_42710
1jdi_A_ZN_301_ZN_10417
1jdi_B_ZN_302_ZN_10418
1jdi_C_ZN_303_ZN_10419
1jdi_D_ZN_304_ZN_10420
1jdi_E_ZN_305_ZN_10421
1jdi_F_ZN_306_ZN_10422
1k0w_A_ZN_301_ZN_10417
1k0w_B_ZN_302_ZN_10418
1k0w_C_ZN_303_ZN_10419
1k0w_D_ZN_304_ZN_10420
1k0w_E_ZN_305_ZN_10421
1k0w_F_ZN_306_ZN_10422
1ojr_A_ZN_1275_ZN_2237
2uyu_A_ZN_1275_ZN_4291
2uyu_E_ZN_1275_ZN_4293
2uyv_A_ZN_1276_ZN_8507
2uyv_B_ZN_1276_ZN_8538
2uyv_C_ZN_1276_ZN_8549
2uyv_D_ZN_1276_ZN_8560
2v29_A_ZN_1274_ZN_4262
2v29_A_ZN_1275_ZN_4263
2v2a_A_ZN_1275_ZN_2270
2v2b_A_ZN_1274_ZN_2114

2v9e_A_ZN_1275_ZN_4575
2v9e_B_ZN_1275_ZN_4594
2v9f_A_ZN_1279_ZN_2144
2v9g_A_ZN_1276_ZN_8523
2v9g_B_ZN_1277_ZN_8544
2v9g_C_ZN_1276_ZN_8555
2v9g_D_ZN_1276_ZN_8566
2v9i_A_ZN_1275_ZN_4243
2v9i_B_ZN_1275_ZN_4250
2v9l_A_ZN_1275_ZN_4535
2v9m_A_ZN_1275_ZN_4705
2v9m_B_ZN_1275_ZN_4737
2v9n_A_ZN_1275_ZN_9163
2v9n_B_ZN_1275_ZN_9203
2v9n_C_ZN_1275_ZN_9243
2v9n_D_ZN_1275_ZN_9283
2v9o_A_ZN_1275_ZN_4298
2v9o_E_ZN_1275_ZN_4300
3fua_A_ZN_216_ZN_1604
3m4r_A_ZN_220_ZN_1662
4fua_A_ZN_216_ZN_1595

------- SF_184 --------
1a85_A_ZN_998_ZN_1501
1a86_A_ZN_998_ZN_1283
1ayk_A_ZN_171_ZN_2568
1b3d_A_ZN_302_ZN_2726
1b3d_B_ZN_302_ZN_2731
1b8y_A_ZN_302_ZN_1335
1biw_A_ZN_302_ZN_2726
1biw_B_ZN_802_ZN_2731
1bm6_A_ZN_257_ZN_2677
1bqo_A_ZN_302_ZN_2726
1bqo_B_ZN_302_ZN_2731
1bqq_M_ZN_290_ZN_2840
1buv_M_ZN_1187_ZN_2840
1bzs_A_ZN_998_ZN_1306
1c3i_A_ZN_261_ZN_2732
1c3i_B_ZN_261_ZN_2737
1c8t_A_ZN_261_ZN_2654
1c8t_B_ZN_261_ZN_2691
1caq_A_ZN_302_ZN_1342
1cge_A_ZN_302_ZN_1545
1cgf_A_ZN_302_ZN_3098
1cgf_B_ZN_302_ZN_3103
1cgl_A_ZN_302_ZN_3201
1cgl_B_ZN_302_ZN_3285
1ciz_A_ZN_302_ZN_1342
1ck7_A_ZN_991_ZN_4933
1cqr_A_ZN_1302_ZN_2726
1cqr_B_ZN_2302_ZN_2731
1cxv_A_ZN_3_ZN_2572
1cxv_B_ZN_4_ZN_2604
1d5j_A_ZN_302_ZN_2726
1d5j_B_ZN_802_ZN_2755
1d7x_A_ZN_302_ZN_2726
1d7x_B_ZN_802_ZN_2754
1d8f_A_ZN_302_ZN_2726

1d8f_B_ZN_802_ZN_2731
1d8m_A_ZN_302_ZN_2726
1d8m_B_ZN_802_ZN_2731
1eak_A_ZN_996_ZN_13361
1eak_B_ZN_996_ZN_13375
1eak_C_ZN_996_ZN_13389
1eak_D_ZN_996_ZN_13398
1eub_A_ZN_275_ZN_2630
1fbl_A_ZN_997_ZN_2972
1fls_A_ZN_167_ZN_2431
1fm1_A_ZN_167_ZN_2431
1g05_A_ZN_302_ZN_2650
1g05_B_ZN_802_ZN_2655
1g49_A_ZN_302_ZN_2726
1g49_B_ZN_802_ZN_2731
1g4k_A_ZN_302_ZN_4022
1g4k_B_ZN_302_ZN_4050
1g4k_C_ZN_302_ZN_4084
1gkc_A_ZN_1451_ZN_2542
1gkc_B_ZN_1452_ZN_2572
1gkd_A_ZN_1451_ZN_2534
1gkd_B_ZN_1451_ZN_2562
1gxd_A_ZN_1633_ZN_12939
1gxd_B_ZN_1633_ZN_12947
1hfc_A_ZN_276_ZN_1247
1hfs_A_ZN_258_ZN_1274
1hov_A_ZN_165_ZN_2517
1hv5_A_ZN_5501_ZN_7997
1hv5_B_ZN_5504_ZN_8102
1hv5_C_ZN_5507_ZN_8207
1hv5_D_ZN_5510_ZN_8312
1hv5_E_ZN_5513_ZN_8417
1hv5_F_ZN_5516_ZN_8522
1hy7_A_ZN_302_ZN_2656
1hy7_B_ZN_802_ZN_2661
1i73_A_ZN_998_ZN_1347
1i76_A_ZN_998_ZN_1318
1jan_A_ZN_998_ZN_1596
1jao_A_ZN_998_ZN_1541
1jap_A_ZN_998_ZN_1267
1jaq_A_ZN_998_ZN_1514
1jh1_A_ZN_998_ZN_1253
1jiz_A_ZN_258_ZN_2610
1jiz_B_ZN_258_ZN_2642
1jj9_A_ZN_998_ZN_1253
1jk3_A_ZN_401_ZN_1293
1kbc_A_ZN_998_ZN_2593
1kbc_B_ZN_998_ZN_2625
1l6j_A_ZN_501_ZN_3183
1mmb_A_ZN_998_ZN_1514
1mmp_A_ZN_2_ZN_2552
1mmp_B_ZN_2_ZN_2588
1mmq_A_ZN_2_ZN_1271
1mmr_A_ZN_2_ZN_1276
1mnc_A_ZN_282_ZN_1236
1os2_A_ZN_870_ZN_7778
1os2_B_ZN_370_ZN_7788
1os2_C_ZN_470_ZN_7797

1os2_D_ZN_570_ZN_7805
1os2_E_ZN_670_ZN_7815
1os2_F_ZN_770_ZN_7824
1os9_A_ZN_902_ZN_7778
1os9_B_ZN_907_ZN_7783
1os9_C_ZN_912_ZN_7788
1os9_D_ZN_917_ZN_7793
1os9_E_ZN_922_ZN_7798
1os9_F_ZN_927_ZN_7803
1q3a_A_ZN_465_ZN_3751
1q3a_B_ZN_470_ZN_3756
1q3a_C_ZN_475_ZN_3761
1qia_A_ZN_302_ZN_5122
1qia_B_ZN_302_ZN_5127
1qia_C_ZN_302_ZN_5132
1qia_D_ZN_302_ZN_5137
1qib_A_ZN_502_ZN_1275
1qic_A_ZN_502_ZN_5106
1qic_B_ZN_502_ZN_5111
1qic_C_ZN_502_ZN_5116
1qic_D_ZN_502_ZN_5121
1rm8_A_ZN_501_ZN_1347
1rmz_A_ZN_265_ZN_1249
1ros_A_ZN_401_ZN_2500
1ros_B_ZN_501_ZN_2540
1slm_A_ZN_258_ZN_2182
1sln_A_ZN_258_ZN_1611
1su3_A_ZN_915_ZN_6684
1su3_B_ZN_915_ZN_6697
1uea_A_ZN_2_ZN_5524
1uea_C_ZN_2_ZN_5529
1ums_A_ZN_2_ZN_2575,1ums_A_ZN_2_ZN_2576
1umt_A_ZN_2_ZN_2575
1usn_A_ZN_258_ZN_1332
1utt_A_ZN_1268_ZN_1283
1utz_A_ZN_1269_ZN_2569
1utz_B_ZN_1268_ZN_2609
1xuc_A_ZN_1262_ZN_2671
1xuc_B_ZN_1262_ZN_2703
1xud_A_ZN_1262_ZN_2671
1xud_B_ZN_1262_ZN_2705
1xur_A_ZN_1262_ZN_2671
1xur_B_ZN_1262_ZN_2701
1y93_A_ZN_265_ZN_2418
1ycm_A_ZN_265_ZN_2425
1you_A_ZN_302_ZN_2641
1you_B_ZN_304_ZN_2683
1z3j_A_ZN_265_ZN_2425
1zp5_A_ZN_998_ZN_1287
1zs0_A_ZN_998_ZN_1286
1ztq_A_ZN_551_ZN_5036
1ztq_B_ZN_553_ZN_5076
1ztq_C_ZN_555_ZN_5116
1ztq_D_ZN_557_ZN_5156
1zvx_A_ZN_998_ZN_1286
2ayk_A_ZN_171_ZN_2388
2clt_A_ZN_1201_ZN_5804
2clt_B_ZN_1401_ZN_5810

2d1n_A_ZN_271_ZN_2648
2d1n_B_ZN_281_ZN_2688
2d1o_A_ZN_258_ZN_2702
2d1o_B_ZN_263_ZN_2740
2ddy_A_ZN_176_ZN_2677
2e2d_A_ZN_501_ZN_2729
2hu6_A_ZN_265_ZN_1241
2j0t_A_ZN_1266_ZN_6498
2j0t_B_ZN_1267_ZN_6503
2j0t_C_ZN_1264_ZN_6508
2jnp_A_ZN_249_ZN_2491
2jsd_A_ZN_275_ZN_2406
2jt5_A_ZN_257_ZN_2494
2jt6_A_ZN_257_ZN_2494
2k2g_A_ZN_3_ZN_2542
2ovx_A_ZN_445_ZN_2546
2ovx_B_ZN_445_ZN_2590
2ovz_A_ZN_445_ZN_2514
2ovz_B_ZN_445_ZN_2564
2ow0_A_ZN_445_ZN_2514
2ow0_B_ZN_445_ZN_2555
2ow1_A_ZN_445_ZN_2514
2ow1_B_ZN_445_ZN_2550
2ow2_A_ZN_445_ZN_2490
2ow2_B_ZN_445_ZN_2516
2ow9_A_ZN_602_ZN_2699
2ow9_B_ZN_607_ZN_2742
2oxu_A_ZN_265_ZN_1241
2oxw_A_ZN_265_ZN_1259
2oxz_A_ZN_265_ZN_1274
2oy2_A_ZN_998_ZN_2523
2oy2_F_ZN_998_ZN_2527
2oy4_A_ZN_998_ZN_2486
2oy4_F_ZN_998_ZN_2490
2ozr_A_ZN_4002_ZN_10568
2ozr_B_ZN_4007_ZN_10605
2ozr_C_ZN_4012_ZN_10642
2ozr_D_ZN_4017_ZN_10684
2ozr_E_ZN_4022_ZN_10726
2ozr_F_ZN_4027_ZN_10768
2ozr_G_ZN_4032_ZN_10810
2ozr_H_ZN_4037_ZN_10847
2pjt_A_ZN_303_ZN_5160
2pjt_B_ZN_303_ZN_5197
2pjt_C_ZN_303_ZN_5233
2pjt_D_ZN_303_ZN_5270
2poj_A_ZN_265_ZN_2508
2srt_A_ZN_258_ZN_2677
2tcl_A_ZN_172_ZN_1293
2usn_A_ZN_258_ZN_1315
2w0d_A_ZN_1265_ZN_5073
2w0d_B_ZN_1265_ZN_5118
2w0d_C_ZN_1265_ZN_5150
2w0d_D_ZN_1265_ZN_5192
2wo8_A_ZN_1269_ZN_5107
2wo8_B_ZN_1270_ZN_5168
2wo8_C_ZN_1269_ZN_5177
2wo8_D_ZN_1268_ZN_5233

2wo9_A_ZN_1269_ZN_5104
2wo9_B_ZN_1269_ZN_5173
2wo9_C_ZN_1270_ZN_5193
2wo9_D_ZN_1268_ZN_5258
2woa_A_ZN_1271_ZN_5119
2woa_B_ZN_1271_ZN_5186
2woa_C_ZN_1269_ZN_5191
2woa_D_ZN_1268_ZN_5250
2xs3_A_ZN_998_ZN_2707
2xs3_B_ZN_998_ZN_2709
2xs4_A_ZN_998_ZN_1355
2z2d_A_ZN_265_ZN_2415
3ayk_A_ZN_171_ZN_2388
3ba0_A_ZN_472_ZN_2993
3dng_A_ZN_998_ZN_2602
3dng_B_ZN_998_ZN_2639
3dpe_A_ZN_998_ZN_1286
3dpf_A_ZN_998_ZN_2569
3dpf_B_ZN_998_ZN_2615
3ehx_A_ZN_265_ZN_1241
3ehy_A_ZN_265_ZN_1241
3elm_A_ZN_301_ZN_2635
3elm_B_ZN_301_ZN_2675
3f15_A_ZN_265_ZN_1241
3f16_A_ZN_265_ZN_1241
3f17_A_ZN_265_ZN_1241
3f18_A_ZN_265_ZN_1241
3f19_A_ZN_265_ZN_1241
3f1a_A_ZN_265_ZN_1241
3i7g_A_ZN_1262_ZN_2622
3i7g_B_ZN_2262_ZN_2656
3i7i_A_ZN_1262_ZN_2594
3i7i_B_ZN_2262_ZN_2636
3kec_A_ZN_902_ZN_2657
3kec_B_ZN_902_ZN_2710
3kej_A_ZN_902_ZN_2535
3kej_B_ZN_902_ZN_2572
3kek_A_ZN_902_ZN_2555
3kek_B_ZN_902_ZN_2593
3kry_A_ZN_1998_ZN_5207
3kry_B_ZN_2998_ZN_5246
3kry_C_ZN_3998_ZN_5285
3kry_D_ZN_4998_ZN_5324
3lik_A_ZN_265_ZN_1288
3lil_A_ZN_265_ZN_1323
3lir_A_ZN_265_ZN_1289
3ljg_A_ZN_265_ZN_1300
3lk8_A_ZN_265_ZN_1241
3ma2_A_ZN_295_ZN_4567
3ma2_D_ZN_295_ZN_4563
3n2u_A_ZN_265_ZN_1241
3n2v_A_ZN_265_ZN_1241
3nx7_A_ZN_265_ZN_1241
3usn_A_ZN_170_ZN_2620
456c_A_ZN_273_ZN_3019
456c_B_ZN_273_ZN_3070
4ayk_A_ZN_171_ZN_2568
830c_A_ZN_273_ZN_3147

830c_B_ZN_273_ZN_3198
966c_A_ZN_266_ZN_1512

------- SF_185 --------
1xcr_A_ZN_1001_ZN_4863
1xcr_B_ZN_1002_ZN_4868
1xv2_A_ZN_801_ZN_7412
1xv2_B_ZN_802_ZN_7413
1xv2_C_ZN_803_ZN_7414
1xv2_D_ZN_804_ZN_7415
2h6l_A_ZN_401_ZN_3346
2h6l_B_ZN_402_ZN_3351
2h6l_C_ZN_403_ZN_3356
2p6y_A_ZN_201_ZN_1019

------- SF_186 --------
1h1z_A_ZN_1224_ZN_3285
1h1z_B_ZN_1224_ZN_3291
1tqx_A_ZN_900_ZN_3506
1tqx_B_ZN_902_ZN_3512
2fli_A_ZN_1981_ZN_19751
2fli_B_ZN_1982_ZN_19766
2fli_C_ZN_1983_ZN_19781
2fli_D_ZN_1984_ZN_19796
2fli_E_ZN_1985_ZN_19811
2fli_F_ZN_1986_ZN_19826
2fli_G_ZN_1991_ZN_19841
2fli_H_ZN_1992_ZN_19856
2fli_I_ZN_1993_ZN_19871
2fli_J_ZN_1994_ZN_19886
2fli_K_ZN_1995_ZN_19901
2fli_L_ZN_1996_ZN_19916

------- SF_187 --------
2qfi_B_ZN_301_ZN_4413,2qfi_B_ZN_304_ZN_4416
3byr_A_ZN_501_ZN_735,3byr_A_ZN_502_ZN_736
3byr_A_ZN_503_ZN_737
3h90_A_ZN_291_ZN_8720,3h90_A_ZN_293_ZN_8722
3h90_A_ZN_292_ZN_8721
3h90_A_ZN_4_ZN_8719
3h90_B_ZN_291_ZN_8725
3h90_B_ZN_292_ZN_8726,3h90_B_ZN_294_ZN_8728
3h90_B_ZN_293_ZN_8727
3h90_C_ZN_1_ZN_8731,3h90_C_ZN_3_ZN_8733
3h90_C_ZN_291_ZN_8734
3h90_C_ZN_2_ZN_8732
3h90_D_ZN_292_ZN_8739,3h90_D_ZN_5_ZN_8737
3h90_D_ZN_293_ZN_8740
3h90_D_ZN_6_ZN_8738

------- SF_188 --------
1m55_A_ZN_202_ZN_3163
1m55_B_ZN_201_ZN_3166
1qx0_A_ZN_294_ZN_2808

------- SF_189 --------
1oi0_A_ZN_1122_ZN_3386
1oi0_B_ZN_1123_ZN_3395

1oi0_C_ZN_1123_ZN_3396
1oi0_D_ZN_1123_ZN_3397
1r5x_A_ZN_122_ZN_1653
1r5x_B_ZN_122_ZN_1654
2znr_A_ZN_2_ZN_1435

------- SF_190 --------
1qwy_A_ZN_400_ZN_1824
2b0p_A_ZN_400_ZN_1983
2b0p_B_ZN_401_ZN_1997
2b13_A_ZN_400_ZN_2007
2b13_B_ZN_401_ZN_2018
2b44_A_ZN_400_ZN_2024
2b44_B_ZN_401_ZN_2035
3it5_A_ZN_183_ZN_5632
3it5_B_ZN_183_ZN_5633
3it5_E_ZN_183_ZN_5634
3it5_G_ZN_183_ZN_5635
3it7_A_ZN_183_ZN_2876
3it7_B_ZN_183_ZN_2899

------- SF_191 --------
1ck1_A_ZN_300_ZN_1946
1cqv_A_ZN_240_ZN_1867
1ha5_A_ZN_2221_ZN_7039
1ha5_B_ZN_3221_ZN_7040
1ha5_C_ZN_4221_ZN_7041
1ha5_D_ZN_5221_ZN_7042
1i4p_A_ZN_240_ZN_1909
1i4q_A_ZN_240_ZN_1909
1i4r_A_ZN_240_ZN_1909
1i4x_A_ZN_240_ZN_1916
1l0y_B_ZN_702_ZN_7105
1l0y_D_ZN_704_ZN_7108
1ste_A_ZN_500_ZN_1904
1uns_A_ZN_1237_ZN_1895
1uup_A_ZN_2222_ZN_7253
1uup_B_ZN_3222_ZN_7254
1uup_C_ZN_4222_ZN_7255
1uup_D_ZN_5222_ZN_7256
2aq2_B_ZN_1001_ZN_2767
3bvg_A_ZN_240_ZN_1868
3bvm_A_ZN_240_ZN_1855
3bvz_A_ZN_240_ZN_1868

------- SF_192 --------
2hsi_A_ZN_283_ZN_3487
2hsi_B_ZN_283_ZN_3488
3csq_A_ZN_335_ZN_10465
3csq_B_ZN_335_ZN_10466
3csq_C_ZN_335_ZN_10467
3csq_D_ZN_335_ZN_10468

------- SF_193 --------
2nly_A_ZN_300_ZN_1652
2yxo_A_ZN_501_ZN_4209
2yxo_B_ZN_1501_ZN_4223
2yz5_A_ZN_501_ZN_4204

2yz5_B_ZN_1501_ZN_4219
2z4g_A_ZN_501_ZN_4190
2z4g_B_ZN_1501_ZN_4193

------- SF_194 --------
1mzb_A_ZN_201_ZN_1040
2o03_A_ZN_203_ZN_958

------- SF_195 --------
1u1h_A_ZN_766_ZN_5785
1u1j_A_ZN_766_ZN_5785
1u22_A_ZN_766_ZN_5785
1xdj_A_ZN_1051_ZN_11211
1xdj_B_ZN_1052_ZN_11241
1xpg_A_ZN_1887_ZN_11645
1xpg_B_ZN_1888_ZN_11692
3bq5_A_ZN_800_ZN_11233
3bq5_B_ZN_801_ZN_11246
3bq6_A_ZN_800_ZN_11006
3bq6_B_ZN_801_ZN_11007

------- SF_196 --------
3chv_A_ZN_302_ZN_2263
3e02_A_ZN_311_ZN_2490
3e49_A_ZN_500_ZN_9523
3e49_B_ZN_500_ZN_9530
3e49_C_ZN_500_ZN_9558
3e49_D_ZN_500_ZN_9586
3lot_A_ZN_501_ZN_10007
3lot_B_ZN_501_ZN_10020
3lot_C_ZN_501_ZN_10029
3lot_D_ZN_501_ZN_10043
3no5_A_ZN_275_ZN_12571
3no5_B_ZN_275_ZN_12580
3no5_C_ZN_275_ZN_12581
3no5_D_ZN_275_ZN_12606
3no5_E_ZN_275_ZN_12623
3no5_F_ZN_275_ZN_12628

------- SF_197 --------
1ef0_A_ZN_701_ZN_6478,1ef0_A_ZN_701_ZN_6479
1ef0_B_ZN_701_ZN_6480

------- SF_198 --------
1eh6_A_ZN_208_ZN_1274
1eh7_A_ZN_208_ZN_1234
1eh8_A_ZN_208_ZN_1269
1yfh_A_ZN_301_ZN_4945
1yfh_B_ZN_303_ZN_4946
1yfh_C_ZN_302_ZN_4947
3kzy_A_ZN_180_ZN_2509
3kzy_B_ZN_200_ZN_2510
3kzz_A_ZN_180_ZN_1232
3l00_A_ZN_180_ZN_1249

------- SF_199 --------
1enq_A_ZN_238_ZN_7041
1enq_B_ZN_238_ZN_7042

1enq_C_ZN_238_ZN_7043
1enq_D_ZN_238_ZN_7044
1enr_A_ZN_239_ZN_1826
3enr_A_ZN_1001_ZN_3618
3enr_B_ZN_1002_ZN_3621

------- SF_200 --------
1r61_A_ZN_1001_ZN_3256
1r61_B_ZN_1002_ZN_3282,1r61_B_ZN_1002_ZN_3283
3krv_B_ZN_206_ZN_3246

------- SF_201 --------
1oek_A_ZN_1194_ZN_1516
1s7d_A_ZN_201_ZN_1529
1txl_A_ZN_216_ZN_1539

------- SF_202 --------
1yg9_A_ZN_401_ZN_2611
2nr6_A_ZN_602_ZN_11691
2nr6_B_ZN_602_ZN_11692
3liz_A_ZN_333_ZN_5962

------- SF_203 --------
1yt3_A_ZN_1001_ZN_3094,1yt3_A_ZN_1001_ZN_3095
3g0z_A_ZN_333_ZN_2035

------- SF_204 --------
2cs7_A_ZN_201_ZN_1299
2cs7_B_ZN_202_ZN_1300
2cs7_C_ZN_203_ZN_1301

------- SF_205 --------
2faw_A_ZN_1001_ZN_4176
2faw_B_ZN_1002_ZN_4223

------- SF_206 --------
2fgy_A_ZN_621_ZN_7374
2fgy_B_ZN_721_ZN_7376

------- SF_207 --------
2ijd_1_ZN_645_ZN_10123
2ijd_2_ZN_645_ZN_10144
3ol6_A_ZN_2001_ZN_17819
3ol6_E_ZN_2002_ZN_17840
3ol6_I_ZN_2003_ZN_17853
3ol6_M_ZN_2004_ZN_17870
3ol7_I_ZN_2003_ZN_17920
3ol7_M_ZN_2004_ZN_17971
3ol8_A_ZN_2004_ZN_17779
3ol8_E_ZN_2003_ZN_17817
3ol8_I_ZN_2002_ZN_17843
3ol8_M_ZN_2001_ZN_17861
3ol9_A_ZN_2001_ZN_17924
3ol9_E_ZN_2002_ZN_17946
3ol9_I_ZN_2003_ZN_17968
3ol9_M_ZN_2004_ZN_17994
3ola_A_ZN_2004_ZN_17841
3ola_E_ZN_2003_ZN_17874

3ola_I_ZN_2002_ZN_17887
3ola_M_ZN_2001_ZN_17933
3olb_A_ZN_2001_ZN_17819
3olb_E_ZN_2002_ZN_17855
3olb_I_ZN_2003_ZN_17899
3olb_M_ZN_2004_ZN_17939

------- SF_208 --------
2j7u_A_ZN_1884_ZN_4703
2j7w_A_ZN_1884_ZN_4623

------- SF_209 --------
2jox_A_ZN_110_ZN_1641

------- SF_210 --------
2l0z_A_ZN_486_ZN_670

------- SF_211 --------
2o03_A_ZN_201_ZN_956

------- SF_212 --------
2oog_A_ZN_403_ZN_13320
2oog_B_ZN_401_ZN_13372
2oog_C_ZN_406_ZN_13438
2oog_D_ZN_405_ZN_13456
2oog_E_ZN_404_ZN_13516
2oog_F_ZN_402_ZN_13547

------- SF_213 --------
2osd_A_ZN_166_ZN_1199
2oso_A_ZN_163_ZN_1323

------- SF_214 --------
2peb_A_ZN_200_ZN_1875
2peb_B_ZN_200_ZN_1894

------- SF_215 --------
2q1z_B_ZN_197_ZN_5181,2q1z_B_ZN_197_ZN_5191
2q1z_D_ZN_196_ZN_5182,2q1z_D_ZN_197_ZN_5193

------- SF_216 --------
2q08_A_ZN_981_ZN_40825
2q08_B_ZN_982_ZN_40827
2q08_C_ZN_983_ZN_40828
2q08_D_ZN_984_ZN_40829
2q08_E_ZN_985_ZN_40831
2q08_F_ZN_986_ZN_40832
2q08_G_ZN_987_ZN_40833
2q08_H_ZN_988_ZN_40834
2q08_I_ZN_989_ZN_40836
2q08_J_ZN_990_ZN_40837
2q08_K_ZN_991_ZN_40839
2q08_L_ZN_992_ZN_40840
2q6e_A_ZN_501_ZN_10183
2q6e_B_ZN_502_ZN_10185
2q6e_C_ZN_503_ZN_10186
2qee_A_ZN_5021_ZN_41389
2qee_B_ZN_5022_ZN_41391

2qee_C_ZN_5023_ZN_41393
2qee_D_ZN_5024_ZN_41397
2qee_E_ZN_5025_ZN_41399
2qee_F_ZN_5026_ZN_41401
2qee_G_ZN_5027_ZN_41405
2qee_H_ZN_5028_ZN_41407
2qee_I_ZN_5029_ZN_41409
2qee_J_ZN_5030_ZN_41413
2qee_K_ZN_5031_ZN_41415
2qee_L_ZN_5032_ZN_41417
3hk5_A_ZN_430_ZN_6783
3hk5_B_ZN_430_ZN_6802
3hk7_A_ZN_431_ZN_40881
3hk7_B_ZN_429_ZN_40895
3hk7_C_ZN_430_ZN_40913
3hk7_D_ZN_430_ZN_40930
3hk7_E_ZN_431_ZN_40952
3hk7_F_ZN_429_ZN_40966
3hk7_G_ZN_430_ZN_40983
3hk7_H_ZN_430_ZN_41001
3hk7_I_ZN_430_ZN_41019
3hk7_J_ZN_430_ZN_41036
3hk7_K_ZN_431_ZN_41058
3hk7_L_ZN_429_ZN_41071
3hk8_A_ZN_430_ZN_6762
3hk8_B_ZN_430_ZN_6782
3hk9_A_ZN_431_ZN_40882
3hk9_B_ZN_429_ZN_40897
3hk9_C_ZN_430_ZN_40915
3hk9_D_ZN_431_ZN_40937
3hk9_E_ZN_429_ZN_40951
3hk9_F_ZN_430_ZN_40969
3hk9_G_ZN_430_ZN_40988
3hk9_H_ZN_430_ZN_41006
3hk9_I_ZN_430_ZN_41025
3hk9_J_ZN_430_ZN_41043
3hk9_K_ZN_430_ZN_41061
3hk9_L_ZN_430_ZN_41080
3hka_A_ZN_430_ZN_10182
3hka_B_ZN_431_ZN_10205
3hka_C_ZN_429_ZN_10219

------- SF_217 --------
1a1f_A_ZN_201_ZN_1147
1a1f_A_ZN_202_ZN_1148
1a1f_A_ZN_203_ZN_1149
1a1g_A_ZN_201_ZN_1144
1a1g_A_ZN_202_ZN_1145
1a1g_A_ZN_203_ZN_1146
1a1h_A_ZN_201_ZN_1163
1a1h_A_ZN_202_ZN_1164
1a1h_A_ZN_203_ZN_1165
1a1i_A_ZN_201_ZN_1161
1a1i_A_ZN_202_ZN_1162
1a1i_A_ZN_203_ZN_1163
1a1j_A_ZN_201_ZN_1161
1a1j_A_ZN_202_ZN_1162
1a1j_A_ZN_203_ZN_1163

1a1k_A_ZN_201_ZN_1157
1a1k_A_ZN_202_ZN_1158
1a1k_A_ZN_203_ZN_1159
1a1l_A_ZN_201_ZN_1168
1a1l_A_ZN_202_ZN_1169
1a1l_A_ZN_203_ZN_1170
1aay_A_ZN_201_ZN_1183
1aay_A_ZN_202_ZN_1184
1aay_A_ZN_203_ZN_1185
1ard_A_ZN_1_ZN_496
1are_A_ZN_1_ZN_488
1arf_A_ZN_1_ZN_499
1bbo_A_ZN_60_ZN_963
1bbo_A_ZN_61_ZN_964
1f2i_G_ZN_1201_ZN_5011
1f2i_G_ZN_1202_ZN_5012
1f2i_H_ZN_2201_ZN_5013
1f2i_H_ZN_2202_ZN_5014
1f2i_I_ZN_3201_ZN_5015
1f2i_I_ZN_3202_ZN_5016
1f2i_J_ZN_4201_ZN_5017
1f2i_J_ZN_4202_ZN_5018
1f2i_K_ZN_5201_ZN_5019
1f2i_K_ZN_5202_ZN_5020
1f2i_L_ZN_6201_ZN_5021
1f2i_L_ZN_6202_ZN_5022
1fu9_A_ZN_37_ZN_579
1fv5_A_ZN_37_ZN_550
1g2d_C_ZN_301_ZN_2774
1g2d_C_ZN_302_ZN_2775
1g2d_C_ZN_303_ZN_2776
1g2d_F_ZN_304_ZN_2777
1g2d_F_ZN_305_ZN_2778
1g2d_F_ZN_306_ZN_2779
1g2f_C_ZN_301_ZN_2754
1g2f_C_ZN_302_ZN_2755
1g2f_C_ZN_303_ZN_2756
1g2f_F_ZN_304_ZN_2757
1g2f_F_ZN_305_ZN_2758
1g2f_F_ZN_306_ZN_2759
1jk1_A_ZN_201_ZN_1158
1jk1_A_ZN_202_ZN_1159
1jk1_A_ZN_203_ZN_1160
1jk2_A_ZN_201_ZN_1233
1jk2_A_ZN_202_ZN_1234
1jk2_A_ZN_203_ZN_1235
1jn7_A_ZN_37_ZN_586
1klr_A_ZN_31_ZN_500
1kls_A_ZN_31_ZN_499
1llm_C_ZN_301_ZN_2012
1llm_C_ZN_302_ZN_2013
1llm_D_ZN_303_ZN_2014
1llm_D_ZN_304_ZN_2015
1m36_A_ZN_34_ZN_576
1mey_C_ZN_88_ZN_2640
1mey_C_ZN_89_ZN_2641
1mey_C_ZN_90_ZN_2642
1mey_F_ZN_88_ZN_2643

1mey_F_ZN_89_ZN_2644
1mey_F_ZN_90_ZN_2645
1mey_G_ZN_90_ZN_2646
1ncs_A_ZN_61_ZN_798
1p47_A_ZN_201_ZN_2332
1p47_A_ZN_202_ZN_2333
1p47_A_ZN_203_ZN_2334
1p47_B_ZN_204_ZN_2335
1p47_B_ZN_205_ZN_2336
1p47_B_ZN_206_ZN_2337
1p7a_A_ZN_38_ZN_585
1paa_A_ZN_160_ZN_448
1rmd_A_ZN_120_ZN_916
1srk_A_ZN_36_ZN_499
1tf3_A_ZN_102_ZN_2413
1tf3_A_ZN_2_ZN_2414
1tf3_A_ZN_3_ZN_2415
1tf6_A_ZN_201_ZN_5490
1tf6_A_ZN_202_ZN_5491
1tf6_A_ZN_203_ZN_5492
1tf6_A_ZN_204_ZN_5493
1tf6_A_ZN_205_ZN_5494
1tf6_A_ZN_206_ZN_5495
1tf6_D_ZN_201_ZN_5496
1tf6_D_ZN_202_ZN_5497
1tf6_D_ZN_203_ZN_5498
1tf6_D_ZN_204_ZN_5499
1tf6_D_ZN_205_ZN_5500
1tf6_D_ZN_206_ZN_5501
1u85_A_ZN_34_ZN_529
1u86_A_ZN_36_ZN_567
1ubd_C_ZN_501_ZN_1722
1ubd_C_ZN_502_ZN_1723
1ubd_C_ZN_503_ZN_1724
1ubd_C_ZN_504_ZN_1725
1un6_B_ZN_204_ZN_4523
1un6_B_ZN_205_ZN_4524
1un6_B_ZN_206_ZN_4525
1un6_C_ZN_204_ZN_4529
1un6_C_ZN_205_ZN_4530
1un6_C_ZN_206_ZN_4531
1un6_D_ZN_205_ZN_4534
1un6_D_ZN_206_ZN_4535
1va1_A_ZN_100_ZN_605
1va2_A_ZN_100_ZN_531
1va3_A_ZN_100_ZN_505
1wir_A_ZN_201_ZN_1883
1wjp_A_ZN_301_ZN_1629
1wjp_A_ZN_501_ZN_1630
1wjp_A_ZN_701_ZN_1631
1wjv_A_ZN_201_ZN_1123
1wjv_A_ZN_401_ZN_1124
1x3c_A_ZN_201_ZN_1099
1x5w_A_ZN_201_ZN_1024
1x5w_A_ZN_401_ZN_1025
1x6e_A_ZN_201_ZN_1038
1x6e_A_ZN_401_ZN_1039
1x6f_A_ZN_201_ZN_1369

1x6h_A_ZN_201_ZN_1253
1x6h_A_ZN_401_ZN_1254
1xf7_A_ZN_30_ZN_490
1xrz_A_ZN_31_ZN_506
1y0j_B_ZN_37_ZN_1141
1yuj_A_ZN_64_ZN_1581
1zaa_C_ZN_201_ZN_1162
1zaa_C_ZN_202_ZN_1163
1zaa_C_ZN_203_ZN_1164
1zfd_A_ZN_71_ZN_519
1znf_A_ZN_27_ZN_425
1znm_A_ZN_29_ZN_432,1znm_A_ZN_29_ZN_433
1zr9_A_ZN_117_ZN_1040
1zu1_A_ZN_129_ZN_1951
1zu1_A_ZN_130_ZN_1952
2adr_A_ZN_162_ZN_989
2adr_A_ZN_163_ZN_990
2cot_A_ZN_201_ZN_1173
2cot_A_ZN_401_ZN_1174
2csh_A_ZN_200_ZN_1679
2csh_A_ZN_300_ZN_1680
2csh_A_ZN_400_ZN_1681
2ct1_A_ZN_201_ZN_1158
2ct1_A_ZN_401_ZN_1159
2ct5_A_ZN_201_ZN_1093
2ctd_A_ZN_201_ZN_1433
2ctd_A_ZN_401_ZN_1434
2czr_A_ZN_2001_ZN_1833
2d9h_A_ZN_201_ZN_1148
2d9h_A_ZN_401_ZN_1149
2d9k_A_ZN_401_ZN_1078
2d9k_A_ZN_601_ZN_1079
2djr_A_ZN_201_ZN_1097
2dlk_A_ZN_201_ZN_1207
2dlk_A_ZN_401_ZN_1208
2dlq_A_ZN_200_ZN_1965
2dlq_A_ZN_300_ZN_1966
2dlq_A_ZN_400_ZN_1967
2dlq_A_ZN_500_ZN_1968
2dmd_A_ZN_191_ZN_1432
2dmd_A_ZN_241_ZN_1433
2dmd_A_ZN_291_ZN_1434
2drp_A_ZN_171_ZN_2616
2drp_A_ZN_172_ZN_2617
2drp_D_ZN_173_ZN_2618
2drp_D_ZN_174_ZN_2619
2e72_A_ZN_201_ZN_686
2ebt_A_ZN_100_ZN_1590
2ebt_A_ZN_200_ZN_1591
2ebt_A_ZN_300_ZN_1592
2ee8_A_ZN_301_ZN_1687
2ee8_A_ZN_501_ZN_1688
2ee8_A_ZN_701_ZN_1689
2ej4_A_ZN_201_ZN_1455
2ej4_A_ZN_401_ZN_1456
2el4_A_ZN_200_ZN_636
2el5_A_ZN_200_ZN_571
2el6_A_ZN_200_ZN_666

2elm_A_ZN_181_ZN_569
2eln_A_ZN_181_ZN_554
2elo_A_ZN_181_ZN_544
2elp_A_ZN_181_ZN_558
2elq_A_ZN_181_ZN_539
2elr_A_ZN_181_ZN_524
2els_A_ZN_181_ZN_569
2elt_A_ZN_181_ZN_531
2elu_A_ZN_181_ZN_582
2elv_A_ZN_181_ZN_568
2elw_A_ZN_181_ZN_589
2elx_A_ZN_181_ZN_523
2ely_A_ZN_200_ZN_642
2elz_A_ZN_200_ZN_662
2em0_A_ZN_200_ZN_645
2em1_A_ZN_201_ZN_609
2em2_A_ZN_201_ZN_654
2em3_A_ZN_201_ZN_642
2em4_A_ZN_201_ZN_647
2em5_A_ZN_201_ZN_640
2em6_A_ZN_201_ZN_635
2em7_A_ZN_201_ZN_649
2em8_A_ZN_201_ZN_668
2em9_A_ZN_201_ZN_652
2ema_A_ZN_201_ZN_655
2emb_A_ZN_201_ZN_659
2emc_A_ZN_201_ZN_631
2eme_A_ZN_201_ZN_633
2emf_A_ZN_201_ZN_634
2emg_A_ZN_201_ZN_646
2emh_A_ZN_201_ZN_650
2emi_A_ZN_201_ZN_652
2emj_A_ZN_201_ZN_618
2emk_A_ZN_201_ZN_643
2eml_A_ZN_201_ZN_638
2emm_A_ZN_201_ZN_645
2emp_A_ZN_201_ZN_622
2emv_A_ZN_201_ZN_619
2emw_A_ZN_201_ZN_618
2emx_A_ZN_201_ZN_603
2emy_A_ZN_201_ZN_640
2emz_A_ZN_201_ZN_644
2en0_A_ZN_201_ZN_587
2en1_A_ZN_201_ZN_645
2en2_A_ZN_201_ZN_577
2en3_A_ZN_201_ZN_648
2en4_A_ZN_201_ZN_675
2en6_A_ZN_181_ZN_618
2en7_A_ZN_181_ZN_615
2en8_A_ZN_181_ZN_632
2en9_A_ZN_181_ZN_656
2ena_A_ZN_181_ZN_654
2enc_A_ZN_181_ZN_620
2ene_A_ZN_181_ZN_664
2enf_A_ZN_181_ZN_645
2enh_A_ZN_181_ZN_636
2ent_A_ZN_200_ZN_674
2eoe_A_ZN_201_ZN_640

2eof_A_ZN_201_ZN_606
2eog_A_ZN_201_ZN_620
2eoh_A_ZN_201_ZN_673
2eoi_A_ZN_201_ZN_630
2eoj_A_ZN_201_ZN_592
2eok_A_ZN_201_ZN_570
2eol_A_ZN_201_ZN_578
2eom_A_ZN_201_ZN_651
2eon_A_ZN_201_ZN_635
2eoo_A_ZN_201_ZN_659
2eop_A_ZN_201_ZN_636
2eoq_A_ZN_201_ZN_640
2eor_A_ZN_201_ZN_637
2eos_A_ZN_201_ZN_600
2eou_A_ZN_201_ZN_591
2eov_A_ZN_201_ZN_664
2eow_A_ZN_201_ZN_623
2eox_A_ZN_201_ZN_644
2eoy_A_ZN_201_ZN_685
2eoz_A_ZN_201_ZN_636
2ep0_A_ZN_201_ZN_650
2ep1_A_ZN_201_ZN_640
2ep2_A_ZN_201_ZN_643
2ep3_A_ZN_201_ZN_641
2epa_A_ZN_300_ZN_1108
2epa_A_ZN_400_ZN_1109
2epc_A_ZN_201_ZN_547
2epp_A_ZN_201_ZN_937
2epq_A_ZN_201_ZN_644
2epr_A_ZN_201_ZN_711
2eps_A_ZN_201_ZN_769
2ept_A_ZN_201_ZN_579
2epu_A_ZN_201_ZN_626
2epv_A_ZN_201_ZN_610
2epw_A_ZN_201_ZN_649
2epx_A_ZN_201_ZN_689
2epy_A_ZN_201_ZN_591
2epz_A_ZN_201_ZN_631
2eq0_A_ZN_201_ZN_664
2eq1_A_ZN_201_ZN_632
2eq2_A_ZN_201_ZN_629
2eq3_A_ZN_201_ZN_625
2eq4_A_ZN_201_ZN_648
2eqw_A_ZN_201_ZN_598
2ghf_A_ZN_205_ZN_1638
2ghf_A_ZN_305_ZN_1639
2gqj_A_ZN_200_ZN_1424
2gqj_A_ZN_300_ZN_1425
2hgh_A_ZN_191_ZN_3166
2hgh_A_ZN_192_ZN_3167
2hgh_A_ZN_193_ZN_3168
2i13_A_ZN_501_ZN_4113
2i13_A_ZN_502_ZN_4114
2i13_A_ZN_503_ZN_4115
2i13_A_ZN_504_ZN_4116
2i13_A_ZN_505_ZN_4117
2i13_A_ZN_506_ZN_4118
2i13_B_ZN_507_ZN_4119

2i13_B_ZN_508_ZN_4120
2i13_B_ZN_509_ZN_4121
2i13_B_ZN_510_ZN_4122
2i13_B_ZN_512_ZN_4123
2i5o_A_ZN_336_ZN_526
2j7j_A_ZN_1086_ZN_717
2j7j_A_ZN_1087_ZN_718
2j7j_A_ZN_1088_ZN_719
2jp9_A_ZN_131_ZN_3069
2jp9_A_ZN_132_ZN_3070
2jp9_A_ZN_133_ZN_3071
2jp9_A_ZN_134_ZN_3072
2jpa_A_ZN_131_ZN_2880
2jpa_A_ZN_132_ZN_2881
2jpa_A_ZN_133_ZN_2882
2jpa_A_ZN_134_ZN_2883
2jvx_A_ZN_29_ZN_424
2jvy_A_ZN_29_ZN_434
2kmk_A_ZN_83_ZN_2321
2kmk_A_ZN_84_ZN_2322
2kmk_A_ZN_85_ZN_2323
2kvf_A_ZN_83_ZN_461
2kvg_A_ZN_85_ZN_391
2kvh_A_ZN_84_ZN_431
2prt_A_ZN_201_ZN_1513
2prt_A_ZN_202_ZN_1514
2prt_A_ZN_203_ZN_1515
2prt_A_ZN_204_ZN_1516
2vrd_A_ZN_1062_ZN_993
2vy4_A_ZN_1088_ZN_579
2wbs_A_ZN_1484_ZN_1012
2wbs_A_ZN_1485_ZN_1013
2wbs_A_ZN_1486_ZN_1014
2wbt_A_ZN_1131_ZN_1926
2wbt_B_ZN_1130_ZN_1928
2wbu_A_ZN_1484_ZN_1116
2wbu_A_ZN_1485_ZN_1117
2wbu_A_ZN_1486_ZN_1118
2yrh_A_ZN_200_ZN_622
2yrj_A_ZN_200_ZN_635
2yrk_A_ZN_201_ZN_820
2yrm_A_ZN_201_ZN_585
2yso_A_ZN_181_ZN_666
2ysp_A_ZN_181_ZN_649
2ysv_A_ZN_201_ZN_574
2yt9_A_ZN_201_ZN_1427
2yt9_A_ZN_202_ZN_1428
2yt9_A_ZN_203_ZN_1429
2yta_A_ZN_201_ZN_562
2ytb_A_ZN_301_ZN_575
2ytd_A_ZN_201_ZN_663
2yte_A_ZN_201_ZN_596
2ytf_A_ZN_201_ZN_640
2ytg_A_ZN_201_ZN_646
2yth_A_ZN_201_ZN_640
2yti_A_ZN_201_ZN_639
2ytj_A_ZN_201_ZN_667
2ytk_A_ZN_201_ZN_647

2ytm_A_ZN_181_ZN_621
2ytn_A_ZN_201_ZN_646
2yto_A_ZN_201_ZN_634
2ytp_A_ZN_181_ZN_648
2ytq_A_ZN_201_ZN_620
2ytr_A_ZN_201_ZN_645
2yts_A_ZN_201_ZN_665
2ytt_A_ZN_181_ZN_649
2yu5_A_ZN_201_ZN_640
2yu8_A_ZN_201_ZN_645
3iuf_A_ZN_1_ZN_265
3m99_B_ZN_100_ZN_5519
3m99_D_ZN_105_ZN_5520
3mhh_C_ZN_100_ZN_5737
3mhh_E_ZN_97_ZN_5738
3mhs_C_ZN_100_ZN_6617
3mhs_E_ZN_97_ZN_6618
3mjh_B_ZN_70_ZN_3176
3mjh_D_ZN_70_ZN_3210
4znf_A_ZN_31_ZN_503
5znf_A_ZN_31_ZN_501
7znf_A_ZN_31_ZN_511

------- SF_218 --------
1e31_A_ZN_801_ZN_2236
1e31_B_ZN_801_ZN_2238
1f3h_A_ZN_341_ZN_2216
1f3h_B_ZN_342_ZN_2227
1g73_B_ZN_501_ZN_4013
1g73_D_ZN_502_ZN_4014
1i3o_E_ZN_1_ZN_5585
1i3o_F_ZN_2_ZN_5586
1jd4_A_ZN_501_ZN_1587
1jd4_B_ZN_502_ZN_1588
1m4m_A_ZN_501_ZN_930
1m4m_A_ZN_502_ZN_931
1oxn_A_ZN_1001_ZN_3985
1oxn_B_ZN_1002_ZN_3986
1oxn_C_ZN_1003_ZN_3987
1oxn_D_ZN_1004_ZN_3988
1oxn_E_ZN_1005_ZN_4011
1oxq_A_ZN_1001_ZN_3986
1oxq_B_ZN_1002_ZN_3987
1oxq_C_ZN_1003_ZN_3988
1oxq_D_ZN_1004_ZN_3989
1oxq_E_ZN_1005_ZN_4012
1oy7_A_ZN_1001_ZN_3987
1oy7_B_ZN_1002_ZN_3988
1oy7_C_ZN_1003_ZN_3989
1oy7_D_ZN_1004_ZN_3990
1oy7_E_ZN_1005_ZN_4013
1q4q_A_ZN_604_ZN_8620
1q4q_B_ZN_601_ZN_8621
1q4q_C_ZN_602_ZN_8622
1q4q_D_ZN_603_ZN_8623
1q4q_E_ZN_605_ZN_8624
1q4q_F_ZN_606_ZN_8625
1q4q_G_ZN_607_ZN_8626

1q4q_H_ZN_608_ZN_8627
1q4q_I_ZN_609_ZN_8628
1q4q_J_ZN_610_ZN_8629
1qbh_A_ZN_364_ZN_1596
1sdz_A_ZN_155_ZN_863
1se0_A_ZN_201_ZN_864
1tfq_A_ZN_999_ZN_1834
1tft_A_ZN_999_ZN_1834
1tw6_A_ZN_1001_ZN_1567
1tw6_B_ZN_1001_ZN_1568
1xb0_A_ZN_403_ZN_4831
1xb0_B_ZN_503_ZN_4838
1xb0_C_ZN_603_ZN_4842
1xb0_D_ZN_703_ZN_4846
1xb0_E_ZN_803_ZN_4850
1xb0_F_ZN_903_ZN_4854
1xb1_A_ZN_403_ZN_4809
1xb1_C_ZN_603_ZN_4818
1xb1_D_ZN_703_ZN_4821
1xb1_E_ZN_803_ZN_4825
1xb1_F_ZN_903_ZN_4829
1xox_A_ZN_999_ZN_3710
1xox_B_ZN_998_ZN_3709
2i3h_A_ZN_1001_ZN_1567
2i3h_B_ZN_1001_ZN_1568
2i3i_A_ZN_1001_ZN_1497
2i3i_B_ZN_1001_ZN_1533
2jk7_A_ZN_1347_ZN_822
2opy_A_ZN_102_ZN_868
2opz_A_ZN_501_ZN_3685
2opz_B_ZN_502_ZN_3686
2opz_C_ZN_503_ZN_3687
2opz_D_ZN_504_ZN_3688
2poi_A_ZN_100_ZN_620
2pop_B_ZN_1500_ZN_6267
2pop_D_ZN_3500_ZN_6268
2qfa_A_ZN_143_ZN_2093
2qra_A_ZN_100_ZN_2516
2qra_B_ZN_1100_ZN_2515
2qra_C_ZN_2100_ZN_2514
2qra_D_ZN_3100_ZN_2513
2rax_A_ZN_341_ZN_4283
2rax_E_ZN_341_ZN_4284
2rax_X_ZN_341_ZN_4285
2uvl_A_ZN_1336_ZN_1560
2uvl_B_ZN_1338_ZN_1567
2vm5_A_ZN_1245_ZN_860
2vsl_A_ZN_1347_ZN_825
3clx_A_ZN_502_ZN_3303
3clx_B_ZN_502_ZN_3340
3clx_C_ZN_502_ZN_3413
3clx_D_ZN_502_ZN_3266
3cm2_A_ZN_502_ZN_8392
3cm2_B_ZN_502_ZN_8429
3cm2_C_ZN_502_ZN_8466
3cm2_D_ZN_502_ZN_8355
3cm2_E_ZN_502_ZN_8503
3cm2_F_ZN_502_ZN_8540

3cm2_G_ZN_502_ZN_8577
3cm2_H_ZN_502_ZN_8614
3cm2_I_ZN_502_ZN_8651
3cm2_J_ZN_502_ZN_8688
3cm7_A_ZN_6_ZN_3373
3cm7_B_ZN_5_ZN_3410
3cm7_C_ZN_4_ZN_3336
3cm7_D_ZN_3_ZN_3519
3d9t_A_ZN_501_ZN_1644
3d9t_B_ZN_501_ZN_1645
3d9u_A_ZN_501_ZN_794
3eyl_A_ZN_502_ZN_1653
3eyl_B_ZN_502_ZN_1691
3f7g_A_ZN_1001_ZN_3958
3f7g_B_ZN_1001_ZN_3959
3f7g_C_ZN_1001_ZN_3960
3f7g_D_ZN_1001_ZN_3961
3f7g_E_ZN_1001_ZN_4018
3f7h_A_ZN_1001_ZN_1482
3f7h_B_ZN_1001_ZN_1521
3f7i_A_ZN_1001_ZN_1482
3f7i_B_ZN_1001_ZN_1518
3g76_A_ZN_502_ZN_6542
3g76_B_ZN_502_ZN_6611
3g76_C_ZN_502_ZN_6680
3g76_D_ZN_502_ZN_6749
3g76_E_ZN_502_ZN_6818
3g76_F_ZN_502_ZN_6887
3g76_G_ZN_502_ZN_6956
3g76_H_ZN_502_ZN_7025
3gt9_A_ZN_1001_ZN_1449
3gt9_B_ZN_1001_ZN_1486
3gta_A_ZN_1001_ZN_1472
3gta_B_ZN_1001_ZN_1509
3hl5_A_ZN_502_ZN_1509
3hl5_B_ZN_502_ZN_1547
3m0a_D_ZN_401_ZN_1894
3m0d_D_ZN_401_ZN_2051
3m1d_A_ZN_1000_ZN_1249
3m1d_B_ZN_1000_ZN_1250
3mup_A_ZN_1_ZN_3375
3mup_B_ZN_2_ZN_3413
3mup_C_ZN_3_ZN_3451
3mup_D_ZN_4_ZN_3489
3oz1_A_ZN_501_ZN_3391
3oz1_B_ZN_501_ZN_3437
3oz1_C_ZN_501_ZN_3483
3oz1_D_ZN_501_ZN_3529

------- SF_219 --------
2csv_A_ZN_400_ZN_1067
2d8u_A_ZN_401_ZN_911
2d8v_A_ZN_401_ZN_950
2did_A_ZN_401_ZN_735
2dif_A_ZN_401_ZN_735
2dja_A_ZN_401_ZN_1266
2dq5_A_ZN_1187_ZN_731
2kwq_A_ZN_843_ZN_1473

3ddt_A_ZN_47_ZN_1056
3ddt_B_ZN_47_ZN_1058
3ddt_C_ZN_47_ZN_1060

------- SF_220 --------
1e0e_A_ZN_147_ZN_1495
1e0e_B_ZN_147_ZN_1496
1k6y_A_ZN_401_ZN_5929
1k6y_B_ZN_402_ZN_5936
1k6y_C_ZN_403_ZN_5943
1k6y_D_ZN_404_ZN_5950
1wja_A_ZN_56_ZN_1463
1wja_B_ZN_56_ZN_1464
1wjb_A_ZN_56_ZN_1687
1wjb_B_ZN_56_ZN_1688
1wjc_A_ZN_56_ZN_1463
1wjc_B_ZN_56_ZN_1464
1wjd_A_ZN_56_ZN_1687
1wjd_B_ZN_56_ZN_1688
3f9k_A_ZN_210_ZN_46729
3f9k_B_ZN_210_ZN_46731
3f9k_c_ZN_210_ZN_46757
3f9k_d_ZN_210_ZN_46759
3f9k_E_ZN_210_ZN_46733
3f9k_F_ZN_210_ZN_46735
3f9k_g_ZN_210_ZN_46761
3f9k_h_ZN_210_ZN_46763
3f9k_I_ZN_210_ZN_46737
3f9k_J_ZN_210_ZN_46739
3f9k_k_ZN_210_ZN_46765
3f9k_l_ZN_210_ZN_46767
3f9k_M_ZN_210_ZN_46741
3f9k_N_ZN_210_ZN_46743
3f9k_o_ZN_210_ZN_46769
3f9k_p_ZN_210_ZN_46771
3f9k_Q_ZN_210_ZN_46745
3f9k_R_ZN_210_ZN_46747
3f9k_s_ZN_210_ZN_46773
3f9k_t_ZN_210_ZN_46775
3f9k_U_ZN_210_ZN_46749
3f9k_V_ZN_210_ZN_46751
3f9k_Y_ZN_210_ZN_46753
3f9k_Z_ZN_210_ZN_46755
3hpg_A_ZN_220_ZN_12799
3hpg_B_ZN_220_ZN_12800
3hpg_C_ZN_220_ZN_12801
3hpg_D_ZN_220_ZN_12802
3hpg_E_ZN_220_ZN_12803
3hpg_F_ZN_220_ZN_12804
3hph_A_ZN_220_ZN_8634
3hph_B_ZN_220_ZN_8647
3hph_C_ZN_220_ZN_8653
3hph_D_ZN_220_ZN_8660
3l2q_A_ZN_393_ZN_4886
3l2r_A_ZN_393_ZN_4887
3l2u_A_ZN_393_ZN_4889
3l2v_A_ZN_393_ZN_4863
3l2w_A_ZN_393_ZN_4888

3nnq_A_ZN_201_ZN_1577
3nnq_B_ZN_201_ZN_1582
3os0_A_ZN_393_ZN_5428
3os1_A_ZN_393_ZN_5227
3os2_A_ZN_393_ZN_5228
3oy9_A_ZN_393_ZN_5076
3oya_A_ZN_393_ZN_5099
3oyb_A_ZN_393_ZN_5080
3oyc_A_ZN_393_ZN_5104
3oyd_A_ZN_393_ZN_5129
3oye_A_ZN_393_ZN_5107
3oyf_A_ZN_393_ZN_5093
3oyg_A_ZN_393_ZN_5110
3oyh_A_ZN_393_ZN_5080
3oyi_A_ZN_393_ZN_5069
3oyj_A_ZN_393_ZN_5082
3oyk_A_ZN_393_ZN_5056
3oyl_A_ZN_393_ZN_5083
3oym_A_ZN_393_ZN_5172
3oyn_A_ZN_393_ZN_5109

------- SF_221 --------
1fb1_A_ZN_300_ZN_7731
1fb1_B_ZN_300_ZN_7732
1fb1_C_ZN_300_ZN_7733
1fb1_D_ZN_300_ZN_7734
1fb1_E_ZN_300_ZN_7735
1fbx_A_ZN_3316_ZN_25996
1fbx_B_ZN_3317_ZN_25999
1fbx_C_ZN_3318_ZN_26001
1fbx_D_ZN_3319_ZN_26003
1fbx_E_ZN_3320_ZN_26005
1fbx_F_ZN_3321_ZN_26006
1fbx_G_ZN_3322_ZN_26009
1fbx_H_ZN_3323_ZN_26011
1fbx_I_ZN_3324_ZN_26013
1fbx_J_ZN_3325_ZN_26015
1fbx_K_ZN_3326_ZN_26016
1fbx_L_ZN_3327_ZN_26019
1fbx_M_ZN_3328_ZN_26021
1fbx_N_ZN_3329_ZN_26023
1fbx_O_ZN_3330_ZN_26025
1is8_A_ZN_3102_ZN_22021
1is8_B_ZN_3109_ZN_22022
1is8_C_ZN_3107_ZN_22023
1is8_D_ZN_3108_ZN_22024
1is8_E_ZN_3105_ZN_22025
1is8_F_ZN_3110_ZN_22026
1is8_G_ZN_3103_ZN_22027
1is8_H_ZN_3104_ZN_22028
1is8_I_ZN_3101_ZN_22029
1is8_J_ZN_3106_ZN_22030
1wm9_A_ZN_1001_ZN_7332
1wm9_B_ZN_1002_ZN_7333
1wm9_C_ZN_1003_ZN_7334
1wm9_D_ZN_1004_ZN_7335
1wm9_E_ZN_1005_ZN_7336
1wpl_A_ZN_1110_ZN_22121

1wpl_B_ZN_1108_ZN_22169
1wpl_C_ZN_1103_ZN_22200
1wpl_D_ZN_1104_ZN_22231
1wpl_E_ZN_1102_ZN_22262
1wpl_F_ZN_1105_ZN_22276
1wpl_G_ZN_1101_ZN_22324
1wpl_H_ZN_1107_ZN_22355
1wpl_I_ZN_1109_ZN_22386
1wpl_J_ZN_1106_ZN_22417
1wuq_A_ZN_1001_ZN_7296
1wuq_B_ZN_1002_ZN_7363
1wuq_C_ZN_1003_ZN_7364
1wuq_D_ZN_1004_ZN_7431
1wuq_E_ZN_1005_ZN_7465
1wur_A_ZN_1001_ZN_7332
1wur_B_ZN_1002_ZN_7397
1wur_C_ZN_1003_ZN_7398
1wur_D_ZN_1004_ZN_7431
1wur_E_ZN_1005_ZN_7496

------- SF_222 --------
2dip_A_ZN_401_ZN_1515
2e5r_A_ZN_401_ZN_937
2fc7_A_ZN_401_ZN_1167
3nih_A_ZN_3_ZN_647
3nii_A_ZN_3_ZN_645
3nij_A_ZN_3_ZN_646
3nik_A_ZN_3_ZN_2581
3nik_B_ZN_3_ZN_2584
3nik_D_ZN_3_ZN_2587
3nik_F_ZN_3_ZN_2590
3nil_A_ZN_3_ZN_2554
3nil_B_ZN_3_ZN_2557
3nil_D_ZN_3_ZN_2560
3nil_F_ZN_3_ZN_2563
3nim_A_ZN_3_ZN_2579
3nim_B_ZN_3_ZN_2582
3nim_D_ZN_3_ZN_2585
3nim_F_ZN_3_ZN_2588
3nin_A_ZN_3_ZN_1318
3nin_B_ZN_3_ZN_1321
3nis_A_ZN_3_ZN_2549
3nis_B_ZN_3_ZN_2556
3nis_D_ZN_3_ZN_2563
3nis_F_ZN_3_ZN_2566
3nit_A_ZN_3_ZN_694
3ny1_A_ZN_6_ZN_1096
3ny1_B_ZN_3_ZN_1099
3ny2_A_ZN_3_ZN_4308
3ny2_B_ZN_6_ZN_4311
3ny2_C_ZN_9_ZN_4314
3ny2_D_ZN_12_ZN_4317
3ny2_E_ZN_15_ZN_4320
3ny2_F_ZN_18_ZN_4323
3ny2_G_ZN_21_ZN_4326
3ny2_H_ZN_24_ZN_4329
3ny3_A_ZN_3_ZN_581

------- SF_223 --------
2i50_A_ZN_337_ZN_1927
2ida_A_ZN_104_ZN_1552
2uzg_A_ZN_133_ZN_1478
3c5k_A_ZN_201_ZN_842
3gv4_A_ZN_201_ZN_802
3m99_A_ZN_474_ZN_5516
3mhh_A_ZN_474_ZN_5734
3mhs_A_ZN_474_ZN_6613

------- SF_224 --------
1k2f_A_ZN_601_ZN_2999
1k2f_A_ZN_605_ZN_3002
1k2f_B_ZN_602_ZN_3015
1k2f_B_ZN_606_ZN_3016
2a25_A_ZN_601_ZN_1222
2an6_A_ZN_601_ZN_6373
2an6_A_ZN_602_ZN_6374
2an6_B_ZN_603_ZN_6375
2an6_B_ZN_604_ZN_6376
2an6_C_ZN_605_ZN_6377
2an6_C_ZN_606_ZN_6378
2an6_D_ZN_607_ZN_6379
2an6_D_ZN_608_ZN_6380
2eod_A_ZN_300_ZN_962
2eod_A_ZN_400_ZN_963
2yre_A_ZN_601_ZN_1429
2yre_A_ZN_701_ZN_1430
2yuc_A_ZN_201_ZN_1090
2yuc_A_ZN_401_ZN_1091

------- SF_225 --------
2wbt_A_ZN_1130_ZN_1925
2wbt_B_ZN_1129_ZN_1927
3hcs_A_ZN_303_ZN_2523
3hcs_A_ZN_304_ZN_2524
3hcs_A_ZN_305_ZN_2525
3hcs_B_ZN_308_ZN_2528
3hcs_B_ZN_309_ZN_2529
3hcs_B_ZN_310_ZN_2530
3hct_A_ZN_303_ZN_2021
3hcu_A_ZN_303_ZN_4066
3knv_A_ZN_203_ZN_944

------- SF_226 --------
1pi1_A_ZN_196_ZN_1517
2hjn_A_ZN_315_ZN_1660

------- SF_227 --------
1zw8_A_ZN_66_ZN_1000
1zw8_A_ZN_67_ZN_1001
2rpc_A_ZN_201_ZN_2414
2rpc_A_ZN_401_ZN_2415
2rpc_A_ZN_601_ZN_2416
2rpc_A_ZN_801_ZN_2417

------- SF_228 --------
2q1z_B_ZN_196_ZN_5180,2q1z_B_ZN_196_ZN_5190

2q1z_D_ZN_196_ZN_5192,2q1z_D_ZN_197_ZN_5183
2z2s_B_ZN_204_ZN_9993
2z2s_D_ZN_204_ZN_9994
2z2s_F_ZN_204_ZN_10000
2z2s_H_ZN_204_ZN_10006
3hug_B_ZN_109_ZN_10473
3hug_D_ZN_109_ZN_10479
3hug_F_ZN_109_ZN_10480
3hug_H_ZN_109_ZN_10486
3hug_J_ZN_109_ZN_10497
3hug_L_ZN_110_ZN_10503
3hug_N_ZN_109_ZN_10509
3hug_P_ZN_110_ZN_10515
3hug_R_ZN_109_ZN_10521
3hug_T_ZN_109_ZN_10527

------- SF_229 --------
3eph_A_ZN_1_ZN_9627
3eph_B_ZN_1_ZN_9643
3epj_A_ZN_1_ZN_9627
3epj_B_ZN_1_ZN_9637
3epk_A_ZN_1_ZN_9627
3epk_B_ZN_1_ZN_9650
3epl_A_ZN_1_ZN_9634
3epl_B_ZN_1_ZN_9645
3h1w_A_ZN_397_ZN_2989

------- SF_230 --------
1ej6_@_ZN_2000_ZN_34492

------- SF_231 --------
2bai_A_ZN_110_ZN_496

------- SF_232 --------
2dkt_A_ZN_241_ZN_2165
2k2c_A_ZN_138_ZN_2124

------- SF_233 --------
2dmi_A_ZN_200_ZN_1766
2dmi_A_ZN_300_ZN_1767

------- SF_234 --------
2giv_A_ZN_501_ZN_2108
2ou2_A_ZN_490_ZN_2048
2ozu_A_ZN_800_ZN_2141
2pq8_A_ZN_501_ZN_2125
2rc4_A_ZN_1_ZN_2095

------- SF_235 --------
2k9h_A_ZN_101_ZN_842
2k9h_A_ZN_102_ZN_843

------- SF_236 --------
2iyk_A_ZN_1164_ZN_2456
2iyk_B_ZN_1164_ZN_2459
2wjv_A_ZN_2_ZN_13072
2wjv_B_ZN_2_ZN_13140
2wjy_A_ZN_2_ZN_6171

------- SF_237 --------
1faq_A_ZN_2_ZN_847
1far_A_ZN_2_ZN_847
1kbe_A_ZN_1_ZN_778
1kbf_A_ZN_1_ZN_778
1ptq_A_ZN_1_ZN_404
1ptr_A_ZN_1_ZN_389
1r79_A_ZN_201_ZN_1209
1rfh_A_ZN_2_ZN_924,1rfh_A_ZN_2_ZN_925
1tbn_A_ZN_1_ZN_1026
1tbo_A_ZN_1_ZN_1026,1tbo_A_ZN_1_ZN_1030
1v5n_A_ZN_401_ZN_1358
1xa6_A_ZN_467_ZN_3256
1y8f_A_ZN_701_ZN_773
2db6_A_ZN_201_ZN_1145
2e73_A_ZN_401_ZN_1184
2eli_A_ZN_201_ZN_1259
2enn_A_ZN_300_ZN_1167
2enz_A_ZN_300_ZN_973
2fnf_X_ZN_2_ZN_923
2row_A_ZN_601_ZN_1376
2vrw_B_ZN_1565_ZN_4413
2yuu_A_ZN_201_ZN_1235
3bji_A_ZN_4_ZN_8365
3bji_B_ZN_2_ZN_8366
3cxl_A_ZN_500_ZN_3169
3ky9_A_ZN_902_ZN_8813
3ky9_B_ZN_902_ZN_8815

------- SF_238 --------
1i3q_A_ZN_2008_ZN_28170
1i50_A_ZN_3008_ZN_28291
1i6h_A_ZN_1735_ZN_28441
1k83_A_ZN_3008_ZN_27837
1nik_A_ZN_1735_ZN_28302
1r5u_A_ZN_1735_ZN_28303
1r9s_A_ZN_1735_ZN_28466
1r9t_A_ZN_1735_ZN_29222
1sfo_A_ZN_1735_ZN_28652
1twa_A_ZN_3008_ZN_27736
1twc_A_ZN_3008_ZN_27718
1twf_A_ZN_3008_ZN_28297
1twg_A_ZN_3008_ZN_27701
1twh_A_ZN_3008_ZN_27707
1wcm_A_ZN_2457_ZN_30950
1y1v_A_ZN_1735_ZN_31809
1y1w_A_ZN_1735_ZN_31810
1y77_A_ZN_1735_ZN_31811
2b63_A_ZN_2457_ZN_31737
2b8k_A_ZN_1735_ZN_31046
2e2h_A_ZN_1735_ZN_28976
2e2i_A_ZN_1735_ZN_29666
2e2j_A_ZN_1735_ZN_29179
2ja5_A_ZN_2465_ZN_31674
2ja6_A_ZN_2465_ZN_32026
2ja7_A_ZN_2472_ZN_63952
2ja7_M_ZN_2458_ZN_63954

2ja8_A_ZN_2465_ZN_32015
2nvq_A_ZN_1735_ZN_29373
2nvt_A_ZN_1735_ZN_29141
2nvx_A_ZN_1735_ZN_29386
2nvy_A_ZN_3008_ZN_28297
2nvz_A_ZN_1735_ZN_28978
2pmz_A_ZN_1002_ZN_48121
2pmz_Q_ZN_1002_ZN_48126
2r7z_A_ZN_1735_ZN_31812
2r92_A_ZN_1508_ZN_31618
2r93_A_ZN_1508_ZN_31506
2vum_A_ZN_2457_ZN_32092
2waq_A_ZN_1300_ZN_26468
2waq_B_ZN_1300_ZN_26472
2waq_N_ZN_100_ZN_26483
2wb1_A_ZN_1880_ZN_52739
2wb1_W_ZN_1880_ZN_52761
2yu9_A_ZN_1735_ZN_29477
3cqz_A_ZN_3008_ZN_27328
3fki_A_ZN_1735_ZN_31417
3gtg_A_ZN_1735_ZN_30072
3gtj_A_ZN_1735_ZN_29980
3gtk_A_ZN_1735_ZN_30118
3gtl_A_ZN_1735_ZN_29262
3gtm_A_ZN_1736_ZN_30567
3gto_A_ZN_1735_ZN_29265
3gtp_A_ZN_1735_ZN_29285
3gtq_A_ZN_1735_ZN_28631
3h0g_A_ZN_2457_ZN_62878
3h0g_M_ZN_2457_ZN_62887
3h3v_B_ZN_1735_ZN_31785
3hkz_A_ZN_1001_ZN_53073
3hkz_I_ZN_1001_ZN_53086
3hkz_N_ZN_1001_ZN_53084
3hkz_P_ZN_1001_ZN_53085
3hkz_W_ZN_1001_ZN_53097
3hkz_X_ZN_1001_ZN_53098
3hou_A_ZN_9985_ZN_63680
3hou_M_ZN_9993_ZN_63688
3hov_A_ZN_2457_ZN_31785
3how_A_ZN_2457_ZN_31884
3hox_A_ZN_2457_ZN_31926
3hoy_A_ZN_2457_ZN_31811
3hoz_A_ZN_2457_ZN_31969
3i4m_A_ZN_2457_ZN_32364
3i4n_A_ZN_2457_ZN_32316
3k1f_A_ZN_3008_ZN_32338
3k7a_A_ZN_1735_ZN_29033

------- SF_239 --------
1bor_A_ZN_58_ZN_424
1ldk_C_ZN_4002_ZN_7926
1tot_A_ZN_53_ZN_828
1vyx_A_ZN_1062_ZN_916

------- SF_240 --------
1hw7_A_ZN_260_ZN_1817
1vq0_A_ZN_300_ZN_4577

1vq0_B_ZN_300_ZN_4596
1vzy_A_ZN_1291_ZN_4385
1vzy_B_ZN_1287_ZN_4402
1xjh_A_ZN_63_ZN_928

------- SF_241 --------
2fpr_A_ZN_502_ZN_2536
2fpr_B_ZN_501_ZN_2541
2fps_A_ZN_502_ZN_2571
2fps_B_ZN_501_ZN_2574
2fpu_A_ZN_506_ZN_2549
2fpu_B_ZN_505_ZN_2552
2fpw_A_ZN_502_ZN_2568
2fpw_B_ZN_501_ZN_2570
2fpx_A_ZN_602_ZN_2536
2fpx_B_ZN_601_ZN_2538
2gmw_A_ZN_300_ZN_3050
2gmw_B_ZN_300_ZN_3051
3esq_A_ZN_212_ZN_1425
3esr_A_ZN_212_ZN_1425
3l1u_A_ZN_212_ZN_2860
3l1u_B_ZN_212_ZN_2862
3l1v_A_ZN_212_ZN_2833
3l1v_B_ZN_212_ZN_2840
3l8e_A_ZN_801_ZN_2891
3l8e_B_ZN_802_ZN_2896
3l8f_A_ZN_501_ZN_1435
3l8g_A_ZN_189_ZN_1435
3l8h_A_ZN_901_ZN_5334
3l8h_B_ZN_902_ZN_5344
3l8h_C_ZN_903_ZN_5357
3l8h_D_ZN_904_ZN_5364

------- SF_242 --------
1a1r_A_ZN_901_ZN_3299
1a1r_B_ZN_902_ZN_3300
1bt7_A_ZN_301_ZN_2451
1cu1_A_ZN_999_ZN_9617
1cu1_B_ZN_1999_ZN_9618
1dxp_A_ZN_201_ZN_2739
1dxp_B_ZN_201_ZN_2740
1dxw_A_ZN_301_ZN_2486
1dy9_B_ZN_300_ZN_2811
1jxp_A_ZN_190_ZN_2775
1jxp_B_ZN_490_ZN_2776
1n1l_A_ZN_300_ZN_2737
1n1l_B_ZN_300_ZN_2767
1ns3_A_ZN_190_ZN_2815
1ns3_B_ZN_690_ZN_2816
1rgq_A_ZN_193_ZN_2797
1rgq_B_ZN_193_ZN_2798
1rtl_A_ZN_301_ZN_2737
1rtl_B_ZN_301_ZN_2770
1z8r_A_ZN_151_ZN_2254
2a4g_A_ZN_301_ZN_2729
2a4g_C_ZN_302_ZN_2779
2a4q_A_ZN_301_ZN_2729
2a4q_C_ZN_302_ZN_2788

2a4r_A_ZN_301_ZN_2729
2a4r_C_ZN_302_ZN_2780
2f9u_A_ZN_901_ZN_2780
2f9u_C_ZN_901_ZN_2834
2f9v_A_ZN_901_ZN_2749
2f9v_C_ZN_902_ZN_2800
2fm2_A_ZN_200_ZN_2719
2fm2_C_ZN_201_ZN_2776
2gvf_A_ZN_901_ZN_2750
2gvf_C_ZN_902_ZN_2808
2hrv_A_ZN_143_ZN_2227
2hrv_B_ZN_143_ZN_2228
2k1q_A_ZN_301_ZN_2536
2o8m_A_ZN_201_ZN_2779
2o8m_B_ZN_201_ZN_2781
2obo_A_ZN_901_ZN_2763
2obo_C_ZN_902_ZN_2804
2obq_A_ZN_901_ZN_2723
2obq_C_ZN_902_ZN_2724
2oc0_A_ZN_901_ZN_2729
2oc0_C_ZN_902_ZN_2773
2oc1_A_ZN_901_ZN_2741
2oc1_C_ZN_902_ZN_2787
2oc7_A_ZN_901_ZN_2729
2oc7_C_ZN_901_ZN_2774
2oc8_A_ZN_901_ZN_2759
2oc8_C_ZN_902_ZN_2801
2oin_A_ZN_901_ZN_2688
2oin_B_ZN_902_ZN_2689
2qv1_A_ZN_901_ZN_2695
2qv1_B_ZN_902_ZN_2696
2xcf_A_ZN_400_ZN_2739
2xcf_B_ZN_400_ZN_2787
2xcn_A_ZN_1183_ZN_2776
2xcn_B_ZN_1182_ZN_2819
3eyd_A_ZN_901_ZN_2729
3eyd_C_ZN_901_ZN_2773
3kee_A_ZN_2000_ZN_5653
3kee_B_ZN_2000_ZN_5706
3kee_C_ZN_2000_ZN_5759
3kee_D_ZN_2000_ZN_5824
3kf2_A_ZN_901_ZN_2745
3kf2_B_ZN_902_ZN_2746
3kn2_A_ZN_901_ZN_2729
3kn2_C_ZN_902_ZN_2782
3knx_A_ZN_901_ZN_2729
3knx_C_ZN_902_ZN_2788
3m5l_A_ZN_1_ZN_1514
3m5m_A_ZN_1184_ZN_2967
3m5m_B_ZN_2_ZN_2968
3m5n_A_ZN_1181_ZN_5864
3m5n_B_ZN_1181_ZN_5870
3m5n_C_ZN_1181_ZN_5876
3m5n_D_ZN_1181_ZN_5882
3m5o_A_ZN_1183_ZN_3066
3m5o_B_ZN_2_ZN_3067
3o8b_A_ZN_800_ZN_9617
3o8b_B_ZN_800_ZN_9628

3o8c_A_ZN_800_ZN_9740
3o8c_B_ZN_800_ZN_9746
3o8r_A_ZN_800_ZN_9740
3o8r_B_ZN_800_ZN_9773
3oyp_A_ZN_189_ZN_2777
3oyp_B_ZN_189_ZN_2821

------- SF_243 --------
1q68_A_ZN_201_ZN_1112
1q69_A_ZN_207_ZN_768

------- SF_244 --------
1wj2_A_ZN_470_ZN_1123
2ayd_A_ZN_369_ZN_621
2rpr_A_ZN_201_ZN_1400

------- SF_245 --------
2fyg_A_ZN_303_ZN_964
2g9t_A_ZN_999_ZN_20941
2g9t_B_ZN_999_ZN_20943
2g9t_C_ZN_999_ZN_20945
2g9t_D_ZN_999_ZN_20947
2g9t_E_ZN_999_ZN_20949
2g9t_F_ZN_999_ZN_20951
2g9t_G_ZN_999_ZN_20953
2g9t_H_ZN_999_ZN_20955
2g9t_I_ZN_999_ZN_20957
2g9t_J_ZN_999_ZN_20959
2g9t_K_ZN_999_ZN_20961
2g9t_L_ZN_999_ZN_20963
2g9t_M_ZN_999_ZN_20965
2g9t_N_ZN_999_ZN_20967
2g9t_O_ZN_999_ZN_20969
2g9t_P_ZN_999_ZN_20971
2g9t_Q_ZN_999_ZN_20973
2g9t_R_ZN_999_ZN_20975
2g9t_S_ZN_999_ZN_20977
2g9t_T_ZN_999_ZN_20979
2g9t_U_ZN_999_ZN_20981
2g9t_V_ZN_999_ZN_20983
2g9t_W_ZN_999_ZN_20985
2g9t_X_ZN_999_ZN_20987
2ga6_A_ZN_999_ZN_20992
2ga6_B_ZN_999_ZN_20994
2ga6_C_ZN_999_ZN_20996
2ga6_D_ZN_999_ZN_20998
2ga6_E_ZN_999_ZN_21000
2ga6_F_ZN_999_ZN_21002
2ga6_G_ZN_999_ZN_21004
2ga6_H_ZN_999_ZN_21006
2ga6_I_ZN_999_ZN_21008
2ga6_J_ZN_999_ZN_21010
2ga6_K_ZN_999_ZN_21012
2ga6_L_ZN_999_ZN_21014
2ga6_M_ZN_999_ZN_21016
2ga6_N_ZN_999_ZN_21018
2ga6_O_ZN_999_ZN_21020
2ga6_P_ZN_999_ZN_21022

2ga6_Q_ZN_999_ZN_21024
2ga6_R_ZN_999_ZN_21026
2ga6_S_ZN_999_ZN_21028
2ga6_T_ZN_999_ZN_21030
2ga6_U_ZN_999_ZN_21032
2ga6_V_ZN_999_ZN_21034
2ga6_W_ZN_999_ZN_21036
2ga6_X_ZN_999_ZN_21038
3mp2_A_ZN_1_ZN_1660

------- SF_246 --------
1bnl_A_ZN_179_ZN_5533
1bnl_B_ZN_179_ZN_5534
1bnl_C_ZN_179_ZN_5535
1bnl_D_ZN_179_ZN_5536
1dy0_A_ZN_401_ZN_1402
1dy1_A_ZN_401_ZN_1422

------- SF_247 --------
1fn9_A_ZN_1001_ZN_5767
1fn9_B_ZN_1002_ZN_5768
1jmu_G_ZN_702_ZN_23363
1jmu_H_ZN_703_ZN_23374
1jmu_I_ZN_701_ZN_23385

------- SF_248 --------
1mr1_C_ZN_601_ZN_4671
1mr1_D_ZN_602_ZN_4672

------- SF_249 --------
1odh_A_ZN_1171_ZN_1808

------- SF_250 --------
1occ_F_ZN_99_ZN_28503
1occ_S_ZN_99_ZN_28508
1oco_F_ZN_99_ZN_28586
1oco_S_ZN_99_ZN_28592
1ocr_F_ZN_99_ZN_28706
1ocr_S_ZN_99_ZN_28712
1ocz_F_ZN_99_ZN_28592
1ocz_S_ZN_99_ZN_28604
1v54_F_ZN_99_ZN_29334
1v54_S_ZN_99_ZN_30477
1v55_F_ZN_99_ZN_29397
1v55_S_ZN_99_ZN_30477
2dyr_F_ZN_99_ZN_29367
2dyr_S_ZN_99_ZN_30492
2dys_F_ZN_99_ZN_29397
2dys_S_ZN_99_ZN_30573
2eij_F_ZN_99_ZN_29367
2eij_S_ZN_99_ZN_30543
2eik_F_ZN_99_ZN_29369
2eik_S_ZN_99_ZN_30484
2eil_F_ZN_99_ZN_29368
2eil_S_ZN_99_ZN_30493
2eim_F_ZN_99_ZN_29368
2eim_S_ZN_99_ZN_30516
2ein_F_ZN_99_ZN_29369

2ein_S_ZN_99_ZN_30436
2occ_F_ZN_99_ZN_28640
2occ_S_ZN_99_ZN_28646
2zxw_F_ZN_99_ZN_29116
2zxw_S_ZN_99_ZN_30347
3abk_F_ZN_99_ZN_29435
3abk_S_ZN_99_ZN_30446
3abl_F_ZN_99_ZN_29063
3abl_S_ZN_99_ZN_30347
3abm_F_ZN_99_ZN_29032
3abm_S_ZN_99_ZN_30231
3ag1_F_ZN_99_ZN_29263
3ag1_S_ZN_99_ZN_30408
3ag2_F_ZN_99_ZN_29316
3ag2_S_ZN_99_ZN_30547
3ag3_F_ZN_99_ZN_29298
3ag3_S_ZN_99_ZN_30613
3ag4_F_ZN_99_ZN_29283
3ag4_S_ZN_99_ZN_30441

------- SF_251 --------
2dkt_A_ZN_341_ZN_2167
2dkt_A_ZN_391_ZN_2168
2dkt_A_ZN_441_ZN_2169
2k2c_A_ZN_141_ZN_2127

------- SF_252 --------
3ifu_A_ZN_182_ZN_1370

------- SF_253 --------
1a85_A_ZN_999_ZN_1502
1a86_A_ZN_999_ZN_1284
1af0_A_ZN_472_ZN_3568
1akl_A_ZN_500_ZN_3507
1ast_A_ZN_999_ZN_1593
1atl_A_ZN_401_ZN_3236
1atl_B_ZN_402_ZN_3261
1ayk_A_ZN_170_ZN_2567
1b3d_A_ZN_301_ZN_2725
1b3d_B_ZN_301_ZN_2730
1b8y_A_ZN_301_ZN_1334
1biw_A_ZN_301_ZN_2725
1biw_B_ZN_801_ZN_2730
1bkc_A_ZN_1_ZN_8087
1bkc_C_ZN_1_ZN_8117
1bkc_E_ZN_1_ZN_8147
1bkc_I_ZN_1_ZN_8177
1bm6_A_ZN_256_ZN_2676
1bqb_A_ZN_350_ZN_2356
1bqo_A_ZN_301_ZN_2725
1bqo_B_ZN_301_ZN_2730
1bqq_M_ZN_289_ZN_2839
1bsw_A_ZN_800_ZN_1529
1bud_A_ZN_800_ZN_1530
1buv_M_ZN_1186_ZN_2839
1bzs_A_ZN_999_ZN_1307
1c3i_A_ZN_260_ZN_2731
1c3i_B_ZN_260_ZN_2736

1c7k_A_ZN_133_ZN_1017
1c8t_A_ZN_260_ZN_2653
1c8t_B_ZN_260_ZN_2690
1caq_A_ZN_301_ZN_1341
1cge_A_ZN_301_ZN_1544
1cgf_A_ZN_301_ZN_3097
1cgf_B_ZN_301_ZN_3102
1cgl_A_ZN_301_ZN_3200
1cgl_B_ZN_301_ZN_3284
1ciz_A_ZN_301_ZN_1341
1ck7_A_ZN_990_ZN_4932
1cqr_A_ZN_1301_ZN_2725
1cqr_B_ZN_2301_ZN_2730
1cxv_A_ZN_1_ZN_2571
1cxv_B_ZN_2_ZN_2603
1d5j_A_ZN_301_ZN_2725
1d5j_B_ZN_801_ZN_2754
1d7x_A_ZN_301_ZN_2725
1d7x_B_ZN_801_ZN_2753
1d8f_A_ZN_301_ZN_2725
1d8f_B_ZN_801_ZN_2730
1d8m_A_ZN_301_ZN_2725
1d8m_B_ZN_801_ZN_2730
1dmt_A_ZN_755_ZN_5639
1dth_A_ZN_901_ZN_3185
1dth_B_ZN_902_ZN_3187
1e1h_A_ZN_501_ZN_6465
1e1h_C_ZN_502_ZN_6466
1eak_A_ZN_997_ZN_13362
1eak_B_ZN_997_ZN_13376
1eak_C_ZN_997_ZN_13390
1eak_D_ZN_997_ZN_13399
1eb6_A_ZN_178_ZN_1382
1epw_A_ZN_1291_ZN_10588
1esp_A_ZN_323_ZN_2397
1eub_A_ZN_276_ZN_2631
1ezm_A_ZN_302_ZN_2287
1f31_A_ZN_1291_ZN_10482
1f82_A_ZN_500_ZN_3457
1fbl_A_ZN_998_ZN_2973
1fj3_A_ZN_500_ZN_2460
1fjo_A_ZN_500_ZN_2459
1fjq_A_ZN_501_ZN_2468
1fjt_A_ZN_501_ZN_2463
1fju_A_ZN_501_ZN_2463
1fjv_A_ZN_501_ZN_2456
1fjw_A_ZN_501_ZN_2456
1fls_A_ZN_166_ZN_2430
1fm1_A_ZN_166_ZN_2430
1g05_A_ZN_301_ZN_2649
1g05_B_ZN_801_ZN_2654
1g12_A_ZN_200_ZN_1284
1g49_A_ZN_301_ZN_2725
1g49_B_ZN_801_ZN_2730
1g4k_A_ZN_301_ZN_4021
1g4k_B_ZN_301_ZN_4049
1g4k_C_ZN_301_ZN_4083
1g9a_A_ZN_1291_ZN_10588

1g9b_A_ZN_1291_ZN_10588
1g9c_A_ZN_1291_ZN_10588
1g9d_A_ZN_1291_ZN_10588
1g9k_A_ZN_600_ZN_3495
1ge5_A_ZN_200_ZN_1277
1ge6_A_ZN_200_ZN_1253
1ge7_A_ZN_200_ZN_2556
1ge7_B_ZN_200_ZN_2568
1gkc_A_ZN_1450_ZN_2541
1gkc_B_ZN_1451_ZN_2571
1gkd_A_ZN_1450_ZN_2533
1gkd_B_ZN_1450_ZN_2561
1go7_P_ZN_1485_ZN_3525
1go8_P_ZN_1486_ZN_3515
1gw6_A_ZN_1615_ZN_4911
1gxd_A_ZN_1634_ZN_12940
1gxd_B_ZN_1634_ZN_12948
1gxw_A_ZN_1321_ZN_2455
1h19_A_ZN_701_ZN_4878
1h71_P_ZN_600_ZN_3413
1hfc_A_ZN_275_ZN_1246
1hfs_A_ZN_257_ZN_1273
1hov_A_ZN_166_ZN_2518
1hs6_A_ZN_701_ZN_4878
1htd_A_ZN_401_ZN_3213
1htd_B_ZN_402_ZN_3215
1hv5_A_ZN_5502_ZN_7998
1hv5_B_ZN_5505_ZN_8103
1hv5_C_ZN_5508_ZN_8208
1hv5_D_ZN_5511_ZN_8313
1hv5_E_ZN_5514_ZN_8418
1hv5_F_ZN_5517_ZN_8523
1hy7_A_ZN_301_ZN_2655
1hy7_B_ZN_801_ZN_2660
1hyt_A_ZN_805_ZN_2444
1i1e_A_ZN_1291_ZN_10658
1i1i_P_ZN_701_ZN_5350
1i1i_P_ZN_702_ZN_5351
1i73_A_ZN_999_ZN_1348
1i76_A_ZN_999_ZN_1319
1iag_A_ZN_999_ZN_1622
1j36_A_ZN_701_ZN_9803
1j36_B_ZN_702_ZN_9833
1j37_A_ZN_701_ZN_9803
1j37_B_ZN_702_ZN_9804
1j38_A_ZN_701_ZN_9803
1j38_B_ZN_702_ZN_9804
1j7n_A_ZN_9001_ZN_11986
1j7n_B_ZN_9002_ZN_11992
1jan_A_ZN_999_ZN_1597
1jao_A_ZN_999_ZN_1542
1jap_A_ZN_999_ZN_1268
1jaq_A_ZN_999_ZN_1515
1jh1_A_ZN_999_ZN_1254
1jiw_P_ZN_481_ZN_4300
1jiz_A_ZN_257_ZN_2609
1jiz_B_ZN_257_ZN_2641
1jj9_A_ZN_999_ZN_1254

1jk3_A_ZN_400_ZN_1292
1k7g_A_ZN_486_ZN_3479
1k7i_A_ZN_486_ZN_3527
1k7q_A_ZN_486_ZN_3524
1kap_P_ZN_613_ZN_3613
1kbc_A_ZN_999_ZN_2594
1kbc_B_ZN_999_ZN_2626
1kei_A_ZN_405_ZN_2485
1kjo_A_ZN_405_ZN_2452
1kjp_A_ZN_405_ZN_2465
1kkk_A_ZN_405_ZN_2465
1kl6_A_ZN_405_ZN_2459
1kr6_A_ZN_405_ZN_2466
1kro_A_ZN_405_ZN_2457
1ks7_A_ZN_405_ZN_2465
1kto_A_ZN_405_ZN_2438
1kuh_A_ZN_133_ZN_1017
1l3f_E_ZN_321_ZN_2433,1l3f_E_ZN_322_ZN_2434
1l6j_A_ZN_500_ZN_3182
1lml_A_ZN_578_ZN_3527
1lnd_E_ZN_800_ZN_2467,1lnd_E_ZN_801_ZN_2468
1lnf_E_ZN_800_ZN_2467
1mmb_A_ZN_999_ZN_1515
1mmp_A_ZN_1_ZN_2551
1mmp_B_ZN_1_ZN_2587
1mmq_A_ZN_1_ZN_1270
1mmr_A_ZN_1_ZN_1275
1mnc_A_ZN_281_ZN_1235
1nd1_A_ZN_400_ZN_1598
1npc_A_ZN_323_ZN_2396
1o86_A_ZN_701_ZN_4666
1o8a_A_ZN_701_ZN_4750
1omj_A_ZN_600_ZN_3372
1os0_A_ZN_600_ZN_2434
1os2_A_ZN_869_ZN_7777
1os2_B_ZN_369_ZN_7787
1os2_C_ZN_469_ZN_7796
1os2_D_ZN_569_ZN_7804
1os2_E_ZN_669_ZN_7814
1os2_F_ZN_769_ZN_7823
1os9_A_ZN_901_ZN_7777
1os9_B_ZN_906_ZN_7782
1os9_C_ZN_911_ZN_7787
1os9_D_ZN_916_ZN_7792
1os9_E_ZN_921_ZN_7797
1os9_F_ZN_926_ZN_7802
1pe5_A_ZN_317_ZN_2434
1pe7_A_ZN_317_ZN_2452
1pe8_A_ZN_317_ZN_2452
1pwp_A_ZN_9001_ZN_12122
1pwp_B_ZN_9002_ZN_12123
1pwq_A_ZN_9001_ZN_12057
1pwq_B_ZN_9002_ZN_12058
1pwu_A_ZN_9001_ZN_12183
1pwu_B_ZN_9002_ZN_12184
1pww_A_ZN_9001_ZN_12188
1pww_B_ZN_9002_ZN_12189
1q3a_A_ZN_464_ZN_3750

1q3a_B_ZN_469_ZN_3755
1q3a_C_ZN_474_ZN_3760
1qf0_A_ZN_320_ZN_2434
1qf1_A_ZN_320_ZN_2434
1qf2_A_ZN_320_ZN_2434
1qia_A_ZN_301_ZN_5121
1qia_B_ZN_301_ZN_5126
1qia_C_ZN_301_ZN_5131
1qia_D_ZN_301_ZN_5136
1qib_A_ZN_501_ZN_1274
1qic_A_ZN_501_ZN_5105
1qic_B_ZN_501_ZN_5110
1qic_C_ZN_501_ZN_5115
1qic_D_ZN_501_ZN_5120
1qji_A_ZN_1201_ZN_1932
1qjj_A_ZN_250_ZN_1961
1qua_A_ZN_999_ZN_1499
1r1h_A_ZN_1001_ZN_5639
1r1i_A_ZN_1001_ZN_5639
1r1j_A_ZN_1001_ZN_5639
1r42_A_ZN_804_ZN_5214
1r4l_A_ZN_803_ZN_5182
1r54_A_ZN_201_ZN_1583
1r55_A_ZN_201_ZN_1612
1rm8_A_ZN_500_ZN_1346
1rmz_A_ZN_264_ZN_1248
1ros_A_ZN_400_ZN_2499
1ros_B_ZN_500_ZN_2539
1s0c_A_ZN_1291_ZN_10588
1s0d_A_ZN_1291_ZN_10597
1s0e_A_ZN_1291_ZN_10587
1s0f_A_ZN_1291_ZN_10431
1s4b_P_ZN_1_ZN_5306
1sat_A_ZN_472_ZN_3541
1slm_A_ZN_257_ZN_2181
1sln_A_ZN_257_ZN_1610
1smp_A_ZN_472_ZN_4309
1sqm_A_ZN_1001_ZN_4873
1srp_A_ZN_920_ZN_3569
1su3_A_ZN_913_ZN_6683
1su3_B_ZN_913_ZN_6696
1t3a_A_ZN_422_ZN_6400
1t3a_B_ZN_422_ZN_6402
1t3c_A_ZN_422_ZN_6584
1t3c_B_ZN_422_ZN_6587
1thl_A_ZN_324_ZN_2438
1tli_A_ZN_317_ZN_2453
1tlp_E_ZN_321_ZN_2476
1tlx_A_ZN_401_ZN_2477
1tmn_E_ZN_321_ZN_2474
1u4g_A_ZN_9800_ZN_2306
1uea_A_ZN_1_ZN_5523
1uea_C_ZN_1_ZN_5528
1ums_A_ZN_1_ZN_2574,1ums_A_ZN_1_ZN_2575
1umt_A_ZN_1_ZN_2574
1usn_A_ZN_257_ZN_1331
1utt_A_ZN_1267_ZN_1282
1utz_A_ZN_1268_ZN_2568

1utz_B_ZN_1267_ZN_2608
1uze_A_ZN_701_ZN_4668
1uzf_A_ZN_701_ZN_4701
1wgz_A_ZN_601_ZN_12340
1wgz_B_ZN_602_ZN_12347
1wgz_C_ZN_603_ZN_12348
1wni_A_ZN_999_ZN_1593
1xtf_A_ZN_429_ZN_6927
1xtf_B_ZN_857_ZN_6928
1xtg_A_ZN_426_ZN_3900
1xuc_A_ZN_1261_ZN_2670
1xuc_B_ZN_1261_ZN_2702
1xud_A_ZN_1261_ZN_2670
1xud_B_ZN_1261_ZN_2704
1xur_A_ZN_1261_ZN_2670
1xur_B_ZN_1261_ZN_2700
1y3g_E_ZN_321_ZN_2438
1y79_1_ZN_700_ZN_5470
1y8j_A_ZN_800_ZN_5639
1y93_A_ZN_264_ZN_2417
1ycm_A_ZN_264_ZN_2424
1you_A_ZN_301_ZN_2640
1you_B_ZN_303_ZN_2682
1yp1_A_ZN_999_ZN_1541
1yqy_A_ZN_781_ZN_4165
1yvg_A_ZN_469_ZN_3224
1z1w_A_ZN_800_ZN_6297
1z3j_A_ZN_264_ZN_2424
1z5h_A_ZN_2001_ZN_12658
1z5h_B_ZN_2002_ZN_12679
1z7h_A_ZN_445_ZN_3456
1z9g_E_ZN_1005_ZN_2438
1zb7_A_ZN_500_ZN_3326
1zdp_E_ZN_1005_ZN_2438
1zkw_A_ZN_422_ZN_6572
1zkw_B_ZN_822_ZN_6579
1zkx_A_ZN_423_ZN_6302
1zkx_B_ZN_822_ZN_6306
1zl6_A_ZN_422_ZN_6306
1zl6_B_ZN_822_ZN_6312
1zn3_A_ZN_422_ZN_6404
1zn3_B_ZN_822_ZN_6409
1zp5_A_ZN_999_ZN_1288
1zs0_A_ZN_999_ZN_1287
1ztq_A_ZN_550_ZN_5035
1ztq_B_ZN_552_ZN_5075
1ztq_C_ZN_554_ZN_5115
1ztq_D_ZN_556_ZN_5155
1zvx_A_ZN_999_ZN_1287
1zxc_A_ZN_201_ZN_4103
1zxc_B_ZN_202_ZN_4130
1zxv_A_ZN_9001_ZN_12039
1zxv_B_ZN_9002_ZN_12040
2a7g_E_ZN_401_ZN_2434
2a8a_A_ZN_440_ZN_3266
2a8h_A_ZN_486_ZN_4095
2a8h_B_ZN_486_ZN_4123
2a97_A_ZN_1437_ZN_6339

2a97_B_ZN_2437_ZN_6345
2aig_P_ZN_999_ZN_1649
2ajf_A_ZN_901_ZN_12632
2ajf_B_ZN_901_ZN_12687
2ayk_A_ZN_170_ZN_2387
2c6f_A_ZN_701_ZN_9837
2c6f_B_ZN_701_ZN_9937
2c6n_A_ZN_701_ZN_9471
2c6n_B_ZN_701_ZN_9570
2cki_A_ZN_999_ZN_4135
2cki_B_ZN_999_ZN_4157
2clt_A_ZN_1202_ZN_5805
2clt_B_ZN_1402_ZN_5811
2d1n_A_ZN_270_ZN_2647
2d1n_B_ZN_280_ZN_2687
2d1o_A_ZN_257_ZN_2701
2d1o_B_ZN_262_ZN_2739
2ddf_A_ZN_1_ZN_3898
2ddf_B_ZN_4_ZN_3933
2ddy_A_ZN_177_ZN_2678
2dq6_A_ZN_900_ZN_6935
2dqm_A_ZN_900_ZN_6951
2dw0_A_ZN_700_ZN_6489
2dw0_B_ZN_700_ZN_6596
2dw1_A_ZN_700_ZN_6483
2dw1_B_ZN_700_ZN_6561
2dw2_A_ZN_700_ZN_6554
2dw2_B_ZN_700_ZN_6671
2e2d_A_ZN_500_ZN_2728
2e3x_A_ZN_800_ZN_5408
2ero_A_ZN_700_ZN_6589
2ero_B_ZN_700_ZN_6621
2erp_A_ZN_700_ZN_6589
2erp_B_ZN_700_ZN_6649
2erq_A_ZN_700_ZN_6542
2erq_B_ZN_700_ZN_6559
2etf_A_ZN_500_ZN_7036
2etf_B_ZN_501_ZN_7042
2fpq_A_ZN_500_ZN_3406
2fv5_A_ZN_3_ZN_4109
2fv5_B_ZN_4_ZN_4142
2fv9_A_ZN_1_ZN_3984
2fv9_B_ZN_4_ZN_4014
2g4z_A_ZN_321_ZN_2439
2g7n_A_ZN_455_ZN_3366
2g7p_A_ZN_452_ZN_6626
2g7p_B_ZN_451_ZN_6627
2g7q_A_ZN_452_ZN_6626
2g7q_B_ZN_451_ZN_6640
2gtq_A_ZN_1001_ZN_6918
2h1j_A_ZN_601_ZN_9340
2h1j_B_ZN_701_ZN_9341
2h1n_A_ZN_601_ZN_9361
2h1n_B_ZN_701_ZN_9368
2hpo_A_ZN_1250_ZN_7285
2hpt_A_ZN_950_ZN_7092
2hu6_A_ZN_264_ZN_1240
2i47_A_ZN_802_ZN_8083

2i47_B_ZN_801_ZN_8113
2i47_C_ZN_804_ZN_8143
2i47_D_ZN_803_ZN_8179
2ilp_A_ZN_500_ZN_6649
2ilp_B_ZN_500_ZN_6668
2ima_A_ZN_500_ZN_6519
2ima_B_ZN_500_ZN_6534
2imb_A_ZN_500_ZN_6429
2imb_B_ZN_500_ZN_6443
2imc_A_ZN_600_ZN_6509
2imc_B_ZN_600_ZN_6510
2ise_A_ZN_500_ZN_6811
2ise_B_ZN_501_ZN_6812
2isg_A_ZN_500_ZN_6735
2isg_B_ZN_501_ZN_6736
2ish_A_ZN_500_ZN_6735
2ish_B_ZN_501_ZN_6736
2iul_A_ZN_1634_ZN_4865
2iux_A_ZN_1621_ZN_4721
2j0t_A_ZN_1267_ZN_6499
2j0t_B_ZN_1268_ZN_6504
2j0t_C_ZN_1265_ZN_6509
2j83_A_ZN_999_ZN_4086
2j83_B_ZN_999_ZN_4133
2jih_A_ZN_1553_ZN_4406
2jih_B_ZN_1554_ZN_4446
2jnp_A_ZN_250_ZN_2492
2jsd_A_ZN_276_ZN_2407
2jt5_A_ZN_256_ZN_2493
2jt6_A_ZN_256_ZN_2493
2k2g_A_ZN_2_ZN_2541
2np0_A_ZN_1291_ZN_10774
2nyy_A_ZN_1_ZN_13509
2nz9_A_ZN_1_ZN_26988
2nz9_B_ZN_1297_ZN_26989
2o36_A_ZN_690_ZN_5304
2o3e_A_ZN_679_ZN_5353
2oc2_A_ZN_701_ZN_4759
2oi0_A_ZN_2_ZN_2110
2ovx_A_ZN_444_ZN_2545
2ovx_B_ZN_444_ZN_2589
2ovz_A_ZN_444_ZN_2513
2ovz_B_ZN_444_ZN_2563
2ow0_A_ZN_444_ZN_2513
2ow0_B_ZN_444_ZN_2554
2ow1_A_ZN_444_ZN_2513
2ow1_B_ZN_444_ZN_2549
2ow2_A_ZN_444_ZN_2489
2ow2_B_ZN_444_ZN_2515
2ow9_A_ZN_601_ZN_2698
2ow9_B_ZN_606_ZN_2741
2oxu_A_ZN_264_ZN_1240
2oxw_A_ZN_264_ZN_1258
2oxz_A_ZN_264_ZN_1273
2oy2_A_ZN_999_ZN_2524
2oy2_F_ZN_999_ZN_2528
2oy4_A_ZN_999_ZN_2487
2oy4_F_ZN_999_ZN_2491

2ozr_A_ZN_4001_ZN_10567
2ozr_B_ZN_4006_ZN_10604
2ozr_C_ZN_4011_ZN_10641
2ozr_D_ZN_4016_ZN_10683
2ozr_E_ZN_4021_ZN_10725
2ozr_F_ZN_4026_ZN_10767
2ozr_G_ZN_4031_ZN_10809
2ozr_H_ZN_4036_ZN_10846
2pjt_A_ZN_302_ZN_5159
2pjt_B_ZN_302_ZN_5196
2pjt_C_ZN_302_ZN_5232
2pjt_D_ZN_302_ZN_5269
2poj_A_ZN_264_ZN_2507
2qn0_A_ZN_431_ZN_3475
2qpj_A_ZN_1_ZN_5639
2r59_A_ZN_701_ZN_4879
2rjp_A_ZN_1_ZN_8597
2rjp_B_ZN_1_ZN_8637
2rjp_C_ZN_1_ZN_8677
2rjp_D_ZN_1_ZN_8717
2rjq_A_ZN_1_ZN_2266
2srt_A_ZN_257_ZN_2676
2tcl_A_ZN_170_ZN_1291
2tli_A_ZN_317_ZN_2453
2tlx_A_ZN_322_ZN_2488
2tmn_E_ZN_321_ZN_2438
2usn_A_ZN_257_ZN_1314
2v4b_A_ZN_1552_ZN_4318
2v4b_B_ZN_1553_ZN_4334
2vj8_A_ZN_1611_ZN_4879
2vqx_A_ZN_1342_ZN_2529
2w0d_A_ZN_1264_ZN_5072
2w0d_B_ZN_1264_ZN_5117
2w0d_C_ZN_1264_ZN_5149
2w0d_D_ZN_1264_ZN_5191
2w12_A_ZN_1203_ZN_1658
2w13_A_ZN_1203_ZN_1748
2w14_A_ZN_1203_ZN_1703
2w15_A_ZN_1203_ZN_1699
2w2d_A_ZN_1434_ZN_13803
2w2d_C_ZN_1438_ZN_13855
2wo8_A_ZN_1268_ZN_5106
2wo8_B_ZN_1269_ZN_5167
2wo8_C_ZN_1268_ZN_5176
2wo8_D_ZN_1267_ZN_5232
2wo9_A_ZN_1268_ZN_5103
2wo9_B_ZN_1268_ZN_5172
2wo9_C_ZN_1269_ZN_5192
2wo9_D_ZN_1267_ZN_5257
2woa_A_ZN_1270_ZN_5118
2woa_B_ZN_1270_ZN_5185
2woa_C_ZN_1268_ZN_5190
2woa_D_ZN_1267_ZN_5249
2x7m_A_ZN_1174_ZN_2887
2x8y_A_ZN_1616_ZN_4884
2x8z_A_ZN_1616_ZN_4899
2x90_A_ZN_1618_ZN_4940
2x91_A_ZN_1619_ZN_4939

2x92_A_ZN_1615_ZN_4900
2x93_A_ZN_1615_ZN_4915
2x94_A_ZN_1616_ZN_4900
2x95_A_ZN_1615_ZN_4930
2x96_A_ZN_1617_ZN_4944
2x97_A_ZN_1616_ZN_4919
2xdt_A_ZN_5000_ZN_6924
2xhl_A_ZN_1442_ZN_6831
2xhm_A_ZN_1616_ZN_4928
2xpy_A_ZN_1673_ZN_5130
2xpz_A_ZN_1673_ZN_5120
2xq0_A_ZN_1672_ZN_5136
2xs3_A_ZN_999_ZN_2708
2xs3_B_ZN_999_ZN_2710
2xs4_A_ZN_999_ZN_1356
2z2d_A_ZN_264_ZN_2414
2zxg_A_ZN_900_ZN_7084
3ahm_A_ZN_565_ZN_9385
3ahm_B_ZN_565_ZN_9394
3ahn_A_ZN_566_ZN_9429
3ahn_B_ZN_565_ZN_9438
3aho_A_ZN_565_ZN_9377
3aho_B_ZN_565_ZN_9433
3aig_A_ZN_999_ZN_1618
3ayk_A_ZN_170_ZN_2387
3b2p_A_ZN_950_ZN_7254
3b2x_A_ZN_950_ZN_7304
3b2z_A_ZN_1_ZN_17295
3b2z_B_ZN_1_ZN_17298
3b2z_C_ZN_1_ZN_17301
3b2z_D_ZN_1_ZN_17304
3b2z_E_ZN_1_ZN_17307
3b2z_F_ZN_1_ZN_17310
3b2z_G_ZN_1_ZN_17313
3b2z_H_ZN_1_ZN_17316
3b34_A_ZN_950_ZN_7481
3b37_A_ZN_950_ZN_7126
3b3b_A_ZN_950_ZN_7146
3b7r_L_ZN_701_ZN_4879
3b7s_A_ZN_701_ZN_4928
3b7t_A_ZN_701_ZN_4906
3b7u_X_ZN_701_ZN_4879
3b8z_A_ZN_901_ZN_3339
3b8z_B_ZN_901_ZN_3373
3b92_A_ZN_502_ZN_2054
3ba0_A_ZN_471_ZN_2992
3bkk_A_ZN_701_ZN_4915
3bkl_A_ZN_701_ZN_4879
3bon_A_ZN_500_ZN_3497
3boo_A_ZN_500_ZN_3511
3bwi_A_ZN_450_ZN_3425
3c37_A_ZN_301_ZN_3561
3c37_B_ZN_301_ZN_3569
3c88_A_ZN_450_ZN_3451
3c89_A_ZN_450_ZN_3453
3c8a_A_ZN_450_ZN_3453
3c8b_A_ZN_450_ZN_3461
3ce2_A_ZN_703_ZN_4812

3cho_A_ZN_701_ZN_4879
3chp_A_ZN_701_ZN_4863
3chq_A_ZN_701_ZN_4854
3chr_A_ZN_611_ZN_4879
3chs_A_ZN_701_ZN_4878
3cia_A_ZN_701_ZN_18690
3cia_B_ZN_701_ZN_18691
3cia_C_ZN_701_ZN_18692
3cia_D_ZN_701_ZN_18693
3cki_A_ZN_501_ZN_3012
3d0g_A_ZN_901_ZN_12542
3d0g_B_ZN_901_ZN_12589
3d0h_A_ZN_901_ZN_12527
3d0h_B_ZN_901_ZN_12544
3d0i_A_ZN_901_ZN_12523
3d3x_A_ZN_428_ZN_6595
3d3x_B_ZN_822_ZN_6626
3dbk_A_ZN_302_ZN_2319
3dda_A_ZN_450_ZN_3476
3ddb_A_ZN_450_ZN_3478
3dng_A_ZN_999_ZN_2603
3dng_B_ZN_999_ZN_2640
3dnz_A_ZN_405_ZN_2485
3do0_A_ZN_405_ZN_2485
3do1_A_ZN_405_ZN_2485
3do2_A_ZN_405_ZN_2485
3dpe_A_ZN_999_ZN_1287
3dpf_A_ZN_999_ZN_2570
3dpf_B_ZN_999_ZN_2616
3ds9_A_ZN_500_ZN_3303
3dse_A_ZN_501_ZN_3198
3dsl_A_ZN_503_ZN_6429
3dsl_B_ZN_1_ZN_6493
3dwb_A_ZN_771_ZN_5307
3e8r_A_ZN_1_ZN_3981
3e8r_B_ZN_2_ZN_4046
3ebg_A_ZN_1_ZN_7235
3ebh_A_ZN_1_ZN_7326
3ebi_A_ZN_1_ZN_7322
3edg_A_ZN_210_ZN_1615
3edh_A_ZN_210_ZN_1698
3edi_A_ZN_210_ZN_1603
3edz_A_ZN_1_ZN_4062
3edz_B_ZN_2_ZN_4105
3ehx_A_ZN_264_ZN_1240
3ehy_A_ZN_264_ZN_1240
3elm_A_ZN_300_ZN_2634
3elm_B_ZN_300_ZN_2674
3ewj_A_ZN_1_ZN_4004
3ewj_B_ZN_2_ZN_4034
3f15_A_ZN_264_ZN_1240
3f16_A_ZN_264_ZN_1240
3f17_A_ZN_264_ZN_1240
3f18_A_ZN_264_ZN_1240
3f19_A_ZN_264_ZN_1240
3f1a_A_ZN_264_ZN_1240
3f28_A_ZN_321_ZN_2438
3f2p_A_ZN_2005_ZN_2453

3fcq_A_ZN_322_ZN_2441
3ffz_A_ZN_1300_ZN_20113
3ffz_B_ZN_1300_ZN_20120
3fgd_A_ZN_321_ZN_2465
3fh5_A_ZN_1001_ZN_4840
3fh7_A_ZN_1001_ZN_4855
3fh8_A_ZN_1001_ZN_4863
3fhe_A_ZN_1001_ZN_4851
3fie_A_ZN_428_ZN_7044
3fie_B_ZN_822_ZN_7045
3fii_A_ZN_822_ZN_3473
3flf_A_ZN_2004_ZN_2457
3for_A_ZN_805_ZN_2438
3fts_A_ZN_701_ZN_4855
3ftu_A_ZN_701_ZN_4867
3ftv_A_ZN_701_ZN_4855
3ftw_A_ZN_701_ZN_4855
3ftx_A_ZN_701_ZN_4855
3fty_A_ZN_701_ZN_4846
3ftz_A_ZN_701_ZN_4846
3fu0_A_ZN_701_ZN_4855
3fu3_A_ZN_701_ZN_4855
3fu5_A_ZN_701_ZN_4846
3fu6_A_ZN_701_ZN_4846
3fud_A_ZN_701_ZN_4846
3fue_A_ZN_701_ZN_4855
3fuf_A_ZN_701_ZN_4855
3fuh_A_ZN_701_ZN_4850
3fui_A_ZN_704_ZN_4851
3fuj_A_ZN_701_ZN_4849
3fuk_A_ZN_701_ZN_4869
3ful_A_ZN_701_ZN_4839
3fum_A_ZN_701_ZN_4839
3fun_A_ZN_701_ZN_4846
3fv4_A_ZN_321_ZN_2485
3fvp_A_ZN_321_ZN_2465
3fwd_A_ZN_321_ZN_2452
3fxp_A_ZN_3000_ZN_2428
3g42_A_ZN_500_ZN_8114
3g42_B_ZN_500_ZN_8145
3g42_C_ZN_500_ZN_8176
3g42_D_ZN_500_ZN_8207
3gbo_A_ZN_302_ZN_1591
3hb2_P_ZN_486_ZN_3528
3hbu_P_ZN_486_ZN_3558,3hbu_P_ZN_487_ZN_3559
3hbv_P_ZN_486_ZN_2938
3hdb_A_ZN_620_ZN_3297
3hq2_A_ZN_502_ZN_8161
3hq2_B_ZN_502_ZN_8174
3hy7_A_ZN_901_ZN_3362
3hy7_B_ZN_901_ZN_3389
3hy9_A_ZN_901_ZN_3353
3hy9_B_ZN_901_ZN_3389
3hyg_A_ZN_901_ZN_3339
3hyg_B_ZN_901_ZN_3370
3i7g_A_ZN_1261_ZN_2621
3i7g_B_ZN_2261_ZN_2655
3i7i_A_ZN_1261_ZN_2593

3i7i_B_ZN_2261_ZN_2635
3k3q_B_ZN_251_ZN_4291
3k7n_A_ZN_704_ZN_3145
3kec_A_ZN_901_ZN_2656
3kec_B_ZN_901_ZN_2709
3ked_A_ZN_875_ZN_7011
3kej_A_ZN_901_ZN_2534
3kej_B_ZN_901_ZN_2571
3kek_A_ZN_901_ZN_2554
3kek_B_ZN_901_ZN_2592
3khi_A_ZN_301_ZN_1787
3kmc_A_ZN_1_ZN_3934
3kmc_B_ZN_2_ZN_3964
3kme_A_ZN_1_ZN_3899
3kme_B_ZN_2_ZN_3933
3kry_A_ZN_1999_ZN_5208
3kry_B_ZN_2999_ZN_5247
3kry_C_ZN_3999_ZN_5286
3kry_D_ZN_4999_ZN_5325
3l0t_A_ZN_1_ZN_3919
3l0t_B_ZN_2_ZN_3953
3l0v_A_ZN_1_ZN_3906
3l0v_B_ZN_2_ZN_3928
3l3n_A_ZN_701_ZN_4691
3le9_A_ZN_1_ZN_3885
3le9_B_ZN_2_ZN_3916
3lea_A_ZN_485_ZN_4091
3lea_B_ZN_485_ZN_4128
3lgp_A_ZN_1_ZN_3912
3lgp_B_ZN_2_ZN_3954
3lik_A_ZN_264_ZN_1287
3lil_A_ZN_264_ZN_1322
3lir_A_ZN_264_ZN_1288
3ljg_A_ZN_264_ZN_1299
3ljt_A_ZN_901_ZN_1750
3lk8_A_ZN_264_ZN_1240
3lmc_A_ZN_212_ZN_1554
3lq0_A_ZN_999_ZN_1930
3lqb_A_ZN_201_ZN_1610
3lum_A_ZN_999_ZN_8185
3lum_B_ZN_999_ZN_8226
3lum_C_ZN_999_ZN_8279
3lum_D_ZN_999_ZN_8307
3lun_A_ZN_999_ZN_4033
3lun_B_ZN_999_ZN_4062
3ma2_A_ZN_294_ZN_4566
3ma2_D_ZN_294_ZN_4562
3n2u_A_ZN_264_ZN_1240
3n2v_A_ZN_264_ZN_1240
3nf3_A_ZN_600_ZN_3258
3nqx_A_ZN_1_ZN_2279
3nqy_B_ZN_520_ZN_3673
3nqz_B_ZN_1_ZN_3681
3nx7_A_ZN_264_ZN_1240
3nxq_A_ZN_650_ZN_9893
3nxq_B_ZN_650_ZN_10051
3p1v_A_ZN_426_ZN_6592
3p1v_B_ZN_426_ZN_6623

3p24_A_ZN_999_ZN_11437
3p24_B_ZN_999_ZN_11447
3p24_C_ZN_999_ZN_11448
3p24_D_ZN_999_ZN_11474
3p7p_E_ZN_321_ZN_2439
3p7q_E_ZN_321_ZN_2439
3p7r_E_ZN_321_ZN_2439
3p7s_E_ZN_321_ZN_2439
3p7t_E_ZN_321_ZN_2439
3p7u_E_ZN_321_ZN_2439
3p7v_E_ZN_321_ZN_2439
3p7w_E_ZN_321_ZN_2439
3tli_A_ZN_317_ZN_2453
3tmn_E_ZN_321_ZN_2461
3usn_A_ZN_169_ZN_2619
456c_A_ZN_272_ZN_3018
456c_B_ZN_272_ZN_3069
4aig_A_ZN_999_ZN_1618
4ayk_A_ZN_170_ZN_2567
4tli_A_ZN_401_ZN_2453
4tln_A_ZN_321_ZN_2438
4tmn_E_ZN_321_ZN_2475
5tli_A_ZN_317_ZN_2455
5tln_A_ZN_321_ZN_2438
5tmn_E_ZN_321_ZN_2471
6tli_A_ZN_321_ZN_2454
6tmn_E_ZN_321_ZN_2471
7tli_A_ZN_401_ZN_2456
7tln_A_ZN_321_ZN_2438
830c_A_ZN_272_ZN_3146
830c_B_ZN_272_ZN_3197
8tli_A_ZN_401_ZN_2463
8tln_E_ZN_321_ZN_2461
966c_A_ZN_265_ZN_1511

------- SF_254 --------
1hr6_B_ZN_501_ZN_27828
1hr6_D_ZN_502_ZN_27829
1hr6_F_ZN_503_ZN_27830
1hr6_H_ZN_504_ZN_27846
1hr7_B_ZN_501_ZN_27639
1hr7_D_ZN_502_ZN_27640
1hr7_F_ZN_503_ZN_27641
1hr7_H_ZN_504_ZN_27642
1hr8_B_ZN_501_ZN_27980
1hr8_D_ZN_502_ZN_27981
1hr8_F_ZN_503_ZN_27982
1hr8_H_ZN_504_ZN_27998
1hr9_B_ZN_501_ZN_27904
1hr9_D_ZN_502_ZN_27905
1hr9_F_ZN_503_ZN_27906
1hr9_H_ZN_504_ZN_27907
1ie0_A_ZN_1001_ZN_1239
1inn_A_ZN_167_ZN_2317
1inn_B_ZN_167_ZN_2318
1j6v_A_ZN_167_ZN_1164
1j6w_A_ZN_176_ZN_2495
1j6w_B_ZN_176_ZN_2505

1j6x_A_ZN_161_ZN_2442
1j6x_B_ZN_161_ZN_2452
1j98_A_ZN_300_ZN_1239
1joe_A_ZN_205_ZN_4586
1joe_B_ZN_206_ZN_4588
1joe_C_ZN_207_ZN_4590
1joe_D_ZN_208_ZN_4592
1jqw_A_ZN_300_ZN_1193
1jvi_A_ZN_300_ZN_1227
1q2l_A_ZN_963_ZN_7371
1vgx_A_ZN_167_ZN_2308
1vgx_B_ZN_167_ZN_2309
1vh2_A_ZN_167_ZN_1172
1vje_A_ZN_167_ZN_2306
1vje_B_ZN_167_ZN_2325
2fge_A_ZN_996_ZN_15549
2fge_B_ZN_996_ZN_15553
2g54_A_ZN_1100_ZN_15953
2g54_B_ZN_1200_ZN_15960
2jg4_A_ZN_2012_ZN_15654
2jg4_B_ZN_2012_ZN_15667
2wby_A_ZN_3012_ZN_16209
2wby_B_ZN_3012_ZN_16210
2wc0_A_ZN_3012_ZN_16194
2wc0_B_ZN_3012_ZN_16219
2wk3_A_ZN_3013_ZN_15680
2wk3_B_ZN_3013_ZN_15681
3cww_A_ZN_2002_ZN_15539
3cww_B_ZN_2001_ZN_15560
3e4a_A_ZN_2000_ZN_15749
3e4a_B_ZN_2000_ZN_15833
3e4z_B_ZN_2_ZN_15690
3e4z_C_ZN_68_ZN_15691
3e50_A_ZN_1_ZN_15732
3e50_D_ZN_51_ZN_15733
3go9_A_ZN_603_ZN_3728
3hgz_A_ZN_1_ZN_15812
3hgz_B_ZN_2_ZN_15813
3n56_A_ZN_2001_ZN_15591
3n56_B_ZN_2002_ZN_15604
3n57_A_ZN_1_ZN_15657
3n57_B_ZN_2_ZN_15658
3ofi_A_ZN_2012_ZN_15669
3ofi_B_ZN_2012_ZN_15676

------- SF_255 --------
1xyd_A_ZN_94_ZN_2929
1xyd_B_ZN_94_ZN_2932
2psr_A_ZN_103_ZN_772
2wc8_A_ZN_95_ZN_2984
2wc8_B_ZN_95_ZN_2986
2wc8_C_ZN_95_ZN_2988
2wc8_D_ZN_95_ZN_3003
2wcb_A_ZN_100_ZN_1527
2wcb_B_ZN_100_ZN_1529
2wnd_A_ZN_103_ZN_768
2wor_A_ZN_1099_ZN_793
2wos_A_ZN_1099_ZN_793

3cr2_A_ZN_94_ZN_718
3cr5_X_ZN_96_ZN_775
3czt_X_ZN_95_ZN_790
3d0y_A_ZN_94_ZN_1486
3d0y_A_ZN_95_ZN_1487
3d10_A_ZN_94_ZN_1504
3d10_A_ZN_95_ZN_1505
3psr_A_ZN_103_ZN_1542

------- SF_256 --------
1nyr_A_ZN_1002_ZN_10391
1v4p_A_ZN_1001_ZN_3728
1v4p_B_ZN_1002_ZN_3729
1v4p_C_ZN_1003_ZN_3730
1wnu_A_ZN_1001_ZN_2500
1wnu_B_ZN_1002_ZN_2508
1wxo_A_ZN_1001_ZN_3741
1wxo_B_ZN_1002_ZN_3742
1wxo_C_ZN_1003_ZN_3743
2ztg_A_ZN_902_ZN_5925
2zzf_A_ZN_753_ZN_5928
2zzg_A_ZN_901_ZN_11997
2zzg_B_ZN_901_ZN_12027
3kew_A_ZN_240_ZN_3755
3kew_B_ZN_240_ZN_3756

------- SF_257 --------
1p42_A_ZN_501_ZN_4405,1p42_A_ZN_502_ZN_4406
1p42_B_ZN_505_ZN_4425,1p42_B_ZN_506_ZN_4426
1xxe_A_ZN_319_ZN_4363
1yh8_A_ZN_501_ZN_4299
1yh8_B_ZN_505_ZN_4321
1yhc_A_ZN_601_ZN_4299
1yhc_B_ZN_602_ZN_4356
2go3_A_ZN_602_ZN_4345
2go3_B_ZN_601_ZN_4398
2go4_A_ZN_601_ZN_4301
2go4_B_ZN_602_ZN_4335
2ier_A_ZN_601_ZN_4317,2ier_A_ZN_606_ZN_4320
2ier_B_ZN_602_ZN_4371,2ier_B_ZN_607_ZN_4373
2j65_A_ZN_301_ZN_4340,2j65_A_ZN_302_ZN_4341
2j65_B_ZN_301_ZN_4386,2j65_B_ZN_302_ZN_4387
2jt2_A_ZN_336_ZN_4356
2o3z_A_ZN_501_ZN_4344
2o3z_B_ZN_503_ZN_4369
2ves_A_ZN_1295_ZN_6918
2ves_B_ZN_1289_ZN_6950
2ves_C_ZN_1300_ZN_6987
3nzk_A_ZN_307_ZN_4818
3nzk_B_ZN_307_ZN_4889
3p3c_A_ZN_401_ZN_4486
3p3e_A_ZN_400_ZN_4636
3p3g_A_ZN_301_ZN_4705

------- SF_258 --------
1li5_A_ZN_963_ZN_5677
1li5_B_ZN_964_ZN_5678
1li7_A_ZN_963_ZN_5743

1li7_B_ZN_964_ZN_5751
1u0b_B_ZN_462_ZN_5097
3c8z_A_ZN_413_ZN_6250
3c8z_B_ZN_413_ZN_6316

------- SF_259 --------
2ce7_A_ZN_1603_ZN_19198
2ce7_B_ZN_1606_ZN_19227
2ce7_C_ZN_1607_ZN_19256
2ce7_D_ZN_1607_ZN_19285
2ce7_E_ZN_1603_ZN_19314
2ce7_F_ZN_1606_ZN_19343
2cea_A_ZN_1603_ZN_19198
2cea_B_ZN_1606_ZN_19227
2cea_C_ZN_1607_ZN_19256
2cea_D_ZN_1607_ZN_19285
2cea_E_ZN_1603_ZN_19314
2cea_F_ZN_1606_ZN_19343
3b4r_A_ZN_225_ZN_3407
3b4r_B_ZN_225_ZN_3408
3kds_E_ZN_996_ZN_9896
3kds_F_ZN_996_ZN_9930
3kds_G_ZN_996_ZN_9964

------- SF_260 --------
3csk_A_ZN_712_ZN_5702
3fvy_A_ZN_1000_ZN_5642

------- SF_261 --------
1au1_B_ZN_169_ZN_3625

------- SF_262 --------
1fr2_B_ZN_301_ZN_1697
1fsj_B_ZN_901_ZN_4215
1fsj_C_ZN_902_ZN_4221
1fsj_D_ZN_903_ZN_4227
1fsj_E_ZN_904_ZN_4233
1m08_A_ZN_600_ZN_2119
1m08_B_ZN_600_ZN_2125
1mz8_B_ZN_600_ZN_3512
1mz8_D_ZN_600_ZN_3518
1v13_A_ZN_200_ZN_1917
1v13_B_ZN_200_ZN_1918
1v15_A_ZN_1132_ZN_5134
1v15_B_ZN_1135_ZN_5135
1v15_C_ZN_1132_ZN_5136
1v15_D_ZN_1135_ZN_5137
1zns_A_ZN_1500_ZN_1452
2gyk_B_ZN_301_ZN_3416
2gyk_F_ZN_301_ZN_3422
2gze_B_ZN_301_ZN_1701
2gzf_B_ZN_301_ZN_1678
2gzg_B_ZN_301_ZN_1707
2gzi_B_ZN_301_ZN_1706
2gzj_B_ZN_301_ZN_3416
2gzj_F_ZN_301_ZN_3422
2ivh_A_ZN_1577_ZN_1742
2jaz_B_ZN_600_ZN_3322

2jaz_D_ZN_600_ZN_3328
2jb0_B_ZN_600_ZN_1660
2jbg_B_ZN_1577_ZN_3355
2jbg_D_ZN_1577_ZN_3361
3gjn_B_ZN_600_ZN_3231
3gjn_C_ZN_600_ZN_3232
3gkl_A_ZN_600_ZN_3216
3gkl_B_ZN_600_ZN_3217
7cei_B_ZN_600_ZN_1727

------- SF_263 --------
1oek_A_ZN_1195_ZN_1517

------- SF_264 --------
1qr2_A_ZN_231_ZN_4435
1qr2_B_ZN_232_ZN_4489
1sg0_A_ZN_231_ZN_3651
1sg0_B_ZN_232_ZN_3722
1xi2_A_ZN_231_ZN_3651
1xi2_B_ZN_232_ZN_3723,1xi2_B_ZN_432_ZN_3724
1zx1_A_ZN_231_ZN_3573
1zx1_B_ZN_231_ZN_3645
2bzs_A_ZN_231_ZN_3651
2bzs_B_ZN_232_ZN_3723
2qmy_A_ZN_231_ZN_3651
2qmy_B_ZN_232_ZN_3718
2qmz_A_ZN_231_ZN_3651
2qmz_B_ZN_232_ZN_3727
2qr2_A_ZN_231_ZN_4435
2qr2_B_ZN_232_ZN_4489
2qwx_A_ZN_231_ZN_3860
2qwx_B_ZN_231_ZN_3931
2qx4_A_ZN_231_ZN_3817
2qx4_B_ZN_231_ZN_3888
2qx6_A_ZN_231_ZN_3730
2qx6_B_ZN_231_ZN_3801
2qx8_A_ZN_231_ZN_3786
2qx8_B_ZN_231_ZN_3866
2qx9_A_ZN_231_ZN_3740
2qx9_B_ZN_231_ZN_3794
3fw1_A_ZN_231_ZN_3650
3g5m_A_ZN_231_ZN_3718
3g5m_B_ZN_231_ZN_3823
3gam_A_ZN_233_ZN_3821
3gam_B_ZN_233_ZN_3892

------- SF_265 --------
2hcn_A_ZN_2_ZN_3934
2hcn_A_ZN_3_ZN_3935
2hcs_A_ZN_1_ZN_3938
2hcs_A_ZN_2_ZN_3939
2hfz_A_ZN_907_ZN_4918
2j7u_A_ZN_1885_ZN_4704
2j7w_A_ZN_1885_ZN_4624

------- SF_266 --------
2zh0_B_ZN_4002_ZN_12805
2zh0_C_ZN_4003_ZN_12817

2zh0_D_ZN_4001_ZN_12829
2zh0_F_ZN_4008_ZN_12841
2zh0_G_ZN_4009_ZN_12863
2zh0_H_ZN_4007_ZN_12864
2zh0_J_ZN_4011_ZN_12865
2zh0_K_ZN_4012_ZN_12877
2zh0_L_ZN_4010_ZN_12899
2zh0_N_ZN_4005_ZN_12922
2zh0_O_ZN_4006_ZN_12923
2zh0_P_ZN_4004_ZN_12935

------- SF_267 --------
3hwp_A_ZN_295_ZN_4707
3hwp_B_ZN_295_ZN_4710

------- SF_268 --------
1exk_A_ZN_80_ZN_1153
1exk_A_ZN_81_ZN_1154
1nlt_A_ZN_351_ZN_1816
1nlt_A_ZN_352_ZN_1817
2ctt_A_ZN_201_ZN_1477
2ctt_A_ZN_401_ZN_1478

------- SF_269 --------
2r6f_A_ZN_1005_ZN_13926
2r6f_A_ZN_1006_ZN_13927
2r6f_B_ZN_1008_ZN_13983
2r6f_B_ZN_1009_ZN_13984
2vf7_A_ZN_1844_ZN_18800
2vf7_A_ZN_1845_ZN_18801
2vf7_B_ZN_1844_ZN_18856
2vf7_B_ZN_1845_ZN_18857
2vf7_C_ZN_1844_ZN_18912
2vf7_C_ZN_1845_ZN_18913
2vf8_A_ZN_1845_ZN_12641
2vf8_A_ZN_1846_ZN_12642
2vf8_B_ZN_1845_ZN_12712
2vf8_B_ZN_1846_ZN_12713

------- SF_270 --------
2bx2_L_ZN_1512_ZN_4081
2c0b_L_ZN_1511_ZN_3773
2c4r_L_ZN_1512_ZN_3774
2vmk_B_ZN_1514_ZN_14180
2vmk_D_ZN_1516_ZN_14191
2vrt_D_ZN_1509_ZN_13194

------- SF_271 --------
1adn_A_ZN_93_ZN_733
1eyf_A_ZN_93_ZN_1450
1u8b_A_ZN_150_ZN_1725
1wpk_A_ZN_200_ZN_2339
1zgw_A_ZN_500_ZN_3367

------- SF_272 --------
1p9r_A_ZN_601_ZN_2934
1p9w_A_ZN_601_ZN_2979

------- SF_273 --------
1dgz_A_ZN_38_ZN_644
1vt2_4_ZN_781_ZN_89816
2i2t_4_ZN_101_ZN_89790
2i2v_4_ZN_401_ZN_89790
2qam_4_ZN_617_ZN_89823
2qao_4_ZN_624_ZN_89824
2qba_4_ZN_617_ZN_89812
2qbc_4_ZN_624_ZN_89813
2qbe_4_ZN_617_ZN_91260
2qbg_4_ZN_624_ZN_91261
2qbi_4_ZN_617_ZN_91291
2qbk_4_ZN_624_ZN_91292
2qov_4_ZN_617_ZN_89781
2qox_4_ZN_624_ZN_89782
2qoz_4_ZN_617_ZN_89823
2qp1_4_ZN_624_ZN_89824
2z4l_4_ZN_617_ZN_91302
2z4n_4_ZN_624_ZN_91303
2zjp_4_ZN_38_ZN_84392
3df2_4_ZN_3617_ZN_89781
3df4_4_ZN_3624_ZN_89782
3dll_4_ZN_38_ZN_83628
3hux_9_ZN_38_ZN_90792
3huz_9_ZN_38_ZN_90530
3i1n_4_ZN_802_ZN_90111
3i1p_4_ZN_781_ZN_89816
3i1r_4_ZN_802_ZN_90111
3i1t_4_ZN_781_ZN_89816
3i20_4_ZN_802_ZN_90111
3i22_4_ZN_781_ZN_89816
3kni_9_ZN_38_ZN_90943
3knk_9_ZN_38_ZN_90780
3knm_9_ZN_38_ZN_90732
3kno_9_ZN_38_ZN_90402
3oas_4_ZN_781_ZN_89822
3oat_4_ZN_783_ZN_90173
3ofc_4_ZN_795_ZN_90137
3ofd_4_ZN_781_ZN_89822
3ofr_4_ZN_790_ZN_90167
3ofz_4_ZN_790_ZN_90143
3og0_4_ZN_781_ZN_89822
3orb_4_ZN_790_ZN_90169

------- SF_274 --------
2rhq_A_ZN_1_ZN_8338
2rhs_C_ZN_3_ZN_16484

------- SF_275 --------
2bx9_A_ZN_1054_ZN_4718
2bx9_B_ZN_1054_ZN_4719
2bx9_C_ZN_1054_ZN_4720
2bx9_D_ZN_1054_ZN_4721
2bx9_E_ZN_1054_ZN_4722
2bx9_F_ZN_1054_ZN_4723
2bx9_G_ZN_1054_ZN_4724
2bx9_H_ZN_1054_ZN_4725
2bx9_I_ZN_1054_ZN_4726

2bx9_J_ZN_1054_ZN_4727
2bx9_K_ZN_1054_ZN_4728
2bx9_L_ZN_1054_ZN_4729
2ko8_A_ZN_54_ZN_2371
2ko8_B_ZN_54_ZN_2372
2ko8_C_ZN_54_ZN_2373
2zp8_E_ZN_54_ZN_4504
2zp8_F_ZN_54_ZN_4505
2zp8_G_ZN_54_ZN_4506
2zp8_H_ZN_54_ZN_4507
2zp8_I_ZN_54_ZN_4508
2zp8_J_ZN_54_ZN_4509
2zp9_C_ZN_54_ZN_5649
2zp9_D_ZN_54_ZN_5650
2zp9_E_ZN_54_ZN_5651
2zp9_I_ZN_54_ZN_5652
2zp9_J_ZN_54_ZN_5653
3lcz_A_ZN_54_ZN_1612
3lcz_B_ZN_54_ZN_1613
3lcz_C_ZN_54_ZN_1614
3lcz_D_ZN_54_ZN_1615
3ld0_1_ZN_260_ZN_19208
3ld0_2_ZN_60_ZN_19210
3ld0_3_ZN_160_ZN_19211
3ld0_4_ZN_260_ZN_19213
3ld0_5_ZN_60_ZN_19214
3ld0_6_ZN_160_ZN_19216
3ld0_7_ZN_260_ZN_19217
3ld0_8_ZN_60_ZN_19218
3ld0_9_ZN_160_ZN_19220
3ld0_a_ZN_260_ZN_19221
3ld0_A_ZN_60_ZN_19174
3ld0_B_ZN_160_ZN_19175
3ld0_b_ZN_60_ZN_19222
3ld0_c_ZN_160_ZN_19224
3ld0_C_ZN_260_ZN_19177
3ld0_d_ZN_260_ZN_19225
3ld0_D_ZN_60_ZN_19178
3ld0_E_ZN_160_ZN_19180
3ld0_e_ZN_60_ZN_19226
3ld0_f_ZN_160_ZN_19227
3ld0_F_ZN_260_ZN_19181
3ld0_g_ZN_260_ZN_19228
3ld0_G_ZN_60_ZN_19182
3ld0_H_ZN_160_ZN_19183
3ld0_h_ZN_60_ZN_19230
3ld0_i_ZN_160_ZN_19232
3ld0_I_ZN_260_ZN_19184
3ld0_j_ZN_260_ZN_19233
3ld0_J_ZN_60_ZN_19186
3ld0_K_ZN_160_ZN_19188
3ld0_k_ZN_60_ZN_19234
3ld0_l_ZN_160_ZN_19236
3ld0_L_ZN_260_ZN_19189
3ld0_m_ZN_260_ZN_19237
3ld0_M_ZN_60_ZN_19190
3ld0_N_ZN_160_ZN_19191
3ld0_O_ZN_260_ZN_19192

3ld0_P_ZN_60_ZN_19194
3ld0_Q_ZN_160_ZN_19195
3ld0_R_ZN_260_ZN_19196
3ld0_S_ZN_60_ZN_19198
3ld0_T_ZN_160_ZN_19200
3ld0_U_ZN_260_ZN_19201
3ld0_V_ZN_60_ZN_19202
3ld0_W_ZN_160_ZN_19204
3ld0_X_ZN_260_ZN_19205
3ld0_Y_ZN_60_ZN_19206
3ld0_Z_ZN_160_ZN_19207

------- SF_276 --------
1b55_A_ZN_1_ZN_2713
1btk_A_ZN_1_ZN_2675
1btk_B_ZN_180_ZN_2677
1bwn_A_ZN_171_ZN_2717
1bwn_B_ZN_171_ZN_2774
2e6i_A_ZN_201_ZN_981
2ys2_A_ZN_201_ZN_692
2z0p_A_ZN_301_ZN_5301
2z0p_B_ZN_302_ZN_5330
2z0p_C_ZN_303_ZN_5374
2z0p_D_ZN_304_ZN_5403

------- SF_277 --------
2xcm_E_ZN_1223_ZN_6064
2xcm_F_ZN_1223_ZN_6066
2yrt_A_ZN_401_ZN_1093

------- SF_278 --------
1ajy_A_ZN_101_ZN_2293,1ajy_A_ZN_102_ZN_2294
1ajy_B_ZN_101_ZN_2295,1ajy_B_ZN_102_ZN_2296
1f4s_P_ZN_64_ZN_1647,1f4s_P_ZN_65_ZN_1648
1f5e_P_ZN_64_ZN_1647,1f5e_P_ZN_65_ZN_1648
1hwt_C_ZN_136_ZN_4004,1hwt_C_ZN_137_ZN_4005
1hwt_D_ZN_136_ZN_4006,1hwt_D_ZN_137_ZN_4007
1hwt_G_ZN_136_ZN_4009,1hwt_G_ZN_137_ZN_4010
1hwt_H_ZN_136_ZN_4011,1hwt_H_ZN_137_ZN_4012
1pyc_A_ZN_127_ZN_673,1pyc_A_ZN_128_ZN_674
1pyi_A_ZN_21_ZN_1826,1pyi_A_ZN_22_ZN_1827
1pyi_B_ZN_321_ZN_1828,1pyi_B_ZN_322_ZN_1829
1qp9_A_ZN_131_ZN_4018,1qp9_A_ZN_132_ZN_4019
1qp9_B_ZN_131_ZN_4020,1qp9_B_ZN_132_ZN_4021
1qp9_C_ZN_131_ZN_4023,1qp9_C_ZN_132_ZN_4024
1qp9_D_ZN_131_ZN_4025,1qp9_D_ZN_132_ZN_4026
1zme_C_ZN_1_ZN_1801,1zme_C_ZN_2_ZN_1802
1zme_D_ZN_1_ZN_1803,1zme_D_ZN_2_ZN_1804
2alc_A_ZN_64_ZN_1012,2alc_A_ZN_65_ZN_1013
2er8_A_ZN_104_ZN_3000,2er8_A_ZN_105_ZN_3001
2er8_B_ZN_104_ZN_3002,2er8_B_ZN_105_ZN_3003
2er8_C_ZN_105_ZN_3004,2er8_C_ZN_106_ZN_3005
2er8_D_ZN_107_ZN_3006,2er8_D_ZN_108_ZN_3007
2ere_A_ZN_104_ZN_1483,2ere_A_ZN_105_ZN_1484
2ere_B_ZN_104_ZN_1485,2ere_B_ZN_105_ZN_1486
2erg_A_ZN_104_ZN_1531,2erg_A_ZN_105_ZN_1532
2erg_B_ZN_104_ZN_1533,2erg_B_ZN_105_ZN_1534
2hap_C_ZN_136_ZN_2074,2hap_C_ZN_137_ZN_2075

2hap_D_ZN_137_ZN_2077,2hap_D_ZN_138_ZN_2078
3alc_A_ZN_64_ZN_1012,3alc_A_ZN_65_ZN_1013
3coq_A_ZN_1001_ZN_2273,3coq_A_ZN_1002_ZN_2274
3coq_B_ZN_1_ZN_2275,3coq_B_ZN_2_ZN_2276

------- SF_279 --------
1ldj_B_ZN_203_ZN_6670
1ldk_C_ZN_4003_ZN_7927
1u6g_B_ZN_1231_ZN_15496
2cs3_A_ZN_200_ZN_1294
2ecl_A_ZN_401_ZN_1219
2hye_D_ZN_4003_ZN_16946
3dpl_R_ZN_203_ZN_3861
3dqv_R_ZN_4004_ZN_8840
3dqv_Y_ZN_4003_ZN_8845

------- SF_280 --------
2woj_A_ZN_1353_ZN_9406
2woj_C_ZN_1352_ZN_9473
2woo_B_ZN_1323_ZN_14119
2woo_C_ZN_1323_ZN_14120
2woo_F_ZN_1323_ZN_14121
3h84_A_ZN_355_ZN_5076
3io3_A_ZN_349_ZN_1879
3iqw_A_ZN_3_ZN_4152

------- SF_281 --------
1co4_A_ZN_43_ZN_591

------- SF_282 --------
1lpv_A_ZN_54_ZN_863,1lpv_A_ZN_54_ZN_864

------- SF_283 --------
1g71_A_ZN_348_ZN_5707
1g71_B_ZN_348_ZN_5721
1v33_A_ZN_1000_ZN_2859
1v34_A_ZN_1000_ZN_2859

------- SF_284 --------
2gp3_A_ZN_401_ZN_5656
2gp3_B_ZN_401_ZN_5658
2gp5_A_ZN_401_ZN_5627
2gp5_B_ZN_401_ZN_5639
2oq6_A_ZN_502_ZN_5753
2oq6_B_ZN_502_ZN_5765
2oq7_A_ZN_502_ZN_5557
2oq7_B_ZN_502_ZN_5569
2os2_A_ZN_502_ZN_5754
2os2_B_ZN_502_ZN_5766
2ot7_A_ZN_502_ZN_5744
2ot7_B_ZN_502_ZN_5756
2ox0_A_ZN_502_ZN_5784
2ox0_B_ZN_502_ZN_5797
2p5b_A_ZN_351_ZN_5855
2p5b_B_ZN_351_ZN_5871
2pxj_A_ZN_1001_ZN_5705
2pxj_B_ZN_2001_ZN_5717
2q8c_A_ZN_505_ZN_5634

2q8c_B_ZN_506_ZN_5646
2q8d_A_ZN_505_ZN_5678
2q8d_B_ZN_506_ZN_5688
2q8e_A_ZN_505_ZN_5620
2q8e_B_ZN_506_ZN_5632
2vd7_A_ZN_502_ZN_5628
2vd7_B_ZN_502_ZN_5642
2wwj_A_ZN_502_ZN_2859
2wwj_B_ZN_502_ZN_5714
2xml_A_ZN_1349_ZN_5516
2xml_B_ZN_1349_ZN_5541
3dxt_A_ZN_355_ZN_2697
3dxu_A_ZN_350_ZN_2694
3njy_A_ZN_502_ZN_5618
3njy_B_ZN_502_ZN_5634

------- SF_285 --------
1a7t_A_ZN_251_ZN_3491,1a7t_A_ZN_252_ZN_3492
1a7t_B_ZN_251_ZN_3506,1a7t_B_ZN_252_ZN_3507
1a8t_A_ZN_251_ZN_3517,1a8t_A_ZN_252_ZN_3518
1a8t_B_ZN_251_ZN_3553,1a8t_B_ZN_252_ZN_3554
1bc2_A_ZN_228_ZN_3347,1bc2_A_ZN_229_ZN_3348
1bc2_B_ZN_228_ZN_3354,1bc2_B_ZN_229_ZN_3355
1bmc_A_ZN_228_ZN_1658
1bvt_A_ZN_228_ZN_1717,1bvt_A_ZN_229_ZN_1712
1dd6_A_ZN_502_ZN_3400,1dd6_A_ZN_503_ZN_3401
1dd6_B_ZN_504_ZN_3431,1dd6_B_ZN_505_ZN_3432
1ddk_A_ZN_500_ZN_1724,1ddk_A_ZN_501_ZN_1725
1dxk_A_ZN_228_ZN_1716
1hlk_A_ZN_1001_ZN_3489,1hlk_A_ZN_1002_ZN_3490
1hlk_B_ZN_1003_ZN_3515,1hlk_B_ZN_1004_ZN_3516
1jje_A_ZN_251_ZN_3443,1jje_A_ZN_252_ZN_3444
1jje_B_ZN_251_ZN_3475,1jje_B_ZN_252_ZN_3476
1jjt_A_ZN_251_ZN_3443,1jjt_A_ZN_252_ZN_3444
1jjt_B_ZN_251_ZN_3478,1jjt_B_ZN_252_ZN_3479
1jt1_A_ZN_400_ZN_2058,1jt1_A_ZN_401_ZN_2059
1k07_A_ZN_1_ZN_4151,1k07_A_ZN_2_ZN_4152
1k07_B_ZN_3_ZN_4189,1k07_B_ZN_4_ZN_4190
1ko2_A_ZN_300_ZN_1738
1ko3_A_ZN_1_ZN_1735,1ko3_A_ZN_2_ZN_1736
1kr3_A_ZN_301_ZN_3487,1kr3_A_ZN_302_ZN_3488
1kr3_B_ZN_401_ZN_3513,1kr3_B_ZN_402_ZN_3514
1l9y_A_ZN_1_ZN_4155,1l9y_A_ZN_2_ZN_4156
1l9y_B_ZN_1_ZN_4190,1l9y_B_ZN_2_ZN_4191
1m2x_A_ZN_901_ZN_7227,1m2x_A_ZN_902_ZN_7228
1m2x_B_ZN_901_ZN_7250,1m2x_B_ZN_902_ZN_7251
1m2x_C_ZN_901_ZN_7273,1m2x_C_ZN_902_ZN_7274
1m2x_D_ZN_901_ZN_7296,1m2x_D_ZN_902_ZN_7297
1p9e_A_ZN_401_ZN_4407,1p9e_A_ZN_402_ZN_4408
1p9e_B_ZN_1401_ZN_4414
1qh3_A_ZN_261_ZN_4059,1qh3_A_ZN_262_ZN_4060
1qh3_B_ZN_261_ZN_4073,1qh3_B_ZN_262_ZN_4074
1qh5_A_ZN_261_ZN_4059,1qh5_A_ZN_262_ZN_4060
1qh5_B_ZN_261_ZN_4081,1qh5_B_ZN_262_ZN_4082
1sml_A_ZN_270_ZN_2003,1sml_A_ZN_271_ZN_2004
1vgn_A_ZN_301_ZN_3406,1vgn_A_ZN_302_ZN_3407
1vgn_B_ZN_303_ZN_3422,1vgn_B_ZN_304_ZN_3423
1wuo_A_ZN_301_ZN_6849

1wuo_B_ZN_302_ZN_6854
1wuo_C_ZN_303_ZN_6859
1wuo_D_ZN_304_ZN_6864
1wup_A_ZN_301_ZN_6791,1wup_A_ZN_302_ZN_6792
1wup_B_ZN_303_ZN_6793,1wup_B_ZN_304_ZN_6794
1wup_C_ZN_305_ZN_6795,1wup_C_ZN_306_ZN_6796
1wup_D_ZN_307_ZN_6797,1wup_D_ZN_308_ZN_6798
1x8g_A_ZN_401_ZN_1770
1x8h_A_ZN_401_ZN_1803,1x8h_A_ZN_401_ZN_1804
1x8i_A_ZN_401_ZN_1768
1xm8_A_ZN_700_ZN_4030
1xm8_B_ZN_703_ZN_4043
1y44_A_ZN_400_ZN_4404,1y44_A_ZN_401_ZN_4405
1zkp_A_ZN_245_ZN_8025,1zkp_A_ZN_246_ZN_8026
1zkp_B_ZN_245_ZN_8028,1zkp_B_ZN_246_ZN_8029
1zkp_C_ZN_245_ZN_8033,1zkp_C_ZN_246_ZN_8034
1zkp_D_ZN_245_ZN_8036,1zkp_D_ZN_246_ZN_8037
1znb_A_ZN_1_ZN_3487,1znb_A_ZN_2_ZN_3488
1znb_B_ZN_1_ZN_3490,1znb_B_ZN_2_ZN_3491
2a7m_A_ZN_251_ZN_2088,2a7m_A_ZN_252_ZN_2089
2aio_A_ZN_314_ZN_2003,2aio_A_ZN_315_ZN_2004
2az4_A_ZN_601_ZN_6866,2az4_A_ZN_602_ZN_6867
2az4_B_ZN_603_ZN_6868,2az4_B_ZN_604_ZN_6869
2bc2_A_ZN_228_ZN_3374
2bc2_B_ZN_228_ZN_3390
2bfk_A_ZN_1300_ZN_3491,2bfk_A_ZN_1301_ZN_3492
2bfk_B_ZN_1299_ZN_3555,2bfk_B_ZN_1300_ZN_3556
2bfl_A_ZN_1296_ZN_3397,2bfl_A_ZN_1297_ZN_3398
2bfl_B_ZN_1298_ZN_3442,2bfl_B_ZN_1299_ZN_3443
2bfz_A_ZN_1296_ZN_3380
2bfz_B_ZN_1297_ZN_3429
2bg2_A_ZN_1301_ZN_3411,2bg2_A_ZN_1302_ZN_3412
2bg2_B_ZN_1301_ZN_3483,2bg2_B_ZN_1302_ZN_3484
2bg6_A_ZN_1301_ZN_3428
2bg6_B_ZN_1301_ZN_3498
2bg7_A_ZN_1299_ZN_3395
2bg7_B_ZN_1299_ZN_3442
2bg8_A_ZN_1298_ZN_3402
2bg8_B_ZN_1297_ZN_3465
2bga_A_ZN_1296_ZN_3370
2bga_B_ZN_1293_ZN_3389
2bib_A_ZN_1549_ZN_4551,2bib_A_ZN_1550_ZN_4552
2bmi_A_ZN_271_ZN_3513,2bmi_A_ZN_272_ZN_3514
2bmi_B_ZN_271_ZN_3516,2bmi_B_ZN_272_ZN_3517
2br6_A_ZN_1251_ZN_1915,2br6_A_ZN_1252_ZN_1916
2btn_A_ZN_1251_ZN_1933,2btn_A_ZN_1252_ZN_1934
2cbn_A_ZN_401_ZN_2269,2cbn_A_ZN_402_ZN_2270
2cfu_Z_ZN_1_ZN_5282,2cfu_Z_ZN_2_ZN_5283
2cfz_Z_ZN_1_ZN_5278,2cfz_Z_ZN_2_ZN_5279
2cg2_Z_ZN_1_ZN_5260,2cg2_Z_ZN_2_ZN_5261
2cg3_Z_ZN_1_ZN_5215,2cg3_Z_ZN_2_ZN_5216
2dkf_A_ZN_432_ZN_13309,2dkf_A_ZN_433_ZN_13310
2dkf_B_ZN_432_ZN_13311,2dkf_B_ZN_433_ZN_13312
2dkf_C_ZN_432_ZN_13313,2dkf_C_ZN_433_ZN_13314
2dkf_D_ZN_432_ZN_13315,2dkf_D_ZN_433_ZN_13316
2doo_A_ZN_501_ZN_3385,2doo_A_ZN_502_ZN_3386
2doo_B_ZN_503_ZN_3441,2doo_B_ZN_504_ZN_3442
2e7y_A_ZN_1301_ZN_4464,2e7y_A_ZN_1302_ZN_4465

2e7y_B_ZN_2301_ZN_4486,2e7y_B_ZN_2302_ZN_4487
2fhx_A_ZN_317_ZN_3858
2fhx_B_ZN_317_ZN_3873
2fk6_A_ZN_401_ZN_3523
2fm6_A_ZN_401_ZN_4010,2fm6_A_ZN_402_ZN_4011
2fm6_B_ZN_401_ZN_4045,2fm6_B_ZN_402_ZN_4046
2fu8_A_ZN_401_ZN_4011,2fu8_A_ZN_402_ZN_4012
2fu8_B_ZN_401_ZN_4058,2fu8_B_ZN_402_ZN_4059
2fu9_A_ZN_401_ZN_4077,2fu9_A_ZN_402_ZN_4078
2fu9_B_ZN_401_ZN_4131,2fu9_B_ZN_402_ZN_4132
2gfj_A_ZN_401_ZN_4005,2gfj_A_ZN_402_ZN_4006
2gfj_B_ZN_401_ZN_4050,2gfj_B_ZN_402_ZN_4051
2gfk_A_ZN_401_ZN_4005,2gfk_A_ZN_402_ZN_4006
2gfk_B_ZN_401_ZN_4054,2gfk_B_ZN_402_ZN_4055
2gkl_A_ZN_401_ZN_1771
2gmn_A_ZN_801_ZN_4041,2gmn_A_ZN_802_ZN_4042
2gmn_B_ZN_803_ZN_4044,2gmn_B_ZN_804_ZN_4045
2h6a_A_ZN_320_ZN_4052
2h6a_B_ZN_321_ZN_4073
2hb9_A_ZN_401_ZN_2013,2hb9_A_ZN_402_ZN_2014
2i7t_A_ZN_481_ZN_3238,2i7t_A_ZN_482_ZN_3239
2i7v_A_ZN_481_ZN_3473,2i7v_A_ZN_482_ZN_3474
2nxa_A_ZN_301_ZN_1670
2nyp_A_ZN_301_ZN_1670,2nyp_A_ZN_302_ZN_1671
2nze_A_ZN_401_ZN_3354
2nze_B_ZN_402_ZN_3398
2nzf_A_ZN_228_ZN_1668
2p18_A_ZN_301_ZN_2195,2p18_A_ZN_302_ZN_2196
2p1e_A_ZN_301_ZN_2148,2p1e_A_ZN_302_ZN_2149
2q42_A_ZN_700_ZN_3959
2q42_B_ZN_703_ZN_3969,2q42_B_ZN_703_ZN_3972
2qds_A_ZN_401_ZN_1773
2qdt_A_ZN_401_ZN_2015,2qdt_A_ZN_402_ZN_2016
2qin_A_ZN_2001_ZN_7942,2qin_A_ZN_2002_ZN_7943
2qin_B_ZN_2001_ZN_7944,2qin_B_ZN_2002_ZN_7945
2qin_C_ZN_2001_ZN_7946,2qin_C_ZN_2002_ZN_7947
2qin_D_ZN_2001_ZN_7948,2qin_D_ZN_2002_ZN_7949
2qjs_A_ZN_2001_ZN_7421,2qjs_A_ZN_2002_ZN_7422
2qjs_B_ZN_2001_ZN_7423,2qjs_B_ZN_2002_ZN_7424
2qjs_C_ZN_2001_ZN_7425,2qjs_C_ZN_2002_ZN_7426
2qjs_D_ZN_2001_ZN_7427,2qjs_D_ZN_2002_ZN_7428
2r2d_A_ZN_277_ZN_13575,2r2d_A_ZN_278_ZN_13576
2r2d_B_ZN_277_ZN_13600,2r2d_B_ZN_278_ZN_13601
2r2d_C_ZN_277_ZN_13624,2r2d_C_ZN_278_ZN_13625
2r2d_D_ZN_277_ZN_13653,2r2d_D_ZN_278_ZN_13654
2r2d_E_ZN_277_ZN_13666,2r2d_E_ZN_278_ZN_13667
2r2d_F_ZN_277_ZN_13684,2r2d_F_ZN_278_ZN_13685
2uyx_A_ZN_1296_ZN_1713,2uyx_A_ZN_1297_ZN_1714
2whg_A_ZN_1262_ZN_3507,2whg_A_ZN_1263_ZN_3508
2whg_B_ZN_1262_ZN_3522,2whg_B_ZN_1263_ZN_3523
2wrs_A_ZN_1262_ZN_3468
2wrs_B_ZN_1262_ZN_3508
2xf4_A_ZN_1211_ZN_1628
2xr1_A_ZN_1638_ZN_9348,2xr1_A_ZN_1639_ZN_9349
2xr1_B_ZN_1638_ZN_9350,2xr1_B_ZN_1639_ZN_9351
2yz3_A_ZN_301_ZN_3389,2yz3_A_ZN_302_ZN_3390
2yz3_B_ZN_303_ZN_3416,2yz3_B_ZN_304_ZN_3417
2zo4_A_ZN_318_ZN_2340,2zo4_A_ZN_319_ZN_2341

2zwr_A_ZN_208_ZN_3064,2zwr_A_ZN_209_ZN_3065
2zwr_B_ZN_208_ZN_3072,2zwr_B_ZN_209_ZN_3073
3a4y_A_ZN_452_ZN_13397
3a4y_B_ZN_448_ZN_13486
3a4y_C_ZN_446_ZN_13565
3a4y_D_ZN_441_ZN_13611
3adr_A_ZN_262_ZN_4049,3adr_A_ZN_263_ZN_4050
3adr_B_ZN_262_ZN_4098,3adr_B_ZN_263_ZN_4099
3af5_A_ZN_662_ZN_5151,3af5_A_ZN_665_ZN_5154
3af6_A_ZN_657_ZN_5234,3af6_A_ZN_658_ZN_5235
3bc2_A_ZN_228_ZN_1669
3bk1_A_ZN_948_ZN_4332,3bk1_A_ZN_949_ZN_4333
3bk2_A_ZN_948_ZN_4311,3bk2_A_ZN_949_ZN_4312
3dha_A_ZN_255_ZN_2107,3dha_A_ZN_256_ZN_2108
3dhb_A_ZN_251_ZN_2102,3dhb_A_ZN_252_ZN_2103
3dhc_A_ZN_251_ZN_2062,3dhc_A_ZN_252_ZN_2063
3f9o_A_ZN_308_ZN_1886,3f9o_A_ZN_309_ZN_1887
3fai_A_ZN_1_ZN_1884,3fai_A_ZN_2_ZN_1885
3fcz_A_ZN_292_ZN_3387,3fcz_A_ZN_293_ZN_3388
3fcz_B_ZN_292_ZN_3389,3fcz_B_ZN_293_ZN_3390
3h3e_A_ZN_256_ZN_2035
3i13_A_ZN_228_ZN_1660,3i13_A_ZN_229_ZN_1661
3idz_A_ZN_455_ZN_13444,3idz_A_ZN_456_ZN_13445
3idz_B_ZN_457_ZN_13587,3idz_B_ZN_458_ZN_13588
3idz_C_ZN_447_ZN_13664,3idz_C_ZN_448_ZN_13665
3idz_D_ZN_438_ZN_13696,3idz_D_ZN_439_ZN_13697
3ie0_A_ZN_454_ZN_13459,3ie0_A_ZN_455_ZN_13460
3ie0_B_ZN_452_ZN_13569,3ie0_B_ZN_453_ZN_13570
3ie0_C_ZN_451_ZN_13666,3ie0_C_ZN_452_ZN_13667
3ie0_D_ZN_441_ZN_13713,3ie0_D_ZN_442_ZN_13714
3ie1_A_ZN_452_ZN_13693,3ie1_A_ZN_453_ZN_13694
3ie1_B_ZN_449_ZN_13780,3ie1_B_ZN_450_ZN_13781
3ie1_C_ZN_442_ZN_13832,3ie1_C_ZN_443_ZN_13833
3ie1_D_ZN_441_ZN_13879,3ie1_D_ZN_442_ZN_13880
3ie2_A_ZN_457_ZN_13431
3ie2_B_ZN_451_ZN_13527
3ie2_C_ZN_446_ZN_13598
3ie2_D_ZN_440_ZN_13639
3iek_A_ZN_461_ZN_13462,3iek_A_ZN_462_ZN_13463
3iek_B_ZN_464_ZN_13632,3iek_B_ZN_465_ZN_13633
3iek_C_ZN_455_ZN_13749,3iek_C_ZN_456_ZN_13750
3iek_D_ZN_450_ZN_13841,3iek_D_ZN_451_ZN_13842
3iel_A_ZN_468_ZN_13545,3iel_A_ZN_469_ZN_13546
3iel_B_ZN_458_ZN_13717,3iel_B_ZN_459_ZN_13718
3iel_C_ZN_458_ZN_13865,3iel_C_ZN_459_ZN_13866
3iel_D_ZN_449_ZN_13968,3iel_D_ZN_450_ZN_13969
3iem_A_ZN_448_ZN_13909,3iem_A_ZN_449_ZN_13910
3iem_B_ZN_451_ZN_14014,3iem_B_ZN_452_ZN_14015
3iem_C_ZN_444_ZN_14076,3iem_C_ZN_445_ZN_14077
3iem_D_ZN_443_ZN_14133,3iem_D_ZN_444_ZN_14134
3iof_A_ZN_1_ZN_1921
3iog_A_ZN_1_ZN_1891
3knr_A_ZN_228_ZN_6559,3knr_A_ZN_229_ZN_6560
3knr_B_ZN_228_ZN_6582
3knr_C_ZN_228_ZN_6583,3knr_C_ZN_229_ZN_6584
3knr_D_ZN_228_ZN_6607
3kns_A_ZN_228_ZN_6544,3kns_A_ZN_229_ZN_6545
3kns_B_ZN_228_ZN_6589,3kns_B_ZN_229_ZN_6590

3kns_C_ZN_228_ZN_6604,3kns_C_ZN_229_ZN_6605
3kns_D_ZN_228_ZN_6610,3kns_D_ZN_229_ZN_6611
3l6n_A_ZN_301_ZN_1813,3l6n_A_ZN_302_ZN_1814
3znb_A_ZN_1_ZN_3412
3znb_B_ZN_1_ZN_3415
4znb_A_ZN_1_ZN_3396
4znb_B_ZN_1_ZN_3398

------- SF_286 --------
1amp_A_ZN_501_ZN_2212,1amp_A_ZN_502_ZN_2213
1cg2_A_ZN_500_ZN_11127,1cg2_A_ZN_501_ZN_11128
1cg2_B_ZN_500_ZN_11131,1cg2_B_ZN_501_ZN_11132
1cg2_C_ZN_500_ZN_11134,1cg2_C_ZN_501_ZN_11135
1cg2_D_ZN_500_ZN_11137,1cg2_D_ZN_501_ZN_11138
1cp6_A_ZN_501_ZN_2213,1cp6_A_ZN_502_ZN_2214
1cp7_A_ZN_901_ZN_2034,1cp7_A_ZN_902_ZN_2035
1f2o_A_ZN_901_ZN_2046,1f2o_A_ZN_902_ZN_2047
1f2p_A_ZN_901_ZN_2035,1f2p_A_ZN_902_ZN_2036
1fno_A_ZN_501_ZN_3120,1fno_A_ZN_502_ZN_3121
1ft7_A_ZN_501_ZN_2213,1ft7_A_ZN_502_ZN_2214
1igb_A_ZN_501_ZN_2698,1igb_A_ZN_502_ZN_2699
1jwq_A_ZN_1001_ZN_1365
1lfw_A_ZN_1001_ZN_3653,1lfw_A_ZN_1002_ZN_3654
1lok_A_ZN_901_ZN_2253,1lok_A_ZN_902_ZN_2254
1q7l_A_ZN_1001_ZN_4452,1q7l_A_ZN_1002_ZN_4451
1q7l_C_ZN_1011_ZN_4454,1q7l_C_ZN_1012_ZN_4453
1qq9_A_ZN_901_ZN_2048,1qq9_A_ZN_902_ZN_2049
1r3n_A_ZN_500_ZN_26915,1r3n_A_ZN_501_ZN_26916
1r3n_B_ZN_500_ZN_26924,1r3n_B_ZN_501_ZN_26925
1r3n_C_ZN_500_ZN_26933,1r3n_C_ZN_501_ZN_26934
1r3n_D_ZN_500_ZN_26942,1r3n_D_ZN_501_ZN_26943
1r3n_E_ZN_500_ZN_26951,1r3n_E_ZN_501_ZN_26952
1r3n_F_ZN_500_ZN_26967,1r3n_F_ZN_501_ZN_26968
1r3n_G_ZN_500_ZN_26969,1r3n_G_ZN_501_ZN_26970
1r3n_H_ZN_500_ZN_26985,1r3n_H_ZN_501_ZN_26986
1r43_A_ZN_500_ZN_6719,1r43_A_ZN_501_ZN_6720
1r43_B_ZN_500_ZN_6744,1r43_B_ZN_501_ZN_6745
1rtq_A_ZN_701_ZN_4308,1rtq_A_ZN_702_ZN_4309
1tf8_A_ZN_901_ZN_2119,1tf8_A_ZN_902_ZN_2120
1tf9_A_ZN_901_ZN_2136,1tf9_A_ZN_902_ZN_2137
1tkf_A_ZN_901_ZN_2091,1tkf_A_ZN_902_ZN_2092
1tkh_A_ZN_901_ZN_2132,1tkh_A_ZN_902_ZN_2133
1tkj_A_ZN_901_ZN_2153,1tkj_A_ZN_902_ZN_2154
1txr_A_ZN_501_ZN_2212,1txr_A_ZN_502_ZN_2213
1vhe_A_ZN_372_ZN_2777
1vix_A_ZN_419_ZN_6331,1vix_A_ZN_420_ZN_6332
1vix_B_ZN_419_ZN_6363,1vix_B_ZN_420_ZN_6364
1xbu_A_ZN_901_ZN_2155,1xbu_A_ZN_902_ZN_2156
1xfo_A_ZN_354_ZN_10519,1xfo_A_ZN_382_ZN_10520
1xfo_B_ZN_354_ZN_10521,1xfo_B_ZN_371_ZN_10522
1xfo_C_ZN_354_ZN_10523,1xfo_C_ZN_355_ZN_10524
1xfo_D_ZN_354_ZN_10525,1xfo_D_ZN_355_ZN_10526
1xjo_A_ZN_901_ZN_2003,1xjo_A_ZN_902_ZN_2004
1xov_A_ZN_1001_ZN_2490
1xry_A_ZN_1001_ZN_2212,1xry_A_ZN_1002_ZN_2213
1y0r_A_ZN_1001_ZN_2580,1y0r_A_ZN_1002_ZN_2581
1y0y_A_ZN_1001_ZN_2598,1y0y_A_ZN_1002_ZN_2599
1ylo_A_ZN_1446_ZN_15895,1ylo_A_ZN_1447_ZN_15896

1ylo_B_ZN_1546_ZN_15897,1ylo_B_ZN_1547_ZN_15898
1ylo_C_ZN_1646_ZN_15899,1ylo_C_ZN_1647_ZN_15900
1ylo_D_ZN_1746_ZN_15901,1ylo_D_ZN_1747_ZN_15902
1ylo_E_ZN_1846_ZN_15903,1ylo_E_ZN_1847_ZN_15904
1ylo_F_ZN_1946_ZN_15905,1ylo_F_ZN_1947_ZN_15906
1z2l_A_ZN_511_ZN_6408,1z2l_A_ZN_512_ZN_6409
1z2l_B_ZN_513_ZN_6415,1z2l_B_ZN_514_ZN_6416
1z8l_A_ZN_1751_ZN_22317,1z8l_A_ZN_1752_ZN_22318
1z8l_B_ZN_2751_ZN_22487,1z8l_B_ZN_2752_ZN_22488
1z8l_C_ZN_3751_ZN_22657,1z8l_C_ZN_3752_ZN_22658
1z8l_D_ZN_4751_ZN_22827,1z8l_D_ZN_4752_ZN_22828
2afm_A_ZN_391_ZN_5223
2afm_B_ZN_392_ZN_5229
2afo_A_ZN_391_ZN_5191
2afo_B_ZN_392_ZN_5197
2afs_A_ZN_391_ZN_5229
2afs_B_ZN_392_ZN_5235
2afu_A_ZN_391_ZN_5191
2afu_B_ZN_392_ZN_5206
2afw_A_ZN_996_ZN_5223
2afw_B_ZN_997_ZN_5240
2afx_A_ZN_996_ZN_5223
2afx_B_ZN_997_ZN_5241
2afz_A_ZN_391_ZN_5223
2afz_B_ZN_392_ZN_5236
2anp_A_ZN_501_ZN_2214,2anp_A_ZN_502_ZN_2215
2c6c_A_ZN_1751_ZN_5332,2c6c_A_ZN_1752_ZN_5333
2c6g_A_ZN_1751_ZN_5278,2c6g_A_ZN_1752_ZN_5279
2c6p_A_ZN_1751_ZN_5157,2c6p_A_ZN_1752_ZN_5158
2cij_A_ZN_1751_ZN_5329,2cij_A_ZN_1752_ZN_5330
2dea_A_ZN_401_ZN_2244,2dea_A_ZN_402_ZN_2245
2ek8_A_ZN_1001_ZN_3121,2ek8_A_ZN_1002_ZN_3122
2ek9_A_ZN_1001_ZN_3121,2ek9_A_ZN_1002_ZN_3122
2iq6_A_ZN_292_ZN_2239,2iq6_A_ZN_293_ZN_2240
2jbj_A_ZN_1751_ZN_5320,2jbj_A_ZN_1752_ZN_5321
2jbk_A_ZN_801_ZN_5320,2jbk_A_ZN_802_ZN_5321
2nyq_A_ZN_300_ZN_2227,2nyq_A_ZN_301_ZN_2228
2oot_A_ZN_1751_ZN_5948,2oot_A_ZN_1752_ZN_5949
2or4_A_ZN_1751_ZN_5882,2or4_A_ZN_1752_ZN_5883
2pvv_A_ZN_1751_ZN_5806,2pvv_A_ZN_1752_ZN_5807
2pvw_A_ZN_1751_ZN_5780,2pvw_A_ZN_1752_ZN_5781
2qyv_A_ZN_501_ZN_7249,2qyv_A_ZN_502_ZN_7250
2qyv_B_ZN_501_ZN_7291,2qyv_B_ZN_502_ZN_7292
2v8d_A_ZN_500_ZN_6688,2v8d_A_ZN_501_ZN_6689
2v8d_B_ZN_500_ZN_6690,2v8d_B_ZN_501_ZN_6691
2v8g_A_ZN_500_ZN_13418,2v8g_A_ZN_501_ZN_13419
2v8g_B_ZN_500_ZN_13437,2v8g_B_ZN_501_ZN_13438
2v8g_C_ZN_500_ZN_13450,2v8g_C_ZN_501_ZN_13451
2v8g_D_ZN_500_ZN_13463,2v8g_D_ZN_501_ZN_13464
2v8h_A_ZN_500_ZN_13590,2v8h_A_ZN_501_ZN_13591
2v8h_B_ZN_500_ZN_13612,2v8h_B_ZN_501_ZN_13613
2v8h_C_ZN_500_ZN_13634,2v8h_C_ZN_501_ZN_13635
2v8h_D_ZN_500_ZN_13656,2v8h_D_ZN_501_ZN_13657
2v8v_A_ZN_1455_ZN_13368,2v8v_A_ZN_1456_ZN_13369
2v8v_B_ZN_1454_ZN_13370,2v8v_B_ZN_1455_ZN_13371
2v8v_C_ZN_1454_ZN_13381,2v8v_C_ZN_1455_ZN_13382
2v8v_D_ZN_1454_ZN_13400,2v8v_D_ZN_1455_ZN_13401
2vl1_A_ZN_500_ZN_13552,2vl1_A_ZN_501_ZN_13553

2vl1_B_ZN_500_ZN_13554
2vl1_C_ZN_500_ZN_13555
2vl1_D_ZN_500_ZN_13556
2wzn_A_ZN_401_ZN_2653,2wzn_A_ZN_402_ZN_2654
2xef_A_ZN_1751_ZN_5928,2xef_A_ZN_1752_ZN_5929
2xeg_A_ZN_1751_ZN_5776,2xeg_A_ZN_1752_ZN_5777
2xei_A_ZN_1751_ZN_5716,2xei_A_ZN_1752_ZN_5717
2xej_A_ZN_1751_ZN_5775,2xej_A_ZN_1752_ZN_5776
2zed_A_ZN_391_ZN_5221
2zed_B_ZN_392_ZN_5227
2zee_A_ZN_391_ZN_5219
2zee_B_ZN_392_ZN_5225
2zef_A_ZN_391_ZN_5221
2zef_B_ZN_392_ZN_5222
2zeg_A_ZN_391_ZN_5226
2zeg_B_ZN_392_ZN_5237
2zeh_A_ZN_391_ZN_5223
2zeh_B_ZN_392_ZN_5229
2zel_A_ZN_391_ZN_5209
2zel_B_ZN_392_ZN_5215
2zem_A_ZN_391_ZN_5217
2zem_B_ZN_392_ZN_5223
2zen_A_ZN_391_ZN_5209
2zen_B_ZN_392_ZN_5215
2zeo_A_ZN_391_ZN_5210
2zeo_B_ZN_392_ZN_5216
2zep_A_ZN_391_ZN_5204
2zep_B_ZN_392_ZN_5210
2zog_A_ZN_2001_ZN_7461,2zog_A_ZN_2002_ZN_7462
2zog_B_ZN_3001_ZN_7485,2zog_B_ZN_3002_ZN_7486
3a9l_A_ZN_251_ZN_3221
3a9l_B_ZN_251_ZN_3227
3b35_A_ZN_292_ZN_2524,3b35_A_ZN_293_ZN_2525
3b3c_A_ZN_501_ZN_2210,3b3c_A_ZN_502_ZN_2211
3b3s_A_ZN_292_ZN_2357,3b3s_A_ZN_293_ZN_2358
3b3t_A_ZN_292_ZN_2323,3b3t_A_ZN_293_ZN_2324
3b3v_A_ZN_292_ZN_2384,3b3v_A_ZN_293_ZN_2385
3b3w_A_ZN_292_ZN_2215,3b3w_A_ZN_293_ZN_2216
3b7i_A_ZN_292_ZN_2246,3b7i_A_ZN_293_ZN_2266
3bhx_A_ZN_1751_ZN_5916,3bhx_A_ZN_1752_ZN_5917
3bi0_A_ZN_1751_ZN_5906,3bi0_A_ZN_1752_ZN_5907
3bi1_A_ZN_1751_ZN_5883,3bi1_A_ZN_1752_ZN_5884
3bxm_A_ZN_1751_ZN_5853,3bxm_A_ZN_1752_ZN_5854
3czx_A_ZN_301_ZN_5434
3czx_B_ZN_301_ZN_5435
3czx_C_ZN_301_ZN_5436
3czx_D_ZN_301_ZN_5437
3d7d_A_ZN_1751_ZN_5925,3d7d_A_ZN_1752_ZN_5926
3d7f_A_ZN_1751_ZN_5918,3d7f_A_ZN_1752_ZN_5919
3d7g_A_ZN_1751_ZN_5913,3d7g_A_ZN_1752_ZN_5914
3d7h_A_ZN_1751_ZN_5904,3d7h_A_ZN_1752_ZN_5905
3dlj_A_ZN_2001_ZN_7186,3dlj_A_ZN_2002_ZN_7187
3dlj_B_ZN_2001_ZN_7202,3dlj_B_ZN_2002_ZN_7203
3fec_A_ZN_1751_ZN_5955,3fec_A_ZN_1751_ZN_5956,3fec_A_ZN_1752_ZN_5957,3fec_A_ZN_1752_ZN_5958
3fed_A_ZN_1751_ZN_5891,3fed_A_ZN_1752_ZN_5892
3fee_A_ZN_1751_ZN_5795,3fee_A_ZN_1752_ZN_5796
3ff3_A_ZN_1751_ZN_5927,3ff3_A_ZN_1752_ZN_5928

3fh4_A_ZN_300_ZN_2237,3fh4_A_ZN_301_ZN_2238
3ic1_A_ZN_1001_ZN_5721,3ic1_A_ZN_1002_ZN_5722
3ic1_B_ZN_1001_ZN_5761,3ic1_B_ZN_1002_ZN_5760
3ife_A_ZN_411_ZN_3595,3ife_A_ZN_412_ZN_3596
3iib_A_ZN_1_ZN_3365,3iib_A_ZN_2_ZN_3366
3isz_A_ZN_1001_ZN_5699
3isz_B_ZN_1001_ZN_5705
3iww_A_ZN_1751_ZN_5573,3iww_A_ZN_1752_ZN_5574
3k9t_A_ZN_435_ZN_3397
3kl9_A_ZN_355_ZN_30769,3kl9_A_ZN_356_ZN_30770
3kl9_B_ZN_355_ZN_30771,3kl9_B_ZN_356_ZN_30772
3kl9_C_ZN_355_ZN_30773,3kl9_C_ZN_356_ZN_30774
3kl9_D_ZN_355_ZN_30775,3kl9_D_ZN_356_ZN_30776
3kl9_E_ZN_355_ZN_30777,3kl9_E_ZN_356_ZN_30778
3kl9_F_ZN_355_ZN_30779,3kl9_F_ZN_356_ZN_30780
3kl9_G_ZN_355_ZN_30781,3kl9_G_ZN_356_ZN_30782
3kl9_H_ZN_355_ZN_30783,3kl9_H_ZN_356_ZN_30784
3kl9_I_ZN_355_ZN_30785,3kl9_I_ZN_356_ZN_30786
3kl9_J_ZN_355_ZN_30787,3kl9_J_ZN_356_ZN_30788
3kl9_K_ZN_355_ZN_30789,3kl9_K_ZN_356_ZN_30790
3kl9_L_ZN_355_ZN_30791,3kl9_L_ZN_356_ZN_30792
3mru_A_ZN_491_ZN_7529,3mru_A_ZN_492_ZN_7530
3mru_B_ZN_491_ZN_7531,3mru_B_ZN_492_ZN_7532
3ne8_A_ZN_1_ZN_1895
3pfe_A_ZN_501_ZN_3972,3pfe_A_ZN_502_ZN_3973
3pfo_A_ZN_450_ZN_6624,3pfo_A_ZN_451_ZN_6625
3pfo_B_ZN_450_ZN_6650,3pfo_B_ZN_451_ZN_6651

------- SF_287 --------
1aye_A_ZN_400_ZN_3176
1bav_A_ZN_310_ZN_9749
1bav_B_ZN_310_ZN_9762
1bav_C_ZN_310_ZN_9775
1bav_D_ZN_310_ZN_9788
1cbx_A_ZN_309_ZN_2439
1cps_A_ZN_308_ZN_2438
1cpx_A_ZN_308_ZN_2423,1cpx_A_ZN_309_ZN_2424
1dtd_A_ZN_301_ZN_2846
1f57_A_ZN_310_ZN_2477
1h8l_A_ZN_999_ZN_3160
1hdq_A_ZN_1309_ZN_2460
1hdu_A_ZN_1308_ZN_9749
1hdu_B_ZN_1308_ZN_9765
1hdu_D_ZN_1308_ZN_9781
1hdu_E_ZN_1308_ZN_9797
1hee_A_ZN_1308_ZN_9749
1hee_B_ZN_1308_ZN_9766
1hee_D_ZN_1308_ZN_9783
1hee_E_ZN_1308_ZN_9800
1iy7_A_ZN_308_ZN_2438
1jqg_A_ZN_413_ZN_3231
1kwm_A_ZN_400_ZN_6581
1kwm_B_ZN_400_ZN_6595
1m4l_A_ZN_1308_ZN_2464
1nsa_A_ZN_501_ZN_3176
1obr_A_ZN_400_ZN_2583
1pca_A_ZN_400_ZN_3194
1pyt_B_ZN_350_ZN_7033

1qmu_A_ZN_999_ZN_3180
1uwy_A_ZN_1405_ZN_3206
1yme_A_ZN_310_ZN_2438
1yw4_A_ZN_501_ZN_4856
1yw4_B_ZN_502_ZN_4857
1z5r_A_ZN_400_ZN_7312
1z5r_B_ZN_500_ZN_7313
1z5r_C_ZN_600_ZN_7314
1zg7_A_ZN_400_ZN_7324
1zg7_B_ZN_500_ZN_7342
1zg7_C_ZN_600_ZN_7360
1zg8_A_ZN_400_ZN_7326
1zg8_B_ZN_500_ZN_7343
1zg8_C_ZN_600_ZN_7360
1zg9_A_ZN_400_ZN_7324
1zg9_B_ZN_500_ZN_7338
1zg9_C_ZN_600_ZN_7352
1zlh_A_ZN_555_ZN_2904
1zli_A_ZN_501_ZN_2976
2abz_A_ZN_1001_ZN_6509
2abz_B_ZN_1002_ZN_6510
2bco_A_ZN_501_ZN_5047
2bco_B_ZN_502_ZN_5048
2bo9_A_ZN_999_ZN_8487
2bo9_C_ZN_999_ZN_8502
2boa_A_ZN_999_ZN_6457
2boa_B_ZN_999_ZN_6486
2c1c_A_ZN_625_ZN_4907
2c1c_B_ZN_625_ZN_4909
2ctb_A_ZN_308_ZN_2452
2ctc_A_ZN_308_ZN_2444
2gu2_A_ZN_601_ZN_4933
2gu2_B_ZN_602_ZN_4949
2i3c_A_ZN_314_ZN_4863
2i3c_B_ZN_314_ZN_4884
2jew_A_ZN_1309_ZN_2439
2o4h_A_ZN_401_ZN_4845
2o4h_B_ZN_401_ZN_4859
2o53_A_ZN_314_ZN_4863
2o53_B_ZN_314_ZN_4874
2pcu_A_ZN_999_ZN_2501
2piy_A_ZN_400_ZN_7312
2piy_B_ZN_500_ZN_7346
2piy_C_ZN_600_ZN_7380
2piz_A_ZN_400_ZN_7326
2piz_B_ZN_500_ZN_7354
2piz_C_ZN_600_ZN_7382
2pj0_A_ZN_400_ZN_7312
2pj0_B_ZN_500_ZN_7346
2pj0_C_ZN_600_ZN_7380
2pj1_A_ZN_400_ZN_7326
2pj1_B_ZN_500_ZN_7358
2pj1_C_ZN_600_ZN_7390
2pj2_A_ZN_400_ZN_7312
2pj2_B_ZN_500_ZN_7344
2pj2_C_ZN_600_ZN_7376
2pj3_A_ZN_400_ZN_7326
2pj3_B_ZN_500_ZN_7360

2pj3_C_ZN_600_ZN_7394
2pj4_A_ZN_400_ZN_4875
2pj4_B_ZN_500_ZN_4912
2pj5_A_ZN_400_ZN_7319
2pj5_B_ZN_500_ZN_7355
2pj5_C_ZN_600_ZN_7391
2pj6_A_ZN_400_ZN_2438
2pj7_A_ZN_400_ZN_7312
2pj7_B_ZN_500_ZN_7343
2pj7_C_ZN_600_ZN_7374
2pj8_A_ZN_400_ZN_7312
2pj8_B_ZN_500_ZN_7349
2pj8_C_ZN_600_ZN_7386
2pj9_A_ZN_400_ZN_2438
2pja_A_ZN_400_ZN_7312
2pja_B_ZN_500_ZN_7357
2pja_C_ZN_600_ZN_7402
2pjb_A_ZN_400_ZN_7319
2pjb_B_ZN_500_ZN_7364
2pjb_C_ZN_600_ZN_7409
2pjc_A_ZN_400_ZN_7312
2pjc_B_ZN_500_ZN_7358
2pjc_C_ZN_600_ZN_7404
2q4z_A_ZN_602_ZN_4866,2q4z_B_ZN_602_ZN_4881
2q51_A_ZN_314_ZN_4863
2q51_A_ZN_315_ZN_4864,2q51_B_ZN_314_ZN_4884
2rfh_A_ZN_1308_ZN_2438
2v77_A_ZN_1042_ZN_4909
2v77_B_ZN_1042_ZN_4960
3d4u_A_ZN_309_ZN_2993
3d66_A_ZN_501_ZN_9865
3d66_B_ZN_501_ZN_9866
3d66_C_ZN_501_ZN_9867
3d67_A_ZN_501_ZN_9896
3d67_B_ZN_501_ZN_9897
3d67_C_ZN_501_ZN_9898
3d68_A_ZN_501_ZN_9941
3d68_B_ZN_501_ZN_9942
3d68_C_ZN_501_ZN_9943
3dgv_A_ZN_318_ZN_9625
3dgv_B_ZN_317_ZN_9796
3dgv_C_ZN_310_ZN_9968
3fju_A_ZN_999_ZN_2969
3fvl_A_ZN_1309_ZN_7327
3fvl_C_ZN_1309_ZN_7344
3fvl_E_ZN_1309_ZN_7361
3fx6_A_ZN_309_ZN_7324
3fx6_C_ZN_309_ZN_7344
3fx6_E_ZN_309_ZN_7364
3glj_A_ZN_401_ZN_3207
3hlp_A_ZN_401_ZN_4841
3hlp_B_ZN_402_ZN_4866
3huv_A_ZN_401_ZN_2413
3i1u_A_ZN_401_ZN_2424
3ieh_A_ZN_276_ZN_2060
3kgq_A_ZN_401_ZN_2415
3lms_A_ZN_309_ZN_3069
3lwu_A_ZN_374_ZN_2932

3mn8_A_ZN_999_ZN_12132
3mn8_B_ZN_999_ZN_12180
3mn8_C_ZN_999_ZN_12210
3mn8_D_ZN_999_ZN_12246
3nfz_A_ZN_319_ZN_2387
3nh4_A_ZN_319_ZN_2379
3nh5_A_ZN_319_ZN_2394
3nh8_A_ZN_319_ZN_2391
3osl_A_ZN_999_ZN_5983
3osl_C_ZN_999_ZN_5984
4cpa_I_ZN_308_ZN_5449
4cpa_J_ZN_309_ZN_5450
5cpa_A_ZN_308_ZN_2439
6cpa_A_ZN_308_ZN_2439
7cpa_A_ZN_308_ZN_2439
8cpa_A_ZN_308_ZN_2439

------- SF_288 --------
1ddz_A_ZN_1_ZN_7487
1ddz_A_ZN_2_ZN_7488
1ddz_B_ZN_3_ZN_7489
1ddz_B_ZN_4_ZN_7490
1ekj_A_ZN_4001_ZN_13182
1ekj_B_ZN_4002_ZN_13194
1ekj_C_ZN_4003_ZN_13204
1ekj_D_ZN_4004_ZN_13230
1ekj_E_ZN_4005_ZN_13231
1ekj_F_ZN_4006_ZN_13242
1ekj_G_ZN_4007_ZN_13252
1ekj_H_ZN_4008_ZN_13262
1g5c_A_ZN_1001_ZN_7547
1g5c_B_ZN_1002_ZN_7565
1g5c_C_ZN_1003_ZN_7567
1g5c_D_ZN_1004_ZN_7584
1g5c_E_ZN_1005_ZN_7585
1g5c_F_ZN_1006_ZN_7602
1i6o_A_ZN_301_ZN_3424
1i6o_B_ZN_302_ZN_3425
1i6p_A_ZN_301_ZN_1717
1t75_A_ZN_221_ZN_6865
1t75_B_ZN_221_ZN_6866
1t75_D_ZN_221_ZN_6867
1t75_E_ZN_221_ZN_6868
1ylk_A_ZN_401_ZN_5089
1ylk_B_ZN_402_ZN_5096
1ylk_C_ZN_403_ZN_5100
1ylk_D_ZN_404_ZN_5107
1ym3_A_ZN_301_ZN_1448
2a5v_A_ZN_401_ZN_6168
2a5v_B_ZN_402_ZN_6175
2a5v_C_ZN_403_ZN_6183
2a5v_D_ZN_404_ZN_6184
2a8c_A_ZN_1230_ZN_10597
2a8c_B_ZN_2230_ZN_10598
2a8c_C_ZN_3230_ZN_10609
2a8c_D_ZN_4230_ZN_10610
2a8c_E_ZN_5230_ZN_10621
2a8c_F_ZN_6230_ZN_10632

2a8d_A_ZN_1230_ZN_10597
2a8d_B_ZN_2230_ZN_10602
2a8d_C_ZN_3230_ZN_10617
2a8d_D_ZN_4230_ZN_10627
2a8d_E_ZN_5230_ZN_10637
2a8d_F_ZN_6230_ZN_10652
2esf_A_ZN_300_ZN_3424
2esf_B_ZN_300_ZN_3429
2fgy_A_ZN_620_ZN_7373
2fgy_B_ZN_720_ZN_7375
2w3n_A_ZN_1232_ZN_5267
2w3n_B_ZN_1234_ZN_5282
2w3n_C_ZN_1232_ZN_5287
2w3q_A_ZN_1231_ZN_1771
3e1v_A_ZN_230_ZN_2893
3e1v_B_ZN_230_ZN_2894
3e1w_A_ZN_230_ZN_2893
3e1w_B_ZN_230_ZN_2894
3e24_A_ZN_230_ZN_2945
3e24_B_ZN_230_ZN_2961
3e28_A_ZN_230_ZN_9947
3e28_B_ZN_230_ZN_9968
3e28_C_ZN_230_ZN_9979
3e28_D_ZN_230_ZN_9995
3e28_E_ZN_230_ZN_10006
3e28_F_ZN_230_ZN_10022
3e2a_A_ZN_230_ZN_9913
3e2a_B_ZN_230_ZN_9924
3e2a_C_ZN_230_ZN_9935
3e2a_D_ZN_230_ZN_9946
3e2a_E_ZN_230_ZN_9957
3e2a_F_ZN_230_ZN_9973
3e2w_A_ZN_230_ZN_9757
3e2w_B_ZN_230_ZN_9773
3e2w_C_ZN_230_ZN_9779
3e2w_D_ZN_230_ZN_9785
3e2w_E_ZN_230_ZN_9801
3e2w_F_ZN_230_ZN_9812
3e2x_A_ZN_230_ZN_2923
3e2x_B_ZN_230_ZN_2924
3e31_A_ZN_230_ZN_2890
3e31_B_ZN_230_ZN_2891
3e3f_A_ZN_230_ZN_2897
3e3f_B_ZN_230_ZN_2914
3e3g_A_ZN_230_ZN_9933
3e3g_B_ZN_230_ZN_9954
3e3g_C_ZN_230_ZN_9965
3e3g_D_ZN_230_ZN_9976
3e3g_E_ZN_230_ZN_9982
3e3g_F_ZN_230_ZN_9998
3e3i_A_ZN_230_ZN_20327
3e3i_B_ZN_230_ZN_20333
3e3i_C_ZN_230_ZN_20348
3e3i_D_ZN_230_ZN_20358
3e3i_E_ZN_230_ZN_20368
3e3i_F_ZN_230_ZN_20383
3e3i_G_ZN_230_ZN_20388
3e3i_H_ZN_230_ZN_20393

```
3e3i_I_ZN_230_ZN_20408
3e3i_J_ZN_230_ZN_20418
3e3i_K_ZN_230_ZN_20428
3e3i_L_ZN_230_ZN_20438
3eyx_A_ZN_1_ZN_3133
3eyx_B_ZN_2_ZN_3150

------- SF_289 --------
1ajb_A_ZN_450_ZN_6603,1ajb_A_ZN_451_ZN_6604
1ajb_B_ZN_950_ZN_6611,1ajb_B_ZN_951_ZN_6612
1ajc_A_ZN_450_ZN_6579,1ajc_A_ZN_451_ZN_6580
1ajc_B_ZN_950_ZN_6582,1ajc_B_ZN_951_ZN_6583
1ajd_A_ZN_450_ZN_6603,1ajd_A_ZN_451_ZN_6604
1ajd_B_ZN_950_ZN_6606,1ajd_B_ZN_951_ZN_6607
1alh_A_ZN_450_ZN_8071
1alh_B_ZN_450_ZN_8082
1ali_A_ZN_450_ZN_6561,1ali_A_ZN_451_ZN_6562
1ali_B_ZN_450_ZN_6569,1ali_B_ZN_451_ZN_6570
1alj_A_ZN_451_ZN_6561
1alj_B_ZN_451_ZN_6568
1alk_A_ZN_450_ZN_6611,1alk_A_ZN_451_ZN_6612
1alk_B_ZN_450_ZN_6619,1alk_B_ZN_451_ZN_6620
1ani_A_ZN_450_ZN_6571,1ani_A_ZN_451_ZN_6572,1ani_A_ZN_452_ZN_6573
1ani_B_ZN_450_ZN_6589,1ani_B_ZN_451_ZN_6590,1ani_B_ZN_452_ZN_6591
1anj_A_ZN_450_ZN_6567,1anj_A_ZN_451_ZN_6568,1anj_A_ZN_452_ZN_6569
1anj_B_ZN_450_ZN_6580,1anj_B_ZN_451_ZN_6581,1anj_B_ZN_452_ZN_6582
1b8j_A_ZN_450_ZN_6621,1b8j_A_ZN_451_ZN_6622
1b8j_B_ZN_450_ZN_6629,1b8j_B_ZN_451_ZN_6630
1ed8_A_ZN_450_ZN_6623,1ed8_A_ZN_451_ZN_6624,1ed8_A_ZN_452_ZN_6625
1ed8_B_ZN_950_ZN_6637,1ed8_B_ZN_951_ZN_6638,1ed8_B_ZN_952_ZN_6639
1ed9_A_ZN_450_ZN_6611,1ed9_A_ZN_451_ZN_6612
1ed9_B_ZN_950_ZN_6619,1ed9_B_ZN_951_ZN_6620
1ei6_A_ZN_408_ZN_12218,1ei6_A_ZN_409_ZN_12219
1ei6_B_ZN_408_ZN_12227,1ei6_B_ZN_409_ZN_12228
1ei6_C_ZN_408_ZN_12239,1ei6_C_ZN_409_ZN_12240
1ei6_D_ZN_408_ZN_12248,1ei6_D_ZN_409_ZN_12249
1elx_A_ZN_450_ZN_6609,1elx_A_ZN_451_ZN_6610
1elx_B_ZN_450_ZN_6617,1elx_B_ZN_451_ZN_6618
1ely_A_ZN_450_ZN_6611,1ely_A_ZN_451_ZN_6612
1ely_B_ZN_450_ZN_6619,1ely_B_ZN_451_ZN_6620
1elz_A_ZN_450_ZN_6607,1elz_A_ZN_451_ZN_6608
1elz_B_ZN_450_ZN_6615,1elz_B_ZN_451_ZN_6616
1ew2_A_ZN_1001_ZN_3668,1ew2_A_ZN_1002_ZN_3669
1ew8_A_ZN_450_ZN_6611,1ew8_A_ZN_451_ZN_6612,1ew8_A_ZN_453_ZN_6624
1ew8_B_ZN_450_ZN_6633,1ew8_B_ZN_451_ZN_6634,1ew8_B_ZN_453_ZN_6646
1ew9_A_ZN_450_ZN_6609,1ew9_A_ZN_451_ZN_6610,1ew9_A_ZN_453_ZN_6617
1ew9_B_ZN_450_ZN_6624,1ew9_B_ZN_451_ZN_6625,1ew9_B_ZN_453_ZN_6632
1hjk_A_ZN_450_ZN_6617,1hjk_A_ZN_451_ZN_6618
1hjk_B_ZN_450_ZN_6625,1hjk_B_ZN_451_ZN_6626
1hqa_A_ZN_450_ZN_6563,1hqa_A_ZN_451_ZN_6564,1hqa_A_ZN_452_ZN_6565
1hqa_B_ZN_450_ZN_6566,1hqa_B_ZN_451_ZN_6567,1hqa_B_ZN_452_ZN_6568
1k7h_A_ZN_477_ZN_7481,1k7h_A_ZN_478_ZN_7482,1k7h_A_ZN_479_ZN_7483
1k7h_B_ZN_477_ZN_7529,1k7h_B_ZN_478_ZN_7530,1k7h_B_ZN_479_ZN_7531
1kh4_A_ZN_450_ZN_6523,1kh4_A_ZN_451_ZN_6524
1kh4_B_ZN_450_ZN_6531,1kh4_B_ZN_451_ZN_6532
1kh5_A_ZN_450_ZN_6523,1kh5_A_ZN_451_ZN_6524
1kh5_B_ZN_950_ZN_6530,1kh5_B_ZN_951_ZN_6531
1kh7_A_ZN_450_ZN_6515,1kh7_A_ZN_451_ZN_6516
```

1kh7_B_ZN_450_ZN_6523,1kh7_B_ZN_451_ZN_6524
1kh9_A_ZN_450_ZN_6523,1kh9_A_ZN_451_ZN_6524
1kh9_B_ZN_950_ZN_6530,1kh9_B_ZN_951_ZN_6531
1khj_A_ZN_450_ZN_6519,1khj_A_ZN_451_ZN_6520
1khj_B_ZN_450_ZN_6525,1khj_B_ZN_451_ZN_6526
1khk_A_ZN_450_ZN_6519,1khk_A_ZN_451_ZN_6520
1khk_B_ZN_450_ZN_6522,1khk_B_ZN_451_ZN_6523
1khl_A_ZN_450_ZN_6519,1khl_A_ZN_451_ZN_6520
1khl_B_ZN_450_ZN_6526,1khl_B_ZN_451_ZN_6527
1khn_A_ZN_450_ZN_6523,1khn_A_ZN_451_ZN_6524,1khn_A_ZN_452_ZN_6525
1khn_B_ZN_450_ZN_6526,1khn_B_ZN_451_ZN_6527,1khn_B_ZN_452_ZN_6528
1shn_A_ZN_479_ZN_7481,1shn_A_ZN_484_ZN_7479,1shn_A_ZN_485_ZN_7480
1shn_B_ZN_480_ZN_7506,1shn_B_ZN_481_ZN_7507,1shn_B_ZN_482_ZN_7508
1shq_A_ZN_484_ZN_7479,1shq_A_ZN_485_ZN_7480
1shq_B_ZN_480_ZN_7506,1shq_B_ZN_481_ZN_7507
1ura_A_ZN_450_ZN_6565,1ura_A_ZN_451_ZN_6566
1ura_B_ZN_450_ZN_6577,1ura_B_ZN_451_ZN_6578
1urb_A_ZN_450_ZN_6565
1urb_B_ZN_450_ZN_6577
1zeb_A_ZN_901_ZN_3707,1zeb_A_ZN_902_ZN_3708
1zed_A_ZN_903_ZN_3703,1zed_A_ZN_904_ZN_3704
1zef_A_ZN_901_ZN_3723,1zef_A_ZN_902_ZN_3724
2anh_A_ZN_450_ZN_6569,2anh_A_ZN_451_ZN_6570,2anh_A_ZN_452_ZN_6571
2anh_B_ZN_450_ZN_6582,2anh_B_ZN_451_ZN_6583,2anh_B_ZN_452_ZN_6584
2g9y_A_ZN_450_ZN_6613,2g9y_A_ZN_451_ZN_6614
2g9y_B_ZN_450_ZN_6626,2g9y_B_ZN_451_ZN_6627
2ga3_A_ZN_450_ZN_6621,2ga3_A_ZN_451_ZN_6622
2ga3_B_ZN_450_ZN_6629,2ga3_B_ZN_451_ZN_6630
2glq_A_ZN_2001_ZN_3690,2glq_A_ZN_2002_ZN_3691
2gsn_A_ZN_1000_ZN_6007,2gsn_A_ZN_1001_ZN_6008
2gsn_B_ZN_1002_ZN_6009,2gsn_B_ZN_1003_ZN_6010
2gso_A_ZN_1000_ZN_6039,2gso_A_ZN_1001_ZN_6040
2gso_B_ZN_1002_ZN_6046,2gso_B_ZN_1003_ZN_6047
2gsu_A_ZN_1000_ZN_5922,2gsu_A_ZN_1001_ZN_5923
2gsu_B_ZN_1002_ZN_5947,2gsu_B_ZN_1003_ZN_5948
2iuc_A_ZN_1001_ZN_5092,2iuc_A_ZN_1002_ZN_5093
2iuc_B_ZN_1006_ZN_5098,2iuc_B_ZN_1007_ZN_5099
2rh6_A_ZN_1_ZN_6070,2rh6_A_ZN_2_ZN_6071
2rh6_B_ZN_1_ZN_6095,2rh6_B_ZN_2_ZN_6096
2w5v_A_ZN_1376_ZN_5245,2w5v_A_ZN_1377_ZN_5246
2w5v_B_ZN_1376_ZN_5249,2w5v_B_ZN_1377_ZN_5250
2w5w_A_ZN_1376_ZN_5245,2w5w_A_ZN_1377_ZN_5246,2w5w_A_ZN_1378_ZN_5247
2w5w_B_ZN_1376_ZN_5248,2w5w_B_ZN_1377_ZN_5249,2w5w_B_ZN_1378_ZN_5250
2w5x_A_ZN_1377_ZN_5247,2w5x_A_ZN_1378_ZN_5248
2w5x_B_ZN_1377_ZN_5251,2w5x_B_ZN_1378_ZN_5252
2x98_A_ZN_1475_ZN_6381,2x98_A_ZN_1476_ZN_6382
2x98_B_ZN_1475_ZN_6400,2x98_B_ZN_1476_ZN_6401
2zkt_A_ZN_414_ZN_5497,2zkt_A_ZN_415_ZN_5498
2zkt_B_ZN_414_ZN_5500,2zkt_B_ZN_415_ZN_5501
3a52_A_ZN_1001_ZN_6048
3a52_B_ZN_1004_ZN_6061
3cmr_A_ZN_450_ZN_6535,3cmr_A_ZN_451_ZN_6536
3cmr_B_ZN_450_ZN_6543,3cmr_B_ZN_451_ZN_6544
3dyc_A_ZN_450_ZN_6571,3dyc_A_ZN_451_ZN_6572
3dyc_B_ZN_450_ZN_6580,3dyc_B_ZN_451_ZN_6581
3e2d_A_ZN_601_ZN_8072,3e2d_A_ZN_602_ZN_8073
3e2d_B_ZN_601_ZN_8117,3e2d_B_ZN_602_ZN_8118

------- SF_290 --------
1aui_A_ZN_522_ZN_4393
1hp1_A_ZN_600_ZN_4029,1hp1_A_ZN_601_ZN_4030
1kbp_A_ZN_439_ZN_14262
1kbp_B_ZN_439_ZN_14264
1kbp_C_ZN_439_ZN_14266
1kbp_D_ZN_439_ZN_14268
1m63_A_ZN_504_ZN_11186
1m63_E_ZN_504_ZN_11192
1su1_A_ZN_301_ZN_5654,1su1_A_ZN_302_ZN_5655
1su1_B_ZN_303_ZN_5661,1su1_B_ZN_304_ZN_5662
1su1_C_ZN_305_ZN_5668,1su1_C_ZN_306_ZN_5669
1su1_D_ZN_307_ZN_5680,1su1_D_ZN_308_ZN_5681
1tco_A_ZN_505_ZN_5038
1ush_A_ZN_600_ZN_4020,1ush_A_ZN_601_ZN_4021
2dxn_A_ZN_1001_ZN_4271,2dxn_A_ZN_1002_ZN_4272
2dxn_B_ZN_1003_ZN_4273,2dxn_B_ZN_1004_ZN_4274
2nxf_A_ZN_401_ZN_2573,2nxf_A_ZN_402_ZN_2574
2p6b_A_ZN_509_ZN_8289
2p6b_C_ZN_512_ZN_8300
2qfp_A_ZN_434_ZN_13954
2qfp_B_ZN_434_ZN_14031
2qfp_C_ZN_434_ZN_14098
2qfp_D_ZN_434_ZN_14175
2qfr_A_ZN_434_ZN_6978
2qfr_B_ZN_434_ZN_7048
2ush_A_ZN_601_ZN_8017,2ush_A_ZN_602_ZN_8018
2ush_B_ZN_601_ZN_8032,2ush_B_ZN_602_ZN_8033
2z1a_A_ZN_5647_ZN_3917,2z1a_A_ZN_5648_ZN_3918
2z72_A_ZN_401_ZN_2885,2z72_A_ZN_402_ZN_2886
2zbm_A_ZN_401_ZN_2741,2zbm_A_ZN_402_ZN_2742
3c9f_A_ZN_601_ZN_8998
3c9f_B_ZN_601_ZN_9003
3kbp_A_ZN_439_ZN_14262
3kbp_B_ZN_439_ZN_14269
3kbp_C_ZN_439_ZN_14276
3kbp_D_ZN_439_ZN_14283
4kbp_A_ZN_439_ZN_14052
4kbp_B_ZN_439_ZN_14129
4kbp_C_ZN_439_ZN_14206
4kbp_D_ZN_439_ZN_14283

------- SF_291 --------
1c3r_A_ZN_501_ZN_5981
1c3r_B_ZN_503_ZN_6004
1c3s_A_ZN_951_ZN_2991
1t64_A_ZN_388_ZN_5631
1t64_B_ZN_1388_ZN_5680
1t67_A_ZN_378_ZN_2709
1t69_A_ZN_378_ZN_2739
1vkg_A_ZN_400_ZN_10801
1vkg_B_ZN_400_ZN_10856
1w22_A_ZN_1375_ZN_5480
1w22_B_ZN_1375_ZN_5509
1zz0_A_ZN_1451_ZN_11058
1zz0_B_ZN_1551_ZN_11065
1zz0_C_ZN_1651_ZN_11072
1zz0_D_ZN_1751_ZN_11079

1zz1_A_ZN_2451_ZN_11043
1zz1_B_ZN_2551_ZN_11065
1zz1_C_ZN_2651_ZN_11087
1zz1_D_ZN_2751_ZN_11109
1zz3_A_ZN_1451_ZN_11042
1zz3_B_ZN_1551_ZN_11056
1zz3_C_ZN_1651_ZN_11070
1zz3_D_ZN_1751_ZN_11084
2gh6_A_ZN_9451_ZN_10993
2gh6_B_ZN_9551_ZN_11020
2gh6_C_ZN_9651_ZN_11047
2gh6_D_ZN_9751_ZN_11074
2v5w_A_ZN_1380_ZN_5803
2v5w_B_ZN_1379_ZN_5806
2v5x_A_ZN_1377_ZN_5622
2v5x_B_ZN_1377_ZN_5671
2vcg_A_ZN_1375_ZN_11176
2vcg_B_ZN_1376_ZN_11205
2vcg_C_ZN_1377_ZN_11240
2vcg_D_ZN_1377_ZN_11275
2vqj_A_ZN_1415_ZN_3075
2vqm_A_ZN_1411_ZN_2970
2vqo_A_ZN_1411_ZN_5503
2vqo_B_ZN_1410_ZN_5540
2vqq_A_ZN_1411_ZN_5574
2vqq_B_ZN_1410_ZN_5611
2vqv_A_ZN_1410_ZN_5359
2vqv_B_ZN_1412_ZN_5403
2vqw_G_ZN_1409_ZN_2872
3c0y_A_ZN_401_ZN_8433
3c0y_B_ZN_403_ZN_8437
3c0y_C_ZN_405_ZN_8441
3c0z_A_ZN_101_ZN_8404
3c0z_B_ZN_101_ZN_8416
3c0z_C_ZN_101_ZN_8429
3c10_A_ZN_101_ZN_8428
3c10_B_ZN_101_ZN_8454
3c10_C_ZN_101_ZN_8480
3ew8_A_ZN_601_ZN_2814
3ewf_A_ZN_400_ZN_11537
3ewf_B_ZN_403_ZN_11541
3ewf_C_ZN_406_ZN_11544
3ewf_D_ZN_409_ZN_11548
3ezp_A_ZN_401_ZN_5589
3ezp_B_ZN_404_ZN_5640
3ezt_A_ZN_402_ZN_5595
3ezt_B_ZN_401_ZN_5646
3f06_A_ZN_404_ZN_5569
3f06_B_ZN_401_ZN_5620
3f07_A_ZN_400_ZN_8381
3f07_B_ZN_403_ZN_8406
3f07_C_ZN_406_ZN_8430
3f0r_A_ZN_400_ZN_8465
3f0r_B_ZN_403_ZN_8512
3f0r_C_ZN_406_ZN_8559
3max_A_ZN_379_ZN_8870
3max_B_ZN_379_ZN_8914
3max_C_ZN_379_ZN_8939

3men_A_ZN_400_ZN_10275
3men_B_ZN_400_ZN_10291
3men_C_ZN_400_ZN_10312
3men_D_ZN_400_ZN_10327

------- SF_292 --------
1bll_E_ZN_488_ZN_3707,1bll_E_ZN_489_ZN_3708
1bpn_A_ZN_488_ZN_3673,1bpn_A_ZN_489_ZN_3674
1gyt_A_ZN_600_ZN_46165,1gyt_A_ZN_601_ZN_46166
1gyt_B_ZN_600_ZN_46171,1gyt_B_ZN_601_ZN_46172
1gyt_C_ZN_600_ZN_46177,1gyt_C_ZN_601_ZN_46178
1gyt_D_ZN_600_ZN_46183,1gyt_D_ZN_601_ZN_46184
1gyt_E_ZN_600_ZN_46189,1gyt_E_ZN_601_ZN_46190
1gyt_F_ZN_600_ZN_46195,1gyt_F_ZN_601_ZN_46196
1gyt_G_ZN_600_ZN_46201,1gyt_G_ZN_601_ZN_46202
1gyt_H_ZN_600_ZN_46207,1gyt_H_ZN_601_ZN_46208
1gyt_I_ZN_600_ZN_46213,1gyt_I_ZN_601_ZN_46214
1gyt_J_ZN_600_ZN_46219,1gyt_J_ZN_601_ZN_46220
1gyt_K_ZN_600_ZN_46225,1gyt_K_ZN_601_ZN_46226
1gyt_L_ZN_600_ZN_46231,1gyt_L_ZN_601_ZN_46232
1lam_A_ZN_488_ZN_3707,1lam_A_ZN_489_ZN_3708
1lan_A_ZN_488_ZN_3714,1lan_A_ZN_489_ZN_3715
1lap_A_ZN_488_ZN_4488,1lap_A_ZN_489_ZN_4489
1lcp_A_ZN_488_ZN_7507,1lcp_A_ZN_489_ZN_7508
1lcp_B_ZN_488_ZN_7544,1lcp_B_ZN_489_ZN_7545
2ewb_A_ZN_488_ZN_3768,2ewb_A_ZN_489_ZN_3769
2hc9_A_ZN_701_ZN_3784,2hc9_A_ZN_702_ZN_3785
2j9a_A_ZN_1493_ZN_3983,2j9a_A_ZN_1494_ZN_3984
3h8f_A_ZN_501_ZN_22099
3h8f_B_ZN_501_ZN_22106
3h8f_C_ZN_501_ZN_22113
3h8f_D_ZN_501_ZN_22120
3h8f_E_ZN_501_ZN_22127
3h8f_F_ZN_501_ZN_22134
3h8g_A_ZN_501_ZN_22091
3h8g_B_ZN_501_ZN_22120
3h8g_C_ZN_501_ZN_22149
3h8g_D_ZN_501_ZN_22178
3h8g_E_ZN_501_ZN_22207
3h8g_F_ZN_501_ZN_22236
3jru_A_ZN_629_ZN_7188,3jru_A_ZN_630_ZN_7189
3jru_B_ZN_631_ZN_7181,3jru_B_ZN_632_ZN_7182
3kqx_A_ZN_1001_ZN_46743
3kqx_B_ZN_1001_ZN_46794
3kqx_C_ZN_1001_ZN_46870
3kqx_D_ZN_1001_ZN_46920
3kqx_E_ZN_1001_ZN_46996
3kqx_F_ZN_1001_ZN_47068
3kqx_G_ZN_1001_ZN_47121
3kqx_H_ZN_1001_ZN_47184
3kqx_I_ZN_1001_ZN_47231
3kqx_J_ZN_1001_ZN_47281
3kqx_K_ZN_1001_ZN_47355
3kqx_L_ZN_1001_ZN_47441
3kqz_A_ZN_1001_ZN_46767,3kqz_A_ZN_1004_ZN_46766
3kqz_B_ZN_1001_ZN_46819,3kqz_B_ZN_1004_ZN_46818
3kqz_C_ZN_1001_ZN_46876,3kqz_C_ZN_1004_ZN_46877
3kqz_D_ZN_1001_ZN_46932,3kqz_D_ZN_1004_ZN_46933

3kqz_E_ZN_1001_ZN_47009,3kqz_E_ZN_1004_ZN_47010
3kqz_F_ZN_1001_ZN_47092,3kqz_F_ZN_1004_ZN_47093
3kqz_G_ZN_1001_ZN_47151,3kqz_G_ZN_1004_ZN_47152
3kqz_H_ZN_1001_ZN_47220,3kqz_H_ZN_1004_ZN_47221
3kqz_I_ZN_1001_ZN_47263,3kqz_I_ZN_1004_ZN_47262
3kqz_J_ZN_1001_ZN_47318,3kqz_J_ZN_1004_ZN_47319
3kqz_K_ZN_1001_ZN_47393,3kqz_K_ZN_1004_ZN_47394
3kqz_L_ZN_1001_ZN_47495,3kqz_L_ZN_1004_ZN_47496
3kr4_A_ZN_1001_ZN_47395
3kr4_B_ZN_1001_ZN_47459
3kr4_C_ZN_1001_ZN_47522
3kr4_D_ZN_1001_ZN_47572
3kr4_E_ZN_1001_ZN_47648
3kr4_F_ZN_1001_ZN_47713
3kr4_G_ZN_1001_ZN_47776
3kr4_H_ZN_1001_ZN_47857
3kr4_I_ZN_1001_ZN_47905
3kr4_J_ZN_1001_ZN_47966
3kr4_K_ZN_1001_ZN_48035
3kr4_L_ZN_1001_ZN_48105
3kr5_A_ZN_1001_ZN_47327,3kr5_A_ZN_1004_ZN_47326
3kr5_B_ZN_1001_ZN_47394,3kr5_B_ZN_1004_ZN_47393
3kr5_C_ZN_1001_ZN_47454,3kr5_C_ZN_1004_ZN_47455
3kr5_D_ZN_1001_ZN_47512,3kr5_D_ZN_1004_ZN_47513
3kr5_E_ZN_1001_ZN_47586,3kr5_E_ZN_1004_ZN_47587
3kr5_F_ZN_1001_ZN_47659,3kr5_F_ZN_1004_ZN_47660
3kr5_G_ZN_1001_ZN_47725,3kr5_G_ZN_1004_ZN_47726
3kr5_H_ZN_1001_ZN_47809,3kr5_H_ZN_1004_ZN_47810
3kr5_I_ZN_1001_ZN_47861,3kr5_I_ZN_1004_ZN_47860
3kr5_J_ZN_1001_ZN_47929,3kr5_J_ZN_1004_ZN_47930
3kr5_K_ZN_1001_ZN_48001,3kr5_K_ZN_1004_ZN_48002
3kr5_L_ZN_1001_ZN_48078,3kr5_L_ZN_1004_ZN_48079

------- SF_293 --------
1pq4_A_ZN_1002_ZN_3964
1pq4_B_ZN_1001_ZN_3965
1psz_A_ZN_1000_ZN_2278
1toa_A_ZN_501_ZN_4295
1toa_B_ZN_501_ZN_4302
2ogw_A_ZN_500_ZN_4172
2ogw_B_ZN_501_ZN_4173
2osv_A_ZN_601_ZN_4109
2osv_B_ZN_602_ZN_4110
2ov3_A_ZN_339_ZN_2022
2prs_A_ZN_501_ZN_4163
2prs_B_ZN_502_ZN_4169
2ps0_A_ZN_502_ZN_4093
2ps0_B_ZN_501_ZN_4095
3cx3_A_ZN_314_ZN_4135
3cx3_B_ZN_312_ZN_4138
3gi1_A_ZN_501_ZN_3905
3gi1_B_ZN_501_ZN_3906
3hjt_A_ZN_1_ZN_4069
3hjt_B_ZN_2_ZN_4070

------- SF_294 --------
1q74_A_ZN_304_ZN_8369
1q74_B_ZN_304_ZN_8394

1q74_C_ZN_304_ZN_8395
1q74_D_ZN_304_ZN_8396
2ixd_A_ZN_1234_ZN_3723
2ixd_B_ZN_1232_ZN_3728
3dff_A_ZN_274_ZN_2105
3dfi_A_ZN_300_ZN_1866
3dfk_A_ZN_300_ZN_2076
3dfm_A_ZN_401_ZN_2076
3dfm_A_ZN_402_ZN_2077
3dfm_A_ZN_405_ZN_2080

------- SF_295 --------
1j2t_A_ZN_302_ZN_11935
1j2t_B_ZN_302_ZN_11947
1j2t_C_ZN_302_ZN_11959
1j2t_D_ZN_302_ZN_11971
1j2t_E_ZN_302_ZN_11978
1j2t_F_ZN_302_ZN_11985
1j2u_A_ZN_301_ZN_11936,1j2u_A_ZN_302_ZN_11935
1j2u_B_ZN_301_ZN_11948,1j2u_B_ZN_302_ZN_11947
1j2u_C_ZN_301_ZN_11960,1j2u_C_ZN_302_ZN_11959
1j2u_D_ZN_301_ZN_11972,1j2u_D_ZN_302_ZN_11971
1j2u_E_ZN_301_ZN_11979,1j2u_E_ZN_302_ZN_11978
1j2u_F_ZN_301_ZN_11986,1j2u_F_ZN_302_ZN_11985
1q3k_A_ZN_300_ZN_12007,1q3k_A_ZN_301_ZN_12008
1q3k_B_ZN_300_ZN_12015,1q3k_B_ZN_301_ZN_12016
1q3k_C_ZN_300_ZN_12023,1q3k_C_ZN_301_ZN_12024
1q3k_D_ZN_300_ZN_12031,1q3k_D_ZN_301_ZN_12032
1q3k_E_ZN_300_ZN_12033,1q3k_E_ZN_301_ZN_12034
1q3k_F_ZN_300_ZN_12041,1q3k_F_ZN_301_ZN_12042
1v7z_A_ZN_301_ZN_11936
1v7z_B_ZN_3301_ZN_11962
1v7z_C_ZN_4301_ZN_11988
1v7z_D_ZN_5301_ZN_12014
1v7z_E_ZN_6301_ZN_12040
1v7z_F_ZN_7301_ZN_12066
3a6d_A_ZN_301_ZN_11899
3a6d_B_ZN_301_ZN_11921
3a6d_C_ZN_301_ZN_11943
3a6d_D_ZN_301_ZN_11965
3a6d_E_ZN_301_ZN_11987
3a6d_F_ZN_301_ZN_12009
3a6e_A_ZN_301_ZN_11910
3a6e_B_ZN_301_ZN_11917
3a6e_C_ZN_301_ZN_11924
3a6e_D_ZN_301_ZN_11931
3a6e_E_ZN_301_ZN_11938
3a6e_F_ZN_301_ZN_11945
3a6f_A_ZN_301_ZN_11858
3a6f_B_ZN_301_ZN_11865
3a6f_C_ZN_301_ZN_11872
3a6f_D_ZN_301_ZN_11879
3a6f_E_ZN_301_ZN_11886
3a6f_F_ZN_301_ZN_11893
3a6g_A_ZN_301_ZN_11757
3a6g_B_ZN_301_ZN_11759
3a6g_C_ZN_301_ZN_11761
3a6g_D_ZN_301_ZN_11763

```
3a6g_E_ZN_301_ZN_11765
3a6g_F_ZN_301_ZN_11767
3a6h_A_ZN_301_ZN_11837
3a6h_B_ZN_301_ZN_11840
3a6h_C_ZN_301_ZN_11843
3a6h_D_ZN_301_ZN_11846
3a6h_E_ZN_301_ZN_11849
3a6h_F_ZN_301_ZN_11852
3a6j_A_ZN_301_ZN_11861
3a6j_B_ZN_301_ZN_11886
3a6j_C_ZN_301_ZN_11906
3a6j_D_ZN_301_ZN_11926
3a6j_E_ZN_301_ZN_11937
3a6j_F_ZN_301_ZN_11957
3a6k_A_ZN_301_ZN_11900
3a6k_B_ZN_301_ZN_11903
3a6k_C_ZN_301_ZN_11906
3a6k_D_ZN_301_ZN_11909
3a6k_E_ZN_301_ZN_11912
3a6k_F_ZN_301_ZN_11915
3a6l_A_ZN_300_ZN_11903,3a6l_A_ZN_301_ZN_11904
3a6l_B_ZN_300_ZN_11906,3a6l_B_ZN_301_ZN_11907
3a6l_C_ZN_300_ZN_11909,3a6l_C_ZN_301_ZN_11910
3a6l_D_ZN_300_ZN_11912,3a6l_D_ZN_301_ZN_11913
3a6l_E_ZN_300_ZN_11915,3a6l_E_ZN_301_ZN_11916
3a6l_F_ZN_300_ZN_11918,3a6l_F_ZN_301_ZN_11919
3lub_A_ZN_301_ZN_23857,3lub_A_ZN_302_ZN_23858
3lub_B_ZN_301_ZN_23861,3lub_B_ZN_302_ZN_23862
3lub_C_ZN_301_ZN_23868,3lub_C_ZN_302_ZN_23869
3lub_D_ZN_301_ZN_23880,3lub_D_ZN_302_ZN_23881
3lub_E_ZN_301_ZN_23893,3lub_E_ZN_302_ZN_23894
3lub_F_ZN_301_ZN_23903,3lub_F_ZN_302_ZN_23904
3lub_G_ZN_301_ZN_23907,3lub_G_ZN_302_ZN_23908
3lub_H_ZN_301_ZN_23915,3lub_H_ZN_302_ZN_23916
3lub_I_ZN_301_ZN_23918,3lub_I_ZN_302_ZN_23919
3lub_J_ZN_301_ZN_23923,3lub_J_ZN_302_ZN_23924
3lub_K_ZN_301_ZN_23931,3lub_K_ZN_302_ZN_23932
3lub_L_ZN_301_ZN_23942,3lub_L_ZN_302_ZN_23943

------- SF_296 --------
2gx8_A_ZN_374_ZN_8382,2gx8_A_ZN_375_ZN_8383
2gx8_B_ZN_374_ZN_8422,2gx8_B_ZN_375_ZN_8423
2gx8_C_ZN_374_ZN_8439,2gx8_C_ZN_375_ZN_8440
2nyd_A_ZN_401_ZN_5082,2nyd_A_ZN_402_ZN_5083
2nyd_B_ZN_401_ZN_5084,2nyd_B_ZN_402_ZN_5085
3lnl_A_ZN_401_ZN_5183,3lnl_A_ZN_402_ZN_5184
3lnl_B_ZN_401_ZN_5204,3lnl_B_ZN_402_ZN_5205

------- SF_297 --------
3di4_A_ZN_286_ZN_4346
3di4_B_ZN_286_ZN_4355
3oru_A_ZN_250_ZN_1895

------- SF_298 --------
1hi9_A_ZN_300_ZN_10576,1hi9_A_ZN_301_ZN_10577
1hi9_B_ZN_300_ZN_10578,1hi9_B_ZN_301_ZN_10579
1hi9_C_ZN_300_ZN_10580,1hi9_C_ZN_301_ZN_10581
1hi9_D_ZN_300_ZN_10582,1hi9_D_ZN_301_ZN_10583
```

1hi9_E_ZN_300_ZN_10584,1hi9_E_ZN_301_ZN_10585

------- SF_299 --------
1ps6_A_ZN_330_ZN_4807
1ps6_B_ZN_331_ZN_4821
1ps7_A_ZN_331_ZN_9573
1ps7_B_ZN_330_ZN_9574
1ps7_C_ZN_333_ZN_9575
1ps7_D_ZN_332_ZN_9576
1ptm_A_ZN_330_ZN_4862
1ptm_B_ZN_331_ZN_4868

------- SF_300 --------
2bz0_A_ZN_1174_ZN_2659
2bz0_B_ZN_1174_ZN_2693
2bz1_A_ZN_1174_ZN_1350

------- SF_301 --------
2h1i_A_ZN_300_ZN_4873
2h1i_B_ZN_300_ZN_4876
2h1i_C_ZN_300_ZN_4881

------- SF_302 --------
2pw6_A_ZN_272_ZN_1871

------- SF_303 --------
2q7s_A_ZN_400_ZN_4407
2q7s_B_ZN_400_ZN_4408

------- SF_304 --------
3iuu_A_ZN_495_ZN_3828

------- SF_305 --------
1gup_A_ZN_350_ZN_11123
1gup_B_ZN_350_ZN_11162
1gup_C_ZN_350_ZN_11201
1gup_D_ZN_350_ZN_11240
1guq_A_ZN_350_ZN_11087
1guq_B_ZN_350_ZN_11126
1guq_C_ZN_350_ZN_11165
1guq_D_ZN_350_ZN_11204
1hxp_A_ZN_350_ZN_5357
1hxp_B_ZN_349_ZN_5392
1hxq_A_ZN_350_ZN_5408
1hxq_B_ZN_350_ZN_5430
1y23_A_ZN_1001_ZN_5567
1y23_B_ZN_1002_ZN_5568
1y23_C_ZN_1003_ZN_5570
1y23_D_ZN_1004_ZN_5571
1y23_E_ZN_1005_ZN_5572
1z84_A_ZN_603_ZN_4887
1z84_A_ZN_604_ZN_4888
1z84_B_ZN_605_ZN_4923
1z84_B_ZN_606_ZN_4924
1zwj_A_ZN_400_ZN_4759
1zwj_A_ZN_401_ZN_4760
1zwj_B_ZN_402_ZN_4761
1zwj_B_ZN_403_ZN_4762

2eo4_A_ZN_201_ZN_1227
2h39_A_ZN_352_ZN_4907
2h39_A_ZN_353_ZN_4908
2h39_B_ZN_352_ZN_4948
2h39_B_ZN_353_ZN_4949
2oik_A_ZN_201_ZN_4427
2oik_B_ZN_201_ZN_4447
2oik_C_ZN_201_ZN_4461
2oik_D_ZN_201_ZN_4475
2q4h_A_ZN_603_ZN_4880
2q4h_A_ZN_604_ZN_4881
2q4h_B_ZN_605_ZN_4916,2q4h_B_ZN_605_ZN_4934
2q4h_B_ZN_606_ZN_4917,2q4h_B_ZN_606_ZN_4935
2q4l_A_ZN_400_ZN_4759
2q4l_A_ZN_401_ZN_4760
2q4l_B_ZN_402_ZN_4761
2q4l_B_ZN_403_ZN_4762
3imi_A_ZN_201_ZN_4656
3imi_B_ZN_202_ZN_4667
3imi_C_ZN_203_ZN_4673
3imi_D_ZN_204_ZN_4679
3ksv_A_ZN_201_ZN_1051
3l7x_A_ZN_140_ZN_1190
3o0m_A_ZN_151_ZN_2067
3o0m_B_ZN_151_ZN_2103
3oj7_A_ZN_114_ZN_880
3omf_A_ZN_114_ZN_897
3oxk_A_ZN_120_ZN_911

------- SF_306 --------
1xc3_A_ZN_302_ZN_2287
1z05_A_ZN_406_ZN_3191
1z6r_A_ZN_501_ZN_11729
1z6r_B_ZN_502_ZN_11730
1z6r_C_ZN_503_ZN_11731
1z6r_D_ZN_504_ZN_11732
2aa4_A_ZN_1001_ZN_4154
2aa4_B_ZN_2001_ZN_4155
2ap1_A_ZN_304_ZN_2392
2qm1_A_ZN_1002_ZN_10120
2qm1_B_ZN_1003_ZN_10122
2qm1_C_ZN_1001_ZN_10124
2qm1_D_ZN_1004_ZN_10127
3bp8_A_ZN_410_ZN_6946
3bp8_B_ZN_407_ZN_6947
3eo3_A_ZN_801_ZN_5889
3eo3_B_ZN_801_ZN_5890
3eo3_C_ZN_801_ZN_5891
3lm9_A_ZN_302_ZN_2298
3ohr_A_ZN_302_ZN_2355

------- SF_307 --------
1nku_A_ZN_188_ZN_2921
1p7m_A_ZN_188_ZN_2923
2jg6_A_ZN_1187_ZN_1527
2ofi_A_ZN_302_ZN_1959
2ofk_A_ZN_201_ZN_2904
2ofk_B_ZN_201_ZN_2942

------- SF_308 --------
2xcm_E_ZN_1222_ZN_6063
2xcm_F_ZN_1222_ZN_6065
2yrt_A_ZN_201_ZN_1092

------- SF_309 --------
1zh1_A_ZN_199_ZN_2527
1zh1_B_ZN_199_ZN_2528
3fqm_A_ZN_901_ZN_2469
3fqm_B_ZN_902_ZN_2476
3fqq_A_ZN_901_ZN_2469
3fqq_B_ZN_902_ZN_2498

------- SF_310 --------
2b5l_C_ZN_3002_ZN_20401
2b5l_D_ZN_3004_ZN_20403

------- SF_311 --------
2eg3_A_ZN_301_ZN_3589
2eg3_B_ZN_302_ZN_3610
2eg4_A_ZN_301_ZN_3602
2eg4_B_ZN_302_ZN_3628

------- SF_312 --------
2iim_A_ZN_500_ZN_508

------- SF_313 --------
2k7r_A_ZN_129_ZN_1735

------- SF_314 --------
2kak_A_ZN_130_ZN_635

------- SF_315 --------
2xal_A_ZN_700_ZN_6787
2xal_B_ZN_700_ZN_6853
2xam_A_ZN_700_ZN_6760
2xam_B_ZN_700_ZN_6826
2xan_A_ZN_700_ZN_6656
2xan_B_ZN_700_ZN_6721
2xao_A_ZN_700_ZN_6637
2xao_B_ZN_700_ZN_6670
2xar_A_ZN_700_ZN_6716
2xar_B_ZN_700_ZN_6753

------- SF_316 --------
2xoc_A_ZN_991_ZN_3434
2xoc_B_ZN_991_ZN_3476
2xoy_A_ZN_991_ZN_3387
2xoy_B_ZN_991_ZN_3392
2xoz_A_ZN_991_ZN_3422
2xoz_B_ZN_991_ZN_3427
2xp0_A_ZN_991_ZN_3409
2xp0_B_ZN_991_ZN_3414

------- SF_317 --------
2yre_A_ZN_401_ZN_1427

------- SF_318 --------
3cg7_A_ZN_299_ZN_4825
3cg7_B_ZN_299_ZN_4826
3cm5_A_ZN_299_ZN_4795
3cm5_B_ZN_299_ZN_4797
3cm6_A_ZN_299_ZN_4809
3cm6_B_ZN_299_ZN_4811

------- SF_319 --------
1a4l_A_ZN_400_ZN_11165
1a4l_B_ZN_900_ZN_11185
1a4l_C_ZN_1400_ZN_11205
1a4l_D_ZN_1900_ZN_11225
1a4m_A_ZN_400_ZN_11173
1a4m_B_ZN_900_ZN_11193
1a4m_C_ZN_1400_ZN_11213
1a4m_D_ZN_1900_ZN_11233
1add_A_ZN_400_ZN_2794
1bf6_A_ZN_1_ZN_4598,1bf6_A_ZN_293_ZN_4599
1bf6_B_ZN_293_ZN_4624,1bf6_B_ZN_294_ZN_4625
1dpm_A_ZN_800_ZN_5027,1dpm_A_ZN_801_ZN_5028
1dpm_B_ZN_802_ZN_5064,1dpm_B_ZN_803_ZN_5065
1eyw_A_ZN_401_ZN_2527,1eyw_A_ZN_402_ZN_2528
1ez2_A_ZN_401_ZN_5035,1ez2_A_ZN_402_ZN_5036
1ez2_B_ZN_401_ZN_5048,1ez2_B_ZN_402_ZN_5049
1fkw_A_ZN_400_ZN_2793
1fkx_A_ZN_400_ZN_2789
1gkp_A_ZN_1460_ZN_21463,1gkp_A_ZN_1461_ZN_21464
1gkp_B_ZN_1460_ZN_21470,1gkp_B_ZN_1461_ZN_21471
1gkp_C_ZN_1460_ZN_21477,1gkp_C_ZN_1461_ZN_21478
1gkp_D_ZN_1460_ZN_21489,1gkp_D_ZN_1461_ZN_21490
1gkp_E_ZN_1460_ZN_21511,1gkp_E_ZN_1461_ZN_21512
1gkp_F_ZN_1460_ZN_21528,1gkp_F_ZN_1461_ZN_21529
1gkq_A_ZN_1460_ZN_14309,1gkq_A_ZN_1461_ZN_14310
1gkq_B_ZN_1460_ZN_14311,1gkq_B_ZN_1461_ZN_14312
1gkq_C_ZN_1460_ZN_14313,1gkq_C_ZN_1461_ZN_14314
1gkq_D_ZN_1460_ZN_14315,1gkq_D_ZN_1461_ZN_14316
1gkr_A_ZN_1452_ZN_13685,1gkr_A_ZN_1453_ZN_13686
1gkr_B_ZN_1452_ZN_13687,1gkr_B_ZN_1453_ZN_13688
1gkr_C_ZN_1452_ZN_13689,1gkr_C_ZN_1453_ZN_13690
1gkr_D_ZN_1452_ZN_13691,1gkr_D_ZN_1453_ZN_13692
1hzy_A_ZN_401_ZN_5103,1hzy_A_ZN_402_ZN_5104
1hzy_B_ZN_401_ZN_5162,1hzy_B_ZN_402_ZN_5163
1i0d_A_ZN_401_ZN_5097
1i0d_B_ZN_401_ZN_5149
1itq_A_ZN_401_ZN_5807,1itq_A_ZN_402_ZN_5808
1itq_B_ZN_411_ZN_5837,1itq_B_ZN_412_ZN_5838
1itu_A_ZN_401_ZN_5807,1itu_A_ZN_402_ZN_5808
1itu_B_ZN_411_ZN_5853,1itu_B_ZN_412_ZN_5854
1j79_A_ZN_400_ZN_5445,1j79_A_ZN_401_ZN_5446
1j79_B_ZN_400_ZN_5470,1j79_B_ZN_401_ZN_5471
1k1d_A_ZN_501_ZN_28489,1k1d_A_ZN_502_ZN_28490
1k1d_B_ZN_501_ZN_28491,1k1d_B_ZN_502_ZN_28492
1k1d_C_ZN_501_ZN_28493,1k1d_C_ZN_502_ZN_28494
1k1d_D_ZN_501_ZN_28495,1k1d_D_ZN_502_ZN_28496
1k1d_E_ZN_501_ZN_28497,1k1d_E_ZN_502_ZN_28498
1k1d_F_ZN_501_ZN_28499,1k1d_F_ZN_502_ZN_28500
1k1d_G_ZN_501_ZN_28501,1k1d_G_ZN_502_ZN_28502

1k1d_H_ZN_501_ZN_28503,1k1d_H_ZN_502_ZN_28504
1krm_A_ZN_501_ZN_2790
1m65_A_ZN_300_ZN_1808
1m68_A_ZN_301_ZN_1810,1m68_A_ZN_302_ZN_1811,1m68_A_ZN_303_ZN_1812
1m7j_A_ZN_801_ZN_3591,1m7j_A_ZN_802_ZN_3592
1ndv_A_ZN_400_ZN_2790
1ndw_A_ZN_400_ZN_2790
1ndy_A_ZN_400_ZN_2790
1ndz_A_ZN_400_ZN_2790
1nfg_A_ZN_501_ZN_14057,1nfg_A_ZN_502_ZN_14058
1nfg_B_ZN_601_ZN_14059,1nfg_B_ZN_602_ZN_14060
1nfg_C_ZN_701_ZN_14061,1nfg_C_ZN_702_ZN_14062
1nfg_D_ZN_801_ZN_14063,1nfg_D_ZN_802_ZN_14064
1o5r_A_ZN_400_ZN_2791
1onw_A_ZN_800_ZN_5623,1onw_A_ZN_801_ZN_5624
1onw_B_ZN_802_ZN_5635,1onw_B_ZN_803_ZN_5636
1onx_A_ZN_401_ZN_5777,1onx_A_ZN_402_ZN_5778
1onx_B_ZN_501_ZN_5788,1onx_B_ZN_502_ZN_5789
1p6b_A_ZN_401_ZN_5058,1p6b_A_ZN_402_ZN_5059
1p6b_A_ZN_406_ZN_5060
1p6b_B_ZN_403_ZN_5077,1p6b_B_ZN_404_ZN_5078
1p6b_B_ZN_405_ZN_5079
1p6c_A_ZN_401_ZN_5055,1p6c_A_ZN_402_ZN_5056
1p6c_B_ZN_403_ZN_5084,1p6c_B_ZN_404_ZN_5085
1pb0_A_ZN_1301_ZN_5439,1pb0_A_ZN_1302_ZN_5440,1pb0_A_ZN_1303_ZN_5441
1pb0_B_ZN_1311_ZN_5455,1pb0_B_ZN_1312_ZN_5456,1pb0_B_ZN_1313_ZN_5457
1pb0_C_ZN_1321_ZN_5472,1pb0_C_ZN_1322_ZN_5473,1pb0_C_ZN_1323_ZN_5474
1po9_A_ZN_601_ZN_5503,1po9_A_ZN_602_ZN_5504
1po9_B_ZN_603_ZN_5505,1po9_B_ZN_604_ZN_5506
1poj_A_ZN_601_ZN_5739,1poj_A_ZN_602_ZN_5740
1poj_B_ZN_603_ZN_5759,1poj_B_ZN_604_ZN_5760
1pok_A_ZN_601_ZN_5549,1pok_A_ZN_602_ZN_5550
1pok_B_ZN_603_ZN_5542,1pok_B_ZN_604_ZN_5543
1qxl_A_ZN_400_ZN_2790
1rjp_A_ZN_601_ZN_3599
1rjq_A_ZN_601_ZN_3596
1rjr_A_ZN_601_ZN_3596,1rjr_A_ZN_602_ZN_3597
1rk5_A_ZN_601_ZN_3596
1rk6_A_ZN_601_ZN_3605
1uio_A_ZN_400_ZN_2787
1uip_A_ZN_400_ZN_2791
1uml_A_ZN_400_ZN_2791
1v4y_A_ZN_501_ZN_3590
1v51_A_ZN_601_ZN_3599,1v51_A_ZN_602_ZN_3600
1v79_A_ZN_400_ZN_2791
1v7a_A_ZN_400_ZN_2791
1vfl_A_ZN_501_ZN_2791
1w1i_E_ZN_501_ZN_35882
1w1i_F_ZN_501_ZN_35883
1w1i_G_ZN_501_ZN_35884
1w1i_H_ZN_501_ZN_35885
1wxy_A_ZN_400_ZN_2791
1wxz_A_ZN_400_ZN_2791
1xge_A_ZN_400_ZN_5447,1xge_A_ZN_401_ZN_5448
1xge_B_ZN_400_ZN_5460,1xge_B_ZN_401_ZN_5461
1xrf_A_ZN_1423_ZN_2805
1xrt_A_ZN_1423_ZN_5702
1xrt_B_ZN_1424_ZN_5703

1ybq_A_ZN_391_ZN_5774,1ybq_A_ZN_392_ZN_5775
1ybq_B_ZN_391_ZN_5795,1ybq_B_ZN_392_ZN_5796
1yix_A_ZN_601_ZN_4203,1yix_A_ZN_604_ZN_4204
1yix_B_ZN_602_ZN_4205,1yix_B_ZN_603_ZN_4206
1zzm_A_ZN_401_ZN_2041,1zzm_A_ZN_402_ZN_2042
2a3l_A_ZN_840_ZN_5052
2ada_A_ZN_400_ZN_2794
2anu_A_ZN_405_ZN_10927,2anu_A_ZN_505_ZN_10928,2anu_A_ZN_605_ZN_10929,2anu_A_ZN_705_ZN_10930
2anu_B_ZN_405_ZN_10934,2anu_B_ZN_505_ZN_10935,2anu_B_ZN_605_ZN_10936,2anu_B_ZN_705_ZN_10937
2anu_C_ZN_405_ZN_10940,2anu_C_ZN_505_ZN_10941,2anu_C_ZN_605_ZN_10942,2anu_C_ZN_705_ZN_10943
2anu_D_ZN_405_ZN_10947,2anu_D_ZN_505_ZN_10948,2anu_D_ZN_605_ZN_10949,2anu_D_ZN_705_ZN_10950
2anu_E_ZN_405_ZN_10953,2anu_E_ZN_505_ZN_10954,2anu_E_ZN_605_ZN_10955,2anu_E_ZN_705_ZN_10956
2anu_F_ZN_405_ZN_10960,2anu_F_ZN_505_ZN_10961,2anu_F_ZN_605_ZN_10962,2anu_F_ZN_705_ZN_10963
2aqo_A_ZN_801_ZN_5582,2aqo_A_ZN_802_ZN_5583
2aqo_B_ZN_803_ZN_5584,2aqo_B_ZN_804_ZN_5585
2aqv_A_ZN_801_ZN_5562,2aqv_A_ZN_802_ZN_5563
2aqv_B_ZN_803_ZN_5564,2aqv_B_ZN_804_ZN_5565
2bb0_A_ZN_1601_ZN_6294
2bb0_B_ZN_2601_ZN_6299
2bgn_E_ZN_501_ZN_36105
2bgn_F_ZN_501_ZN_36106
2bgn_G_ZN_501_ZN_36107
2bgn_H_ZN_501_ZN_36108
2dvt_A_ZN_1501_ZN_10432
2dvt_B_ZN_1502_ZN_10433
2dvt_C_ZN_1503_ZN_10434
2dvt_D_ZN_1504_ZN_10435
2dvu_A_ZN_1501_ZN_10465
2dvu_B_ZN_1502_ZN_10477
2dvu_C_ZN_1503_ZN_10489
2dvu_D_ZN_1504_ZN_10501
2dvx_A_ZN_1501_ZN_10444
2dvx_B_ZN_1502_ZN_10455
2dvx_C_ZN_1503_ZN_10466
2dvx_D_ZN_1504_ZN_10477
2e1w_A_ZN_400_ZN_2791
2e25_A_ZN_400_ZN_2695,2e25_A_ZN_401_ZN_2696
2eg6_A_ZN_400_ZN_5368,2eg6_A_ZN_401_ZN_5369
2eg6_B_ZN_400_ZN_5370,2eg6_B_ZN_401_ZN_5371
2eg7_A_ZN_400_ZN_5399,2eg7_A_ZN_401_ZN_5400
2eg7_B_ZN_400_ZN_5414,2eg7_B_ZN_401_ZN_5415
2eg8_A_ZN_400_ZN_5350,2eg8_A_ZN_401_ZN_5351
2eg8_B_ZN_400_ZN_5364,2eg8_B_ZN_401_ZN_5365
2ftw_A_ZN_522_ZN_3820,2ftw_A_ZN_523_ZN_3821
2fty_A_ZN_601_ZN_16627,2fty_A_ZN_602_ZN_16628
2fty_B_ZN_601_ZN_16629,2fty_B_ZN_602_ZN_16630
2fty_C_ZN_601_ZN_16631,2fty_C_ZN_602_ZN_16632
2fty_D_ZN_601_ZN_16633,2fty_D_ZN_602_ZN_16634
2fvk_A_ZN_601_ZN_16620,2fvk_A_ZN_602_ZN_16621
2fvk_B_ZN_601_ZN_16630,2fvk_B_ZN_602_ZN_16631
2fvk_C_ZN_601_ZN_16640,2fvk_C_ZN_602_ZN_16641
2fvk_D_ZN_601_ZN_16650,2fvk_D_ZN_602_ZN_16651

2fvm_A_ZN_601_ZN_16617,2fvm_A_ZN_602_ZN_16618
2fvm_B_ZN_601_ZN_16628,2fvm_B_ZN_602_ZN_16629
2fvm_C_ZN_601_ZN_16639,2fvm_C_ZN_602_ZN_16640
2fvm_D_ZN_601_ZN_16641,2fvm_D_ZN_602_ZN_16642
2g3f_A_ZN_1601_ZN_6347
2g3f_B_ZN_2601_ZN_6357
2gwg_A_ZN_401_ZN_5192
2gwg_B_ZN_401_ZN_5193
2gwn_A_ZN_601_ZN_3660,2gwn_A_ZN_602_ZN_3661
2hbv_A_ZN_401_ZN_5193
2hbv_B_ZN_402_ZN_5195
2hpi_A_ZN_1221_ZN_9129,2hpi_A_ZN_1222_ZN_9130
2hpm_A_ZN_1221_ZN_9129,2hpm_A_ZN_1222_ZN_9130
2ics_A_ZN_400_ZN_2858,2ics_A_ZN_401_ZN_2859
2imr_A_ZN_500_ZN_2915
2o4m_A_ZN_3001_ZN_10282,2o4m_A_ZN_3003_ZN_10283
2o4m_B_ZN_3005_ZN_10297,2o4m_B_ZN_3006_ZN_10298
2o4m_C_ZN_3007_ZN_10317,2o4m_C_ZN_3008_ZN_10318
2o4m_P_ZN_3002_ZN_10344,2o4m_P_ZN_3004_ZN_10345
2o4q_A_ZN_2401_ZN_10166,2o4q_A_ZN_2402_ZN_10167
2o4q_B_ZN_2403_ZN_10173,2o4q_B_ZN_2404_ZN_10174
2o4q_K_ZN_2405_ZN_10180,2o4q_K_ZN_2406_ZN_10181
2o4q_P_ZN_2407_ZN_10187,2o4q_P_ZN_2408_ZN_10188
2ob3_A_ZN_901_ZN_5181,2ob3_A_ZN_902_ZN_5182
2ob3_B_ZN_903_ZN_5197,2ob3_B_ZN_904_ZN_5198
2ogj_A_ZN_418_ZN_16457,2ogj_A_ZN_419_ZN_16458
2ogj_B_ZN_500_ZN_16459,2ogj_B_ZN_501_ZN_16460
2ogj_C_ZN_600_ZN_16461,2ogj_C_ZN_601_ZN_16462
2ogj_D_ZN_700_ZN_16468,2ogj_D_ZN_701_ZN_16469
2ogj_E_ZN_800_ZN_16475,2ogj_E_ZN_801_ZN_16476
2ogj_F_ZN_900_ZN_16477,2ogj_F_ZN_901_ZN_16478
2ood_A_ZN_500_ZN_3619
2oql_A_ZN_401_ZN_5177,2oql_A_ZN_402_ZN_5178
2oql_B_ZN_401_ZN_5199,2oql_B_ZN_402_ZN_5200
2p50_A_ZN_601_ZN_10859
2p50_B_ZN_601_ZN_10860
2p50_C_ZN_601_ZN_10861
2p50_D_ZN_601_ZN_10862
2p53_A_ZN_401_ZN_5751
2p53_B_ZN_401_ZN_5772
2paj_A_ZN_493_ZN_3196
2pgf_A_ZN_401_ZN_3000
2pgr_A_ZN_401_ZN_2950
2plm_A_ZN_407_ZN_3211
2qpx_A_ZN_376_ZN_3182,2qpx_A_ZN_377_ZN_3183
2qt3_A_ZN_501_ZN_6297
2qt3_B_ZN_502_ZN_6298
2rag_A_ZN_418_ZN_11329,2rag_A_ZN_419_ZN_11330
2rag_B_ZN_418_ZN_11332,2rag_B_ZN_419_ZN_11333
2rag_C_ZN_418_ZN_11335,2rag_C_ZN_419_ZN_11336
2rag_D_ZN_418_ZN_11338,2rag_D_ZN_419_ZN_11339
2uz9_A_ZN_1452_ZN_3548
2vr2_A_ZN_1494_ZN_3695,2vr2_A_ZN_1495_ZN_3696
2vun_A_ZN_401_ZN_11145
2vun_B_ZN_401_ZN_11160
2vun_C_ZN_401_ZN_11169
2vun_D_ZN_401_ZN_11178
2w9m_A_ZN_1565_ZN_8343,2w9m_A_ZN_1566_ZN_8344,2w9m_A_ZN_1567_ZN_8345

2w9m_B_ZN_1565_ZN_8348,2w9m_B_ZN_1566_ZN_8349,2w9m_B_ZN_1567_ZN_8350
2wm1_A_ZN_1333_ZN_2637
2y1h_A_ZN_1273_ZN_3912,2y1h_A_ZN_1274_ZN_3913
2y1h_B_ZN_1273_ZN_3920,2y1h_B_ZN_1274_ZN_3921
2z00_A_ZN_1004_ZN_3226,2z00_A_ZN_1006_ZN_3227
2z24_A_ZN_400_ZN_5341,2z24_A_ZN_401_ZN_5342
2z24_B_ZN_400_ZN_5354,2z24_B_ZN_401_ZN_5355
2z25_A_ZN_400_ZN_5395,2z25_A_ZN_401_ZN_5396
2z25_B_ZN_400_ZN_5408,2z25_B_ZN_401_ZN_5409
2z26_A_ZN_400_ZN_5467,2z26_A_ZN_401_ZN_5468
2z26_B_ZN_400_ZN_5487,2z26_B_ZN_401_ZN_5488
2z27_A_ZN_400_ZN_5356,2z27_A_ZN_401_ZN_5357
2z27_B_ZN_400_ZN_5369,2z27_B_ZN_401_ZN_5370
2z28_A_ZN_400_ZN_5334,2z28_A_ZN_401_ZN_5335
2z28_B_ZN_400_ZN_5347,2z28_B_ZN_401_ZN_5348
2z29_A_ZN_400_ZN_5378,2z29_A_ZN_401_ZN_5379
2z29_B_ZN_400_ZN_5391,2z29_B_ZN_401_ZN_5392
2z2a_A_ZN_400_ZN_5317,2z2a_A_ZN_401_ZN_5318
2z2a_B_ZN_400_ZN_5330,2z2a_B_ZN_401_ZN_5331
2z2b_A_ZN_1337_ZN_2613,2z2b_A_ZN_338_ZN_2614
2z7g_A_ZN_355_ZN_2791
3cjp_A_ZN_301_ZN_4092,3cjp_A_ZN_302_ZN_4093
3cjp_B_ZN_301_ZN_4094,3cjp_B_ZN_302_ZN_4095
3d6n_A_ZN_423_ZN_5635
3dc8_A_ZN_501_ZN_7391,3dc8_A_ZN_502_ZN_7392
3dc8_B_ZN_501_ZN_7415,3dc8_B_ZN_502_ZN_7416
3dcp_A_ZN_301_ZN_6594
3dcp_B_ZN_302_ZN_6597
3dcp_C_ZN_303_ZN_6600
3dug_A_ZN_425_ZN_24158,3dug_A_ZN_428_ZN_24191
3dug_B_ZN_425_ZN_24204,3dug_B_ZN_428_ZN_24231
3dug_C_ZN_425_ZN_24244,3dug_C_ZN_428_ZN_24247
3dug_D_ZN_425_ZN_24248,3dug_D_ZN_428_ZN_24257
3dug_E_ZN_425_ZN_24258,3dug_E_ZN_428_ZN_24273
3dug_F_ZN_425_ZN_24274,3dug_F_ZN_428_ZN_24277
3dug_G_ZN_425_ZN_24278,3dug_G_ZN_428_ZN_24287
3dug_H_ZN_425_ZN_24288,3dug_H_ZN_428_ZN_24297
3e0f_A_ZN_301_ZN_2210
3e0l_A_ZN_1452_ZN_6968
3e0l_B_ZN_1452_ZN_6969
3e38_A_ZN_1_ZN_5553,3e38_A_ZN_2_ZN_5554,3e38_A_ZN_3_ZN_5555
3e38_B_ZN_4_ZN_5567,3e38_B_ZN_5_ZN_5568,3e38_B_ZN_6_ZN_5569
3ewc_A_ZN_372_ZN_2898
3ewd_A_ZN_371_ZN_2956
3f2d_A_ZN_5_ZN_8435
3fdk_A_ZN_401_ZN_2442,3fdk_A_ZN_402_ZN_2443
3feq_A_ZN_425_ZN_48257,3feq_A_ZN_426_ZN_48258
3feq_B_ZN_425_ZN_48259,3feq_B_ZN_426_ZN_48260
3feq_C_ZN_425_ZN_48261,3feq_C_ZN_426_ZN_48262
3feq_D_ZN_425_ZN_48263,3feq_D_ZN_426_ZN_48264
3feq_E_ZN_425_ZN_48265,3feq_E_ZN_426_ZN_48266
3feq_F_ZN_425_ZN_48267,3feq_F_ZN_426_ZN_48268
3feq_G_ZN_425_ZN_48269,3feq_G_ZN_426_ZN_48270
3feq_H_ZN_425_ZN_48271,3feq_H_ZN_426_ZN_48272
3feq_I_ZN_425_ZN_48273,3feq_I_ZN_426_ZN_48274
3feq_J_ZN_425_ZN_48275,3feq_J_ZN_426_ZN_48276
3feq_K_ZN_425_ZN_48277,3feq_K_ZN_426_ZN_48278
3feq_L_ZN_425_ZN_48279,3feq_L_ZN_426_ZN_48280

3feq_M_ZN_425_ZN_48281,3feq_M_ZN_426_ZN_48282
3feq_N_ZN_425_ZN_48283,3feq_N_ZN_426_ZN_48284
3feq_O_ZN_425_ZN_48285,3feq_O_ZN_426_ZN_48286
3feq_P_ZN_425_ZN_48287,3feq_P_ZN_426_ZN_48288
3gip_A_ZN_484_ZN_7123
3gip_B_ZN_484_ZN_7134
3giq_A_ZN_482_ZN_7127,3giq_A_ZN_483_ZN_7128
3giq_B_ZN_482_ZN_7143,3giq_B_ZN_483_ZN_7144
3gri_A_ZN_500_ZN_6468
3gri_B_ZN_500_ZN_6473
3guw_A_ZN_301_ZN_7453,3guw_A_ZN_302_ZN_7454
3guw_B_ZN_301_ZN_7455,3guw_B_ZN_302_ZN_7456
3guw_C_ZN_301_ZN_7457,3guw_C_ZN_302_ZN_7458
3guw_D_ZN_301_ZN_7459,3guw_D_ZN_302_ZN_7460
3hm7_A_ZN_447_ZN_20531
3hm7_B_ZN_447_ZN_20532
3hm7_C_ZN_447_ZN_20533
3hm7_D_ZN_447_ZN_20534
3hm7_E_ZN_447_ZN_20535
3hm7_F_ZN_447_ZN_20536
3hpa_A_ZN_480_ZN_6497
3hpa_B_ZN_480_ZN_6498
3icj_A_ZN_601_ZN_3786,3icj_A_ZN_602_ZN_3787
3id7_A_ZN_401_ZN_2981,3id7_A_ZN_402_ZN_2982
3ij6_A_ZN_312_ZN_9746
3ij6_B_ZN_312_ZN_9748
3ij6_C_ZN_312_ZN_9750
3ij6_D_ZN_312_ZN_9752
3isi_X_ZN_3000_ZN_3009,3isi_X_ZN_3001_ZN_3010
3itc_A_ZN_501_ZN_3056,3itc_A_ZN_502_ZN_3057
3jze_A_ZN_401_ZN_11041,3jze_A_ZN_402_ZN_11042
3jze_B_ZN_403_ZN_11066,3jze_B_ZN_404_ZN_11067
3jze_C_ZN_405_ZN_11097,3jze_C_ZN_406_ZN_11098
3jze_D_ZN_407_ZN_11122,3jze_D_ZN_408_ZN_11123
3k2g_A_ZN_400_ZN_11075,3k2g_A_ZN_401_ZN_11076
3k2g_B_ZN_400_ZN_11086,3k2g_B_ZN_401_ZN_11087
3k2g_C_ZN_400_ZN_11096,3k2g_C_ZN_401_ZN_11097
3k2g_D_ZN_400_ZN_11106,3k2g_D_ZN_401_ZN_11107
3k5x_A_ZN_402_ZN_2996,3k5x_A_ZN_403_ZN_2997
3km8_A_ZN_400_ZN_5598
3km8_B_ZN_400_ZN_5618
3lgd_A_ZN_850_ZN_7903
3lgd_B_ZN_850_ZN_7951
3lgg_A_ZN_512_ZN_7900
3lgg_B_ZN_512_ZN_7963
3ls9_A_ZN_457_ZN_6959
3ls9_B_ZN_457_ZN_6960
3lsb_A_ZN_458_ZN_6974
3lsb_B_ZN_457_ZN_6975
3lsc_A_ZN_458_ZN_6974
3lsc_B_ZN_457_ZN_6975
3ly0_A_ZN_364_ZN_5455,3ly0_A_ZN_365_ZN_5456
3ly0_B_ZN_364_ZN_5469,3ly0_B_ZN_365_ZN_5470
3mkv_A_ZN_425_ZN_25093,3mkv_A_ZN_426_ZN_25094
3mkv_B_ZN_425_ZN_25105,3mkv_B_ZN_426_ZN_25106
3mkv_C_ZN_425_ZN_25117,3mkv_C_ZN_426_ZN_25118
3mkv_D_ZN_425_ZN_25130,3mkv_D_ZN_426_ZN_25131
3mkv_E_ZN_425_ZN_25148,3mkv_E_ZN_426_ZN_25149

3mkv_F_ZN_425_ZN_25160,3mkv_F_ZN_426_ZN_25161
3mkv_G_ZN_425_ZN_25166,3mkv_G_ZN_426_ZN_25167
3mkv_H_ZN_425_ZN_25177,3mkv_H_ZN_426_ZN_25178
3mpg_A_ZN_429_ZN_6513,3mpg_A_ZN_430_ZN_6514
3mpg_B_ZN_429_ZN_6515,3mpg_B_ZN_430_ZN_6516
3mtw_A_ZN_1_ZN_3039,3mtw_A_ZN_2_ZN_3040
3mvi_A_ZN_901_ZN_5587
3mvi_B_ZN_901_ZN_5594
3n2c_A_ZN_425_ZN_48545,3n2c_A_ZN_426_ZN_48546
3n2c_B_ZN_425_ZN_48559,3n2c_B_ZN_426_ZN_48560
3n2c_C_ZN_425_ZN_48573,3n2c_C_ZN_426_ZN_48574
3n2c_D_ZN_425_ZN_48587,3n2c_D_ZN_426_ZN_48588
3n2c_E_ZN_425_ZN_48601,3n2c_E_ZN_426_ZN_48602
3n2c_F_ZN_425_ZN_48615,3n2c_F_ZN_426_ZN_48616
3n2c_G_ZN_425_ZN_48629,3n2c_G_ZN_426_ZN_48630
3n2c_H_ZN_425_ZN_48643,3n2c_H_ZN_426_ZN_48644
3n2c_I_ZN_425_ZN_48657,3n2c_I_ZN_426_ZN_48658
3n2c_J_ZN_425_ZN_48671,3n2c_J_ZN_426_ZN_48672
3n2c_K_ZN_425_ZN_48685,3n2c_K_ZN_426_ZN_48686
3n2c_L_ZN_425_ZN_48699,3n2c_L_ZN_426_ZN_48700
3n2c_M_ZN_425_ZN_48713,3n2c_M_ZN_426_ZN_48714
3n2c_N_ZN_425_ZN_48727,3n2c_N_ZN_426_ZN_48728
3n2c_O_ZN_425_ZN_48741,3n2c_O_ZN_426_ZN_48742
3n2c_P_ZN_425_ZN_48755,3n2c_P_ZN_426_ZN_48756
3neh_A_ZN_320_ZN_4956,3neh_A_ZN_321_ZN_4957
3neh_B_ZN_320_ZN_4973,3neh_B_ZN_321_ZN_4974
3o0f_A_ZN_302_ZN_2239
3ojg_A_ZN_401_ZN_2545
3ou8_A_ZN_327_ZN_5068
3ou8_B_ZN_327_ZN_5069
3ovg_A_ZN_362_ZN_16654,3ovg_A_ZN_363_ZN_16655
3ovg_B_ZN_362_ZN_16661,3ovg_B_ZN_363_ZN_16662
3ovg_C_ZN_362_ZN_16668,3ovg_C_ZN_363_ZN_16669
3ovg_D_ZN_362_ZN_16675,3ovg_D_ZN_363_ZN_16676
3ovg_E_ZN_362_ZN_16682,3ovg_E_ZN_363_ZN_16683
3ovg_F_ZN_362_ZN_16689,3ovg_F_ZN_363_ZN_16690
3pan_A_ZN_327_ZN_5068
3pan_B_ZN_327_ZN_5079
3pao_A_ZN_327_ZN_5068
3pao_B_ZN_327_ZN_5079
3pbm_A_ZN_327_ZN_5068
3pbm_B_ZN_327_ZN_5079
3pnu_A_ZN_336_ZN_5517,3pnu_A_ZN_337_ZN_5518
3pnu_B_ZN_336_ZN_5524,3pnu_B_ZN_337_ZN_5525

------- SF_320 --------
1d8w_A_ZN_450_ZN_12879
1d8w_B_ZN_450_ZN_12880
1d8w_C_ZN_450_ZN_12881
1d8w_D_ZN_450_ZN_12882
1de5_A_ZN_450_ZN_13259
1de5_B_ZN_450_ZN_13271
1de5_C_ZN_450_ZN_13283
1de5_D_ZN_450_ZN_13295
1de6_A_ZN_450_ZN_13252
1de6_B_ZN_450_ZN_13265
1de6_C_ZN_450_ZN_13278
1de6_D_ZN_450_ZN_13291

1i6n_A_ZN_401_ZN_2231
1qtw_A_ZN_301_ZN_4400,1qtw_A_ZN_302_ZN_4401,1qtw_A_ZN_303_ZN_4402
1qum_A_ZN_301_ZN_2717,1qum_A_ZN_302_ZN_2718,1qum_A_ZN_303_ZN_2719
1xll_A_ZN_398_ZN_6057,1xll_A_ZN_399_ZN_6058
1xll_B_ZN_398_ZN_6059,1xll_B_ZN_399_ZN_6060
1xp3_A_ZN_301_ZN_2313,1xp3_A_ZN_302_ZN_2314,1xp3_A_ZN_303_ZN_2315
2hcv_A_ZN_501_ZN_13015,2hcv_A_ZN_502_ZN_13016
2hcv_B_ZN_503_ZN_13017,2hcv_B_ZN_504_ZN_13018
2hcv_C_ZN_505_ZN_13019,2hcv_C_ZN_506_ZN_13020
2hcv_D_ZN_507_ZN_13021,2hcv_D_ZN_508_ZN_13022
2i56_A_ZN_501_ZN_13088,2i56_A_ZN_502_ZN_13089
2i56_B_ZN_503_ZN_13101,2i56_B_ZN_504_ZN_13102
2i56_C_ZN_505_ZN_13114,2i56_C_ZN_506_ZN_13115
2i56_D_ZN_507_ZN_13127,2i56_D_ZN_508_ZN_13128
2i57_A_ZN_501_ZN_13061,2i57_A_ZN_502_ZN_13062
2i57_B_ZN_503_ZN_13075,2i57_B_ZN_504_ZN_13076
2i57_C_ZN_505_ZN_13089,2i57_C_ZN_506_ZN_13090
2i57_D_ZN_507_ZN_13103,2i57_D_ZN_508_ZN_13104
2nq9_A_ZN_401_ZN_2609,2nq9_A_ZN_402_ZN_2610,2nq9_A_ZN_403_ZN_2611
2nqh_A_ZN_301_ZN_2168,2nqh_A_ZN_302_ZN_2169,2nqh_A_ZN_303_ZN_2170
2nqj_A_ZN_351_ZN_4952,2nqj_A_ZN_352_ZN_4953,2nqj_A_ZN_353_ZN_4954
2nqj_B_ZN_403_ZN_4955
2q02_A_ZN_300_ZN_8592
2q02_B_ZN_300_ZN_8599
2q02_C_ZN_300_ZN_8607
2q02_D_ZN_300_ZN_8614
2qw5_A_ZN_400_ZN_5350
2qw5_B_ZN_400_ZN_5362
2x7v_A_ZN_1287_ZN_2280,2x7v_A_ZN_1288_ZN_2281,2x7v_A_ZN_1289_ZN_2282
2x7w_A_ZN_1287_ZN_2280
3aal_A_ZN_303_ZN_2325
3cqj_A_ZN_285_ZN_4390
3cqj_B_ZN_285_ZN_4392
3cqk_A_ZN_285_ZN_4387
3cqk_B_ZN_285_ZN_4394
3iui_A_ZN_501_ZN_12999,3iui_A_ZN_502_ZN_13000
3iui_B_ZN_503_ZN_13001,3iui_B_ZN_504_ZN_13002
3iui_C_ZN_505_ZN_13003,3iui_C_ZN_506_ZN_13004
3iui_D_ZN_507_ZN_13005,3iui_D_ZN_508_ZN_13006
3ju2_A_ZN_285_ZN_2130

------- SF_321 --------
1b57_A_ZN_360_ZN_5251
1b57_A_ZN_361_ZN_5252
1b57_B_ZN_360_ZN_5267
1b57_B_ZN_361_ZN_5268
1dos_A_ZN_1005_ZN_6967,1dos_A_ZN_1005_ZN_6968
1dos_B_ZN_1323_ZN_6970,1dos_B_ZN_1323_ZN_6971
1gvf_A_ZN_288_ZN_4272
1gvf_B_ZN_288_ZN_4308
1zen_A_ZN_359_ZN_2568
1zen_A_ZN_360_ZN_2569
2isv_A_ZN_326_ZN_4615
2isv_B_ZN_326_ZN_4626
2isw_A_ZN_326_ZN_4551
3c4u_A_ZN_309_ZN_4524
3c4u_B_ZN_309_ZN_4526
3c52_A_ZN_308_ZN_4570

3c52_B_ZN_308_ZN_4583
3c56_A_ZN_308_ZN_4570
3c56_B_ZN_308_ZN_4589
3ekl_A_ZN_352_ZN_2740
3ekz_A_ZN_354_ZN_2611
3elf_A_ZN_352_ZN_2724
3gak_B_ZN_332_ZN_4518
3gay_A_ZN_326_ZN_4860
3gay_B_ZN_328_ZN_4881
3gb6_A_ZN_326_ZN_4837,3gb6_A_ZN_330_ZN_4858
3gb6_B_ZN_328_ZN_4859,3gb6_B_ZN_331_ZN_4880
3n9r_A_ZN_308_ZN_36876
3n9r_B_ZN_308_ZN_36894
3n9r_e_ZN_308_ZN_36984
3n9r_j_ZN_308_ZN_37002
3n9r_K_ZN_308_ZN_36912
3n9r_P_ZN_308_ZN_36930
3n9r_U_ZN_308_ZN_36948
3n9r_Z_ZN_308_ZN_36966
3n9s_A_ZN_309_ZN_9378
3n9s_B_ZN_308_ZN_9381

------- SF_322 --------
1aw5_A_ZN_400_ZN_2529
1b4e_A_ZN_400_ZN_2497
1e51_A_ZN_501_ZN_4699
1eb3_A_ZN_1342_ZN_2673
1gjp_A_ZN_400_ZN_2695
1h7n_A_ZN_1342_ZN_2666
1h7o_A_ZN_1343_ZN_2684
1h7p_A_ZN_1342_ZN_2664
1h7r_A_ZN_1344_ZN_2671
1i8j_A_ZN_400_ZN_4909
1i8j_B_ZN_400_ZN_4925
1l6s_A_ZN_400_ZN_4919
1l6s_B_ZN_400_ZN_4935
1l6y_A_ZN_400_ZN_4929
1l6y_B_ZN_400_ZN_4969
1ohl_A_ZN_400_ZN_2668
1w31_A_ZN_400_ZN_2692
1w54_A_ZN_1340_ZN_4989
1w54_B_ZN_1340_ZN_4995
1w56_A_ZN_1339_ZN_5510
1w56_B_ZN_1340_ZN_5517
1w5m_A_ZN_1341_ZN_5551
1w5m_B_ZN_1340_ZN_5559
1w5n_A_ZN_1340_ZN_5452
1w5n_B_ZN_1340_ZN_5459
1w5o_A_ZN_1339_ZN_5310
1w5o_B_ZN_1339_ZN_5319
1w5p_A_ZN_1345_ZN_5385
1w5p_B_ZN_1348_ZN_5440
1w5q_A_ZN_1339_ZN_5399
1w5q_B_ZN_1340_ZN_5408
1ylv_A_ZN_400_ZN_2652

------- SF_323 --------
1lt8_A_ZN_501_ZN_5340

1lt8_B_ZN_502_ZN_5369
1umy_A_ZN_1399_ZN_11808
1umy_B_ZN_1399_ZN_11813
1umy_C_ZN_1406_ZN_11818
1umy_D_ZN_1405_ZN_11819
3bof_A_ZN_701_ZN_8766
3bof_B_ZN_702_ZN_8777
3bol_A_ZN_701_ZN_8690
3bol_B_ZN_702_ZN_8693,3bol_B_ZN_703_ZN_8694

------- SF_324 --------
2ci6_A_ZN_1281_ZN_2111
2ci7_A_ZN_1281_ZN_2150

------- SF_325 --------
2i2x_A_ZN_501_ZN_43897
2i2x_C_ZN_502_ZN_43990
2i2x_E_ZN_503_ZN_44082
2i2x_G_ZN_504_ZN_44175
2i2x_I_ZN_505_ZN_44267
2i2x_K_ZN_506_ZN_44360
2i2x_M_ZN_507_ZN_44452
2i2x_O_ZN_508_ZN_44545

------- SF_326 --------
3fvz_A_ZN_821_ZN_2726

------- SF_327 --------
1nn7_A_ZN_201_ZN_889
1s1g_A_ZN_152_ZN_1876
1s1g_B_ZN_152_ZN_1877
2i2r_A_ZN_601_ZN_18975
2i2r_B_ZN_602_ZN_18976
2i2r_C_ZN_603_ZN_18977
2i2r_D_ZN_604_ZN_18978
2i2r_I_ZN_605_ZN_18979
2i2r_J_ZN_606_ZN_18980
2i2r_K_ZN_607_ZN_18981
2i2r_L_ZN_608_ZN_18982
2nz0_B_ZN_301_ZN_5085
2nz0_D_ZN_301_ZN_5088
3kvt_A_ZN_179_ZN_836

------- SF_328 --------
1ji3_A_ZN_402_ZN_6123
1ji3_B_ZN_404_ZN_6125
1ku0_A_ZN_701_ZN_6089
1ku0_B_ZN_702_ZN_6091
2dsn_A_ZN_2001_ZN_6109
2dsn_B_ZN_2002_ZN_6113
2hih_A_ZN_601_ZN_6146
2hih_B_ZN_1601_ZN_6148
2w22_A_ZN_402_ZN_3059
2z5g_A_ZN_2001_ZN_6103
2z5g_B_ZN_2002_ZN_6106

------- SF_329 --------
1bp3_@_ZN_500_ZN_3125

------- SF_330 --------
1mbx_A_ZN_210_ZN_3636
1mbx_B_ZN_211_ZN_3674
1r6o_A_ZN_411_ZN_3770
1r6o_B_ZN_412_ZN_3808

------- SF_331 --------
1ons_A_ZN_451_ZN_2168

------- SF_332 --------
2j0e_A_ZN_1265_ZN_3943
2j0e_B_ZN_1265_ZN_3946
3e7f_A_ZN_301_ZN_3938
3e7f_B_ZN_300_ZN_3956
3eb9_A_ZN_301_ZN_3946
3eb9_B_ZN_300_ZN_3960

------- SF_333 --------
2oo4_A_ZN_6001_ZN_3417

------- SF_334 --------
3b5q_A_ZN_500_ZN_7285
3b5q_B_ZN_500_ZN_7350

------- SF_335 --------
1f0j_A_ZN_1101_ZN_5557
1f0j_B_ZN_1201_ZN_5562
1mkd_A_ZN_2001_ZN_31813
1mkd_B_ZN_2003_ZN_31834
1mkd_C_ZN_2005_ZN_31855
1mkd_D_ZN_2007_ZN_31876
1mkd_E_ZN_2009_ZN_31897
1mkd_F_ZN_2011_ZN_31918
1mkd_G_ZN_2013_ZN_31939
1mkd_H_ZN_2015_ZN_31960
1mkd_I_ZN_2017_ZN_31981
1mkd_J_ZN_2019_ZN_32002
1mkd_K_ZN_2021_ZN_32023
1mkd_L_ZN_2023_ZN_32044
1oyn_A_ZN_501_ZN_10707,1oyn_A_ZN_502_ZN_10708
1oyn_B_ZN_503_ZN_10749,1oyn_B_ZN_504_ZN_10750
1oyn_C_ZN_505_ZN_10791,1oyn_C_ZN_506_ZN_10792
1oyn_D_ZN_507_ZN_10833,1oyn_D_ZN_508_ZN_10834
1ptw_A_ZN_501_ZN_10707,1ptw_A_ZN_502_ZN_10708
1ptw_B_ZN_501_ZN_10732,1ptw_B_ZN_502_ZN_10733
1ptw_C_ZN_501_ZN_10757,1ptw_C_ZN_502_ZN_10758
1ptw_D_ZN_501_ZN_10782,1ptw_D_ZN_502_ZN_10783
1q9m_A_ZN_501_ZN_10723,1q9m_A_ZN_502_ZN_10724
1q9m_B_ZN_503_ZN_10745,1q9m_B_ZN_504_ZN_10746
1q9m_C_ZN_505_ZN_10767,1q9m_C_ZN_506_ZN_10768
1q9m_D_ZN_507_ZN_10789,1q9m_D_ZN_508_ZN_10790
1rkp_A_ZN_501_ZN_2524
1ro6_A_ZN_529_ZN_5547
1ro6_B_ZN_529_ZN_5632
1ro9_A_ZN_529_ZN_5446,1ro9_A_ZN_530_ZN_5447
1ro9_B_ZN_529_ZN_5472,1ro9_B_ZN_530_ZN_5473
1ror_A_ZN_601_ZN_5446,1ror_A_ZN_602_ZN_5447

1ror_B_ZN_701_ZN_5471,1ror_B_ZN_702_ZN_5472
1t9r_A_ZN_1_ZN_2452
1t9s_A_ZN_1_ZN_5277
1t9s_B_ZN_1_ZN_5303
1taz_A_ZN_1001_ZN_2693
1tb5_A_ZN_1001_ZN_5237
1tb5_B_ZN_1001_ZN_5262
1tb7_A_ZN_1001_ZN_5279
1tb7_B_ZN_1001_ZN_5385
1tbb_A_ZN_1001_ZN_5306
1tbb_B_ZN_1001_ZN_5368
1tbf_A_ZN_1_ZN_2659
1udt_A_ZN_1001_ZN_2544
1udu_A_ZN_1001_ZN_5087
1udu_B_ZN_2001_ZN_5089
1uho_A_ZN_1001_ZN_2544
1xlx_A_ZN_1001_ZN_5232
1xlx_B_ZN_1001_ZN_5259
1xlz_A_ZN_1001_ZN_5229
1xlz_B_ZN_1001_ZN_5252
1xm4_A_ZN_1001_ZN_5245
1xm4_B_ZN_1001_ZN_5272
1xm6_A_ZN_1001_ZN_5470
1xm6_B_ZN_1001_ZN_5491
1xmu_A_ZN_1001_ZN_5310
1xmu_B_ZN_1001_ZN_5338
1xmy_A_ZN_1001_ZN_5229
1xmy_B_ZN_1001_ZN_5251
1xn0_A_ZN_1001_ZN_5253
1xn0_B_ZN_1001_ZN_5295
1xom_A_ZN_1001_ZN_5291
1xom_B_ZN_2001_ZN_5379
1xon_A_ZN_1001_ZN_5291
1xon_B_ZN_1001_ZN_5417
1xoq_A_ZN_1001_ZN_5331
1xoq_B_ZN_1001_ZN_5407
1xor_A_ZN_1001_ZN_5256
1xor_B_ZN_1001_ZN_5315
1xos_A_ZN_1001_ZN_2599
1xot_A_ZN_1001_ZN_5273
1xot_B_ZN_1001_ZN_5237
1xoz_A_ZN_1_ZN_2639
1xp0_A_ZN_1_ZN_2639
1y2b_A_ZN_1001_ZN_5353
1y2b_B_ZN_1001_ZN_5411
1y2c_A_ZN_1001_ZN_5310
1y2c_B_ZN_1001_ZN_5370
1y2d_A_ZN_1001_ZN_5315
1y2d_B_ZN_1001_ZN_5385
1y2e_A_ZN_1001_ZN_5261
1y2e_B_ZN_1001_ZN_5314
1y2h_A_ZN_1001_ZN_5237
1y2h_B_ZN_1001_ZN_5258
1y2j_A_ZN_1001_ZN_5237
1y2j_B_ZN_1001_ZN_5260
1y2k_A_ZN_1001_ZN_5293
1y2k_B_ZN_1001_ZN_5388
1z1l_A_ZN_3412_ZN_2746

1zkl_A_ZN_501_ZN_2586
1zkn_A_ZN_601_ZN_10731
1zkn_B_ZN_601_ZN_10749
1zkn_C_ZN_601_ZN_10767
1zkn_D_ZN_601_ZN_10785
2chm_A_ZN_1859_ZN_2613
2fm0_A_ZN_501_ZN_10707
2fm0_B_ZN_503_ZN_10747
2fm0_C_ZN_505_ZN_10787
2fm0_D_ZN_507_ZN_10827
2fm5_A_ZN_501_ZN_10707
2fm5_B_ZN_503_ZN_10747
2fm5_C_ZN_505_ZN_10787
2fm5_D_ZN_507_ZN_10827
2h40_A_ZN_501_ZN_2524
2h42_A_ZN_501_ZN_7806
2h42_B_ZN_503_ZN_7841
2h42_C_ZN_505_ZN_7876
2h44_A_ZN_501_ZN_2652
2hd1_A_ZN_101_ZN_5393
2hd1_B_ZN_101_ZN_5411
2hek_A_ZN_602_ZN_6226
2hek_B_ZN_601_ZN_6336
2o6i_A_ZN_501_ZN_7245
2o6i_B_ZN_502_ZN_7255
2o8h_A_ZN_1001_ZN_2493
2oun_A_ZN_402_ZN_5286
2oun_B_ZN_404_ZN_5311
2oup_A_ZN_777_ZN_5267
2oup_B_ZN_777_ZN_5269
2ouq_A_ZN_402_ZN_5148
2ouq_B_ZN_404_ZN_5174
2ouv_A_ZN_777_ZN_5293
2ouv_B_ZN_777_ZN_5295
2ouy_A_ZN_777_ZN_5183
2ouy_B_ZN_777_ZN_5207
2ovv_A_ZN_9001_ZN_2485
2ovy_A_ZN_9001_ZN_2500
2pw3_A_ZN_501_ZN_5283
2pw3_B_ZN_501_ZN_5306
2qgs_A_ZN_301_ZN_3366
2qgs_B_ZN_301_ZN_3367
2qyk_A_ZN_1_ZN_5435
2qyk_B_ZN_1_ZN_5465
2qyl_A_ZN_501_ZN_2708
2qym_A_ZN_1_ZN_2331
2qyn_A_ZN_501_ZN_5300
2qyn_B_ZN_501_ZN_5330
2r8q_A_ZN_1_ZN_5339
2r8q_B_ZN_1_ZN_5357
2wey_A_ZN_1772_ZN_5286
2wey_B_ZN_1772_ZN_5313
2yy2_A_ZN_101_ZN_5374
2yy2_B_ZN_103_ZN_5392
3bjc_A_ZN_876_ZN_2523
3d3p_A_ZN_1_ZN_2716
3dyn_A_ZN_901_ZN_5415
3dyn_B_ZN_901_ZN_5417

3ecm_A_ZN_1_ZN_2746
3ecn_A_ZN_1_ZN_5507
3ecn_B_ZN_1_ZN_5525
3frg_A_ZN_504_ZN_2736
3g3n_A_ZN_1_ZN_2619
3g45_A_ZN_801_ZN_5980
3g45_B_ZN_801_ZN_6008
3g4g_A_ZN_801_ZN_10897
3g4g_B_ZN_803_ZN_10928
3g4g_C_ZN_805_ZN_10959
3g4g_D_ZN_807_ZN_10990
3g4i_A_ZN_801_ZN_10660
3g4i_B_ZN_803_ZN_10704
3g4i_C_ZN_805_ZN_10748
3g4i_D_ZN_807_ZN_10786
3g4k_A_ZN_801_ZN_10629
3g4k_B_ZN_803_ZN_10688
3g4k_C_ZN_805_ZN_10722
3g4k_D_ZN_807_ZN_10773
3g4l_A_ZN_801_ZN_10594
3g4l_B_ZN_803_ZN_10639
3g4l_C_ZN_805_ZN_10680
3g4l_D_ZN_807_ZN_10717
3g58_A_ZN_624_ZN_10767
3g58_B_ZN_624_ZN_10816
3g58_C_ZN_624_ZN_10860
3g58_D_ZN_624_ZN_10904
3gwt_A_ZN_504_ZN_2753
3hc8_A_ZN_864_ZN_2664
3hdz_A_ZN_864_ZN_2641
3hmv_A_ZN_1_ZN_5685
3hmv_B_ZN_2_ZN_5718
3hqw_A_ZN_1_ZN_2463
3hqy_A_ZN_1_ZN_2500
3hqz_A_ZN_1_ZN_2500
3hr1_A_ZN_1_ZN_2530
3i8v_A_ZN_2_ZN_5424
3i8v_B_ZN_1_ZN_5453
3iad_A_ZN_620_ZN_10985
3iad_B_ZN_620_ZN_11060
3iad_C_ZN_620_ZN_11121
3iad_D_ZN_620_ZN_11167
3iak_A_ZN_1_ZN_2640
3ibj_A_ZN_1_ZN_10458
3ibj_B_ZN_2_ZN_10460
3itm_A_ZN_1_ZN_10285
3itm_B_ZN_2_ZN_10286
3itm_C_ZN_3_ZN_10287
3itm_D_ZN_4_ZN_10288
3itu_A_ZN_1_ZN_10856
3itu_B_ZN_2_ZN_10874
3itu_C_ZN_3_ZN_10892
3itu_D_ZN_4_ZN_10910
3jsi_A_ZN_901_ZN_5415
3jsi_B_ZN_901_ZN_5439
3jsw_A_ZN_901_ZN_5415
3jsw_B_ZN_901_ZN_5443
3jwq_A_ZN_899_ZN_10575

3jwq_B_ZN_899_ZN_10610
3jwq_C_ZN_899_ZN_10645
3jwq_D_ZN_899_ZN_10680
3jwr_A_ZN_899_ZN_5587
3jwr_B_ZN_899_ZN_5637
3k3e_A_ZN_601_ZN_5267
3k3e_B_ZN_601_ZN_5293
3k3h_A_ZN_601_ZN_5291
3k3h_B_ZN_601_ZN_5317
3k4s_A_ZN_1_ZN_2697
3kkt_A_ZN_1_ZN_5558
3kkt_B_ZN_2_ZN_5583
3lxg_A_ZN_761_ZN_2529
3ly2_A_ZN_1001_ZN_21558
3ly2_B_ZN_1001_ZN_21593
3ly2_C_ZN_1001_ZN_21628
3ly2_D_ZN_1001_ZN_21658
3ly2_E_ZN_1001_ZN_21688
3ly2_F_ZN_1001_ZN_21718
3ly2_G_ZN_1001_ZN_21748
3ly2_H_ZN_1001_ZN_21778

------- SF_336 --------
1dqs_A_ZN_402_ZN_5699
1dqs_B_ZN_402_ZN_5762
1jpu_A_ZN_371_ZN_2787
1jq5_A_ZN_371_ZN_2829
1jqa_A_ZN_371_ZN_2734
1kq3_A_ZN_401_ZN_2794
1nr5_A_ZN_600_ZN_5988
1nr5_B_ZN_601_ZN_6051
1nrx_A_ZN_600_ZN_5828
1nrx_B_ZN_1601_ZN_5880
1nua_A_ZN_600_ZN_5851
1nua_B_ZN_601_ZN_5852
1nva_A_ZN_600_ZN_5839
1nva_B_ZN_601_ZN_5868
1nvb_A_ZN_600_ZN_5996
1nvb_B_ZN_601_ZN_6060
1nvd_A_ZN_600_ZN_5874
1nvd_B_ZN_601_ZN_5893
1nve_A_ZN_600_ZN_11626
1nve_B_ZN_601_ZN_11672
1nve_C_ZN_602_ZN_11717
1nve_D_ZN_1603_ZN_11763
1nvf_A_ZN_600_ZN_8923
1nvf_B_ZN_601_ZN_8969
1nvf_C_ZN_602_ZN_9014
1oj7_B_ZN_1389_ZN_12383
1oj7_C_ZN_1388_ZN_12384
1oj7_D_ZN_1389_ZN_12440
1rrm_A_ZN_387_ZN_5722
1rrm_B_ZN_387_ZN_5765
1sg6_A_ZN_1600_ZN_5787
1sg6_B_ZN_1601_ZN_5832
1ta9_A_ZN_1502_ZN_5938
1ta9_B_ZN_1501_ZN_5963
1vhd_A_ZN_371_ZN_5692

1vhd_B_ZN_371_ZN_5745
1xag_A_ZN_601_ZN_2844
1xah_A_ZN_600_ZN_5233
1xah_B_ZN_601_ZN_5278
1xai_A_ZN_600_ZN_5665
1xai_B_ZN_601_ZN_5683
1xaj_A_ZN_600_ZN_5405
1xaj_B_ZN_601_ZN_5467
1xal_A_ZN_600_ZN_5381
1xal_B_ZN_601_ZN_5443
3ce9_A_ZN_400_ZN_10762
3ce9_B_ZN_400_ZN_10782
3ce9_C_ZN_400_ZN_10787
3ce9_D_ZN_400_ZN_10812

------- SF_337 --------
1ah7_A_ZN_246_ZN_2034,1ah7_A_ZN_247_ZN_2035,1ah7_A_ZN_248_ZN_2036
1ak0_A_ZN_271_ZN_2079,1ak0_A_ZN_272_ZN_2080,1ak0_A_ZN_273_ZN_2081
1ca1_A_ZN_371_ZN_3011
1gyg_A_ZN_1371_ZN_6005,1gyg_A_ZN_1372_ZN_6006
1gyg_B_ZN_1371_ZN_6007,1gyg_B_ZN_1372_ZN_6008
1kho_A_ZN_371_ZN_5993,1kho_A_ZN_372_ZN_5994
1kho_A_ZN_373_ZN_5995
1kho_B_ZN_374_ZN_5996,1kho_B_ZN_375_ZN_5997,1kho_B_ZN_376_ZN_5998
1olp_A_ZN_1373_ZN_11917,1olp_A_ZN_1375_ZN_11919
1olp_A_ZN_1374_ZN_11918
1olp_B_ZN_1373_ZN_11922,1olp_B_ZN_1375_ZN_11924
1olp_B_ZN_1374_ZN_11923
1olp_C_ZN_1373_ZN_11927,1olp_C_ZN_1375_ZN_11929
1olp_C_ZN_1374_ZN_11928
1olp_D_ZN_1373_ZN_11932,1olp_D_ZN_1374_ZN_11933,1olp_D_ZN_1375_ZN_11934
1p5x_A_ZN_246_ZN_2015,1p5x_A_ZN_247_ZN_2016,1p5x_A_ZN_248_ZN_2017
1p6d_A_ZN_246_ZN_2015,1p6d_A_ZN_247_ZN_2016,1p6d_A_ZN_248_ZN_2017
1p6e_A_ZN_246_ZN_2015,1p6e_A_ZN_247_ZN_2016,1p6e_A_ZN_248_ZN_2017
1qm6_A_ZN_400_ZN_6015,1qm6_A_ZN_401_ZN_6016
1qm6_B_ZN_400_ZN_6017,1qm6_B_ZN_401_ZN_6018
1qmd_A_ZN_401_ZN_6031,1qmd_A_ZN_402_ZN_6032
1qmd_B_ZN_401_ZN_6036,1qmd_B_ZN_402_ZN_6037
2ffz_A_ZN_246_ZN_2014,2ffz_A_ZN_247_ZN_2015,2ffz_A_ZN_248_ZN_2016
2fgn_A_ZN_246_ZN_1990,2fgn_A_ZN_247_ZN_1991,2fgn_A_ZN_248_ZN_1992
2huc_A_ZN_246_ZN_1998,2huc_A_ZN_247_ZN_1999,2huc_A_ZN_248_ZN_2000
2wxt_A_ZN_1371_ZN_2902
2wxu_A_ZN_1375_ZN_2912
2wy6_A_ZN_1371_ZN_8399
2wy6_B_ZN_1372_ZN_8411
2wy6_C_ZN_1372_ZN_8426

------- SF_338 --------
1ai0_B_ZN_31_ZN_4729
1ai0_H_ZN_31_ZN_4782
1aiy_B_ZN_31_ZN_4729
1aiy_D_ZN_31_ZN_4743
1ben_B_ZN_31_ZN_1527
1ben_D_ZN_31_ZN_1538
1ev3_B_ZN_31_ZN_793
1ev3_D_ZN_31_ZN_811
1ev6_B_ZN_301_ZN_2437
1ev6_D_ZN_302_ZN_2448

1evr_B_ZN_301_ZN_2434
1evr_D_ZN_302_ZN_2451
1fu2_B_ZN_501_ZN_1629
1fu2_D_ZN_1501_ZN_1630
1fu2_F_ZN_601_ZN_1634
1fu2_H_ZN_1601_ZN_1635
1fub_B_ZN_501_ZN_815
1fub_D_ZN_1501_ZN_816
1g7a_B_ZN_901_ZN_3302
1g7a_B_ZN_951_ZN_3303,1g7a_B_ZN_961_ZN_3304
1g7a_D_ZN_911_ZN_3306
1g7a_D_ZN_921_ZN_3307
1g7a_F_ZN_931_ZN_3312
1g7a_H_ZN_941_ZN_3348
1g7b_B_ZN_901_ZN_3197
1g7b_B_ZN_951_ZN_3198,1g7b_B_ZN_961_ZN_3199
1g7b_D_ZN_911_ZN_3201
1g7b_D_ZN_921_ZN_3202
1g7b_F_ZN_931_ZN_3207
1g7b_H_ZN_941_ZN_3234
1htv_B_ZN_1300_ZN_2299
1izb_B_ZN_101_ZN_815
1izb_D_ZN_102_ZN_816
1j73_B_ZN_31_ZN_815
1j73_D_ZN_31_ZN_816
1jca_B_ZN_41_ZN_808
1jca_D_ZN_42_ZN_809
1lph_B_ZN_31_ZN_800
1lph_D_ZN_31_ZN_808
1mpj_B_ZN_31_ZN_801
1mpj_D_ZN_31_ZN_803
1mso_D_ZN_501_ZN_1717
1mso_D_ZN_502_ZN_1718
1os3_B_ZN_501_ZN_787
1os3_D_ZN_502_ZN_789
1os4_B_ZN_1301_ZN_2272
1os4_D_ZN_1302_ZN_2273
1q4v_B_ZN_101_ZN_815
1q4v_D_ZN_102_ZN_823
1qiy_M_ZN_1_ZN_2514
1qiy_M_ZN_2_ZN_2515
1qiz_M_ZN_1_ZN_2514
1qiz_M_ZN_2_ZN_2515
1qj0_E_ZN_1_ZN_804
1qj0_E_ZN_2_ZN_805
1rwe_B_ZN_201_ZN_828
1rwe_D_ZN_301_ZN_837
1trz_B_ZN_31_ZN_803
1trz_D_ZN_31_ZN_805
1tyl_B_ZN_31_ZN_796
1tyl_D_ZN_31_ZN_808
1tym_B_ZN_31_ZN_784
1tym_D_ZN_31_ZN_796
1uz9_B_ZN_1030_ZN_451
1w8p_B_ZN_1030_ZN_2365
1w8p_D_ZN_1030_ZN_2373
1wav_B_ZN_31_ZN_2438
1wav_D_ZN_31_ZN_2446

1xda_B_ZN_30_ZN_1626
1xda_D_ZN_30_ZN_1650
1xda_F_ZN_30_ZN_1674
1xda_H_ZN_30_ZN_1698
1xw7_B_ZN_101_ZN_824
1xw7_D_ZN_201_ZN_833
1zeg_B_ZN_31_ZN_834
1zeg_D_ZN_31_ZN_850
1zeh_B_ZN_31_ZN_850
1zeh_D_ZN_31_ZN_860
1zei_B_ZN_54_ZN_2525
1zei_C_ZN_54_ZN_2535
1zni_B_ZN_31_ZN_825
1zni_B_ZN_32_ZN_826
1zni_D_ZN_31_ZN_830
1znj_B_ZN_31_ZN_2391
1znj_D_ZN_31_ZN_2407
2a3g_B_ZN_501_ZN_815
2a3g_D_ZN_502_ZN_816
2ins_B_ZN_1_ZN_783
2ins_D_ZN_31_ZN_784
2oly_B_ZN_302_ZN_2375
2oly_H_ZN_301_ZN_2436
2olz_B_ZN_402_ZN_2388
2olz_H_ZN_401_ZN_2416
2om0_B_ZN_801_ZN_7195
2om0_b_ZN_806_ZN_7335
2om0_D_ZN_802_ZN_7209
2om0_h_ZN_805_ZN_7369
2om0_R_ZN_803_ZN_7271
2om0_Y_ZN_804_ZN_7305
2om1_B_ZN_801_ZN_7192
2om1_b_ZN_806_ZN_7310
2om1_D_ZN_802_ZN_7204
2om1_h_ZN_805_ZN_7338
2om1_R_ZN_803_ZN_7248
2om1_Y_ZN_804_ZN_7282
2omg_B_ZN_201_ZN_1194
2omh_B_ZN_201_ZN_1193
2omi_B_ZN_202_ZN_2370
2omi_H_ZN_201_ZN_2396
2qiu_B_ZN_101_ZN_837
2qiu_D_ZN_102_ZN_838
2tci_B_ZN_201_ZN_797
2tci_D_ZN_31_ZN_798
2vjz_B_ZN_1030_ZN_808
2vjz_D_ZN_1029_ZN_811
2vk0_B_ZN_1031_ZN_771
2vk0_D_ZN_1030_ZN_794
2vk0_D_ZN_1031_ZN_795,2vk0_D_ZN_1032_ZN_796
2w44_B_ZN_1030_ZN_1126
2ws6_J_ZN_1030_ZN_2526
2ws6_L_ZN_1029_ZN_2535
2ws7_B_ZN_1020_ZN_1981
2ws7_D_ZN_1024_ZN_1990
2zp6_B_ZN_31_ZN_805
2zp6_D_ZN_31_ZN_806
3brr_B_ZN_31_ZN_866

3brr_B_ZN_32_ZN_867
3brr_D_ZN_31_ZN_868
3bxq_B_ZN_101_ZN_1599
3bxq_D_ZN_102_ZN_1600
3e7y_B_ZN_30_ZN_806
3e7y_D_ZN_30_ZN_808
3e7z_B_ZN_30_ZN_795
3e7z_D_ZN_30_ZN_797
3fhp_B_ZN_31_ZN_1667
3fhp_D_ZN_31_ZN_1668
3fq9_B_ZN_101_ZN_1606
3fq9_D_ZN_102_ZN_1607
3gky_B_ZN_31_ZN_827
3gky_D_ZN_31_ZN_836
3ins_B_ZN_31_ZN_1622
3ins_D_ZN_31_ZN_1623
3jsd_B_ZN_31_ZN_817
3jsd_D_ZN_31_ZN_826
3kq6_A_ZN_301_ZN_823
3kq6_B_ZN_101_ZN_825
3kq6_D_ZN_201_ZN_833
3mth_B_ZN_31_ZN_805
3mth_D_ZN_31_ZN_807
4ins_B_ZN_31_ZN_835
4ins_D_ZN_31_ZN_836
6ins_E_ZN_30_ZN_791
6ins_F_ZN_30_ZN_792
7ins_D_ZN_31_ZN_1373

------- SF_339 --------
1kae_A_ZN_1101_ZN_6497
1kae_B_ZN_1102_ZN_6570
1kah_A_ZN_501_ZN_6435
1kah_B_ZN_502_ZN_6436
1kar_A_ZN_501_ZN_6423
1kar_B_ZN_502_ZN_6424

------- SF_340 --------
2x3y_A_ZN_1196_ZN_11441
2x3y_B_ZN_1196_ZN_11442
2x3y_C_ZN_1196_ZN_11443
2x3y_D_ZN_1196_ZN_11444
2x3y_E_ZN_1196_ZN_11445
2x3y_F_ZN_1196_ZN_11446
2x3y_G_ZN_1196_ZN_11447
2x3y_H_ZN_1196_ZN_11448
2xbl_A_ZN_1197_ZN_5755
2xbl_B_ZN_1196_ZN_5787
2xbl_C_ZN_1196_ZN_5806
2xbl_D_ZN_1198_ZN_5835

------- SF_341 --------
2zws_A_ZN_647_ZN_5238
2zxc_A_ZN_647_ZN_9945
2zxc_B_ZN_647_ZN_9972

------- SF_342 --------
3fns_A_ZN_329_ZN_5183

3fns_B_ZN_329_ZN_5187

------- SF_343 --------
1d8d_A_ZN_1001_ZN_6040
1dce_B_ZN_900_ZN_14156
1dce_D_ZN_950_ZN_14157
1fpp_B_ZN_438_ZN_5845
1ft1_B_ZN_1001_ZN_5953
1ft2_B_ZN_1001_ZN_5842
1jcq_B_ZN_1001_ZN_5928
1jcr_B_ZN_1001_ZN_5989
1jcs_B_ZN_1001_ZN_6001
1kzo_B_ZN_1001_ZN_6002
1kzp_B_ZN_1001_ZN_5987
1ld7_B_ZN_1001_ZN_5903
1ld8_B_ZN_1001_ZN_5911
1ltx_B_ZN_900_ZN_10759
1mzc_B_ZN_1001_ZN_5903
1n4p_B_ZN_378_ZN_32205
1n4p_D_ZN_378_ZN_32237
1n4p_F_ZN_378_ZN_32268
1n4p_H_ZN_378_ZN_32300
1n4p_J_ZN_378_ZN_32331
1n4p_L_ZN_378_ZN_32363
1n4q_B_ZN_378_ZN_32241
1n4q_D_ZN_378_ZN_32272
1n4q_F_ZN_378_ZN_32303
1n4q_H_ZN_378_ZN_32335
1n4q_J_ZN_378_ZN_32366
1n4q_L_ZN_378_ZN_32398
1n4r_B_ZN_378_ZN_32335
1n4r_D_ZN_378_ZN_32354
1n4r_F_ZN_378_ZN_32373
1n4r_H_ZN_378_ZN_32393
1n4r_J_ZN_378_ZN_32412
1n4r_L_ZN_378_ZN_32432
1n4s_B_ZN_378_ZN_32338
1n4s_D_ZN_378_ZN_32370
1n4s_F_ZN_378_ZN_32401
1n4s_H_ZN_378_ZN_32433
1n4s_J_ZN_378_ZN_32464
1n4s_L_ZN_378_ZN_32496
1n94_B_ZN_2_ZN_5817
1n95_B_ZN_500_ZN_5817
1n9a_B_ZN_500_ZN_5817
1ni1_B_ZN_500_ZN_5817
1nl4_B_ZN_500_ZN_5818
1o1r_B_ZN_1001_ZN_5836
1o1s_B_ZN_1001_ZN_5836
1o1t_B_ZN_2001_ZN_5837
1o5m_B_ZN_1001_ZN_5836
1qbq_B_ZN_1000_ZN_5853
1s63_B_ZN_1001_ZN_5937
1s64_B_ZN_378_ZN_32082
1s64_D_ZN_378_ZN_32130
1s64_F_ZN_378_ZN_32178
1s64_H_ZN_378_ZN_32227
1s64_J_ZN_378_ZN_32276

1s64_L_ZN_378_ZN_32325
1sa4_B_ZN_439_ZN_5935
1sa5_B_ZN_438_ZN_5951
1tn6_B_ZN_1001_ZN_6020
1tn7_B_ZN_1001_ZN_6001
1tn8_B_ZN_1001_ZN_5984
1tnb_B_ZN_378_ZN_32447
1tnb_D_ZN_378_ZN_32477
1tnb_F_ZN_378_ZN_32508
1tnb_H_ZN_378_ZN_32551
1tnb_J_ZN_378_ZN_32582
1tnb_L_ZN_378_ZN_32612
1tno_B_ZN_378_ZN_32425
1tno_D_ZN_378_ZN_32467
1tno_F_ZN_378_ZN_32498
1tno_H_ZN_378_ZN_32541
1tno_J_ZN_378_ZN_32572
1tno_L_ZN_378_ZN_32602
1tnu_B_ZN_378_ZN_32465
1tnu_D_ZN_378_ZN_32495
1tnu_F_ZN_378_ZN_32526
1tnu_H_ZN_378_ZN_32569
1tnu_J_ZN_378_ZN_32600
1tnu_L_ZN_378_ZN_32630
1tny_B_ZN_378_ZN_32382
1tny_D_ZN_378_ZN_32413
1tny_F_ZN_378_ZN_32444
1tny_H_ZN_378_ZN_32487
1tny_J_ZN_378_ZN_32518
1tny_L_ZN_378_ZN_32549
1tnz_B_ZN_378_ZN_32441
1tnz_D_ZN_378_ZN_32471
1tnz_F_ZN_378_ZN_32502
1tnz_H_ZN_378_ZN_32545
1tnz_J_ZN_378_ZN_32576
1tnz_L_ZN_378_ZN_32606
1x81_B_ZN_2_ZN_5817
2bed_B_ZN_1_ZN_5822
2f0y_B_ZN_501_ZN_5786
2h6f_B_ZN_1001_ZN_5978
2h6g_B_ZN_1001_ZN_5959
2h6h_B_ZN_1001_ZN_5999
2h6i_B_ZN_1001_ZN_5958
2iej_B_ZN_939_ZN_5951
2r2l_B_ZN_1_ZN_5836
2zir_B_ZN_901_ZN_5849
2zis_B_ZN_901_ZN_5832
3c72_B_ZN_332_ZN_5065
3dpy_B_ZN_1001_ZN_5975
3dra_B_ZN_391_ZN_5604
3dss_B_ZN_332_ZN_5128
3dst_B_ZN_332_ZN_5087
3dsu_B_ZN_332_ZN_5050
3dsv_B_ZN_332_ZN_5096
3dsw_B_ZN_332_ZN_5098
3dsx_B_ZN_332_ZN_5092
3e30_B_ZN_1001_ZN_5951
3e32_B_ZN_1001_ZN_5951

3e33_B_ZN_1001_ZN_5951
3e34_B_ZN_1001_ZN_5951
3e37_B_ZN_1001_ZN_5914
3eu5_B_ZN_428_ZN_5845
3euv_B_ZN_428_ZN_5850
3hxb_B_ZN_333_ZN_4967
3hxc_B_ZN_332_ZN_5050
3hxd_B_ZN_332_ZN_5065
3hxe_B_ZN_332_ZN_4970
3hxf_B_ZN_332_ZN_4911
3ksl_B_ZN_2001_ZN_5853
3ksq_B_ZN_2001_ZN_5853

------- SF_344 --------
2g02_A_ZN_416_ZN_3224
2g0d_A_ZN_416_ZN_3224
3e6u_A_ZN_501_ZN_12977
3e6u_B_ZN_503_ZN_12979
3e6u_C_ZN_502_ZN_12978
3e6u_D_ZN_504_ZN_12980
3e73_A_ZN_501_ZN_6439
3e73_B_ZN_502_ZN_6460

------- SF_345 --------
1a71_A_ZN_401_ZN_5575
1a71_B_ZN_401_ZN_5627
1a72_A_ZN_376_ZN_2788
1adb_A_ZN_375_ZN_6786
1adb_B_ZN_375_ZN_6845
1adc_A_ZN_375_ZN_6786
1adc_B_ZN_375_ZN_6845
1adf_A_ZN_376_ZN_3394
1adg_A_ZN_376_ZN_3394
1agn_A_ZN_376_ZN_11162
1agn_B_ZN_376_ZN_11221
1agn_C_ZN_376_ZN_11286
1agn_D_ZN_376_ZN_11337
1axe_A_ZN_401_ZN_5579
1axe_B_ZN_401_ZN_5631
1axg_A_ZN_401_ZN_11137
1axg_B_ZN_401_ZN_11189
1axg_C_ZN_401_ZN_11241
1axg_D_ZN_401_ZN_11293
1bto_A_ZN_375_ZN_11145
1bto_B_ZN_375_ZN_11201
1bto_C_ZN_375_ZN_11257
1bto_D_ZN_375_ZN_11313
1cdo_A_ZN_376_ZN_5609
1cdo_B_ZN_376_ZN_5655
1d1s_A_ZN_376_ZN_11162
1d1s_B_ZN_376_ZN_11233
1d1s_C_ZN_376_ZN_11310
1d1s_D_ZN_376_ZN_11365
1d1t_A_ZN_376_ZN_11162
1d1t_B_ZN_376_ZN_11233
1d1t_C_ZN_376_ZN_11310
1d1t_D_ZN_376_ZN_11365
1deh_A_ZN_376_ZN_5566

1deh_B_ZN_376_ZN_5618
1e3e_A_ZN_378_ZN_5625
1e3e_B_ZN_378_ZN_5671
1e3i_A_ZN_380_ZN_5636
1e3i_B_ZN_380_ZN_5691
1e3j_A_ZN_902_ZN_2589
1e3l_A_ZN_380_ZN_5633
1e3l_B_ZN_380_ZN_5679
1ee2_A_ZN_1300_ZN_5782
1ee2_B_ZN_1302_ZN_5857
1f8f_A_ZN_372_ZN_2674
1h2b_A_ZN_1362_ZN_5388
1h2b_B_ZN_1362_ZN_5445
1hdx_A_ZN_376_ZN_5566
1hdx_B_ZN_376_ZN_5619
1hdy_A_ZN_376_ZN_5564
1hdy_B_ZN_376_ZN_5616
1hdz_A_ZN_376_ZN_5552
1hdz_B_ZN_376_ZN_5598
1het_A_ZN_400_ZN_6109
1het_B_ZN_400_ZN_6181
1hld_A_ZN_375_ZN_5573
1hld_B_ZN_775_ZN_5641
1hso_A_ZN_1376_ZN_5554
1hso_B_ZN_2376_ZN_5606
1hsz_A_ZN_1376_ZN_5566
1hsz_B_ZN_2376_ZN_5612
1ht0_A_ZN_1376_ZN_5560
1ht0_B_ZN_2376_ZN_5606
1htb_A_ZN_376_ZN_5556
1htb_B_ZN_376_ZN_5608
1jqb_A_ZN_1353_ZN_10594
1jqb_B_ZN_2353_ZN_10595
1jqb_C_ZN_3353_ZN_10596
1jqb_D_ZN_4353_ZN_10597
1ju9_A_ZN_375_ZN_5569
1ju9_B_ZN_375_ZN_5598
1jvb_A_ZN_500_ZN_2624
1kev_A_ZN_353_ZN_10565
1kev_B_ZN_353_ZN_10614
1kev_C_ZN_353_ZN_10663
1kev_D_ZN_353_ZN_10712
1kol_A_ZN_1001_ZN_5871
1kol_B_ZN_1003_ZN_5922
1lde_A_ZN_375_ZN_11145
1lde_B_ZN_375_ZN_11199
1lde_C_ZN_375_ZN_11253
1lde_D_ZN_375_ZN_11307
1ldy_A_ZN_375_ZN_11145
1ldy_B_ZN_375_ZN_11200
1ldy_C_ZN_375_ZN_11255
1ldy_D_ZN_375_ZN_11310
1llu_A_ZN_343_ZN_20129
1llu_B_ZN_343_ZN_20183
1llu_C_ZN_343_ZN_20233
1llu_D_ZN_343_ZN_20287
1llu_E_ZN_343_ZN_20337
1llu_F_ZN_343_ZN_20391

1llu_G_ZN_343_ZN_20441
1llu_H_ZN_343_ZN_20495
1m6h_A_ZN_1376_ZN_5548
1m6h_B_ZN_1376_ZN_5566
1m6w_A_ZN_1376_ZN_5548
1m6w_B_ZN_1376_ZN_5581
1ma0_A_ZN_5376_ZN_5548
1ma0_B_ZN_6376_ZN_5619
1mc5_A_ZN_376_ZN_5548
1mc5_B_ZN_376_ZN_5627
1mg0_A_ZN_375_ZN_11141
1mg0_B_ZN_375_ZN_11206
1mg0_C_ZN_375_ZN_11271
1mg0_D_ZN_375_ZN_11336
1mgo_A_ZN_375_ZN_5563
1mgo_B_ZN_375_ZN_5609
1mp0_A_ZN_376_ZN_5548
1mp0_B_ZN_376_ZN_5605
1n8k_A_ZN_375_ZN_5608
1n8k_B_ZN_375_ZN_5667
1n92_A_ZN_375_ZN_5573
1n92_B_ZN_375_ZN_5633
1nto_A_ZN_500_ZN_15717
1nto_B_ZN_500_ZN_15719
1nto_C_ZN_500_ZN_15721
1nto_D_ZN_500_ZN_15723
1nto_E_ZN_500_ZN_15725
1nto_H_ZN_500_ZN_15727
1nvg_A_ZN_500_ZN_2623
1p0c_A_ZN_1502_ZN_5460
1p0c_B_ZN_2502_ZN_5473
1p0f_A_ZN_1502_ZN_5457
1p0f_B_ZN_1503_ZN_5513
1p1r_A_ZN_375_ZN_11145
1p1r_B_ZN_375_ZN_11201
1p1r_C_ZN_375_ZN_11265
1p1r_D_ZN_375_ZN_11321
1ped_A_ZN_352_ZN_10565
1ped_B_ZN_352_ZN_10566
1ped_C_ZN_352_ZN_10567
1ped_D_ZN_352_ZN_10568
1piw_A_ZN_1502_ZN_5564
1piw_B_ZN_1502_ZN_5566
1pl6_A_ZN_402_ZN_10869
1pl6_B_ZN_402_ZN_10934
1pl6_C_ZN_402_ZN_10999
1pl6_D_ZN_402_ZN_11064
1pl7_A_ZN_402_ZN_10853
1pl7_B_ZN_402_ZN_10854
1pl7_C_ZN_402_ZN_10855
1pl7_D_ZN_402_ZN_10856
1pl8_A_ZN_402_ZN_10933
1pl8_B_ZN_402_ZN_10978
1pl8_C_ZN_402_ZN_11023
1pl8_D_ZN_402_ZN_11068
1ps0_A_ZN_1502_ZN_2783
1q1n_A_ZN_1502_ZN_2783
1qlh_A_ZN_375_ZN_2787

1qlj_A_ZN_375_ZN_2787
1qv6_A_ZN_375_ZN_5575
1qv6_B_ZN_375_ZN_5639
1qv7_A_ZN_375_ZN_5575
1qv7_B_ZN_375_ZN_5631
1r37_A_ZN_500_ZN_5279
1r37_B_ZN_500_ZN_5331
1rjw_A_ZN_402_ZN_10222
1rjw_B_ZN_402_ZN_10236
1rjw_C_ZN_402_ZN_10262
1rjw_D_ZN_402_ZN_10282
1teh_A_ZN_376_ZN_5548
1teh_B_ZN_376_ZN_5594
1u3t_A_ZN_376_ZN_5554
1u3t_B_ZN_376_ZN_5612
1u3u_A_ZN_376_ZN_5566
1u3u_B_ZN_376_ZN_5622
1u3v_A_ZN_376_ZN_5566
1u3v_B_ZN_376_ZN_5622
1u3w_A_ZN_376_ZN_5560
1u3w_B_ZN_376_ZN_5617
1uuf_A_ZN_402_ZN_2586
1vj0_A_ZN_401_ZN_11308
1vj0_B_ZN_401_ZN_11313
1vj0_C_ZN_401_ZN_11317
1vj0_D_ZN_401_ZN_11323
1y9a_A_ZN_1000_ZN_5423
1y9a_C_ZN_2000_ZN_5439
1ye3_A_ZN_375_ZN_2787
1ykf_A_ZN_354_ZN_10577
1ykf_B_ZN_354_ZN_10626
1ykf_C_ZN_354_ZN_10675
1ykf_D_ZN_354_ZN_10724
1yqd_A_ZN_1000_ZN_5437
1yqd_B_ZN_4000_ZN_5496
1yqx_A_ZN_1000_ZN_5437
1yqx_B_ZN_4000_ZN_5488
2b5w_A_ZN_800_ZN_2791
2b83_A_ZN_1353_ZN_10557
2b83_B_ZN_2353_ZN_10558
2b83_C_ZN_3353_ZN_10559
2b83_D_ZN_4353_ZN_10560
2cd9_A_ZN_1367_ZN_5710,2cd9_A_ZN_1367_ZN_5711
2cd9_B_ZN_1367_ZN_5713,2cd9_B_ZN_1367_ZN_5714
2cda_A_ZN_1368_ZN_5708
2cda_B_ZN_1368_ZN_5758
2cdb_A_ZN_1370_ZN_11656
2cdb_B_ZN_1370_ZN_11722
2cdb_C_ZN_1370_ZN_11788
2cdb_D_ZN_1370_ZN_11855
2cdc_A_ZN_1371_ZN_11840
2cdc_B_ZN_1371_ZN_11914
2cdc_C_ZN_1371_ZN_11988
2cdc_D_ZN_1372_ZN_12072
2cf5_A_ZN_402_ZN_2668
2cf6_A_ZN_402_ZN_2668
2d8a_A_ZN_501_ZN_2542
2dph_A_ZN_1002_ZN_6020

2dph_B_ZN_1004_ZN_6066
2dq4_A_ZN_501_ZN_5141
2dq4_B_ZN_503_ZN_5155
2ejv_A_ZN_501_ZN_5132
2ejv_B_ZN_501_ZN_5178
2fze_A_ZN_376_ZN_5558,2fze_A_ZN_376_ZN_5559
2fze_B_ZN_376_ZN_5608,2fze_B_ZN_376_ZN_5609
2fzw_A_ZN_376_ZN_5665
2fzw_B_ZN_376_ZN_5717
2h6e_A_ZN_400_ZN_2485
2hcy_A_ZN_348_ZN_10333
2hcy_B_ZN_348_ZN_10386
2hcy_C_ZN_348_ZN_10394
2hcy_D_ZN_348_ZN_10447
2jhg_A_ZN_401_ZN_6148
2jhg_B_ZN_401_ZN_6216
2nvb_A_ZN_354_ZN_10581
2nvb_B_ZN_1354_ZN_10582
2nvb_C_ZN_2354_ZN_10583
2nvb_D_ZN_3354_ZN_10584
2ohx_A_ZN_401_ZN_5573
2ohx_B_ZN_401_ZN_5623
2oui_A_ZN_361_ZN_11000
2oui_B_ZN_361_ZN_11040
2oui_C_ZN_361_ZN_11082
2oui_D_ZN_361_ZN_11118
2vwg_A_ZN_800_ZN_2832
2vwh_A_ZN_800_ZN_2832
2vwp_A_ZN_800_ZN_2820
2vwq_A_ZN_800_ZN_2820
2xaa_A_ZN_1346_ZN_9783
2xaa_B_ZN_1346_ZN_9829
2xaa_C_ZN_1346_ZN_9881
2xaa_D_ZN_1346_ZN_9933
3bto_A_ZN_375_ZN_11145
3bto_B_ZN_375_ZN_11201
3bto_C_ZN_375_ZN_11257
3bto_D_ZN_375_ZN_11313
3cos_A_ZN_501_ZN_11232
3cos_B_ZN_501_ZN_11290
3cos_C_ZN_501_ZN_11352
3cos_D_ZN_501_ZN_11406
3fpc_A_ZN_353_ZN_10964
3fpc_B_ZN_353_ZN_10972
3fpc_C_ZN_353_ZN_11002
3fpc_D_ZN_353_ZN_11038
3fpl_A_ZN_352_ZN_2671
3fsr_A_ZN_353_ZN_10697
3fsr_C_ZN_353_ZN_10716
3ftn_A_ZN_354_ZN_10609
3ftn_B_ZN_355_ZN_10635
3ftn_C_ZN_353_ZN_10663
3ftn_D_ZN_357_ZN_10685
3hud_A_ZN_376_ZN_5566
3hud_B_ZN_376_ZN_5568
3i4c_A_ZN_500_ZN_14712
3i4c_B_ZN_500_ZN_14714
3i4c_C_ZN_500_ZN_14716

3i4c_D_ZN_500_ZN_14718
3i4c_E_ZN_500_ZN_14720
3i4c_H_ZN_500_ZN_14722
3jv7_A_ZN_502_ZN_9922
3jv7_B_ZN_502_ZN_9980
3jv7_C_ZN_502_ZN_10038
3jv7_D_ZN_502_ZN_10096
3m6i_A_ZN_401_ZN_5419
3m6i_B_ZN_401_ZN_5465
3meq_A_ZN_401_ZN_10052
3meq_B_ZN_401_ZN_10111
3meq_C_ZN_401_ZN_10145
3meq_D_ZN_401_ZN_10157
3oq6_A_ZN_375_ZN_5735
3oq6_B_ZN_375_ZN_5810
5adh_A_ZN_375_ZN_2787
6adh_A_ZN_375_ZN_5572
6adh_B_ZN_375_ZN_5574
7adh_A_ZN_375_ZN_2786
8adh_A_ZN_376_ZN_2787

------- SF_346 --------
1j3g_A_ZN_210_ZN_2877
1lba_A_ZN_151_ZN_1152
1oht_A_ZN_1185_ZN_1370
1yb0_A_ZN_160_ZN_3775
1yb0_B_ZN_160_ZN_3781
1yb0_C_ZN_160_ZN_3787
2ar3_A_ZN_161_ZN_3763
2ar3_B_ZN_161_ZN_3769
2ar3_C_ZN_161_ZN_3775
2bh7_A_ZN_300_ZN_2031
2wkx_A_ZN_300_ZN_2087
3d2z_A_ZN_262_ZN_2074
3hma_A_ZN_190_ZN_2357
3hma_B_ZN_190_ZN_2358
3hmb_A_ZN_155_ZN_3599
3hmb_B_ZN_155_ZN_3600
3hmb_C_ZN_155_ZN_3601
3lat_A_ZN_215_ZN_3417,3lat_A_ZN_216_ZN_3418
3lat_B_ZN_215_ZN_3435,3lat_B_ZN_216_ZN_3436

------- SF_347 --------
1azv_A_ZN_155_ZN_2238
1azv_B_ZN_155_ZN_2240
1b4l_A_ZN_155_ZN_1109
1b4t_A_ZN_155_ZN_1105
1ba9_A_ZN_154_ZN_2194
1bzo_A_ZN_151_ZN_1111
1cb4_B_ZN_153_ZN_2177
1cbj_A_ZN_153_ZN_2170
1cbj_B_ZN_153_ZN_2172
1do5_A_ZN_28_ZN_4470
1do5_B_ZN_29_ZN_4471
1do5_C_ZN_30_ZN_4472
1do5_D_ZN_27_ZN_4473
1e9p_B_ZN_153_ZN_2146
1e9q_B_ZN_153_ZN_2171

1eqw_A_ZN_501_ZN_4450
1eqw_B_ZN_503_ZN_4452
1eqw_C_ZN_505_ZN_4454
1eqw_D_ZN_507_ZN_4456
1eso_A_ZN_149_ZN_1105
1f18_A_ZN_155_ZN_1116
1f1a_A_ZN_155_ZN_1108
1f1g_A_ZN_4002_ZN_6644
1f1g_B_ZN_4004_ZN_6646
1f1g_C_ZN_4006_ZN_6648
1f1g_D_ZN_4008_ZN_6650
1f1g_E_ZN_4010_ZN_6652
1f1g_F_ZN_4012_ZN_6654
1fun_A_ZN_155_ZN_11102
1fun_B_ZN_155_ZN_11111
1fun_C_ZN_155_ZN_11115
1fun_D_ZN_155_ZN_11119
1fun_E_ZN_155_ZN_11123
1fun_F_ZN_155_ZN_11104
1fun_G_ZN_155_ZN_11113
1fun_H_ZN_155_ZN_11117
1fun_I_ZN_155_ZN_11121
1fun_J_ZN_155_ZN_11125
1hl4_A_ZN_155_ZN_4002
1hl4_B_ZN_155_ZN_4003
1hl5_A_ZN_155_ZN_19803
1hl5_B_ZN_155_ZN_19805
1hl5_C_ZN_155_ZN_19807
1hl5_D_ZN_155_ZN_19810
1hl5_E_ZN_155_ZN_19813
1hl5_F_ZN_155_ZN_19815
1hl5_G_ZN_155_ZN_19817
1hl5_H_ZN_155_ZN_19819
1hl5_I_ZN_155_ZN_19821
1hl5_J_ZN_155_ZN_19823
1hl5_K_ZN_155_ZN_19825
1hl5_L_ZN_155_ZN_19827
1hl5_M_ZN_155_ZN_19829
1hl5_N_ZN_155_ZN_19831
1hl5_O_ZN_155_ZN_19833
1hl5_P_ZN_155_ZN_19836
1hl5_Q_ZN_155_ZN_19838
1hl5_S_ZN_155_ZN_19840
1ib5_A_ZN_201_ZN_1110
1ibb_A_ZN_201_ZN_1109
1ibd_A_ZN_201_ZN_1110
1ibf_A_ZN_201_ZN_1109
1ibh_A_ZN_201_ZN_1105
1jcv_A_ZN_155_ZN_1109
1jk9_A_ZN_301_ZN_5959
1jk9_C_ZN_302_ZN_5966
1kmg_A_ZN_154_ZN_2194
1l3n_A_ZN_155_ZN_4378
1l3n_B_ZN_155_ZN_4380
1mfm_A_ZN_154_ZN_1154
1n18_A_ZN_155_ZN_11102
1n18_B_ZN_155_ZN_11104
1n18_C_ZN_155_ZN_11106

1n18_D_ZN_155_ZN_11108
1n18_E_ZN_155_ZN_11115
1n18_F_ZN_155_ZN_11117
1n18_G_ZN_155_ZN_11119
1n18_H_ZN_155_ZN_11121
1n18_I_ZN_155_ZN_11123
1n18_J_ZN_155_ZN_11125
1n19_A_ZN_155_ZN_2226
1n19_B_ZN_155_ZN_2228
1oaj_A_ZN_1152_ZN_1110
1oal_A_ZN_152_ZN_1113
1oez_W_ZN_1154_ZN_3581
1oez_X_ZN_1154_ZN_3587
1oez_Y_ZN_1154_ZN_3593
1oez_Z_ZN_1154_ZN_3599
1ozu_A_ZN_355_ZN_2001
1p1v_A_ZN_202_ZN_3190
1p1v_B_ZN_204_ZN_3197
1p1v_C_ZN_206_ZN_3204
1ptz_A_ZN_201_ZN_2224
1ptz_B_ZN_201_ZN_2231
1pu0_A_ZN_201_ZN_11152
1pu0_B_ZN_201_ZN_11154
1pu0_C_ZN_201_ZN_11161
1pu0_D_ZN_201_ZN_11163
1pu0_E_ZN_201_ZN_11165
1pu0_F_ZN_201_ZN_11167
1pu0_G_ZN_201_ZN_11169
1pu0_H_ZN_201_ZN_11171
1pu0_I_ZN_201_ZN_11173
1pu0_J_ZN_201_ZN_11175
1q0e_A_ZN_153_ZN_2231
1q0e_B_ZN_153_ZN_2234
1s4i_A_ZN_501_ZN_4528
1s4i_B_ZN_401_ZN_4525
1s4i_C_ZN_601_ZN_4531
1s4i_D_ZN_701_ZN_4533
1sda_B_ZN_808_ZN_4402
1sda_G_ZN_811_ZN_4404
1sda_O_ZN_802_ZN_4398
1sda_Y_ZN_805_ZN_4400
1sdy_A_ZN_153_ZN_4430
1sdy_B_ZN_153_ZN_4432
1sdy_C_ZN_153_ZN_4434
1sdy_D_ZN_153_ZN_4436
1sos_A_ZN_155_ZN_11132
1sos_B_ZN_155_ZN_11141
1sos_C_ZN_155_ZN_11145
1sos_D_ZN_155_ZN_11149
1sos_E_ZN_155_ZN_11158
1sos_F_ZN_155_ZN_11134
1sos_G_ZN_155_ZN_11143
1sos_H_ZN_155_ZN_11147
1sos_I_ZN_155_ZN_11151
1sos_J_ZN_155_ZN_11160
1spd_A_ZN_155_ZN_2230
1spd_B_ZN_155_ZN_2232
1srd_A_ZN_156_ZN_4418

1srd_B_ZN_156_ZN_4420
1srd_C_ZN_156_ZN_4422
1srd_D_ZN_156_ZN_4424
1sxa_A_ZN_153_ZN_2190
1sxa_B_ZN_153_ZN_2192
1sxb_A_ZN_153_ZN_2190
1sxb_B_ZN_153_ZN_2192
1sxc_A_ZN_153_ZN_2190
1sxc_B_ZN_153_ZN_2192
1sxn_A_ZN_153_ZN_2138
1sxn_B_ZN_153_ZN_2141
1sxs_A_ZN_153_ZN_2153
1sxs_B_ZN_153_ZN_2159
1sxz_A_ZN_153_ZN_2157
1sxz_B_ZN_153_ZN_2163
1to4_A_ZN_802_ZN_4505
1to4_B_ZN_812_ZN_4508
1to4_C_ZN_822_ZN_4511
1to4_D_ZN_832_ZN_4514
1to5_A_ZN_711_ZN_4483
1to5_B_ZN_721_ZN_4485
1to5_C_ZN_731_ZN_4491
1to5_D_ZN_741_ZN_4493
1uxl_A_ZN_155_ZN_11336
1uxl_B_ZN_155_ZN_11343
1uxl_C_ZN_155_ZN_11350
1uxl_D_ZN_155_ZN_11357
1uxl_E_ZN_155_ZN_11364
1uxl_F_ZN_155_ZN_11371
1uxl_G_ZN_155_ZN_11378
1uxl_H_ZN_155_ZN_11380
1uxl_I_ZN_155_ZN_11387
1uxl_J_ZN_155_ZN_11394
1uxm_A_ZN_155_ZN_13358
1uxm_B_ZN_155_ZN_13360
1uxm_C_ZN_155_ZN_13362
1uxm_D_ZN_155_ZN_13364
1uxm_E_ZN_155_ZN_13366
1uxm_F_ZN_155_ZN_13368
1uxm_G_ZN_155_ZN_13370
1uxm_H_ZN_155_ZN_13372
1uxm_I_ZN_155_ZN_13374
1uxm_J_ZN_155_ZN_13376
1uxm_K_ZN_155_ZN_13378
1uxm_L_ZN_155_ZN_13380
1xso_A_ZN_152_ZN_2191
1xso_B_ZN_153_ZN_2193
1xtl_A_ZN_1174_ZN_4554
1xtl_B_ZN_1172_ZN_4550
1xtl_C_ZN_1176_ZN_4558
1xtl_D_ZN_1178_ZN_4561
1xtm_A_ZN_504_ZN_2283
1xtm_B_ZN_501_ZN_2278
1yai_A_ZN_153_ZN_3319
1yai_B_ZN_153_ZN_3321
1yai_C_ZN_153_ZN_3323
1yaz_A_ZN_155_ZN_1109
1yso_A_ZN_155_ZN_1109

1z9n_A_ZN_201_ZN_4684
1z9n_B_ZN_201_ZN_4729
1z9n_C_ZN_201_ZN_4731
1z9n_D_ZN_201_ZN_4776
1z9p_A_ZN_201_ZN_2351
1z9p_B_ZN_201_ZN_2353
2aeo_A_ZN_153_ZN_2188
2aeo_B_ZN_153_ZN_2193
2af2_A_ZN_154_ZN_4376
2af2_B_ZN_154_ZN_4377
2aps_A_ZN_400_ZN_2325
2aps_B_ZN_600_ZN_2328
2aqm_A_ZN_201_ZN_1166
2aqn_A_ZN_201_ZN_3487
2aqn_B_ZN_201_ZN_3490
2aqn_C_ZN_201_ZN_3497
2aqp_A_ZN_201_ZN_2337
2aqp_B_ZN_201_ZN_2364
2aqq_A_ZN_201_ZN_3510
2aqq_B_ZN_201_ZN_3512
2aqq_C_ZN_201_ZN_3520
2aqr_A_ZN_201_ZN_3462
2aqr_B_ZN_201_ZN_3464
2aqr_C_ZN_201_ZN_3467
2aqs_A_ZN_201_ZN_2343
2aqs_B_ZN_201_ZN_2346
2aqt_A_ZN_201_ZN_3471
2aqt_B_ZN_201_ZN_3464
2aqt_C_ZN_201_ZN_3461
2c9s_A_ZN_1156_ZN_2415
2c9s_F_ZN_1158_ZN_2456
2c9u_A_ZN_1158_ZN_2287
2c9u_F_ZN_1157_ZN_2296
2c9v_A_ZN_155_ZN_2413
2c9v_F_ZN_155_ZN_2427
2e46_A_ZN_172_ZN_1184
2e47_A_ZN_172_ZN_2342
2e47_B_ZN_172_ZN_2372
2gbt_A_ZN_155_ZN_4032
2gbt_B_ZN_155_ZN_4034
2gbv_A_ZN_155_ZN_11072
2gbv_B_ZN_155_ZN_11074
2gbv_C_ZN_155_ZN_11076
2gbv_D_ZN_155_ZN_11078
2gbv_E_ZN_155_ZN_11080
2gbv_F_ZN_155_ZN_11082
2gbv_G_ZN_155_ZN_11084
2gbv_H_ZN_155_ZN_11086
2gbv_I_ZN_155_ZN_11088
2gbv_J_ZN_155_ZN_11090
2jcw_A_ZN_155_ZN_1109
2jlp_A_ZN_226_ZN_5086
2jlp_B_ZN_226_ZN_5089
2jlp_C_ZN_226_ZN_5098
2jlp_D_ZN_226_ZN_5107
2k4w_A_ZN_156_ZN_2193
2nnx_A_ZN_155_ZN_4451
2nnx_B_ZN_155_ZN_4453

2nnx_C_ZN_155_ZN_4455
2nnx_D_ZN_155_ZN_4457
2q2l_A_ZN_1001_ZN_2187
2q2l_B_ZN_1002_ZN_2194
2sod_B_ZN_153_ZN_4390
2sod_G_ZN_153_ZN_4392
2sod_O_ZN_153_ZN_4386
2sod_Y_ZN_153_ZN_4388
2v0a_A_ZN_1155_ZN_2376
2v0a_F_ZN_1157_ZN_2393
2vr6_A_ZN_1156_ZN_2382
2vr6_F_ZN_1156_ZN_2393
2vr7_A_ZN_1157_ZN_2253
2vr7_F_ZN_1157_ZN_2269
2vr8_A_ZN_1158_ZN_2312
2vr8_F_ZN_1158_ZN_2327
2wko_A_ZN_155_ZN_2231
2wko_F_ZN_155_ZN_2236
2wwn_A_ZN_1163_ZN_2215
2wwn_B_ZN_1163_ZN_2232
2wwo_A_ZN_1162_ZN_2246
2wwo_B_ZN_1162_ZN_2250
2wyt_A_ZN_155_ZN_4727
2wyt_F_ZN_155_ZN_4779
2wyz_A_ZN_155_ZN_2291
2wyz_F_ZN_155_ZN_2320
2wz0_A_ZN_155_ZN_2272
2wz0_F_ZN_155_ZN_2294
2wz5_A_ZN_155_ZN_2276
2wz5_F_ZN_155_ZN_2303
2wz6_A_ZN_155_ZN_2289
2wz6_F_ZN_155_ZN_2319
2xjk_A_ZN_155_ZN_1135
2xjl_A_ZN_155_ZN_1156
2z7u_A_ZN_300_ZN_2187
2z7u_B_ZN_300_ZN_2189
2z7w_A_ZN_300_ZN_2187
2z7w_B_ZN_300_ZN_2189
2z7y_A_ZN_300_ZN_2187
2z7y_B_ZN_300_ZN_2189
2z7z_A_ZN_300_ZN_2187
2z7z_B_ZN_300_ZN_2189
2zkw_A_ZN_401_ZN_2155
2zkw_B_ZN_402_ZN_2157
2zkx_A_ZN_401_ZN_4183
2zkx_B_ZN_402_ZN_4185
2zkx_C_ZN_403_ZN_4187
2zkx_D_ZN_404_ZN_4189
2zky_A_ZN_601_ZN_11111
2zky_B_ZN_602_ZN_11112
2zky_C_ZN_603_ZN_11113
2zky_D_ZN_604_ZN_11114
2zky_E_ZN_605_ZN_11115
2zky_F_ZN_606_ZN_11116
2zky_G_ZN_607_ZN_11117
2zky_H_ZN_608_ZN_11118
2zky_I_ZN_609_ZN_11119
2zky_J_ZN_610_ZN_11120

2zow_A_ZN_300_ZN_2187
2zow_B_ZN_300_ZN_2189
3ce1_A_ZN_202_ZN_1081
3cqp_A_ZN_155_ZN_4283
3cqp_B_ZN_155_ZN_4299
3cqp_D_ZN_155_ZN_4302
3cqq_A_ZN_154_ZN_1916
3f7k_A_ZN_203_ZN_1142
3f7l_A_ZN_203_ZN_1141
3gqf_A_ZN_155_ZN_6667
3gqf_B_ZN_155_ZN_6669
3gqf_C_ZN_155_ZN_6671
3gqf_D_ZN_155_ZN_6673
3gqf_E_ZN_155_ZN_6675
3gqf_F_ZN_155_ZN_6676
3gtt_A_ZN_155_ZN_6661
3gtt_B_ZN_155_ZN_6662
3gtt_C_ZN_155_ZN_6663
3gtt_D_ZN_155_ZN_6664
3gtt_E_ZN_155_ZN_6665
3gtt_F_ZN_155_ZN_6666
3gtv_A_ZN_155_ZN_13410
3gtv_B_ZN_155_ZN_13411
3gtv_C_ZN_155_ZN_13412
3gtv_D_ZN_155_ZN_13413
3gtv_E_ZN_155_ZN_13414
3gtv_F_ZN_155_ZN_13415
3gtv_G_ZN_155_ZN_13416
3gtv_H_ZN_155_ZN_13417
3gtv_I_ZN_155_ZN_13418
3gtv_J_ZN_155_ZN_13419
3gtv_K_ZN_155_ZN_13420
3gtv_L_ZN_155_ZN_13421
3gzo_A_ZN_155_ZN_11152
3gzo_B_ZN_155_ZN_11154
3gzo_C_ZN_155_ZN_11162
3gzo_D_ZN_155_ZN_11164
3gzo_E_ZN_155_ZN_11166
3gzo_F_ZN_155_ZN_11168
3gzo_G_ZN_155_ZN_11170
3gzo_H_ZN_155_ZN_11172
3gzo_I_ZN_155_ZN_11181
3gzo_J_ZN_155_ZN_11183
3hw7_A_ZN_153_ZN_2188
3hw7_B_ZN_154_ZN_2191
3kbf_A_ZN_159_ZN_1147
3kh3_A_ZN_156_ZN_13326
3kh3_B_ZN_156_ZN_13328
3kh3_C_ZN_156_ZN_13330
3kh3_D_ZN_156_ZN_13332
3kh3_E_ZN_156_ZN_13334
3kh3_F_ZN_156_ZN_13336
3kh3_G_ZN_156_ZN_13338
3kh3_H_ZN_156_ZN_13340
3kh3_I_ZN_156_ZN_13342
3kh3_J_ZN_156_ZN_13344
3kh3_K_ZN_156_ZN_13346
3kh3_L_ZN_156_ZN_13348

3kh4_A_ZN_155_ZN_6668
3kh4_B_ZN_155_ZN_6670
3kh4_C_ZN_155_ZN_6672
3kh4_D_ZN_155_ZN_6674
3kh4_E_ZN_155_ZN_6676
3kh4_F_ZN_155_ZN_6678
3km2_A_ZN_155_ZN_26161
3km2_B_ZN_155_ZN_26162
3km2_C_ZN_155_ZN_26163
3km2_D_ZN_155_ZN_26164
3km2_E_ZN_155_ZN_26165
3km2_F_ZN_155_ZN_26166
3km2_G_ZN_155_ZN_26167
3km2_H_ZN_155_ZN_26168
3km2_I_ZN_155_ZN_26169
3km2_J_ZN_155_ZN_26170
3km2_K_ZN_155_ZN_26171
3km2_L_ZN_155_ZN_26172
3km2_M_ZN_155_ZN_26173
3km2_N_ZN_155_ZN_26174
3km2_O_ZN_155_ZN_26175
3km2_P_ZN_155_ZN_26176
3km2_Q_ZN_155_ZN_26177
3km2_R_ZN_155_ZN_26178
3km2_S_ZN_155_ZN_26179
3km2_T_ZN_155_ZN_26180
3km2_U_ZN_155_ZN_26181
3km2_V_ZN_155_ZN_26182
3km2_W_ZN_155_ZN_26183
3km2_X_ZN_155_ZN_26184
3l9e_A_ZN_155_ZN_4453
3l9e_B_ZN_155_ZN_4454
3l9e_C_ZN_155_ZN_4455
3l9e_D_ZN_155_ZN_4456
3l9y_A_ZN_155_ZN_2213
3l9y_B_ZN_155_ZN_2215
3ltv_A_ZN_1001_ZN_6598
3ltv_B_ZN_1001_ZN_6599
3ltv_C_ZN_1001_ZN_6600
3ltv_D_ZN_1001_ZN_6601
3ltv_E_ZN_1001_ZN_6602
3ltv_F_ZN_1001_ZN_6603
3sod_B_ZN_153_ZN_4388
3sod_G_ZN_153_ZN_4386
3sod_O_ZN_153_ZN_4382
3sod_Y_ZN_153_ZN_4384

------- SF_348 --------
1hty_A_ZN_1102_ZN_8197
1hww_A_ZN_1102_ZN_8197
1hxk_A_ZN_1102_ZN_8197
1o7d_A_ZN_3_ZN_7152
1ps3_A_ZN_1601_ZN_8198
1qwn_A_ZN_2005_ZN_8540
1qwu_A_ZN_2004_ZN_8197
1qx1_A_ZN_2004_ZN_8449
1r33_A_ZN_1163_ZN_8197
1r34_A_ZN_1101_ZN_8197

1tqs_A_ZN_1401_ZN_8263
1tqt_A_ZN_1301_ZN_8260
1tqu_A_ZN_1400_ZN_8361
1tqv_A_ZN_1300_ZN_8253
1tqw_A_ZN_1400_ZN_8306
2alw_A_ZN_5001_ZN_8197
2f18_A_ZN_1805_ZN_8346
2f1a_A_ZN_1805_ZN_8328
2f1b_A_ZN_1804_ZN_8307
2f7o_A_ZN_5001_ZN_8234
2f7p_A_ZN_5001_ZN_8393
2f7q_A_ZN_5001_ZN_8272
2f7r_A_ZN_5001_ZN_8258
2fyv_A_ZN_2003_ZN_8237
2ow6_A_ZN_3001_ZN_8403
2ow7_A_ZN_5001_ZN_8322
2wyh_A_ZN_902_ZN_14436
2wyh_B_ZN_902_ZN_14479
2wyi_A_ZN_1906_ZN_14366
2wyi_B_ZN_1908_ZN_14435
3blb_A_ZN_1047_ZN_8289
3bub_A_ZN_1047_ZN_8586
3bud_A_ZN_1048_ZN_8431
3bui_A_ZN_1046_ZN_8545
3bup_A_ZN_1048_ZN_8308
3buq_A_ZN_1048_ZN_8311
3bvt_A_ZN_1048_ZN_8474
3bvu_A_ZN_1048_ZN_8443
3bvv_A_ZN_1047_ZN_8588
3bvw_A_ZN_1046_ZN_8482
3bvx_A_ZN_1046_ZN_8587
3cv5_A_ZN_1047_ZN_8390
3czn_A_ZN_1102_ZN_8450
3czs_A_ZN_1102_ZN_8463
3d4y_A_ZN_1047_ZN_8303
3d4z_A_ZN_1046_ZN_8266
3d50_A_ZN_1047_ZN_8307
3d51_A_ZN_1046_ZN_8261
3d52_A_ZN_1046_ZN_8211
3ddf_A_ZN_3001_ZN_8484
3ddg_A_ZN_3001_ZN_8316
3dx0_A_ZN_1047_ZN_8378
3dx1_A_ZN_1048_ZN_8379
3dx2_A_ZN_1046_ZN_8350
3dx3_A_ZN_1047_ZN_8427
3dx4_A_ZN_1047_ZN_8363
3ejp_A_ZN_1047_ZN_8431
3ejq_A_ZN_1047_ZN_8340
3ejr_A_ZN_1047_ZN_8410
3ejs_A_ZN_1047_ZN_8362
3ejt_A_ZN_1047_ZN_8381
3eju_A_ZN_1047_ZN_8372

------- SF_349 --------
1nzj_A_ZN_700_ZN_2165
2zlz_A_ZN_300_ZN_2365
3aii_A_ZN_1001_ZN_3759

------- SF_350 --------
1ton_A_ZN_247_ZN_1736
2bdh_A_ZN_401_ZN_6685
2psy_A_ZN_2000_ZN_1824

------- SF_351 --------
1vq2_A_ZN_702_ZN_1323
2hvv_A_ZN_504_ZN_2264
2hvv_B_ZN_502_ZN_2285
2hvw_A_ZN_1001_ZN_3524
2hvw_B_ZN_1003_ZN_3581
2hvw_C_ZN_1005_ZN_3638

------- SF_352 --------
2kqb_A_ZN_1001_ZN_793
2kqc_A_ZN_1001_ZN_738
2kqd_A_ZN_1001_ZN_793
2kqe_A_ZN_1001_ZN_738
2kuo_A_ZN_449_ZN_1392
2kuo_A_ZN_450_ZN_1393
2xoc_A_ZN_995_ZN_3438
2xoc_B_ZN_995_ZN_3480
2xoy_A_ZN_995_ZN_3391
2xoy_B_ZN_995_ZN_3396
2xoz_A_ZN_995_ZN_3426
2xoz_B_ZN_995_ZN_3431
2xp0_A_ZN_995_ZN_3413
2xp0_B_ZN_995_ZN_3418

------- SF_353 --------
3nih_A_ZN_1_ZN_645,3nih_A_ZN_2_ZN_646
3nii_A_ZN_1_ZN_643,3nii_A_ZN_2_ZN_644
3nij_A_ZN_1_ZN_644,3nij_A_ZN_2_ZN_645
3nik_A_ZN_1_ZN_2579,3nik_A_ZN_2_ZN_2580
3nik_B_ZN_1_ZN_2582,3nik_B_ZN_2_ZN_2583
3nik_D_ZN_1_ZN_2585,3nik_D_ZN_2_ZN_2586
3nik_F_ZN_1_ZN_2588,3nik_F_ZN_2_ZN_2589
3nil_A_ZN_1_ZN_2552,3nil_A_ZN_2_ZN_2553
3nil_B_ZN_1_ZN_2555,3nil_B_ZN_2_ZN_2556
3nil_D_ZN_1_ZN_2558,3nil_D_ZN_2_ZN_2559
3nil_F_ZN_1_ZN_2561,3nil_F_ZN_2_ZN_2562
3nim_A_ZN_1_ZN_2577,3nim_A_ZN_2_ZN_2578
3nim_B_ZN_1_ZN_2580,3nim_B_ZN_2_ZN_2581
3nim_D_ZN_1_ZN_2583,3nim_D_ZN_2_ZN_2584
3nim_F_ZN_1_ZN_2586,3nim_F_ZN_2_ZN_2587
3nin_A_ZN_1_ZN_1316,3nin_A_ZN_2_ZN_1317
3nin_B_ZN_1_ZN_1319,3nin_B_ZN_2_ZN_1320
3nis_A_ZN_1_ZN_2547,3nis_A_ZN_2_ZN_2548
3nis_B_ZN_1_ZN_2554,3nis_B_ZN_2_ZN_2555
3nis_D_ZN_1_ZN_2561,3nis_D_ZN_2_ZN_2562
3nis_F_ZN_1_ZN_2564,3nis_F_ZN_2_ZN_2565
3nit_A_ZN_1_ZN_692,3nit_A_ZN_2_ZN_693
3ny1_A_ZN_4_ZN_1094,3ny1_A_ZN_5_ZN_1095
3ny1_B_ZN_1_ZN_1097,3ny1_B_ZN_2_ZN_1098
3ny2_A_ZN_1_ZN_4306,3ny2_A_ZN_2_ZN_4307
3ny2_B_ZN_4_ZN_4309,3ny2_B_ZN_5_ZN_4310
3ny2_C_ZN_7_ZN_4312,3ny2_C_ZN_8_ZN_4313
3ny2_D_ZN_10_ZN_4315,3ny2_D_ZN_11_ZN_4316

3ny2_E_ZN_13_ZN_4318,3ny2_E_ZN_14_ZN_4319
3ny2_F_ZN_16_ZN_4321,3ny2_F_ZN_17_ZN_4322
3ny2_G_ZN_19_ZN_4324,3ny2_G_ZN_20_ZN_4325
3ny2_H_ZN_22_ZN_4327,3ny2_H_ZN_23_ZN_4328
3ny3_A_ZN_1_ZN_579,3ny3_A_ZN_2_ZN_580

------- SF_354 --------
1f5f_A_ZN_251_ZN_1369
1lhn_A_ZN_501_ZN_1412
1lhv_A_ZN_501_ZN_1374

------- SF_355 --------
1ozj_A_ZN_145_ZN_2686
1ozj_B_ZN_145_ZN_2687
3kmp_A_ZN_2_ZN_2645
3kmp_B_ZN_1_ZN_2652

------- SF_356 --------
1uns_A_ZN_1238_ZN_1896

------- SF_357 --------
1x6m_A_ZN_201_ZN_5940
1x6m_B_ZN_201_ZN_5947
1x6m_C_ZN_201_ZN_5972
1x6m_D_ZN_201_ZN_5990

------- SF_358 --------
1xer_A_ZN_106_ZN_772
2vkr_A_ZN_106_ZN_5418
2vkr_B_ZN_106_ZN_5434
2vkr_C_ZN_106_ZN_5450
2vkr_D_ZN_106_ZN_5466
2vkr_E_ZN_106_ZN_5482
2vkr_F_ZN_106_ZN_5498
2vkr_G_ZN_106_ZN_5514

------- SF_359 --------
2c2f_A_ZN_1211_ZN_1468
2c2u_A_ZN_1209_ZN_1512

------- SF_360 --------
2fuq_A_ZN_1_ZN_12000
2fuq_B_ZN_2_ZN_12053
2fut_A_ZN_1_ZN_11900
2fut_B_ZN_2_ZN_11936
3e7j_A_ZN_1_ZN_11879
3e7j_B_ZN_2_ZN_11948
3e80_A_ZN_1_ZN_17935
3e80_B_ZN_2_ZN_18023
3e80_C_ZN_3_ZN_18106

------- SF_361 --------
2l0z_A_ZN_487_ZN_671

------- SF_362 --------
2qsw_A_ZN_201_ZN_804

------- SF_363 --------

3kdk_A_ZN_1_ZN_3045
3kdk_B_ZN_3_ZN_3047

------- SF_364 --------
3lnn_A_ZN_360_ZN_4347

------- SF_365 --------
3mi9_C_ZN_87_ZN_5059
3mia_C_ZN_87_ZN_5044

------- SF_366 --------
3mo0_A_ZN_10_ZN_3923
3mo0_B_ZN_9_ZN_4032

------- SF_367 --------
3pt6_A_ZN_1_ZN_15516
3pt6_A_ZN_2_ZN_15517
3pt6_A_ZN_4_ZN_15519
3pt6_B_ZN_2_ZN_15521
3pt6_B_ZN_3_ZN_15522
3pt6_B_ZN_4_ZN_15523
3pt9_A_ZN_1_ZN_6500
3pta_A_ZN_1_ZN_7989
3pta_A_ZN_2_ZN_7990
3pta_A_ZN_5_ZN_7992
