# Supplementary material for: Minimal Functional Sites Allow a Classification of Zinc Sites in Proteins
Source: PLoS One. 2011 Oct 17;6(10):e26325. doi: 10.1371/journal.pone.0026325 (PMC3197139; doi:10.1371/journal.pone.0026325)
Supplement: Table S4 — Schematic picture of the structures of the representative Zn-sites included in each Zn-cluster. (PDF) [file pone.0026325.s004.pdf]

Table S4. Schematic picture of the structures of the representative Zn-sites included in each Zn-cluster, showing the Zn atoms and their protein ligands. Zn atoms in the site are identified by PDB code, PDB residue number and PDB chain identifier (these two latter in parentheses). Underlined ligands are ligands that have no counterpart in the other sites of the cluster (i.e., they are not structurally superimposable to a ligand of the other sites in the cluster). Below each picture, (i) the name of the protein containing the site, (ii) the number of Zn-sites and (in parentheses) the number of non-redundant Zn-proteins represented by the site, and (iii) the site function, are reported.

Zinc Ribbons

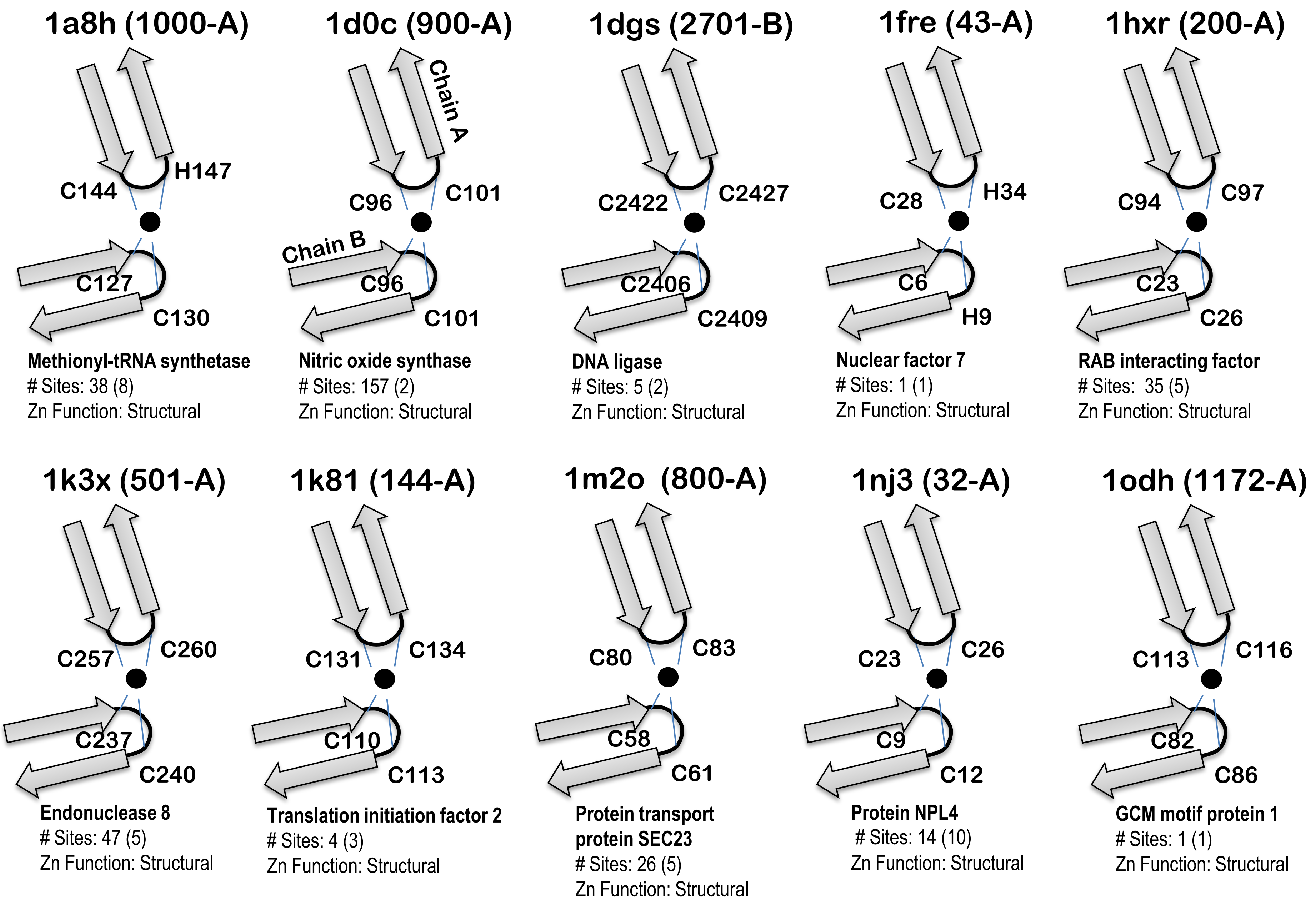

Zinc Ribbons

1p91 (1301-A)

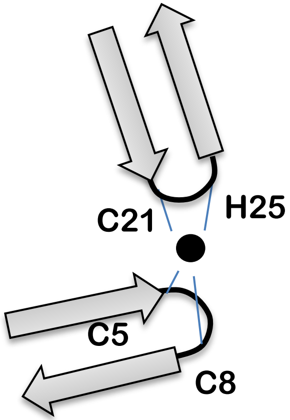

rRNA (guanine-N(1)-)-Methyltransferase
Sites: 2 (1)
Zn Function: Structural

1pfv (552-A)

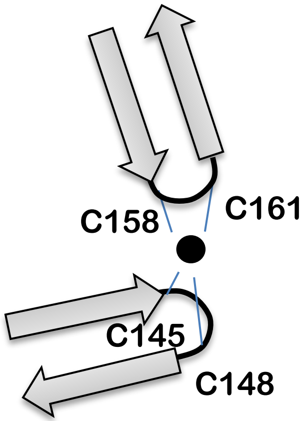

Met-tRNA synthetase
Sites: 12 (1)
Zn Function: Structural

1qf8 (216-A)

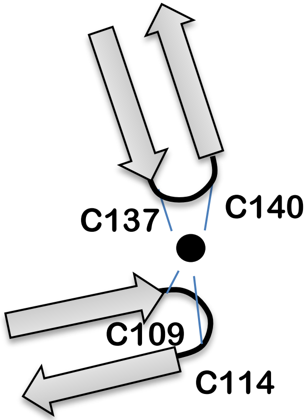

Casein kinase II subunit beta
Sites: 16 (1)
Zn Function: Structural

1t8h (1-A)

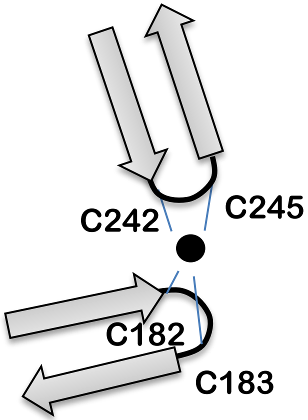

Ylmd protein
Sites: 1 (1)
Function: Unknown

1twf (3005-L)

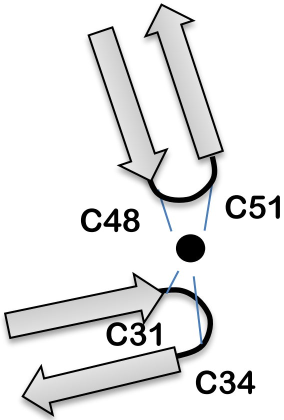

RNA polymerases I, II, and III subunit ABC4
Sites: 70 (5)
Function: Structural

1twf (3006-A)

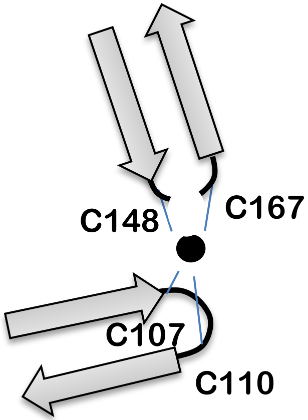

RNA polymerase II subunit 1
Sites: 120 (6)
Function: Structural

1vk6 (301-A)

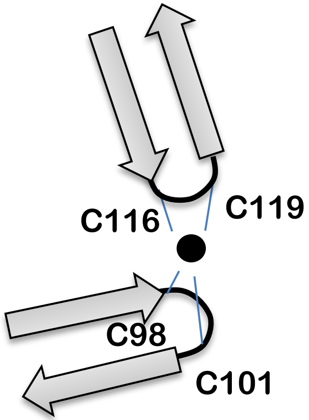

NADH pyrophosphatase
Sites: 7 (4)
Function: Structural

1wge (201-A)

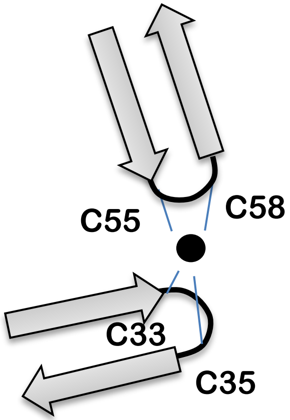

DPH3 homolog
Sites: 12 (6)
Zn Function: Structural

1x4j (401-A)

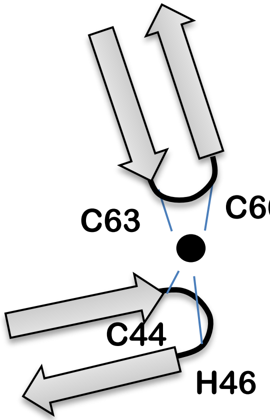

RING finger protein 38
Sites: 9 (7)
Zn Function: Structural

1yc5 (1001-A)

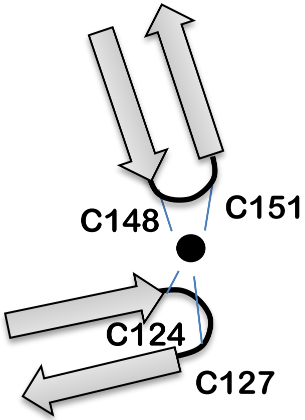

NAD-dependent deacetylase
Sites: 79 (14)
Zn Function: Structural

Zinc Ribbons

1zin (219-A)

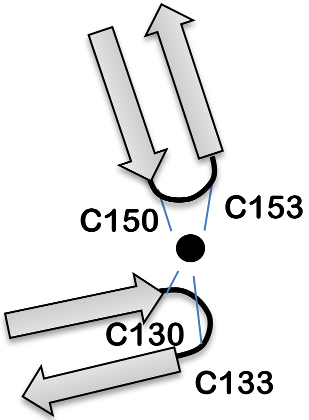

Adenylate kinase
Sites: 30 (4)
Zn Function: Structural

1zt2 (999-A)

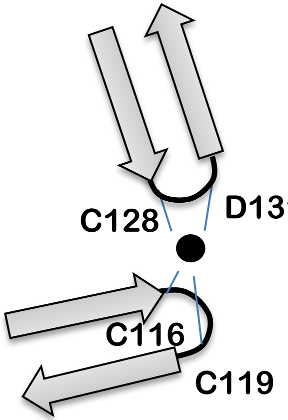

Probable DNA primase small subunit
Sites: 2 (1)
Zn Function: Structural

2a6h (7458-D)

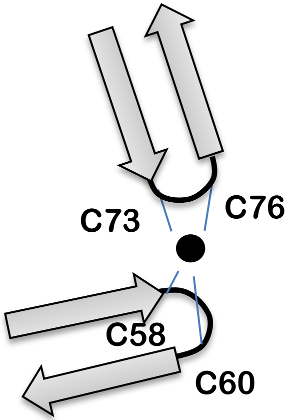

RNA polymerase subunit beta
Sites: 26 (1)
Function: Structural

2air (800-B)

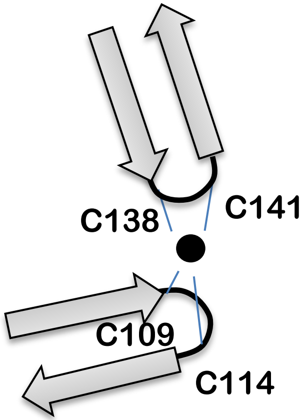

Aspartate carbamoyltransferase regulatory chain
Sites: 108 (3)
Function: Structural

2apo (501-B)

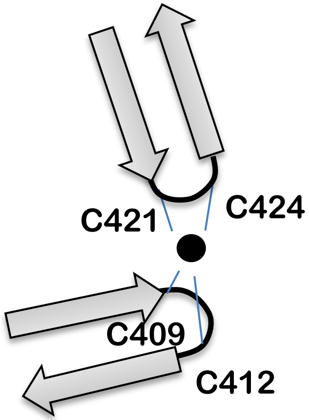

tRNA-uridine isomerase
Sites: 9 (1)
Function: Structural

2ayj (57-A)

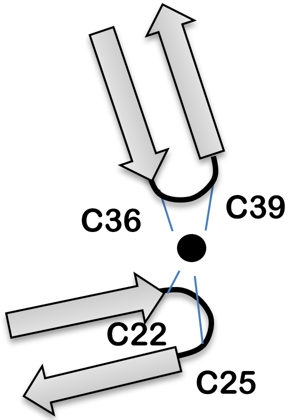

50S ribosomal protein L40e
Sites: 10 (3)
Function: Structural

2ba1 (201-A)

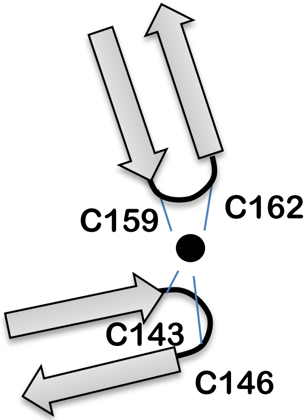

Putative uncharacterized protein
Sites: 9 (1)
Zn Function: Structural

2ckl (1115-B)

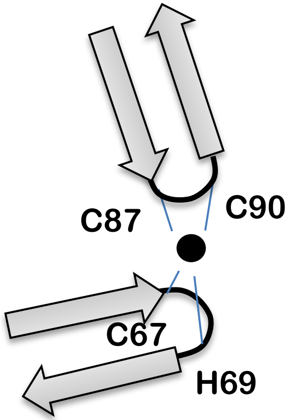

Polycomb complex protein BMI-1
Sites: 5 (4)
Zn Function: Structural

2con (201-A)

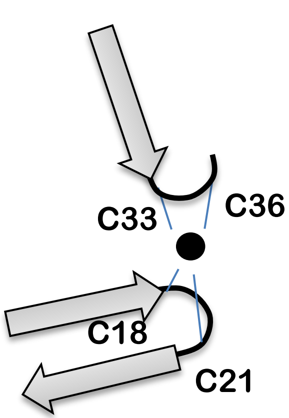

RNA-binding protein NOB1
Sites: 23 (12)
Zn Function: Structural

2d8s (401-A)

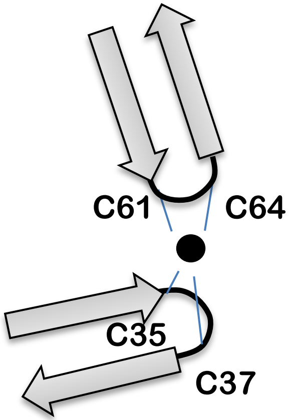

E3 ubiquitin-protein ligase MARCH8
Sites: 1 (1)
Zn Function: Structural

Zinc Ribbons

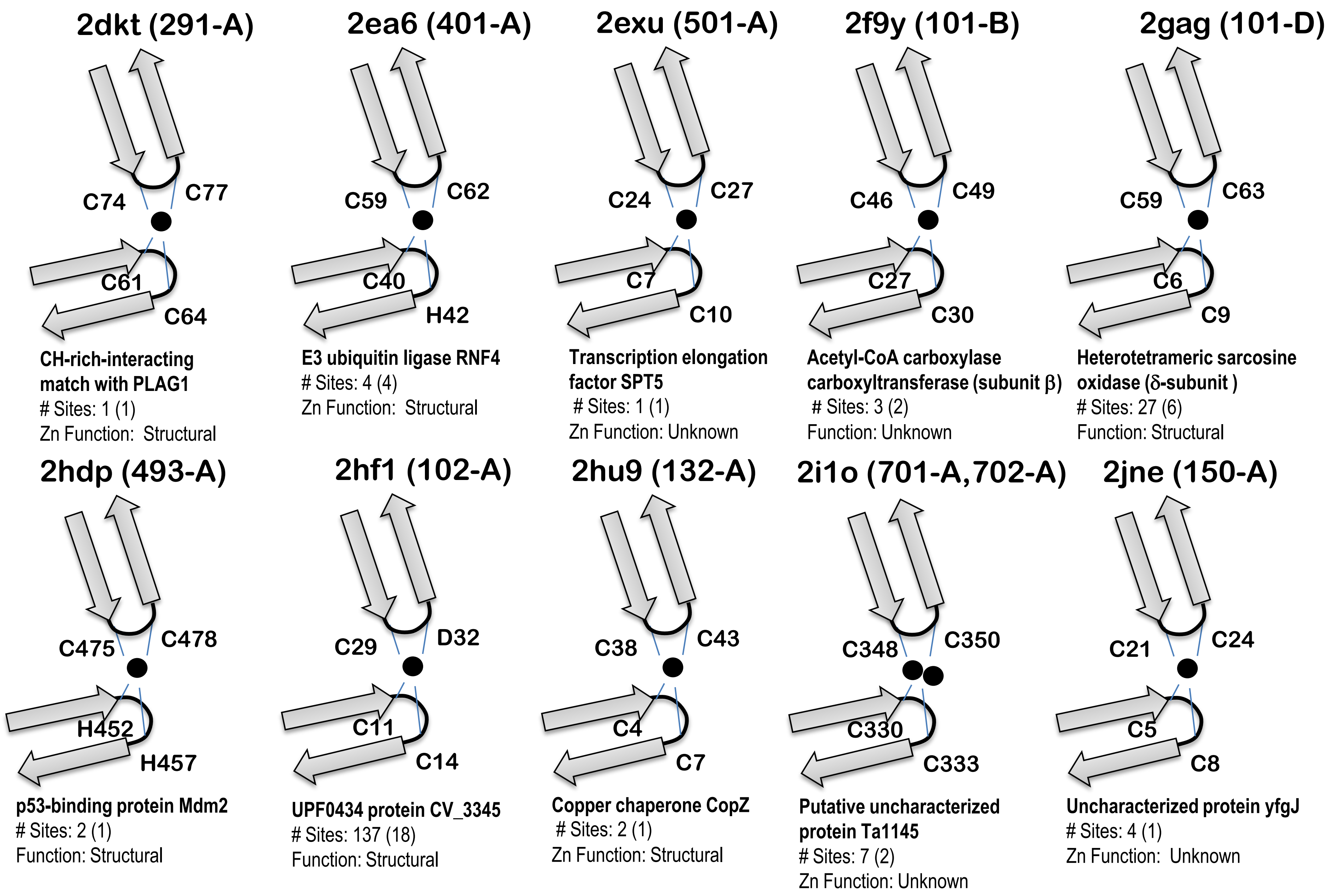

Zinc Ribbons

2jox (108-A,109-A)

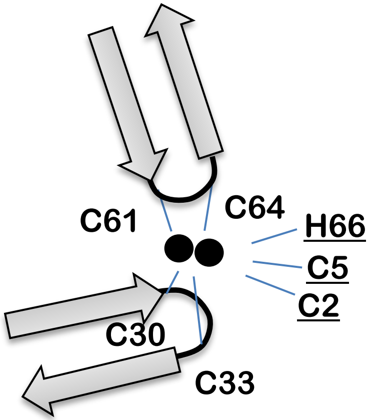

Protein Churchill
Sites: 1 (1)
Zn Function: Structural

2k5c (96-A)

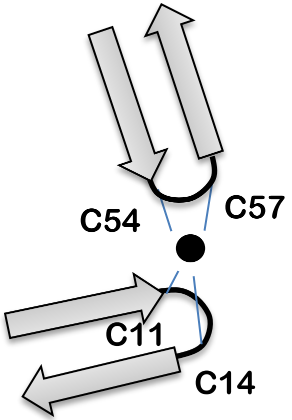

Putative uncharacterized protein
Sites: 1 (1)
Zn Function: Unknown

2kkr (500-A)

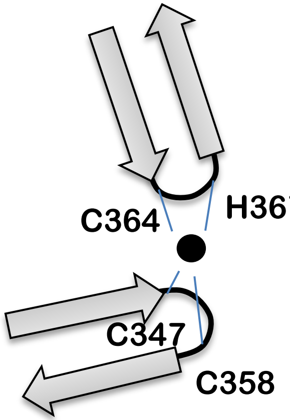

Ataxin-7
Sites: 2 (2)
Function: Structural

2kv1 (125-A)

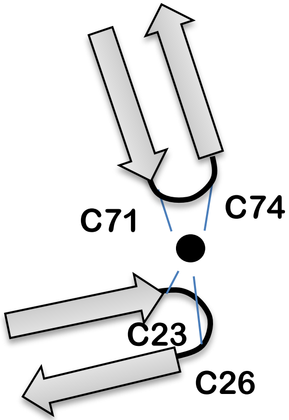

Methionine-R-sulfoxide reductase B1
Sites: 11 (5)
Function: Unknown

2r6f (1004-A)

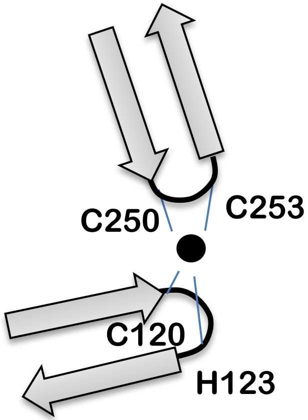

Excinuclease ABC subunit A
Sites: 2 (1)
Function: Structural

2rf5 (1-A)

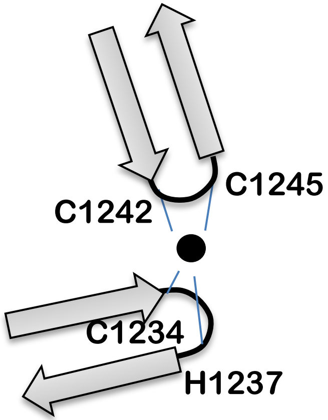

Poly [ADP-ribose] polymerase 1
Sites: 13 (1)
Function: Structural

2riq (1-A)

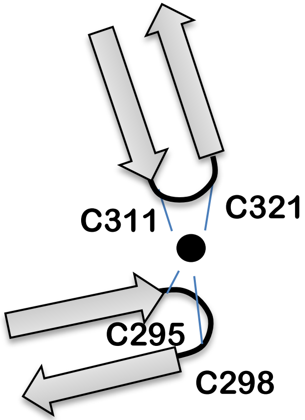

Tankyrase-1
Sites: 2 (1)
Function: Structural

2xoc (992-A, 993-A)

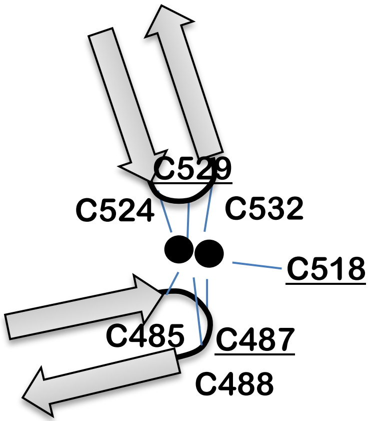

E3 ubiquitin-protein ligase CHFR
Sites: 8 (1)
Zn Function: Structural

2yu4 (201-A)

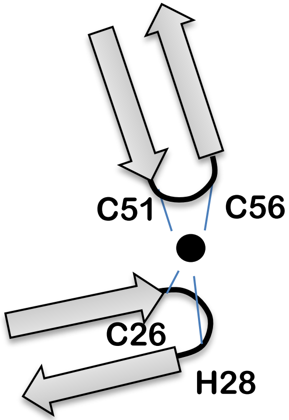

E3 SUMO-protein ligase NSE2
Sites: 6 (5)
Zn Function: Structural

2zae (121-B)

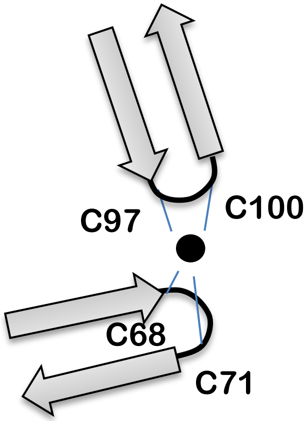

Ribonuclease P protein component 1
Sites: 5 (1)
Zn Function: Structural

Zinc Ribbons

3bvo (301-A)

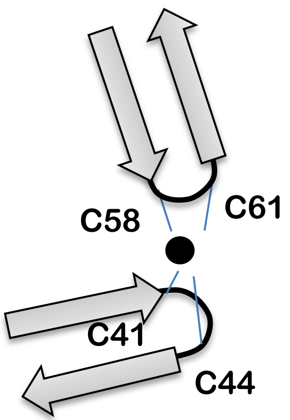

DnaJ homolog subfamily C member 20
Sites: 2 (1)
Zn Function: Unknown

3cng (508-A)

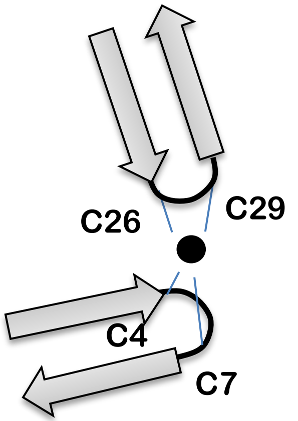

NUDIX hydrolase
Sites: 4 (1)
Zn Function: Unknown

3e9s (318-A)

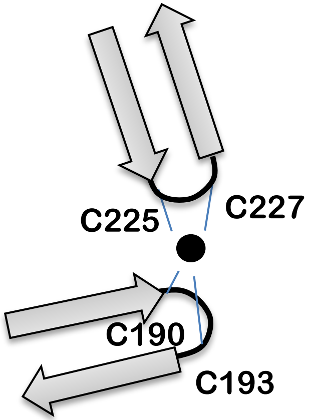

Replicase polyprotein 1ab
Sites: 3 (1)
Zn Function: Unknown

3epz (701-A)

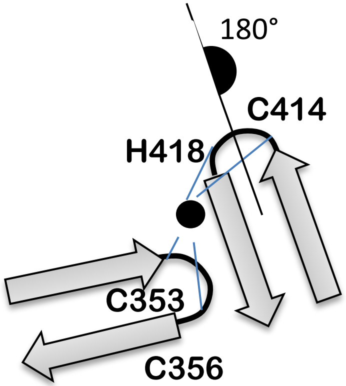

DNA (cytosine-5)-methyltransferase 1
Sites: 2 (1)
Zn Function: Unknown

3f2b (4-A)

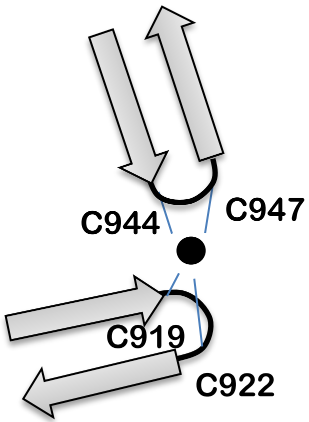

DNA-directed DNA polymerase III alpha chain
Sites: 3 (1)
Function: Structural

3fl2 (1002-A)

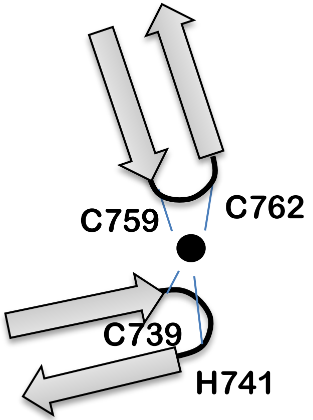

Nuclear zinc finger Protein Np95
Sites: 42 (30)
Function: Structural

3flo (1-B)

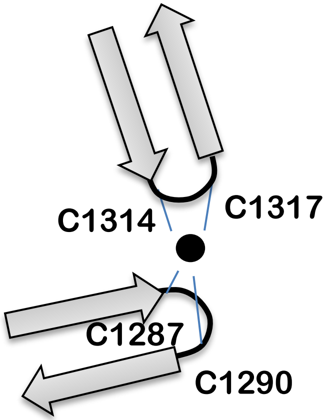

DNA polymerase I subunit A
Sites: 4 (1)
Function: Structural

3flo (2-B)

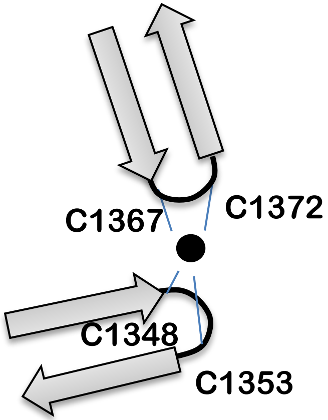

DNA polymerase I subunit A
Sites: 4 (1)
Function: Structural

3gj3 (300-B)

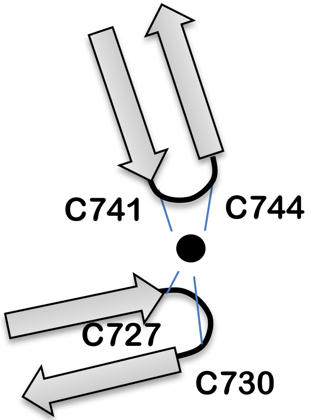

GTP-binding nuclear protein Ran
Sites: 16 (4)
Zn Function: Structural

3ir9 (501-A)

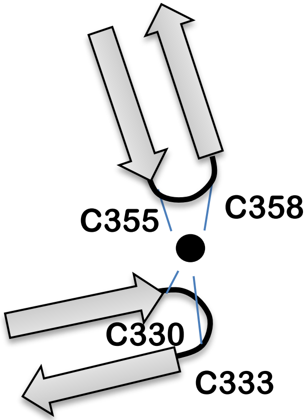

Translation termination factor aRF1
Sites: 2 (1)
Zn Function: Unknown

Zinc Ribbons

3irb (201-A)

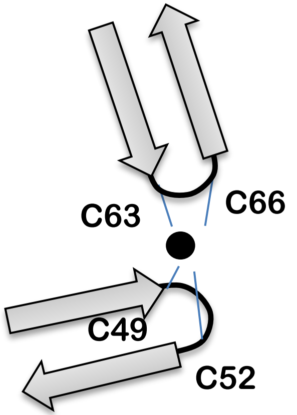

Putative uncharacterized protein
Sites: 2 (1)
Zn Function: Unknown

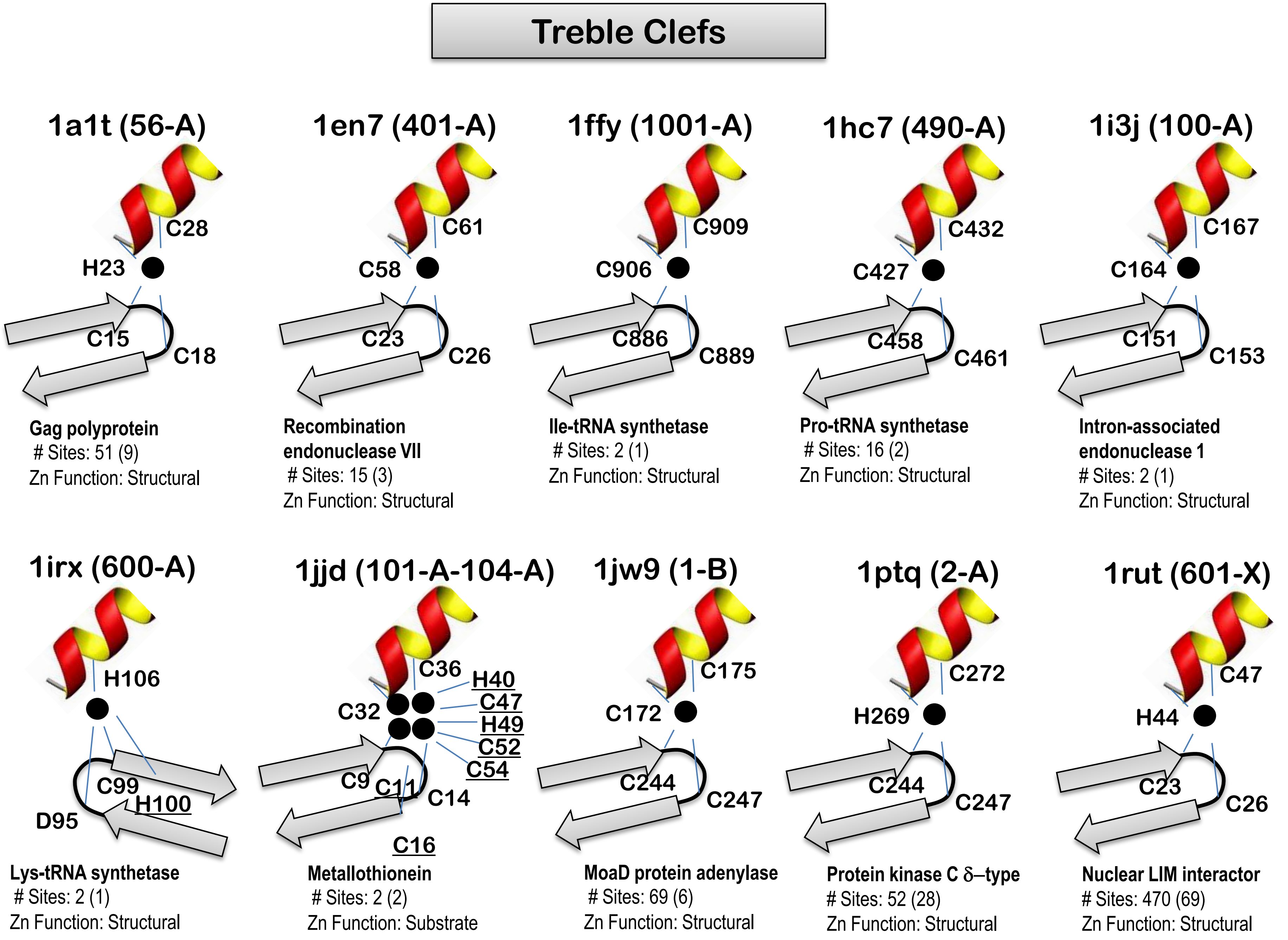

Treble Clefs
1a1t (56-A)
C28
H23
C15
C18
Gag polyprotein
Sites: 51 (9)
Zn Function: Structural
1en7 (401-A)
C61
C58
C23
C26
Recombination endonuclease VII
Sites: 15 (3)
Zn Function: Structural
1ffy (1001-A)
C909
C906
C886
C889
Ile-tRNA synthetase
Sites: 2 (1)
Zn Function: Structural
1hc7 (490-A)
C432
C427
C458
C461
Pro-tRNA synthetase
Sites: 16 (2)
Zn Function: Structural
1i3j (100-A)
C167
C164
C151
C153
Intron-associated endonuclease 1
Sites: 2 (1)
Zn Function: Structural
1irx (600-A)
H106
C99
H100
D95
Lys-tRNA synthetase
Sites: 2 (1)
Zn Function: Structural
1jjd (101-A-104-A)
C36
H40
C47
H49
C52
C54
C32
C9
C11
C14
C16
Metallothionein
Sites: 2 (2)
Zn Function: Substrate
1jw9 (1-B)
C175
C172
C244
C247
MoaD protein adenylase
Sites: 69 (6)
Zn Function: Structural
1ptq (2-A)
C272
H269
C244
C247
Protein kinase C δ–type
Sites: 52 (28)
Zn Function: Structural
1rut (601-X)
C47
H44
C23
C26
Nuclear LIM interactor
Sites: 470 (69)
Zn Function: Structural

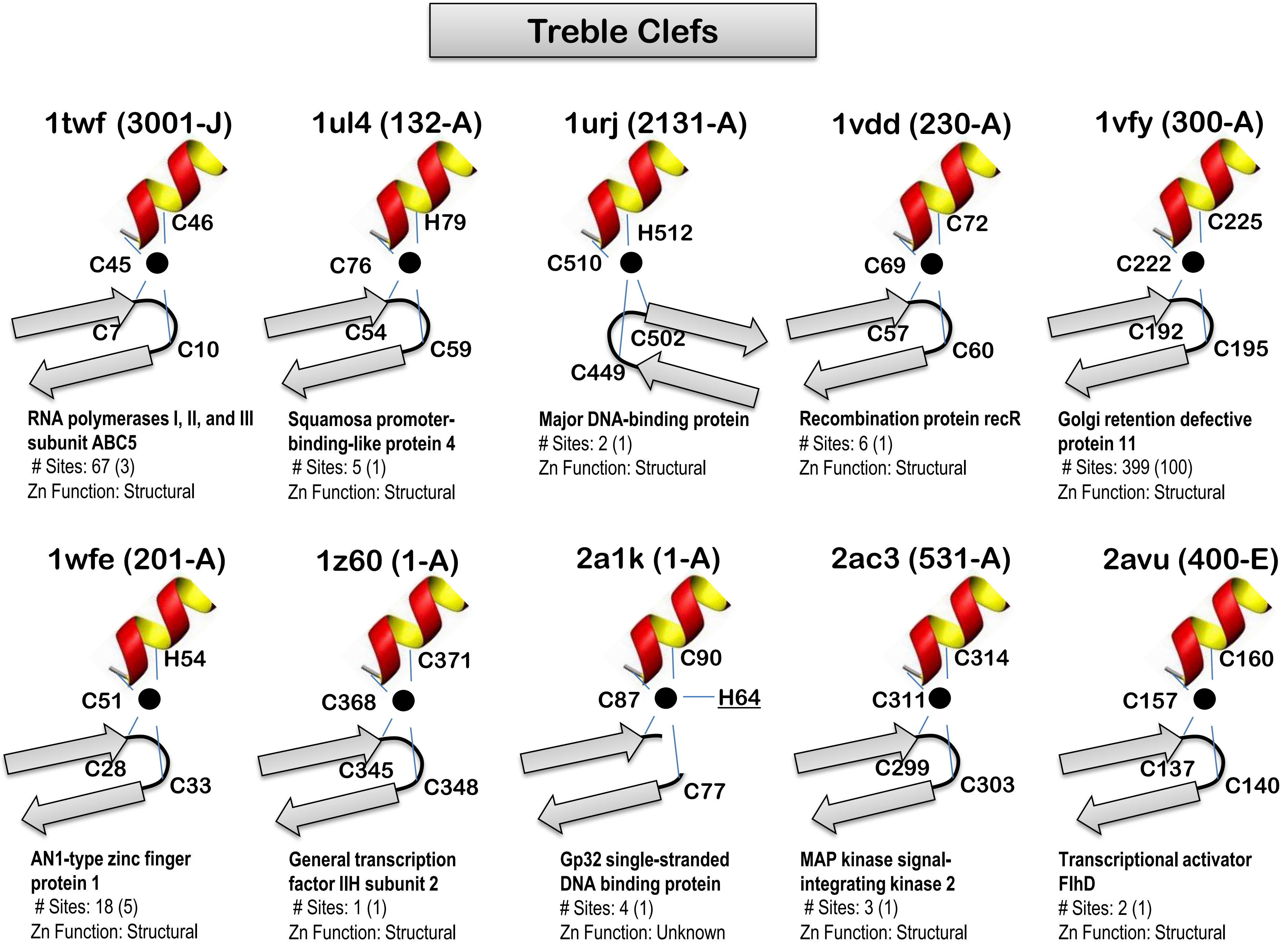
Treble Clefs
1twf (3001-J)
C46
C45
C7
C10
RNA polymerases I, II, and III subunit ABC5
Sites: 67 (3)
Zn Function: Structural
1ul4 (132-A)
H79
C76
C54
C59
Squamosa promoter-binding-like protein 4
Sites: 5 (1)
Zn Function: Structural
1urj (2131-A)
H512
C510
C502
C449
Major DNA-binding protein
Sites: 2 (1)
Zn Function: Structural
1vdd (230-A)
C72
C69
C57
C60
Recombination protein recR
Sites: 6 (1)
Zn Function: Structural
1vfy (300-A)
C225
C222
C192
C195
Golgi retention defective protein 11
Sites: 399 (100)
Zn Function: Structural
1wfe (201-A)
H54
C51
C28
C33
AN1-type zinc finger protein 1
Sites: 18 (5)
Zn Function: Structural
1z60 (1-A)
C371
C368
C345
C348
General transcription factor IIH subunit 2
Sites: 1 (1)
Zn Function: Structural
2a1k (1-A)
C90
C87
H64
C77
Gp32 single-stranded DNA binding protein
Sites: 4 (1)
Zn Function: Unknown
2ac3 (531-A)
C314
C311
C299
C303
MAP kinase signal-integrating kinase 2
Sites: 3 (1)
Zn Function: Structural
2avu (400-E)
C160
C157
C137
C140
Transcriptional activator FlhD
Sites: 2 (1)
Zn Function: Structural

Treble Clefs

2cs8 (601-A)

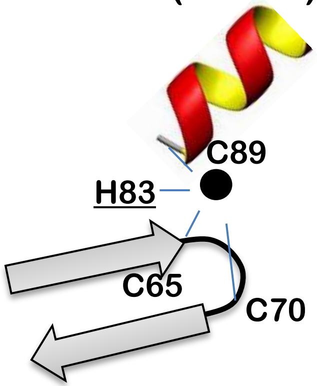

Suppression of tumorigenicity 18 protein
Sites: 4 (2)
Zn Function: Structural

2csv (200-A)

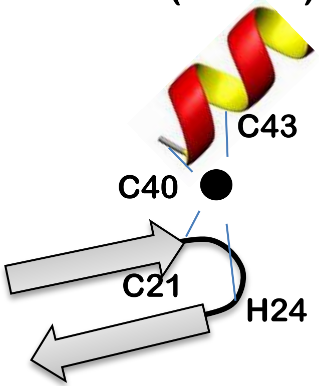

Tripartite motif-containing protein 29
Sites: 10 (5)
Zn Function: Structural

2d6f (1900-D)

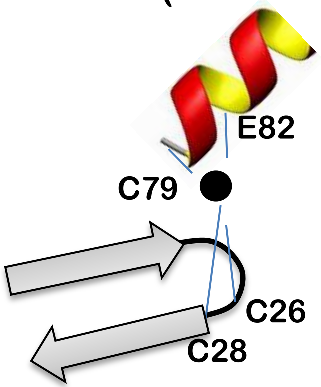

Glu-ADT subunit E
Sites: 28 (3)
Zn Function: Structural

2d8q (201-A)

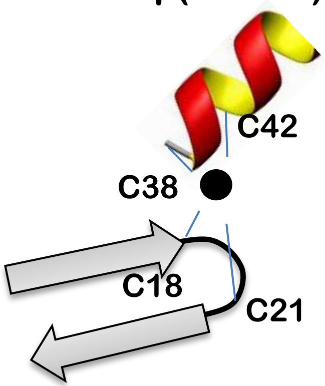

Zinc finger MYND domain-containing protein 10
Sites: 40 (13)
Zn Function: Structural

2d8r (401-A)

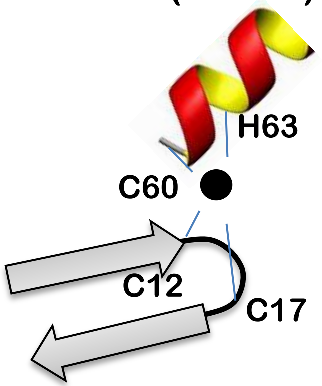

THAP domain-containing protein 2
Sites: 6 (4)
Zn Function: Structural

2dkt (191-A)

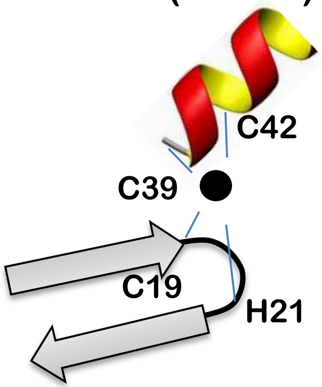

Androgen receptor N-terminal-interacting protein
Sites: 2 (1)
Zn Function: Unknown

2e61 (201-A)

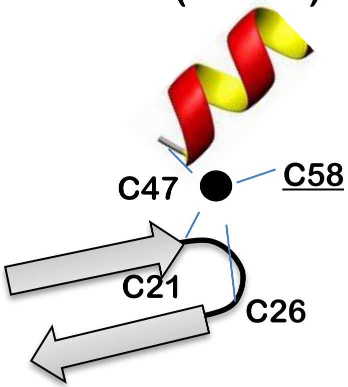

Zinc finger CW-type PWWP domain protein 1
Sites: 2 (1)
Zn Function: Structural

2ea5 (201-A)

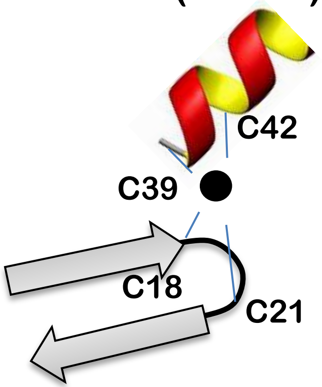

Cell growth regulatory gene 19 protein
Sites: 1 (1)
Zn Function: Structural

2fe3 (201-A)

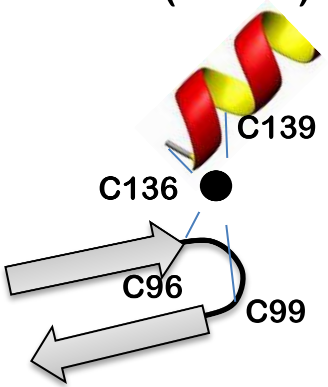

Peroxide operon regulator
Sites: 6 (1)
Zn Function: Structural

2hqh (1500-E)

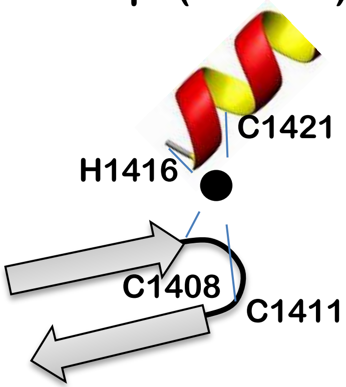

Dynactin subunit 1
Sites: 8 (1)
Zn Function: Structural

Treble Clefs

2hye (3002-B)

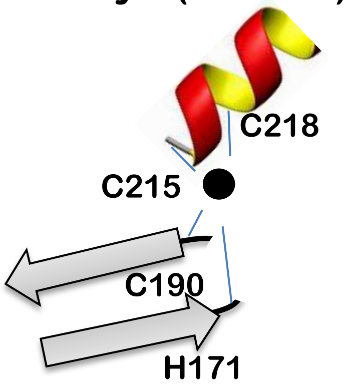

Non-structural protein V
Sites: 1 (1)
Zn Function: Unknown

2i9w (201-A)

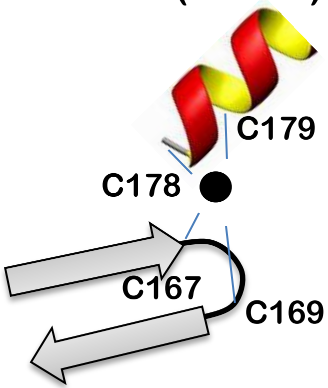

Putative uncharacterized protein
Sites: 7 (3)
Zn Function: Structural

2inp (6-A)

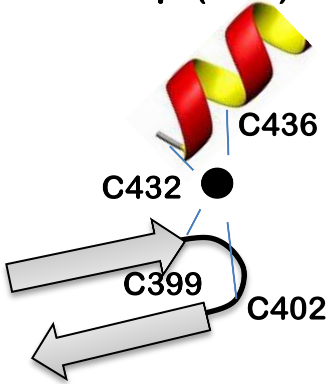

Phenol hydroxylase component phN
Sites: 2 (1)
Zn Function: Structural

2ioi (3001-A)

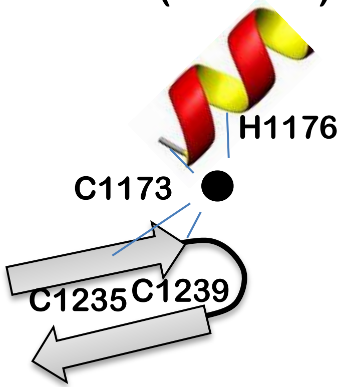

Cellular tumor antigen p53
Sites: 120 (3)
Zn Function: Structural

2j02 (639-Z)

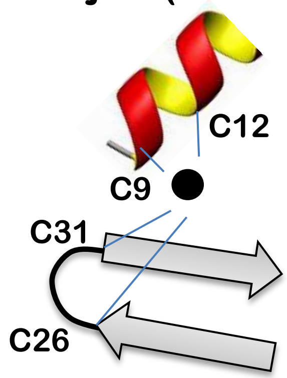

Ribosomal protein
Sites: 68 (1)
Zn Function: Structural

2jmi (201-A)

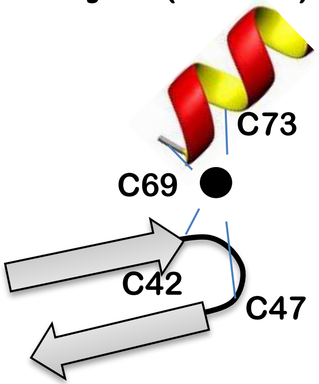

Protein YNG1
Sites: 2 (1)
Zn Function: Structural

2k0a (108-A)

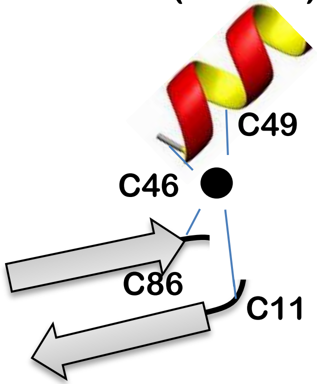

Pre-mRNA-splicing factor RDS3
Sites: 2 (1)
Zn Function: Structural

2k0a (109-A)

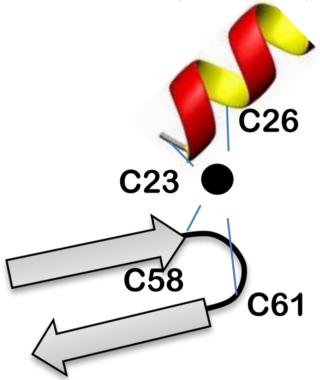

Pre-mRNA-splicing factor RDS3
Sites: 1 (1)
Zn Function: Structural

2k17 (904-A)

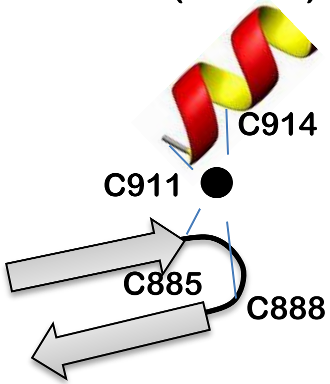

Transcription initiation factor TFIID subunit 3
Sites: 4 (1)
Zn Function: Structural

2kdp (1-A)

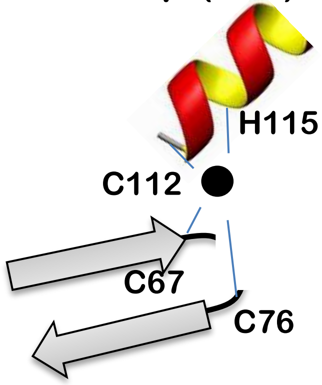

30 kDa Sin3-associated polypeptide
Sites: 1 (1)
Zn Function: Structural

Treble Clefs

2kgg (54-A)

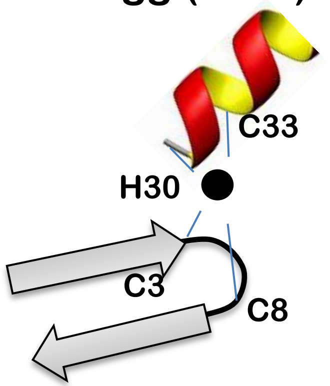

Lysine-specific demethylase 5A
Sites: 3 (1)
Zn Function: Structural

2kgo (109-A)

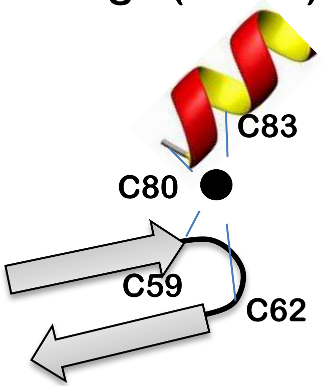

Uncharacterized protein ybil
Sites: 1 (1)
Zn Function: Structural

2o03 (202-A)

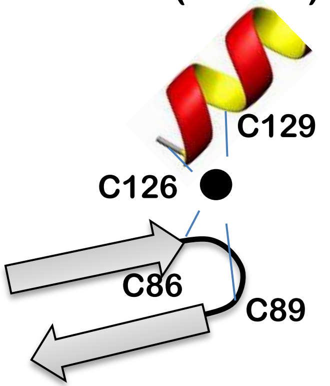

Ferric uptake regulation protein
Sites: 1 (1)
Zn Function: Structural

2ro1 (201-A)

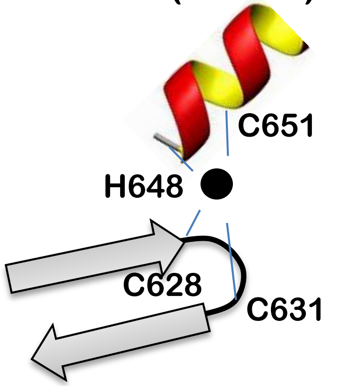

KRAB-associated protein 1
Sites: 1 (1)
Zn Function: Structural

2v89 (1488-B)

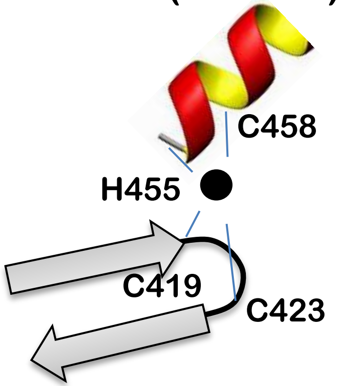

V(D)J recombination-activating protein 2
Sites: 7 (1)
Zn Function: Structural

2v89 (1489-B)

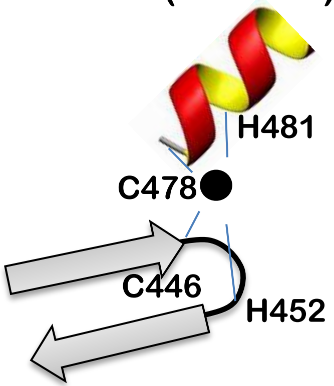

V(D)J recombination-activating protein 2
Sites: 10 (1)
Zn Function: Structural

2v9k (1533-A)

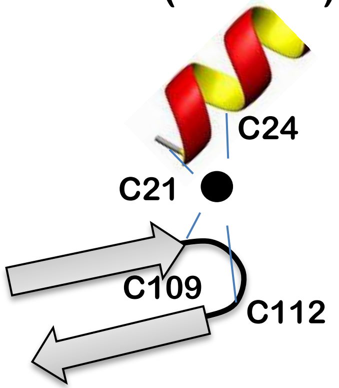

tRNA pseudouridine 55 synthase
Sites: 1 (1)
Zn Function: Structural

2wb0 (601-X)

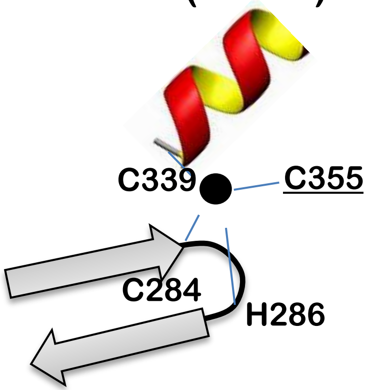

Early E2A DNA-binding protein
Sites: 14 (1)
Zn Function: Structural

2wjy (1-A)

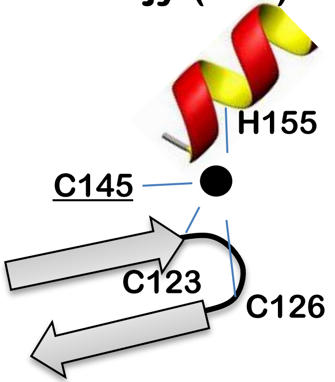

Regulator of nonsense transcripts 1
Sites: 5 (1)
Zn Function: Structural

2wjy (3-A)

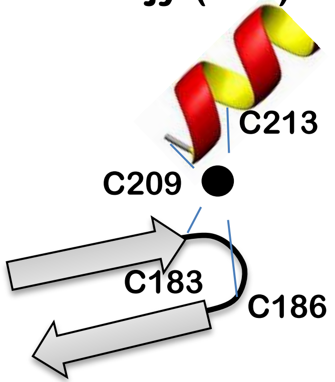

Regulator of nonsense transcripts 1
Sites: 5 (1)
Zn Function: Structural

Treble Clefs

2x5r (1126-A)

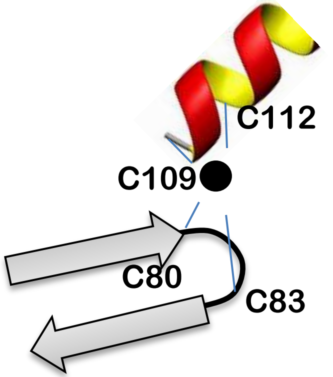

Putative uncharacterized protein
Sites: 1 (1)
Zn Function: Unknown

2x7m (1175-A)

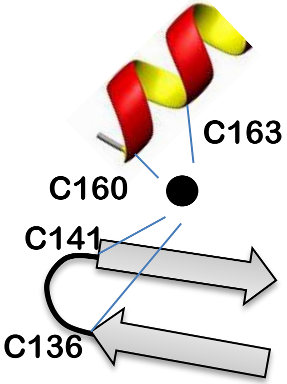

Archaemetzincin
Sites: 3 (2)
Zn Function: Structural

2zze (753-A)

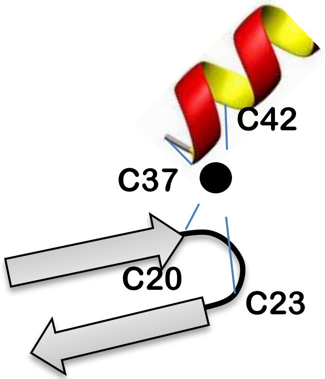

Ala-tRNA synthetase
Sites: 5 (1)
Zn Function: Structural

3eb5 (1001-A)

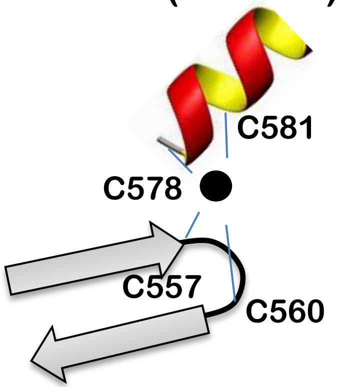

Apoptosis inhibitor 2
Sites: 26 (6)
Zn Function: Structural

3ebe (500-A)

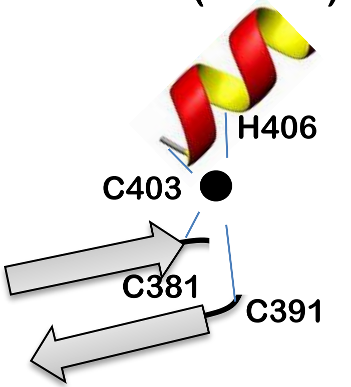

Protein MCM10 homolog
Sites: 5 (2)
Zn Function: Structural

3g9m (527-A)

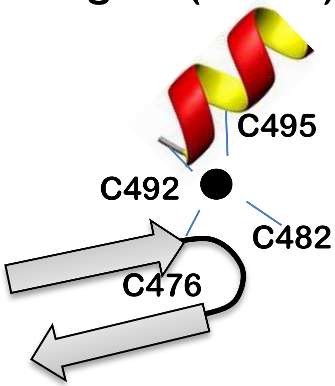

Glucocorticoid receptor
Sites: 100 (8)
Zn Function: Unknown

3gl6 (3-A)

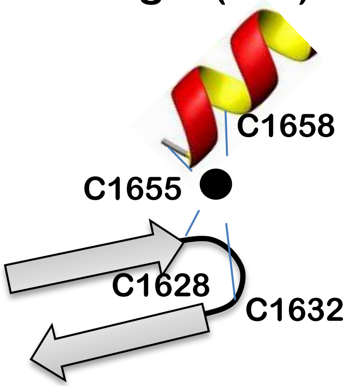

Lysine-specific demethylase 5A
Sites: 11 (2)
Zn Function: Structural

3gox (301-A)

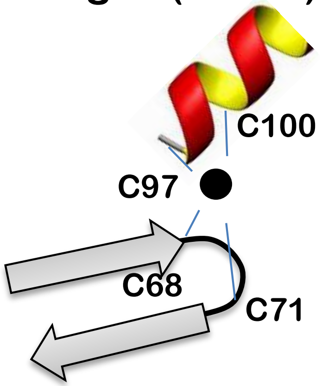

Putative uncharacterized protein
Sites: 4 (1)
Zn Function: Structural

3h0n(201-A)

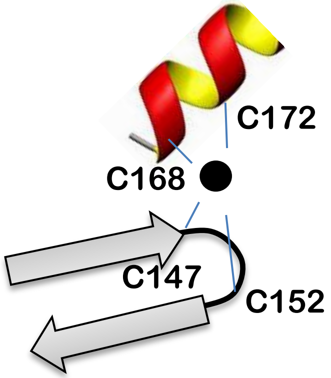

Putative uncharacterized protein
Sites: 1 (1)
Zn Function: Unknown

3kno(1-4)

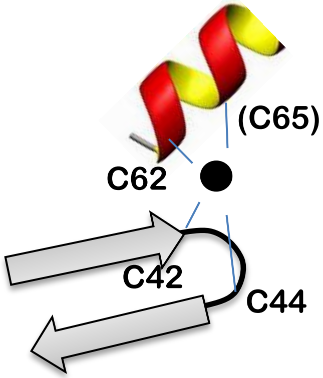

50S ribosomal protein L31
Sites: 4 (1)
Zn Function: Structural

Treble Clefs

3pt9 (2-A)

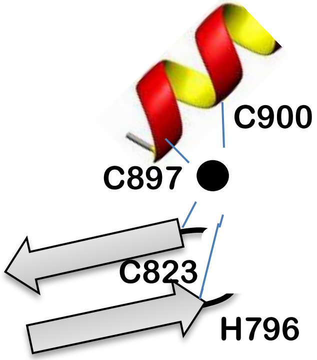

DNA (cytosine-5)-methyltransferase 1
Sites: 4 (1)
Zn Function: Structural

Zinc Necklaces

1a73 (201-A)

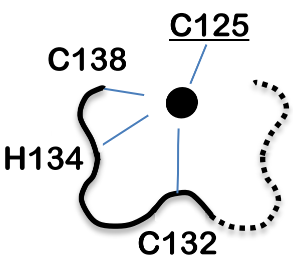

Intron-encoded endonuclease I-PpoI
Sites: 16 (1)
Zn Function: Structural

1a73 (202-A)

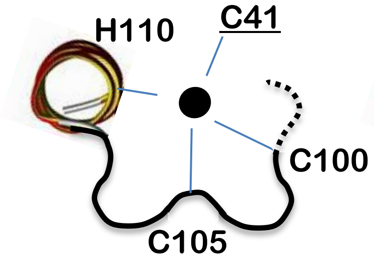

Intron-encoded endonuclease I-PpoI
Sites: 16 (1)
Zn Function: Structural

1f81 (88-A)

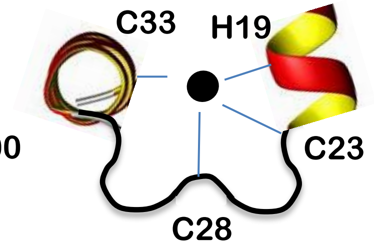

CREB-binding protein
Sites: 30 (2)
Zn Function: Structural

1ia9 (2001-A)

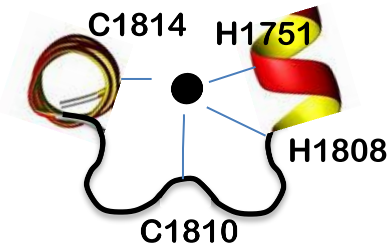

Channel-kinase 1
Sites: 16 (2)
Zn Function: Structural

1kwg (806-A)

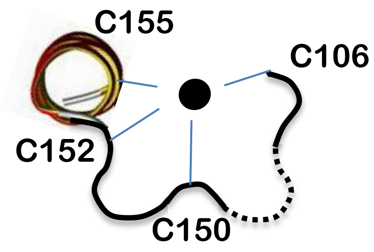

Beta-galactosidase
Sites: 2 (1)
Zn Function: Structural

1lpv (53-A)

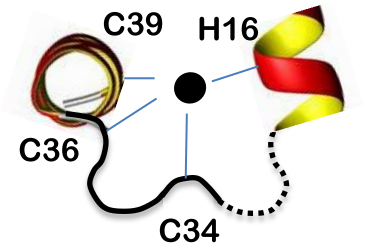

Protein doublesex
Sites: 1 (1)
Zn Function: Structural

1m9o (1-A)

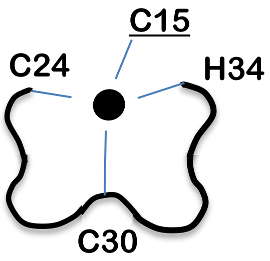

Tristetraprolin
Sites: 6 (3)
Zn Function: Structural

1ml9 (1-A,2-A,3-A)

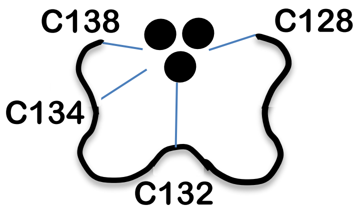

Histone H3-K9 methyltransferase dim-5
Sites: 38 (8)
Zn Function: Unknown

1mwz (75-A)

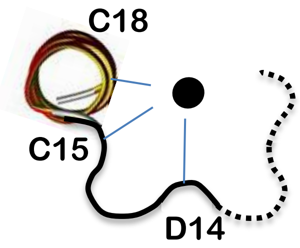

Pb-, Cd, Zn and Hg-transporting ATPase
Sites: 1 (1)
Zn Function: Substrate

1n8k (376-A)

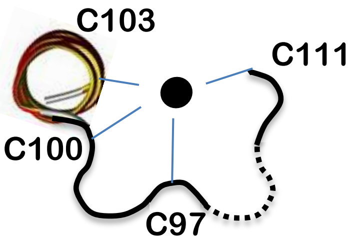

Alcohol dehydrogenase E chain
Sites: 236 (19)
Zn Function: Structural

Zinc Necklaces

1oqj (183-A)

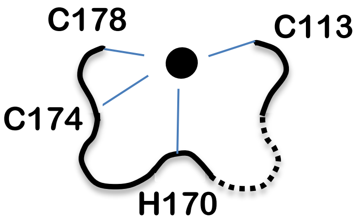

Glucocorticoid modulatory element-binding protein 1
Sites: 2 (1)
Zn Function: Structural

1oyw (801-A)

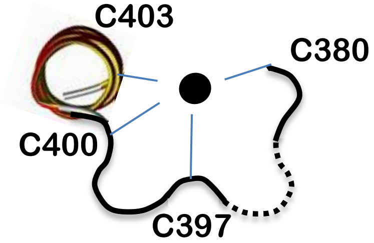

ATP-dependent DNA helicase recQ
Sites: 57 (4)
Zn Function: Structural

1p6o (400-B)

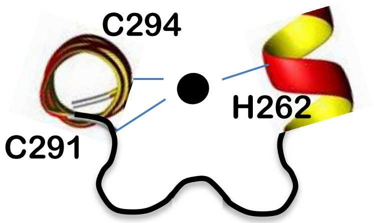

Cytosine deaminase
Sites: 150 (24)
Zn Function: Catalytic

1q08 (401-A,402-A)

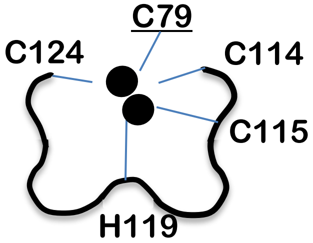

Zn(II)-responsive Regulator of zntA
Sites: 5 (2)
Zn Function: Regulatory

1r5y (400-A)

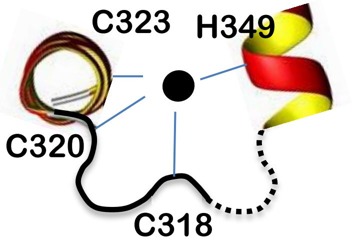

Queuine tRNA-ribosyltransferase
Sites: 75 (3)
Zn Function: Structural

1rni (201-A)

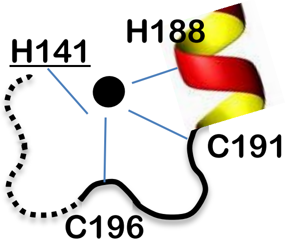

ORF904
Sites: 4 (1)
Zn Function: Structural

1svm (700-A)

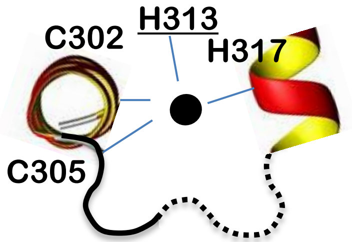

Large T antigen
Sites: 24 (1)
Zn Function: Structural

1t3k (201-A)

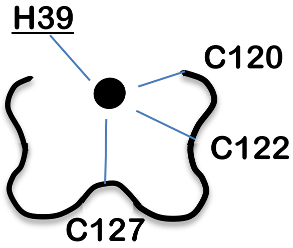

Dual specificity phosphatase Cdc25
Sites: 1 (1)
Zn Function: Structural

1t9h (411-A)

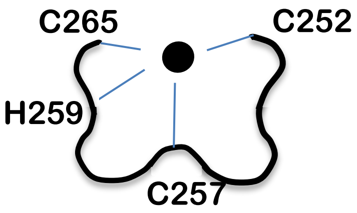

Putative ribosome biogenesis GTPase RsgA
Sites: 6 (4)
Zn Function: Structural

1twf (3002-C)

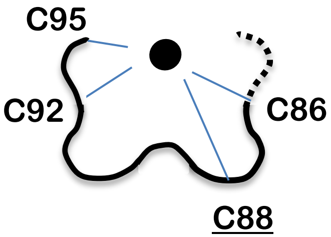

DNA-directed RNA polymerase II subunit RPB3
Sites: 60 (2)
Zn Function: Structural

Zinc Necklaces

1u2w (501-A)

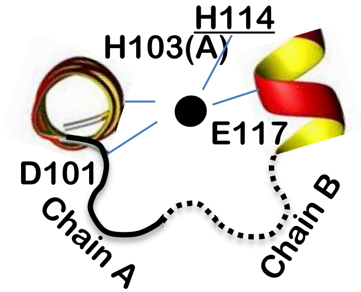

Cd/Zn- efflux system accessory protein
Sites: 7 (3)
Zn Function: Regulatory

1vsr (201-A)

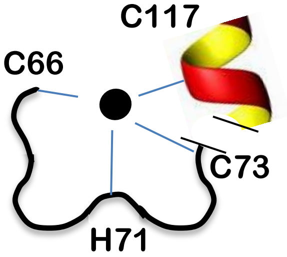

Very short patch repair protein
Sites: 3 (1)
Zn Function: Structural

1xto (100-A)

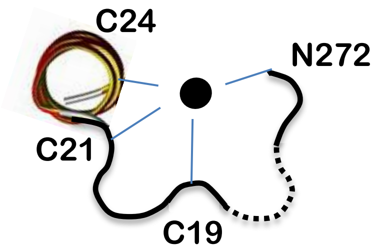

Coenzyme PQQ synthesis protein B
Sites: 6 (2)
Zn Function: Unknown

1z3i (900-X)

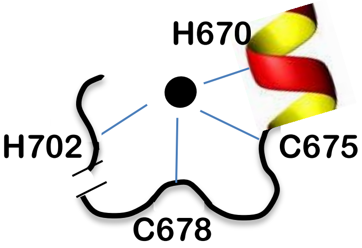

RAD54-like
Sites: 1 (1)
Zn Function: Structural

2a5h (421-A)

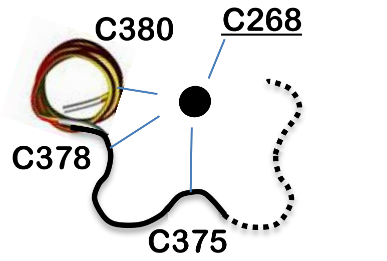

L-lysine 2,3-aminomutase
Sites: 4 (1)
Zn Function: Structural

2a6h (7412-D)

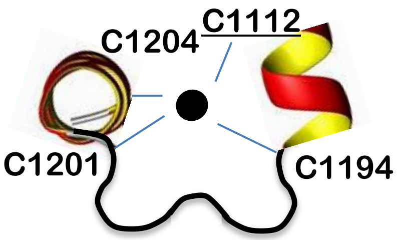

DNA-directed RNA polymerase subunit β
Sites: 33 (2)
Zn Function: Structural

2b5l (3001-C)

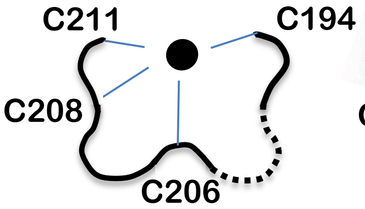

Non-structural protein V
Sites: 3 (1)
Zn Function: Structural

2bjr (1369-A)

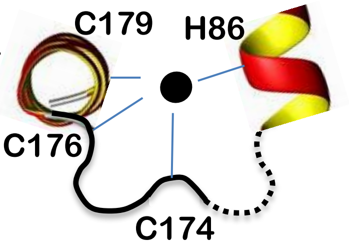

MFP2b
Sites: 2 (1)
Zn Function: Unknown

2e5s (401-A)

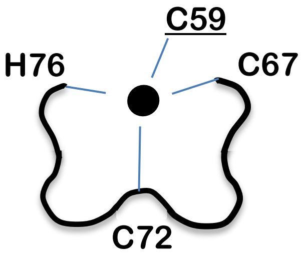

Muscleblind-like protein 2
Sites: 29 (4)
Zn Function: Structural

2f44 (255-A)

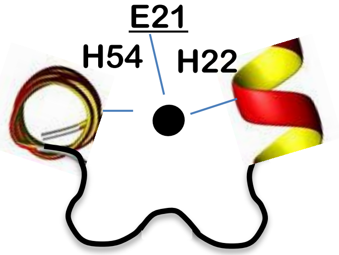

Proline-rich synapse-associated protein 2
Sites: 3 (1)
Zn Function: Structural

Zinc Necklaces

2fea (1302-A)

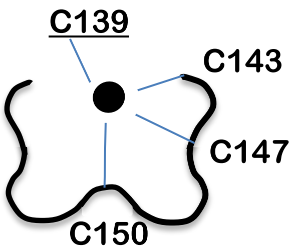

HK-MTPenyl-1-P phosphatase
\# Sites: 2 (1)
Zn Function: Structural

2fyg (302-A)

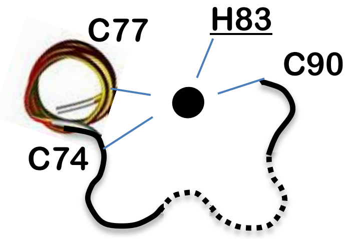

Replicase polyprotein 1a
\# Sites: 49 (1)
Zn Function: Structural

2glz (200-A)

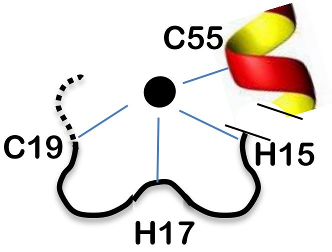

Formylmethanofuran dehydrogenase subunit E region
\# Sites: 3 (2)
Zn Function: Unknown

2iwj (1050-A)

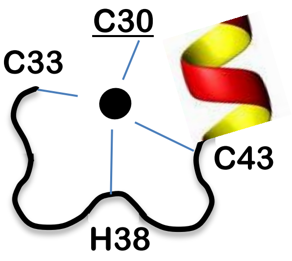

Gag-Pol polyprotein
\# Sites: 1 (1)
Zn Function: Structural

2j2s (2215-A)

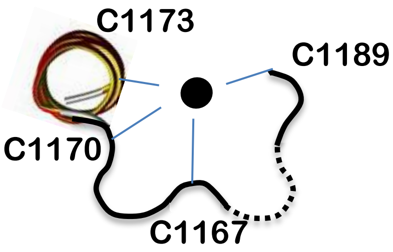

Histone-lysine N-methyltransferase MLL
\# Sites: 6 (1)
Zn Function: Structural

2kak (150-A, 170-A,190-A)

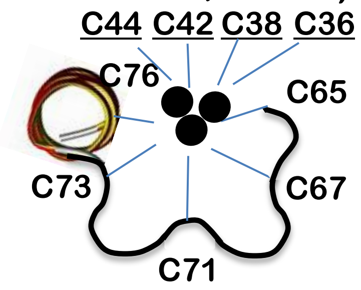

Zinc metallothionein class II
\# Sites: 1 (1)
Zn Function: Substrate

2kr1 (65-A)

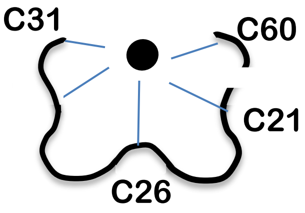

Ubiquitin-protein ligase E3A
\# Sites: 1 (1)
Zn Function: Unknown

2pg3 (300-A)

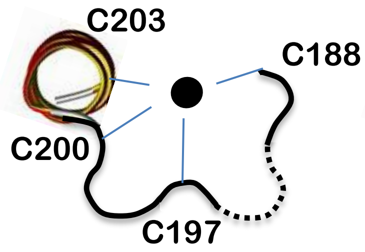

7-cyano-7-deazaguanine synthase
\# Sites: 7 (1)
Zn Function: Unknown

2pkg (175-C)

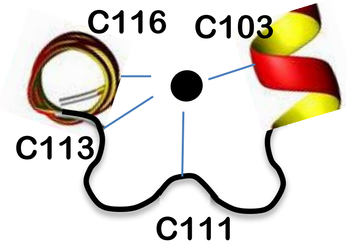

Medium tumor antigen-associated 61 kDa protein
\# Sites: 12 (1)
Zn Function: Structural

2rfi (504-A)

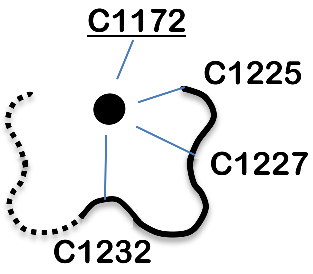

Histone-lysine N-methyltransferase EHMT1
\# Sites: 33 (6)
Zn Function: Unknown

Zinc Necklaces

2yre (501-A)

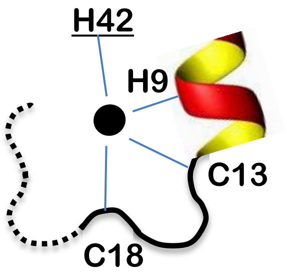

F-box only protein 30
Sites: 1 (1)
Zn Function: Unknown

3c0y (404-B)

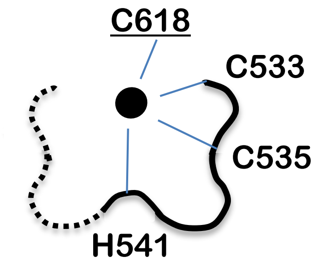

Histone deacetylase 7
Sites: 12 (1)
Zn Function: Structural

3f07 (409-A)

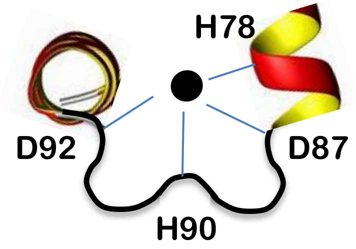

Histone deacetylase 8
Sites: 1 (1)
Zn Function: Unknown

3hko (701-A)

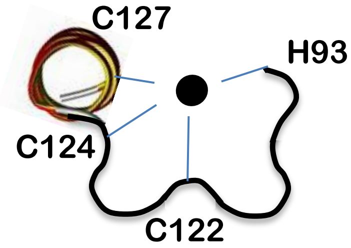

Ca/calmodulin-dependent protein kinase
Sites: 1 (1)
Zn Function: Unknown

3l0a (266-A)

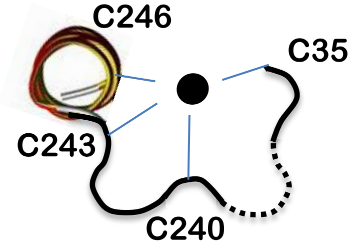

Putative uncharacterized protein
Sites: 1 (1)
Zn Function: Unknown

3mhs (475-A)

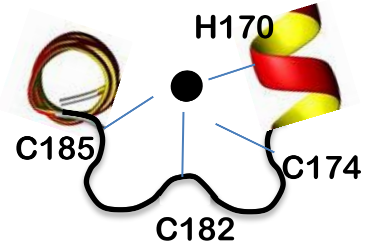

Ubiquitin carboxyl-terminal hydrolase 8
Sites: 3 (1)
Zn Function: Structural

3mhs (476-A)

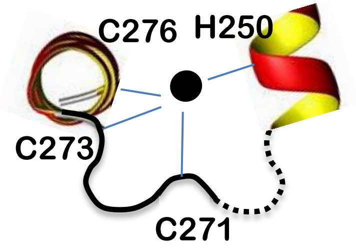

Ubiquitin carboxyl-terminal hydrolase 8
Sites: 3 (1)
Zn Function: Structural

3mi9 (88-B/C)

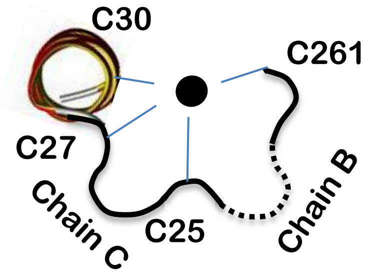

Protein Tat (C) / Cyclin-T1 (B)
Sites: 2 (1)
Zn Function: Unknown

3mln(501-A)

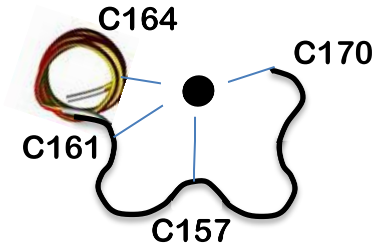

b-cell factor 1
Sites: 9 (1)
Zn Function: Structural

Zinc Rafts

1dyq (234-A)

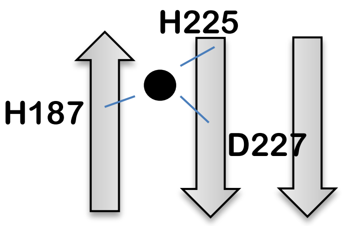

Enterotoxin type A
Sites: 15 (9)
Zn Function: Unknown

1ef0 (701-A)

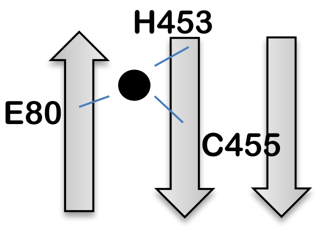

V-type proton ATPase catalytic subunit A
Sites: 2 (1)
Zn Function: Catalytic

1eh6 (200-A)

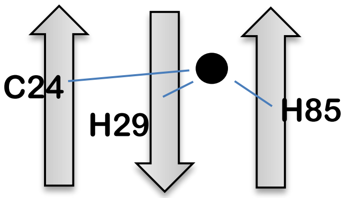

Methylated-DNA--protein-cysteine methyltransferase
Sites: 10 (1)
Zn Function: Structural

1enr (239-A)

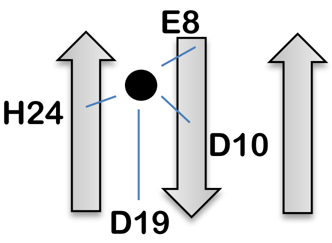

Concanavalin-A
Sites: 7 (1)
Zn Function: Regulatory

1evl (1-A)

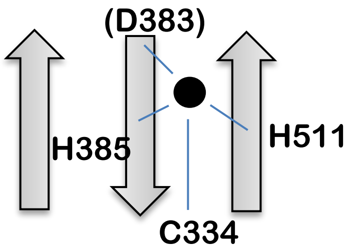

Threonyl-tRNA synthetase
Sites: 37 (5)
Zn Function: Catalytic

1i76 (998-A)

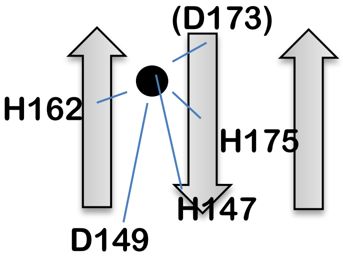

Neutrophil collagenase
Sites: 269 (4)
Zn Function: Structural

1im5 (400-A)

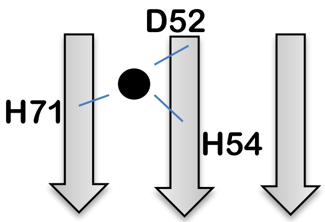

hypothetical pyrazinamidase nicotinamidase
Sites: 13 (5)
Zn Function: Catalytic

1lug (1001-A)

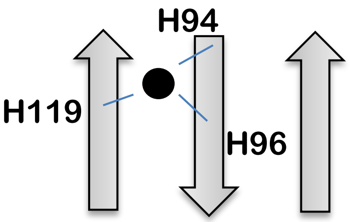

Carbonic anhydrase 2
Sites: 365 (8)
Zn Function: Catalytic

1m55 (201-B)

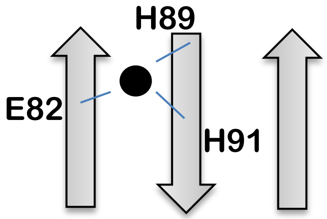

DNA binding trs helicase
Sites: 3 (2)
Zn Function: Catalytic

1oi0 (1122-A)

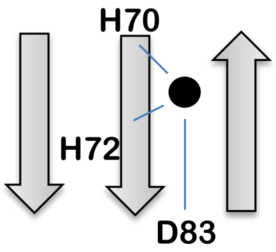

Putative uncharacterized protein
Sites: 7 (2)
Zn Function: Catalytic

Zinc Rafts

1qwy (400-A)

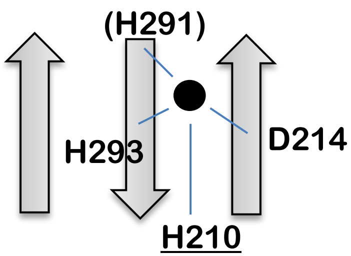

Glycyl-glycine endopeptidase lytM
Sites: 13 (2)
Zn Function: Catalytic

1r61 (1001-A)

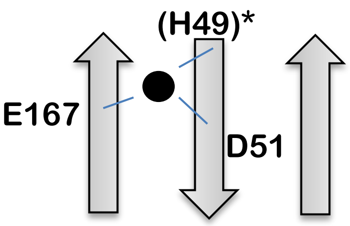

Hydrolase
Sites: 3 (1)
Zn Function: Unknown

1sr9 (703-B)

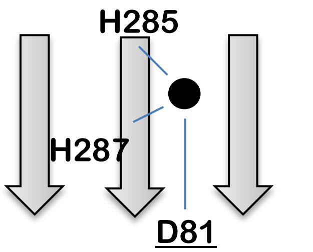

2-isopropylmalate synthase
Sites: 18 (5)
Zn Function: Catalytic

1t0a (661-A)

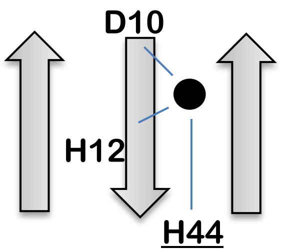

2-C-methyl-D-erythritol 2,4-cyclodiphosphate synthase
Sites: 97 (7)
Zn Function: Catalytic

1thj (214-A)

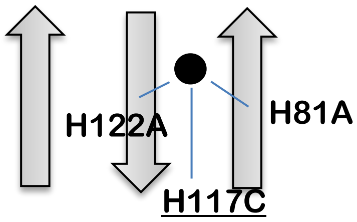

Carbonic anhydrase
Sites: 17 (5)
Zn Function: Catalytic

1txl (216-A)

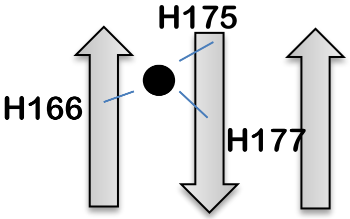

Metal-binding protein ZinT
Sites: 3 (1)
Zn Function: Unknown

1vhh (400-A)

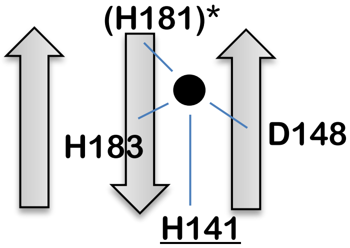

Sonic hedgehog protein
Sites: 42 (7)
Zn Function: Catalytic

1yg9 (401-A)

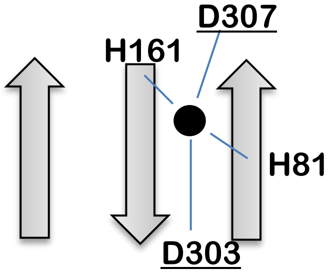

Aspartic protease Bla g 2
Sites: 4 (1)
Zn Function: Structural

1yt3 (1001-A)

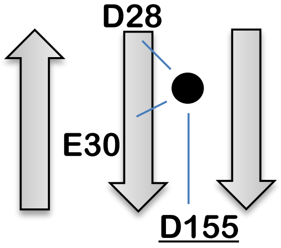

Ribonuclease D
Sites: 2 (1)
Zn Function: Catalytic

1zsw (1-A)

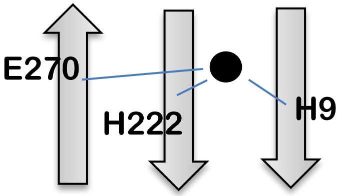

Glyoxalase family protein
Sites: 30 (9)
Zn Function: Catalytic

Zinc Rafts

2aq2 (1001-B)

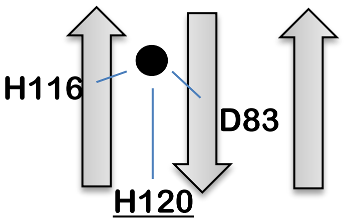

T-cell receptor beta chain V region C5
Sites: 22 (2)
Zn Function: Unknown

2cs7 (203-C)

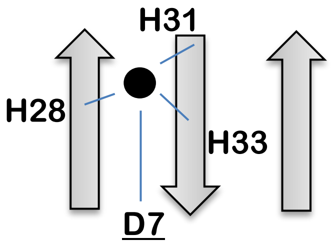

Conserved domain protein
Sites: 3 (1)
Zn Function: Unknown

2faw (1001-A)

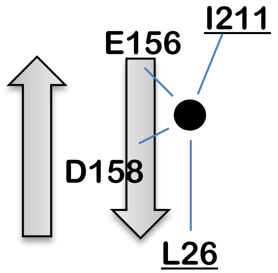

Glutamine cyclotransferase
Sites: 2 (1)
Zn Function: Structural

2fgy (721-B)

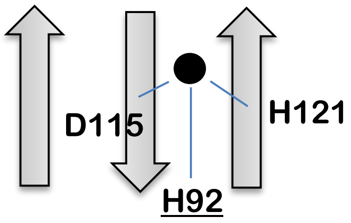

Carboxysome shell polypeptide
Sites: 2 (1)
Zn Function: Catalytic

2fli (1982-B)

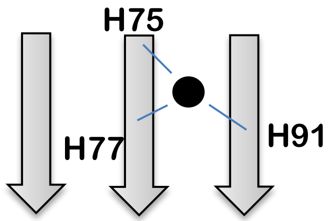

Ribulose-phosphate 3-epimerase
Sites: 16 (3)
Zn Function: Catalytic

2g64 (2001-A)

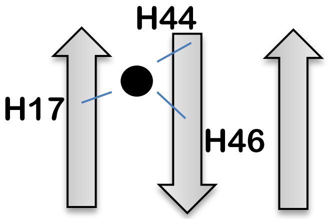

Putative 6-pyruvoyl tetrahydrobiopterin synthase
Sites: 26 (5)
Zn Function: Catalytic

2hsi (1-A)

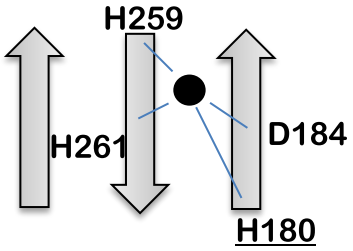

Putative uncharacterized protein
Sites: 6 (2)
Zn Function: Catalytic

2ijd (1-1)

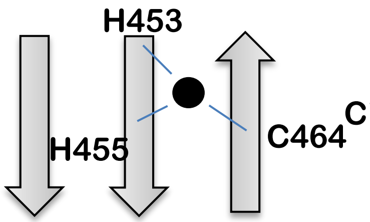

Genome polyprotein
Sites: 24 (1)
Zn Function: Structural

2j7u (1884-A)

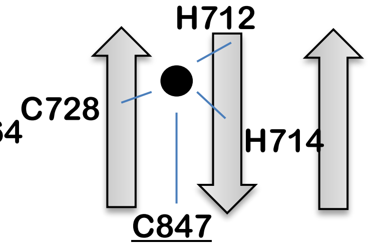

Polyprotein
Sites: 2 (1)
Zn Function: Structural

2jox (110-A)

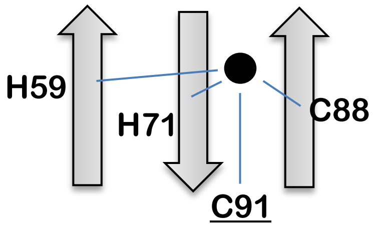

Protein Churchill
Sites: 1 (1)
Zn Function: Structural

Zinc Rafts

2l0z (486-A)

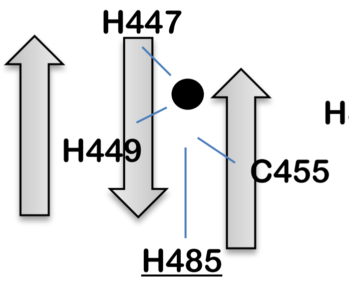

Pre-glycoprotein polyprotein GP complex
Sites: 1 (1)
Zn Function: Unknown

2nly (300-A)

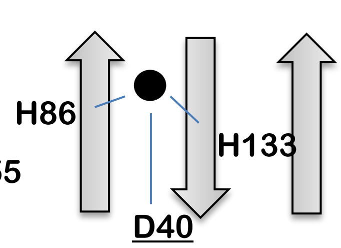

BH1492 protein
Sites: 7 (2)
Zn Function: Catalytic

2o03 (201-A)

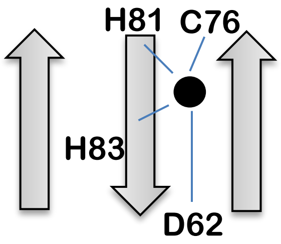

Ferric uptake regulation protein
Sites: 1 (1)
Zn Function: Regulatory

2o03 (203-A)

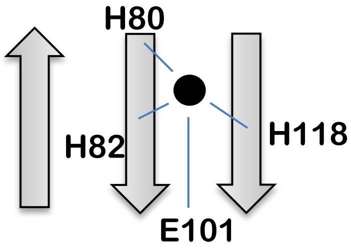

Ferric uptake regulation protein
Sites: 2 (2)
Zn Function: Regulatory

2o1q (1-A)

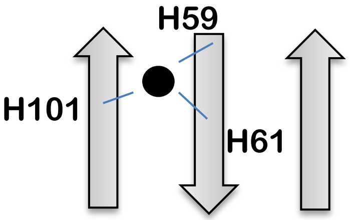

Putative acetyl/propionyl-coa carboxylase
Sites: 50 (15)
Zn Function: Catalytic

2oog (401-B)

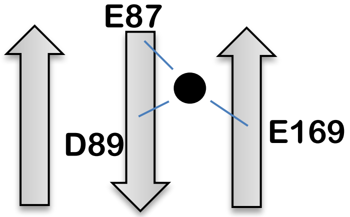

Glycerophosphoryl diester phosphodiesterase
Sites: 6 (1)
Zn Function: Unknown

2oso (1-A)

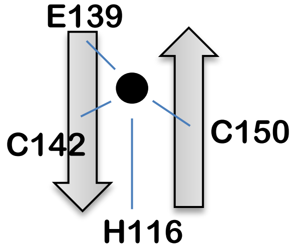

Uncharacterized protein MJ1460
Sites: 2 (1)
Zn Function: Unknown

2p6y (201-A)

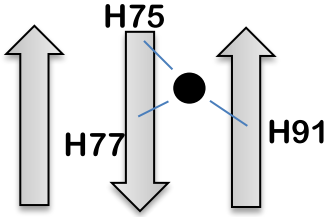

Putative uncharacterized protein
Sites: 10 (4)
Zn Function: Catalytic

2peb (200-A)

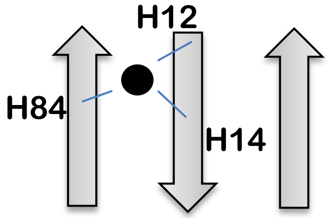

Putative dioxygenase
Sites: 2 (1)
Zn Function: Unknown

2q1z (197-B)

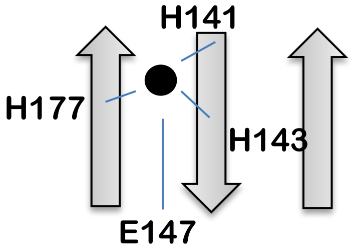

Sigma24, RpoE
Sites: 2 (1)
Zn Function: Unknown

Zinc Rafts

2v9l (1275-A)

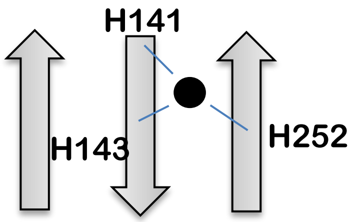

Rhamnulose-1-phosphate aldolase
Sites: 78 (5)
Zn Function: Catalytic

3bq5 (800-A)

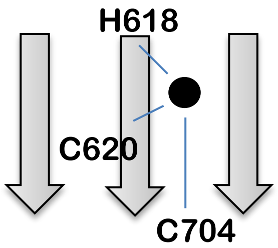

Cobalamin-independent methionine synthase
Sites: 11 (2)
Zn Function: Catalytic

3byr (501-A, 502-A)

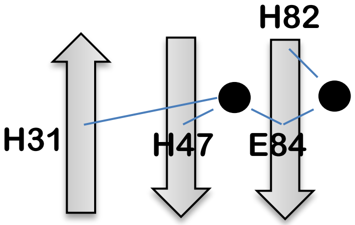

CzrB protein
Sites: 15 (3)
Zn Function: Substrate

3chv (302-A)

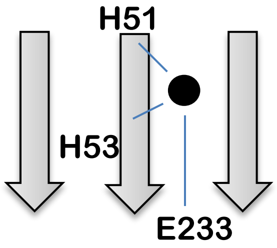

Putative uncharacterized protein
Sites: 16 (2)
Zn Function: Unknown

3hka (429-C)

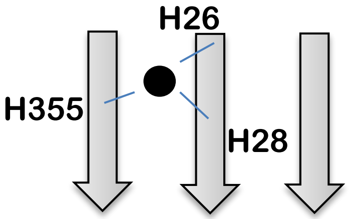

BH0493 protein
Sites: 58 (1)
Zn Function: Catalytic

C2H2 Zinc Fingers

1ej6 (2000-C)

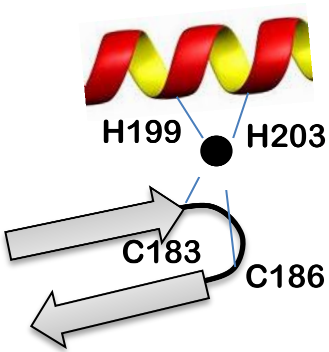

Inner capsid protein lambda-1
Sites: 1 (1)
Zn Function: Structural

1k2f (606-B)

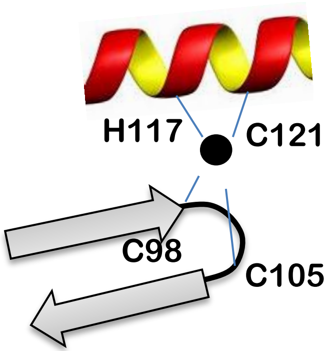

E3 ubiquitin-protein ligase SIAH1A
Sites: 19 (4)
Zn Function: Structural

1k6y (401-A)

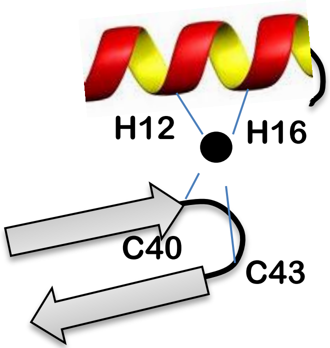

Gag-Pol polyprotein
Sites: 73 (5)
Zn Function: Structural

1llm (301-C)

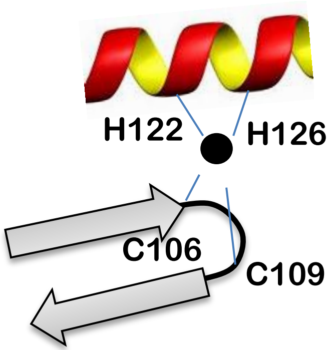

General control protein GCN4
Sites: 385 (75)
Zn Function: Structural

1pi1 (101-A)

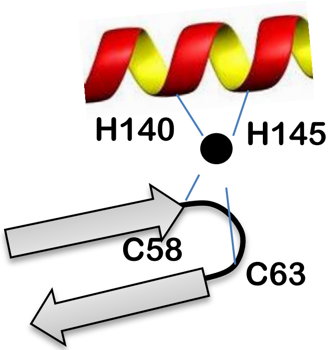

Mps one binder kinase activator-like 1B
Sites: 2 (2)
Zn Function: Structural

1wur (1001-A)

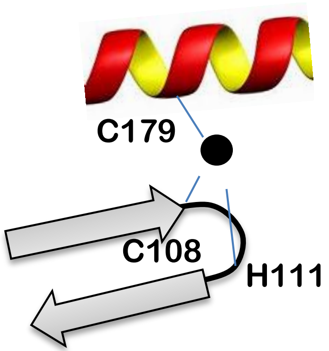

GTP cyclohydrolase 1
Sites: 55 (5)
Zn Function: Catalytic

1zw8 (66-A)

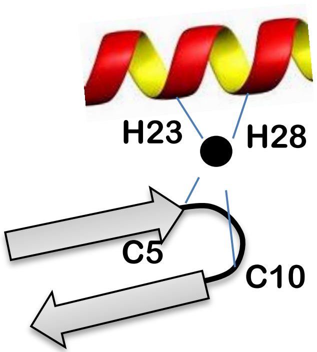

Zinc-responsive transcriptional regulator ZAP1
Sites: 6 (2)
Zn Function: Structural

2bai (110-A)

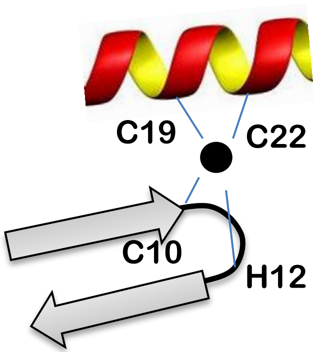

Genome polyprotein
Sites: 1 (1)
Zn Function: Structural

2csv (400-A)

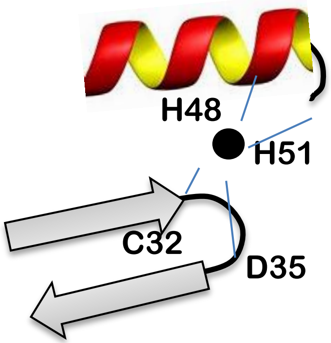

Tripartite motif-containing protein 29
Sites: 11 (6)
Zn Function: Structural

2dip (401-A)

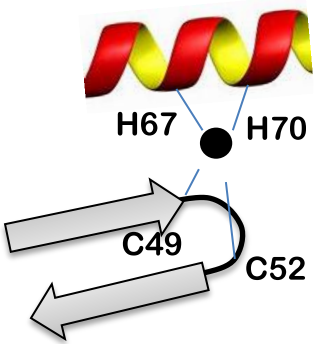

E3 ubiquitin-protein ligase ZSWIM2
Sites: 36 (5)
Zn Function: Structural

C2H2 Zinc Fingers

2dkt (241-A)

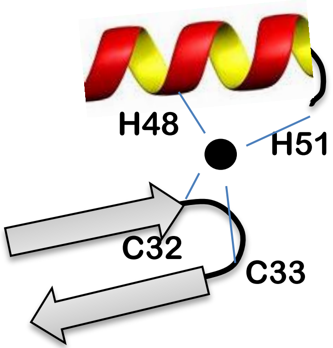

Androgen receptor N-terminal-interacting protein
Sites: 2 (1)
Zn Function: Structural

2dmi (300-A)

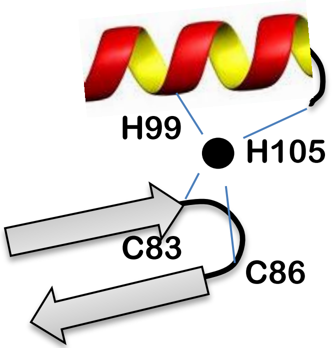

Teashirt homolog 3
Sites: 2 (1)
Zn Function: Structural

2giv (501-A)

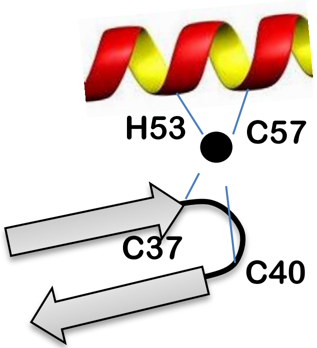

Probable histone acetyltransferase MYST1
Sites: 5 (1)
Zn Function: Structural

2k9h (101-A)

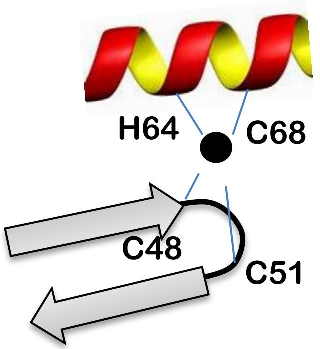

Glycoprotein
Sites: 2 (1)
Zn Function: Structural

2q1z (196-B)

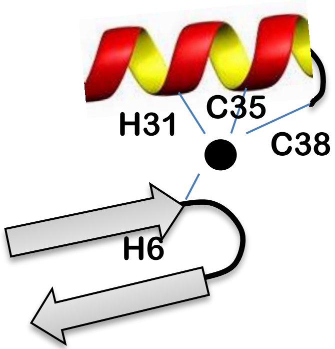

Sigma24, RpoE
Sites: 16 (2)
Zn Function: Structural

2qfa (143-A)

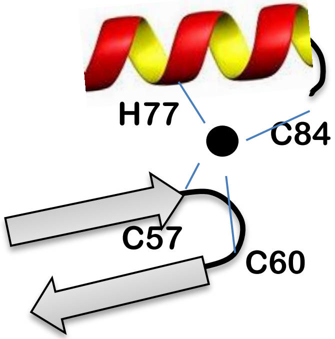

Baculoviral IAP repeat-containing protein 5
Sites: 140 (11)
Zn Function: Structural

2wjy (2-A)

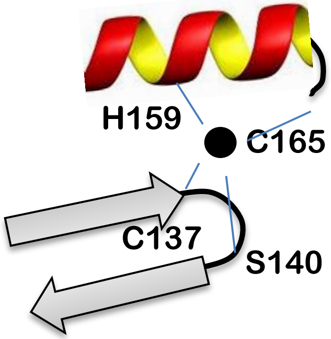

Regulator of nonsense transcripts 1
Sites: 5 (1)
Zn Function: Structural

3c5k (201-A)

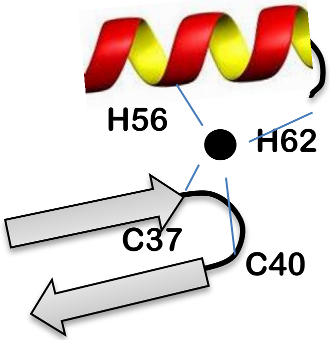

Histone deacetylase 6
Sites: 8 (5)
Zn Function: Structural

3eph (1-A)

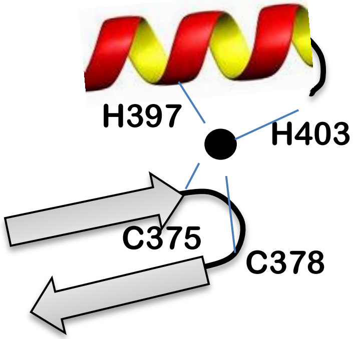

tRNA dimethylallyltransferase, mitochondrial
Sites: 9 (2)
Zn Function: Structural

3hct (303-A)

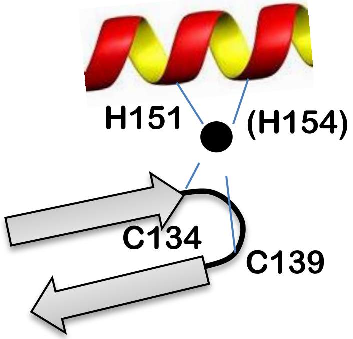

TNF receptor-associated factor 6
Sites: 11 (3)
Zn Function: Structural

Loosened Zinc Ribbons

1bor (58-A)

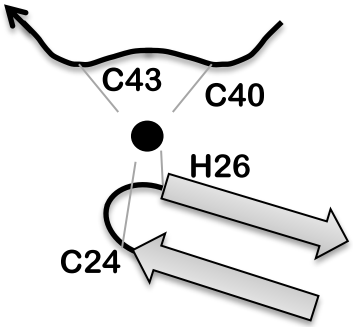

Protein PML
Sites: 4 (4)
Zn Function: Structural

1dy0 (401-A)

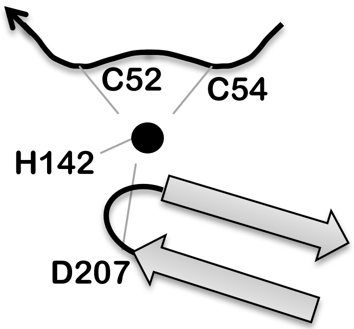

Collagen alpha-1(XVIII) chain
Sites: 6 (1)
Zn Function: Structural

1fn9 (1001-A)

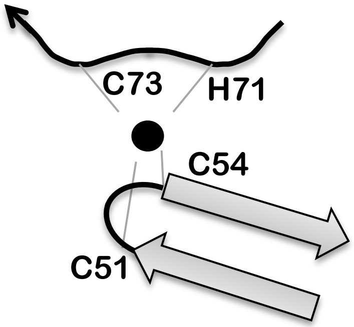

Outer capsid protein sigma-3
Sites: 5 (1)
Zn Function: Structural

1mr1 (601-C)

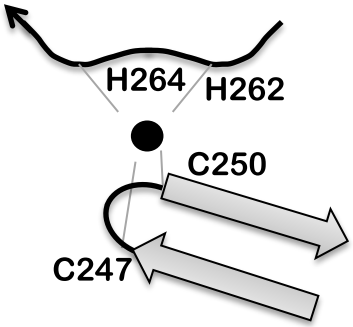

Ski oncogene
Sites: 2 (1)
Zn Function: Structural

1odh (1171-A)

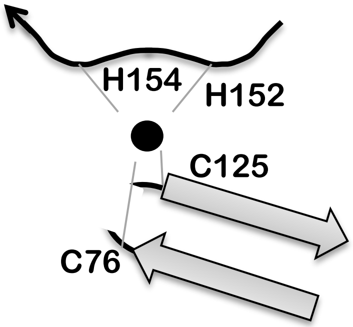

Chorion-specific transcription factor GCMa
Sites: 1 (1)
Zn Function: Structural

1ptq (1-A)

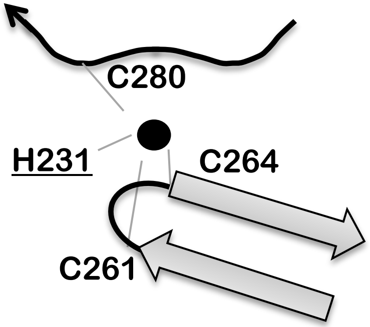

Protein kinase C delta type
Sites: 27 (13)
Zn Function: Structural

1q68 (201-A)

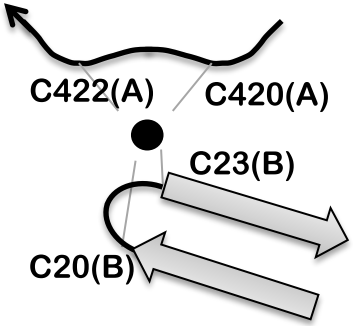

T-cell surface glycoprotein CD4
Sites: 2 (2)
Zn Function: Structural

1twf (3008-A)

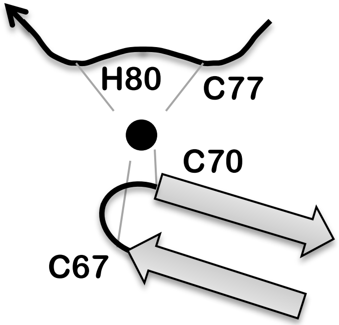

DNA-directed RNA polymerase II subunit RPB1
Sites: 75 (5)
Zn Function: Structural

1v54 (401-F)

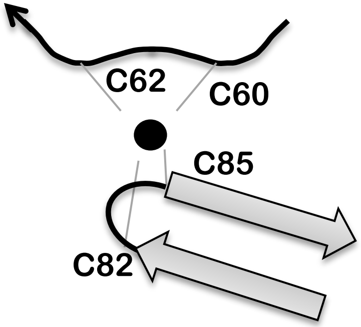

Cytochrome c oxidase polypeptide Via
Sites: 44 (1)
Zn Function: Structural

1vzy (1291-A)

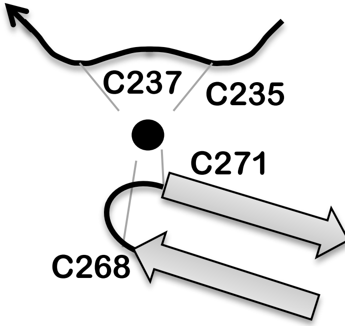

33 kDa chaperonin
Sites: 6 (3)
Zn Function: Regulatory

Loosened Zinc Ribbons

1wj2 (470-A)

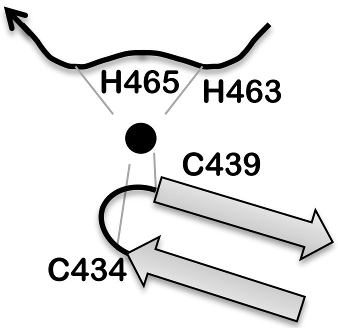

Probable WRKY transcription factor 4
Sites: 3 (2)
Zn Function: Structural

2dkt (441-A)

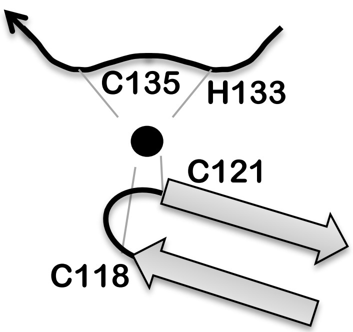

Androgen receptor N-terminal-interacting protein
Sites: 4 (1)
Zn Function: Structural

2fyg (303-A)

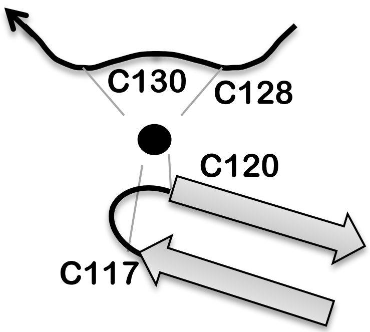

Replicase polyprotein 1a
Sites: 50 (2)
Zn Function: Structural

2gmw (300-A)

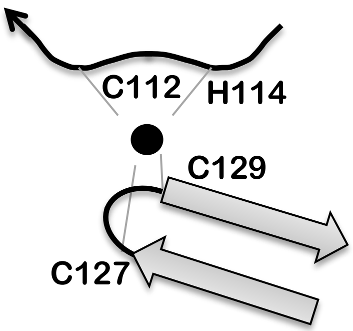

D,D-heptose 1,7-bisphosphate phosphatase
Sites: 26 (3)
Zn Function: Structural

2hrv (143-A)

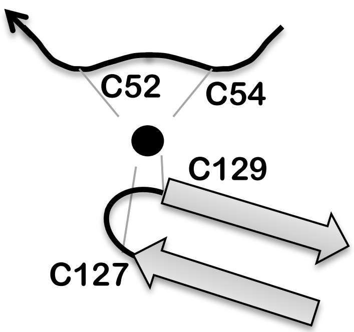

Genome polyprotein
Sites: 88 (3)
Zn Function: Structural

3ifu (182-A)

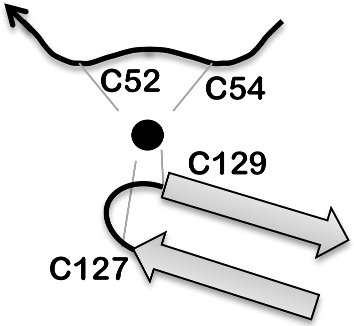

Non-structural protein
Sites: 1 (1)
Zn Function: Structural

Helical Anchors

1au1 (1-B)

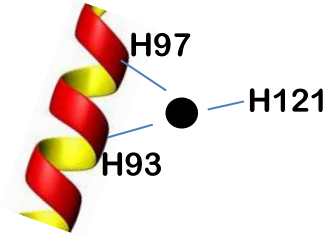

Interferon beta
Sites: 1 (1)
Zn Function: Structural

1c7k(133-A)

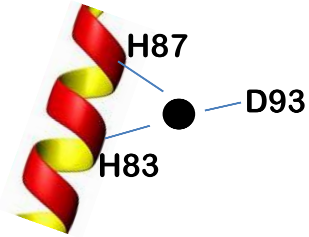

Extracellular small neutral protease
Sites: 758 (74)
Zn Function: Catalytic

1fr2 (301-B)

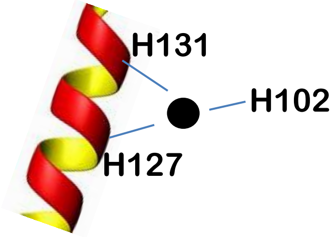

Colicin-E9 immunity protein#
Sites: 35 (1)
Zn Function: Catalytic

1j98 (300-A)

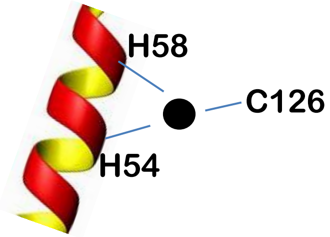

S-ribosylhomocysteine lyase
Sites: 66 (15)
Zn Function: Catalytic

1oek (1195-A)

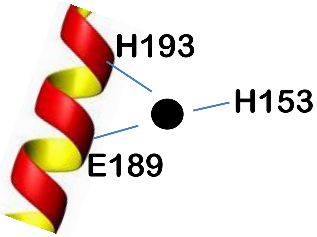

Metal-binding protein ZinT
Sites: 1 (1)
Zn Function: Unknown

2ves (1295-A)

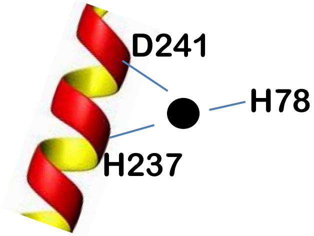

UDP-3-O-[3-hydroxymyristoyl] N-acetylglucosamine deacetylase
Sites: 26 (2)
Zn Function: Catalytic

1sg0 (231-A)

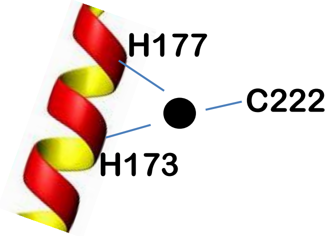

Quinone reductase 2
Sites: 31 (1)
Zn Function: Catalytic

1u0b (201-B)

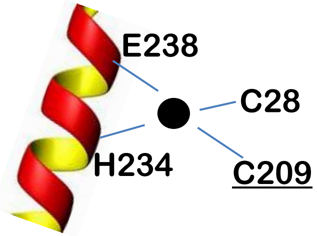

Cysteinyl-tRNA synthetase
Sites: 7 (2)
Zn Function: Catalytic

1v4p (1002-B)

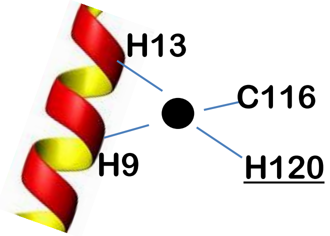

Alanyl-tRNA deacylase AlaX-S
Sites: 15 (4)
Zn Function: Catalytic

2ce7 (1603-A)

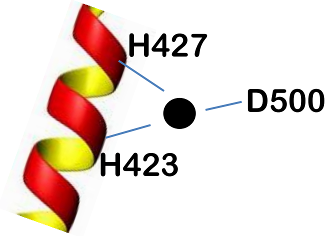

ATP-dependent zinc metalloprotease FtsH
Sites: 17 (2)
Zn Function: Catalytic

Helical Anchors

2j7u (1885-A)

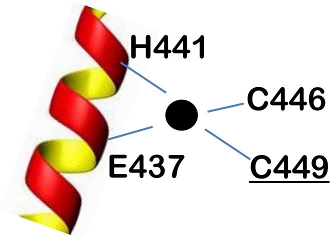

Polyprotein
Sites: 7 (1)
Zn Function: Unknown

2psr (103-A)

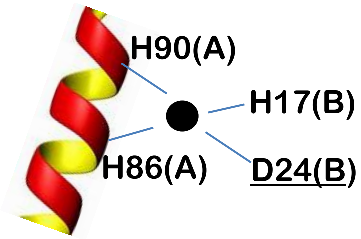

Protein S100-A7
Sites: 20 (5)
Zn Function: Regulatory

2zh0 (4002-B)

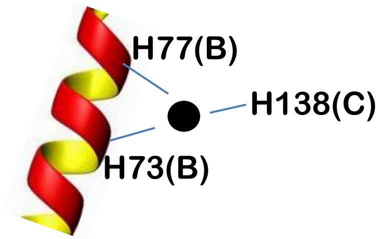

Hut operon positive regulatory protein
Sites: 12 (1)
Zn Function: Unknown

3fvy (1000-A)

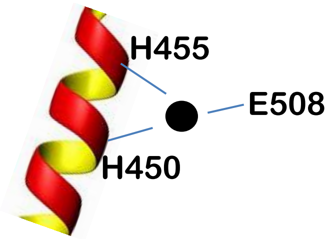

Dipeptidyl peptidase 3
Sites: 2 (2)
Zn Function: Unknown

3hwp (295-A)

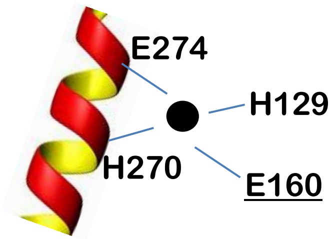

PhlG
Sites: 2 (1)
Zn Function:Catalytic

Shuffled Zinc Ribbons – type I

1adn (93-A)

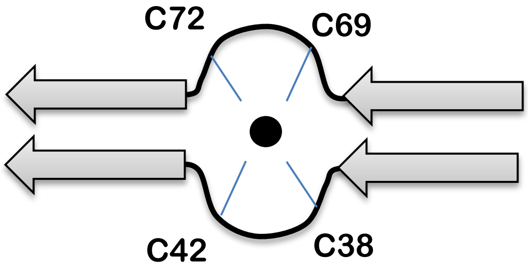

Bifunctional transcriptional activator/DNA repair enzyme Ada
Sites: 5 (1)
Zn Function: Catalytic

1nlt (351-A)

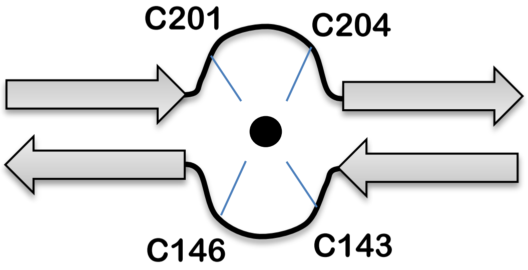

Mitochondrial protein import protein MAS5
Sites: 6 (3)
Zn Function: Structural

1p9r (601-A)

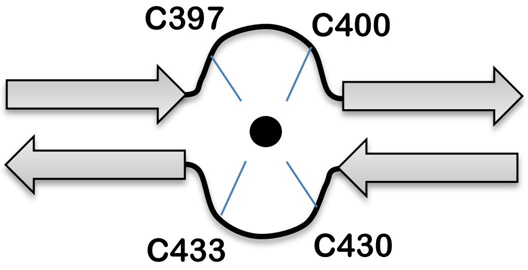

General secretion pathway protein E
Sites: 2 (1)
Zn Function:Unknown

2i2t (101-4)

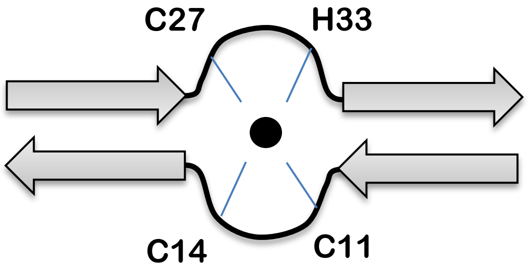

50S ribosomal protein L36
Sites: 42 (1)
Zn Function: Structural

2r6f (1005-A)

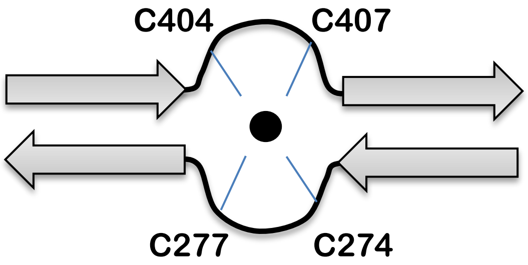

Excinuclease ABC subunit A
Sites: 14 (2)
Zn Function: Structural

2rhq (1-A)

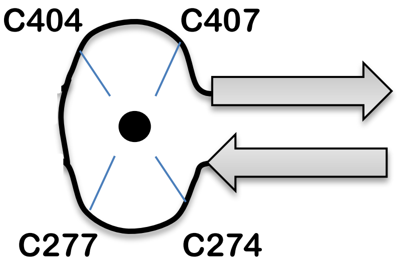

Phenylalanyl-tRNA synthetase alpha chain
Sites: 2 (1)
Zn Function: Structural

Shuffled Zinc Ribbons – type I

2vmk (1514-B)

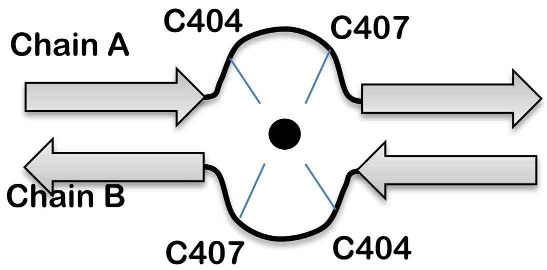

Ribonuclease E
Sites: 6 (2)
Zn Function: Structural

2zp8 (54-E)

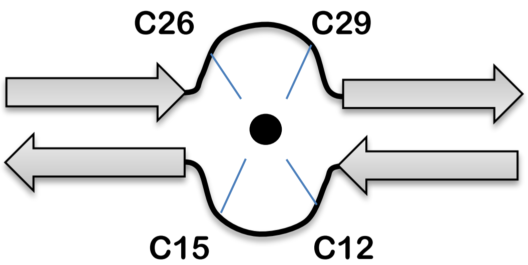

Tryptophan RNA-binding attenuator protein inhibitory protein
Sites: 78 (1)
Zn Function: Structural

Zn2Cys6 Zinc Fingers

1co4 (43-A)

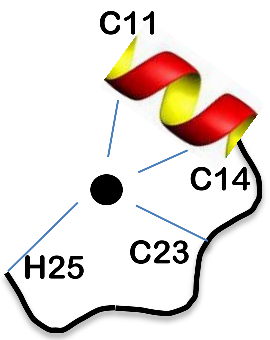

Metal-activated transcriptional activator protein AMT1
Sites: 1 (1)
Zn Function: Structural

1hwt (136-C,137-C)

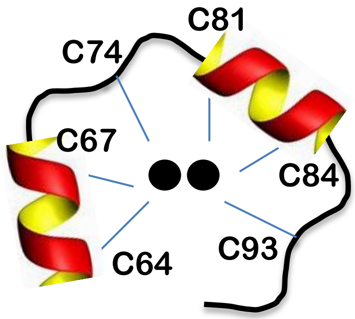

Heme-responsive zinc finger transcription factor HAP1
Sites: 31 (6)
Zn Function: Structural

1ldj (203-B)

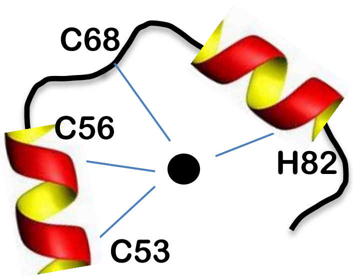

Cullin-1
Sites: 9 (3)
Zn Function: Structural

1lpv (54-A)

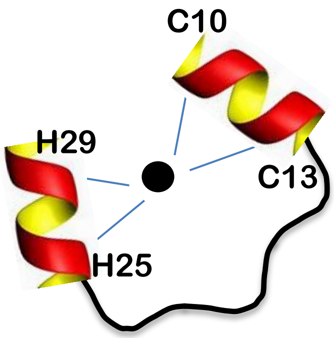

Protein doublesex
Sites: 1 (1)
Zn Function: Structural

1v33 (1000-A)

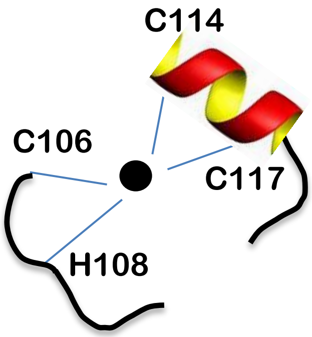

DNA primase small subunit
Sites: 4 (1)
Zn Function: Structural

2ox0 (502-A)

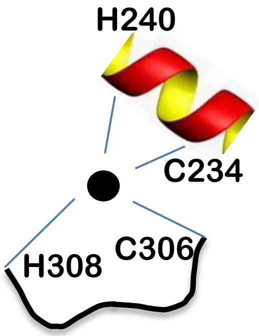

Lysine-specific demethylase 4A
Sites: 34 (1)
Zn Function: Structural

3h84 (355-A)

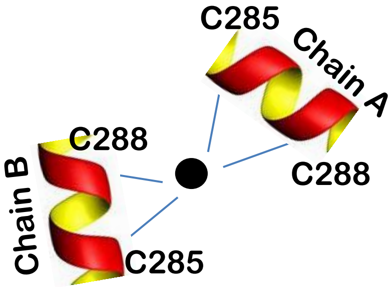

ATPase GET3
Sites: 8 (2)
Zn Function: Structural

Shuffled Zinc Ribbons – type II

1btk (1-A)

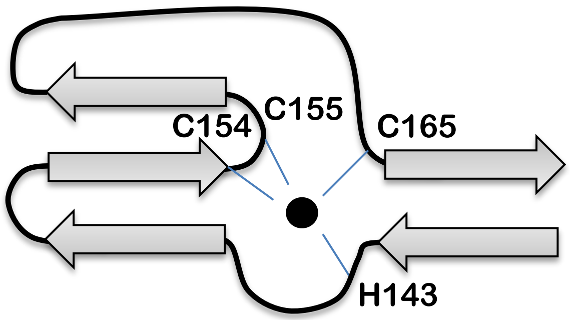

Tyrosine-protein kinase BTK
Sites: 11 (3)
Zn Function: Structural

2yrt (401-A)

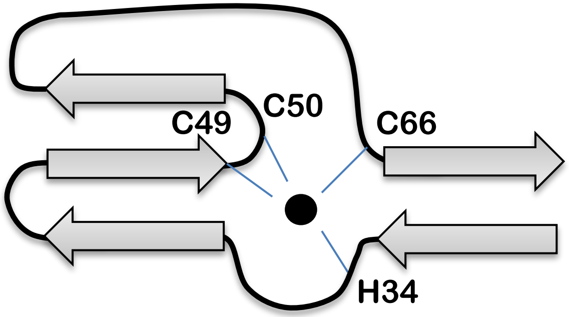

Cysteine and histidine-rich domain-containing protein 1
Sites: 3 (2)
Zn Function: Unknown
