# Supplementary material for: Minimal Functional Sites Allow a Classification of Zinc Sites in Proteins
Source: PLoS One. 2011 Oct 17;6(10):e26325. doi: 10.1371/journal.pone.0026325 (PMC3197139; doi:10.1371/journal.pone.0026325)
Supplement: Table S5 — Results of the clustering of representative Zn-sites using different distance threshold values for defining spatially proximal residues in building the MFS templates. (PDF) [file pone.0026325.s005.pdf]

Table S5. Results of the clustering of representative Zn-sites using different distance threshold values for defining spatially proximal residues in building the MFS templates. The clusters were built using the 99th percentile of all non-zero similarity scores obtained from FAST as the threshold similarity score in each case. The number of "correct" and "incorrect" clusters (and of the sites therein) is based on the final, manually refined, set of clusters, i.e., clusters containing only sites that belong to one and the same cluster in the final set are taken as "correct", and the others are "incorrect". Note that "correct" clusters may also be sub-clusters (i.e., parts) of the same final cluster.

**Distance threshold (Å)**	**# of correct clusters**	**# of sites in correct clusters**	**# of incorrect clusters**	**# of sites in incorrect clusters**
3.0	3	17	0	0
4.0	9	70	1	2
**5.0**	**6**	**74**	**0**	**0**
6.0	3	13	2	69
7.0	6	22	2	83
8.0	3	10	2	106
9.0	4	12	4	113
10.0	5	14	9	132
